# Molecular variants, clonal evolution and clinical relevance in pediatric and adult T-cell lymphoblastic neoplasia

**DOI:** 10.1038/s41408-026-01488-w

**Published:** 2026-04-02

**Authors:** Sarah Sandmann, Marcel te Vrugt, Gerrit Randau, Thomas Beder, Martin Neumann, Toni Lange, Amelie Alfert, Stephanie Mueller, Marc Hotfilder, Corinne Rossi, Cornelia Eckert, Anja Moericke, Johanna Maria Horns, Martin Zimmermann, Julian Varghese, Monika Brüggemann, Birgit Burkhardt

**Affiliations:** 1 Institute of Medical Data Science, Otto-von-Guericke-University, Magdeburg, Germany; 2 Pediatric Hematology and Oncology and NHL-BFM study center, University Hospital Muenster, Muenster, Germany; 3 A Clinical Research Unit “CATCH ALL” (KFO 5010/1) funded by the Deutsche Forschungsgemeinschaft, Kiel, Germany; 4 Department of Medicine II, Hematology and Oncology, University Hospital Schleswig-Holstein, Kiel, Germany; 5 Department of Psychiatry, University of Muenster, Muenster, Germany; 6 Clinic for Pediatric Oncology, Hematology, Immunology, and Pneumology, Center for Pediatric and Adolescent Medicine, University Hospital Heidelberg, Heidelberg, Germany; 7 Department of Pediatric Oncology/Hematology, Charité Universitaetsmedizin Berlin, Berlin, Germany; 8 Department of Pediatrics I, Pediatric Hematology/Oncology, ALL-BFM Study Group, Christian Albrechts University Kiel and University Hospital Schleswig-Holstein, Kiel, Germany; 9 Department of Pediatric Hematology and Oncology, Hannover Medical School, Hannover, Germany

**Keywords:** Cancer genetics, Cancer genomics, Cancer genetics

## Abstract

T-cell lymphoblastic lymphoma (T-LBL) and T-cell acute lymphoblastic leukemia (T-ALL) originate from thymic T-cell precursors, with ongoing debate on whether they are variants of the same disease or distinct entities. For 211 patients, including pediatric and adult T-ALL and T-LBL cases, targeted next-generation sequencing and SNP-arrays were performed, and single-nucleotide variants, indels and copy-number variants (CNVs) were analyzed. We aimed to assess genetic differences between T-ALL and T-LBL across age. Generally, mutational landscape analysis identified mutated *PHF6* being associated with higher, *NOTCH1* with lower age at diagnosis for both T-LBL and T-ALL. Association of CNVs with higher age was evident for T-ALL, but not T-LBL. Analysis of clonal evolution revealed that CNVs – especially deletions and LOH in chromosome 9 (LOH_in_9p) – were observed as first mutational event in both pediatric T-ALL and T-LBL. The sequence of genetic events, starting with LOH_in_9p followed by mutations in *NOTCH1*, was significantly more frequent in pediatric T-ALL and T-LBL. Detailed evaluation of the patients’ individual clonal evolution indicated that the proportion of malignant cells without *NOTCH*^*MT*^ determines the risk of relapse (hazard ratio 1.032, *p* = 4.65*10^−5^). In T-ALL, aside from MRD, validated molecular markers for risk-group stratification remain limited. Our data suggest that molecular metrics analogous to those in T-LBL may help refining risk stratification in T-ALL as well.

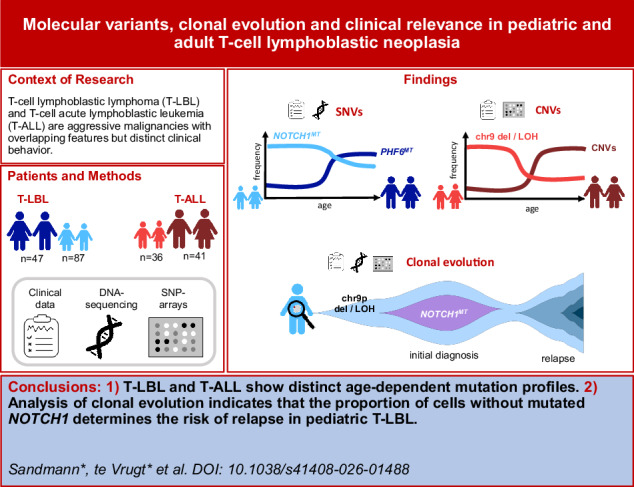

T-cell lymphoblastic lymphoma (T-LBL) and T-cell acute lymphoblastic leukemia (T-ALL) arise from malignant transformation of immature T-cell precursors. They are clinically distinguished by the extent of bone marrow (BM) infiltration at diagnosis, with T-ALL defined by ≥25% BM involvement. T-ALL represents 15% of childhood ALL cases, with current treatment protocols achieving an event-free survival (EFS) of ~85%, but relapse EFS is only ~20%. In adults, T-ALL comprises ~25% of ALL cases with a median age of 25–30 years and overall survival rates of 40–50%, again with poor outcomes at relapse [1, 2].

T-LBL is the second most common Non-Hodgkin lymphoma (NHL) subtype in children and adolescents, with a median onset age of 8.8 years and EFS rates of ~80%. Relapsed pediatric T-LBL patients have a poor prognosis, with survival rates of 10–30% [3]. In adults, T-LBL accounts for 90% of all LBL cases, with survival of 50–60% and dismal outcomes after relapse [4]. The World Health Organization classifies T-LBL and T-ALL as a single entity due to overlapping clinical and biological features, yet recent studies have revealed molecular differences [5], including *TRB::NOTCH1* fusions that are restricted to pediatric T-LBL and associated with high relapse risk [6].

Clonal evolution analyses allow assessment of tumor progression over time. However, these approaches are not widely used clinically, as automated reconstruction tools often generate unreliable results [7] and single-cell DNA sequencing still faces technical and analytical barriers. Existing studies primarily examine single-nucleotide variants (SNVs) and indels while modeling their underlying copy number. Integrated analyses combining SNVs, indels, and copy number variants (CNVs) remain limited. Here, we use next-generation sequencing (NGS) and SNP-array data to characterize molecular genetic differences between pediatric and adult T-LBL and T-ALL and evaluate their clonal evolution.

Clinical characteristics of the four subgroups, pediatric T-LBL, pediatric T-ALL, adult T-LBL and adult T-ALL, are summarized in Supplementary Table 1. Pediatric T-LBL samples were obtained from reference-reviewed cases from the NHL-BFM Registry 2012 and the LBL-2018 clinical trial, depending on material availability. Pediatric T-ALL cases represent relapses with 1:2 matched controls from the AIEOP-BFM ALL-2009 study [8].

We analyzed SNVs and indels in 211 age-stratified T-ALL and T-LBL cases using a 52-gene NGS panel and appreci8 for variant calling (Supplementary Table 2, Supplementary Methods). The total mutation burden did not differ significantly between subgroups (T-ALL: pediatric x̅ = 3.08 adult x̅ = 3.32; T-LBL pediatric x̅ = 3.13 adult x̅ = 3.28). Pathway-based grouping revealed distinct age- and entity-specific patterns, although *NOTCH1* remained the most frequently mutated gene in all groups (Fig. 1A, Supplementary Data 1; detection threshold 1% in Supplementary Fig. 1).

**Fig. 1:**
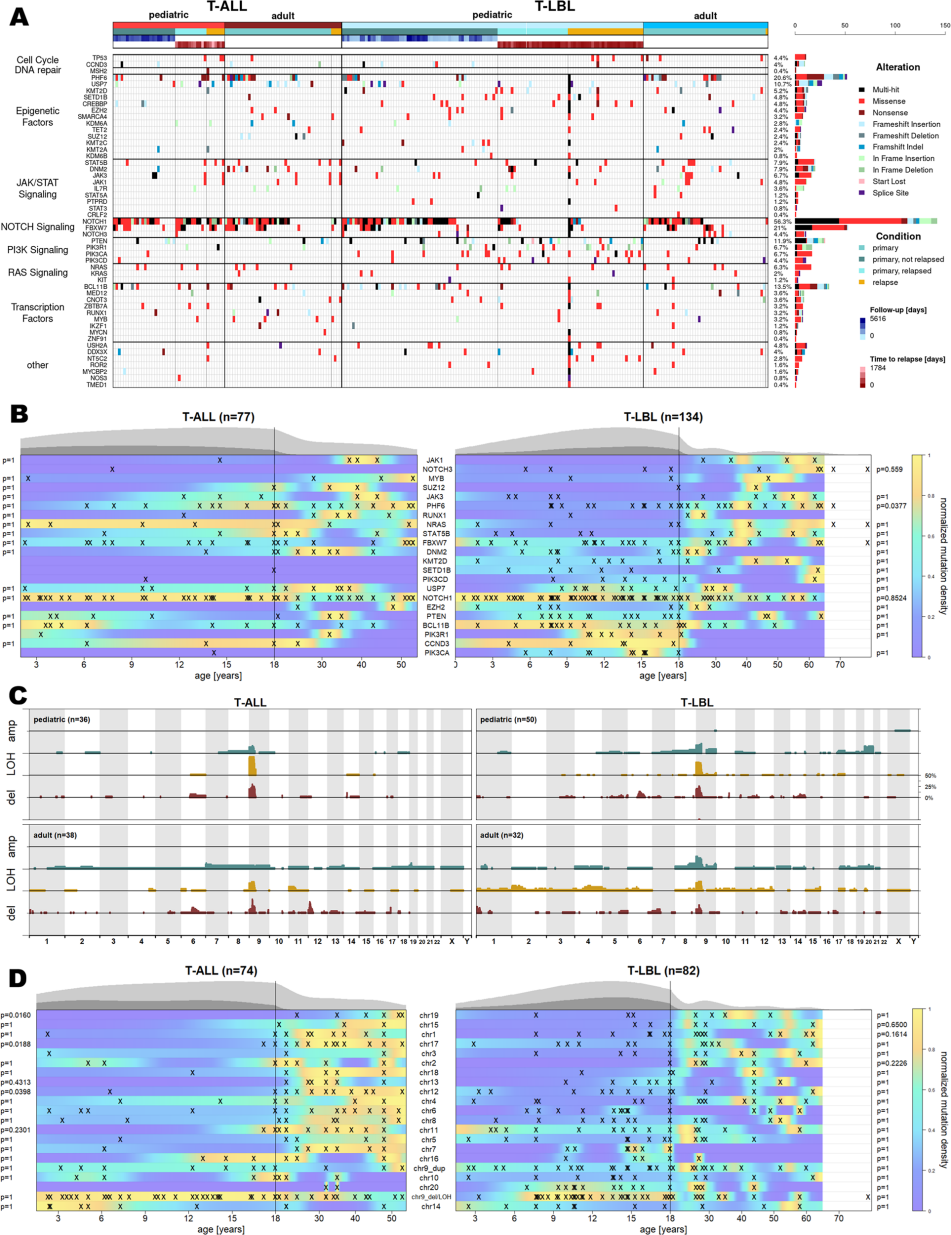
Mutational landscape in T-ALL and T-LBL considering SNVs, small indels and CNVs. **A** Variants detected by NGS (detection threshold 10%). Mutated genes are categorized by affected pathway. Pediatric and adult cases are distinguished. For pediatric cases, information on follow-up and time to relapse is provided, respectively. **B** Age-dependent distribution of the 22 genes with highest mutation frequencies in T-ALL and T-LBL. An ‘x’ marks a gene being mutated in one patient. Colors define the mutation density normalized for baseline age distribution, additionally depicted as grey curve on top of the plot (light grey: all patients, dark grey: relapsed patients). All genes mutated in at least 5% of T-ALL or T-LBL patients are included. Adjusted p-values (Wilcoxon Rank-Sum test) are reported if a gene is mutated in ≥5% of the patients per subgroup. **C** Cumulative frequencies of CNVs detected by SNP arrays. CNVs are distinguished by their precise copy number: CN = 0 and CN = 1 (red: deletion; del), CN = 2 (gold: loss of heterozygosity; LOH), CN = 3 and CN = 4 (blue: amplification; amp). Results for the subgroups T-ALL pediatric, T-ALL adult, T-LBL pediatric and T-LBL adult are displayed. Only primary tumor samples are included. Data are scaled for a maximum frequency of 50%. **D** Age-dependent distribution of the top-20 mutated chromosomes in T-ALL and T-LBL. An ‘x’ marks a chromosome being mutated in one patient. Colors define the mutation density normalized for baseline age distribution, additionally depicted as gray curve on top of the plot (light grey: all patients, dark grey: relapsed patients). All chromosomes mutated in at least 5% of T-ALL or T-LBL patients are included. Adjusted *p* values (Wilcoxon Rank-Sum test) are reported if a gene is mutated in ≥5% of the patients per subgroup.

Alterations in *RAS*, *JAK-STAT*, and *PI3K* signaling varied between cohorts. *PI3K*-pathway mutations (*PIK3CA, PTEN, FBXW7*) were enriched in pediatric cases, particularly in T-LBL. *JAK1*^*MT*^, *SUZ12*^*MT*^ and *DNM2*^*MT*^ showed no significant age associations, but *JAK1*^*MT*^ occurred only in adult T-LBL (9%), *SUZ12*^*MT*^ only in adult T-ALL (10%), and *DNM2*^*MT*^ was more common in adult T-LBL (17% vs. 3%). *PHF6*^*MT*^ were significantly more frequent in adult compared to pediatric T-LBL (34% vs. 13%; *p*_adj_ = 0.0410). In pediatric T-LBL, *PHF6*^*MT*^ occurred exclusively in non-relapsed cases. In T-ALL, *PHF6*^*MT*^ were more common in adults, but age-adjusted analyses showed no significant differences (adult 37% vs pediatric 19%; Fig. 1A, B; Supplementary Figs. 2, 3).

*NOTCH1*^*MT*^ were more prevalent in pediatric than adult patients (T-ALL: 78% vs. 61%; T-LBL: 60% vs. 49%). In pediatric T-LBL, *NOTCH1*^*MT*^ were enriched in non-relapsed cases (73% vs. 30%). Furthermore, *NOTCH1* status was associated with time to relapse (*p* = 0.0256; Supplementary Fig. 4). Most *NOTCH1* hotspot mutations affected the intracellular and HD domains, whereas truncating variants were confined to the PEST domain, consistent with constitutive activation [9] (Supplementary Fig. 5).

Exploratory analyses of unadjusted p-values revealed mutual exclusivity of *NOTCH1* and *PTEN* in pediatric T-ALL (odds ratio OR = 0.06; *p* = 0.0194) and pediatric T-LBL (OR = 0.2; *p* = 0.0193). This pattern persisted in non-relapsed pediatric T-ALL (OR = 0.05; *p* = 0.0209) but not in relapsed cases. Pediatric T-LBL additionally showed exclusivity of *NOTCH1* with *PIK3R1*, *PIK3CA*, and *SETD1B*. In adult T-LBL, *NOTCH1* and *NOTCH3* were mutually exclusive (OR = 0; *p* = 0.0046) (Supplementary Figs. 6, 7). Because pediatric samples were enriched for relapse events due to case–control design, relapse proportions do not reflect population incidence.

Overall, the mutational landscape of T-LBL and T-ALL was highly similar, consistent with prior studies [5]. Both share common driver pathways, including *NOTCH1*^*MT*^, *FBXW7*^*MT*^ and *PTEN*^*MT*^. *PHF6* was the second most frequently mutated gene in our cohort (T-LBL 20%, T-ALL 29%), comparable to reported frequencies [5, 10]. In pediatric T-LBL, *PHF6*^*MT*^ were confined to non-relapsed patients, aligning with favorable associations in adult T-LBL [11], whereas in pediatric T-ALL *PHF6*^*MT*^ have been linked to inferior outcome [12], suggesting disease- and age-specific functional contexts.

Considering CNVs, mutational burden was comparable across the four subgroups, with mean counts of x̅ = 2.25 (sd = 2.30) in pediatric and x̅ = 4.34 (sd = 4.04) in adult T-ALL, and x̅ = 4.12 (sd = 3.68) in pediatric and x̅ = 5.66 (sd = 7.46) in adult T-LBL. Outcome-based comparisons in pediatric cases revealed no clear differences (pediatric T-ALL: non-relapsed x̅ = 2.71 (sd = 2.65) vs. relapsed x̅ = 1.33 (sd = 0.89); pediatric T-LBL: x̅ = 3.68 (sd = 3.83) vs. x̅ = 4.68 (sd = 3.50); Supplementary Data 2, Supplementary Methods).

CNVs affecting chr9p were the most abundant alterations across all subgroups. Deletions were more frequent in pediatric than adult patients (weighted frequencies: pediatric T-ALL 35% vs. adult 20%; pediatric T-LBL 32% vs. adult 21%). A distinguishing feature between pediatric T-LBL and T-ALL was the enrichment of dup20 in pediatric T-LBL, particularly in relapsed samples: chr20 duplications occurred in 7% of primary non-relapsed, but in 25% of primary relapsed, 27% of first-relapse, and 36% of second/third-relapse pediatric T-LBL cases. Conversely, chr9p duplications were more common in non-relapsed pediatric T-LBL (24% vs 5%). A slight enrichment of chr6q deletions was also observed in relapsed pediatric T-LBL (6% vs. 3%; Fig. 1C; Supplementary Fig. 8).

In T-ALL, age-dependent, cut-off–free analysis demonstrated significant associations between increasing age and CNVs in multiple chromosomes, including chr19 (*p*_adj_ = 0.0166), chr17 (*p*_adj_ = 0.0454), and chr12 (*p*_adj_ = 0.0398), whereas chr14 showed an inverse pattern (Fig. 1D). No comparable age-associated accumulation was observed in T-LBL. Deletions or LOH involving chr9 showed the highest density between 7 and 20 years, although this trend was not statistically significant.

Our findings indicate a clear age-related increase in CNV burden in T-ALL but not in T-LBL. CNVs involving chromosome 9, particularly deletions of chr9p, are well-characterized drivers in both entities. The *CDKN2A/2B* locus on 9p is frequently lost [5, 10].

For 149 patients, we integrated SNVs, indels and CNVs by cancer cell fraction and reconstructed clonal evolution [13] (details in Supplementary Methods). In pediatric T-ALL, *NOTCH1*^*MT*^ frequently co-occurred with chr9p deletions (38.7%) and LOH (35.5%), whereas these combinations were less common in pediatric T-LBL (deletions 15%, LOH 19%). In pediatric T-LBL, *NOTCH1*^*MT*^ strongly associated with chr9p CNVs, particularly in non-relapsed patients (Supplementary Figs. 9A, B, 10A, B). Variant-ordering analyses showed that clones harboring *NOTCH1*^*MT*^, and less frequently *FBXW7*^*MT*^, typically arose as descendants of chr9p deletion/LOH events. CNVs preceded small variants in both entities, except for chr9p duplications, which occasionally occurred as descendants of *NOTCH1*^*MT*^ or *FBXW7*^*MT*^ in non-relapsed pediatric T-LBL (Supplementary Figs. 9C, D, 10C, D).

Analysis of conserved evolutionary trajectories confirmed that CNVs in chr9p represent early founder events. In pediatric T-ALL, LOH_in_9p→NOTCH1 (*p*_adj_ = 0.0152) and LOH_in_9p→BCL11B→NOTCH1 (*p*_adj_ = 0.0046) were significantly enriched patterns. In pediatric T-LBL, highly significant trajectories included LOH_in_9p→NOTCH1 (*p*_adj_ = 0.0016), LOH_in_9p→NOTCH1→FBXW7 (*p*_adj_ = 0.0006) and LOH_in_9p→USP7→FBXW7 (*p*_adj_ = 0.0006). Only non-relapsed pediatric T-LBL showed successive CNVs (dup_in_9p), although not significantly (Fig. 2A, B; Supplementary Fig. 10E, F). Weighted nested-level analysis substantiated early occurrence of chr9 deletions/LOH in both diseases, preceding the accumulation of additional genomic alterations (pediatric T-ALL: nested-level_tree_ = 3.35, nested-level_chr9del/LOH_ = 1.04; pediatric T-LBL: nested-level_tree_ = 4.31, nested-level_chr9del/LOH_ = 1.13).

**Fig. 2:**
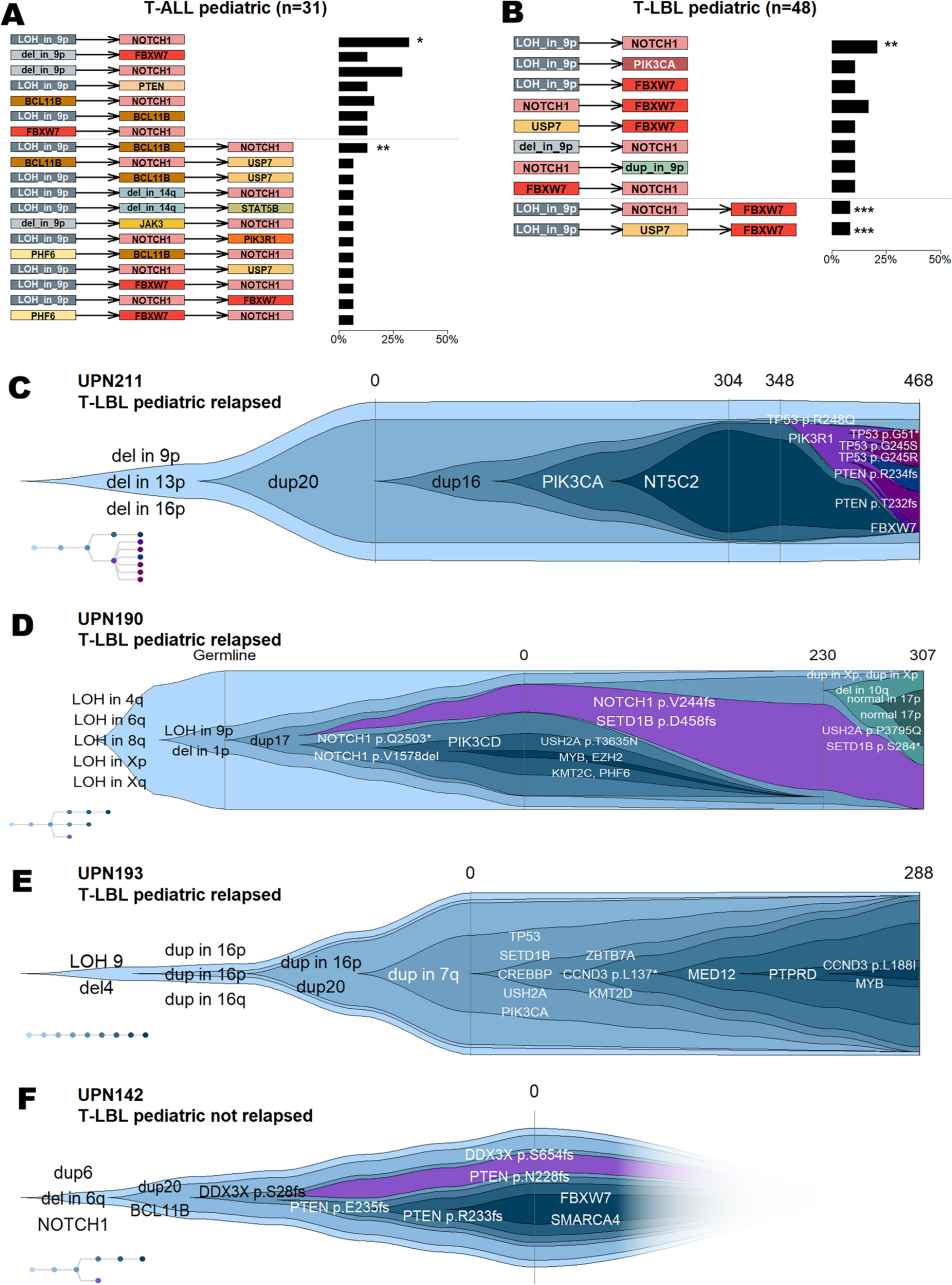
Clonal order and evolution of variants in pediatric T-ALL and T-LBL patients. **A**, **B** To determine significant associations in the order of variants, conserved evolutionary trajectories are analyzed. Patterns of length 2 and 3 are considered. Trajectories observed in 10% (2 levels) and 5% (3 levels) of samples in the corresponding subgroups are included. Significant adjusted *p*-values are marked (*<0.05, **<0.01, ***<0.001). Data shows an increased pattern of CNVs – especially deletions and LOH affecting chr9p – preceding small variants. **A** T-ALL pediatric. **B** T-LBL pediatric. **C**–**F** Dolphin plots show the development of cancer cell fraction (CCF) over time. Respectively, shark plots are a simplification of the underlying phylogenetic tree, in the lower left corner. Vertical lines mark the time points at which samples were taken. Numbers specify the days passed since initial diagnosis. If >1 variant per gene is detected for a patient, information on the precise change on protein level is provided. **C** Patient with 3 relapses. **D** Patient with 2 relapses. **E** Patient with 1 relapse. **F** Patient without relapse. Despite just one time point being available, sequencing data allows to clearly identify two branches co-existing at primary time point: three frame-shift variants affecting *PTEN* are detected. Lying within a range of 20 bp on the genome, the variants can be clearly identified to affect different alleles. As no duplication of chr10 can be observed, these variants have to emerge on two or three different branches. However, considering CCFs, *PTEN* p.Q235fs and p.R233fs jointly developing on one branch, leading to bi-allelic drop-out, and *PTEN* p.N228fs developing on a parallel branch, is the only possible explanation for the observed data.

Individual clonal reconstructions by clevRvis [14] further illustrated these dynamics (Fig. 2C–F; Supplementary Data 3, Supplementary Figs. 12–24). UPN211 showed progressive branching from diagnosis to third relapse, with early CNVs followed by increasing mutational load and later emergence of highly branched TP53/*PTEN*-altered clones. UPN190 displayed LOH at high frequency already present in germline (Supplementary Fig. 11), two *NOTCH1*-marked branches that regressed, and later CNV-rich subclones at relapse 2. UPN193 exhibited a linear pattern with sequential CNVs followed by small variants, including a relapse-specific *KMT2D*^*MT*^ (p.R5086*). In contrast, relapse-free UPN142 showed two branches with diverse *PTEN* variants descending from an early *NOTCH1*^*MT*^ founder clone.

Across patients, *NOTCH1*^*MT*^ frequently appeared as early founding events associated with favorable outcome, consistent with published data [5, 6, 13]. Cox regression–evaluating the difference between the frequency of the largest clone and clones with *NOTCH1*^*MT*^–showed that increasing proportions of tumor cells lacking *NOTCH1*^*MT*^ significantly elevated relapse risk (hazard ratio HR = 1.032; 95%-CI = [1.016; 1.048]; *p* = 4.65 × 10⁻⁵). Thus, an increase of tumor cells with *NOTCH1*^*WT*^ by 1% may increase the risk of relapse by 3.2%. Re-analysis of five published relapsed pediatric T-LBL cases showed that although 3/5 harbored *NOTCH1*^*MT*^ at diagnosis, all retained subclonal *NOTCH1*-negative branches (5–25%) [15].

Together, these results suggest that the timing and clonal position of *NOTCH1*^*MT*^, rather than its mere presence, influences relapse risk. Early *NOTCH1*^*MT*^ may initiate disease but do not appear to drive relapse, whereas subclones lacking *NOTCH1*^*MT*^ may underlie treatment resistance. Integrating SNVs, CNVs, and clonal architectures highlights molecular distinctions between T-ALL and T-LBL and supports incorporating early chr9p CNV/LOH events and NOTCH1 status into future risk stratification, particularly in T-ALL where validated molecular markers remain limited.

## Supplementary information

Supplemental Information

Supplemental Data 1

Supplemental Data 2

Supplemental Data 3

## Data Availability

NGS data are available at SRA under accession number PRJNA1216741. SNP-array data are available at GEO under accession number GSE288263. Information on all detected small variants and CNVs and reconstructed clonal evolution can be found in Supplementary Data 1–3. Code is available at https://github.com/sandmanns/ce_tlbl_tall.
