# Supplementary material for: Molecular variants, clonal evolution and clinical relevance in pediatric and adult T-cell lymphoblastic neoplasia
Source: Blood Cancer J. 2026 Apr 2;16(1):57. doi: 10.1038/s41408-026-01488-w (PMC13066408; doi:10.1038/s41408-026-01488-w)
Supplement: Supplementary file 1 — Supplemental Information [file 41408_2026_1488_MOESM1_ESM.pdf]

Supplementary Information

1. Materials and Methods

Cohort Characteristics

Patients were treated uniformly according to recent national treatment protocols for LBL and ALL. Clinical data for patient characteristics, diagnostics, treatment, and outcome were obtained from the clinical trial / registry databases. DNA samples include material from initial diagnosis, corresponding germline, and, if applicable, relapse. Subgroups include 87 pediatric T-LBL cases (27 relapsed, 60 non-relapsed), 36 pediatric T-ALL (12 relapsed, 24 non-relapsed), 47 adult T-LBL (1 corresponding relapse sample available), and 41 adult T-ALL, (4 corresponding relapse samples available) – in total, 211 patients. Part of the data (n = 21) evaluated in this study was produced and kindly provided by the INFORM program (Heipertz, et al. 2023; Peterziel, et al. 2022; van Tilburg, et al. 2021; Worst, et al. 2016).

Targeted sequencing

The targeted sequencing of 211 primary, 177 corresponding germline and 37 relapse samples was performed with the targeted gene panel, which was used in the trial LBL 2018 NCT (NCT04043494)/EU CT-No.(2023-508101-24-00) as part of the molecular genetic analyzes, covering exons of 52 genes. The relevance of this selection and its relation to T-LBL/T-ALL oncogenesis was described prior (Ruether, et al. 2022). Library preparation was performed with the Illumina DNA Prep protocol according to the manufacturer's instructions.

SNP arrays

Detection of CNVs in 157 primary, 160 corresponding germline and 32 relapse samples was performed using the Illumina Infinium global screening array v3.0.

Whole exome sequencing

For 10 cases (10 germline, 1 primary, 10 relapse samples), whole exome sequencing (WES) data, which had already been generated by the DKFZ (Deutsches Krebsforschungszentrum Heidelberg) in the context of the INFORM registry, could be used for detection and validation of variants.

For 9/10 germline samples, overlapping targeted sequencing and SNP array data are available. Thus, WES could be used for validation of small variants detected in targeted sequencing and CNVs detected in SNP array data. Furthermore, 1/1 primary sample has overlapping next-generation sequencing (NGS) and SNP array data as well as 7/10 (NGS) and 5/10 (SNP array) relapse samples.

Detection of small variants

Alignment against the human reference genome hg19 was performed using BWA mem (Li 2013). Variant calling was performed using the established pipeline appreci8 (Sandmann, et al. 2018) to allow for valid detection of low-frequency variants (thresholds: minimum coverage 50, minimum number of reads with the alternative allele 5, minimum variant allele frequency VAF 1%).

Pooling over all germline samples, germline variants were automatically filtered. Assuming a binomial distribution, 99% confidence intervals for the estimated VAFs were calculated for all putative variants. A variant detected in a primary or relapse sample was filtered as germline if the confidence interval overlapped with the estimated VAF of the corresponding variant call in at least one germline sample. Unclear calls were inspected manually.

To investigate early, potentially aggressive clones below detection thresholds, all samples of a patient were screened for variants called in only one of the samples. Additional expert-based, manual review of all variant calls was performed evaluating extended annotation (e.g. presence and frequency in databases and *in silico* prediction) as well as inspecting variants in the Integrative Genomics Viewer (Robinson, et al. 2017). Further details are provided in the Supplemental Methods, the final list of single-nucleotide variant (SNV) and indel calls is provided in Supplemental Data 1.

Detection of CNVs: Primary filtration of low-quality samples and CNV calls

Initial Copy Number Variant (CNV) calling was performed using GenomeStudio Software version 2.0 (Illumina, San Diego, CA) with default configuration. To identify and filter low-quality samples, standard deviation of the log2Ratio was determined. All samples with sd(log2Ratio)≥0.8 were excluded from further analysis (n=8) as they do not warrant for valid CNV calling. Samples with 0.4≤sd(log2Ratio)<0.8 were considered being of intermediate quality, allowing for detection of very large CNVs. Calls with a distance <10,000bp were merged if the same copy number was detected. Subsequently, filtration of the calls for length ≥1,000,000bp for deletions and ≥10,000,000bp for amplifications and loss of heterozygosity

(LOH) was performed. For the corresponding regions of CNV, the density distribution of the B allele frequency (BAF) was determined (R 4.3.2 (R Core Team 2024), base function density() with adjust=0.4). Assuming a true CNV is characterized by only 2 peaks, CNV calls with 3 peaks were excluded as false positives. For high-quality samples (sd(log2Ratio)<0.4), calls with distance <10,000bp were merged. Subsequently, calls were filtered for length ≥10,000bp for deletions and ≥1,000,000bp for amplifications and LOH.

To ensure high sensitivity and specificity of our CNV calling, B allele frequency (BAF) and log2R were additionally visualized and inspected manually for all samples. Additional putative CNVs that were discovered by this process and missed by GenomeStudio due to increased noise level of the sample were manually added to the interim list of filtered CNVs.

Detection of CNVs: Calculation of CCFs and secondary filtration

Subsequent to primary filtration of low-quality samples and CNV calls, detailed further analysis was performed. Cancer Cell Fractions (CCFs) of all filtered CNV calls were calculated. To do so, homozygous SNPs were determined in the corresponding matching germline samples (BAF≤0.1 or BAF≥0.9) and discarded from the analysis of primary and relapse samples. Subsequently, the BAFs of the remaining SNPs in the tumor samples were normalized for the major allele (e.g. changing BAF=0.3 to 1-0.3=0.7) and transformed to CCF (for deletions and LOH: (2*BAF-1)/BAF; for duplications: (2*BAF-1)/(1-BAF), SNPs with random variation in BAF leading to CCFs >150% for duplications were replaced by NA).

A 2-sided t-test was applied (H_0: CCF=0, H_1: CCF≠0). The estimate, i.e. the mean CCF, the 95% confidence intervals of the estimates as well as the number of SNPs on which the statistic is based were determined. While no cut-off for CCF was applied for additional filtration of CNV calls, all calls were manually investigated, evaluating CCF statistics, visualizing BAF and log2Ratio and comparing SNP array to targeted sequencing data in case of CNVs overlapping our target panel. Conditional on the resolution and noise level of SNP arrays, all reported CNVs are characterized by CCF>15%. The final list of CNVs is provided in Supplemental Data 2.

Variant calling in WES data

Detection of small variants in the WES samples was performed analogously to targeted sequencing data, focusing on positions covered by our target panel. Regarding CNV calling, we used the available WES data to mimic SNP arrays. For every sample, non-homozygous SNPs were identified based on germline array data (BAF≤0.1 or BAF≥0.9). For the remaining SNPs overlapping exonic positions, coverage and BAF were determined based on WES data. CCFs were estimated and detailed analysis was performed analogously to SNP arrays.

Mutational landscape analysis

All analyzes involving the evaluation, annotation and visualization of variants were performed using R 4.3.2.(R Core Team 2024) For small variants, our main results focus on the clinically relevant threshold of VAF≥10%. In the supplement, additional results are available for experimental VAF cut-off 1%.

To determine mutation frequencies for CNVs, we used R package GenVisR (Skidmore, et al. 2016), function cnFreq(). Five different types of CNVs can be observed in our cohort, based on the copy numbers reported by GenomeStudio: CN=0 and CN=1 (deletions), CN=2 (loss of heterozygosity, LOH), CN=3 and CN=4 (amplifications).

Survival analysis

Cox regression was performed using R packages 'survival' (https://CRAN.R-project.org/package=survival) and 'survminer' (https://CRAN.R-project.org/package=survminer).

Age-dependent analysis

Analysis of cut-off free, age-dependent distribution of mutated genes (in case of small variants) and chromosomes (in case of CNVs) was performed. All genes/chromosomes mutated in at least 5% of T-ALL or T-LBL patients were included, resulting in a total of 22 mutated genes and 20 mutated chromosomes considered in this analysis.

A patient's mutational status of a gene was considered a binary event – mutated or not mutated. The precise number of variants per gene per patient, was not evaluated. Baseline density over the whole cohort was determined using R function density() with n=500 and smoothing bandwith 'SJ'. Analogously, distribution of age was determined over all patients harboring at least one variant in a considered gene. Subsequently, the gene-specific distribution was normalized for the baseline age distribution. Of note, for subgroup analysis of T-LBL, due to data sparseness the maximum age was set to 64. Data on only two patients at higher age (68 and 79) are available. As this would lead to an over-estimation of age-dependent mutation density for genes mutated in these two patients, they were excluded from density calculations. Instead, their mutational status is just reported as 'x'.

If a gene was mutated in min. 5% of the patients per subgroup, Wilcoxon Rank-Sum test was applied to identify genes/chromosomes with a significant age-dependent mutation pattern. Reported p values were adjusted for multiple testing using Bonferroni correction.

Analysis of co-occurrence and mutual exclusivity

Considering the top-22 mutated genes in our target panel, we analyzed the co-occurrence and mutual exclusivity of variants. The Odds Ratio (OR) provides an indicator of which variants are likely to co-occur (OR>1) and which variants are mutually exclusive (OR<1). For genes mutated in ≥5% per subgroup, we tested for statistical significance using Fisher's exact test and adjusted for multiple testing (Bonferroni). For additional exploratory analysis, significant un-adjusted p values were marked.

Clonal evolution: Filtration of incomplete patient data sets

We reconstructed clonal evolution by integrating results on small variants detected by NGS, CNVs detected by SNP arrays, and information on their increase and decrease in frequency over time. Data on 211 patients are available. However, incomplete data hamper valid estimation of the tumor development. Therefore, patients were excluded if 1) no SNP array data were available (n=45), 2) SNP array data were only available for germline samples (n=6), 3) no data were available for a final time point, that is time point `primary' for non-relapse samples and time point `relapse' for relapse samples (n=8), 4) no germline data were available (n=3). Altogether, n=48 pediatric T-LBL (n=16 with 1 relapse, n=5 with 2 relapses, n=1 with 3 relapses), n=31 pediatric T-ALL (n=7 with relapse), n=32 adult T-LBL (1 with relapse) and n=38 T-ALL adult (n=3 with relapse) cases remained.

Of note, we also analyzed the clonal evolution in patients without relapse, that is evaluating one time point compared to germline. This limited data may potentially complicate the reconstruction of clonal evolution, bearing the risk of underestimating the correct number of branches dependent on the precise constellation of variants. Nonetheless, it is possible to validly determine the order by which variants developed.

Clonal evolution: Reconstruction of tumor development

Clonal evolution was reconstructed manually, following the example of da Silva et al and Reutter et al (da Silva-Coelho, et al. 2017; Reutter, et al. 2021). For CNVs, CCFs were calculated as previously described. For small variants, the CCF can be calculated as 2*VAF. In case of overlapping CNVs, CCFs were adjusted according to the formula provided by Sandmann et al (Sandmann, et al. 2022). Inaccuracies in the estimated CCFs were considered

by evaluating 95% confidence intervals (for CNVs: caused by random variation in the observed BAFs and a low number of SNPs; for SNVs/indels: caused by low coverage of the region).

Ultimately, each variant was assigned to a clone. The CCFs of the clones are provided for all time points including germline in Supplementary Data 3. Of note, we only consider linear and branching evolution, providing additional information on the number of branches. While neutral evolution is a common concept, the definition of a precise threshold for the number of branches that differentiates branching from neutral evolution is unclear.

For every clone, the nested level (= depth of the clonal evolution tree), the number of siblings (= clones on the same nested level, existing in parallel) and the ID of the parent clone are provided. This information allows to distinguish parallel, dependent branches from independent branches. Additionally, we provide information on the number of alleles vs the number of non-mutated alleles, to identify bi-allelic events. However, a clear indication is not always possible. If, for example, two distant variants on NOTCH1 are detected, it is unclear whether they affect the same or different alleles. The chance of observing a bi-allelic event is 50%. Therefore, we denote the number of healthy alleles as 0.5 out of 2.

Visualization of clonal evolution was realized using clevRvis.(Sandmann, et al. 2022a), functions sharkPlot() and dolphinPlot() (with enabled time point interpolation).

Clonal co-occurrence and order of variants: Harmonization of CNVs

For samples with reconstructed clonal evolution, we analyzed the clonal co-occurrence and order of variants present at primary time point. This approach has been previously described by Schwede et al (Schwede, et al. 2024). However, as our data set contains both information on small variants as well as CNVs, we extended the approach to perform a combined analysis of mutated genes and mutated chromosomes.

The great diversity of CNVs may hamper integration across different samples. Therefore, we apply the following approach for harmonization of CNVs: an exemplary deletion, located on the p arm of chromosome 9, is re-labeled to “del_in_9p”; a deletion on the q arm analogously to “del_in_9q”. The precise length of the variant on the corresponding arm is not considered. A deletion affecting the whole p arm of chromosome 9 is equally labeled “del_in_9p”. A deletion affecting the whole chromosome 9 is split to two CNVs, namely “del_in_9p” and “del_in_9q”. While this approach bears a simplification of previously determined CNVs, it allows for integration of data to identify common patterns across different samples.

Clonal co-occurrence and order of variants: Count and visualization

For every pair of two variants – genes and chromosomes mutated in at least 10% of pediatric T-ALL or pediatric T-LBL patients – we determined the number of patient samples that feature both variants. No statistical test was applied. By color-coding, we added information on the frequency of two variants appearing in the same clone, that is whether they developed on the same evolutionary branch and thus, cells harboring both variants exist.

For pairs of variants appearing in the same clone, we performed additional analysis of order. We applied a cut-off of ≥10% for samples featuring two variants in the same clone. By color-coding, we added information on the frequency of variant 1 being ancestor to variant 2. If a gene was affected by more than one variant, e.g. in a linear evolutionary pattern, one variant in *FBXW7* in clone 1, another in clone 3 and one in clone 4 and, in addition, one variant in *KRAS* in clone 2, then *FBXW7* (in clone 1) was observed being ancestor to *KRAS*, but *KRAS* was also observed being ancestor to *FBXW7* (in clones 2 and 3). Both relations were counted. Yet, *KRAS*➔*FBXW7* was only counted once – and not thrice – as it was observed in one patient. As our study is based on the analysis of bulk DNA-seq data, some variants showing only minor differences in CCF were assumed to have developed simultaneously. In this case, for two variants A and B developing at the same time, we neither counted A as possible ancestor of B, nor B as ancestor of A.

For the subgroup of T-LBL pediatric, we performed an additional analysis of clonal co-occurrence and order of variants, comparing relapsed vs not relapsed patients at their primary time point. All genes and chromosomes mutated in at least 10% of pediatric T-LBL patients were included.

Conserved evolutionary trajectories

To extend the prior analysis on the clonal order of variants, we determined conserved evolutionary trajectories in primary samples. Mutated genes in combination with mutated chromosomes were evaluated, excluding simultaneously developing variants. Harmonization of CNVs was realized as it already was for the analysis of clonal co-occurrence and order of variants. We followed our previously outlined method (Sandmann, et al. 2024), taking patterns of 2 and 3 levels into account. We calculated the probability p_{ij} for observing a pattern *i* in patient *j* using Laplace-distribution.

$$p_{ij}=\frac{\#observed\ patterns}{\#possible\ patterns}$$

The number of observed patterns was counted directly from the given data. The number of possible patterns could be calculated as

$$\#possible\ patterns = \frac{m!}{(m-l)!}$$

with m being the number of unique mutations and l being the number of levels, that is 2 or 3. Patterns were filtered for frequency. Cut-offs of ≥10% for levels of 2, and ≥5% for levels of 3, which are more complex and can only be observed in evolutionary trees of nested level ≥3, were applied. For each filtered pattern i observed in a group of n patients, the probability vector $P_{i^*} = [p_{i1}, p_{i2}, ..., p_{in}]$ was defined. The vector was assumed to follow a Poisson binomial distribution, being the sum of n independent not identically distributed Binomial variables. Statistical significance was assessed by calculating the probability for observing pattern i in $\geq c_i$ out of n patients. P values were adjusted for multiple testing using Bonferroni correction. Of note, an observed pattern does not imply that two variants were acquired in directly consecutive clones.

Data availability

NGS data are available at the SRA under accession number PRJNA1216741. SNP-array data are available at GEO under accession number GSE288263. Information on all detected small variants and CNVs and reconstructed clonal evolution can be found in a data supplement available with the online version of this article.

Code availability

Code is available at https://github.com/sandmanns/ce_tlbl_tall.

2. Tables

Supplementary Table 1: Clinical characteristics of patients from the studied cohort. The table summarizes the baseline characterization of the four subgroups T-LBL pediatric, T-ALL pediatric, T-LBL adult and T-ALL adult.

**Patient Characteristics**	**T-LBL pediatric**	**T-ALL pediatric**	**T-LBL adult**	**T-ALL adult**
**total number**	87	36	47	41
**sex**				
male	61 (70 %)	27 (75%)	31 (66 %)	28 (68 %)
female	26 (30 %)	9 (25%)	16 (34 %)	13 (32 %)
**age**				
<10	36 (41 %)	20 (56 %)	-	-
Okt 14	33 (38 %)	12 (33 %)	-	-
≥15-18	18 (21 %)	4 (11 %)	-	-
18-35	-	-	28 (60%)	26 (63%)
36-65	-	-	17 (36%)	15 (37%)
>65	-	-	2 (4%)	-
**stage**				
II	1 (1 %)			
III	62 (71 %)			
IV	24 (28 %)			
**bone marrow involvement**				
no	67 (77 %)	-	1 (4 %)	-
yes	20 (23 %)	36	22 (96 %)	41
**relapse**				
not relapsed	60 (69 %)	24 (67 %)		
follow-up [years] median (inter quartile range)	9.58 (3.13)	10.06 (3.61)	4.86 (1.80	4.24 (1.19)
relapsed	27 (31%)	12 (33%)	1*	4*
time to first relapse [years] median (inter quartile range)	0.83 (0.49)	1.32 (1.67)	1.28 (-)	1.42 (0.33)

*Information on relapse status in adult T-LBL and adult T-ALL is not available for all samples. The given numbers indicate the cases with material available in this study.

Supplementary Table 2: OMICs data of patients from the studied cohort. The table summarizes the NGS and SNP-array data of the four subgroups T-LBL pediatric, T-ALL pediatric, T-LBL adult and T-ALL adult.

**Patient Characteristics**	**T-LBL pediatric**	**T-ALL pediatric**	**T-LBL adult**	**T-ALL adult**
**total number**	87	36	47	41
**collected NGS data**				
germline	56 (64%)	36 (100%)	45 (96%)	41 (100%)
primary	87 (100%)	36 (100%)	47 (100%)	41 (100%)
relapse	23 (85%)*	7 (58%)	1 (100%)	4 (100%)
**collected SNP array data**				
germline	55 (63%)	36 (100%)	32 (68%)	38 (93%)
primary	50 (57%)	36 (100%)	33 (70%)	38 (93%)
relapse	21 (78%)**	6 (50%)	1 (100%)	3 (75%)

* NGS data are available for 23 out of 27 patients for at least one relapse time point; for 22/27 data on the first re-lapse are available, for 6/7 patients data on a second and for 1/1 patients data on a third relapse are available.

** SNP array data are available for 21 out of 27 patients for at least one relapse time point; for 20/27 data on the first relapse are available, for 6/7 patients data on a second and for 1/1 patient data on a third relapse are available.

3. Figures

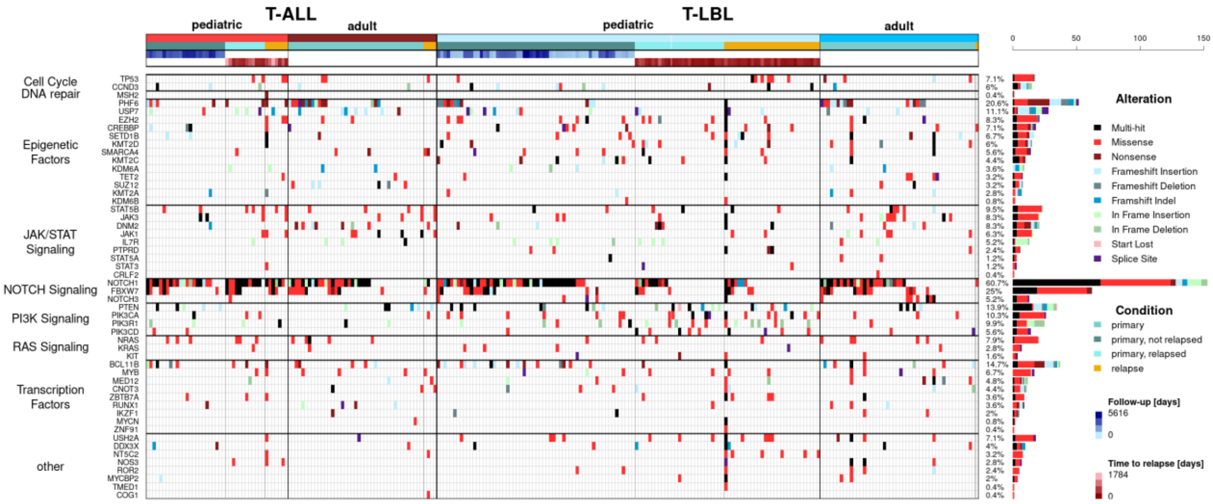

Supplementary Figure 1: Mutational landscape in T-ALL and T-LBL considering small variants. Results on SNVs and small indels are visualized, detected in the 52 genes targeted by next-generation sequencing (detection threshold 1%). Mutated genes are categorized by affected pathway. Pediatric and adult cases are distinguished. For pediatric cases, information on follow-up and time to relapse is provided respectively.

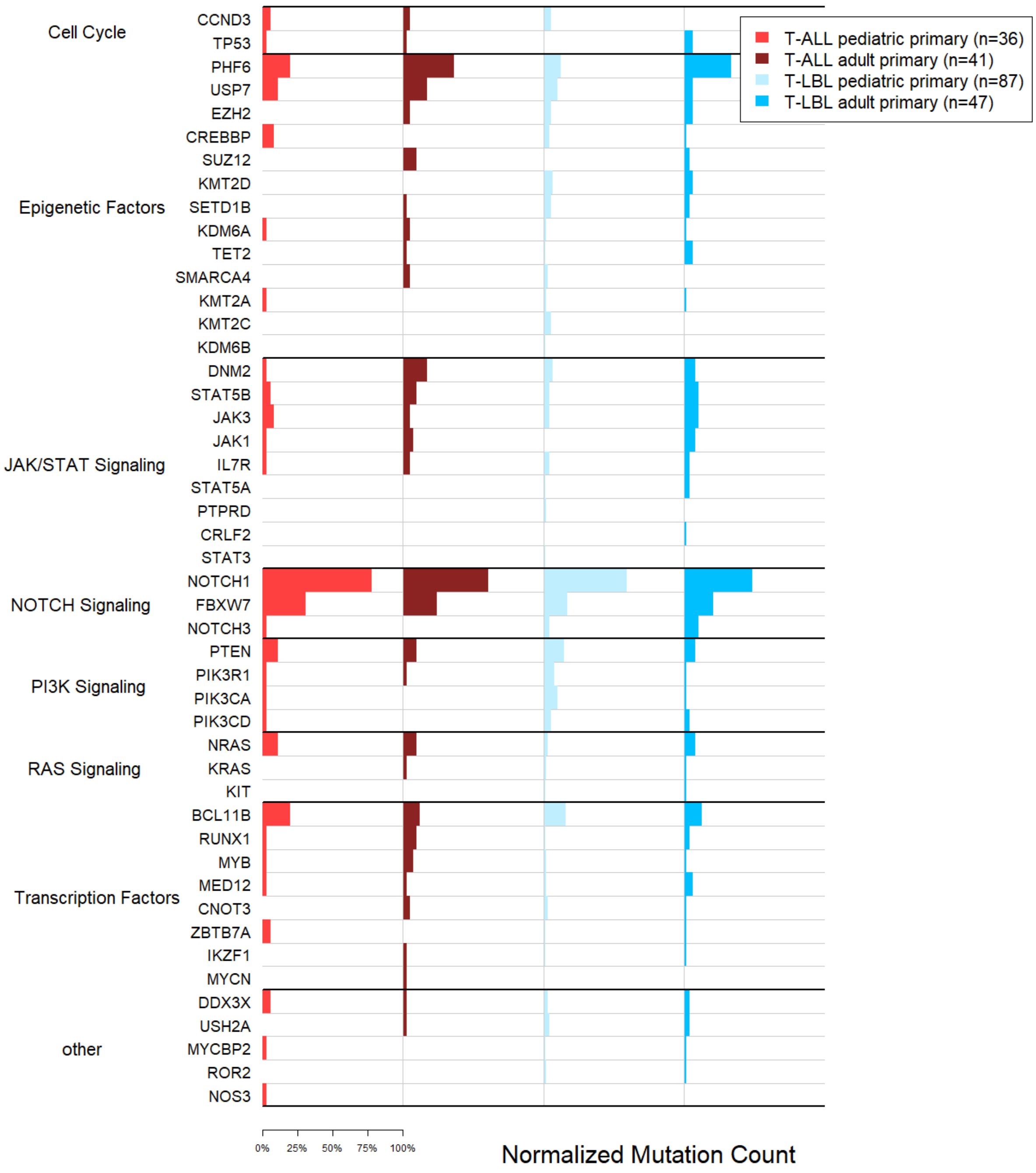

Supplementary Figure 2: Normalized mutation counts per gene for primary samples T-ALL pediatric, T-ALL adult, T-LBL pediatric and T-LBL adult. Results on SNVs and small indels are visualized, detected in 47 out of 52 genes targeted by next-generation sequencing (detection threshold 10%). Counts are normalized for the size of the corresponding subgroup. Mutated genes are categorized by affected pathway.

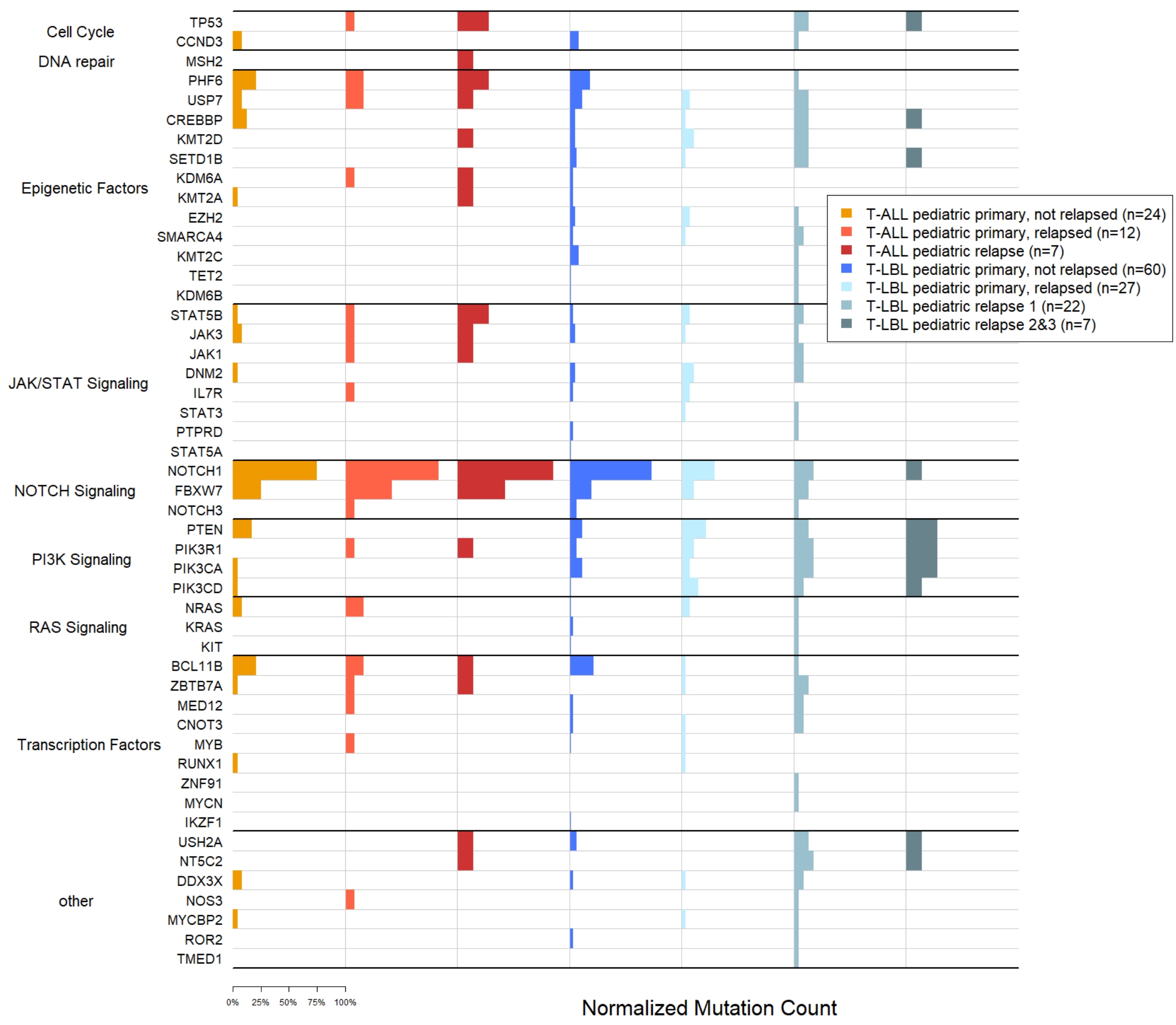

Supplementary Figure 3: Normalized mutation counts per gene for primary and relapse samples T-ALL pediatric and T-LBL pediatric. Results on SNVs and small indels are visualized, detected in 49 out of 52 genes targeted by next-generation sequencing (detection threshold 10%). Counts are normalized for the size of the corresponding subgroup. Mutated genes are categorized by affected pathway.

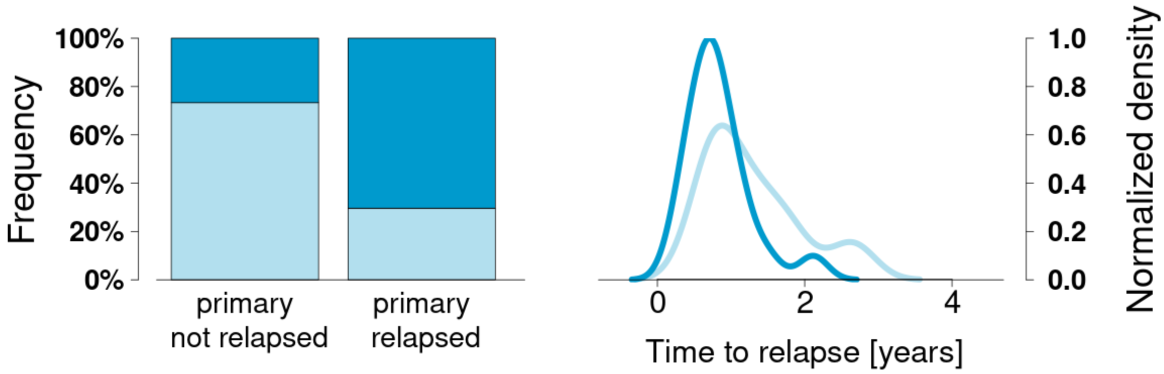

Supplementary Figure 5: Distribution of mutated *NOTCH1* in pediatric T-LBL. Relative frequencies are distinguished between non-relapsed and relapsed cases (1-sided Wilcoxon rank-sum test, *p*=0.0256).

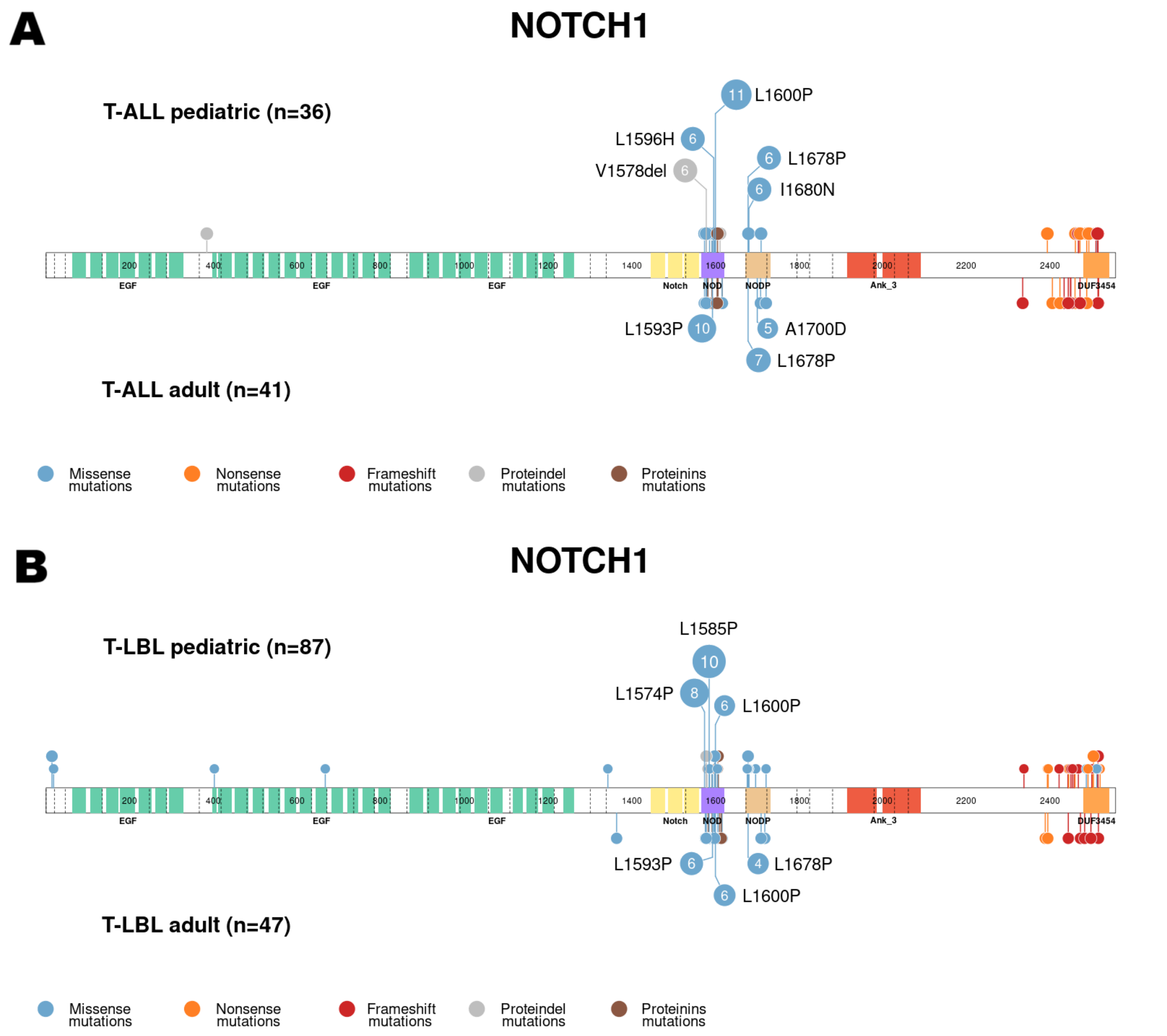

Supplementary Figure 4: Lollipop plot of *NOTCH1*. Results on SNVs and small indels are visualized (detection threshold 10%). **A)** Location and type of variants detected in T-ALL patients, comparing pediatric to adult samples. **B**) Location and type of variants detected in T-LBL patients, comparing pediatric to adult samples.

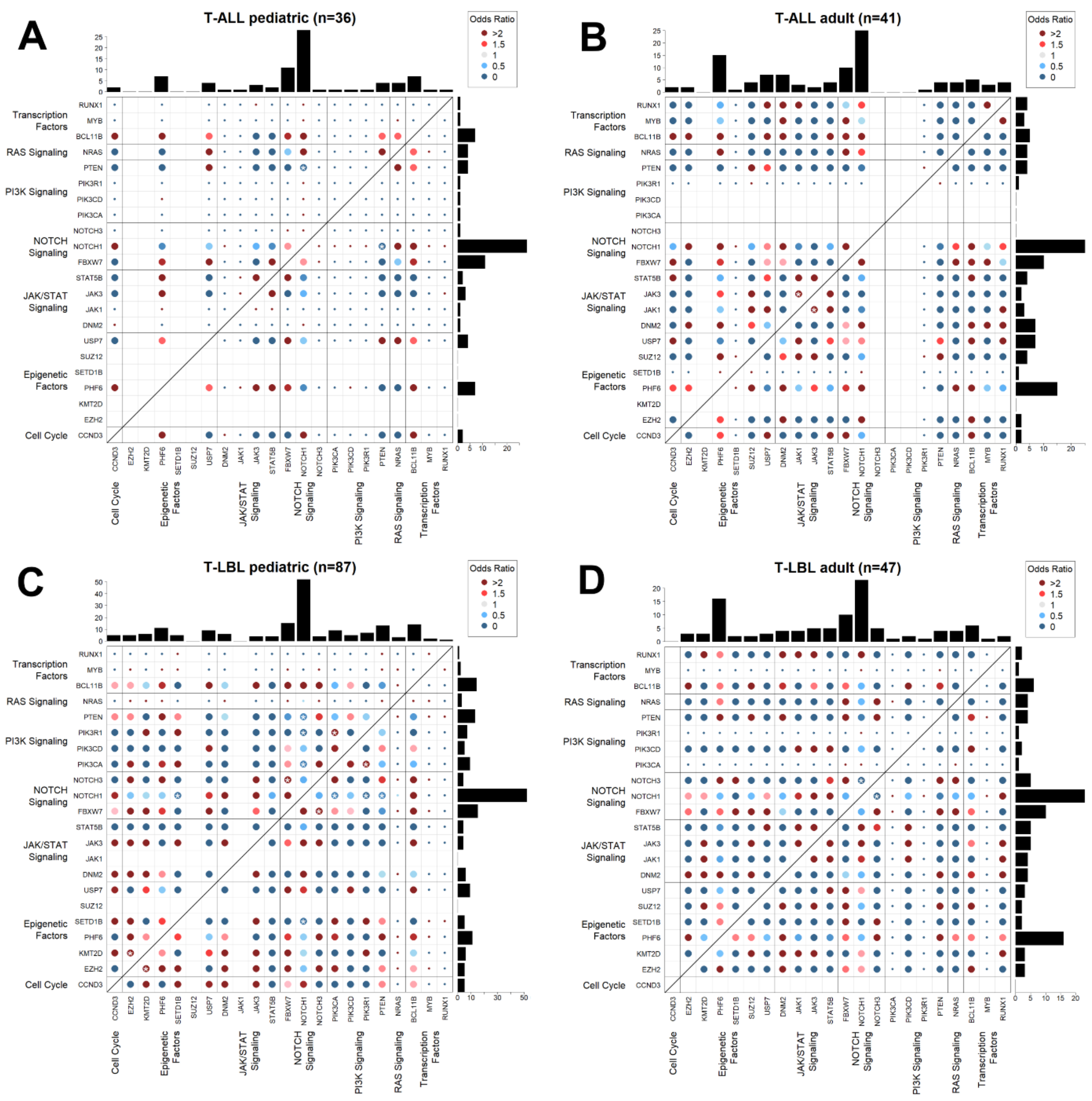

Supplementary Figure 6: Co-occurrence and mutual exclusivity of mutated genes in T-ALL and T-LBL. Top-22 targeted genes, mutated in ≥5% of T-ALL or T-LBL patients. Genes are categorized by affected pathway. Colors define the odds ratios (OR). A value >1 (red) indicates co-occurring, a value < 1(blue) mutually exclusive variants. For genes mutated in ≥5% of the considered subgroup (large dots), Fisher's exact test was applied. Adjusting p values for multiple testing, no significant results can be observed. Relations with significant un-adjusted p value are marked by white asterisks for exploratory analysis. For genes mutated in ≥5% in another but the considered subgroup, OR is just visualized (small dots) and no test is applied. Barplots on the axes show the mutation frequencies per subgroup. **A**) T-ALL pediatric. **B**) T-ALL adult. **C**) T-LBL pediatric. **D**) T-LBL adult

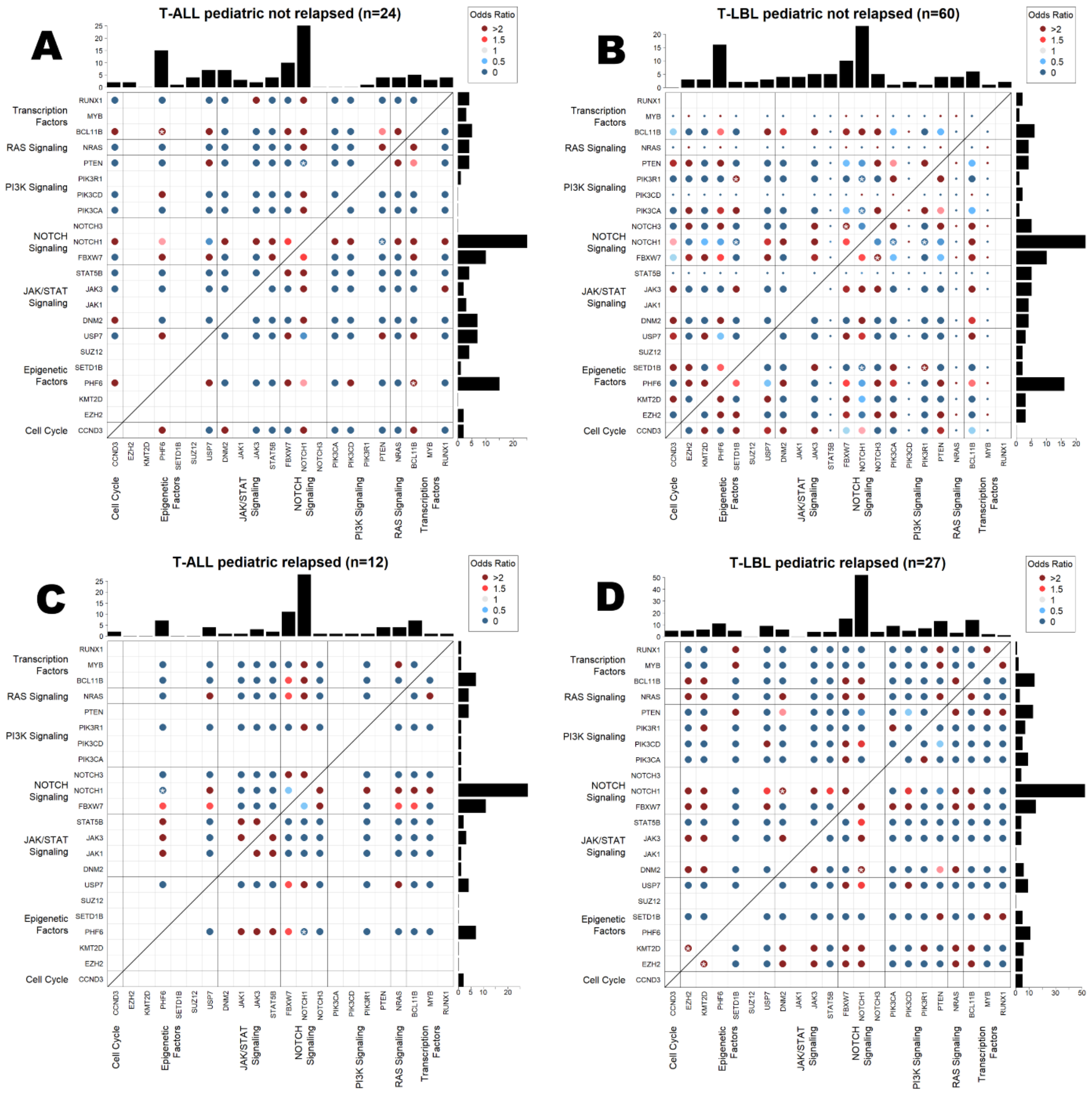

Supplementary Figure 7: Co-occurrence and mutual exclusivity of mutated genes for not relapsed and relapsed T-ALL pediatric and T-LBL pediatric. Top-22 targeted genes, mutated in ≥5% of T-ALL or T-LBL patients. Genes are categorized by affected pathway. Colors define the odds ratios (OR). A value >1 (red) indicates co-occurring, a value < 1(blue) mutually exclusive variants. For genes mutated in ≥5% of the considered subgroup (large dots), Fisher's exact test was applied. Adjusting p values for multiple testing, no significant results can be observed. Relations with significant un-adjusted p value are marked by white asterisks for exploratory analysis. For genes mutated in ≥5% in another but the considered subgroup, OR is just visualized (small dots) and no test is applied. Barplots on the axes show the mutation frequencies per subgroup. **A**) T-ALL pediatric not relapsed. **B**) T-LBL pediatric not relapsed. **C**) T-ALL pediatric relapsed. **D**) T-LBL pediatric relapsed.

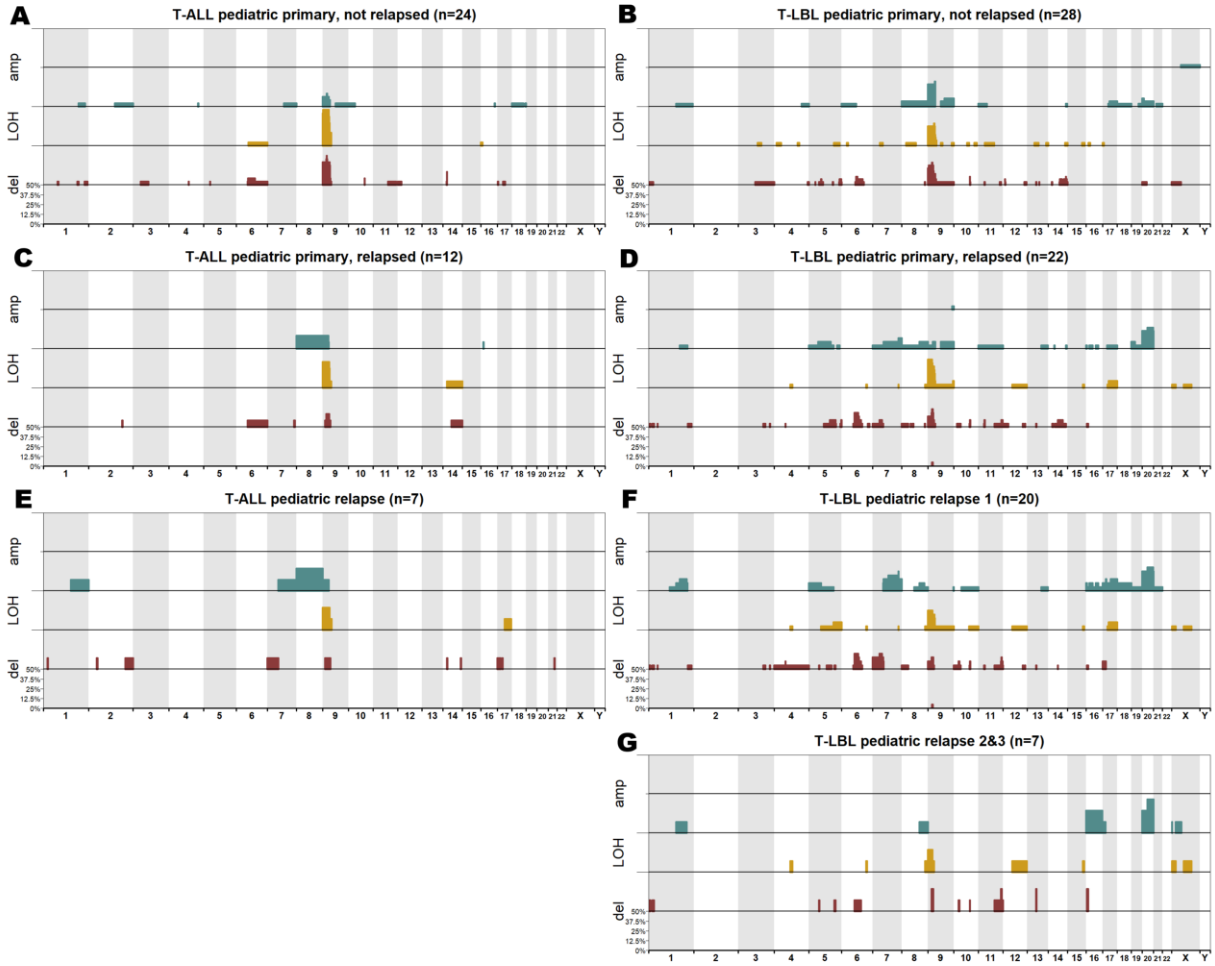

Supplementary Figure 8: Cumulative frequencies of CNVs detected by SNP arrays for primary and relapse samples T-ALL pediatric and T-LBL pediatric. CNVs are distinguished by their precise copy number: CN=0 and CN=1 (red: deletion; del), CN=2 (gold: loss of heterozygosity; LOH), CN=3 and CN=4 (blue: amplification; amp). Data are scaled for a maximum frequency of 50%. **A**) T-ALL pediatric primary, not relapsed. **B**) T-LBL pediatric primary, not relapsed. **C**) T-ALL pediatric primary, relapsed. **D**) T-LBL pediatric primary, relapsed. **E**) T-ALL pediatric relapse. **F**) T-LBL pediatric relapse 1. **G**) T-LBL pediatric relapse 2&3.

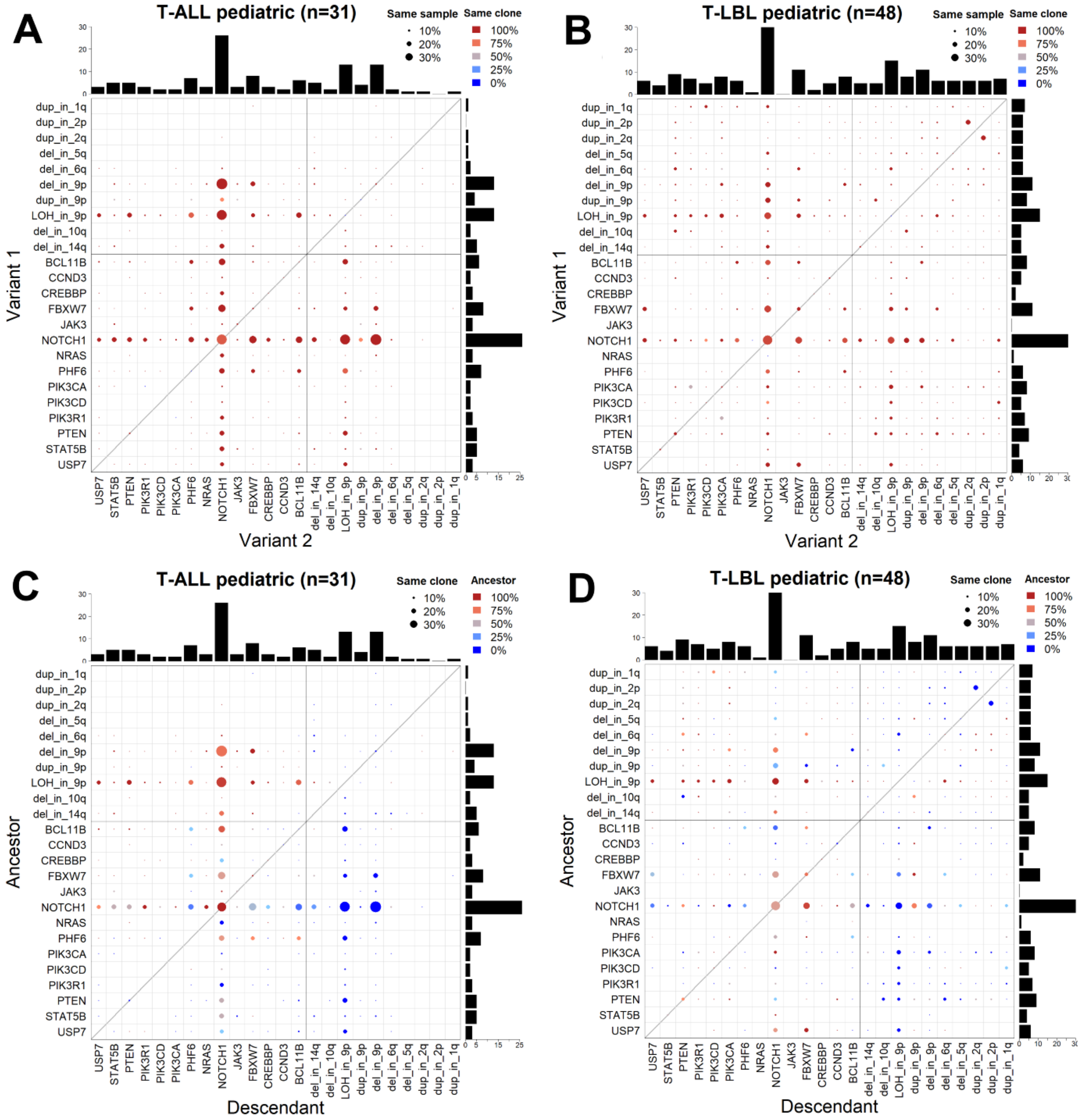

Supplementary Figure 9: Clonal co-occurrence and order of variants in pediatric T-ALL and T-LBL patients. A-B) Clonal co-occurrence of SNVs/indels and CNVs in ≥10% of the samples per subgroup are included. The dot size correlates with the percentage of patient samples for which two variants co-occur. The color codes the frequency of variants observed in the same clone, which is the same evolutionary branch. **A**) T-ALL pediatric. **B**) T-LBL pediatric. **C-D**) For pairs of variants showing clonal co-occurrence, clonal order is determined. SNVs/indels and CNVs mutated in ≥10% of the samples per subgroup are included. The dot size correlates with the percentage of patient samples for which two variants were observed in the same clone. The color codes the frequency by which variant 1 was observed being the ancestor to variant 2. Of note, if variant 1 is marked the ancestor of variant 2 in 0% of the patients, and variant 2 is equally marked the ancestor of variant 1 in 0%, this indicates that the two variants are always observed appearing at the same time. **C**) T-ALL pediatric. **D**) T-LBL pediatric.

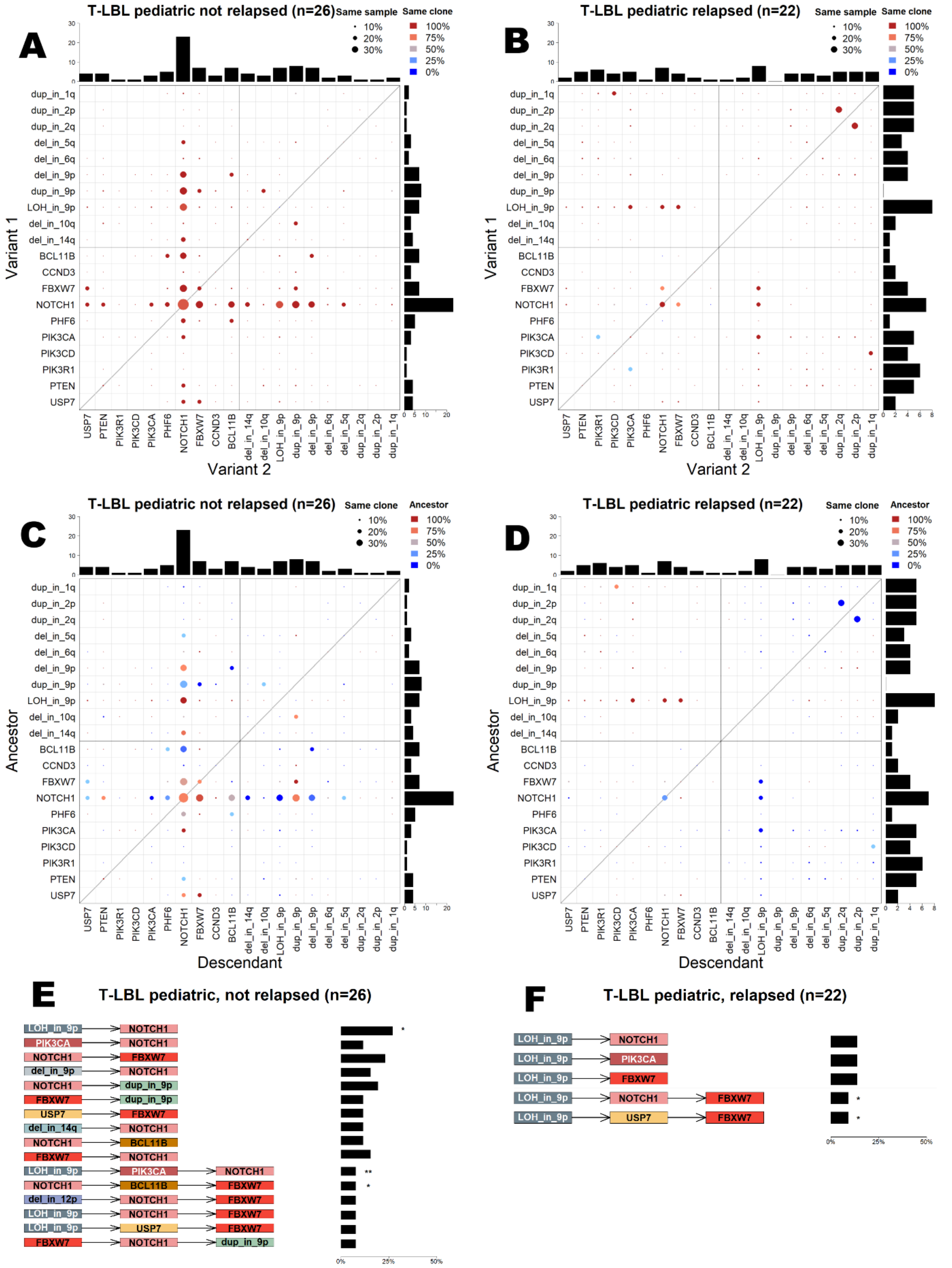

Supplementary Figure 10: Clonal co-occurrence and order of variants in pediatric T-LBL patients. Primary samples of not relapsed and relapsed patients are distinguished. Genes and chromosomes mutated in ≥10% of the samples in subgroup T-LBL pediatric are included. **A-B**) The dot size defines the percentage of patient samples for which clonal co-occurrence was detected. The color codes the frequency of variants observed in the same clone, that is the

same evolutionary branch. **A**) Not relapsed samples. **B**) Relapsed samples. **C-D**) For pairs of variants showing clonal co-occurrence, the analysis is continued by evaluating the order of variants. The dot size defines the percentage of patient samples for which two variants were observed in the same clone. The color codes the frequency by which variant 1 was observed being the ancestor to variant 2. **C**) Not relapsed samples. **D**) Relapsed samples. **E-F**) Continuing the analysis of clonal order of variants, conserved evolutionary trajectories are analyzed. Patterns of length 2 and 3 are considered. Trajectories observed in 10% (2 levels) and 5% (3 levels) of samples in the corresponding subgroups are included. Significant adjusted p-values are marked (*<0.05, **<0.01). **E**) Not relapsed samples. **F**) Relapsed samples.

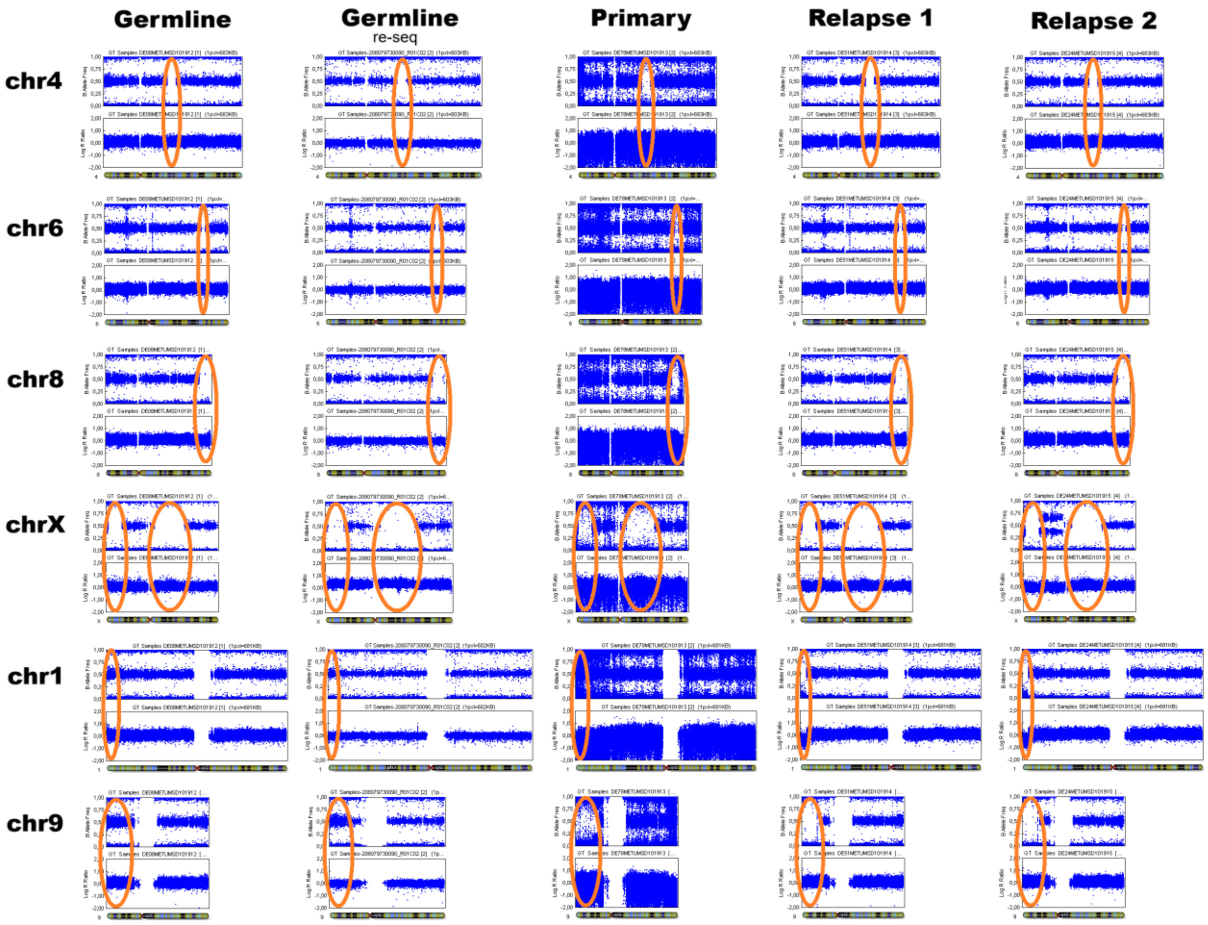

Supplementary Figure 11: Visualized SNP array data of patient T-LBL pediatric UPN190. Marked regions show the LOH on chromosomes 4, 6, 8 and X that are present at 100% CCF in the germline, the re-sequenced germline, the primary, relapse 1 and relapse 2 sample. A deletion in chromosome 1p and an LOH in chromosome 9p can be detected at low frequencies in the two germline samples and show an increasing frequency over time in primary, relapse 1 and relapse 2. Of note, primary sample shows a high level of noise. Still the marked variants can clearly be observed.

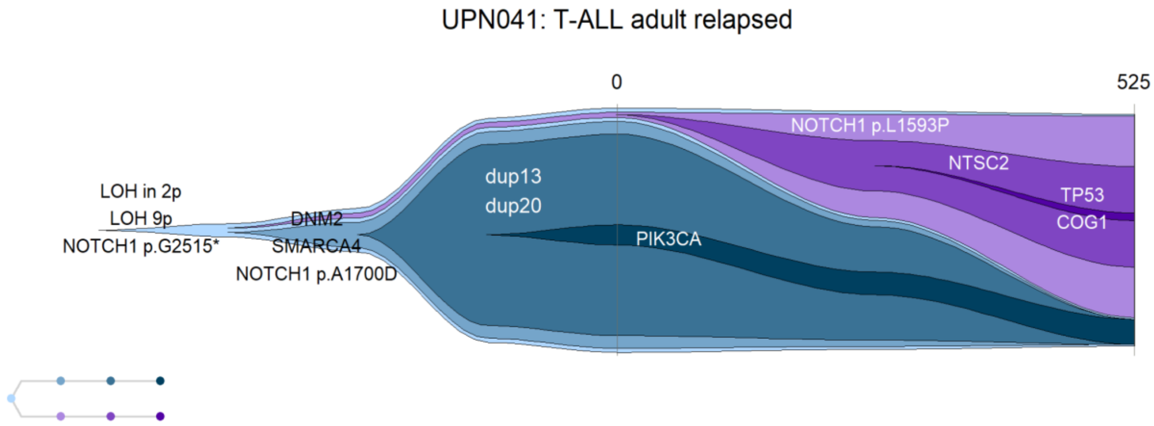

Supplementary Figure 12: Branching clonal evolution in a relapsed adult T-ALL patient. The initial clone is characterized by two CNVs (LOH in 2p and LOH9p) and a variant affecting *NOTCH1*. The subsequently evolving branch, dominant at primary time point, is characterized by a second variant affecting *NOTCH1* and further small variants and CNVs. A second branch, equally harboring an additional SNV affecting *NOTCH1* is already present at low frequency. Applied therapy successfully reduces the first branch. However, due to evolutionary advantage, the second branch expands, acquires further variants and likely causes the relapse observed at day 525.

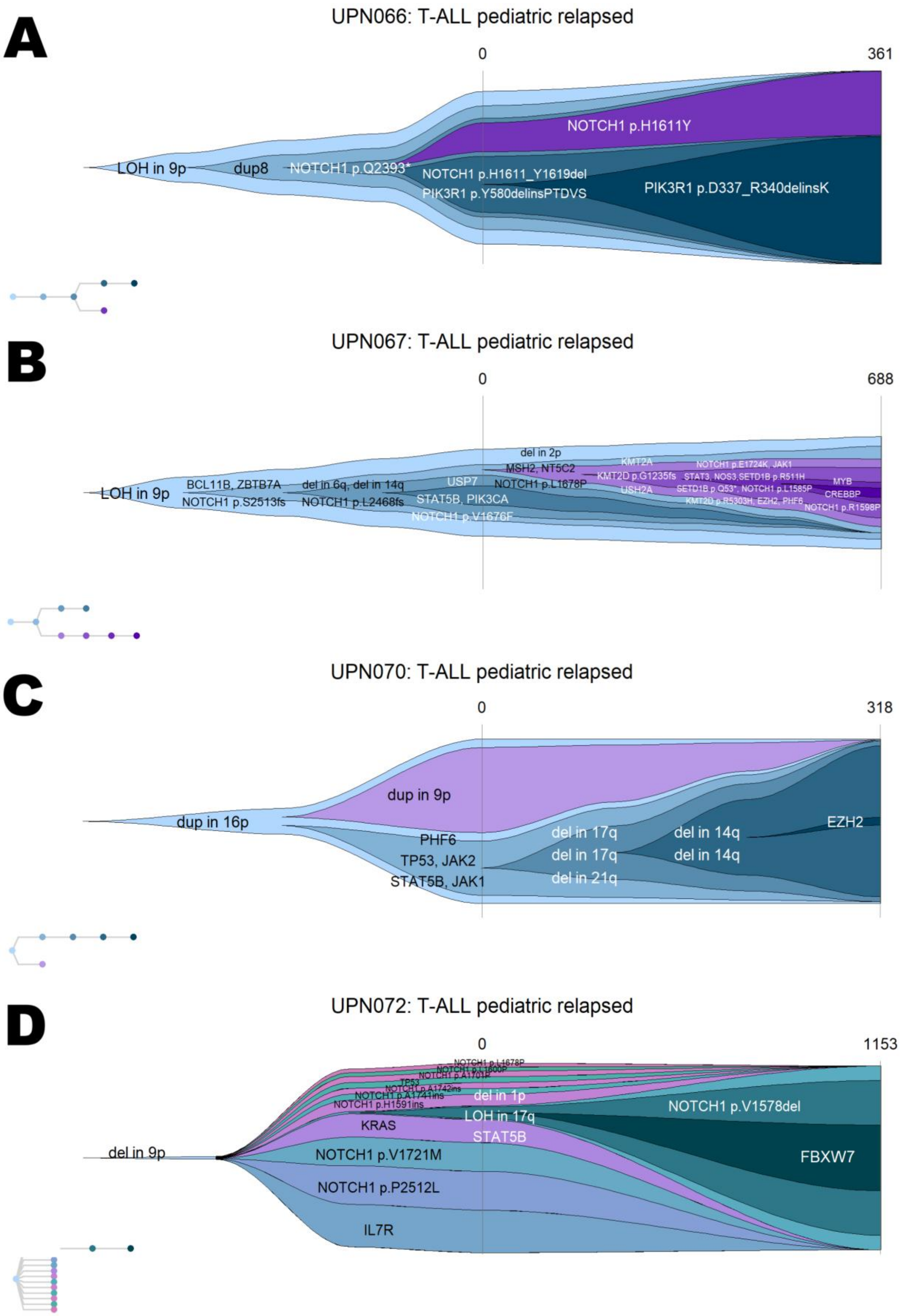

Supplementary Figure 13: Branching clonal evolution in relapsed pediatric T-ALL patients. A) Two main branches can be observed to co-exist at primary time point, each

characterized by a *NOTCH1* variant affecting p.H1611 (SNV vs del). Branch 1 acquires an additional variant in *PIK3R1*. However, at relapse, clones at both branches have increased in frequency without one dominating the other. **B**) At primary time point, only one branch is visible, characterized by several CNVs and small variants. Among other, three variants affecting *NOTCH1* can be observed, leading to both alleles being mutated in clone 3. Subsequent to primary diagnosis, a second branch evolves coming from clone 2, acquiring a huge number of additional variants. The first branch, showing the double-hit in *NOTCH1*, completely vanishes until relapse. **C**) Two main branches co-exist at primary time point. At relapse, however, branch 2, characterized by dup in 9q, completely vanishes – either by therapy or by being removed by branch 1, which acquired several additional CNVs and small variants. **D**) Following an initial deletion in 9p, a high number of branches, primarily characterized by variants in *NOTCH1*, develops. One branch, featuring *NOTCH1* (p.V1578del), acquires an additional SNV in *FBXW7* and successfully replaces all the other clones on the remaining 10 branches.

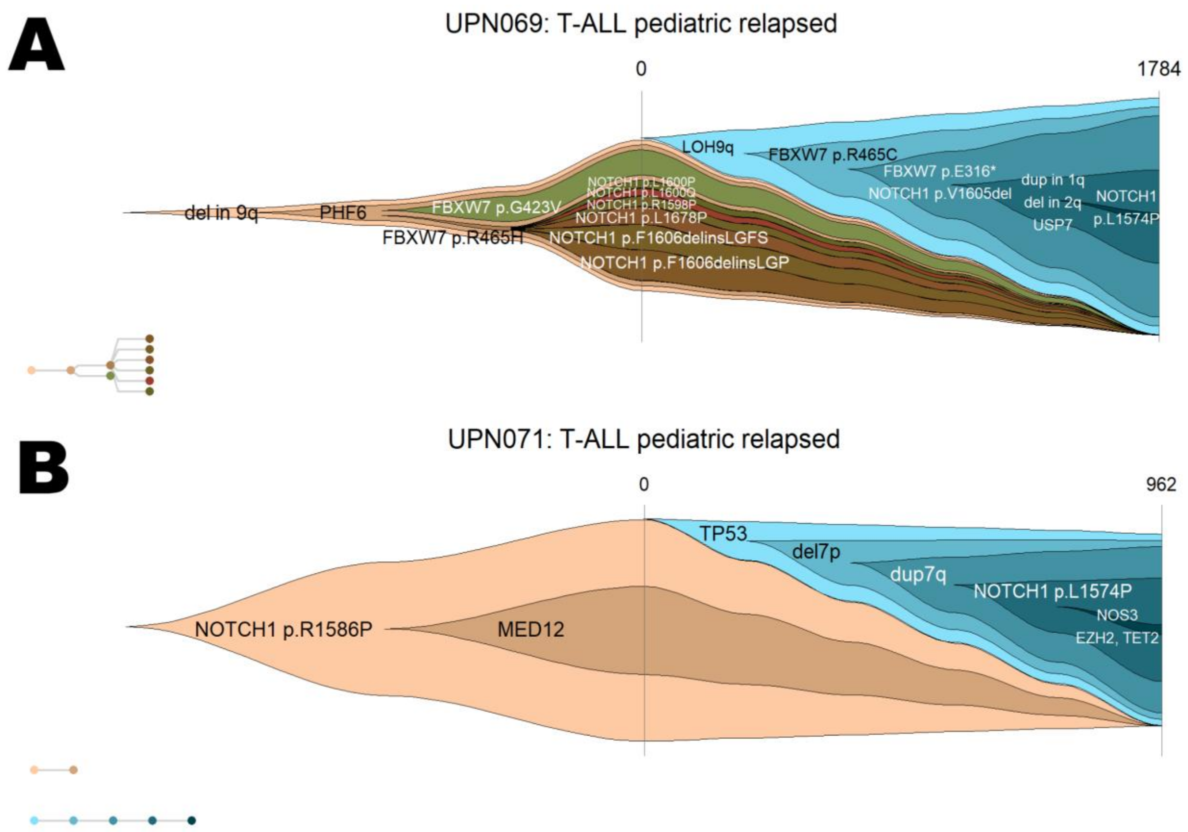

Supplementary Figure 14: Clonal evolution in two independent branches in pediatric T-ALL patients. A) At initial time point, a pattern of branching evolution can be observed. However, it is followed by a new independent branch of linear evolution towards relapse. None of the variants present at initial time point was detectable at relapse. Thus, another event not detectable by our analysis techniques, e.g. an SNV in a gene outside our target panel, might be the event linking both time points. As an alternative, considering the large time span between initial diagnosis and relapse, a second independent disease might be the reason. **B**) At initial time point, linear evolution can be observed. It is followed by a new independent branch of linear evolution, acquiring several CNVs and small variants, towards relapse.

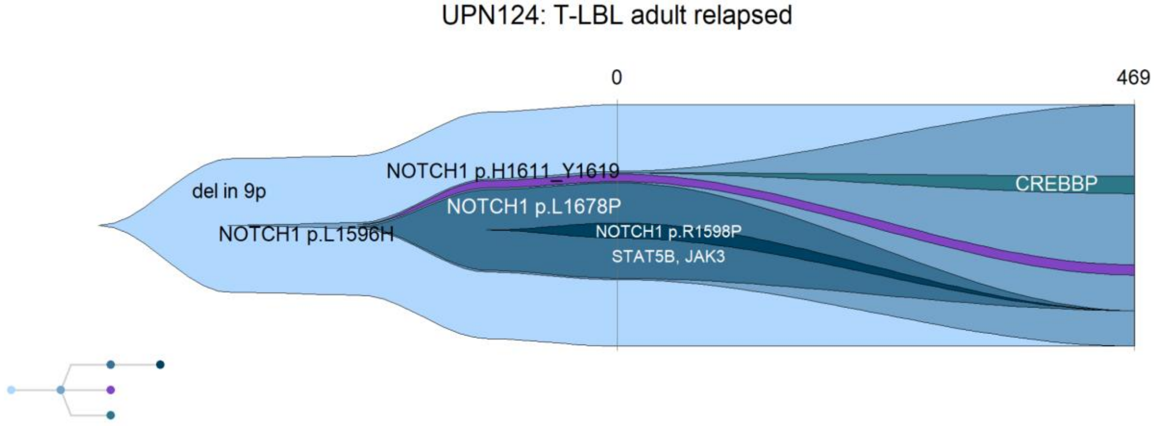

Supplementary Figure 15: Branching clonal evolution in a relapsed adult T-LBL patient. The initial clone is characterized by a deletion in chromosome 9p. Subsequently, four variants in *NOTCH1* are acquired, forming two different branches. Branch 1, characterized by 3 linearly developed SNVs in *NOTCH1,* dominates over branch 2 at primary time point. However, branch 1 completely vanishes until relapse. Nevertheless, branch 2 does not become dominant es well, but remains at low frequency. A newly developing third branch, characterized by an SNV in *CREBBP*, equally presents at low frequency. A majority of cells at relapse are characterized by initial del in 9p and *NOTCH1*(p.L1596H).

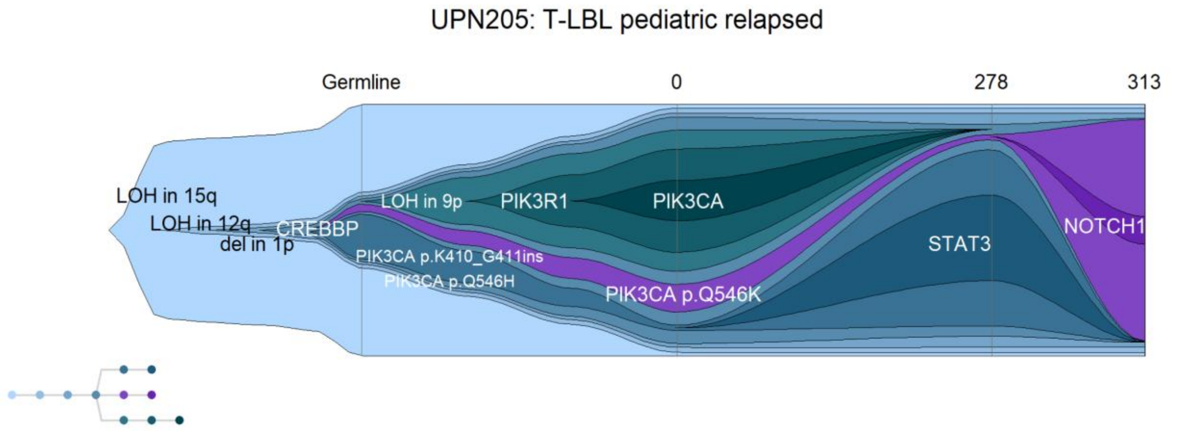

Supplementary Figure 16: Branching clonal evolution in a twice relapsed pediatric T-LBL patient. An LOH in chromosome 15q can already be detected in the germline sample. Further variants are present at low frequency. At initial diagnosis, three branches are visible, branch 3 (LOH in 9p, SNVs in *PIK3R1* and *PIK3CA*) dominating the disease. Towards relapse 1, branch 3 vanishes completely and branch 1, acquiring additional *STAT3* p.R31Q, becomes the prevailing clone. Towards relapse 2, however, this branch is also eradicated and branch 2 expands considerably, acquiring a variant in *NOTCH1* in a small sub-population (CCF=11%).

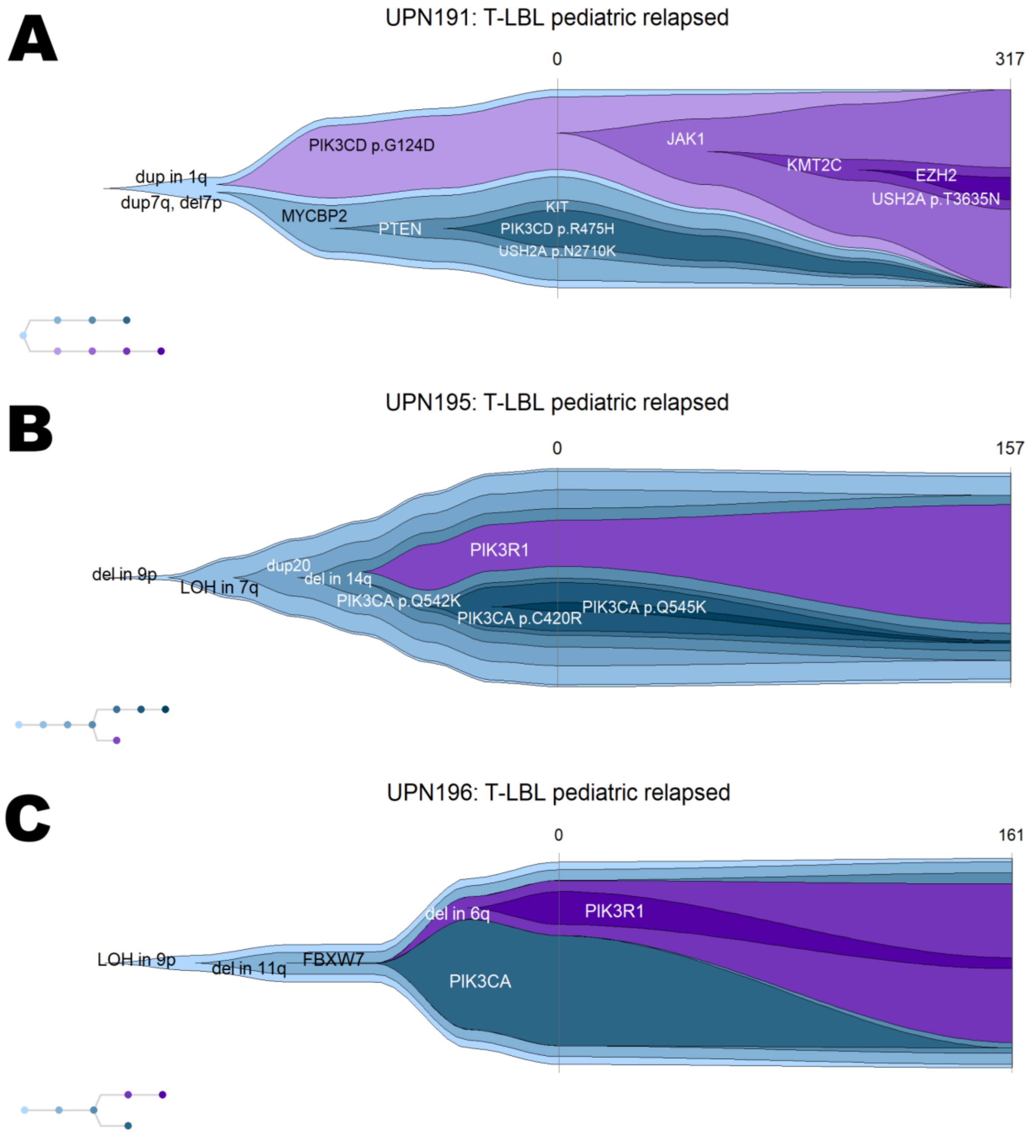

Supplementary Figure 17: Primary branching clonal evolution in relapsed pediatric T-LBL patients. At initial diagnosis, two branches co-exist at comparable CCFs. Towards relapse, however, one of the branches goes down, partly vanishing completely. The remaining branch expands. **A**) Starting from an initial clone showing 3 CNVs, two branches evolve. One, characterized by an SNV in *PIK3CD* acquires additional variants and completely dominates relapse. **B**) Clones with 3 variants in *PIK3CA* and one variant in *PIK3R1* co-exist. At time point 2, the clone harboring *PIK3R1* has expanded massively. Only few cells with mutated *PIK3CA* (p.Q542K) can still be detected. **C**) Clones with *PIK3CA* (branch 1) and del in 6q+*PIK3R1* (branch 2) co-exist. Without acquiring further variants, branch 2 dominates the relapse, while cells with mutated *PIK3CA* are no longer detectable.

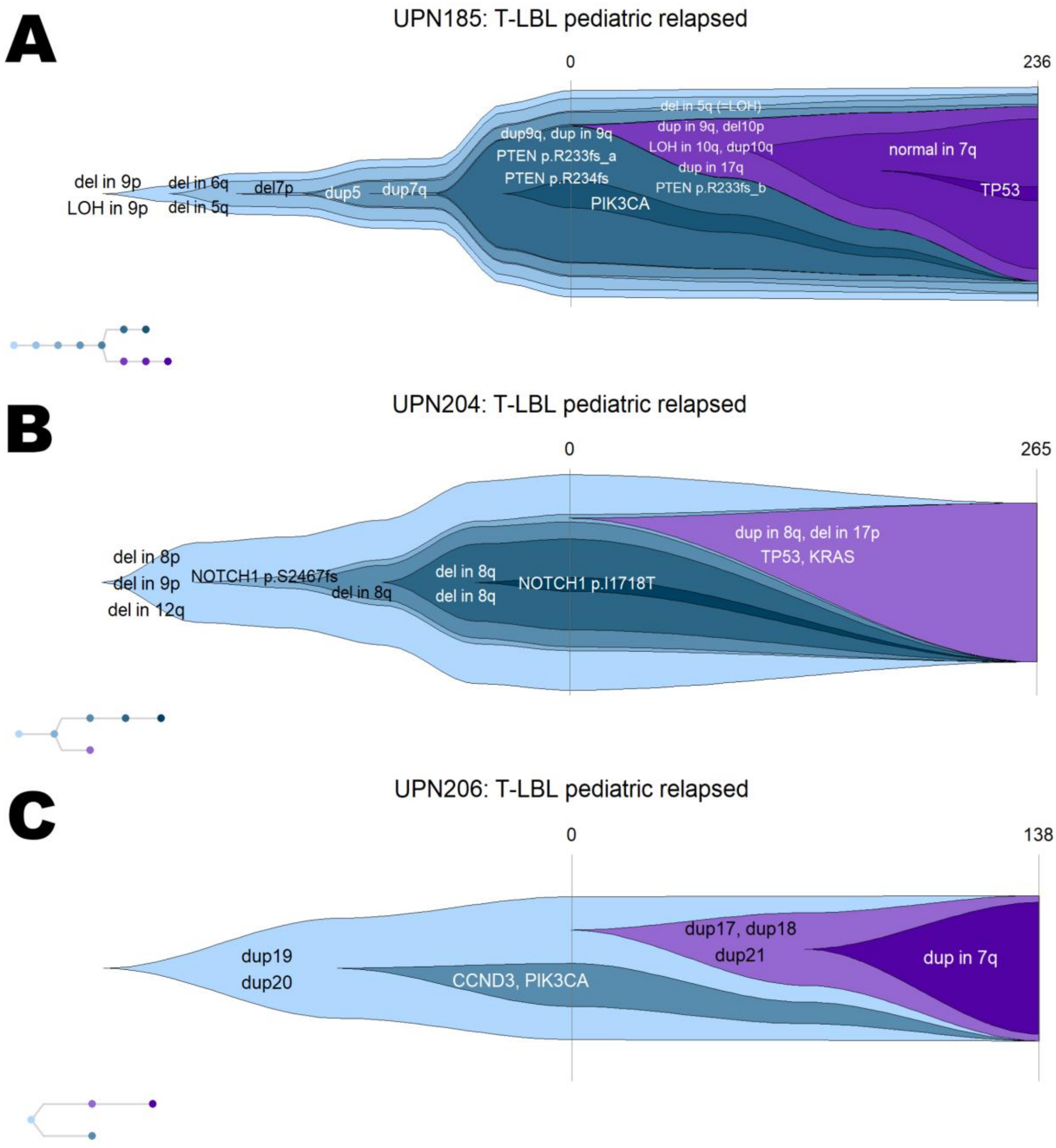

Supplementary Figure 18: Secondary branching clonal evolution with a major branch at initial diagnosis in relapsed pediatric T-LBL patients. At initial diagnosis, only one branch harboring several variants can be detected. Towards relapse, however, several clonal populations of this branch go down, while a new parallel branch evolves, dominating relapse. **A**) Initially, the patient acquires a high number of CNVs. The dominant clone at primary time point is characterized by dup9q, an additional dup in 9q, and two frame-shift variants in *PTEN*. The dominant clone at relapse harbors several additional CNVs, a re-mutation of 7q, as well as another frame-shift variant in *PTEN*. **B**) Three deletions and a frame-shift variant in *NOTCH1* are initially acquired. The dominant clone at time point 2 features, among others, an SNV in *KRAS* and a double-hit affecting *TP53* (SNV + del in 17p). **C**) The clonal evolution is mainly characterized by CNVs. The dominant clonal population at relapse additionally acquired dup17, dup18, dup21 and dup in 7q.

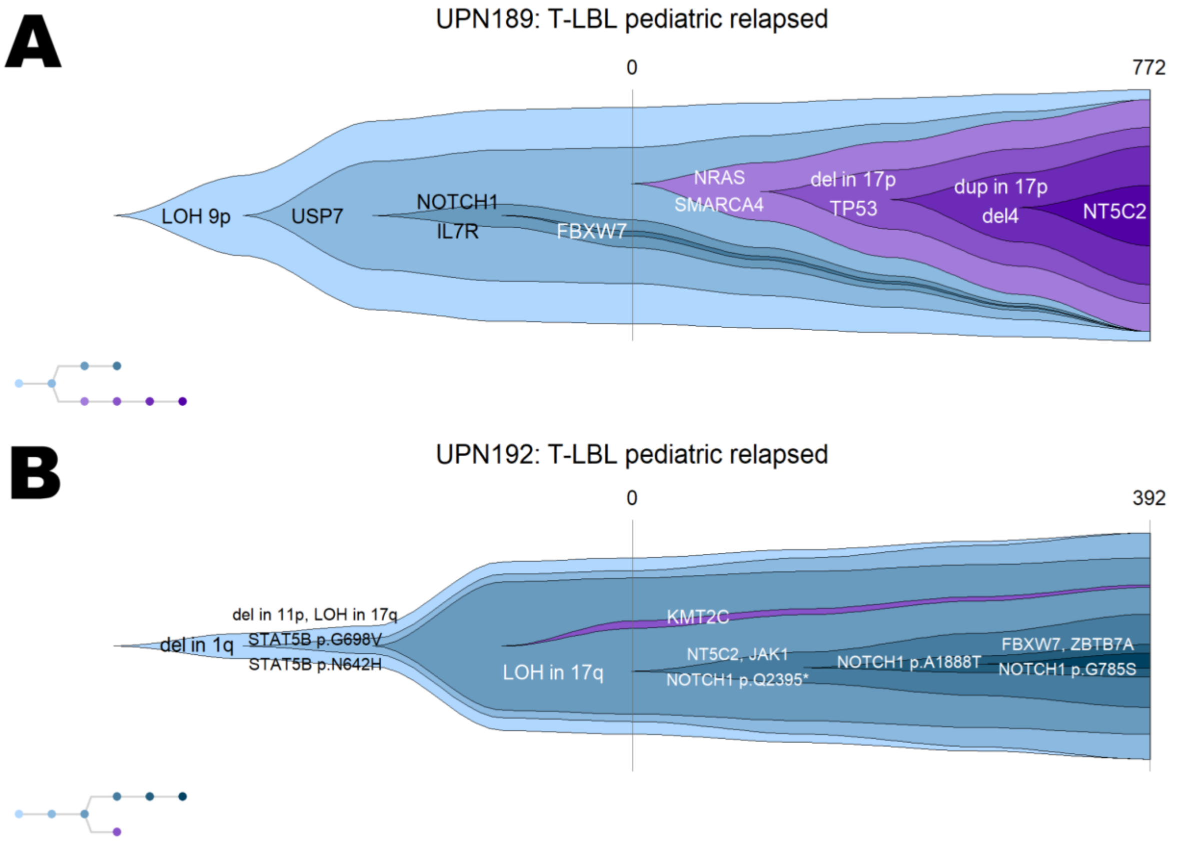

Supplementary Figure 19: Secondary branching clonal evolution with a minor branch at initial diagnosis in relapsed pediatric T-LBL patients. At initial diagnosis, only one branch, including a small clonal sub-population, can be detected. Towards relapse, this sub-population goes down or is mainly reduced, while the preceding clone acquires several new variants and expands considerably. **A**) A sub-clone characterized by variants in *NOTCH1*, *IL7R* and *FBXW7* (CCF=11%) completely vanishes towards relapse. The newly developing, dominating branch is characterized, among others, by double-hit of *TP53* (SNV + del in 17p). **B**) A minor sub-clone with mutated *KMT2C* at initial diagnosis is further reduced in frequency towards relapse. Instead, a new branch, mainly characterized by variants in *NOTCH1*, evolves.

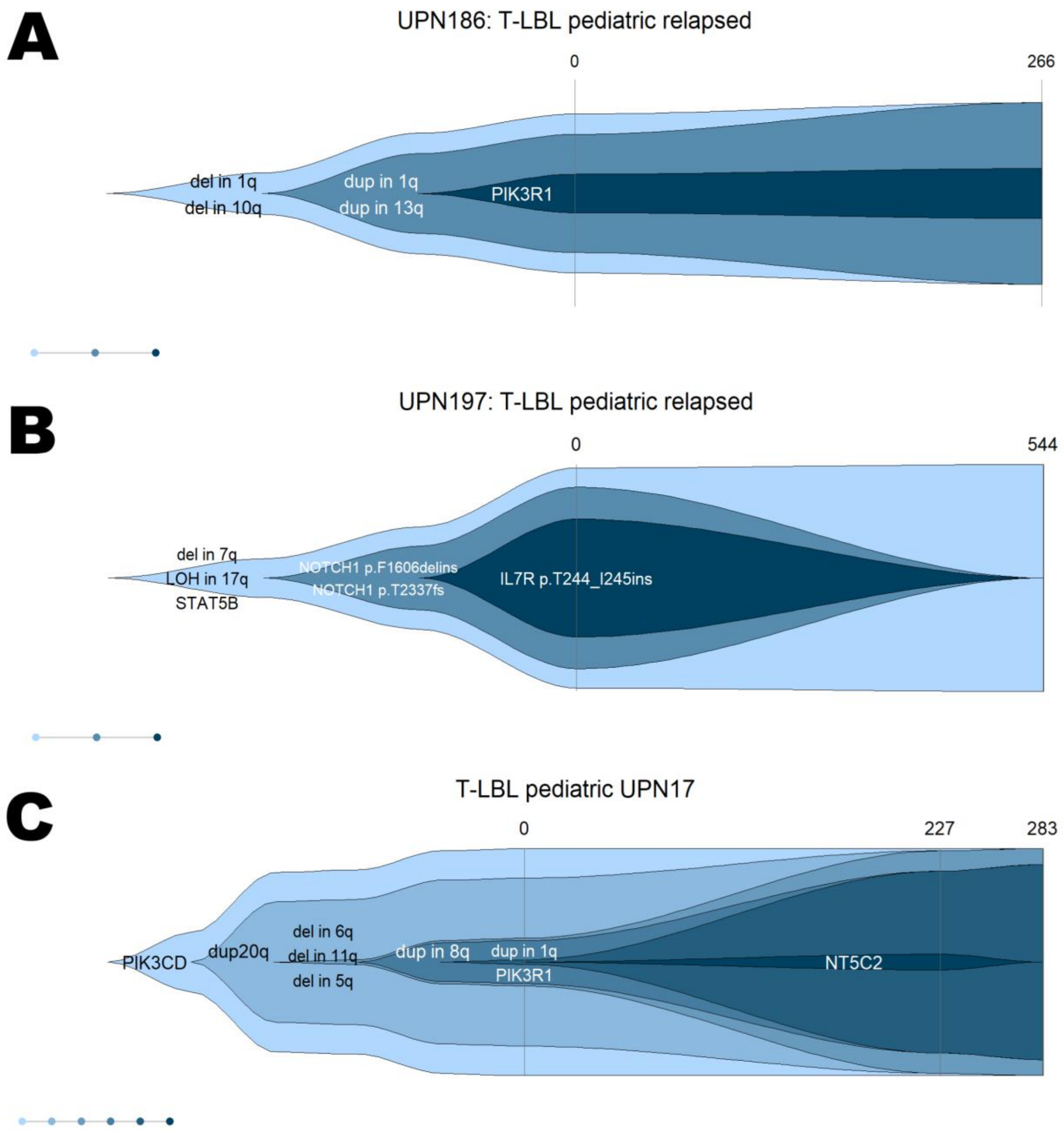

Supplementary Figure 20: Linear clonal evolution in relapsed pediatric T-LBL patients. **A**) Three clones, characterized by CNVs and mutated *PIK3R1* can be detected at initial diagnosis. No further variants are acquired towards relapse. Instead, the existing clones further expand. **B**) Variants are organized in three clones. The second, notably, by two variants in *NOTCH1*. Towards relapse, this clone, including its sub-clone harboring an insertion in gene *IL7R*, completely vanishes. Only the initial clone, not featuring any variants in *NOTCH1*, remains. **C**) Clones characterized by several CNVs and small variants in *PIK3CD* and *PIK3R1* are present at initial diagnosis. At relapse 1, a sub-clonal population additionally harboring an SNV in *NT5C2* can be observed. However, at second relapse, the clone is no longer detectable. The preceding clones further expanded.

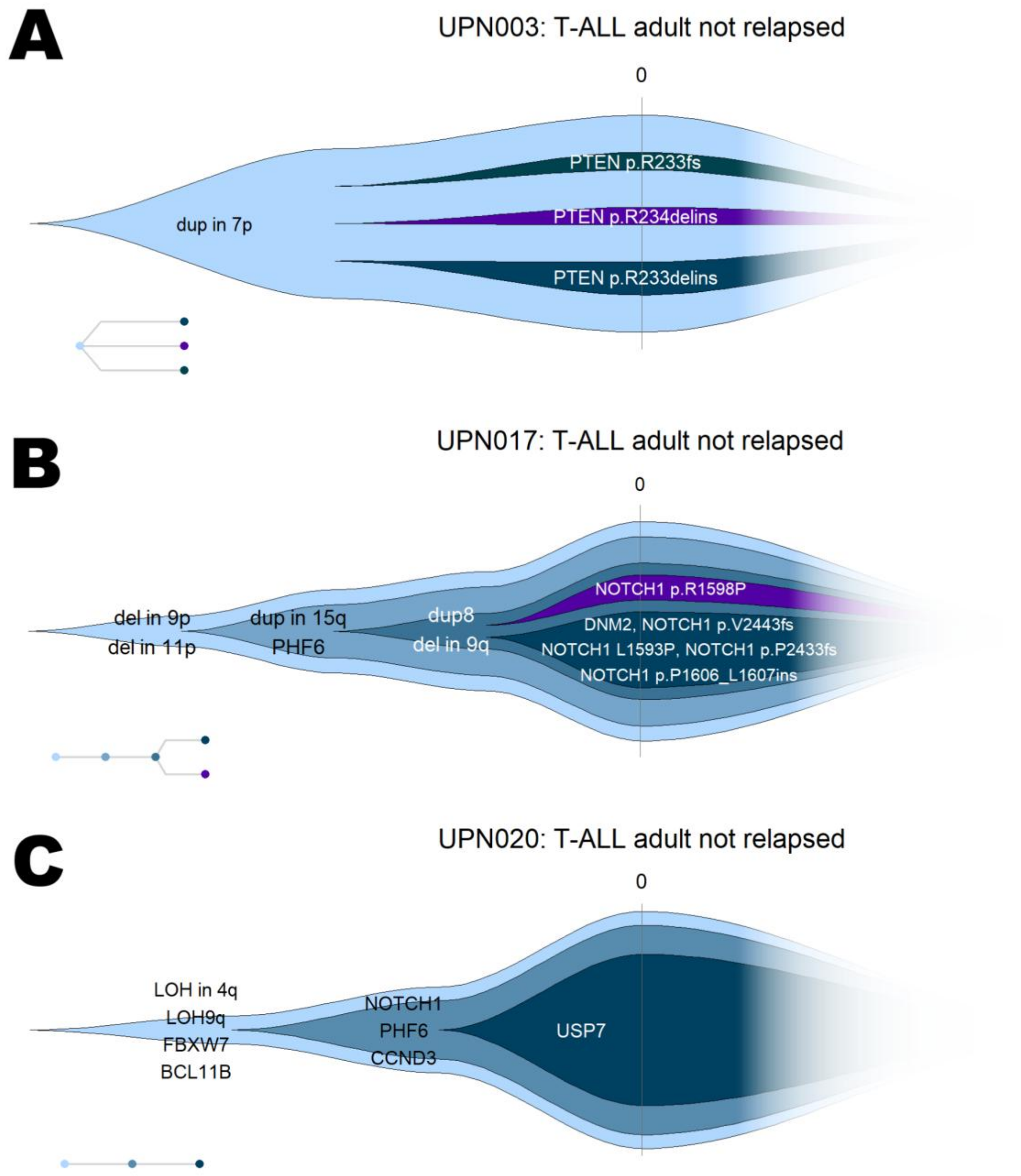

Supplementary Figure 21: Clonal evolution in not relapsed adult T-ALL patients. A) Branching evolution. Three clones emerge, each characterized by a variant in *PTEN*. Sequencing data clearly shows that the variants do not lie on the same reads and thus, do not cluster together. Instead, three branches can be observed. Low CCFs indicates no significant evolutionary advantage of any clone. **B**) Branching evolution. Initial clones are characterized by CNVs. Two branching clones evolve, each characterized by a pattern of *NOTCH1* variants. The larger of the two clones shows deficient *NOTCH1*. **C**) Linear evolution. The initial clone is characterized by both CNVs (LOH in 4q, LOH9q) and point mutations (*FBXW7* and *BCL11B*). In the second clone, among others, a variant affecting *NOTCH1* emerges.

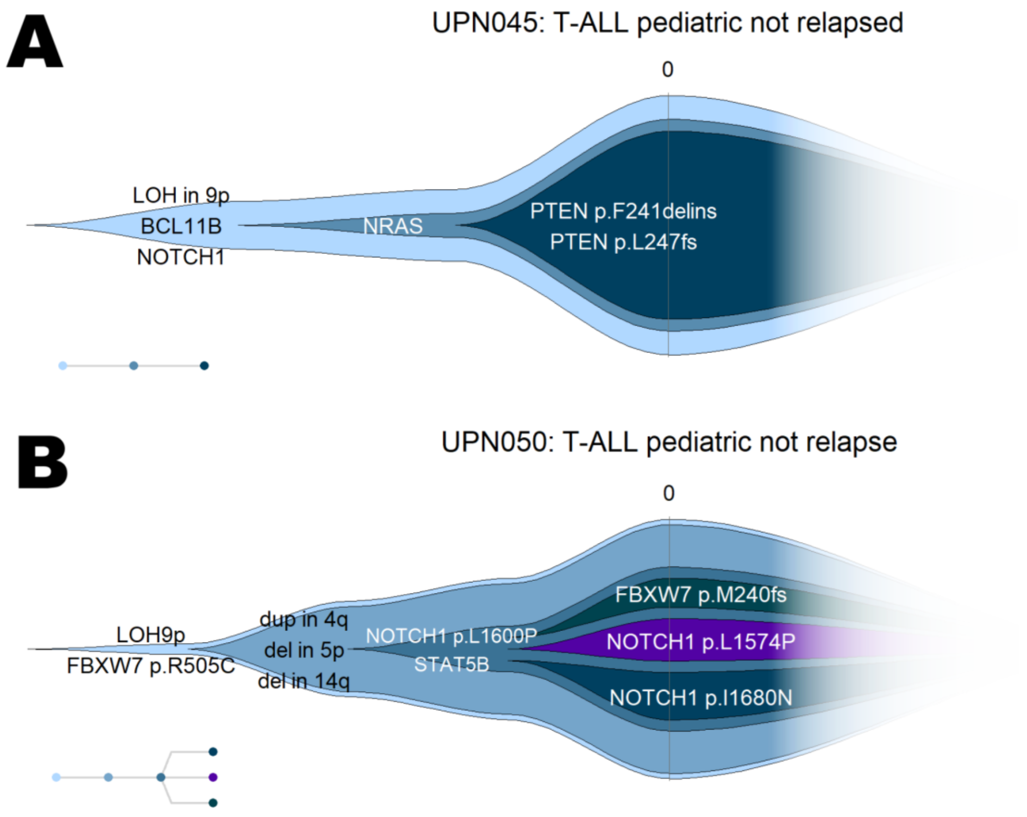

Supplementary Figure 22: Clonal evolution in not relapsed pediatric T-ALL patients. A) Linear evolution. The initial clone is, among others, characterized by mutated *NOTCH1*. Subsequent clones harbor variants in *NRAS* and *PTEN*, where the two variants in *PTEN* affect different alleles. **B**) Branching evolution. Three clones of comparable CCF evolve, characterized by mutated *NOTCH1* and FBXW7. Clone 5 (purple) features a bi-allelic event, likely leading to deficient *NOTCH1*.

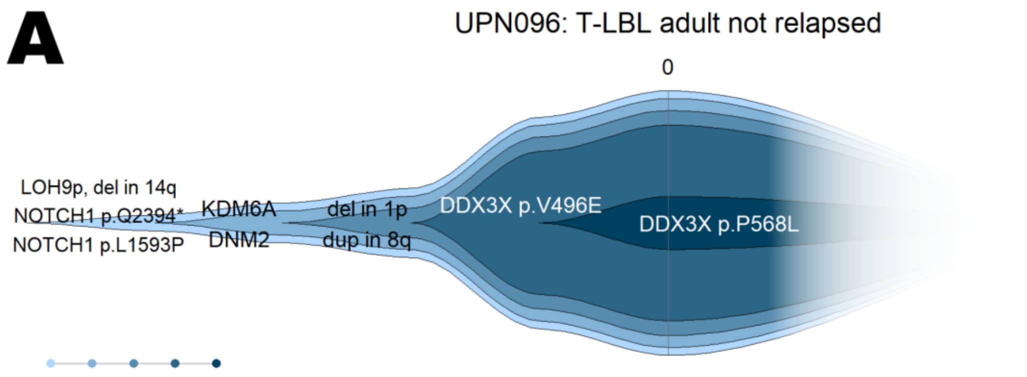

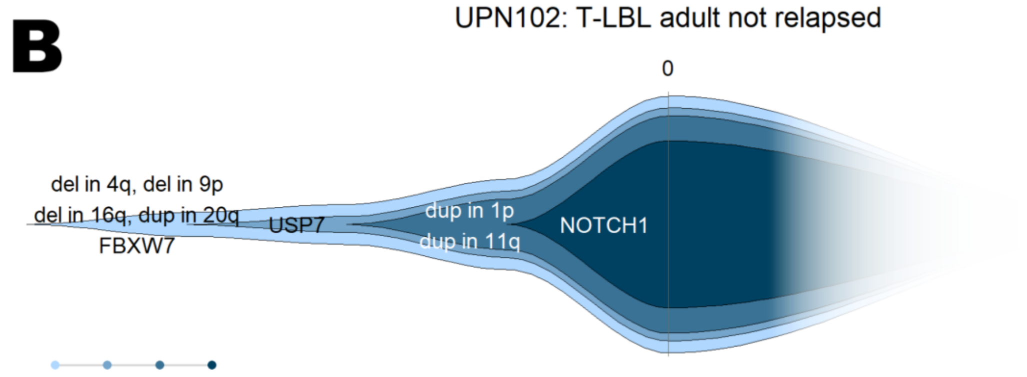

Supplementary Figure 23: Clonal evolution in not relapsed adult T-LBL patients. A) Linear evolution. The first clone shows a combination of CNVs (LOH9p and del in 14q) and two variants in *NOTCH1*. The male patient has likely deficient *KDM6A* and *DDX3X*, both located on the X chromosome. **B**) Linear evolution. Several CNVs, characterizing the initial clone can be observed. Over time, further CNVs are acquired (dup in 1p and dup in 11q). However, the initial clone is also characterized by a variant in *FBXW7*, the dominating fourth clone by a variant in *NOTCH1*, both previously reported as being associated with good prognosis.

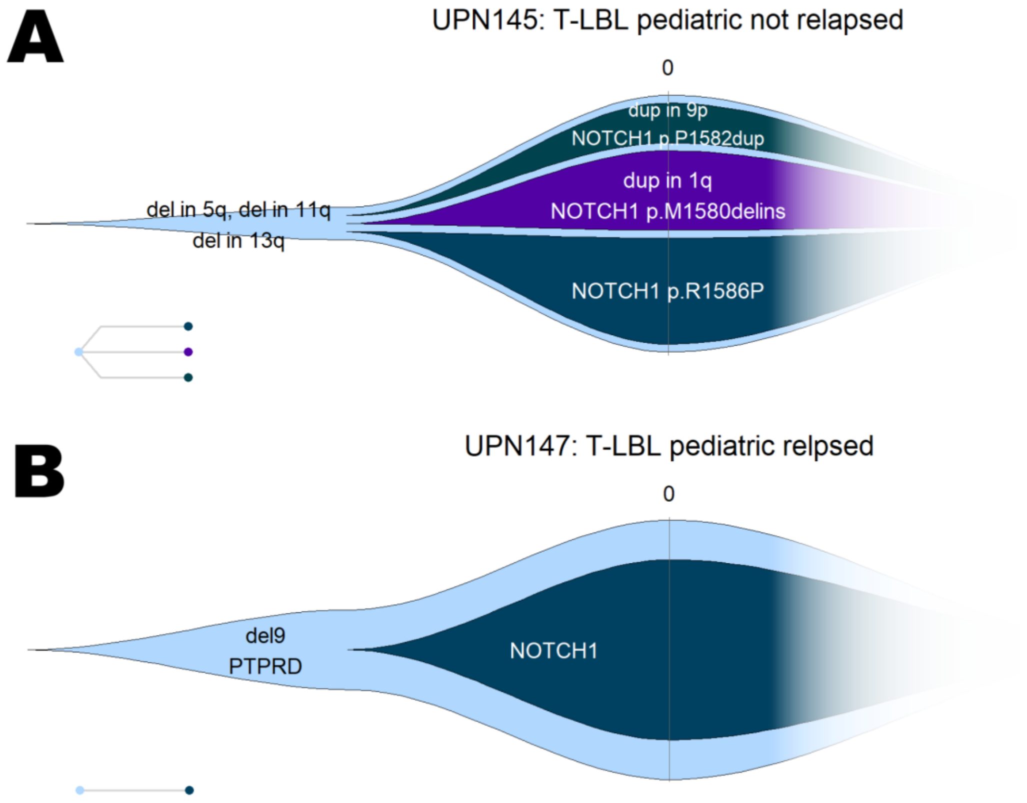

Supplementary Figure 24: Clonal evolution in not relapsed pediatric T-LBL. A) Branching evolution. Following three initial CNVs, three branches develop. Each clone is characterized by a different variant affecting *NOTCH1*. Sequencing data clearly show that the variants are not located on the same read and thus, do not cluster together. **B**) Linear evolution. The initial clone is characterized by a deletion of chromosome 9, overlapping a point mutation in *PTPRD* and thus likely leading to deficient protein. The subsequent frame-shift variant affecting *NOTCH1* is equally overlapping del9.

4. References

Heipertz AE, et al. Outcome of children and adolescents with relapsed/refractory/progressive malignancies treated with molecularly informed targeted drugs in the Pediatric Precision Oncology Registry INFORM. JCO Precis Oncol. 2023;7:e2300015.

Peterziel H, et al. Drug sensitivity profiling of 3D tumor tissue cultures in the pediatric precision oncology program INFORM. NPJ Precis Oncol. 2022;6:94.

van Tilburg CM, et al. The Pediatric Precision Oncology INFORM Registry: Clinical outcome and benefit for patients with very high-evidence targets. Cancer Discov. 2021;11:2764-2779.

Worst BC, et al. Next-generation personalised medicine for high-risk paediatric cancer patients – The INFORM pilot study. Eur J Cancer. 2016;65:91-101.

Ruether C, et al. Design of a targeted next-generation DNA sequencing panel for pediatric T-cell lymphoblastic lymphoma to unravel biology and optimize treatment. Genes Chromosomes Cancer. 2022;61:459-470.

Li H. Aligning sequence reads, clone sequences and assembly contigs with BWA-MEM. ArXiv. 2013;1303.

Sandmann S, et al. appreci8: a pipeline for precise variant calling integrating 8 tools. Bioinformatics. 2018;34:4205-4212.

Robinson JT, et al. Variant Review with the Integrative Genomics Viewer. Cancer Res. 2017;77(21):e31-e34.

R Core Team. R: A Language and Environment for Statistical Computing. R Foundation for Statistical Computing, Vienna, Austria. 2024.

Skidmore ZL, et al. GenVisR: Genomic visualizations in R. Bioinformatics. 2016;32:3012-3014.

da Silva-Coelho P, et al. Clonal evolution in myelodysplastic syndromes. Nat Commun. 2017;8:15099.

Reutter K, et al. Reconstructing clonal evolution in relapsed and non-relapsed Burkitt lymphoma. Leukemia. 2021;35:639-643.

Sandmann S, et al. Clonal Evolution at First Sight: A Combined Visualization of Diverse Diagnostic Methods Improves Understanding of Leukemic Progression. Front Oncol. 2022;12:888114.

Sandmann S, et al. clevRvis: visualization techniques for clonal evolution. Gigascience. 2022;12:.

Schwede M, et al. Mutation order in acute myeloid leukemia identifies uncommon patterns of evolution and illuminates phenotypic heterogeneity. Leukemia. 2024;38:1501-1510.

Sandmann S, et al. Identifying conserved trajectories in clonal evolution trees [version 1; not peer reviewed]. F1000Research. 2024;13:870 (poster).
