# Supplementary material for: Molecular variants, clonal evolution and clinical relevance in pediatric and adult T-cell lymphoblastic neoplasia
Source: Blood Cancer J. 2026 Apr 2;16(1):57. doi: 10.1038/s41408-026-01488-w (PMC13066408; doi:10.1038/s41408-026-01488-w)
Supplement: Supplementary file 2 — Supplemental Data 1 [file 41408_2026_1488_MOESM2_ESM.pdf]

ID	Chr	Pos	Ref	Alt	Germline #REF	Germline #ALT	Germline DP	Germline VAF	Tumor #REF	Tumor #ALT	Tumor DP	Tumor VAF	Gene	ENST	Type	Variant
UPN001	9	139399367	G	GTTA	1498	0	1501	0.00 %	1966	781	1974	39.56 %	NOTCH1	ENST00000277541	disruptive_inframe_insertion	p.Phe1592_Leu1593insAsn/c.4775_4776insTAA
UPN001	X	133527976	T	TTGGGGGC	941	0	946	0.00 %	732	177	738	23.98 %	PHF6	ENST00000394292;ENST00000370803;ENST00000332070;ENST00000370799;ENST00000416404;ENST00000370800	frameshift_variant	p.Ser138fs/c.412_413insTGGGGGC;p.Ser104fs/c.310_311insTGGGGGC
UPN001	4	153247366	C	T	988	0	991	0.00 %	719	226	946	23.89 %	FBXW7	ENST00000281708;ENST00000296555;ENST00000263981;ENST00000603548;ENST00000393956;ENST00000603841	missense_variant	p.Arg479Gln/c.1436G>A;p.Arg361Gln/c.1082G>A;p.Arg399Gln/c.1196G>A;p.Arg303Gln/c.908G>A
UPN001	16	3801697	CCCCAGAGAAAATGACAGGACGGTACTTACGTCTGGGGCTGTGAAGGGTCGTCA	C	674	0	675	0.00 %	569	41	611	6.71 %	CREBBP	ENST00000262367;ENST00000382070;ENST00000573517	splice_donor_variant+disruptive_inframe_deletion+splice_region_variant+intron_variant	p.Asp1253_Thr1260del/c.3756_3779+29delTGACGACCCTTCACAGCCCCAGACGTAAGTACCGTCCTGTCATTTTCTCTGGG;p.Asp1215_Thr1222del/c.3642_3665+29delTGACGACCCTTCACAGCCCCAGACGTAAGTACCGTCCTGTCATTTTCTCTGGG;p.Asp21_Thr28del/c.60_83+29delTGACGACCCTTCACAGCCCCAGACGTAAGTACCGTCCTGTCATTTTCTCTGGG
UPN002	X	133551254	GTGTTAA	GTAG	619	3	620	0.48 %	190	312	502	62.15 %	PHF6	ENST00000394292;ENST00000370803;ENST00000332070;ENST00000370799;ENST00000416404	missense_variant+disruptive_inframe_deletion	p.Val299_Lys300delinsArg/c.895_899delGTTAAinsAG;p.Val298_Lys299delinsArg/c.892_896delGTTAAinsAG;p.Val264_Lys265delinsArg/c.790_794delGTTAAinsAG
UPN002	1	115258747	C	T	2009	0	2011	0.00 %	937	445	1385	32.13 %	NRAS	ENST00000369535	missense_variant	p.Gly12Asp/c.35G>A
UPN003	10	89717671	AC	AGGGATCAACAGGTACGCA	965	0	970	0.00 %	814	60	910	6.59 %	PTEN	ENST00000371953	frameshift_variant+missense_variant	p.Arg233fs/c.697delCinsGGGATCAACAGGTACGCA

UPN003	10	89717676	GGGAA	CACTCCCCTTTTAAGCCGGAGAGGAT	920	4	922	0.43 %	847	32	879	3.64 %	PTEN	ENST00000371953	stop_gained+missense_variant+inframe_insertion	p.Arg234_Glu235delinsProLeuProPheTerAlaGlyGluAsp/c.701_705delGGGAAinsCACTCCCCTTTTAAGCCGGAGAGGAT
UPN003	10	89717671	AC	ATTCCGCT	965	2	965	0.21 %	814	30	910	3.30 %	PTEN	ENST00000371953	stop_gained+disruptive_inframe_insertion	p.Arg233delinsPheArgTer/c.697delCinsTTCCGCT
UPN004	X	70341524	T	TGTTCG	900	0	905	0.00 %	786	577	814	70.88 %	MED12	ENST00000333646;ENST00000374102;ENST00000374080	frameshift_variant	p.Ile320fs/c.959_960insGTTCG
UPN004	5	67589599	T	TCCCCTC	592	0	592	0.00 %	490	160	491	32.59 %	PIK3R1	ENST00000396611;ENST00000521381;ENST00000521657;ENST00000274335;ENST00000320694;ENST00000521409;ENST00000336483;ENST00000519025;ENST00000523872	disruptive_inframe_insertion	p.Thr454_Gln455insProLeu/c.1363_1364insCCCTCC;p.Thr154_Gln155insProLeu/c.463_464insCCCTCC;p.Thr91_Gln92insProLeu/c.274_275insCCCTCC;p.Thr184_Gln185insProLeu/c.553_554insCCCTCC;p.Thr127_Gln128insProLeu/c.382_383insCCCTCC
UPN004	10	89717674	A	ACGGG	827	0	830	0.00 %	823	263	827	31.80 %	PTEN	ENST00000371953	frameshift_variant	p.Glu235fs/c.700_703dupCGGG
UPN004	10	89717712	CGTT	CTTCCCTTTGTG	841	0	844	0.00 %	825	235	828	28.38 %	PTEN	ENST00000371953	frameshift_variant	p.Leu247fs/c.737_740delinsTTCCCTTTGTG
UPN004	17	30300212	GA	G	699	0	700	0.00 %	739	241	983	24.52 %	SUZ12	ENST00000322652;ENST00000580398	frameshift_variant	p.Val186fs/c.555delA;p.Val163fs/c.486delA
UPN005	21	36231873	T	A	1048	1	1051	0.10 %	660	573	1234	46.43 %	RUNX1	ENST00000300305;ENST00000344691;ENST00000325074;ENST00000437180;ENST00000399240;ENST00000358356;ENST00000399237;ENST00000486278;ENST00000482318	stop_gained;splice_region_variant	p.Lys171*/c.511A>T;p.Lys144*/c.430A>T;p.Lys159*/c.475A>T;p.Lys147*/c.439A>T;c.*101A>T
UPN005	9	139397640	C	T	1204	2	1208	0.17 %	1004	792	1799	44.02 %	NOTCH1	ENST00000277541	missense_variant	p.Val1721Met/c.5161G>A

UPN005	19	10940813	C	T	1109	0	1112	0.00 %	743	571	1319	43.29 %	DNM2	ENST00000314646;ENST00000585892;ENST00000359692;ENST00000389253;ENST00000355667;ENST00000408974;ENST00000589106	stop_gained	p.Gln768*/c.2302C>T;p.Gln764*/c.2290C>T;p.Gln56*/c.166C>T
UPN005	16	9017254	G	GGACCCCA	1085	0	1090	0.00 %	1208	479	1212	39.52 %	USP7	ENST00000344836;ENST00000563961;ENST00000381886;ENST00000542333;ENST00000566004;ENST00000569230;ENST00000566273;ENST00000564117	frameshift_variant	p.Glu68fs/c.200_201insTGGGGTC;p.Glu52fs/c.152_153insTGGGGTC;p.Glu10fs/c.26_27insTGGGGTC;p.Glu54fs/c.158_159insTGGGGTC
UPN005	5	35874570	A	ACTAGGGCGCGGGTGC	1102	0	1106	0.00 %	1265	165	1271	12.98 %	IL7R	ENST00000303115	inframe_insertion	p.Leu243_Thr244insGlyArgGlyCysLeu/c.729_730insGGGCGCGGGTGCCTA
UPN006	no Variants															
UPN007	X	133549136	C	T	695	0	695	0.00 %	625	262	889	29.47 %	PHF6	ENST00000394292;ENST00000370803;ENST00000332070;ENST00000370799;ENST00000416404;ENST00000370800	stop_gained	p.Arg275*/c.823C>T;p.Arg274*/c.820C>T;p.Arg240*/c.718C>T
UPN007	14	99723911	T	TTTCC	1306	0	1309	0.00 %	1183	338	1187	28.48 %	BCL11B	ENST00000357195;ENST00000345514	frameshift_variant	p.Val109fs/c.320_323dupGGAA
UPN007	X	44942749	TGC	TGGG	608	2	609	0.33 %	653	245	898	27.28 %	KDM6A	ENST00000382899;ENST00000377967;ENST00000536777;ENST00000543216;ENST00000414389;ENST00000433797	frameshift_variant+missense_variant	p.Arg1118fs/c.3352delCinsGG;p.Arg1111fs/c.3331delCinsGG;p.Arg1066fs/c.3196delCinsGG;p.Arg1032fs/c.3094delCinsGG;p.Arg708fs/c.2122delCinsGG;p.Arg753fs/c.2257delCinsGG
UPN008	17	30323881	A	G	655	0	655	0.00 %	472	218	692	31.50 %	SUZ12	ENST00000322652;ENST00000580398	missense_variant	p.His620Arg/c.1859A>G;p.His597Arg/c.1790A>G

UPN008	19	17945969	C	T	1491	0	1493	0.00 %	1141	527	1673	31.50 %	JAK3	ENST00000458235;ENST00000527670;ENST00000534444	missense_variant	p.Arg657Gln/c.1970G>A
UPN008	17	30320342	T	A	679	0	680	0.00 %	457	195	653	29.86 %	SUZ12	ENST00000322652;ENST00000580398	missense_variant	p.Ile428Lys/c.1283T>A;p.Ile405Lys/c.1214T>A
UPN008	X	133511772	ACCA	ACCGATCGCTTGCCC	900	0	901	0.00 %	876	136	877	15.51 %	PHF6	ENST00000394292;ENST00000370803;ENST00000332070;ENST00000370799;ENST00000416404;ENST00000370800	frameshift_variant	p.His43fs/c.128delAinsGATCGCTTGCCC
UPN008	1	65309803	G	A	1209	0	1209	0.00 %	1164	153	1320	11.59 %	JAK1	ENST00000342505	missense_variant	p.Leu783Phe/c.2347C>T
UPN008	1	65305426	G	T	1514	0	1518	0.00 %	1514	85	1603	5.30 %	JAK1	ENST00000342505	missense_variant	p.Thr901Lys/c.2702C>A
UPN009	4	106197354	G	A	1227	57	1285	4.44 %	1026	291	1325	21.96 %	TET2	ENST00000513237;ENST00000540549;ENST00000380013	missense_variant	p.Arg1917Lys/c.5750G>A;p.Arg1896Lys/c.5687G>A
UPN009	21	36252938	C	CGGGA	1406	0	1408	0.00 %	1287	200	1292	15.48 %	RUNX1	ENST00000300305;ENST00000344691;ENST00000325074;ENST00000437180;ENST00000399240;ENST00000358356;ENST00000399237;ENST00000486278;ENST00000455571	frameshift_variant	p.Ala142fs/c.423_424insTCCC;p.Ala115fs/c.342_343insTCCC;p.Ala130fs/c.387_388insTCCC;p.Ala118fs/c.351_352insTCCC;p.Ala129fs/c.384_385insTCCC
UPN010	X	133551319	C	T	377	0	379	0.00 %	51	409	460	88.91 %	PHF6	ENST00000394292;ENST00000370803;ENST00000332070;ENST00000370799;ENST00000416404	stop_gained	p.Arg320*/c.958C>T;p.Arg319*/c.955C>T;p.Arg285*/c.853C>T
UPN010	17	40354794	T	G	1157	1	1161	0.09 %	1032	803	1837	43.71 %	STAT5B	ENST00000293328	missense_variant	p.Ile704Leu/c.2110A>C
UPN010	9	139399365	A	G	1230	1	1235	0.08 %	1209	640	1852	34.56 %	NOTCH1	ENST00000277541	missense_variant	p.Leu1593Pro/c.4778T>C
UPN010	12	25398284	C	T	890	0	891	0.00 %	1268	185	1460	12.67 %	KRAS	ENST00000256078;ENST00000311936;ENST00000557334;ENST00000556131	missense_variant	p.Gly12Asp/c.35G>A
UPN010	9	139399348	C	CCCT	1232	0	1238	0.00 %	1889	105	1892	5.55 %	NOTCH1	ENST00000277541	inframe_insertion	p.Arg1598dup/c.4794_4795insAGG
UPN010	1	115258744	C	A	947	1	948	0.11 %	1552	55	1611	3.41 %	NRAS	ENST00000369535	missense_variant	p.Gly13Val/c.38G>T
UPN010	9	139397768	A	G	1036	0	1040	0.00 %	1636	51	1692	3.01 %	NOTCH1	ENST00000277541	missense_variant	p.Leu1678Pro/c.5033T>C
UPN010	1	115258747	C	G	919	2	924	0.22 %	1518	39	1563	2.50 %	NRAS	ENST00000369535	missense_variant	p.Gly12Ala/c.35G>C

UPN011	9	139397679	A	G	1424	0	1425	0.00 %	991	938	1931	48.58 %	NOTCH1	ENST00000277541	missense_variant	p.Ser1708Pro/c.5122T>C
UPN011	4	153249384	C	T	1099	0	1101	0.00 %	1045	446	1499	29.75 %	FBXW7	ENST00000281708;ENST00000296555;ENST00000263981;ENST00000603548;ENST00000393956;ENST00000603841	missense_variant	p.Arg465His/c.1394G>A;p.Arg347His/c.1040G>A;p.Arg385His/c.1154G>A;p.Arg289His/c.866G>A
UPN011	1	115256532	C	T	900	0	901	0.00 %	900	319	1225	26.04 %	NRAS	ENST00000369535	missense_variant	p.Gly60Glu/c.179G>A
UPN012	16	8999095	G	A	1322	0	1326	0.00 %	788	666	1457	45.71 %	USP7	ENST00000344836;ENST00000381886;ENST00000535863;ENST00000563085;ENST00000542333	stop_gained	p.Arg508*/c.1522C>T;p.Arg492*/c.1474C>T;p.Arg409*/c.1225C>T;p.Arg450*/c.1348C>T
UPN013	16	9010967	ACG	ACCCA	777	3	780	0.38 %	622	374	1001	37.36 %	USP7	ENST00000344836;ENST00000381886;ENST00000535863;ENST00000563085;ENST00000542333	frameshift_variant+missense_variant	p.Val256fs/c.765delCinsTGG;p.Val240fs/c.717delCinsTGG;p.Val157fs/c.468delCinsTGG;p.Val198fs/c.591delCinsTGG
UPN013	9	139390816	G	A	1237	1	1244	0.08 %	1093	370	1465	25.26 %	NOTCH1	ENST00000277541	stop_gained	p.Gln2459*/c.7375C>T
UPN013	9	139390923	G	C	1526	1	1532	0.07 %	1392	290	1685	17.21 %	NOTCH1	ENST00000277541	stop_gained	p.Ser2423*/c.7268C>G
UPN013	9	139399396	CCGGCGGCATCA	TCT	1441	5	1445	0.35 %	1362	251	1620	15.49 %	NOTCH1	ENST00000277541	missense_variant+disruptive_inframe_deletion	p.Leu1579_Glu1583delinsGlnLys/c.4736_4747delTGATGCCGCCGGinsAGA
UPN013	10	89725118	AGATGTT	AATGTTACCC	887	2	887	0.23 %	839	125	964	12.97 %	PTEN	ENST00000371953	missense_variant+inframe_insertion	p.Asp368_Val369delinsMetLeuPro/c.1102_1107delGATGTTinsATGTTACCC
UPN013	5	67589582	TT	AGGAC	579	4	583	0.69 %	679	46	726	6.34 %	PIK3R1	ENST00000396611;ENST00000521381;ENST00000521657;ENST00000274335;ENST00000320694;ENST00000521409;ENST00000336483;ENST00000519025;ENST00000523872	missense_variant+disruptive_inframe_insertion	p.Leu449delinsArgThr/c.1345_1346delTTinsAGGAC;p.Leu149delinsArgThr/c.445_446delTTinsAGGAC;p.Leu86delinsArgThr/c.256_257delTTinsAGGAC;p.Leu179delinsArgThr/c.535_536delTTinsAGGAC;p.Leu122delinsArgThr/c.364_365delTTinsAGGAC

UPN013	5	67591131	AGAC	A	722	0	724	0.00 %	792	34	828	4.11 %	PIK3R1	ENST00000396611;ENST00000521381;ENST00000521657;ENST00000274335;ENST00000320694;ENST00000336483;ENST00000523872	disruptive_inframe_deletion	p.Thr576del/c.1727_1729delCGA;p.Thr276del/c.827_829delCGA;p.Thr306del/c.917_919delCGA;p.Thr213del/c.638_640delCGA
UPN014	X	133527961	AA	ATTGAAGGCGCC	505	0	507	0.00 %	34	244	287	85.02 %	PHF6	ENST00000394292;ENST00000370803;ENST00000332070;ENST00000370799;ENST00000416404;ENST00000370800	frameshift_variant+missense_variant	p.Lys133fs/c.398delAinsTTGAAGGCGCC;p.Lys99fs/c.296delAinsTTGAAGGCGCC
UPN014	9	139391191	G	GT	1785	0	1786	0.00 %	1489	668	1527	43.75 %	NOTCH1	ENST00000277541	frameshift_variant	p.Pro2334fs/c.6999_7000insA
UPN014	19	10930723	T	TGTCCAAGGCG	1547	0	1551	0.00 %	1174	373	1176	31.72 %	DNM2	ENST00000314646;ENST00000585892;ENST00000359692;ENST00000389253;ENST00000355667;ENST00000408974	frameshift_variant	p.Asn582fs/c.1745_1746insGGCGGTCCAA;p.Asn578fs/c.1733_1734insGGCGGTCCAA
UPN014	17	30264386	T	TCCGGC	228	0	229	0.00 %	325	101	327	30.89 %	SUZ12	ENST00000322652;ENST00000580398	frameshift_variant	p.Gly43fs/c.122_126dupCCGGC
UPN015	1	65312365	A	G	1280	1	1281	0.08 %	1359	84	1499	5.60 %	JAK1	ENST00000342505	missense_variant	p.Tyr652His/c.1954T>C
UPN015	1	65312365	A	C	1280	0	1281	0.00 %	1359	55	1499	3.67 %	JAK1	ENST00000342505	missense_variant	p.Tyr652Asp/c.1954T>G
UPN015	19	17949108	C	T	1215	0	1218	0.00 %	1384	41	1429	2.87 %	JAK3	ENST00000458235;ENST00000527670;ENST00000534444	missense_variant	p.Met511Ile/c.1533G>A
UPN016	X	133559248	A	G	455	0	457	0.00 %	355	122	478	25.52 %	PHF6	ENST00000394292;ENST00000370803;ENST00000332070;ENST00000416404	missense_variant	p.His330Arg/c.989A>G;p.His329Arg/c.986A>G;p.His295Arg/c.884A>G
UPN016	1	115258748	C	T	1377	0	1378	0.00 %	1273	200	1477	13.54 %	NRAS	ENST00000369535	missense_variant	p.Gly12Ser/c.34G>A
UPN016	9	139390648	CAG	C	1492	0	1500	0.00 %	1193	142	1343	10.57 %	NOTCH1	ENST00000277541	frameshift_variant	p.Pro2514fs/c.7541_7542delCT
UPN016	4	153247366	C	T	995	0	997	0.00 %	1033	113	1147	9.85 %	FBXW7	ENST00000281708;ENST00000296555;ENST00000263981;ENST00000603548;ENST00000393956;ENST00000603841	missense_variant	p.Arg479Gln/c.1436G>A;p.Arg361Gln/c.1082G>A;p.Arg399Gln/c.1196G>A;p.Arg303Gln/c.908G>A

UPN016	9	139397768	A	G	1569	0	1571	0.00 %	1500	159	1669	9.53 %	NOTCH1	ENST00000277541	missense_variant	p.Leu1678Pro/c.5033T>C
UPN016	17	30267504	A	T	664	0	666	0.00 %	816	65	884	7.35 %	SUZ12	ENST00000322652;ENST00000580398	missense_variant	p.Arg129Trp/c.385A>T;p.Ser129Cys/c.385A>T
UPN017	X	133547981	CCATTAT	CGAGGAAGAA	341	2	342	0.58 %	71	231	301	76.74 %	PHF6	ENST00000394292;ENST00000370803;ENST00000332070;ENST00000370799;ENST00000416404;ENST00000370800	missense_variant+inframe_insertion	p.His240_Tyr241delinsGluGluGlu/c.718_723delCATTATinsGAGGAAGAA;p.His239_Tyr240delinsGluGluGlu/c.715_720delCATTATinsGAGGAAGAA;p.His205_Tyr206delinsGluGluGlu/c.613_618delCATTATinsGAGGAAGAA
UPN017	19	10906072	C	T	1569	0	1571	0.00 %	1477	298	1778	16.76 %	DNM2	ENST00000314646;ENST00000585892;ENST00000359692;ENST00000389253;ENST00000355667;ENST00000408974;ENST00000587830	stop_gained	p.Arg385*/c.1153C>T;p.Arg137*/c.409C>T
UPN017	9	139390863	AC	AGGCCAGATTTTA	1576	4	1578	0.25 %	1390	215	1613	13.33 %	NOTCH1	ENST00000277541	frameshift_variant+stop_gained+missense_vari	p.Val2443fs/c.7327delGinsTAAAATCTGGCC
UPN017	9	139399365	A	G	1766	0	1770	0.00 %	1647	239	1889	12.65 %	NOTCH1	ENST00000277541	missense_variant	p.Leu1593Pro/c.4778T>C
UPN017	9	139390887	CTCAGGAAGCT	CGGTGCTAAACCTGATAGA	1590	8	1593	0.50 %	1322	194	1556	12.47 %	NOTCH1	ENST00000277541	frameshift_variant+stop_gained	p.Phe2433fs/c.7294_7303delAGCTTCCTGAinsTCTATCAGGTTTAGCACC
UPN017	9	139399325	G	GCTAAAA	1824	0	1825	0.00 %	1925	221	1925	11.48 %	NOTCH1	ENST00000277541	disruptive_inframe_insertion	p.Phe1606_Lys1607insPheSer/c.4817_4818insTTTTAG
UPN017	9	139399350	C	G	1817	1	1819	0.05 %	1788	99	1892	5.23 %	NOTCH1	ENST00000277541	missense_variant	p.Arg1598Pro/c.4793G>C
UPN018	9	139399365	A	G	2019	1	2023	0.05 %	925	515	1444	35.66 %	NOTCH1	ENST00000277541	missense_variant	p.Leu1593Pro/c.4778T>C
UPN019	X	133551305	T	C	792	1	794	0.13 %	43	399	442	90.27 %	PHF6	ENST00000394292;ENST00000370803;ENST00000332070;ENST00000370799;ENST00000416404	missense_variant	p.Ile315Thr/c.944T>C;p.Ile314Thr/c.941T>C;p.Ile280Thr/c.839T>C
UPN019	9	139399422	A	G	2489	1	2491	0.04 %	988	540	1529	35.32 %	NOTCH1	ENST00000277541	missense_variant	p.Leu1574Pro/c.4721T>C
UPN019	19	10940819	C	T	1475	0	1480	0.00 %	521	258	781	33.03 %	DNM2	ENST00000314646;ENST00000585892;ENST00000359692;ENST00000389253;ENST00000355667;ENST00000408974;ENST00000589106	stop_gained	p.Arg770*/c.2308C>T;p.Arg766*/c.2296C>T;p.Arg58*/c.172C>T

UPN019	4	153247366	C	T	1513	0	1515	0.00 %	665	302	969	31.17 %	FBXW7	ENST00000281708;ENST00000296555;ENST00000263981;ENST00000603548;ENST00000393956;ENST00000603841	missense_variant	p.Arg479Gln/c.1436G>A;p.Arg361Gln/c.1082G>A;p.Arg399Gln/c.1196G>A;p.Arg303Gln/c.908G>A
UPN019	14	99641758	C	T	1259	0	1262	0.00 %	694	313	1008	31.05 %	BCL11B	ENST00000357195;ENST00000345514;ENST00000443726	missense_variant	p.Arg472His/c.1415G>A;p.Arg401His/c.1202G>A;p.Arg278His/c.833G>A
UPN020	4	153244092	G	A	1226	0	1231	0.00 %	121	1201	1327	90.50 %	FBXW7	ENST00000281708;ENST00000296555;ENST00000263981;ENST00000603548;ENST00000393956;ENST00000603841	missense_variant	p.Arg689Trp/c.2065C>T;p.Arg571Trp/c.1711C>T;p.Arg609Trp/c.1825C>T;p.Arg513Trp/c.1537C>T
UPN020	14	99640631	G	A	1397	0	1399	0.00 %	752	704	1458	48.29 %	BCL11B	ENST00000357195;ENST00000345514;ENST00000443726	stop_gained	p.Gln848*/c.2542C>T;p.Gln777*/c.2329C>T;p.Gln654*/c.1960C>T
UPN020	9	139397768	A	G	1438	1	1444	0.07 %	897	704	1603	43.92 %	NOTCH1	ENST00000277541	missense_variant	p.Leu1678Pro/c.5033T>C
UPN020	X	133511785	G	GA	754	0	755	0.00 %	1005	428	1014	42.21 %	PHF6	ENST00000394292;ENST00000370803;ENST00000332070;ENST00000370799;ENST00000416404;ENST00000370800	splice_donor_variant+intron_variant	c.138_138+1insA
UPN020	6	41903793	A	AGGCT	1618	0	1623	0.00 %	1591	637	1594	39.96 %	CCND3	ENST00000372991;ENST00000511642;ENST00000372987;ENST00000415497;ENST00000372988;ENST00000414200;ENST00000510503	frameshift_variant	p.Leu255fs/c.760_763dupAGCC;p.Leu174fs/c.517_520dupAGCC;p.Leu205fs/c.610_613dupAGCC;p.Leu59fs/c.172_175dupAGCC;p.Leu183fs/c.544_547dupAGCC;p.Gln129fs/c.380_383dupAGCC
UPN020	16	8999170	CG	CAGGTAGGCCCGTATA	1105	0	1101	0.00 %	655	271	934	29.01 %	USP7	ENST00000344836;ENST00000381886;ENST00000535863;ENST00000563085;ENST00000542333	frameshift_variant+synonymous_variant	p.Asp483fs/c.1446delCinsTATACGGGCCTACCT;p.Asp467fs/c.1398delCinsTATACGGGCCTACCT;p.Asp384fs/c.1149delCinsTATACGGGCCTACCT;p.Asp425fs/c.1272delCinsTATACGGGCCTACCT

UPN021	6	135509033	A	T	1106	2	1110	0.18 %	733	190	923	20.59 %	MYB	ENST00000341911;ENST00000339290;ENST00000367812;ENST00000463282;ENST00000525477;ENST00000533837;ENST00000316528;ENST00000442647;ENST00000367814;ENST00000527615;ENST00000525369;ENST00000525002;ENST00000525940;ENST00000526187;ENST00000526565;ENST00000528015;ENST00000528140;ENST00000528774;ENST00000529262;ENST00000531634;ENST00000531737;ENST00000533384;ENST00000533624;ENST00000534044;ENST00000534121;ENST00000528343;ENST00000438901;ENST00000525514;E[illegible]	missense_variant	p.Asn68Ile/c.203A>T;p.Asn22Ile/c.65A>T
UPN021	17	30274658	A	T	910	1	913	0.11 %	656	32	689	4.64 %	SUZ12	ENST00000322652	missense_variant	p.Met137Leu/c.409A>T
UPN021	5	35874599	TTCTCTGTC	CCCTGGGAA	1501	0	1501	0.00 %	1170	31	1232	2.52 %	IL7R	ENST00000303115	missense_variant	p.SerValAlaLeu252SerLeuGlyMet
UPN021	5	35874599	TTCTCTGTC	CCCGGGGAG	1501	0	1501	0.00 %	1170	26	1232	2.11 %	IL7R	ENST00000303115	missense_variant	p.SerValAlaLeu252SerArgGlyVal
UPN022	X	133547902	G	A	456	0	456	0.00 %	91	210	303	69.31 %	PHF6	ENST00000394292;ENST00000370803;ENST00000332070;ENST00000370799;ENST00000416404;ENST00000370800	missense_variant	p.Cys213Tyr/c.638G>A;p.Cys212Tyr/c.635G>A;p.Cys178Tyr/c.533G>A

UPN022	12	122265868	T	G	1970	3	1975	0.15 %	783	394	1180	33.39 %	SETD1B	ENST00000604567;ENST00000542440;ENST00000267197	stop_gained	p.Tyr1873*/c.5619T>G;p.Tyr1830*/c.5490T>G
UPN022	9	139397768	A	G	1825	1	1828	0.05 %	649	313	966	32.40 %	NOTCH1	ENST00000277541	missense_variant	p.Leu1678Pro/c.5033T>C
UPN022	17	30300238	C	CA	678	0	682	0.00 %	444	108	445	24.27 %	SUZ12	ENST00000322652;ENST00000580398	frameshift_variant	p.Arg196fs/c.586dupA;p.Arg173fs/c.517dupA
UPN023	19	54659071	G	A	3110	0	3120	0.00 %	875	686	1568	43.75 %	CNOT3	ENST00000221232;ENST00000406403;ENST00000471126	missense_variant	p.Glu730Lys/c.2188G>A;p.Glu65Lys/c.193G>A
UPN023	19	54647477	A	T	2372	4	2381	0.17 %	625	475	1102	43.10 %	CNOT3	ENST00000221232;ENST00000406403;ENST00000440571;ENST00000447684	missense_variant	p.Ile84Phe/c.250A>T;p.Ile4Phe/c.10A>T;p.Ile2Phe/c.4A>T
UPN023	9	139390846	TG	T	3219	2	3232	0.06 %	823	622	1449	42.93 %	NOTCH1	ENST00000277541	frameshift_variant	p.Ser2449fs/c.7344delC
UPN023	4	153244266	G	A	1945	0	1947	0.00 %	742	539	1285	41.95 %	FBXW7	ENST00000281708;ENST00000296555;ENST00000263981;ENST00000603548;ENST00000393956;ENST00000603841	stop_gained	p.Gln631*/c.1891C>T;p.Gln513*/c.1537C>T;p.Gln551*/c.1651C>T;p.Gln455*/c.1363C>T
UPN023	7	50455090	C	T	2207	0	2212	0.00 %	706	493	1203	40.98 %	IKZF1	ENST00000331340;ENST00000343574;ENST00000438033	stop_gained	p.Arg213*/c.637C>T;p.Arg126*/c.376C>T
UPN023	9	139399367	G	GTGT	3708	0	3718	0.00 %	1471	571	1477	38.66 %	NOTCH1	ENST00000277541	disruptive_inframe_insertion	p.Phe1592delinsLeuHis/c.4775_4776insACA
UPN024	17	40353823	G	A	1091	0	1092	0.00 %	603	506	1110	45.59 %	STAT5B	ENST00000293328	missense_variant	p.Ala766Val/c.2297C>T
UPN024	X	41202045	C	T	1020	0	1023	0.00 %	955	412	1369	30.09 %	DDX3X	ENST00000399959;ENST00000457138;ENST00000542215	missense_variant	p.Pro167Ser/c.499C>T;p.Pro151Ser/c.451C>T;p.Pro211Ser/c.631C>T

UPN024	6	41903730	TGGCTGCTGGAGCCCCGGGGGGCTTTGGGCGCTG	T	1405	0	1407	0.00 %	1114	311	1426	21.81 %	CCND3	ENST00000372991;ENST00000511642;ENST00000372987;ENST00000415497;ENST00000372988;ENST00000414200;ENST00000510503	disruptive_inframe_deletion	p.Pro265_Ser275del/c.794_826delCAGCGCCCAAAGCCCCCCGGGGCTCCAGCAGCC;p.Pro184_Ser194del/c.551_583delCAGCGCCCAAAGCCCCCCGGGGCTCCAGCAGCC;p.Pro215_Ser225del/c.644_676delCAGCGCCCAAAGCCCCCCGGGGCTCCAGCAGCC;p.Pro69_Ser79del/c.206_238delCAGCGCCCAAAGCCCCCCGGGGCTCCAGCAGCC;p.Pro193_Ser203del/c.578_610delCAGCGCCCAAAGCCCCCCGGGGCTCCAGCAGCC;p.Ser139_Pro149del/c.414_446delCAGCGCCCAAAGCCCCCCGGGGCTCCAGCAGCC
UPN025	19	10930662	GAGA	G	1076	0	1076	0.00 %	880	1237	2119	58.38 %	DNM2	ENST00000314646;ENST00000585892;ENST00000359692;ENST00000389253;ENST00000355667;ENST00000408974	inframe_deletion	p.Lys562del/c.1684_1686delAAG;p.Lys558del/c.1672_1674delAAG
UPN025	14	99641824	G	A	861	0	862	0.00 %	827	670	1499	44.70 %	BCL11B	ENST00000357195;ENST00000345514;ENST00000443726	missense_variant	p.Thr450Met/c.1349C>T;p.Thr379Met/c.1136C>T;p.Thr256Met/c.767C>T
UPN025	9	139390849	GG	TGAT	1110	11	1117	0.98 %	1102	880	1990	44.22 %	NOTCH1	ENST00000277541	frameshift_variant+missense_variant	p.Pro2448fs/c.7341_7342delCCinsATCA
UPN025	7	148512087	G	A	916	0	917	0.00 %	914	718	1637	43.86 %	EZH2	ENST00000320356;ENST00000460911;ENST00000350995;ENST00000483967	missense_variant	p.Pro531Ser/c.1591C>T;p.Pro526Ser/c.1576C>T;p.Pro487Ser/c.1459C>T;p.Pro517Ser/c.1549C>T
UPN025	9	139397639	ACGGCCT	A	1004	0	1004	0.00 %	971	675	1649	40.93 %	NOTCH1	ENST00000277541	disruptive_inframe_deletion	p.Glu1719_Ala1720del/c.5156_5161delAGGCCG
UPN025	1	65312365	A	T	1049	1	1052	0.10 %	1794	78	1874	4.16 %	JAK1	ENST00000342505	missense_variant	p.Tyr652Asn/c.1954T>A
UPN026	9	139399296	A	T	1448	0	1450	0.00 %	970	758	1732	43.76 %	NOTCH1	ENST00000277541	missense_variant	p.Ile1616Asn/c.4847T>A

UPN026	X	133549083	C	G	898	0	901	0.00 %	656	474	1133	41.84 %	PHF6	ENST00000394292;E<br>NST00000370803;E<br>NST00000332070;E<br>NST00000370799;E<br>NST00000416404;E<br>NST00000370800	stop_gained	p.Ser257*/c.770C>G;p.Ser256<br>*/c.767C>G;p.Ser222*/c.665C<br>>G
UPN026	6	135515009	C	T	1069	0	1071	0.00 %	849	605	1462	41.38 %	MYB	ENST00000341911;E<br>NST00000339290;E<br>NST00000367812;E<br>NST00000463282;E<br>NST00000525477;E<br>NST00000533837;E<br>NST00000316528;E<br>NST00000442647;E<br>NST00000367814;E<br>NST00000527615;E<br>NST00000420123;E<br>NST00000525369;E<br>NST00000525002;E<br>NST00000525940;E<br>NST00000526187;E<br>NST00000526565;E<br>NST00000528015;E<br>NST00000528140;E<br>NST00000528345;E<br>NST00000528774;E<br>NST00000529262;E<br>NST00000531737;E<br>NST00000533384;E<br>NST00000533624;E<br>NST00000534044;E<br>NST00000534121;E<br>NST00000528343;E<br>NST00000438901;E<br>[illegible]	missense_variant	p.His266Tyr/c.796C>T;p.His24<br>2Tyr/c.724C>T;p.His220Tyr/c.<br>658C>T
UPN026	4	153249384	C	G	1272	3	1276	0.24 %	942	665	1613	41.23 %	FBXW7	ENST00000281708;E<br>NST00000296555;E<br>NST00000263981;E<br>NST00000603548;E<br>NST00000393956;E<br>NST00000603841	missense_variant	p.Arg465Pro/c.1394G>C;p.Arg<br>347Pro/c.1040G>C;p.Arg385Pr<br>o/c.1154G>C;p.Arg289Pro/c.8<br>66G>C

UPN026	19	10908073	C	A	1272	0	1273	0.00 %	948	644	1597	40.33 %	DNM2	ENST00000314646;ENST00000389253;ENST00000408974;ENST00000587830	missense_variant	p.Pro405His/c.1214C>A;p.Pro157His/c.470C>A
UPN026	9	139390778	CGAGGAT	CGGAGAGGCGAC	1355	10	1359	0.74 %	883	431	1309	32.93 %	NOTCH1	ENST00000277541	frameshift_variant+missense_variant	p.Ser2471fs/c.7407_7411delATCCTinsGTCGCCTCTC
UPN026	21	36252868	C	CCAGGGA	1017	0	1020	0.00 %	1237	386	1273	30.32 %	RUNX1	ENST00000300305;ENST00000344691;ENST00000325074;ENST00000437180;ENST00000399240;ENST00000358356;ENST00000399237;ENST00000486278	inframe_insertion	p.Val164_Gly165insValPro/c.493_494insTCCCTG;p.Val137_Gly138insValPro/c.412_413insTCCCTG;p.Val152_Gly153insValPro/c.457_458insTCCCTG;p.Val140_Gly141insValPro/c.421_422insTCCCTG
UPN027	4	153249456	C	T	1047	1	1049	0.10 %	716	558	1279	43.63 %	FBXW7	ENST00000281708;ENST00000296555;ENST00000263981;ENST00000603548;ENST00000393956;ENST00000603841	missense_variant	p.Arg441Gln/c.1322G>A;p.Arg323Gln/c.968G>A;p.Arg361Gln/c.1082G>A;p.Arg265Gln/c.794G>A
UPN027	1	115252203	G	A	1007	0	1007	0.00 %	661	496	1158	42.83 %	NRAS	ENST00000369535	missense_variant	p.Ala146Val/c.437C>T
UPN027	4	153247366	C	T	1007	1	1010	0.10 %	595	443	1039	42.64 %	FBXW7	ENST00000281708;ENST00000296555;ENST00000263981;ENST00000603548;ENST00000393956;ENST00000603841	missense_variant	p.Arg479Gln/c.1436G>A;p.Arg361Gln/c.1082G>A;p.Arg399Gln/c.1196G>A;p.Arg303Gln/c.908G>A
UPN027	9	139399395	T	TCACACA	1495	0	1500	0.00 %	1681	654	1688	38.74 %	NOTCH1	ENST00000277541	inframe_insertion	p.Pro1582_Glu1583insValCys/c.4747_4748insTGTGTG
UPN027	X	133527956	ACAAGAAAAC	GGAAAGGGGAGG	815	6	821	0.73 %	588	201	814	24.69 %	PHF6	ENST00000394292;ENST00000370803;ENST00000332070;ENST00000370799;ENST00000416404;ENST00000370800	frameshift_variant+missense_variant	p.His131fs/c.392_401delACAAGAAAACinsGGAAAGGGGAGG;p.His97fs/c.290_299delACAAGAAAACinsGGAAAGGGGAGG
UPN028	1	65311203	C	A	1398	1	1403	0.07 %	111	1070	1185	90.30 %	JAK1	ENST00000342505	missense_variant	p.Ser703Ile/c.2108G>T
UPN028	21	36164720	G	T	1691	0	1693	0.00 %	992	808	1803	44.81 %	RUNX1	ENST00000300305;ENST00000344691;ENST00000325074;ENST00000437180;ENST00000399240	stop_gained	p.Tyr385*/c.1155C>A;p.Tyr358*/c.1074C>A;p.Tyr373*/c.1119C>A;p.Tyr294*/c.882C>A

UPN028	16	9017172	G	GATCCT	1563	0	1565	0.00 %	1344	487	1344	36.24 %	USP7	ENST00000344836;ENST00000381886;ENST00000542333;ENST00000566004;ENST00000569230;ENST00000566273;ENST00000564117	frameshift_variant	p.Leu95fs/c.282_283insAGGAT;p.Leu79fs/c.234_235insAGGAT;p.Leu37fs/c.108_109insAGGAT;p.Leu81fs/c.240_241insAGGAT
UPN028	9	139399294	A	ATTGGGGGTTCTG	1559	0	1574	0.00 %	1477	499	1503	33.20 %	NOTCH1	ENST00000277541	inframe_insertion	p.Ile1616_Phe1617insGlnAsnProGln/c.4848_4849insCAGAACCCCCAA
UPN029	9	139397768	A	G	1214	1	1216	0.08 %	1232	320	1559	20.53 %	NOTCH1	ENST00000277541	missense_variant	p.Leu1678Pro/c.5033T>C
UPN029	4	153244092	G	A	1074	0	1076	0.00 %	1139	248	1390	17.84 %	FBXW7	ENST00000281708;ENST00000296555;ENST00000263981;ENST00000603548;ENST00000393956;ENST00000603841	missense_variant	p.Arg689Trp/c.2065C>T;p.Arg571Trp/c.1711C>T;p.Arg609Trp/c.1825C>T;p.Arg513Trp/c.1537C>T
UPN029	19	11144101	G	C	1239	0	1242	0.00 %	1340	278	1621	17.15 %	SMARCA4	ENST00000358026;ENST00000344626;ENST00000429416;ENST00000541122;ENST00000589677;ENST00000444061;ENST00000590574;ENST00000413806;ENST00000450717	missense_variant	p.Val1228Leu/c.3682G>C
UPN030	17	7578271	T	A	1296	0	1298	0.00 %	316	1957	2280	85.83 %	TP53	ENST00000269305;ENST00000413465;ENST00000359597;ENST00000420246;ENST00000455263;ENST00000445888;ENST00000509690;ENST00000514944	missense_variant	p.His193Leu/c.578A>T;p.His61Leu/c.182A>T;p.His100Leu/c.299A>T
UPN030	7	148507475	C	A	913	0	915	0.00 %	477	475	952	49.89 %	EZH2	ENST00000320356;ENST00000478654;ENST00000460911;ENST00000350995;ENST00000541220;ENST00000476773;ENST00000483967	missense_variant	p.Gly660Val/c.1979G>T;p.Gly604Val/c.1811G>T;p.Gly655Val/c.1964G>T;p.Gly616Val/c.1847G>T;p.Gly646Val/c.1937G>T

UPN030	X	133527636	C	T	551	1	552	0.18 %	285	242	530	45.66 %	PHF6	ENST00000394292;ENST00000370803;ENST00000332070;ENST00000370799;ENST00000416404;ENST00000370800	stop_gained	p.Arg116*/c.346C>T;p.Arg82*/c.244C>T
UPN030	9	139397702	G	T	1406	2	1409	0.14 %	2157	1328	3492	38.03 %	NOTCH1	ENST00000277541	missense_variant	p.Ala1700Asp/c.5099C>A
UPN030	X	44942755	TCG	TCCC	604	5	605	0.83 %	583	259	845	30.65 %	KDM6A	ENST00000382899;ENST00000377967;ENST00000536777;ENST00000543216;ENST00000414389;ENST00000433797	frameshift_variant+missense_variant	p.Val1120fs/c.3358delGinsCC;p.Val1113fs/c.3337delGinsCC;p.Val1068fs/c.3202delGinsCC;p.Val1034fs/c.3100delGinsCC;p.Val710fs/c.2128delGinsCC;p.Val755fs/c.2263delGinsCC
UPN031	X	133547658	G	T	656	0	657	0.00 %	727	306	1036	29.54 %	PHF6	ENST00000394292;ENST00000370803;ENST00000332070;ENST00000370799;ENST00000416404;ENST00000370800	stop_gained	p.Gly187*/c.559G>T;p.Gly186*/c.556G>T;p.Gly152*/c.454G>T
UPN031	9	139399324	T	TGGGATC	1407	0	1411	0.00 %	1789	418	1799	23.24 %	NOTCH1	ENST00000277541	inframe_insertion	p.Phe1606_Lys1607insAspPro/c.4818_4819insGATCCC
UPN031	5	35874570	A	ACTAAGGCCCCGTGAGGTTAAGTGC	979	0	982	0.00 %	1254	167	1256	13.30 %	IL7R	ENST00000303115	disruptive_inframe_insertion	p.Leu243_Thr244insArgProArgGluValLysCysLeu/c.730_731insGGCCCCGTGAGGTTAAGTGCCTAA
UPN031	17	7577539	G	A	1241	0	1243	0.00 %	1568	151	1725	8.75 %	TP53	ENST00000269305;ENST00000413465;ENST00000359597;ENST00000420246;ENST00000455263;ENST00000445888;ENST00000509690;ENST00000514944	missense_variant	p.Arg248Trp/c.742C>T;p.Arg116Trp/c.346C>T;p.Arg155Trp/c.463C>T
UPN032	9	139390732	G	A	1322	0	1323	0.00 %	1701	325	2036	15.96 %	NOTCH1	ENST00000277541	stop_gained	p.Gln2487*/c.7459C>T
UPN032	9	139399331	C	CACTGAT	1501	0	1504	0.00 %	2114	297	2122	14.00 %	NOTCH1	ENST00000277541	disruptive_inframe_insertion	p.Val1604_Val1605insSerVal/c.4811_4812insATCAGT
UPN032	1	216595196	T	G	609	2	612	0.33 %	807	124	932	13.30 %	USH2A	ENST00000366943;ENST00000307340;ENST00000366942	splice_region_variant+synonymous_variant	p.Val161Val/c.483A>C

UPN033	9	139390649	A	AT	1127	0	1137	0.00 %	1231	546	1241	44.00 %	NOTCH1	ENST00000277541	frameshift_variant	p.Glu2515fs/c.7541_7542insA
UPN033	9	139399410	A	C	1220	0	1221	0.00 %	1039	434	1476	29.40 %	NOTCH1	ENST00000277541	missense_variant	p.Val1578Gly/c.4733T>G
UPN033	9	139399365	A	G	1256	1	1257	0.08 %	1236	229	1472	15.56 %	NOTCH1	ENST00000277541	missense_variant	p.Leu1593Pro/c.4778T>C
UPN034	17	40359729	T	G	746	0	747	0.00 %	442	404	850	47.53 %	STAT5B	ENST00000293328	missense_variant	p.Asn642His/c.1924A>C
UPN034	14	99641758	C	T	680	0	684	0.00 %	521	468	992	47.18 %	BCL11B	ENST00000357195;ENST00000345514;ENST00000443726	missense_variant	p.Arg472His/c.1415G>A;p.Arg401His/c.1202G>A;p.Arg278His/c.833G>A
UPN034	9	139390978	G	A	1425	0	1428	0.00 %	1009	756	1768	42.76 %	NOTCH1	ENST00000277541	stop_gained	p.Gln2405*/c.7213C>T
UPN034	16	9010942	G	GGACCT	753	0	766	0.00 %	1058	371	1071	34.64 %	USP7	ENST00000344836;ENST00000381886;ENST00000535863;ENST00000563085;ENST00000542333	frameshift_variant	p.Phe264fs/c.791_792insAGGTC;p.Phe248fs/c.743_744insAGGTC;p.Phe165fs/c.494_495insAGGTC;p.Phe206fs/c.617_618insAGGTC
UPN034	9	139399353	C	A	1357	1	1360	0.07 %	1616	178	1794	9.92 %	NOTCH1	ENST00000277541	missense_variant	p.Ser1597Ile/c.4790G>T
UPN034	9	139397727	A	G	1295	0	1298	0.00 %	1447	134	1584	8.46 %	NOTCH1	ENST00000277541	missense_variant	p.Cys1692Arg/c.5074T>C
UPN035	10	89717659	TTCAGGACCCACA	GGGATCCCTAATTCT	959	7	963	0.73 %	263	174	443	39.28 %	PTEN	ENST00000371953	frameshift_variant+missense_variant	p.Asn228fs/c.684_696delTTCAGGACCCACAinsGGGATCCCTAATTCT
UPN036	2	16082317	C	T	1773	0	1775	0.00 %	1096	402	1500	26.80 %	MYCN	ENST00000281043	missense_variant	p.Pro44Leu/c.131C>T
UPN037	16	9017114	C	CT	1177	0	1179	0.00 %	1372	584	1373	42.53 %	USP7	ENST00000344836;ENST00000381886;ENST00000535863;ENST00000563085;ENST00000542333;ENST00000566004;ENST00000569230;ENST00000566273;ENST00000564117	frameshift_variant	p.Ser114fs/c.340dupA;p.Ser98fs/c.292dupA;p.Ser15fs/c.43dupA;p.Ser56fs/c.166dupA;p.Ser100fs/c.298dupA
UPN037	4	153249385	G	A	1593	1	1598	0.06 %	983	672	1660	40.48 %	FBXW7	ENST00000281708;ENST00000296555;ENST00000263981;ENST00000603548;ENST00000393956;ENST00000603841	missense_variant	p.Arg465Cys/c.1393C>T;p.Arg347Cys/c.1039C>T;p.Arg385Cys/c.1153C>T;p.Arg289Cys/c.865C>T
UPN038_p	4	153249385	G	A	870	0	874	0.00 %	727	632	1359	46.50 %	FBXW7	ENST00000281708	missense_variant	p.Arg465Cys/c.1393C>T
UPN038_p	4	153332919	G	A	951	1	952	0.11 %	738	481	1227	39.20 %	FBXW7	ENST00000281708	stop_gained	p.Arg13*/c.37C>T
UPN038_p	6	135523774	A	G	0	37	39	94.87 %	3	88	92	95.65 %	MYB	ENST00000367812	missense_variant	p.Arg641Gly/c.1921A>G

UPN038_p	9	139399404	AT	GG	885	3	904	0.33 %	935	603	1561	38.63 %	NOTCH1	ENST00000277541	missense_variant	p.Met1580Pro/c.4738_4739delATinsCC
UPN039_p	10	89717672	C	CGGCGG	1154	0	1156	0.00 %	1280	408	1281	31.85 %	PTEN	ENST00000371953	frameshift_variant	p.Glu235fs/c.698_699insGCGGG
UPN040_p	1	65310518	G	A	738	0	740	0.00 %	560	243	806	30.15 %	JAK1	ENST00000342505	missense_variant	p.Arg724Cys/c.2170C>T
UPN040_p	17	40359729	T	G	934	1	939	0.11 %	248	636	886	71.78 %	STAT5B	ENST00000293328	missense_variant	p.Asn642His/c.1924A>C
UPN040_p	19	17945969	C	T	1214	1	1217	0.08 %	776	422	1201	35.14 %	JAK3	ENST00000458235	missense_variant	p.Arg657Gln/c.1970G>A
UPN040_p	19	54649413	G	A	1357	6	1367	0.44 %	838	415	1254	33.09 %	CNOT3	ENST00000221232	missense_variant	p.Arg188His/c.563G>A
UPN040_p	1	65309790	C	A	777	0	777	0.00 %	958	12	970	1.24 %	JAK1	ENST00000342505	missense_variant	p.Cys787Phe/c.2360G>T
UPN041_p	9	139390648	C	A	825	6	837	0.72 %	504	480	989	48.53 %	NOTCH1	ENST00000277541	stop_gained	p.Glu2515*/c.7543G>T
UPN041_p	9	139397702	G	T	873	0	876	0.00 %	658	527	1189	44.32 %	NOTCH1	ENST00000277541	missense_variant	p.Ala1700Asp/c.5099C>A
UPN041_p	19	10904493	C	T	622	2	625	0.32 %	412	390	806	48.39 %	DNM2	ENST00000314646	missense_variant	p.Arg364Cys/c.1090C>T
UPN041_p	19	11135027	C	A	823	3	827	0.36 %	540	446	989	45.10 %	SMARCA4	ENST00000358026	stop_gained	p.Cys998*/c.2994C>A

Supplemental Data 1

					Germline				Tumor							
ID	Chr	Pos	Ref	Alt	#REF	#ALT	DP	VAF	#REF	#ALT	DP	VAF	Gene	ENST	Type	Variant
UPN038_r	4	153249385	G	A	870	0	874	0.00 %	593	412	1005	41.00 %	FBXW7	ENST00000281708	missense_variant	p.Arg465Cys/c.1393C>T
UPN038_r	6	135523774	A	G	0	37	39	94.87 %	2	52	54	96.30 %	MYB	ENST00000367812	missense_variant	p.Arg641Gly/c.1921A>G
UPN038_r	9	139399330	C	CGGGATA	962	0	967	0.00 %	1246	375	1252	29.95 %	NOTCH1	ENST00000277541	inframe_insertion	p.Val1604_Val1605insTyrPro/c.4812_4813insTATC
UPN038_r	19	11132428	G	A	945	2	947	0.21 %	706	426	1133	37.60 %	SMARCA4	ENST00000358026	missense_variant	p.Glu882Lys/c.2644G>A
UPN039_r	X	70348506	G	C	815	1	819	0.12 %	695	17	715	2.38 %	MED12	ENST00000333646	missense_variant	p.Arg1138Pro/c.3413G>C
UPN039_r	10	89717672	C	CGGCGG	1154	0	1156	0.00 %	1291	83	1293	6.42 %	PTEN	ENST00000371953	frameshift_variant	p.Glu235fs/c.698_699ins
UPN040_r	1	65310518	G	A	738	0	740	0.00 %	848	144	994	14.49 %	JAK1	ENST00000342505	missense_variant	p.Arg724Cys/c.2170C>T
UPN040_r	17	40359729	T	G	934	1	939	0.11 %	549	248	799	31.04 %	STAT5B	ENST00000293328	missense_variant	p.Asn642His/c.1924A>C
UPN040_r	19	17945969	C	T	1214	1	1217	0.08 %	1014	132	1148	11.50 %	JAK3	ENST00000458235	missense_variant	p.Arg657Gln/c.1970G>A
UPN040_r	19	54649413	G	A	1357	6	1367	0.44 %	1131	206	1343	15.34 %	CNOT3	ENST00000221232	missense_variant	p.Arg188His/c.563G>A
UPN041_r	9	139390648	C	A	825	6	837	0.72 %	485	412	905	45.52 %	NOTCH1	ENST00000277541	stop_gained	p.Glu2515*/c.7543G>T
UPN041_r	9	139397702	G	T	873	0	876	0.00 %	904	28	933	3.00 %	NOTCH1	ENST00000277541	missense_variant	p.Ala1700Asp/c.5099C>A
UPN041_r	9	139399365	A	G	826	3	840	0.36 %	594	400	1002	39.92 %	NOTCH1	ENST00000277541	missense_variant	p.Leu1593Pro/c.4778T>C
UPN041_r	10	104852955	C	T	714	0	715	0.00 %	621	158	782	20.20 %	NT5C2	ENST00000343289	missense_variant	p.Arg367Gln/c.1100G>A
UPN041_r	19	10904493	C	T	622	2	625	0.32 %	632	22	657	3.35 %	DNM2	ENST00000314646	missense_variant	p.Arg364Cys/c.1090C>T
UPN041_r	19	11135027	C	A	823	3	827	0.36 %	886	20	909	2.20 %	SMARCA4	ENST00000358026	stop_gained	p.Cys998*/c.2994C>A
UPN041_r	3	178937755	T	C	595	32	629	5.09 %	746	48	796	6.03 %	PIK3CA	ENST00000263967	missense_variant	p.Tyr644His/c.1930T>C
UPN041_r	17	71197777	A	T	828	0	829	0.00 %	964	18	983	1.83 %	COG1	ENST00000299886	missense_variant	p.Asn604Ile/c.1811A>T
UPN041_r	17	7577538	C	T	843	0	843	0.00 %	790	15	807	1.86 %	TP53	ENST00000269305	missense_variant	p.Arg248Gln/c.743G>A

T-ALL adult_Relapse

ID	Chr	Pos	Ref	Alt	Germline #REF	Germline #ALT	Germline DP	Germline VAF	Tumor #REF	Tumor #ALT	Tumor DP	Tumor VAF	Gene	ENST	Type	Variant
UPN042	X	41201855	C	G	804	0	806	0.00 %	288	506	795	63.65 %	DDX3X	ENST00000399959; ENST00000457138; ENST00000542215	stop_gained	p.Ser131*/c.392C>G;p.Ser115*/c.344C>G;p.Ser175*/c.524C>G
UPN042	4	153249385	G	A	1545	0	1548	0.00 %	1107	581	1692	34.34 %	FBXW7	ENST00000281708; ENST00000296555; ENST00000263981; ENST00000603548; ENST00000393956; ENST00000603841	missense_variant	p.Arg465Cys/c.1393C>T;p.Arg347Cys/c.1039C>T;p.Arg385Cys/c.1153C>T;p.Arg289Cys/c.865C>T
UPN043	14	99724015	TA	T	1825	0	1831	0.00 %	1233	311	1548	20.09 %	BCL11B	ENST00000357195; ENST00000345514	frameshift_variant	p.Phe73fs/c.219delT
UPN043	9	139399325	G	GAACCCCTGT	2023	0	2029	0.00 %	1693	324	1706	18.99 %	NOTCH1	ENST00000277541	disruptive_inframe_insertion	p.Val1605_Phe1606insLeuGlnGly/c.4817_4818insACAGGGGTT
UPN043	9	139399422	A	G	1954	2	1961	0.10 %	1672	150	1827	8.21 %	NOTCH1	ENST00000277541	missense_variant	p.Leu1574Pro/c.4721T>C
UPN043	9	139397768	A	G	1873	0	1879	0.00 %	1654	95	1756	5.41 %	NOTCH1	ENST00000277541	missense_variant	p.Leu1678Pro/c.5033T>C
UPN044	9	139399356	A	T	2377	3	2382	0.13 %	1148	947	2097	45.16 %	NOTCH1	ENST00000277541	missense_variant	p.Leu1596His/c.4787T>A
UPN045	14	99641755	T	C	1159	0	1164	0.00 %	480	471	954	49.37 %	BCL11B	ENST00000357195; ENST00000345514; ENST00000443726	missense_variant	p.His473Arg/c.1418A>G;p.His402Arg/c.1205A>G;p.His279Arg/c.836A>G
UPN045	9	139390813	C	A	2154	1	2160	0.05 %	941	913	1857	49.17 %	NOTCH1	ENST00000277541	stop_gained	p.Glu2460*/c.7378G>T
UPN045	1	115258748	C	T	1855	0	1859	0.00 %	793	533	1329	40.11 %	NRAS	ENST00000369535	missense_variant	p.Gly12Ser/c.34G>A
UPN045	10	89717697	T	TGGAGAAGGC	1555	0	1558	0.00 %	1092	399	1096	36.41 %	PTEN	ENST00000371953	disruptive_inframe_insertion	p.Phe241delinsLeuGluLysAla/c.722_723insGGAGAAGGC
UPN045	10	89717712	C	CAGGAAAGT	1698	0	1699	0.00 %	1096	386	1099	35.12 %	PTEN	ENST00000371953	frameshift_variant	p.Leu247fs/c.737_738insAGGAAAGT
UPN046	10	89653861	A	ACC	1012	0	1014	0.00 %	400	293	401	73.07 %	PTEN	ENST00000371953	frameshift_variant	p.Val54fs/c.159_160insCC
UPN047	9	139390666	A	AG	1646	0	1665	0.00 %	1812	598	1860	32.15 %	NOTCH1	ENST00000277541	frameshift_variant	p.Phe2509fs/c.7524dupC
UPN048	9	139399344	A	G	1717	0	1721	0.00 %	1176	965	2146	44.97 %	NOTCH1	ENST00000277541	missense_variant	p.Leu1600Pro/c.4799T>C
UPN048	4	153247366	C	A	1077	0	1079	0.00 %	639	516	1156	44.64 %	FBXW7	ENST00000281708; ENST00000296555; ENST00000263981; ENST00000603548; ENST00000393956; ENST00000603841	missense_variant	p.Arg479Leu/c.1436G>T;p.Arg361Leu/c.1082G>T;p.Arg399Leu/c.1196G>T;p.Arg303Leu/c.908G>T

UPN048	13	77900748	G	GC	1178	0	1180	0.00 %	1324	506	1332	37.99 %	MYCBP2	ENST00000407578; ENST00000544440; ENST00000357337	frameshift_variant	p.Leu55fs/c.162dupG;p.Leu17fs/c.48dupG
UPN049	10	89692788	A	AAGG	989	0	993	0.00 %	870	286	877	32.61 %	PTEN	ENST00000371953	disruptive_inframe_insertion	p.Glu91_Asp92insGly/c.274_275insGAG
UPN049	9	139396885	C	CGCCGCCACGTACATGAAGTGCAGCTGGAATGGGATG	1060	0	1062	0.00 %	928	26	928	2.80 %	NOTCH1	ENST00000277541	disruptive_inframe_insertion	p.Ala1741_Ala1742insIleProPheGlnLeuHisPheMetTyrValAlaAla/c.5222_5223insCATCCCATTCCAGCTGCACTTCATGTACGTGGCGGC
UPN050	4	153247289	G	A	1075	0	1077	0.00 %	664	644	1309	49.20 %	FBXW7	ENST00000281708; ENST00000296555; ENST00000263981; ENST00000603548; ENST00000393956; ENST00000603841	missense_variant	p.Arg505Cys/c.1513C>T;p.Arg387Cys/c.1159C>T;p.Arg425Cys/c.1273C>T;p.Arg329Cys/c.985C>T
UPN050	9	139399344	A	G	1516	2	1522	0.13 %	1167	573	1740	32.93 %	NOTCH1	ENST00000277541	missense_variant	p.Leu1600Pro/c.4799T>C
UPN050	17	40359659	T	A	1109	1	1114	0.09 %	865	379	1244	30.47 %	STAT5B	ENST00000293328	missense_variant	p.Tyr665Phe/c.1994A>T
UPN050	9	139397762	A	T	1445	0	1451	0.00 %	1531	149	1684	8.85 %	NOTCH1	ENST00000277541	missense_variant	p.Ile1680Asn/c.5039T>A
UPN050	9	139399422	A	G	1422	1	1426	0.07 %	1584	135	1723	7.84 %	NOTCH1	ENST00000277541	missense_variant	p.Leu1574Pro/c.4721T>C
UPN050	4	153268089	A	AT	904	0	904	0.00 %	993	57	994	5.73 %	FBXW7	ENST00000281708; ENST00000296555; ENST00000263981; ENST00000603548; ENST00000393956; ENST00000603841	frameshift_variant	p.Met240fs/c.718dupA;p.Met122fs/c.364dupA; p.Met160fs/c.478dupA;p.Met64fs/c.190dupA
UPN051	X	133547934	G	T	487	0	488	0.00 %	30	449	479	93.74 %	PHF6	ENST00000394292; ENST00000370803; ENST00000332070; ENST00000370799; ENST00000416404; ENST00000370800	stop_gained	p.Glu224*/c.670G>T;p.Glu223*/c.667G>T;p.Glu189*/c.565G>T

UPN052	X	41200805	TCTC	CCTCTCGG	503	0	504	0.00 %	84	365	452	80.75 %	DDX3X	ENST00000399959; ENST00000457138; ENST00000441189; ENST00000542215	frameshift_variant+missense_variant	p.Ser74fs/c.220_223delTCTCinsCCTCTCGG;p.Ser58fs/c.172_175delTCTCinsCCTCTCGG;p.Ser118fs/c.352_355delTCTCinsCCTCTCGG
UPN052	X	133511716	T	TTGGGTTAAAATAGG	520	0	520	0.00 %	461	297	464	64.01 %	PHF6	ENST00000394292; ENST00000370803; ENST00000332070; ENST00000370799; ENST00000416404; ENST00000370800	frameshift_variant	p.Arg24fs/c.69_70insTGGGTTAAAATAGG
UPN052	9	139399367	G	GCCC	1553	0	1554	0.00 %	1389	571	1392	41.02 %	NOTCH1	ENST00000277541	disruptive_inframe_insertion	p.Phe1592delinsLeuGly/c.4775_4776insGGG
UPN052	1	9779982	T	C	1185	0	1186	0.00 %	714	469	1188	39.48 %	PIK3CD	ENST00000361110; ENST00000536656; ENST00000377346; ENST00000543390	missense_variant	p.Cys381Arg/c.1141T>C;p.Cys416Arg/c.1246T>C;p.Cys83Arg/c.247T>C
UPN052	9	139391019	T	TAAG	1535	0	1544	0.00 %	1516	554	1536	36.07 %	NOTCH1	ENST00000277541	stop_gained+inframe_insertion	p.Gln2391delinsProTer/c.7171_7172insCTT
UPN053	16	3779603	GC	G	1089	0	1090	0.00 %	1119	318	1446	21.99 %	CREBBP	ENST00000262367; ENST00000382070	frameshift_variant	p.Gly1815fs/c.5444delG;p.Gly1777fs/c.5330delG
UPN053	19	4048233	C	A	1284	2	1289	0.16 %	1164	329	1497	21.98 %	ZBTB7A	ENST00000322357; ENST00000601588	missense_variant	p.Lys424Asn/c.1272G>T
UPN053	9	139399410	A	C	1270	0	1271	0.00 %	1116	264	1388	19.02 %	NOTCH1	ENST00000277541	missense_variant	p.Val1578Gly/c.4733T>G
UPN053	1	9779982	T	C	1049	1	1052	0.10 %	1260	105	1368	7.68 %	PIK3CD	ENST00000361110; ENST00000536656; ENST00000377346; ENST00000543390	missense_variant	p.Cys381Arg/c.1141T>C;p.Cys416Arg/c.1246T>C;p.Cys83Arg/c.247T>C
UPN054	16	3799648	A	AT	777	0	778	0.00 %	591	242	596	40.60 %	CREBBP	ENST00000262367; ENST00000382070; ENST00000573517; ENST00000570939	frameshift_variant	p.Asn1272fs/c.3815dupA;p.Asn1234fs/c.3701dupA;p.Asn40fs/c.119dupA;p.Asn145fs/c.434dupA
UPN054	9	139399408	GCAC	G	1823	10	1837	0.54 %	1169	343	1513	22.67 %	NOTCH1	ENST00000277541	inframe_deletion	p.Val1578del/c.4732_4734delGTG
UPN054	1	115258747	C	T	1260	0	1261	0.00 %	974	179	1156	15.48 %	NRAS	ENST00000369535	missense_variant	p.Gly12Asp/c.35G>A

UPN054	16	3794894	C	T	1189	0	1193	0.00 %	912	82	996	8.23 %	CREBBP	ENST00000262367; ENST00000382070; ENST00000574740; ENST00000573517; ENST00000570939; ENST00000572569	splice_donor_variant+intron_variant	c.3982+1G>A;c.3868+1G>A;n.64+1G>A;c.286+1G>A;c.601+1G>A;n.446+1G>A
UPN054	12	122252315	G	GCGAGCCCGGAGCCGGC	615	0	624	0.00 %	458	35	463	7.56 %	SETD1B	ENST00000604567; ENST00000542440; ENST00000267197	frameshift_variant	p.Pro733fs/c.2196_2197insAGCCCGGAGCCGGCCG
UPN055	X	133512118	T	TTTTGGGG	606	0	607	0.00 %	362	233	364	64.01 %	PHF6	ENST00000394292; ENST00000370803; ENST00000332070; ENST00000370799; ENST00000370800	frameshift_variant	p.Lys75fs/c.222_223insTTTGGGG
UPN055	9	139399344	A	G	2389	2	2396	0.08 %	1306	607	1919	31.63 %	NOTCH1	ENST00000277541	missense_variant	p.Leu1600Pro/c.4799T>C
UPN055	4	153244185	G	A	1644	0	1649	0.00 %	881	365	1249	29.22 %	FBXW7	ENST00000281708; ENST00000296555; ENST00000263981; ENST00000603548; ENST00000393956; ENST00000603841	stop_gained	p.Arg658*/c.1972C>T;p.Arg540*/c.1618C>T;p.Arg578*/c.1732C>T;p.Arg482*/c.1444C>T
UPN055	4	153253801	C	CA	1335	0	1338	0.00 %	1087	298	1091	27.31 %	FBXW7	ENST00000281708; ENST00000296555; ENST00000263981; ENST00000603548; ENST00000393956; ENST00000603841	frameshift_variant	p.Trp311fs/c.931dupT;p.Trp193fs/c.577dupT;p.Trp231fs/c.691dupT;p.Trp135fs/c.403dupT
UPN055	14	99640604	C	CGCGAAT	1715	0	1719	0.00 %	1604	391	1607	24.33 %	BCL11B	ENST00000357195; ENST00000345514; ENST00000443726	inframe_insertion	p.Cys856_Asp857insIleArg/c.2568_2569insATTCGC;p.Cys785_Asp786insIleArg/c.2355_2356insATTCGC;p.Cys662_Asp663insIleArg/c.1986_1987insATTCGC
UPN055	9	139399296	A	T	2048	0	2050	0.00 %	1532	100	1637	6.11 %	NOTCH1	ENST00000277541	missense_variant	p.Ile1616Asn/c.4847T>A

UPN055	4	153249385	G	A	1691	1	1696	0.06 %	1343	24	1370	1.75 %	FBXW7	ENST00000281708; ENST00000296555; ENST00000263981; ENST00000603548; ENST00000393956; ENST00000603841	missense_variant	p.Arg465Cys/c.1393C>T;p.Arg347Cys/c.1039C>T;p.Arg385Cys/c.1153C>T;p.Arg289Cys/c.865C>T
UPN055	9	139399286	GTAG	CCCCA	2072	0	2082	0.00 %	1631	21	1658	1.27 %	NOTCH1	ENST00000277541	missense_variant	p.ProTyr1618LeuGly/c.4853_4857delCCTACinsT
UPN056	9	139397762	A	T	1454	1	1459	0.07 %	896	779	1682	46.31 %	NOTCH1	ENST00000277541	missense_variant	p.Ile1680Asn/c.5039T>A
UPN056	3	178927980	T	C	633	1	635	0.16 %	589	144	735	19.59 %	PIK3CA	ENST00000263967	missense_variant	p.Cys420Arg/c.1258T>C
UPN056	3	178916931	CAACCGT	AAAA	663	4	665	0.60 %	700	45	745	6.04 %	PIK3CA	ENST00000263967; ENST00000468036	missense_variant+inframe_deletion	p.Asn107_Arg108delinsLys/c.318_324delCAACCGTinsAAAA
UPN056	5	67589596	C	CGGG	769	0	769	0.00 %	751	30	753	3.98 %	PIK3R1	ENST00000396611; ENST00000521381; ENST00000521657; ENST00000274335; ENST00000320694; ENST00000521409; ENST00000336483; ENST00000519025; ENST00000523872	inframe_insertion	p.Asn453_Thr454insGly/c.1359_1360insGGG;p.Asn153_Thr154insGly/c.459_460insGGG;p.Asn90_Thr91insGly/c.270_271insGGG;p.Asn183_Thr184insGly/c.549_550insGGG;p.Asn126_Thr127insGly/c.378_379insGGG
UPN056	3	178936091	G	A	917	0	920	0.00 %	966	38	1006	3.78 %	PIK3CA	ENST00000263967	missense_variant	p.Glu545Lys/c.1633G>A
UPN056	3	178952146	G	GA	825	0	825	0.00 %	921	20	924	2.16 %	PIK3CA	ENST00000263967	frameshift_variant	p.Asn1068fs/c.3203dupA
UPN057	X	133551254	G	A	601	0	602	0.00 %	101	353	455	77.58 %	PHF6	ENST00000394292; ENST00000370803; ENST00000332070; ENST00000370799; ENST00000416404	missense_variant	p.Cys298Tyr/c.893G>A;p.Cys297Tyr/c.890G>A;p.Cys263Tyr/c.788G>A
UPN057	14	99641881	A	C	1303	1	1309	0.08 %	679	512	1192	42.95 %	BCL11B	ENST00000357195; ENST00000345514; ENST00000443726	missense_variant	p.Phe431Cys/c.1292T>G;p.Phe360Cys/c.1079T>G;p.Phe237Cys/c.710T>G

UPN057	4	153247289	G	A	1389	2	1394	0.14 %	646	478	1124	42.53 %	FBXW7	ENST00000281708; ENST00000296555; ENST00000263981; ENST00000603548; ENST00000393956; ENST00000603841	missense_variant	p.Arg505Cys/c.1513C>T;p.Arg387Cys/c.1159C>T;p.Arg425Cys/c.1273C>T;p.Arg329Cys/c.985C>T
UPN057	16	3779661	T	TA	1375	0	1378	0.00 %	1384	583	1392	41.88 %	CREBBP	ENST00000262367; ENST00000382070	frameshift_variant	p.Gln1796fs/c.5386_5387insT;p.Gln1758fs/c.5272_5273insT
UPN057	9	139397761	AATC	GCTA	1638	1	1643	0.06 %	832	515	1352	38.09 %	NOTCH1	ENST00000277541	missense_variant	p.GluIle1679AspSer/c.5037_5040delGATTinsTA
UPN057	16	9010979	G	GGTCCCTCCACTC	926	0	930	0.00 %	792	222	803	27.65 %	USP7	ENST00000344836; ENST00000381886; ENST00000535863; ENST00000563085; ENST00000542333	stop_gained+inframe_insertion	p.Ser252delinsTerValGluGlyPro/c.754_755insGAGTGGAGGGAC;p.Ser236delinsTerValGluGlyPro/c.706_707insGAGTGGAGGGAC;p.Ser153delinsTerValGluGlyPro/c.457_458insGAGTGGAGGGAC;p.Ser194delinsTerValGluGlyPro/c.580_581insGAGTGGAGGGAC
UPN057	5	67591096	GAACAGCATTAAACCA	G	1099	0	1103	0.00 %	802	73	891	8.19 %	PIK3R1	ENST00000396611; ENST00000521381; ENST00000521657; ENST00000274335; ENST00000320694; ENST00000336483; ENST00000523872	inframe_deletion	p.Asn564_Pro568del/c.1690_1704delAACAGCATTAAACCA;p.Asn264_Pro268del/c.790_804delAACAGCATTAAACCA;p.Asn294_Pro298del/c.880_894delAACAGCATTAAACCA;p.Asn201_Pro205del/c.601_615delAACAGCATTAAACCA
UPN058	9	139390732	G	A	1447	1	1452	0.07 %	1316	534	1851	28.85 %	NOTCH1	ENST00000277541	stop_gained	p.Gln2487*/c.7459C>T
UPN058	9	139412688	TGGA	T	1268	0	1273	0.00 %	1417	380	1801	21.10 %	NOTCH1	ENST00000277541	inframe_deletion	p.Ser385del/c.1153_1155delTCC
UPN058	9	139391008	G	A	1678	1	1685	0.06 %	2034	22	2058	1.07 %	NOTCH1	ENST00000277541	stop_gained	p.Gln2395*/c.7183C>T
UPN059	9	139399368	A	G	1663	0	1665	0.00 %	1291	403	1696	23.76 %	NOTCH1	ENST00000277541	missense_variant	p.Phe1592Ser/c.4775T>C
UPN059	9	139390716	G	T	1607	0	1613	0.00 %	1238	363	1605	22.62 %	NOTCH1	ENST00000277541	stop_gained	p.Ser2492*/c.7475C>A
UPN059	9	139390779	G	T	1595	1	1599	0.06 %	1204	338	1544	21.89 %	NOTCH1	ENST00000277541	stop_gained	p.Ser2471*/c.7412C>A
UPN059	19	17949121	T	G	1491	4	1497	0.27 %	1142	200	1346	14.86 %	JAK3	ENST00000458235; ENST00000527670; ENST00000534444	missense_variant	p.Gln507Pro/c.1520A>C
UPN059	19	17948006	G	A	1329	1	1333	0.08 %	1209	141	1354	10.41 %	JAK3	ENST00000458235; ENST00000527670; ENST00000534444	missense_variant	p.Ala573Val/c.1718C>T
UPN059	17	40359729	T	G	903	0	905	0.00 %	840	62	904	6.86 %	STAT5B	ENST00000293328	missense_variant	p.Asn642His/c.1924A>C
UPN059	9	139397727	A	G	1700	1	1704	0.06 %	1523	53	1587	3.34 %	NOTCH1	ENST00000277541	missense_variant	p.Cys1692Arg/c.5074T>C

UPN059	9	139399296	A	G	1567	2	1570	0.13 %	1516	31	1551	2.00 %	NOTCH1	ENST00000277541	missense_variant	p.Ile1616Thr/c.4847T>C
UPN059	9	139399389	A	T	1602	0	1612	0.00 %	1574	23	1603	1.43 %	NOTCH1	ENST00000277541	missense_variant	p.Leu1585Gln/c.4754T>A
UPN060	no Varian															
UPN061	X	133547908	T	C	1061	0	1064	0.00 %	451	326	777	41.96 %	PHF6	ENST00000394292; ENST00000370803; ENST00000332070; ENST00000370799; ENST00000416404; ENST00000370800	missense_variant	p.Phe215Ser/c.644T>C;p.Phe214Ser/c.641T>C; p.Phe180Ser/c.539T>C
UPN061	6	41903745	C	CG	1739	0	1742	0.00 %	1883	304	1896	16.03 %	CCND3	ENST00000372991; ENST00000511642; ENST00000372987; ENST00000415497; ENST00000372988; ENST00000414200; ENST00000510503	frameshift_variant	p.Arg271fs/c.811dupC;p.Arg190fs/c.568dupC;p.Arg221fs/c.661dupC;p.Arg75fs/c.223dupC;p.Arg199fs/c.595dupC;p.Leu146fs/c.431dupC
UPN061	9	139399344	A	G	2176	0	2183	0.00 %	1630	254	1892	13.42 %	NOTCH1	ENST00000277541	missense_variant	p.Leu1600Pro/c.4799T>C
UPN061	14	99642119	T	TCA	1585	0	1588	0.00 %	1218	160	1227	13.04 %	BCL11B	ENST00000357195; ENST00000345514; ENST00000443726	frameshift_variant	p.Asn352fs/c.1052_1053dupTG;p.Asn281fs/c.839_840dupTG;p.Asn158fs/c.470_471dupTG
UPN061	6	41903803	T	TCC	1777	0	1783	0.00 %	1969	245	1977	12.39 %	CCND3	ENST00000372991; ENST00000511642; ENST00000372987; ENST00000415497; ENST00000372988; ENST00000414200; ENST00000510503	frameshift_variant	p.Arg252fs/c.753_754insGG;p.Arg171fs/c.510_511insGG;p.Arg202fs/c.603_604insGG;p.Arg56fs/c.165_166insGG;p.Arg180fs/c.537_538insGG; p.Gln125fs/c.373_374insGG
UPN061	14	99641777	G	GGTAAC	1021	0	1022	0.00 %	1018	126	1018	12.38 %	BCL11B	ENST00000357195; ENST00000345514; ENST00000443726	frameshift_variant	p.Gln466fs/c.1395_1396insGTTAC;p.Gln395fs/c.1182_1183insGTTAC;p.Gln272fs/c.813_814insGTTAC
UPN061	9	139390648	CAG	C	1722	0	1726	0.00 %	1430	139	1581	8.79 %	NOTCH1	ENST00000277541	frameshift_variant	p.Pro2514fs/c.7541_7542delCT
UPN061	10	89692791	A	G	969	1	971	0.10 %	709	67	778	8.61 %	PTEN	ENST00000371953	missense_variant	p.Asp92Gly/c.275A>G
UPN061	10	89692905	G	A	1241	0	1243	0.00 %	954	60	1014	5.92 %	PTEN	ENST00000371953	missense_variant	p.Arg130Gln/c.389G>A

UPN062	16	8999171	G	C	1055	0	1058	0.00 %	829	291	1122	25.94 %	USP7	ENST00000344836; ENST00000381886; ENST00000535863; ENST00000563085; ENST00000542333	missense_variant	p.Asp482Glu/c.1446C>G;p.Asp466Glu/c.1398C>G;p.Asp383Glu/c.1149C>G;p.Asp424Glu/c.1272C>G
UPN062	10	89717697	T	TGGGTAGTA	878	0	880	0.00 %	1047	219	1053	20.80 %	PTEN	ENST00000371953	frameshift_variant	p.Phe241fs/c.722_723insGGGTAGTA
UPN062	9	139390821	A	AGG	1324	0	1325	0.00 %	1846	108	1855	5.82 %	NOTCH1	ENST00000277541	frameshift_variant	p.Leu2457fs/c.7369_7370insCC
UPN063	21	36171607	G	A	1347	0	1349	0.00 %	675	564	1239	45.52 %	RUNX1	ENST00000300305; ENST00000344691; ENST00000325074; ENST00000437180; ENST00000399240	stop_gained	p.Arg320*/c.958C>T;p.Arg293*/c.877C>T;p.Arg308*/c.922C>T;p.Arg229*/c.685C>T
UPN063	19	17949121	T	G	1435	3	1446	0.21 %	693	567	1264	44.86 %	JAK3	ENST00000458235; ENST00000527670; ENST00000534444	missense_variant	p.Gln507Pro/c.1520A>C
UPN063	19	17948006	G	A	1375	0	1376	0.00 %	765	619	1391	44.50 %	JAK3	ENST00000458235; ENST00000527670; ENST00000534444	missense_variant	p.Ala573Val/c.1718C>T
UPN063	9	139399344	A	T	1923	0	1930	0.00 %	2159	242	2403	10.07 %	NOTCH1	ENST00000277541	missense_variant	p.Leu1600Gln/c.4799T>A
UPN064	9	139399350	C	G	2672	3	2679	0.11 %	1390	1332	2727	48.84 %	NOTCH1	ENST00000277541	missense_variant	p.Arg1598Pro/c.4793G>C
UPN064	4	153247366	C	T	1674	0	1680	0.00 %	777	618	1399	44.17 %	FBXW7	ENST00000281708; ENST00000296555; ENST00000263981; ENST00000603548; ENST00000393956; ENST00000603841	missense_variant	p.Arg479Gln/c.1436G>A;p.Arg361Gln/c.1082G>A;p.Arg399Gln/c.1196G>A;p.Arg303Gln/c.908G>A
UPN064	1	115256536	C	T	1645	0	1649	0.00 %	1413	97	1517	6.39 %	NRAS	ENST00000369535	missense_variant	p.Ala59Thr/c.175G>A
UPN064	12	122261179	A	AT	2242	0	2294	0.00 %	2292	141	2358	5.98 %	SETD1B	ENST00000604567; ENST00000542440; ENST00000267197	frameshift_variant+stop_gained	p.Asp1566fs/c.4695dupT;p.Asp1523fs/c.4566dupT
UPN065	9	139397768	A	G	1990	1	1996	0.05 %	1072	1114	2216	50.27 %	NOTCH1	ENST00000277541	missense_variant	p.Leu1678Pro/c.5033T>C

UPN065	19	10940881	GCC	GA	1453	2	1454	0.14 %	810	752	1566	48.02 %	DNM2	ENST00000314646; ENST00000585892; ENST00000359692; ENST00000389253; ENST00000355667; ENST00000408974; ENST00000589106	frameshift_variant+missense_variant	p.Pro791fs/c.2371_2372delCCinsA;p.Pro787fs/c.2359_2360delCCinsA;p.Pro79fs/c.235_236delCCinsA
UPN065	11	118390713	ATC	ATGGTCCTTT	1526	6	1532	0.39 %	911	481	1431	33.61 %	KMT2A	ENST00000534358; ENST00000389506; ENST00000354520	frameshift_variant+missense_variant	p.Arg3789fs/c.11365delCinsGGTCCTTT;p.Arg3786fs/c.11356delCinsGGTCCTTT;p.Arg3748fs/c.11242delCinsGGTCCTTT
UPN065	6	41903807	T	TGCAGGTTACGGGG	2167	0	2175	0.00 %	2121	523	2152	24.30 %	CCND3	ENST00000372991; ENST00000511642; ENST00000372987; ENST00000415497; ENST00000372988; ENST00000414200; ENST00000510503	frameshift_variant	p.Leu251fs/c.749_750insCCCCGTAACCTGC;p.Leu170fs/c.506_507insCCCCGTAACCTGC;p.Leu201fs/c.599_600insCCCCGTAACCTGC;p.Leu55fs/c.161_162insCCCCGTAACCTGC;p.Leu179fs/c.533_534insCCCCGTAACCTGC;p.Thr124fs/c.369_370insCCCCGTAACCTGC

ID	Chr	Pos	Ref	Alt	Germline #REF	Germline #ALT	Germline DP	Germline VAF	Tumor #REF	Tumor #ALT	Tumor DP	Tumor VAF	Gene	ENST	Type	Variant
UPN066_p	9	139391014	G	A	2182	0	2188	0.00 %	1679	576	2265	25.43 %	NOTCH1	ENST00000277541	stop_gained	p.Gln2393*/c.7177C>T
UPN066_p	9	139399285	AGTAGGGGAAGA	A	1871	0	1872	0.00 %	1389	271	1664	16.29 %	NOTCH1	ENST00000277541	inframe_deletion	p.His1611_Tyr1619del/c.4831_4857delCACGGCCAGCAGATGATCTTCCCCTAC
UPN066_p	5	67591144	ATA	ACCTACGGACGTGTC	1063	6	1069	0.56 %	961	149	1122	13.28 %	PIK3R1	ENST00000396611;ENST00000521381;ENST00000521657;ENST00000274335;ENST00000320694;ENST00000336483;ENST00000523872	missense_variant+disruptive_inframe_insertion	p.Tyr580delinsProThrAspValSer/c.1738_1739delTAinsCCTACGGACGTGTC;p.Tyr280delinsProThrAspValSer/c.838_839delTAinsCCTACGGACGTGTC;p.Tyr310delinsProThrAspValSer/c.928_929delTAinsCCTACGGACGTGTC;p.Tyr217delinsProThrAspValSer/c.649_650delTAinsCCTACGGACGTG
UPN066_p	9	139399312	G	A	1912	0	1914	0.00 %	1430	121	1553	7.79 %	NOTCH1	ENST00000277541	missense_variant	p.His1611Tyr/c.4831C>T
UPN067_p	14	99640879	G	T	689	0	690	0.00 %	431	80	514	15.56 %	BCL11B	ENST00000357195;ENST00000345514;ENST00000443726	stop_gained	p.Ser765*/c.2294C>A;p.Ser694*/c.2081C>A;p.Ser571*/c.1712C>A
UPN067_p	19	4055148	C	T	2223	0	2228	0.00 %	1425	236	1663	14.19 %	ZBTB7A	ENST00000322357;ENST00000601588	missense_variant	p.Arg28Gln/c.83G>A
UPN067_p	9	139390655	C	CG	2154	0	2164	0.00 %	1555	211	1558	13.54 %	NOTCH1	ENST00000277541	frameshift_variant	p.Ser2513fs/c.7535dupC
UPN067_p	9	139390789	G	GCGGCC	2315	0	2320	0.00 %	1609	174	1614	10.78 %	NOTCH1	ENST00000277541	frameshift_variant	p.Leu2468fs/c.7401_7402insGGCCG
UPN067_p	16	9009168	A	ACCGGGC	1505	0	1506	0.00 %	1127	84	1131	7.43 %	USP7	ENST00000344836;ENST00000381886;ENST00000535863;ENST00000563085;ENST00000542333	inframe_insertion	p.Arg340_Ser341insAlaArg/c.1020_1021insGCCCGG;p.Arg324_Ser325insAlaArg/c.972_973insGCCCGG;p.Arg241_Ser242insAlaArg/c.723_724insGCCCGG;p.Arg282_Ser283insAlaArg/c.846_847insGCCCGG
UPN067_p	9	139397775	C	A	2149	1	2154	0.05 %	1593	74	1671	4.43 %	NOTCH1	ENST00000277541	missense_variant	p.Val1676Phe/c.5026G>T
UPN067_p	17	40359729	T	G	1348	3	1353	0.22 %	1017	42	1061	3.96 %	STAT5B	ENST00000293328	missense_variant	p.Asn642His/c.1924A>C
UPN067_p	3	178936082	G	A	1647	0	1649	0.00 %	1251	48	1304	3.68 %	PIK3CA	ENST00000263967	missense_variant	p.Glu542Lys/c.1624G>A
UPN068_p	X	44942752	G	GTGTCGGATACCAGCCATT	461	0	461	0.00 %	500	274	503	54.47 %	KDM6A	ENST00000382899;ENST00000377967;ENST00000536777;ENST00000543216;ENST00000414389;ENST00000433797	disruptive_inframe_insertion	p.Val1119_Val1120insGlyTyrGlnProPheVal/c.3358_3359insGATACCAGCCATTTGTCG;p.Val1112_Val1113insGlyTyrGlnProPheVal/c.3337_3338insGATACCAGCCATTTGTCG;p.Val1067_Val1068insGlyTyrGlnProPheVal/c.3202_3203insGATACCAGCCATTTGTCG;p.Val1033_Val1034insGlyTyrGlnProPheVal/c.3100_3101insGATACCAGC

UPN068_p	4	153249384	C	T	1053	0	1055	0.00 %	745	514	1264	40.66 %	FBXW7	ENST00000281708;ENST00000296555;ENST00000263981;ENST00000603548;ENST00000393956;ENST00000603841	missense_variant	p.Arg465His/c.1394G>A;p.Arg347His/c.1040G>A;p.Arg385His/c.1154G>A;p.Arg289His/c.866G>A
UPN068_p	9	139397768	A	G	1399	1	1402	0.07 %	1103	695	1803	38.55 %	NOTCH1	ENST00000277541	missense_variant	p.Leu1678Pro/c.5033T>C
UPN068_p	19	15292520	C	G	1209	0	1214	0.00 %	1425	225	1651	13.63 %	NOTCH3	ENST00000263388;ENST00000601011	missense_variant	p.Asp887His/c.2659G>C;p.Asp834His/c.2500G>C
UPN069_p	X	133549136	C	T	514	0	516	0.00 %	180	228	408	55.88 %	PHF6	ENST00000394292;ENST00000370803;ENST00000332070;ENST00000370799;ENST00000416404;ENST00000370800	stop_gained	p.Arg275*/c.823C>T;p.Arg274*/c.820C>T;p.Arg240*/c.718C>T
UPN069_p	4	153249384	C	T	1342	1	1346	0.07 %	857	204	1064	19.17 %	FBXW7	ENST00000281708;ENST00000296555;ENST00000263981;ENST00000603548;ENST00000393956;ENST00000603841	missense_variant	p.Arg465His/c.1394G>A;p.Arg347His/c.1040G>A;p.Arg385His/c.1154G>A;p.Arg289His/c.866G>A
UPN069_p	9	139399325	G	GGGCCCC	1847	0	1849	0.00 %	1520	91	1523	5.98 %	NOTCH1	ENST00000277541	disruptive_inframe_insertion	p.Phe1606delinsLeuGlyPro/c.4817_4818insGGGGCC
UPN069_p	9	139399325	G	GGAGAATCCT	1847	0	1849	0.00 %	1520	77	1523	5.06 %	NOTCH1	ENST00000277541	disruptive_inframe_insertion	p.Phe1606delinsLeuGlyPheSer/c.4817_4818insAGGATTCTC
UPN069_p	4	153249510	C	A	1308	2	1313	0.15 %	1071	56	1127	4.97 %	FBXW7	ENST00000281708;ENST00000296555;ENST00000263981;ENST00000603548;ENST00000393956;ENST00000603841	missense_variant	p.Gly423Val/c.1268G>T;p.Gly305Val/c.914G>T;p.Gly343Val/c.1028G>T;p.Gly247Val/c.740G>T
UPN069_p	9	139397768	A	G	1618	0	1622	0.00 %	1354	52	1419	3.66 %	NOTCH1	ENST00000277541	missense_variant	p.Leu1678Pro/c.5033T>C
UPN069_p	9	139399350	C	G	1798	1	1800	0.06 %	1505	32	1538	2.08 %	NOTCH1	ENST00000277541	missense_variant	p.Arg1598Pro/c.4793G>C
UPN069_p	9	139399344	A	T	1879	0	1888	0.00 %	1507	29	1562	1.86 %	NOTCH1	ENST00000277541	missense_variant	p.Leu1600Gln/c.4799T>A
UPN069_p	9	139399344	A	G	1879	0	1888	0.00 %	1507	24	1562	1.54 %	NOTCH1	ENST00000277541	missense_variant	p.Leu1600Pro/c.4799T>C
UPN070_p	X	133527636	C	T	332	0	332	0.00 %	304	94	398	23.62 %	PHF6	ENST00000394292;ENST00000370803;ENST00000332070;ENST00000370799;ENST00000416404;ENST00000370800	stop_gained	p.Arg116*/c.346C>T;p.Arg82*/c.244C>T

UPN070_p	17	7577548	C	A	1204	1	1205	0.08 %	1313	248	1564	15.86 %	TP53	ENST00000269305;ENST00000413465;ENST00000359597;ENST00000420246;ENST00000455263;ENST00000445888;ENST00000509690;ENST00000514944	missense_variant	p.Gly245Cys/c.733G>T;p.Gly113Cys/c.337G>T;p.Gly152Cys/c.454G>T
UPN070_p	19	17945969	C	T	1253	3	1258	0.24 %	1575	270	1848	14.61 %	JAK3	ENST00000458235;ENST00000527670;ENST00000534444	missense_variant	p.Arg657Gln/c.1970G>A
UPN070_p	17	40354460	A	T	645	0	648	0.00 %	719	115	834	13.79 %	STAT5B	ENST00000293328	missense_variant	p.Val712Glu/c.2135T>A
UPN070_p	1	65310517	C	T	1080	0	1084	0.00 %	1102	173	1279	13.53 %	JAK1	ENST00000342505	missense_variant	p.Arg724His/c.2171G>A
UPN071_p	9	139399386	C	G	2004	2	2008	0.10 %	860	678	1542	43.97 %	NOTCH1	ENST00000277541	missense_variant	p.Arg1586Pro/c.4757G>C
UPN071_p	X	70338634	ACACCGG	A	1542	0	1543	0.00 %	677	373	1052	35.46 %	MED12	ENST00000333646;ENST00000374102;ENST00000374080	disruptive_inframe_deletion	p.His11_Arg12del/c.32_37delACCGGC
UPN072_p	5	35874570	ACTA	GGGGTTAAAGTGTG	1387	6	1393	0.43 %	1086	150	1240	12.10 %	IL7R	ENST00000303115	missense_variant+inframe_insertion	p.Leu243delinsGlyLeuLysCysGly/c.726_729delACTAinsGGGGTTAAAGTGTGGT
UPN072_p	9	139390656	G	A	1873	2	1879	0.11 %	1682	214	1903	11.25 %	NOTCH1	ENST00000277541	missense_variant	p.Pro2512Leu/c.7535C>T
UPN072_p	9	139397640	C	T	1979	0	1982	0.00 %	1776	172	1951	8.82 %	NOTCH1	ENST00000277541	missense_variant	p.Val1721Met/c.5161G>A
UPN072_p	12	25378562	C	T	1302	3	1311	0.23 %	1225	88	1316	6.69 %	KRAS	ENST00000256078;ENST00000311936	missense_variant	p.Ala146Thr/c.436G>A
UPN072_p	17	40359729	T	G	1314	2	1318	0.15 %	1162	56	1218	4.60 %	STAT5B	ENST00000293328	missense_variant	p.Asn642His/c.1924A>C
UPN072_p	9	139399368	A	AAGTGGAAGGAGCT	2264	0	2275	0.00 %	2175	79	2190	3.61 %	NOTCH1	ENST00000277541	inframe_insertion	p.His1591_Phe1592insTyrPheAsnAsnSerSerPheHis/c.4774_4775insACTTTAACAA
UPN072_p	9	139396883	G	GCCGGAGGAGCCCC	1156	0	1159	0.00 %	1209	28	1211	2.31 %	NOTCH1	ENST00000277541	inframe_insertion	p.Ala1741_Ala1742insAspSerPheArgLeuTrpTyrGlyAlaProPro/c.5224_5225insATTC
UPN072_p	9	139396881	C	CGGCCGCCGCCACG	1171	0	1176	0.00 %	1201	26	1203	2.16 %	NOTCH1	ENST00000277541	inframe_insertion	p.Ala1742_Ala1743insSerSerThrSerAsnTyrValAlaAlaAla/c.5226_5227insTCCTCTA
UPN072_p	17	7577569	A	G	1961	0	1964	0.00 %	1685	37	1726	2.14 %	TP53	ENST00000269305;ENST00000413465;ENST00000359597;ENST00000420246;ENST00000455263;ENST00000445888;ENST00000509690;ENST00000514944	missense_variant	p.Cys238Arg/c.712T>C;p.Cys106Arg/c.316T>C;p.Cys145Arg/c.433T>C
UPN072_p	9	139397700	C	G	2321	3	2333	0.13 %	2102	43	2149	2.00 %	NOTCH1	ENST00000277541	missense_variant	p.Ala1701Pro/c.5101G>C
UPN072_p	9	139399344	A	G	2375	1	2381	0.04 %	2223	38	2265	1.68 %	NOTCH1	ENST00000277541	missense_variant	p.Leu1600Pro/c.4799T>C
UPN072_p	9	139397768	A	G	2229	1	2234	0.04 %	2001	22	2029	1.08 %	NOTCH1	ENST00000277541	missense_variant	p.Leu1678Pro/c.5033T>C

UPN073_p	1	115258747	C	T	1211	0	1215	0.00 %	854	771	1628	47.36 %	NRAS	ENST00000369535	missense_variant	p.Gly12Asp/c.35G>A
UPN073_p	9	139397675	A	G	1501	2	1505	0.13 %	1139	870	2010	43.28 %	NOTCH1	ENST00000277541	missense_variant	p.Leu1709Pro/c.5126T>C
UPN073_p	6	135510951	AGCACCGA	GGGTCCAC	992	0	993	0.00 %	683	414	1102	37.57 %	MYB	ENST00000341911;ENST00000339290;ENST00000367812;ENST00000463282;ENST00000525477;ENST00000533837;ENST00000316528;ENST00000442647;ENST00000367814;ENST00000527615;ENST00000420123;ENST00000525369;ENST00000524588;ENST00000525002;ENST00000525940;ENST00000526187;ENST00000526565;ENST00000528015;ENST00000528140;ENST00000528345;ENST00000528774;ENST00000529262;ENST00000531634;ENST00000531737;ENST00000533384;ENST00000533624;ENST00000534044;ENST00000534121;ENST00000528343;ENST00000438[illegible]	missense_variant	p.GlnHisArg79ArgValHis/c.236_243delAGCACCGAinsGGGTCCAC;p.GlnHisArg55ArgValHis/c.164_171delAGCACCGAinsGGGTCCAC;p.GlnHisArg33ArgValHis/c.98_105delAGCACCGAinsGGGTCCAC
UPN073_p	9	139390791	G	GACCCCTT	1388	0	1388	0.00 %	1809	546	1816	30.07 %	NOTCH1	ENST00000277541	frameshift_variant	p.Ser2467fs/c.7399_7400insTCGGAAGG
UPN074_p	7	150700291	G	A	455	413	869	47.53 %	21	466	488	95.49 %	NOS3	ENST00000484524	missense_variant	p.Cys602Tyr/c.1805G>A
UPN074_p	9	139399422	A	G	1685	0	1686	0.00 %	1053	954	2013	47.39 %	NOTCH1	ENST00000277541	missense_variant	p.Leu1574Pro/c.4721T>C
UPN074_p	4	153250906	G	A	933	0	935	0.00 %	606	538	1146	46.95 %	FBXW7	ENST00000281708;ENST00000296555;ENST00000263981;ENST00000603548;ENST00000393956;ENST00000603841	missense_variant	p.Thr385Ile/c.1154C>T;p.Thr267Ile/c.800C>T;p.Thr305Ile/c.914C>T;p.Thr209Ile/c.626C>T

UPN074_p	14	99641786	C	G	988	3	993	0.30 %	649	558	1209	46.15 %	BCL11B	ENST00000357195;ENST00000345514;ENST00000443726	missense_variant	p.Ala463Pro/c.1387G>C;p.Ala392Pro/c.1174G>C;p.Ala269Pro/c.805G>C
UPN075_p	4	153249384	C	T	1051	0	1053	0.00 %	939	526	1482	35.49 %	FBXW7	ENST00000281708;ENST00000296555;ENST00000263981;ENST00000603548;ENST00000393956;ENST00000603841	missense_variant	p.Arg465His/c.1394G>A;p.Arg347His/c.1040G>A;p.Arg385His/c.1154G>A;p.Arg289His/c.866G>A
UPN075_p	9	139399365	A	G	1269	0	1273	0.00 %	1042	588	1663	35.36 %	NOTCH1	ENST00000277541	missense_variant	p.Leu1593Pro/c.4778T>C
UPN075_p	4	153268144	G	A	987	0	988	0.00 %	827	405	1243	32.58 %	FBXW7	ENST00000281708;ENST00000296555;ENST00000263981;ENST00000603548;ENST00000393956;ENST00000603841	stop_gained	p.Arg222*/c.664C>T;p.Arg104*/c.310C>T;p.Arg142*/c.424C>T;p.Arg46*/c.136C>T
UPN075_p	1	115258748	C	T	1061	1	1063	0.09 %	941	382	1332	28.68 %	NRAS	ENST00000369535	missense_variant	p.Gly12Ser/c.34G>A
UPN075_p	16	9009376	C	CGCTAAAGCA	834	0	836	0.00 %	903	250	913	27.38 %	USP7	ENST00000344836;ENST00000381886;ENST00000535863;ENST00000563085;ENST00000542333	inframe_insertion	p.Leu304_Asp305insCysPheSer/c.912_913insTGCTTTAGC;p.Leu288_Asp289insCysPheSer/c.864_865insTGCTTTAGC;p.Leu205_Asp206insCysPheSer/c.615_616insTGCTTTAGC;p.Leu246_Asp247insCysPheS
UPN075_p	19	11141552	G	A	1078	0	1081	0.00 %	1181	58	1249	4.64 %	SMARCA4	ENST00000358026;ENST00000344626;ENST00000429416;ENST00000541122;ENST00000589677;ENST00000444061;ENST00000590574;ENST00000413806;ENST00000450717;ENST00000592158	missense_variant	p.Asp1177Asn/c.3529G>A;p.Asp22Asn/c.64G>A
UPN075_p	4	153258983	G	A	998	0	998	0.00 %	1122	36	1169	3.08 %	FBXW7	ENST00000281708;ENST00000296555;ENST00000263981;ENST00000603548;ENST00000393956;ENST00000603841	stop_gained	p.Arg278*/c.832C>T;p.Arg160*/c.478C>T;p.Arg198*/c.592C>T;p.Arg102*/c.304C>T

UPN075_p	4	153247289	G	A	1058	2	1064	0.19 %	1395	43	1446	2.97 %	FBXW7	ENST00000281708;ENST00000296555;ENST00000263981;ENST00000603548;ENST00000393956;ENST00000603841	missense_variant	p.Arg505Cys/c.1513C>T;p.Arg387Cys/c.1159C>T;p.Arg425Cys/c.1273C>T;p.Arg329Cys/c.985C>T
UPN075_p	9	139397768	A	G	1371	1	1379	0.07 %	1641	39	1728	2.26 %	NOTCH1	ENST00000277541	missense_variant	p.Leu1678Pro/c.5033T>C
UPN076_p	4	153249384	C	T	1254	0	1261	0.00 %	767	515	1287	40.02 %	FBXW7	ENST00000281708;ENST00000296555;ENST00000263981;ENST00000603548;ENST00000393956;ENST00000603841	missense_variant	p.Arg465His/c.1394G>A;p.Arg347His/c.1040G>A;p.Arg385His/c.1154G>A;p.Arg289His/c.866G>A
UPN076_p	4	153332886	AAGGGTTACCTCTCA	GG	1244	12	1256	0.96 %	687	369	1102	33.48 %	FBXW7	ENST00000281708;ENST00000603548;ENST00000603841;ENST00000604872;ENST00000605042	frameshift_variant+missense_variant	p.Leu19fs/c.56_70delTGAGAGGTAACCCTTinsCC
UPN076_p	9	139399408	GCAC	G	1611	6	1619	0.37 %	1182	542	1726	31.40 %	NOTCH1	ENST00000277541	inframe_deletion	p.Val1578del/c.4732_4734delGTG
UPN076_p	9	139397768	A	G	1648	1	1655	0.06 %	1538	57	1599	3.56 %	NOTCH1	ENST00000277541	missense_variant	p.Leu1678Pro/c.5033T>C
UPN076_p	9	139399293	A	G	1504	0	1506	0.00 %	1472	26	1503	1.73 %	NOTCH1	ENST00000277541	missense_variant	p.Phe1617Ser/c.4850T>C
UPN076_p	1	115258747	C	T	1362	0	1367	0.00 %	1379	21	1402	1.50 %	NRAS	ENST00000369535	missense_variant	p.Gly12Asp/c.35G>A
UPN077_p	16	9012983	TCTTTGAATCCCACC	CCCCTAAAACG	1124	9	1125	0.80 %	602	222	856	25.93 %	USP7	ENST00000344836;ENST00000381886;ENST00000535863;ENST00000563085;ENST00000542333;ENST00000563961;ENST00000565455	stop_gained+splice_acceptor_variant+missense_variant+inframe_deletion+splice_region_variant+intron_variant	p.Trp205_Lys209delinsPheTerGly???/c.612-1_625delGGTGGGATTCAAAGAinsCGTTTTAGGGG;p.Trp189_Lys193delinsPheTerGly???/c.564-1_577delGGTGGGATTCAAAGAinsCGTTTTAGGGG;p.Trp106_Lys110delinsPheTerGly???/c.315-1_328delGGTGGGATTCAAAGAinsCGTTTTAGGGG;p.Trp147_Lys151delinsPheTerG
UPN077_p	9	139399356	A	T	1834	1	1844	0.05 %	1385	347	1739	19.95 %	NOTCH1	ENST00000277541	missense_variant	p.Leu1596His/c.4787T>A
UPN077_p	9	139390648	CAG	C	1667	0	1681	0.00 %	1073	262	1345	19.48 %	NOTCH1	ENST00000277541	frameshift_variant	p.Pro2514fs/c.7541_7542delCT
UPN077_p	9	139397762	A	T	1796	0	1803	0.00 %	1314	190	1510	12.58 %	NOTCH1	ENST00000277541	missense_variant	p.Ile1680Asn/c.5039T>A

UPN077_p	6	41903730	TGGCTGCTGGAGCCCCGGGGGGCTTTGGGCGCTG	T	1720	0	1725	0.00 %	1464	149	1618	9.21 %	CCND3	ENST00000372991;ENST00000511642;ENST00000372987;ENST00000415497;ENST00000372988;ENST00000414200;ENST00000510503	disruptive_inframe_deletion	p.Pro265_Ser275del/c.794_826delCAGCGCCCAAAGCCCCCCGGGGCTCCAGCAGCC;p.Pro184_Ser194del/c.551_583delCAGCGCCCAAAGCCCCCCGGGGCTCCAGCAGCC;p.Pro215_Ser225del/c.644_676delCAGCGCCCAAAGCCCCCCGGGGCTCCAGCAGCC;p.Pro69_Ser79del/c.206_238delCAGCGCCCAAAGCCCCCCGGGGCTCCAGCAGCC;p.
UPN077_p	3	178936082	G	A	1318	0	1321	0.00 %	1081	27	1108	2.44 %	PIK3CA	ENST00000263967	missense_variant	p.Glu542Lys/c.1624G>A
UPN066_r	9	139391014	G	A	2182	0	2188	0.00 %	1145	1155	2302	50.17 %	NOTCH1	ENST00000277541	stop_gained	p.Gln2393*/c.7177C>T
UPN066_r	9	139399285	AGTAGGGGAAGA	A	1871	0	1872	0.00 %	958	532	1494	35.61 %	NOTCH1	ENST00000277541	inframe_deletion	p.His1611_Tyr1619del/c.4831_4857delCACGGCCAGCAGATGATCTTCCCCTAC
UPN066_r	5	67591144	ATA	ACCTACGGACGTGTC	1063	6	1069	0.56 %	497	246	747	32.93 %	PIK3R1	ENST00000396611;ENST00000521381;ENST00000521657;ENST00000274335;ENST00000320694;ENST00000336483;ENST00000523872	missense_variant+disruptive_inframe_insertion	p.Tyr580delinsProThrAspValSer/c.1738_1739delTAinsCCTACGGACGTGTC;p.Tyr280delinsProThrAspValSer/c.838_839delTAinsCCTACGGACGTGTC;p.Tyr310delinsProThrAspValSer/c.928_929delTAinsCCTACGGACGTGTC;p.Tyr217delinsProThrAspValSer/c.649_650delTAinsCCTACGGACGTG
UPN066_r	9	139399312	G	A	1912	0	1914	0.00 %	955	197	1156	17.04 %	NOTCH1	ENST00000277541	missense_variant	p.His1611Tyr/c.4831C>T
UPN066_r	5	67588178	AGATATCTCGAG	GAA	1345	9	1349	0.67 %	590	296	911	32.49 %	PIK3R1	ENST00000396611;ENST00000521381;ENST00000521657;ENST00000274335;ENST00000523807;ENST00000522084;ENST00000320694;ENST00000336483	missense_variant+disruptive_inframe_deletion+splice_region_variant	p.Asp337_Arg340delinsLys/c.1008_1019delAGATATCTCGAGinsGAA;p.Asp67_Arg70delinsLys/c.198_209delAGATATCTCGAGinsGAA;p.Asp37_Arg40delinsLys/c.108_119delAGATATCTCGAGinsGAA
UPN067_r	14	99640879	G	T	689	0	690	0.00 %	465	170	637	26.69 %	BCL11B	ENST00000357195;ENST00000345514;ENST00000443726	stop_gained	p.Ser765*/c.2294C>A;p.Ser694*/c.2081C>A;p.Ser571*/c.1712C>A
UPN067_r	19	4055148	C	T	2223	0	2228	0.00 %	1976	592	2573	23.01 %	ZBTB7A	ENST00000322357;ENST00000601588	missense_variant	p.Arg28Gln/c.83G>A
UPN067_r	9	139390655	C	CG	2154	0	2164	0.00 %	2419	510	2426	21.02 %	NOTCH1	ENST00000277541	frameshift_variant	p.Ser2513fs/c.7535dupC

UPN067_r	7	148525907	C	T	1532	1	1539	0.06 %	953	43	999	4.30 %	EZH2	ENST00000320356;ENST00000492143;ENST00000478654;ENST00000460911;ENST00000350995;ENST00000541220;ENST00000476773;ENST00000483967;ENST00000536783	missense_variant	p.Asp184Asn/c.550G>A;p.Asp175Asn/c.523G>A;p.Asp145Asn/c.433G>A;p.Asp75Asn/c.223G>A
UPN067_r	11	118375914	CA	C	2017	4	2028	0.20 %	1301	219	1523	14.38 %	KMT2A	ENST00000534358;ENST00000389506;ENST00000354520	frameshift_variant	p.Ile3105fs/c.9313delA;p.Ile3102fs/c.9304delA;p.Ile3064fs/c.9190delA
UPN067_r	9	139399389	A	G	2455	2	2463	0.08 %	2566	152	2720	5.59 %	NOTCH1	ENST00000277541	missense_variant	p.Leu1585Pro/c.4754T>C
UPN067_r	1	65303627	C	A	1337	1	1342	0.07 %	837	65	903	7.20 %	JAK1	ENST00000342505	missense_variant	p.Ser1043Ile/c.3128G>T
UPN067_r	12	49418606	C	T	2011	4	2019	0.20 %	1984	114	2101	5.43 %	KMT2D	ENST00000301067	missense_variant	p.Arg5303His/c.15908G>A
UPN067_r	2	47705471	C	G	1637	2	1642	0.12 %	714	168	884	19.00 %	MSH2	ENST00000233146;ENST00000543555;ENST00000406134	stop_gained	p.Tyr757*/c.2271C>G;p.Tyr691*/c.2073C>G
UPN067_r	1	216498688	C	G	1304	2	1309	0.15 %	288	40	329	12.16 %	USH2A	ENST00000366943;ENST00000307340;ENST00000366942	missense_variant	p.Gly368Arg/c.1102G>C
UPN067_r	12	49443666	AC	A	1752	7	1762	0.40 %	1801	292	2096	13.93 %	KMT2D	ENST00000301067	frameshift_variant	p.Gly1235fs/c.3704delG
UPN067_r	7	150706063	G	A	2156	0	2159	0.00 %	2438	169	2611	6.47 %	NOS3	ENST00000297494;ENST00000461406;ENST00000475017	missense_variant	p.Ala720Thr/c.2158G>A;p.Ala514Thr/c.1540G>A;p.Ala13Thr/c.37G>A
UPN067_r	10	104899222	C	T	1104	2	1106	0.18 %	577	121	698	17.34 %	NT5C2	ENST00000343289;ENST00000404739;ENST00000423468;ENST00000452156;ENST00000461461	missense_variant	p.Arg39Gln/c.116G>A;p.Arg10Gln/c.29G>A
UPN067_r	9	139397768	A	G	2213	0	2223	0.00 %	2351	463	2822	16.41 %	NOTCH1	ENST00000277541	missense_variant	p.Leu1678Pro/c.5033T>C
UPN067_r	X	133527949	C	T	711	2	715	0.28 %	422	63	485	12.99 %	PHF6	ENST00000394292;ENST00000370803;ENST00000332070;ENST00000370799;ENST00000416404;ENST00000370800	stop_gained	p.Arg129*/c.385C>T;p.Arg95*/c.283C>T
UPN067_r	9	139396938	C	T	829	0	831	0.00 %	607	52	661	7.87 %	NOTCH1	ENST00000277541	missense_variant+splice_region_variant	p.Glu1724Lys/c.5170G>A

UPN067_r	17	40474356	G	A	1778	0	1782	0.00 %	1192	91	1286	7.08 %	STAT3	ENST00000264657;ENST00000585517;ENST00000389272;ENST00000588969;ENST00000404395	missense_variant	p.Ala682Val/c.2045C>T;p.Ala584Val/c.1751C>T
UPN067_r	12	122248383	G	A	2401	3	2407	0.12 %	2497	170	2674	6.36 %	SETD1B	ENST00000604567;ENST00000542440;ENST00000267197	missense_variant	p.Arg511His/c.1532G>A
UPN067_r	12	122242800	C	T	1675	0	1680	0.00 %	1495	93	1590	5.85 %	SETD1B	ENST00000604567;ENST00000542440;ENST00000267197	stop_gained	p.Gln53*/c.157C>T
UPN067_r	6	135518167	A	T	2044	1	2052	0.05 %	1119	36	1156	3.11 %	MYB	ENST00000341911;ENST00000526187;ENST00000528015;ENST00000528774;ENST00000534121;ENST00000438901	missense_variant	p.Gln424His/c.1272A>T;p.Asn393Ile/c.1178A>T;p.Asn396Ile/c.1187A>T;p.Gln421His/c.1263A>T;p.Gln408His/c.1224A>T
UPN067_r	16	3777874	C	T	1951	0	1954	0.00 %	2211	43	2264	1.90 %	CREBBP	ENST00000262367;ENST00000382070	missense_variant	p.Ala2392Thr/c.7174G>A;p.Ala2354Thr/c.7060G>A
UPN067_r	9	139399350	C	G	2532	0	2535	0.00 %	2893	35	2935	1.19 %	NOTCH1	ENST00000277541	missense_variant	p.Arg1598Pro/c.4793G>C
UPN068_r	X	44942752	G	GTGTCGGATACCAGCCATT	461	0	461	0.00 %	408	253	414	61.11 %	KDM6A	ENST00000382899;ENST00000377967;ENST00000536777;ENST00000543216;ENST00000414389;ENST00000433797	disruptive_inframe_insertion	p.Val1119_Val1120insGlyTyrGlnProPheVal/c.3358_3359insGATACCAGCCATTTGTCG;p.Val1112_Val1113insGlyTyrGlnProPheVal/c.3337_3338insGATACCAGCCATTTGTCG;p.Val1067_Val1068insGlyTyrGlnProPheVal/c.3202_3203insGATACCAGCCATTTGTCG;p.Val1033_Val1034insGlyTyrGlnProPheVal/c.3100_3101insGATACCAGC
UPN068_r	4	153249384	C	T	1053	0	1055	0.00 %	743	637	1381	46.13 %	FBXW7	ENST00000281708;ENST00000296555;ENST00000263981;ENST00000603548;ENST00000393956;ENST00000603841	missense_variant	p.Arg465His/c.1394G>A;p.Arg347His/c.1040G>A;p.Arg385His/c.1154G>A;p.Arg289His/c.866G>A
UPN068_r	9	139397768	A	G	1399	1	1402	0.07 %	1006	789	1804	43.74 %	NOTCH1	ENST00000277541	missense_variant	p.Leu1678Pro/c.5033T>C
UPN069_r	4	153249385	G	A	1330	0	1336	0.00 %	401	313	715	43.78 %	FBXW7	ENST00000281708;ENST00000296555;ENST00000263981;ENST00000603548;ENST00000393956;ENST00000603841	missense_variant	p.Arg465Cys/c.1393C>T;p.Arg347Cys/c.1039C>T;p.Arg385Cys/c.1153C>T;p.Arg289Cys/c.865C>T

UPN069_r	4	153253787	C	A	1145	0	1145	0.00 %	304	206	511	40.31 %	FBXW7	ENST00000281708;ENST00000296555;ENST00000263981;ENST00000603548;ENST00000393956;ENST00000603841	stop_gained	p.Glu316*/c.946G>T;p.Glu198*/c.592G>T;p.Glu236*/c.706G>T;p.Glu140*/c.418G>T
UPN069_r	9	139399422	A	G	1774	2	1778	0.11 %	1482	26	1510	1.72 %	NOTCH1	ENST00000277541	missense_variant	p.Leu1574Pro/c.4721T>C
UPN069_r	9	139399328	GACC	G	1827	0	1831	0.00 %	802	559	1363	41.01 %	NOTCH1	ENST00000277541	disruptive_inframe_deletion	p.Val1605del/c.4812_4814delGGT
UPN069_r	16	9010890	ACTTTGTTAACTTTTTTGTT	GCA	1133	98	1223	8.01 %	468	112	593	18.89 %	USP7	ENST00000344836;ENST00000381886;ENST00000535863;ENST00000563085;ENST00000542333	frameshift_variant+missense_variant	p.Thr276fs/c.825_844delAACAAAAAAGTTAACAAAGTinsTGC;p.Thr260fs/c.777_796delAACAAAAAAGTTAACAAAGTinsTGC;p.Thr177fs/c.528_547delAACAAAAAAGTTAACAAAGTinsTGC;p.Thr218fs/c.651_670
UPN070_r	X	133527636	C	T	332	0	332	0.00 %	144	406	551	73.68 %	PHF6	ENST00000394292;ENST00000370803;ENST00000332070;ENST00000370799;ENST00000416404;ENST00000370800	stop_gained	p.Arg116*/c.346C>T;p.Arg82*/c.244C>T
UPN070_r	17	7577548	C	A	1204	1	1205	0.08 %	560	1335	1898	70.34 %	TP53	ENST00000269305;ENST00000413465;ENST00000359597;ENST00000420246;ENST00000455263;ENST00000445888;ENST00000509690;ENST00000514944	missense_variant	p.Gly245Cys/c.733G>T;p.Gly113Cys/c.337G>T;p.Gly152Cys/c.454G>T
UPN070_r	19	17945969	C	T	1253	3	1258	0.24 %	2074	1382	3464	39.90 %	JAK3	ENST00000458235;ENST00000527670;ENST00000534444	missense_variant	p.Arg657Gln/c.1970G>A
UPN070_r	17	40354460	A	T	645	0	648	0.00 %	821	672	1496	44.92 %	STAT5B	ENST00000293328	missense_variant	p.Val712Glu/c.2135T>A
UPN070_r	1	65310517	C	T	1080	0	1084	0.00 %	1564	925	2498	37.03 %	JAK1	ENST00000342505	missense_variant	p.Arg724His/c.2171G>A

UPN070_r	7	148525907	C	T	916	0	917	0.00 %	1558	31	1594	1.94 %	EZH2	ENST00000320356;ENST00000492143;ENST00000478654;ENST00000460911;ENST00000350995;ENST00000541220;ENST00000476773;ENST00000483967;ENST00000536783	missense_variant	p.Asp184Asn/c.550G>A;p.Asp175Asn/c.523G>A;p.Asp145Asn/c.433G>A;p.Asp75Asn/c.223G>A
UPN071_r	7	150700420	C	G	1219	1	1223	0.08 %	2083	44	2128	2.07 %	NOS3	ENST00000467517	missense_variant	p.Pro592Ala/c.1774C>G
UPN071_r	17	7577538	C	T	1869	0	1869	0.00 %	1284	792	2079	38.10 %	TP53	ENST00000269305;ENST00000413465;ENST00000359597;ENST00000420246;ENST00000455263;ENST00000445888;ENST00000509690;ENST00000514944	missense_variant	p.Arg248Gln/c.743G>A;p.Arg116Gln/c.347G>A;p.Arg155Gln/c.464G>A
UPN071_r	9	139399422	A	G	2008	0	2011	0.00 %	2182	569	2756	20.65 %	NOTCH1	ENST00000277541	missense_variant	p.Leu1574Pro/c.4721T>C
UPN071_r	7	148525907	C	T	1274	0	1279	0.00 %	1375	30	1407	2.13 %	EZH2	ENST00000320356;ENST00000492143;ENST00000478654;ENST00000460911;ENST00000350995;ENST00000541220;ENST00000476773;ENST00000483967;ENST00000536783	missense_variant	p.Asp184Asn/c.550G>A;p.Asp175Asn/c.523G>A;p.Asp145Asn/c.433G>A;p.Asp75Asn/c.223G>A
UPN071_r	4	106158199	C	A	1467	0	1471	0.00 %	1256	23	1282	1.79 %	TET2	ENST00000513237;ENST00000305737;ENST00000540549;ENST00000545826;ENST00000394764;ENST00000265149;ENST00000380013;ENST00000413648	missense_variant	p.Gln1055Lys/c.3163C>A;p.Gln1034Lys/c.3100C>A
UPN072_r	17	40359729	T	G	1314	2	1318	0.15 %	65	1183	1251	94.56 %	STAT5B	ENST00000293328	missense_variant	p.Asn642His/c.1924A>C
UPN072_r	9	139399408	GCAC	G	2277	13	2293	0.57 %	1562	1022	2584	39.55 %	NOTCH1	ENST00000277541	inframe_deletion	p.Val1578del/c.4732_4734delGTG

UPN072_r	4	153244091	C	T	1574	0	1578	0.00 %	1532	321	1860	17.26 %	FBXW7	ENST00000281708;ENST00000296555;ENST00000263981;ENST00000603548;ENST00000393956;ENST00000603841	missense_variant	p.Arg689Gln/c.2066G>A;p.Arg571Gln/c.1712G>A;p.Arg609Gln/c.1826G>A;p.Arg513Gln/c.1538G>A
UPN073_r	no data															
UPN074_r	no data															
UPN075_r	no data															
UPN076_r	no data															
UPN077_r	no data															

ID	Chr	Pos	Ref	Alt	Germline #REF	Germline #ALT	Germline DP	Germline VAF	Tumor #REF	Tumor #ALT	Tumor DP	Tumor VAF	Gene	ENST	Type	Variant
UPN078	4	153244210	C	T	1671	2	1675	0.12 %	496	491	988	49.70 %	FBXW7	ENST00000281708; ENST00000296555; ENST00000263981; ENST00000603548; ENST00000393956; ENST00000603841	stop_gained	p.Trp649*/c.1947G>A;p.Trp531*/c.1593G>A;p.Trp569*/c.1707G>A;p.Trp473*/c.1419G>A
UPN078	9	139399350	C	G	1822	0	1824	0.00 %	617	564	1182	47.72 %	NOTCH1	ENST00000277541	missense_variant	p.Arg1598Pro/c.4793G>C
UPN078	4	153249385	G	A	1795	1	1801	0.06 %	604	476	1085	43.87 %	FBXW7	ENST00000281708; ENST00000296555; ENST00000263981; ENST00000603548; ENST00000393956; ENST00000603841	missense_variant	p.Arg465Cys/c.1393C>T;p.Arg347Cys/c.1039C>T;p.Arg385Cys/c.1153C>T;p.Arg289Cys/c.865C>T
UPN078	1	115258747	C	A	1862	1	1864	0.05 %	834	307	1141	26.91 %	NRAS	ENST00000369535	missense_variant	p.Gly12Val/c.35G>T
UPN078	3	178936082	G	A	1496	0	1500	0.00 %	636	126	763	16.51 %	PIK3CA	ENST00000263967	missense_variant	p.Glu542Lys/c.1624G>A
UPN079	4	153247288	C	A	1749	2	1754	0.11 %	764	642	1410	45.53 %	FBXW7	ENST00000281708; ENST00000296555; ENST00000263981; ENST00000603548; ENST00000393956; ENST00000603841	missense_variant	p.Arg505Leu/c.1514G>T;p.Arg387Leu/c.1160G>T;p.Arg425Leu/c.1274G>T;p.Arg329Leu/c.986G>T
UPN079	9	139399394	CTCC	TCCCGGG	1864	6	1865	0.32 %	1141	748	1895	39.47 %	NOTCH1	ENST00000277541	missense_variant+disruptive_inframe_insertion	p.Glu1583delinsProGly/c.4746_4749delGGAGinsCCCGGGA
UPN080	9	139399422	A	G	2082	4	2090	0.19 %	2204	25	2236	1.12 %	NOTCH1	ENST00000277541	missense_variant	p.Leu1574Pro/c.4721T>C
UPN081	19	15308351	C	A	2064	3	2070	0.14 %	1220	20	1242	1.61 %	NOTCH3	ENST00000263388; ENST00000601011	missense_variant	p.Gly53Cys/c.157G>T;p.Gly52Cys/c.154G>T
UPN082	17	40370235	T	TG	1855	16	1865	0.86 %	859	140	868	16.13 %	STAT5B	ENST00000293328	frameshift_variant	p.Gln368fs/c.1102dupC
UPN082	19	15272336	C	CG	1848	5	1850	0.27 %	1292	169	1297	13.03 %	NOTCH3	ENST00000263388	frameshift_variant	p.Gly2035fs/c.6102dupC
UPN082	19	15276860	G	GC	2144	4	2150	0.19 %	1432	136	1437	9.46 %	NOTCH3	ENST00000263388	frameshift_variant	p.Ala1802fs/c.5404dupG
UPN082	12	49446165	A	AG	1665	2	1671	0.12 %	1779	138	1788	7.72 %	KMT2D	ENST00000301067	frameshift_variant	p.Leu434fs/c.1300dupC
UPN082	X	70356400	G	GC	874	0	874	0.00 %	842	60	843	7.12 %	MED12	ENST00000333646; ENST00000374102; ENST00000374080	frameshift_variant	p.Lys1768fs/c.5301dupC
UPN082	4	106180913	A	G	1471	2	1477	0.14 %	631	48	679	7.07 %	TET2	ENST00000513237; ENST00000540549; ENST00000380013	missense_variant	p.Asp1335Gly/c.4004A>G;p.Asp1314Gly/c.3941A>G
UPN082	7	150710878	C	T	1735	0	1740	0.00 %	441	30	472	6.36 %	NOS3	ENST00000297494; ENST00000461406	missense_variant	p.Arg1108Trp/c.3322C>T;p.Arg902Trp/c.2704C>T

UPN082	12	122263254	G	GC	1698	1	1707	0.06 %	723	46	725	6.34 %	SETD1B	ENST00000604567;<br>ENST00000542440;<br>ENST00000267197	frameshift_variant	p.Ala1776fs/c.5325dupC;p.Ala1733fs/c.5196dupC
UPN082	12	49441815	G	GC	2198	1	2200	0.05 %	897	57	900	6.33 %	KMT2D	ENST00000301067	frameshift_variant	p.Ala1390fs/c.4168dupG
UPN082	12	122260561	A	AC	2134	3	2143	0.14 %	1904	111	1939	5.72 %	SETD1B	ENST00000604567;<br>ENST00000542440;<br>ENST00000267197	frameshift_variant	p.His1362fs/c.4082dupC;p.His1319fs/c.3953dupC
UPN082	12	49434004	T	TG	2058	0	2069	0.00 %	674	38	679	5.60 %	KMT2D	ENST00000301067	frameshift_variant	p.Asn2517fs/c.7548dupC
UPN082	6	135520056	C	A	1216	2	1222	0.16 %	540	32	574	5.57 %	MYB	ENST00000341911;<br>ENST00000339290;<br>ENST00000367812;<br>ENST00000316528;<br>ENST00000442647;<br>ENST00000367814;<br>ENST00000527615;<br>ENST00000525369;<br>ENST00000525002;<br>ENST00000528774;<br>ENST00000533624;<br>ENST00000534044;<br>ENST00000534121;<br>ENST00000528343;<br>ENST00000438901;<br>ENST00000526889;<br>ENST00000531519	missense_variant	p.Thr526Asn/c.1577C>A;p.Thr405Asn/c.1214C>A;p.Thr402Asn/c.1205C>A;p.Thr320Asn/c.959C>A;p.Thr523Asn/c.1568C>A;p.Thr370Asn/c.1109C>A;p.Thr510Asn/c.1529C>A
UPN082	9	94493291	G	GC	2246	2	2254	0.09 %	1056	57	1056	5.40 %	ROR2	ENST00000375708;<br>ENST00000375715	frameshift_variant	p.His362fs/c.1083dupG;p.His222fs/c.663dupG
UPN082	11	118344185	A	AC	1902	6	1908	0.31 %	894	48	909	5.28 %	KMT2A	ENST00000534358;<br>ENST00000531904;<br>ENST00000389506;<br>ENST00000354520	frameshift_variant	p.Ser774fs/c.2318dupC;p.Ser807fs/c.2417dupC
UPN082	19	11097624	G	GC	2022	3	2023	0.15 %	1145	58	1148	5.05 %	SMARCA4	ENST00000358026;<br>ENST00000344626;<br>ENST00000429416;<br>ENST00000541122;<br>ENST00000589677;<br>ENST00000444061;<br>ENST00000590574;<br>ENST00000413806;<br>ENST00000450717	frameshift_variant	p.Gly271fs/c.810dupC

UPN082	19	54652155	G	GC	1650	0	1652	0.00 %	1693	81	1696	4.78 %	CNOT3	ENST00000221232; ENST00000358389; ENST00000406403; ENST00000440571	frameshift_variant	p.Ser392fs/c.1173dupC;p.Ser211fs/c.630dupC;p.Ser313fs/c.936dupC
UPN082	X	70357641	G	GC	856	2	858	0.23 %	884	39	888	4.39 %	MED12	ENST00000333646; ENST00000374102; ENST00000374080	frameshift_variant	p.Ser1970fs/c.5907dupC;p.Ser1966fs/c.5895dupC;p.Ser1967fs/c.5898dupC
UPN082	1	216062006	C	A	1906	0	1908	0.00 %	1021	43	1066	4.03 %	USH2A	ENST00000366943; ENST00000307340	missense_variant	p.Arg2662Ile/c.7985G>T
UPN082	19	15271746	T	TG	2016	2	2024	0.10 %	1325	48	1328	3.61 %	NOTCH3	ENST00000263388	frameshift_variant	p.Ala2233fs/c.6692dupC
UPN082	17	7579470	C	CG	1746	0	1750	0.00 %	3459	73	3478	2.10 %	TP53	ENST00000269305; ENST00000413465; ENST00000359597; ENST00000420246; ENST00000455263; ENST00000445888; ENST00000508793; ENST00000604348; ENST00000503591	frameshift_variant	p.Val73fs/c.216dupC
UPN082	19	11106968	A	T	2031	0	2035	0.00 %	2767	37	2820	1.31 %	SMARCA4	ENST00000358026; ENST00000344626; ENST00000429416; ENST00000541122; ENST00000589677; ENST00000444061; ENST00000590574; ENST00000413806; ENST00000450717	missense_variant	p.Asp558Val/c.1673A>T
UPN083	9	139399344	A	G	2105	1	2108	0.05 %	1702	1367	3075	44.46 %	NOTCH1	ENST00000277541	missense_variant	p.Leu1600Pro/c.4799T>C
UPN083	X	1321382	C	T	1740	2	1745	0.11 %	764	568	1333	42.61 %	CRLF2	ENST00000381567; ENST00000400841; ENST00000381566	missense_variant	p.Val125Met/c.373G>A
UPN083	5	35874572	T	TAACCTTCGAGGCAGAGTG	1446	0	1447	0.00 %	1860	293	1875	15.63 %	IL7R	ENST00000303115	inframe_insertion	p.Thr244_Ile245insPheGluAlaGluCysGlyGluProThr/c.732_733insTTCGAGGC
UPN084	10	89717699	G	T	1116	0	1119	0.00 %	9	188	197	95.43 %	PTEN	ENST00000371953	stop_gained	p.Glu242*/c.724G>T
UPN084	10	89717717	C	A	1166	1	1168	0.09 %	11	175	186	94.09 %	PTEN	ENST00000371953	missense_variant	p.Pro248Thr/c.742C>A

UPN084	X	133547983	A	G	824	2	832	0.24 %	94	184	280	65.71 %	PHF6	ENST00000394292; ENST00000370803; ENST00000332070; ENST00000370799; ENST00000416404; ENST00000370800	missense_variant	p.His240Arg/c.719A>G;p.His239Arg/c.716A>G;p.His205Arg/c.614A>G
UPN084	4	153247366	C	A	1221	1	1223	0.08 %	296	285	581	49.05 %	FBXW7	ENST00000281708; ENST00000296555; ENST00000263981; ENST00000603548; ENST00000393956; ENST00000603841	missense_variant	p.Arg479Leu/c.1436G>T;p.Arg361Leu/c.1082G>T;p.Arg399Leu/c.1196G>T;p.Arg303Leu/c.908G>T
UPN084	4	106155520	G	A	1172	3	1177	0.25 %	225	211	437	48.28 %	TET2	ENST00000513237; ENST00000305737; ENST00000540549; ENST00000545826; ENST00000394764; ENST00000265149; ENST00000380013; ENST00000413648	missense_variant	p.Val162Ile/c.484G>A;p.Val141Ile/c.421G>A
UPN084	19	15284952	C	A	1337	0	1338	0.00 %	784	709	1493	47.49 %	NOTCH3	ENST00000263388	missense_variant	p.Val1555Phe/c.4663G>T
UPN085	4	153244238	C	T	1360	0	1361	0.00 %	16	1300	1322	98.34 %	FBXW7	ENST00000281708; ENST00000296555; ENST00000263981; ENST00000603548; ENST00000393956; ENST00000603841	missense_variant	p.Ser640Asn/c.1919G>A;p.Ser522Asn/c.1565G>A;p.Ser560Asn/c.1679G>A;p.Ser464Asn/c.1391G>A
UPN085	X	133527938	G	A	1095	0	1100	0.00 %	516	487	1006	48.41 %	PHF6	ENST00000394292; ENST00000370803; ENST00000332070; ENST00000370799; ENST00000416404; ENST00000370800	splice_acceptor_variant+intron_variant	c.375-1G>A;c.273-1G>A
UPN085	1	115258747	C	A	1434	1	1437	0.07 %	866	723	1595	45.33 %	NRAS	ENST00000369535	missense_variant	p.Gly12Val/c.35G>T
UPN085	9	139399367	G	GGGA	1924	0	1927	0.00 %	2424	966	2429	39.77 %	NOTCH1	ENST00000277541	disruptive_inframe_insertion	p.Phe1592_Leu1593insPro/c.4775_4776insTCC
UPN085	19	15281488	C	T	1152	0	1157	0.00 %	976	44	1025	4.29 %	NOTCH3	ENST00000263388	missense_variant	p.Val1629Met/c.4885G>A
UPN085	9	139397703	C	G	1866	0	1870	0.00 %	2121	43	2167	1.98 %	NOTCH1	ENST00000277541	missense_variant	p.Ala1700Pro/c.5098G>C

UPN086	X	133551269	A	G	651	1	655	0.15 %	42	771	814	94.72 %	PHF6	ENST00000394292; ENST00000370803; ENST00000332070; ENST00000370799; ENST00000416404	missense_variant	p.His303Arg/c.908A>G;p.His302Arg/c.905A>G;p.His268Arg/c.803A>G
UPN086	17	7579415	C	T	1229	0	1231	0.00 %	24	384	408	94.12 %	TP53	ENST00000269305; ENST00000413465; ENST00000359597; ENST00000420246; ENST00000455263; ENST00000445888; ENST00000508793; ENST00000604348; ENST00000503591	stop_gained	p.Trp91*/c.272G>A
UPN086	14	99724135	CGTCTTCT	GGGATG	1460	6	1465	0.41 %	729	461	1210	38.10 %	BCL11B	ENST00000357195; ENST00000345514	frameshift_variant+missense_variant	p.Glu31fs/c.93_100delAGAAGACGinsCATCCC
UPN086	9	139399408	GCAC	G	1603	5	1612	0.31 %	809	518	1331	38.92 %	NOTCH1	ENST00000277541	inframe_deletion	p.Val1578del/c.4732_4734delGTG
UPN086	4	153244138	C	T	1568	0	1570	0.00 %	2173	52	2227	2.33 %	FBXW7	ENST00000281708; ENST00000296555; ENST00000263981; ENST00000603548; ENST00000393956; ENST00000603841	stop_gained	p.Trp673*/c.2019G>A;p.Trp555*/c.1665G>A;p.Trp593*/c.1779G>A;p.Trp497*/c.1491G>A
UPN087	16	3786705	C	G	1464	0	1466	0.00 %	602	506	1110	45.59 %	CREBBP	ENST00000262367; ENST00000382070	missense_variant	p.Trp1502Cys/c.4506G>C;p.Trp1464Cys/c.4392G>C
UPN087	4	106164934	GAGTAAGTGAAGCCCAGGGC	G	1218	0	1220	0.00 %	782	263	1062	24.76 %	TET2	ENST00000513237; ENST00000540549; ENST00000380013; ENST00000545826; ENST00000265149	frameshift_variant+splice_donor_variant+splice_region_variant+intron_variant	p.Glu1289fs/c.3866_3866+18delAGTAAGTGAAGCCCAGGGC;p.Glu1268fs/c.3803_3803+18delAGTAAGTGAAGCCCAGGGC;c.*127_*127+18delAGTAAGTGAAGCCCAGGGC
UPN088	12	25398285	C	G	1040	209	1249	######	797	605	1404	43.09 %	KRAS	ENST00000256078; ENST00000311936; ENST00000557334; ENST00000556131	missense_variant	p.Gly12Arg/c.34G>C
UPN089	X	133551265	T	TA	660	0	662	0.00 %	500	91	501	18.16 %	PHF6	ENST00000394292; ENST00000370803; ENST00000332070; ENST00000370799; ENST00000416404	frameshift_variant+stop_gained	p.Tyr302fs/c.905dupA;p.Tyr301fs/c.902dupA;p.Tyr267fs/c.800dupA
UPN089	9	139399344	A	G	3187	2	3193	0.06 %	1732	201	1940	10.36 %	NOTCH1	ENST00000277541	missense_variant	p.Leu1600Pro/c.4799T>C

UPN089	6	41903805	AGTG	ATTTTCTAATTGGGCGGC	3041	13	3051	0.43 %	1562	105	1671	6.28 %	CCND3	ENST00000372991; ENST00000511642; ENST00000372987; ENST00000415497; ENST00000372988; ENST00000414200; ENST00000510503	frameshift_variant+missense_variant	p.Ala250fs/c.749_751delCACinsGCCGCCCAATTAGAAAA;p.Ala169fs/c.506_508delCACinsGCCGCCCAATTAGAAAA;p.Ala200fs/c.599_601delCACinsGCCGCCCAATTAGAAAA;p.Ala54fs/c.161_163delCACinsGCCGCCCAATTAGAAAA;p.Ala178fs/c.533_535delCACinsGCCGCCC
UPN089	14	99640660	T	C	2586	0	2599	0.00 %	1515	78	1597	4.88 %	BCL11B	ENST00000357195; ENST00000345514; ENST00000443726	missense_variant	p.Lys838Arg/c.2513A>G;p.Lys767Arg/c.2300A>G;p.Lys644Arg/c.1931A>G
UPN090	9	139399344	A	G	2053	0	2063	0.00 %	576	277	854	32.44 %	NOTCH1	ENST00000277541	missense_variant	p.Leu1600Pro/c.4799T>C
UPN090	17	40461409	C	T	1262	0	1265	0.00 %	667	221	892	24.78 %	STAT5A	ENST00000345506; ENST00000590949; ENST00000546010; ENST00000452307; ENST00000588868; ENST00000587646	missense_variant	p.Ser710Phe/c.2129C>T;p.Ser680Phe/c.2039C>T;p.Ser707Phe/c.2120C>T;p.Ser679Phe/c.2036C>T;p.Ser198Phe/c.593C>T
UPN090	12	49418456	GAATA	G	1709	0	1716	0.00 %	1052	342	1396	24.50 %	KMT2D	ENST00000301067	frameshift_variant	p.Leu5318fs/c.15953_15956delTATT
UPN090	17	30320905	A	G	752	0	753	0.00 %	534	163	698	23.35 %	SUZ12	ENST00000322652; ENST00000580398	missense_variant	p.Arg439Gly/c.1315A>G;p.Arg416Gly/c.1246A>G
UPN090	19	17943636	T	TCGAAGATTGGAC	2068	0	2074	0.00 %	1066	176	1072	16.42 %	JAK3	ENST00000458235; ENST00000527670; ENST00000534444	inframe_insertion	p.Phe817_Glu818insGlyProIlePhe/c.2452_2453insGTCCAATCTTCG
UPN091	X	133551211	T	C	441	0	441	0.00 %	18	308	327	94.19 %	PHF6	ENST00000394292; ENST00000370803; ENST00000332070; ENST00000370799; ENST00000416404	missense_variant	p.Cys284Arg/c.850T>C;p.Cys283Arg/c.847T>C;p.Cys249Arg/c.745T>C
UPN091	4	153247289	G	A	1342	0	1344	0.00 %	717	632	1352	46.75 %	FBXW7	ENST00000281708; ENST00000296555; ENST00000263981; ENST00000603548; ENST00000393956; ENST00000603841	missense_variant	p.Arg505Cys/c.1513C>T;p.Arg387Cys/c.1159C>T;p.Arg425Cys/c.1273C>T;p.Arg329Cys/c.985C>T
UPN091	16	9014234	TC	T	1250	0	1252	0.00 %	431	375	807	46.47 %	USP7	ENST00000344836; ENST00000381886; ENST00000535863; ENST00000563085; ENST00000542333	frameshift_variant	p.Asp198fs/c.592delG;p.Asp182fs/c.544delG;p.Asp99fs/c.295delG;p.Asp140fs/c.418delG

UPN091	4	153247376	T	C	1014	2	1016	0.20 %	509	391	902	43.35 %	FBXW7	ENST00000281708; ENST00000296555; ENST00000263981; ENST00000603548; ENST00000393956; ENST00000603841	missense_variant	p.Ser476Gly/c.1426A>G;p.Ser358Gly/c.1072A>G;p.Ser396Gly/c.1186A>G;p.Ser300Gly/c.898A>G
UPN091	9	139397723	AAGCACTGCG	A	1962	0	1963	0.00 %	1024	96	1121	8.56 %	NOTCH1	ENST00000277541	disruptive_inframe_deletion	p.Ser1690_Cys1692del/c.5069_5077delCGCAGTGCT
UPN091	9	139396907	A	AGCTGCGCCGGCGGGGGC	1121	0	1122	0.00 %	589	21	593	3.54 %	NOTCH1	ENST00000277541	inframe_insertion	p.Gln1733_Leu1734insGlnLeuGluProProProProAlaGln/c.5200_5201insAATT
UPN092	19	17949108	C	A	1214	13	1228	1.06 %	109	815	925	88.11 %	JAK3	ENST00000458235; ENST00000527670; ENST00000534444	missense_variant	p.Met511Ile/c.1533G>T
UPN092	19	17948006	G	A	1165	23	1190	1.93 %	134	865	1000	86.50 %	JAK3	ENST00000458235; ENST00000527670; ENST00000534444	missense_variant	p.Ala573Val/c.1718C>T
UPN092	14	99737545	C	G	786	2	792	0.25 %	662	562	1229	45.73 %	BCL11B	ENST00000357195; ENST00000345514; ENST00000443726	missense_variant	p.Arg4Pro/c.11G>C
UPN092	X	70345251	GGT	G	1192	9	1205	0.75 %	859	626	1487	42.10 %	MED12	ENST00000333646; ENST00000374102; ENST00000374080	frameshift_variant	p.Val760fs/c.2279_2280delTG
UPN093	X	133547940	C	T	615	2	617	0.32 %	5	523	528	99.05 %	PHF6	ENST00000394292; ENST00000370803; ENST00000332070; ENST00000370799; ENST00000416404; ENST00000370800	stop_gained	p.Arg226*/c.676C>T;p.Arg225*/c.673C>T;p.Arg191*/c.571C>T
UPN093	9	139399365	A	G	1889	1	1894	0.05 %	828	709	1539	46.07 %	NOTCH1	ENST00000277541	missense_variant	p.Leu1593Pro/c.4778T>C
UPN093	1	216138812	G	A	1043	0	1044	0.00 %	527	408	938	43.50 %	USH2A	ENST00000366943; ENST00000307340	stop_gained	p.Arg2323*/c.6967C>T
UPN093	9	139390777	G	GAGGTTCCC	1715	0	1720	0.00 %	1240	412	1240	33.23 %	NOTCH1	ENST00000277541	frameshift_variant	p.Leu2472fs/c.7413_7414insGGGAAC
UPN093	9	139399344	A	G	1940	0	1943	0.00 %	1563	34	1600	2.13 %	NOTCH1	ENST00000277541	missense_variant	p.Leu1600Pro/c.4799T>C
UPN094	4	153249510	C	A	1720	0	1724	0.00 %	1801	98	1903	5.15 %	FBXW7	ENST00000281708; ENST00000296555; ENST00000263981; ENST00000603548; ENST00000393956; ENST00000603841	missense_variant	p.Gly423Val/c.1268G>T;p.Gly305Val/c.914G>T;p.Gly343Val/c.1028G>T;p.Gly247Val/c.740G>T

UPN094	19	17943438	A	G	2115	1	2119	0.05 %	1923	84	2010	4.18 %	JAK3	ENST00000458235; ENST00000527670; ENST00000534444	missense_variant	p.Leu857Pro/c.2570T>C
UPN094	1	65304236	G	T	1675	3	1680	0.18 %	1687	47	1736	2.71 %	JAK1	ENST00000342505	missense_variant	p.Pro960His/c.2879C>A
UPN094	9	139397762	A	T	2077	2	2083	0.10 %	2149	33	2186	1.51 %	NOTCH1	ENST00000277541	missense_variant	p.Ile1680Asn/c.5039T>A
UPN095	X	133527595	C	CAT	842	0	845	0.00 %	695	515	701	73.47 %	PHF6	ENST00000394292; ENST00000370803; ENST00000332070; ENST00000370799; ENST00000416404; ENST00000370800	frameshift_variant	p.His104fs/c.307_308dupTA;p.His70fs /c.205_206dupTA
UPN095	5	35867418	G	A	1633	4	1639	0.24 %	851	840	1694	49.59 %	IL7R	ENST00000303115; ENST00000514217; ENST00000343305; ENST00000506850; ENST00000511982	missense_variant	p.Val78Met/c.232G>A
UPN095	17	40457661	G	A	1794	0	1796	0.00 %	332	279	612	45.59 %	STAT5A	ENST00000345506; ENST00000590949; ENST00000546010; ENST00000452307	missense_variant	p.Gly472Ser/c.1414G>A;p.Gly442Ser/ c.1324G>A
UPN095	9	139397768	A	G	2079	0	2084	0.00 %	403	332	738	44.99 %	NOTCH1	ENST00000277541	missense_variant	p.Leu1678Pro/c.5033T>C
UPN095	13	77641982	T	G	1550	0	1554	0.00 %	623	465	1088	42.74 %	MYCBP2	ENST00000407578; ENST00000544440; ENST00000357337; ENST00000429715	missense_variant	p.Arg4063Ser/c.12189A>C;p.Arg4025 Ser/c.12075A>C;p.Arg445Ser/c.1335A >C
UPN096	X	44942747	TGTGCGT GTCGTAT C	GGGGGGCAA ACGG	548	4	548	0.73 %	9	253	269	94.05 %	KDM6A	ENST00000382899; ENST00000377967; ENST00000536777; ENST00000543216; ENST00000414389; ENST00000433797	frameshift_variant+m issense_variant	p.Phe1116fs/c.3348_3362delTGTGCG TGTCGTATCinsGGGGGGCAAACGG;p.P he1109fs/c.3327_3341delTGTGCGTG TCGTATCinsGGGGGGCAAACGG;p.Phe 1064fs/c.3192_3206delTGTGCGTGTC GTATCinsGGGGGGCAAACGG;p.Phe10 30fs/c.3090_3104delTGTGCGTGTCGT
UPN096	X	41205653	T	A	585	0	586	0.00 %	160	454	614	73.94 %	DDX3X	ENST00000399959; ENST00000457138	missense_variant	p.Val496Glu/c.1487T>A;p.Val480Glu/ c.1439T>A
UPN096	9	139391011	G	A	2005	0	2008	0.00 %	1058	1067	2128	50.14 %	NOTCH1	ENST00000277541	stop_gained	p.Gln2394*/c.7180C>T
UPN096	9	139399365	A	G	1927	1	1939	0.05 %	1046	980	2030	48.28 %	NOTCH1	ENST00000277541	missense_variant	p.Leu1593Pro/c.4778T>C

UPN096	19	10904475	G	A	1421	2	1427	0.14 %	715	643	1365	47.11 %	DNM2	ENST00000314646; ENST00000585892; ENST00000359692; ENST00000389253; ENST00000355667; ENST00000408974; ENST00000587830	missense_variant	p.Gly358Arg/c.1072G>A;p.Gly110Arg/c.328G>A
UPN096	X	41206199	C	T	628	0	630	0.00 %	655	72	727	9.90 %	DDX3X	ENST00000399959; ENST00000457138	missense_variant	p.Pro568Leu/c.1703C>T;p.Pro552Leu/c.1655C>T
UPN097	19	17949108	C	T	1489	0	1491	0.00 %	919	673	1599	42.09 %	JAK3	ENST00000458235; ENST00000527670; ENST00000534444	missense_variant	p.Met511Ile/c.1533G>A
UPN097	9	139399283	GTAGTAGGGGAAGATCATC	AAGAG	1541	16	1557	1.03 %	1655	676	2347	28.80 %	NOTCH1	ENST00000277541	missense_variant+disruptive_inframe_deletion	p.Gln1614_Tyr1620delinsProLeu/c.4841_4860delAGATGATCTTCCCCTACTACinsCTCTT
UPN097	1	65311203	C	A	1292	1	1294	0.08 %	1433	543	1979	27.44 %	JAK1	ENST00000342505	missense_variant	p.Ser703Ile/c.2108G>T
UPN097	21	36252987	T	TGGAACAGGGCC	1269	0	1271	0.00 %	1515	326	1521	21.43 %	RUNX1	ENST00000300305; ENST00000344691; ENST00000325074; ENST00000437180; ENST00000482318; ENST00000399240; ENST00000358356; ENST00000399237; ENST00000486278; ENST00000455571	frameshift_variant	p.Asp126fs/c.374_375insGGCCCTGTTCC;p.Asp99fs/c.293_294insGGCCCTGTTCC;p.Asp114fs/c.338_339insGGCCCTGTTCC;p.Arg28fs/c.81_82insGGCCCTGTTCC;p.Asp102fs/c.302_303insGGCCCTGTTCC;p.Asp113fs/c.335_336insGGCCCTGTTCC
UPN098	9	94495617	G	A	1776	2	1781	0.11 %	223	199	422	47.16 %	ROR2	ENST00000375708; ENST00000375715	missense_variant	p.Arg242Cys/c.724C>T;p.Arg102Cys/c.304C>T
UPN098	7	148526829	C	A	750	3	756	0.40 %	115	81	197	41.12 %	EZH2	ENST00000320356; ENST00000492143; ENST00000478654; ENST00000460911; ENST00000350995; ENST00000541220; ENST00000476773; ENST00000483967; ENST00000536783	missense_variant	p.Gly159Trp/c.475G>T;p.Gly150Trp/c.448G>T;p.Gly120Trp/c.358G>T;p.Gly50Trp/c.148G>T
UPN098	14	99641282	C	T	294	0	295	0.00 %	114	32	147	21.77 %	BCL11B	ENST00000357195; ENST00000345514; ENST00000443726	missense_variant	p.Gly631Arg/c.1891G>A;p.Gly560Arg/c.1678G>A;p.Gly437Arg/c.1309G>A

UPN098	7	148515169	C	T	1018	1	1021	0.10 %	274	75	351	21.37 %	EZH2	ENST00000320356; ENST00000478654; ENST00000460911; ENST00000350995; ENST00000541220; ENST00000476773; ENST00000483967	missense_variant	p.Arg347Gln/c.1040G>A;p.Arg333Gln/c.998G>A;p.Arg342Gln/c.1025G>A;p.Arg303Gln/c.908G>A
UPN098	7	50444276	G	A	1078	0	1085	0.00 %	359	88	449	19.60 %	IKZF1	ENST00000331340; ENST00000359197; ENST00000440768; ENST00000349824; ENST00000357364; ENST00000439701	missense_variant	p.Arg69His/c.206G>A
UPN098	4	153247289	G	A	1230	0	1231	0.00 %	357	86	447	19.24 %	FBXW7	ENST00000281708; ENST00000296555; ENST00000263981; ENST00000603548; ENST00000393956; ENST00000603841	missense_variant	p.Arg505Cys/c.1513C>T;p.Arg387Cys/c.1159C>T;p.Arg425Cys/c.1273C>T;p.Arg329Cys/c.985C>T
UPN098	4	153244091	C	T	1031	0	1034	0.00 %	261	53	314	16.88 %	FBXW7	ENST00000281708; ENST00000296555; ENST00000263981; ENST00000603548; ENST00000393956; ENST00000603841	missense_variant	p.Arg689Gln/c.2066G>A;p.Arg571Gln/c.1712G>A;p.Arg609Gln/c.1826G>A;p.Arg513Gln/c.1538G>A
UPN098	1	216372980	G	A	806	1	808	0.12 %	161	31	192	16.15 %	USH2A	ENST00000366943; ENST00000307340; ENST00000366942	missense_variant	p.Ala1267Val/c.3800C>T
UPN098	19	4054414	C	T	367	1	370	0.27 %	115	22	137	16.06 %	ZBTB7A	ENST00000322357; ENST00000601588	missense_variant	p.Gly273Ser/c.817G>A
UPN098	4	153258982	C	T	858	0	858	0.00 %	252	46	298	15.44 %	FBXW7	ENST00000281708; ENST00000296555; ENST00000263981; ENST00000603548; ENST00000393956; ENST00000603841	missense_variant	p.Arg278Gln/c.833G>A;p.Arg160Gln/c.479G>A;p.Arg198Gln/c.593G>A;p.Arg102Gln/c.305G>A

UPN098	4	153249384	C	T	1014	0	1017	0.00 %	337	61	398	15.33 %	FBXW7	ENST00000281708; ENST00000296555; ENST00000263981; ENST00000603548; ENST00000393956; ENST00000603841	missense_variant	p.Arg465His/c.1394G>A;p.Arg347His/c.1040G>A;p.Arg385His/c.1154G>A;p.Arg289His/c.866G>A
UPN098	4	55561756	G	A	1238	0	1240	0.00 %	364	64	428	14.95 %	KIT	ENST00000288135; ENST00000412167	missense_variant	p.Arg49His/c.146G>A
UPN098	7	148514471	C	T	673	0	679	0.00 %	246	36	284	12.68 %	EZH2	ENST00000320356; ENST00000478654; ENST00000460911; ENST00000350995; ENST00000541220; ENST00000476773; ENST00000483967	missense_variant	p.Arg418Gln/c.1253G>A;p.Arg404Gln/c.1211G>A;p.Arg413Gln/c.1238G>A;p.Arg374Gln/c.1121G>A
UPN098	9	139399296	A	T	1428	0	1436	0.00 %	594	69	664	10.39 %	NOTCH1	ENST00000277541	missense_variant	p.Ile1616Asn/c.4847T>A
UPN098	9	139390636	G	A	1371	0	1374	0.00 %	465	49	514	9.53 %	NOTCH1	ENST00000277541	stop_gained	p.Gln2519*/c.7555C>T
UPN098	7	150708877	C	G	816	2	820	0.24 %	466	44	513	8.58 %	NOS3	ENST00000475017	missense_variant+splice_region_variant	p.Leu289Val/c.865C>G
UPN098	7	150700420	C	G	841	0	842	0.00 %	716	53	777	6.82 %	NOS3	ENST00000467517	missense_variant	p.Pro592Ala/c.1774C>G
UPN098	19	4054876	C	T	1462	1	1465	0.07 %	373	25	400	6.25 %	ZBTB7A	ENST00000322357; ENST00000601588	missense_variant	p.Val119Met/c.355G>A
UPN098	6	135518167	A	T	1237	0	1243	0.00 %	460	26	487	5.34 %	MYB	ENST00000341911; ENST00000526187; ENST00000528015; ENST00000528774; ENST00000534121; ENST00000438901	missense_variant	p.Gln424His/c.1272A>T;p.Asn393Ile/c.1178A>T;p.Asn396Ile/c.1187A>T;p.Gln421His/c.1263A>T;p.Gln408His/c.1224A>T
UPN098	7	150700345	T	C	780	0	786	0.00 %	893	32	925	3.46 %	NOS3	ENST00000484524	missense_variant	p.Val620Ala/c.1859T>C
UPN099	X	133511726	G	GA	921	0	922	0.00 %	844	771	852	90.49 %	PHF6	ENST00000394292; ENST00000370803; ENST00000332070; ENST00000370799; ENST00000416404; ENST00000370800	frameshift_variant	p.Cys28fs/c.81dupA
UPN099	12	49424759	C	T	2659	0	2664	0.00 %	570	539	1113	48.43 %	KMT2D	ENST00000301067	missense_variant	p.Asp4530Asn/c.13588G>A

UPN099	21	36252877	C	T	1760	0	1763	0.00 %	1029	923	1957	47.16 %	RUNX1	ENST00000300305; ENST00000344691; ENST00000325074; ENST00000437180; ENST00000399240; ENST00000358356; ENST00000399237; ENST00000486278	missense_variant	p.Arg162Lys/c.485G>A;p.Arg135Lys/c.404G>A;p.Arg150Lys/c.449G>A;p.Arg138Lys/c.413G>A
UPN099	19	10904498	CT	C	1502	0	1506	0.00 %	349	303	653	46.40 %	DNM2	ENST00000314646; ENST00000585892; ENST00000359692; ENST00000389253; ENST00000355667; ENST00000408974; ENST00000587830	frameshift_variant	p.Phe366fs/c.1097delT;p.Phe118fs/c.353delT
UPN099	11	118339537	AA	GGACCGCCC	1846	6	1852	0.32 %	577	385	965	39.90 %	KMT2A	ENST00000534358; ENST00000531904; ENST00000389506; ENST00000354520; ENST00000527869; ENST00000533790	frameshift_variant+missense_variant	p.Ser161fs/c.480_481delAAinsGGACCGCCC;p.Ser194fs/c.579_580delAAinsGGACCGCCC;p.Ser111fs/c.330_331delAAinsGGACCGCCC;p.Ser78fs/c.231_232delAAinsGGACCGCCC
UPN099	9	139397675	A	G	2872	2	2878	0.07 %	842	347	1190	29.16 %	NOTCH1	ENST00000277541	missense_variant	p.Leu1709Pro/c.5126T>C
UPN099	9	139399296	A	C	2728	3	2735	0.11 %	1146	80	1228	6.51 %	NOTCH1	ENST00000277541	missense_variant	p.Ile1616Ser/c.4847T>G
UPN099	X	44942755	T	TCAATTATCCCACC	682	0	682	0.00 %	658	39	659	5.92 %	KDM6A	ENST00000382899; ENST00000377967; ENST00000536777; ENST00000543216; ENST00000414389; ENST00000433797	frameshift_variant	p.Val1120fs/c.3357_3358insAATTATCCCACCC;p.Val1113fs/c.3336_3337insAATTATCCCACCC;p.Val1068fs/c.3201_3202insAATTATCCCACCC;p.Val1034fs/c.3099_3100insAATTATCCCACCC;p.Val710fs/c.2127_2128insAATTATCCC
UPN099	9	139397747	C	A	2900	0	2905	0.00 %	1200	72	1272	5.66 %	NOTCH1	ENST00000277541	missense_variant	p.Cys1685Phe/c.5054G>T
UPN099	9	139399891	G	A	2560	0	2564	0.00 %	1197	26	1225	2.12 %	NOTCH1	ENST00000277541	missense_variant	p.Pro1486Leu/c.4457C>T
UPN099	4	153249385	G	A	2030	4	2037	0.20 %	2071	44	2120	2.08 %	FBXW7	ENST00000281708; ENST00000296555; ENST00000263981; ENST00000603548; ENST00000393956; ENST00000603841	missense_variant	p.Arg465Cys/c.1393C>T;p.Arg347Cys/c.1039C>T;p.Arg385Cys/c.1153C>T;p.Arg289Cys/c.865C>T
UPN100	17	40359729	T	G	1188	0	1191	0.00 %	33	552	585	94.36 %	STAT5B	ENST00000293328	missense_variant	p.Asn642His/c.1924A>C
UPN100	9	139390746	AG	A	2203	0	2205	0.00 %	567	460	1029	44.70 %	NOTCH1	ENST00000277541	frameshift_variant	p.Leu2482fs/c.7444delC

UPN100	19	17945918	A	G	2432	1	2436	0.04 %	531	338	872	38.76 %	JAK3	ENST00000458235; ENST00000527670; ENST00000534444	missense_variant	p.Val674Ala/c.2021T>C
UPN100	1	65305431	G	T	1956	1	1963	0.05 %	814	132	948	13.92 %	JAK1	ENST00000342505	missense_variant	p.Asp899Glu/c.2697C>A
UPN100	1	9787030	G	A	1805	2	1809	0.11 %	775	124	901	13.76 %	PIK3CD	ENST00000361110; ENST00000536656; ENST00000377346	missense_variant	p.Glu1045Lys/c.3133G>A;p.Glu1021Lys/c.3061G>A
UPN100	17	40354811	C	A	2142	1	2143	0.05 %	888	86	975	8.82 %	STAT5B	ENST00000293328	missense_variant	p.Gly698Val/c.2093G>T
UPN101	17	40359729	T	G	1351	1	1353	0.07 %	72	426	501	85.03 %	STAT5B	ENST00000293328	missense_variant	p.Asn642His/c.1924A>C
UPN101	19	17945969	C	T	2707	2	2713	0.07 %	647	407	1055	38.58 %	JAK3	ENST00000458235; ENST00000527670; ENST00000534444	missense_variant	p.Arg657Gln/c.1970G>A
UPN101	9	139391029	G	A	2824	0	2827	0.00 %	691	395	1089	36.27 %	NOTCH1	ENST00000277541	stop_gained	p.Gln2388*/c.7162C>T
UPN102	4	153249400	G	T	1209	1	1210	0.08 %	74	981	1059	92.63 %	FBXW7	ENST00000281708; ENST00000296555; ENST00000263981; ENST00000603548; ENST00000393956; ENST00000603841	missense_variant	p.His460Asn/c.1378C>A;p.His342Asn/c.1024C>A;p.His380Asn/c.1138C>A;p.His284Asn/c.850C>A
UPN102	16	8988661	G	GGCTC	1417	0	1419	0.00 %	1950	869	1966	44.20 %	USP7	ENST00000344836; ENST00000381886; ENST00000535863	frameshift_variant	p.Gln1031fs/c.3090_3091insGAGC;p.Gln1015fs/c.3042_3043insGAGC;p.Gln932fs/c.2793_2794insGAGC
UPN102	9	139399410	A	T	1606	0	1612	0.00 %	2894	1348	4252	31.70 %	NOTCH1	ENST00000277541	missense_variant	p.Val1578Glu/c.4733T>A
UPN103	17	40359729	T	G	1226	2	1231	0.16 %	19	1287	1307	98.47 %	STAT5B	ENST00000293328	missense_variant	p.Asn642His/c.1924A>C
UPN103	9	139399416	A	T	2622	0	2627	0.00 %	1175	1359	2538	53.55 %	NOTCH1	ENST00000277541	missense_variant	p.Val1576Glu/c.4727T>A
UPN103	16	8989563	A	AC	1415	0	1422	0.00 %	1383	638	1393	45.80 %	USP7	ENST00000344836; ENST00000381886; ENST00000535863; ENST00000563085	frameshift_variant	p.Val952fs/c.2854dupG;p.Val936fs/c.2806dupG;p.Val853fs/c.2557dupG
UPN104	X	133551226	GC	TT	566	0	568	0.00 %	24	504	530	95.09 %	PHF6	ENST00000394292; ENST00000370803; ENST00000332070; ENST00000370799; ENST00000416404	missense_variant	p.Ala289Phe/c.865_866delGCinsTT;p.Ala288Phe/c.862_863delGCinsTT;p.Ala254Phe/c.760_761delGCinsTT
UPN104	1	115258747	C	T	1422	0	1424	0.00 %	780	723	1505	48.04 %	NRAS	ENST00000369535	missense_variant	p.Gly12Asp/c.35G>A
UPN104	19	15285054	G	A	1729	2	1733	0.12 %	487	409	896	45.65 %	NOTCH3	ENST00000263388	missense_variant	p.Pro1521Ser/c.4561C>T
UPN104	3	178936091	G	A	828	0	830	0.00 %	1578	115	1693	6.79 %	PIK3CA	ENST00000263967	missense_variant	p.Glu545Lys/c.1633G>A

UPN105	4	153268144	G	A	1448	0	1450	0.00 %	33	646	683	94.58 %	FBXW7	ENST00000281708; ENST00000296555; ENST00000263981; ENST00000603548; ENST00000393956; ENST00000603841	stop_gained	p.Arg222*/c.664C>T;p.Arg104*/c.310C>T;p.Arg142*/c.424C>T;p.Arg46*/c.136C>T
UPN105	19	15284997	G	A	2071	1	2077	0.05 %	586	585	1173	49.87 %	NOTCH3	ENST00000263388	missense_variant	p.Arg1540Cys/c.4618C>T
UPN105	X	133511739	T	TA	1216	0	1217	0.00 %	1040	515	1042	49.42 %	PHF6	ENST00000394292; ENST00000370803; ENST00000332070; ENST00000370799; ENST00000416404; ENST00000370800	frameshift_variant	p.Leu32fs/c.93dupA
UPN105	4	106164029	T	A	1415	3	1419	0.21 %	497	435	934	46.57 %	TET2	ENST00000513237; ENST00000540549; ENST00000545826; ENST00000265149; ENST00000380013	missense_variant	p.Val1201Asp/c.3602T>A;p.Val1180Asp/c.3539T>A;p.Ser1150Thr/c.3448T>A
UPN105	12	122261323	G	A	1261	0	1264	0.00 %	432	309	741	41.70 %	SETD1B	ENST00000604567; ENST00000542440; ENST00000267197	stop_gained	p.Trp1613*/c.4838G>A;p.Trp1570*/c.4709G>A
UPN106	no Varian															
UPN107	4	153244092	G	A	1287	2	1292	0.15 %	765	1136	1902	59.73 %	FBXW7	ENST00000281708; ENST00000296555; ENST00000263981; ENST00000603548; ENST00000393956; ENST00000603841	missense_variant	p.Arg689Trp/c.2065C>T;p.Arg571Trp/c.1711C>T;p.Arg609Trp/c.1825C>T;p.Arg513Trp/c.1537C>T
UPN107	X	133527949	C	T	1023	1	1029	0.10 %	185	146	332	43.98 %	PHF6	ENST00000394292; ENST00000370803; ENST00000332070; ENST00000370799; ENST00000416404; ENST00000370800	stop_gained	p.Arg129*/c.385C>T;p.Arg95*/c.283C>T
UPN107	17	30302654	T	C	801	2	807	0.25 %	621	389	1011	38.48 %	SUZ12	ENST00000322652; ENST00000580398	missense_variant	p.Ser249Pro/c.745T>C;p.Ser226Pro/c.676T>C

UPN107	19	10886515	C	CAGTGAAAA	1395	0	1396	0.00 %	890	262	891	29.41 %	DNM2	ENST00000314646; ENST00000585892; ENST00000359692; ENST00000389253; ENST00000355667; ENST00000408974; ENST00000586939	frameshift_variant	p.Thr175fs/c.523_524insGTGAAAAA;p.Thr17fs/c.49_50insGTGAAAAA
UPN107	10	89692835	G	T	1044	1	1046	0.10 %	624	241	866	27.83 %	PTEN	ENST00000371953	missense_variant	p.Asp107Tyr/c.319G>T
UPN107	14	99640934	TCTCGGACGAGTG	T	988	0	990	0.00 %	320	120	441	27.21 %	BCL11B	ENST00000357195; ENST00000345514; ENST00000443726	inframe_deletion	p.His743_Glu746del/c.2227_2238delCACTCGTCCGAG;p.His672_Glu675del/c.2014_2025delCACTCGTCCGAG;p.His5
UPN107	17	30267504	A	G	710	0	713	0.00 %	202	64	266	24.06 %	SUZ12	ENST00000322652; ENST00000580398	missense_variant+splice_region_variant	p.Arg129Gly/c.385A>G;p.Ser129Gly/c.385A>G
UPN107	10	89717712	C	T	1103	0	1106	0.00 %	468	118	586	20.14 %	PTEN	ENST00000371953	missense_variant	p.Pro246Leu/c.737C>T
UPN107	10	89717671	ACGA	AGGGGGCCCC	1009	4	1011	0.40 %	534	35	569	6.15 %	PTEN	ENST00000371953	missense_variant+inframe_insertion	p.Arg233delinsGlyGlyPro/c.697_699delCGAinsGGGGGCCCC
UPN108	19	17949121	T	G	1474	2	1481	0.14 %	1186	35	1222	2.86 %	JAK3	ENST00000458235; ENST00000527670; ENST00000534444	missense_variant	p.Gln507Pro/c.1520A>C
UPN109	1	115256530	G	T	1361	2	1364	0.15 %	568	574	1150	49.91 %	NRAS	ENST00000369535	missense_variant	p.Gln61Lys/c.181C>A
UPN109	4	153249385	G	A	1553	2	1560	0.13 %	675	652	1333	48.91 %	FBXW7	ENST00000281708; ENST00000296555; ENST00000263981; ENST00000603548; ENST00000393956; ENST00000603841	missense_variant	p.Arg465Cys/c.1393C>T;p.Arg347Cys/c.1039C>T;p.Arg385Cys/c.1153C>T;p.Arg289Cys/c.865C>T
UPN109	19	15281554	C	T	1528	0	1529	0.00 %	1042	219	1264	17.33 %	NOTCH3	ENST00000263388	missense_variant	p.Ala1607Thr/c.4819G>A
UPN109	19	15285008	C	A	2036	4	2043	0.20 %	1605	173	1783	9.70 %	NOTCH3	ENST00000263388	missense_variant	p.Ser1536Ile/c.4607G>T
UPN109	7	50467716	C	A	1625	2	1630	0.12 %	1672	42	1718	2.44 %	IKZF1	ENST00000331340; ENST00000359197; ENST00000343574; ENST00000346667; ENST00000349824; ENST00000357364; ENST00000438033; ENST00000439701	missense_variant	p.Asn317Lys/c.951C>A;p.Asn275Lys/c.825C>A;p.Asn230Lys/c.690C>A;p.Asn87Lys/c.261C>A;p.Asn174Lys/c.522C>A
UPN110	9	139400258	T	G	1608	3	1615	0.19 %	1294	467	1765	26.46 %	NOTCH1	ENST00000277541	missense_variant	p.Ile1364Leu/c.4090A>C

UPN110	5	67591106	A	G	1075	3	1079	0.28 %	816	135	953	14.17 %	PIK3R1	ENST00000396611; ENST00000521381; ENST00000521657; ENST00000274335; ENST00000320694; ENST00000336483; ENST00000523872	missense_variant	p.Lys567Glu/c.1699A>G;p.Lys267Glu/c.799A>G;p.Lys297Glu/c.889A>G;p.Lys204Glu/c.610A>G
UPN110	9	139391137	TGTGCAG	T	1677	0	1679	0.00 %	1621	159	1783	8.92 %	NOTCH1	ENST00000277541	frameshift_variant	p.Leu2350fs/c.7047_7053delGCTGCA
UPN110	4	153249385	G	A	1249	1	1256	0.08 %	1258	104	1365	7.62 %	FBXW7	ENST00000281708; ENST00000296555; ENST00000263981; ENST00000603548; ENST00000393956; ENST00000603841	missense_variant	p.Arg465Cys/c.1393C>T;p.Arg347Cys/c.1039C>T;p.Arg385Cys/c.1153C>T;p.Arg289Cys/c.865C>T
UPN110	9	139391392	CAAAGG	GGCCCGTCT	1584	10	1592	0.63 %	1656	85	1737	4.89 %	NOTCH1	ENST00000277541	missense_variant+inframe_insertion	p.Ala2265_Glu2267delinsGluThrGlyGln/c.6794_6799delCCTTTGinsAGACGG
UPN111	no Varian															
UPN112	X	133511733	GACAGTTACTA	G	548	0	549	0.00 %	230	752	982	76.58 %	PHF6	ENST00000394292; ENST00000370803; ENST00000332070; ENST00000370799; ENST00000416404; ENST00000370800	frameshift_variant	p.Gln30fs/c.88_97delCAGTTACTAA
UPN112	10	89717712	C	CGAGATGA	1220	0	1222	0.00 %	2109	683	2133	32.02 %	PTEN	ENST00000371953	frameshift_variant+stop_gained	p.Leu247fs/c.738_739insAGATGAG
UPN113	X	70342084	G	A	901	0	902	0.00 %	344	262	606	43.23 %	MED12	ENST00000333646; ENST00000374102; ENST00000374080	stop_gained	p.Trp379*/c.1136G>A
UPN113	X	133527608	CTG	C	475	0	475	0.00 %	167	87	254	34.25 %	PHF6	ENST00000394292; ENST00000370803; ENST00000332070; ENST00000370799; ENST00000416404; ENST00000370800	frameshift_variant	p.Ala108fs/c.321_322delTG;p.Ala74fs/c.219_220delTG

UPN113	7	148516687	C	T	1038	0	1039	0.00 %	478	125	603	20.73 %	EZH2	ENST00000320356; ENST00000492143; ENST00000478654; ENST00000460911; ENST00000350995; ENST00000541220; ENST00000476773; ENST00000483967; ENST00000498186; ENST00000483012; ENST00000536783	splice_donor_variant +intron_variant	c.999+1G>A;c.984+1G>A;c.957+1G>A; c.867+1G>A;n.1115+1G>A;c.*728+1G >A;c.657+1G>A
UPN113	19	10934481	T	C	1144	0	1209	0.00 %	794	159	993	16.01 %	DNM2	ENST00000314646; ENST00000585892; ENST00000359692; ENST00000389253; ENST00000355667; ENST00000408974	missense_variant	p.Leu600Pro/c.1799T>C;p.Leu596Pro/ c.1787T>C
UPN114	9	139399320	CGCTTCA	GGCCAAC	0	0	1520	0.00 %	994	605	1551	39.01 %	NOTCH1	ENST00000277541	missense_variant	p.PheLysArg1606CysTrpPro/c.4817_4

UPN114	6	135502673	A	T	108	0	108	0.00 %	105	64	170	37.65 %	MYB	ENST00000341911;<br>ENST00000339290;<br>ENST00000367812;<br>ENST00000463282;<br>ENST00000525477;<br>ENST00000533837;<br>ENST00000316528;<br>ENST00000442647;<br>ENST00000367814;<br>ENST00000527615;<br>ENST00000420123;<br>ENST00000525369;<br>ENST00000524588;<br>ENST00000525002;<br>ENST00000525940;<br>ENST00000526187;<br>ENST00000526565;<br>ENST00000528015;<br>ENST00000528140;<br>ENST00000528345;<br>ENST00000528774;<br>ENST00000529262;<br>ENST00000531634;<br>ENST00000531737;<br>ENST00000533384;<br>ENST00000533624;<br>ENST00000534044;<br>ENST00000534121;<br>[illegible]	missense_variant+splice_region_variant	p.Ser8Cys/c.22A>T
UPN114	10	89717660	TCAGGACCCACACG	GCT	1141	4	1144	0.35 %	430	246	679	36.23 %	PTEN	ENST00000371953	frameshift_variant+missense_variant	p.Ser229fs/c.685_700delTCAGGACCCACACGACinsGCT
UPN114	10	89692978	C	A	912	2	915	0.22 %	416	87	503	17.30 %	PTEN	ENST00000371953	missense_variant	p.Phe154Leu/c.462C>A
UPN115	3	178921549	T	G	1191	0	1193	0.00 %	984	92	1080	8.52 %	PIK3CA	ENST00000263967	missense_variant	p.Val344Gly/c.1031T>G
UPN116	X	133512045	CT	C	268	0	269	0.00 %	18	238	259	91.89 %	PHF6	ENST00000394292;<br>ENST00000370803;<br>ENST00000332070;<br>ENST00000370799;<br>ENST00000370800	frameshift_variant	p.Ala51fs/c.150delT

UPN116	17	7577081	T	C	1785	1	1794	0.06 %	697	789	1489	52.99 %	TP53	ENST00000269305; ENST00000359597; ENST00000420246; ENST00000455263; ENST00000445888; ENST00000509690	missense_variant	p.Glu286Gly/c.857A>G;p.Glu154Gly/c.461A>G
UPN116	9	139397648	A	G	1592	2	1598	0.13 %	1126	498	1626	30.63 %	NOTCH1	ENST00000277541	missense_variant	p.Ile1718Thr/c.5153T>C
UPN116	9	139390864	C	CGG	1826	0	1828	0.00 %	1540	406	1566	25.93 %	NOTCH1	ENST00000277541	frameshift_variant	p.Val2443fs/c.7326_7327insCC
UPN116	9	139390703	G	GTTACAAA	1897	0	1903	0.00 %	1758	250	1762	14.19 %	NOTCH1	ENST00000277541	frameshift_variant+stop_gained	p.Thr2497fs/c.7487_7488insTTTGTAA
UPN116	9	139399313	T	TAGGACCTGC	2060	0	2067	0.00 %	1848	235	1877	12.52 %	NOTCH1	ENST00000277541	disruptive_inframe_insertion	p.Ala1610_His1611insGlnValLeu/c.4829_4830insGCAGGTCCT
UPN117	X	133547566	C	A	1334	0	1337	0.00 %	679	585	1265	46.25 %	PHF6	ENST00000394292; ENST00000370803; ENST00000332070; ENST00000370799; ENST00000416404; ENST00000370800	stop_gained	p.Ser156*/c.467C>A;p.Ser155*/c.464C>A;p.Ser121*/c.362C>A
UPN117	9	139399389	A	G	1947	0	1952	0.00 %	870	744	1618	45.98 %	NOTCH1	ENST00000277541	missense_variant	p.Leu1585Pro/c.4754T>C
UPN117	17	40359729	T	G	1163	0	1166	0.00 %	560	454	1014	44.77 %	STAT5B	ENST00000293328	missense_variant	p.Asn642His/c.1924A>C
UPN117	1	65303620	C	CAAAGGG	1047	0	1049	0.00 %	895	330	896	36.83 %	JAK1	ENST00000342505	disruptive_inframe_insertion	p.Val1045_Phe1046insProLeu/c.3134_3135insCCCTTT
UPN118	X	133527636	C	T	426	0	428	0.00 %	8	108	116	93.10 %	PHF6	ENST00000394292; ENST00000370803; ENST00000332070; ENST00000370799; ENST00000416404; ENST00000370800	stop_gained	p.Arg116*/c.346C>T;p.Arg82*/c.244C>T
UPN118	14	99723871	C	CG	1179	0	1182	0.00 %	1084	449	1088	41.27 %	BCL11B	ENST00000357195; ENST00000345514	frameshift_variant	p.Asp122fs/c.363dupC
UPN118	14	99641824	G	A	1057	0	1057	0.00 %	940	661	1607	41.13 %	BCL11B	ENST00000357195; ENST00000345514; ENST00000443726	missense_variant	p.Thr450Met/c.1349C>T;p.Thr379Met/c.1136C>T;p.Thr256Met/c.767C>T
UPN118	9	139399365	A	G	1624	0	1629	0.00 %	2422	1153	3587	32.14 %	NOTCH1	ENST00000277541	missense_variant	p.Leu1593Pro/c.4778T>C
UPN118	9	139390642	GGGACT	GGACCCGGACTCGAGAGG	1369	7	1371	0.51 %	1744	218	2008	10.86 %	NOTCH1	ENST00000277541	frameshift_variant+missense_variant	p.Glu2515fs/c.7544_7547delAGTCinsGGTTTTAGATCTCATCCTCTCGAGTCCG

UPN118	7	148525907	C	T	992	2	998	0.20 %	528	59	588	10.03 %	EZH2	ENST00000320356; ENST00000492143; ENST00000478654; ENST00000460911; ENST00000350995; ENST00000541220; ENST00000476773; ENST00000483967; ENST00000536783	missense_variant	p.Asp184Asn/c.550G>A;p.Asp175Asn/c.523G>A;p.Asp145Asn/c.433G>A;p.Asp75Asn/c.223G>A
UPN118	21	36164460	A	G	783	0	785	0.00 %	878	81	962	8.42 %	RUNX1	ENST00000300305; ENST00000344691; ENST00000325074; ENST00000437180; ENST00000399240	missense_variant	p.Leu472Pro/c.1415T>C;p.Leu445Pro/c.1334T>C;p.Leu460Pro/c.1379T>C;p.Leu381Pro/c.1142T>C
UPN118	6	135518167	A	T	1584	0	1585	0.00 %	846	73	920	7.93 %	MYB	ENST00000341911; ENST00000526187; ENST00000528015; ENST00000528774; ENST00000534121; ENST00000438901	missense_variant	p.Gln424His/c.1272A>T;p.Asn393Ile/c.1178A>T;p.Asn396Ile/c.1187A>T;p.Gln421His/c.1263A>T;p.Gln408His/c.1224A>T
UPN118	7	150700420	C	G	1177	3	1181	0.25 %	1855	145	2005	7.23 %	NOS3	ENST00000467517	missense_variant	p.Pro592Ala/c.1774C>G
UPN118	X	70349196	G	C	615	1	617	0.16 %	527	35	562	6.23 %	MED12	ENST00000333646; ENST00000374102; ENST00000374080	missense_variant	p.Cys1203Ser/c.3608G>C
UPN118	7	151836833	A	G	1185	0	1188	0.00 %	648	42	691	6.08 %	KMT2C	ENST00000485655; ENST00000355193; ENST00000360104; ENST00000262189; ENST00000424877	start_lost;missense_variant	p.Met1?/c.2T>C;p.Met4853Thr/c.14558T>C;p.Met2352Thr/c.7055T>C;p.Met4796Thr/c.14387T>C;p.Met1409Thr/c.4226T>C
UPN118	12	122255456	C	G	1534	0	1536	0.00 %	1433	63	1500	4.20 %	SETD1B	ENST00000604567	missense_variant	p.Ser1053Trp/c.3158C>G
UPN118	19	11114051	G	A	1209	5	1214	0.41 %	479	21	503	4.17 %	SMARCA4	ENST00000358026; ENST00000344626; ENST00000429416; ENST00000541122; ENST00000589677; ENST00000444061; ENST00000590574; ENST00000413806; ENST00000450717	missense_variant	p.Ser660Asn/c.1979G>A
UPN118	1	215953220	G	T	1329	1	1336	0.07 %	737	31	769	4.03 %	USH2A	ENST00000366943; ENST00000307340	missense_variant	p.Thr3635Asn/c.10904C>A

UPN118	1	115251211	TTGAG	GTGAT	1198	3	1201	0.25 %	530	22	554	3.97 %	NRAS	ENST00000369535	missense_variant	p.LeuAsn171IleThr/c.511_515delCTCA
UPN118	7	150700299	A	C	998	0	1001	0.00 %	1407	56	1471	3.81 %	NOS3	ENST00000484524	missense_variant	p.Asn605His/c.1813A>C
UPN118	13	77671690	T	G	1379	2	1385	0.14 %	857	30	887	3.38 %	MYCBP2	ENST00000407578; ENST00000544440; ENST00000357337	missense_variant	p.Lys3200Thr/c.9599A>C;p.Lys3162Thr/c.9485A>C
UPN118	7	151945499	C	T	1239	0	1241	0.00 %	722	25	749	3.34 %	KMT2C	ENST00000355193; ENST00000262189; ENST00000558084	missense_variant	p.Glu674Lys/c.2020G>A
UPN118	1	216062006	C	A	1322	1	1325	0.08 %	800	26	828	3.14 %	USH2A	ENST00000366943; ENST00000307340	missense_variant	p.Arg2662Ile/c.7985G>T
UPN118	14	99640790	T	A	898	0	898	0.00 %	1182	37	1222	3.03 %	BCL11B	ENST00000357195; ENST00000345514; ENST00000443726	missense_variant	p.Ser795Cys/c.2383A>T;p.Ser724Cys/c.2170A>T;p.Ser601Cys/c.1801A>T
UPN118	16	3820824	C	G	1752	0	1755	0.00 %	707	22	731	3.01 %	CREBBP	ENST00000262367; ENST00000382070	missense_variant	p.Gly876Ala/c.2627G>C;p.Gly838Ala/c.2513G>C
UPN118	11	118343429	C	T	1305	2	1308	0.15 %	696	20	717	2.79 %	KMT2A	ENST00000534358; ENST00000531904; ENST00000389506; ENST00000354520	missense_variant	p.Pro519Ser/c.1555C>T;p.Pro552Ser/c.1654C>T
UPN118	7	150700294	G	A	999	0	1000	0.00 %	1406	40	1447	2.76 %	NOS3	ENST00000484524	missense_variant	p.Ser603Asn/c.1808G>A
UPN118	9	94486540	G	A	1656	0	1662	0.00 %	1784	42	1827	2.30 %	ROR2	ENST00000375708; ENST00000375715	missense_variant	p.Leu746Phe/c.2236C>T;p.Leu606Phe/c.1816C>T
UPN118	1	216246266	T	A	1251	2	1255	0.16 %	887	20	908	2.20 %	USH2A	ENST00000366943; ENST00000307340	missense_variant	p.Tyr1941Phe/c.5822A>T
UPN118	7	150708877	C	G	837	0	837	0.00 %	1213	27	1243	2.17 %	NOS3	ENST00000475017	missense_variant+splice_region_variant	p.Leu289Val/c.865C>G
UPN118	12	49420504	A	C	1479	1	1480	0.07 %	1352	26	1380	1.88 %	KMT2D	ENST00000301067	missense_variant	p.Val5082Gly/c.15245T>G
UPN118	7	150700374	T	C	1302	0	1303	0.00 %	2304	44	2353	1.87 %	NOS3	ENST00000484524	stop_lost	p.Ter630Glnext*?/c.1888T>C
UPN118	12	122247563	T	C	1457	2	1462	0.14 %	1880	21	1902	1.10 %	SETD1B	ENST00000604567; ENST00000542440; ENST00000267197	missense_variant	p.Ser238Pro/c.712T>C
UPN118	7	150696301	G	A	1393	0	1397	0.00 %	1912	21	1938	1.08 %	NOS3	ENST00000297494; ENST00000461406; ENST00000467517; ENST00000484524	missense_variant	p.Gly327Asp/c.980G>A;p.Gly121Asp/c.362G>A
UPN118	9	139391313	G	C	1598	0	1599	0.00 %	3053	33	3092	1.07 %	NOTCH1	ENST00000277541	missense_variant	p.Ala2293Gly/c.6878C>G
UPN119	12	122252318	C	CTA	945	0	947	0.00 %	648	164	651	25.19 %	SETD1B	ENST00000604567; ENST00000542440; ENST00000267197	frameshift_variant	p.Pro733fs/c.2197_2198insTA

UPN120	no Varian															
UPN121	no Varian															
UPN122	1	65305400	G	C	NA	NA	NA	NA	885	371	1258	29.49 %	JAK1	ENST00000342505	missense_variant	p.Leu910Val/c.2728C>G
UPN122	17	7578409	C	T	NA	NA	NA	NA	1115	419	1541	27.19 %	TP53	ENST00000269305;<br>ENST00000413465;<br>ENST00000359597;<br>ENST00000420246;<br>ENST00000455263;<br>ENST00000445888;<br>ENST00000509690;<br>ENST00000514944	missense_variant	p.Arg174Lys/c.521G>A;p.Arg42Lys/c.125G>A;p.Arg81Lys/c.242G>A
UPN122	12	49435105	C	T	NA	NA	NA	NA	1197	444	1646	26.97 %	KMT2D	ENST00000301067	missense_variant	p.Val2150Met/c.6448G>A
UPN122	X	70348196	C	T	NA	NA	NA	NA	1007	332	1341	24.76 %	MED12	ENST00000333646;<br>ENST00000374102;<br>ENST00000374080	missense_variant	p.Ser1087Leu/c.3260C>T
UPN122	X	41205852	G	A	NA	NA	NA	NA	646	153	802	19.08 %	DDX3X	ENST00000399959;<br>ENST00000457138	missense_variant	p.Arg531His/c.1592G>A;p.Arg515His/c.1544G>A
UPN122	9	8507399	C	T	NA	NA	NA	NA	1346	83	1432	5.80 %	PTPRD	ENST00000356435;<br>ENST00000381196;<br>ENST00000355233;<br>ENST00000358503;<br>ENST00000360074;<br>ENST00000397611;<br>ENST00000397617;<br>ENST00000537002;<br>ENST00000540109;<br>ENST00000486161;<br>ENST00000397606	missense_variant	p.Glu527Lys/c.1579G>A;p.Glu514Lys/c.1540G>A;p.Glu524Lys/c.1570G>A;p.Glu517Lys/c.1549G>A
UPN123	1	9780155	TGCCCCTGTGTTCAGATGAGA	GGGCG	NA	NA	NA	NA	188	139	330	42.12 %	PIK3CD	ENST00000361110;<br>ENST00000536656;<br>ENST00000377346;<br>ENST00000543390	splice_acceptor_variant+disruptive_inframe_deletion+splice_region_variant+synonymous_variant+intron_variant	p.Asp412_Lys414delinsGlu/c.1235-15_1240delTGCCCCTGTGTTCAGATGAGAinsGGGCG;p.Asp447_Lys449delinsGlu/c.1340-15_1345delTGCCCCTGTGTTCAGATGAGAinsGGGCG;p.Asp114_Lys116delins
UPN123	19	54646874	CAAG	C	NA	NA	NA	NA	334	181	515	35.15 %	CNOT3	ENST00000221232;<br>ENST00000406403	inframe_deletion	p.Lys17del/c.49_51delAAG

UPN123	14	99642053	T	TGGCC	NA	NA	NA	NA	158	45	160	28.13 %	BCL11B	ENST00000357195; ENST00000345514; ENST00000443726	frameshift_variant	p.Ser374fs/c.1119_1120insGGCC;p.Ser303fs/c.906_907insGGCC;p.Ser180fs/c.537_538insGGCC
UPN124	9	139399356	A	T	1773	0	1776	0.00 %	1472	404	1879	21.50 %	NOTCH1	ENST00000277541	missense_variant	p.Leu1596His/c.4787T>A
UPN124	9	139397768	A	G	1751	3	1758	0.17 %	1496	354	1854	19.09 %	NOTCH1	ENST00000277541	missense_variant	p.Leu1678Pro/c.5033T>C
UPN124	9	139399350	C	G	1782	0	1783	0.00 %	1831	69	1907	3.62 %	NOTCH1	ENST00000277541	missense_variant	p.Arg1598Pro/c.4793G>C
UPN124	17	40359729	T	G	1127	1	1130	0.09 %	1018	32	1052	3.04 %	STAT5B	ENST00000293328	missense_variant	p.Asn642His/c.1924A>C
UPN124	19	17945918	A	G	1782	0	1783	0.00 %	1841	38	1884	2.02 %	JAK3	ENST00000458235	missense_variant	p.Val674Ala/c.2021T>C
UPN124_p	9	139399287	TAGGGGAAGATCATCTGCTG	CCCCTCTGGATAG	1555	16	1567	1.02 %	1736	22	1747	1.26 %	NOTCH1	ENST00000277541	missense_variant+disruptive_inframe_deletion	p.His1611_Tyr1619delinsProIleGlnArgGly/c.4832_4856delACGGCCAGCAGATGATCTTCCCCTAinsCTATCCAGAGGGG

Supplemental Data 1

					Germline				Tumor							
**ID**	**Chr**	**Pos**	**Ref**	**Alt**	**#REF**	**#ALT**	**DP**	**VAF**	**#REF**	**#ALT**	**DP**	**VAF**	**Gene**	**ENST**	**Type**	**Variant**
UPN124_r	9	139399356	A	T	1773	0	1776	0.00 %	461	467	942	49.58 %	NOTCH1	ENST00000277541	missense_variant	p.Leu1596His/c.4787T>A
UPN124_r	16	3781374	C	T	1724	3	1729	0.17 %	757	30	800	3.75 %	CREBBP	ENST00000262367	missense_variant	p.Arg1664His/c.4991G>A

Supplemental Data 1

ID	Chr	Pos	Ref	Alt	Germline #REF	Germline #ALT	Germline DP	Germline VAF	Tumor #REF	Tumor #ALT	Tumor DP	Tumor VAF	Gene	ENST	Type	Variant
UPN125	9	139399422	A	G	1974	1	1985	0.05 %	1520	1126	2650	42.49 %	NOTCH1	ENST00000277541	missense_variant	p.Leu1574Pro/c.4721T>C
UPN125	9	139390690	G	A	1871	1	1876	0.05 %	1398	912	2315	39.40 %	NOTCH1	ENST00000277541	stop_gained	p.Gln2501*/c.7501C>T
UPN125	19	10930662	GAGA	G	1422	0	1425	0.00 %	1108	604	1715	35.22 %	DNM2	ENST00000314646;ENST00000585892;ENST00000359692;ENST00000389253;ENST00000355667;E	inframe_deletion	p.Lys562del/c.1684_1686delAAG;p.Lys558del/c.1672_1674delAAG
UPN125	16	3789666	G	T	1443	0	1450	0.00 %	1212	526	1741	30.21 %	CREBBP	ENST00000262367;ENST00000382070;ENST00000573517;E	missense_variant	p.Ala1398Asp/c.4193C>A;p.Ala1360Asp/c.4079C>A;p.Ala166Asp/c.497C>A;p.Ala271Asp/c.812C>A
UPN125	6	41903798	C	CTTTAAGGGGGT	1766	0	1769	0.00 %	2180	588	2184	26.92 %	CCND3	ENST00000372991;ENST00000511642;ENST00000372987;ENST00000415497;ENST00000372988;E	frameshift_variant+stop_gained	p.Ser254fs/c.758_759insACCCCCTTAAA;p.Ser173fs/c.515_516insACCCCCTTAAA;p.Ser204fs/c.608_609insACCCCCTTAAA;p.Ser58fs/c.170_171insACCCCCTTAAA;p.Ser182fs/c.542_543insACCCCCT
UPN125	16	3823769	G	A	1164	0	1169	0.00 %	1293	35	1333	2.63 %	CREBBP	ENST00000262367;ENST00000382070;ENST00000571826;E	stop_gained	p.Gln816*/c.2446C>T;p.Gln778*/c.2332C>T;p.Gln165*/c.493C>T;p.Gln238*/c.712C>T
UPN126	5	67589609	GAAA	G	959	0	962	0.00 %	1034	218	1252	17.41 %	PIK3R1	ENST00000396611;ENST00000521381;ENST00000521657;ENST00000274335;ENST00000320694;ENST00000521409;ENST00000336483;E	disruptive_inframe_deletion	p.Lys459del/c.1376_1378delAAA;p.Lys159del/c.476_478delAAA;p.Lys96del/c.287_289delAAA;p.Lys189del/c.566_568delAAA;p.Lys132del/c.395_397delAAA
UPN127	X	133559238	TG	T	598	0	600	0.00 %	4	48	52	92.31 %	PHF6	ENST00000394292;ENST00000370803;ENST00000332070;E	frameshift_variant	p.Cys327fs/c.980delG;p.Cys326fs/c.977delG;p.Cys292fs/c.875delG
UPN127	9	139397768	A	G	2506	4	2519	0.16 %	611	699	1312	53.28 %	NOTCH1	ENST00000277541	missense_variant	p.Leu1678Pro/c.5033T>C
UPN127	14	99642100	G	T	1702	2	1707	0.12 %	1521	1310	2834	46.22 %	BCL11B	ENST00000357195;ENST00000345514;ENST00000443726	stop_gained	p.Ser358*/c.1073C>A;p.Ser287*/c.860C>A;p.Ser164*/c.491C>A
UPN127	4	153249385	G	A	1755	0	1760	0.00 %	196	127	325	39.08 %	FBXW7	ENST00000281708;ENST00000296555;ENST00000263981;ENST00000603548;ENST00000393956;E	missense_variant	p.Arg465Cys/c.1393C>T;p.Arg347Cys/c.1039C>T;p.Arg385Cys/c.1153C>T;p.Arg289Cys/c.865C>T
UPN127	19	15273221	T	G	0	0	1602	0.00 %	0	68	366	18.58 %	NOTCH3	ENST00000597756	missense_variant	p.Ile161Leu/c.481A>C

T-LBL pediatric_not relapsed

UPN127	7	151921255	C	T	1397	78	1478	5.28 %	375	46	421	10.93 %	KMT2C	ENST00000355193;ENST00000262189;ENST00000418673	stop_gained	p.Trp1056*/c.3168G>A;p.Trp211*/c.633G>A
UPN128	12	25398285	C	T	1329	100	1431	6.99 %	128	102	230	44.35 %	KRAS	ENST00000256078;ENST00000311936;ENST00000557334;E	missense_variant	p.Gly12Ser/c.34G>A
UPN128	4	153249385	G	A	1434	113	1553	7.28 %	192	139	331	41.99 %	FBXW7	ENST00000281708;ENST00000296555;ENST00000263981;ENST00000603548;ENST00000393956;E	missense_variant	p.Arg465Cys/c.1393C>T;p.Arg347Cys/c.1039C>T;p.Arg385Cys/c.1153C>T;p.Arg289Cys/c.865C>T
UPN128	14	99641430	G	GC	1369	103	1377	7.48 %	1291	548	1311	41.80 %	BCL11B	ENST00000357195;ENST00000345514;ENST00000443726	frameshift_variant	p.Gly582fs/c.1742dupG;p.Gly511fs/c.1529dupG;p.Gly388fs/c.1160dupG
UPN128	9	139397714	G	T	1927	160	2089	7.66 %	357	255	613	41.60 %	NOTCH1	ENST00000277541	missense_variant	p.Ala1696Asp/c.5087C>A
UPN128	4	106164884	C	T	1571	0	1574	0.00 %	284	40	324	12.35 %	TET2	ENST00000513237;ENST00000540549;ENST00000380013	missense_variant	p.Thr1272Met/c.3815C>T;p.Thr1251Met/c.3752C>T
UPN129	16	8989500	T	C	1003	1	1005	0.10 %	794	583	1380	42.25 %	USP7	ENST00000344836;ENST00000381886;ENST00000535863;ENST00000563085;ENST00000563961;E	missense_variant+splice_region_variant	p.Glu973Gly/c.2918A>G;p.Glu957Gly/c.2870A>G;p.Glu874Gly/c.2621A>G;c.*2503A>G;c.*2901A>G
UPN129	9	139390823	A	AATTTGTGAGGT	1582	0	1583	0.00 %	2126	383	2154	17.78 %	NOTCH1	ENST00000277541	frameshift_variant	p.Leu2457fs/c.7367_7368insACCTCACAAAT
UPN129	9	139399350	C	G	1674	0	1682	0.00 %	2168	71	2241	3.17 %	NOTCH1	ENST00000277541	missense_variant	p.Arg1598Pro/c.4793G>C
UPN129	9	139399389	A	G	1555	0	1555	0.00 %	2255	70	2329	3.01 %	NOTCH1	ENST00000277541	missense_variant	p.Leu1585Pro/c.4754T>C
UPN129	4	153247289	G	A	1404	0	1408	0.00 %	1998	48	2048	2.34 %	FBXW7	ENST00000281708;ENST00000296555;ENST00000263981;ENST00000603548;ENST00000393956;E	missense_variant	p.Arg505Cys/c.1513C>T;p.Arg387Cys/c.1159C>T;p.Arg425Cys/c.1273C>T;p.Arg329Cys/c.985C>T
UPN130	16	9010364	G	GGCTACA	1002	8	1015	0.79 %	896	327	905	36.13 %	USP7	ENST00000344836;ENST00000381886;ENST00000535863;ENST00000563085;E	inframe_insertion	p.Cys300_Arg301insCysSer/c.900_901insTGTAGC;p.Cys284_Arg285insCysSer/c.852_853insTGTAGC;p.Cys201_Arg202insCysSer/c.603_604insTGTAGC;p.Cys
UPN130	9	139399422	A	G	1958	21	1983	1.06 %	1491	811	2309	35.12 %	NOTCH1	ENST00000277541	missense_variant	p.Leu1574Pro/c.4721T>C
UPN130	12	49440062	G	A	1817	29	1848	1.57 %	1234	601	1840	32.66 %	KMT2D	ENST00000301067	stop_gained	p.Gln1522*/c.4564C>T

UPN130	4	153249384	C	T	1518	4	1526	0.26 %	1281	265	1548	17.12 %	FBXW7	ENST00000281708;ENST00000296555;ENST00000263981;ENST00000603548;ENST00000393956;	missense_variant	p.Arg465His/c.1394G>A;p.Arg347His/c.1040G>A;p.Arg385His/c.1154G>A;p.Arg289His/c.866G>A
UPN130	4	153249385	G	A	1498	4	1512	0.26 %	1289	243	1534	15.84 %	FBXW7	ENST00000281708;ENST00000296555;ENST00000263981;ENST00000603548;ENST00000393956;	missense_variant	p.Arg465Cys/c.1393C>T;p.Arg347Cys/c.1039C>T;p.Arg385Cys/c.1153C>T;p.Arg289Cys/c.865C>T
UPN130	6	41904404	T	C	1476	1	1482	0.07 %	1454	234	1691	13.84 %	CCND3	ENST00000372991;ENST00000511642;ENST00000372987;ENST00000415497;ENST00000372988;ENST00000414200;E	missense_variant	p.Met202Val/c.604A>G;p.Met121Val/c.361A>G;p.Met152Val/c.454A>G;p.Met6Val/c.16A>G;p.Met130Val/c.388A>G;p.Met136Val/c.406A>G
UPN130	9	139399367	G	GCCC	1851	0	1851	0.00 %	2274	284	2279	12.46 %	NOTCH1	ENST00000277541	disruptive_inframe_i	p.Phe1592delinsLeuGly/c.4775_4776in
UPN130	6	41904418	A	T	1389	4	1395	0.29 %	1413	192	1610	11.93 %	CCND3	ENST00000415497;ENST00000508143;ENST00000372991;ENST00000511642;ENST00000372987;ENST00000372988;ENST00000414200;E	start_lost;missense_variant	p.Met1?/c.2T>A;p.Met197Lys/c.590T>A;p.Met116Lys/c.347T>A;p.Met147Lys/c.440T>A;p.Met125Lys/c.374T>A;p.Met131Lys/c.392T>A
UPN131	9	139399324	T	TGAGGGATCC	1812	0	1819	0.00 %	1565	326	1567	20.80 %	NOTCH1	ENST00000277541	inframe_insertion	p.Phe1606_Lys1607insGlySerLeu/c.4818_4819insGGATCCCTC
UPN132	9	139390655	C	CG	1331	0	1335	0.00 %	2029	899	2038	44.11 %	NOTCH1	ENST00000277541	frameshift_variant	p.Ser2513fs/c.7535dupC
UPN132	9	139399367	G	GAGTTTCGGGCGCTCC	1621	3	1622	0.18 %	2185	733	2189	33.49 %	NOTCH1	ENST00000277541	disruptive_inframe_insertion	p.Phe1592delinsLeuGluArgProLysLeu/c.4775_4776insGGAGCGCCCGAAACT
UPN133	3	178951959	T	A	1351	13	1367	0.95 %	673	599	1273	47.05 %	PIK3CA	ENST00000263967	missense_variant	p.Met1005Lys/c.3014T>A
UPN133	14	99641885	C	A	1593	11	1609	0.68 %	695	618	1314	47.03 %	BCL11B	ENST00000357195;ENST00000345514;ENST00000443726	stop_gained	p.Glu430*/c.1288G>T;p.Glu359*/c.1075G>T;p.Glu236*/c.706G>T
UPN133	9	139399368	A	G	2139	21	2163	0.97 %	933	743	1679	44.25 %	NOTCH1	ENST00000277541	missense_variant	p.Phe1592Ser/c.4775T>C
UPN133	X	70339233	C	CGGCCAGGGT	794	0	798	0.00 %	548	90	550	16.36 %	MED12	ENST00000333646;ENST00000374102;ENST00000374080;E	inframe_insertion	p.Ala38_Leu39insArgValAla/c.114_115insAGGGTGGCC;p.Ala22_Leu23insArgValAla/c.66_67insAGGGTGGCC
UPN133	1	9777666	C	A	1705	3	1715	0.17 %	1252	152	1407	10.80 %	PIK3CD	ENST00000361110;ENST00000536656;ENST00000377346	missense_variant	p.Asn299Lys/c.897C>A;p.Asn334Lys/c.1002C>A

UPN133	19	54653360	CAG	C	2058	0	2063	0.00 %	1365	119	1488	8.00 %	CNOT3	ENST00000221232;ENST00000358389;ENST00000406403;E	frameshift_variant	p.Gly493fs/c.1473_1474delAG;p.Gly312fs/c.930_931delAG;p.Gly24fs/c.66_67delAG
UPN134	10	89717668	C	CACCG	941	68	942	7.22 %	568	491	569	86.29 %	PTEN	ENST00000371953	frameshift_variant	p.Arg233fs/c.695_696insCGAC
UPN134	6	41903800	C	CCCTACTTTTT	1552	127	1554	8.17 %	1537	497	1541	32.25 %	CCND3	ENST00000372991;ENST00000511642;ENST00000372987;ENST00000415497;ENST00000372988;E	frameshift_variant+stop_gained	p.Glu253fs/c.756_757insAAAAAGTAGG;p.Glu172fs/c.513_514insAAAAAGTAGG;p.Glu203fs/c.606_607insAAAAAGTAGG;p.Glu57fs/c.168_169insAAAAAGTAGG;p.Glu181fs/c.540_541insAAAAAG
UPN134	7	151848562	G	GGGACCCAGTTCACTTTCCCTCTGAAGTATTCA	969	29	972	2.98 %	982	164	986	16.63 %	KMT2C	ENST00000355193;ENST00000360104;ENST00000262189;ENST00000424877	frameshift_variant+stop_gained	p.Arg4268fs/c.12801_12802insTGAATACTTCAGAGGGAAAGTGAACTGGGTCC;p.Arg1771fs/c.5310_5311insTGAATACTTCAGAGGGAAAGTGAACTGGGTCC;p.Arg4211fs/c.12630_12631insTGAATAC
UPN134	19	11105661	G	A	1178	2	1182	0.17 %	1534	75	1609	4.66 %	SMARCA4	ENST00000358026;ENST00000344626;ENST00000429416;ENST00000541122;ENST00000589677;ENST00000444061;ENST00000590574;E	missense_variant	p.Arg526His/c.1577G>A
UPN135	X	133547550	G	GGAACTCCTGGGGGATGA	471	1	472	0.21 %	269	136	277	49.10 %	PHF6	ENST00000394292;ENST00000370803;ENST00000332070;ENST00000370799;ENST00000416404;E	frameshift_variant+stop_gained	p.Glu151fs/c.451_452insGAACTCCTGGGGGATGA;p.Glu150fs/c.448_449insGAACTCCTGGGGGATGA;p.Glu116fs/c.346_347insGAACTCCTGGGGGATGA
UPN135	14	99641779	G	A	753	12	769	1.56 %	896	563	1462	38.51 %	BCL11B	ENST00000357195;ENST00000345514;ENST00000443726	missense_variant	p.Ser465Leu/c.1394C>T;p.Ser394Leu/c.1181C>T;p.Ser271Leu/c.812C>T
UPN135	16	9010937	T	TCATAGAACACTCTTTGTAATGCTAAAG	929	3	933	0.32 %	1075	122	1096	11.13 %	USP7	ENST00000344836;ENST00000381886;ENST00000535863;ENST00000563085;ENST00000542333	inframe_insertion	p.Tyr265_Glu266insAlaLeuAlaLeuGlnArgValPheTyr/c.770_796dupCTTTAGCATTACAAAGAGTGTTCTATG;p.Tyr249_Glu250insAlaLeuAlaLeuGlnArgValPheTyr/c.722_748dupCTTTAGCATTACAAAGAGT
UPN136	X	44942749	T	TGAG	607	63	608	######	526	477	532	89.66 %	KDM6A	ENST00000382899;ENST00000377967;ENST00000536777;ENST00000543216;ENST00000414389;E	inframe_insertion	p.Arg1118dup/c.3351_3352insAGG;p.Arg1111dup/c.3330_3331insAGG;p.Arg1066dup/c.3195_3196insAGG;p.Arg1032dup/c.3093_3094insAGG;p.Arg708dup/c.2121_2122insAGG;p.Arg753dup
UPN136	9	139397762	A	T	1602	65	1669	3.89 %	1073	1010	2090	48.33 %	NOTCH1	ENST00000277541	missense_variant	p.Ile1680Asn/c.5039T>A
UPN137	5	35874587	T	TGGGGTG	1360	0	1363	0.00 %	723	287	730	39.32 %	IL7R	ENST00000303115	inframe_insertion	p.Leu248_Ser249insGlyTrp/c.744_745i

UPN137	9	139399324	T	GGGG	1726	0	1729	0.00 %	1461	1008	2500	40.32 %	NOTCH1	ENST00000277541	missense_variant+inf	p.Lys1607delinsProGln/c.4819delAinsC
UPN138	X	133551305	T	C	527	0	527	0.00 %	3	130	134	97.01 %	PHF6	ENST00000394292;ENST00000370803;ENST00000332070;ENST00000370799;E	missense_variant	p.Ile315Thr/c.944T>C;p.Ile314Thr/c.941T>C;p.Ile280Thr/c.839T>C
UPN138	14	99641900	T	TG	1315	0	1319	0.00 %	1605	585	1614	36.25 %	BCL11B	ENST00000357195;ENST00000345514;ENST00000443726	frameshift_variant	p.Lys425fs/c.1272dupC;p.Lys354fs/c.1059dupC;p.Lys231fs/c.690dupC
UPN138	9	139390809	CTCTCCTGGGGCAGAATAGTGTGCACCGCCAGGCTGC	CCCGGA	1485	7	1487	0.47 %	734	262	735	35.65 %	NOTCH1	ENST00000277541	frameshift_variant+missense_variant	p.Ser2449fs/c.7346_7381delGCAGCCTGGCGGTGCACACTATTCTGCCCCAGGAGAinsTCCGG
UPN139	X	133551266	AC	A	661	0	662	0.00 %	38	576	629	91.57 %	PHF6	ENST00000394292;ENST00000370803;ENST00000332070;ENST00000370799;E	frameshift_variant	p.His303fs/c.907delC;p.His302fs/c.904delC;p.His268fs/c.802delC
UPN139	4	153249384	C	T	1560	1	1567	0.06 %	737	818	1559	52.47 %	FBXW7	ENST00000281708;ENST00000296555;ENST00000263981;ENST00000603548;ENST00000393956;E	missense_variant	p.Arg465His/c.1394G>A;p.Arg347His/c.1040G>A;p.Arg385His/c.1154G>A;p.Arg289His/c.866G>A
UPN139	9	139399344	A	G	1921	0	1925	0.00 %	1027	914	1947	46.94 %	NOTCH1	ENST00000277541	missense_variant	p.Leu1600Pro/c.4799T>C
UPN139	4	153271213	T	TG	1033	0	1033	0.00 %	952	422	961	43.91 %	FBXW7	ENST00000281708;ENST00000296555;ENST00000263981;ENST00000603548;E	frameshift_variant	p.Lys189fs/c.564dupC;p.Lys71fs/c.210dupC;p.Lys109fs/c.324dupC
UPN139	12	49420631	C	T	1762	0	1766	0.00 %	1482	390	1873	20.82 %	KMT2D	ENST00000301067	missense_variant	p.Asp5040Asn/c.15118G>A
UPN139	12	49437701	G	A	1701	0	1705	0.00 %	1539	293	1836	15.96 %	KMT2D	ENST00000301067	stop_gained	p.Arg1757*/c.5269C>T
UPN140	9	139399422	A	G	1297	22	1325	1.66 %	1288	1131	2430	46.54 %	NOTCH1	ENST00000277541	missense_variant	p.Leu1574Pro/c.4721T>C
UPN140	16	9012906	G	GA	899	29	901	3.22 %	438	163	438	37.21 %	USP7	ENST00000344836;ENST00000381886;ENST00000535863;ENST00000563085;E	frameshift_variant	p.Thr235fs/c.701dupT;p.Thr219fs/c.653dupT;p.Thr136fs/c.404dupT;p.Thr177fs/c.527dupT
UPN140	14	99641888	A	AGTCCCTAAGCGGGGCAGCCCCGC	892	3	892	0.34 %	1889	250	1891	13.22 %	BCL11B	ENST00000357195;ENST00000345514;ENST00000443726	frameshift_variant	p.Cys429fs/c.1284_1285insGCGGGGCTGCCCCGCTTAGGGAC;p.Cys358fs/c.1071_1072insGCGGGGCTGCCCCGCTTAGGGAC;p.Cys235fs/c.702_703insGCGGGG
UPN141	9	139412636	C	A	1490	378	1871	######	361	293	654	44.80 %	NOTCH1	ENST00000277541	missense_variant	p.Gly403Val/c.1208G>T

UPN141	16	8995038	A	G	1074	212	1289	######	281	143	424	33.73 %	USP7	ENST00000344836;ENST00000381886;ENST00000535863;ENST00000563085;ENST00000563043;E	missense_variant	p.Tyr701His/c.2101T>C;p.Tyr685His/c.2053T>C;p.Tyr602His/c.1804T>C;p.Tyr9His/c.25T>C;p.Tyr2His/c.4T>C
UPN141	7	151921255	C	T	1211	62	1276	4.86 %	304	50	355	14.08 %	KMT2C	ENST00000355193;ENST00000262189;ENST00000418673	stop_gained	p.Trp1056*/c.3168G>A;p.Trp211*/c.633G>A
UPN142	X	41196694	CAG	C	523	0	526	0.00 %	123	154	277	55.60 %	DDX3X	ENST00000399959;ENST00000457138;ENST00000441189;E	frameshift_variant	p.Ser28fs/c.82_83delAG;p.Ser72fs/c.214_215delAG
UPN142	9	139390834	C	CCG	1634	0	1635	0.00 %	1094	442	1103	40.07 %	NOTCH1	ENST00000277541	frameshift_variant	p.Val2453fs/c.7355_7356dupCG
UPN142	14	99641583	C	CGGGGGTT	1040	0	1041	0.00 %	1017	349	1018	34.28 %	BCL11B	ENST00000357195;ENST00000345514;ENST00000443726	frameshift_variant	p.Glu531fs/c.1589_1590insAACCCCC;p.Glu460fs/c.1376_1377insAACCCCC;p.Glu337fs/c.1007_1008insAACCCCC
UPN142	10	89717678	GAAGAC	CAGTAGG	1105	7	1106	0.63 %	352	85	440	19.32 %	PTEN	ENST00000371953	frameshift_variant+s	p.Glu235fs/c.703_708delGAAGACinsC
UPN142	X	41206941	A	AC	572	0	573	0.00 %	297	51	299	17.06 %	DDX3X	ENST00000399959;ENST00000457138;ENST00000441189	frameshift_variant	p.Ser654fs/c.1959dupC;p.Ser638fs/c.1911dupC;p.Ser131fs/c.390dupC
UPN142	10	89717672	C	GA	1131	0	1133	0.00 %	370	73	483	15.11 %	PTEN	ENST00000371953	frameshift_variant+	p.Arg233fs/c.697delCinsGA
UPN142	10	89717658	ATTCAGGACCCACACGA	TTTTTTTTGAGGG	1112	4	1114	0.36 %	370	37	452	8.19 %	PTEN	ENST00000371953	frameshift_variant+missense_variant	p.Asn228fs/c.683_699delATTCAGGACCCACACGAinsTTTTTTTTGAGGG
UPN142	4	153249384	C	T	1229	4	1239	0.32 %	647	40	689	5.81 %	FBXW7	ENST00000281708;ENST00000296555;ENST00000263981;ENST00000603548;ENST00000393956;E	missense_variant	p.Arg465His/c.1394G>A;p.Arg347His/c.1040G>A;p.Arg385His/c.1154G>A;p.Arg289His/c.866G>A
UPN142	19	11129699	G	C	1458	0	1461	0.00 %	684	37	723	5.12 %	SMARCA4	ENST00000358026;ENST00000344626;ENST00000429416;ENST00000541122;ENST00000589677;ENST00000444061;ENST00000590574;E	missense_variant+splice_region_variant	p.Lys835Asn/c.2505G>C
UPN143	4	153249385	G	A	1028	2	1032	0.19 %	722	647	1372	47.16 %	FBXW7	ENST00000281708;ENST00000296555;ENST00000263981;ENST00000603548;ENST00000393956;E	missense_variant	p.Arg465Cys/c.1393C>T;p.Arg347Cys/c.1039C>T;p.Arg385Cys/c.1153C>T;p.Arg289Cys/c.865C>T
UPN143	9	139390861	G	A	1574	3	1578	0.19 %	1524	491	2021	24.29 %	NOTCH1	ENST00000277541	stop_gained	p.Gln2444*/c.7330C>T

UPN143	9	139399350	C	G	1619	0	1622	0.00 %	1829	378	2210	17.10 %	NOTCH1	ENST00000277541	missense_variant	p.Arg1598Pro/c.4793G>C
UPN143	9	139397768	A	T	1581	1	1590	0.06 %	1905	64	1979	3.23 %	NOTCH1	ENST00000277541	missense_variant	p.Leu1678Gln/c.5033T>A
UPN144	9	139390863	ACG	AATTCGAGGAC	1254	9	1255	0.72 %	1779	671	1784	37.61 %	NOTCH1	ENST00000277541	frameshift_variant+missense_variant	p.Asp2442fs/c.7326_7327delCGinsGTCCTCGAAT
UPN144	9	139399325	G	GTGGCCT	1213	1	1213	0.08 %	2373	817	2382	34.30 %	NOTCH1	ENST00000277541	disruptive_inframe_i	p.Phe1606delinsLeuGlyHis/c.4817_481
UPN144	19	11132438	G	A	1227	0	1229	0.00 %	1573	553	2131	25.95 %	SMARCA4	ENST00000358026;ENST00000344626;ENST00000429416;ENST00000541122;ENST00000589677;ENST00000444061;ENST00000590574	missense_variant	p.Arg885His/c.2654G>A
UPN144	14	99641516	C	A	988	7	996	0.70 %	1729	85	1816	4.68 %	BCL11B	ENST00000357195;ENST00000345514;ENST00000443726	stop_gained	p.Glu553*/c.1657G>T;p.Glu482*/c.1444G>T;p.Glu359*/c.1075G>T
UPN144	6	135517050	AGCAAGGT	TCCCA	1252	7	1258	0.56 %	1773	49	1828	2.68 %	MYB	ENST00000525940;ENST00000526187;ENST00000528015;ENST00000528140;ENST00000529262;ENST00000531737;ENST00000526320;ENST00000341911;ENST00000339290;ENST00000367812;ENST00000533837;ENST00000316528;ENST00000442647;ENST00000367814;ENST00000527615;ENST00000525002;ENST00000528774;ENST00000533384;ENST00000533624;E	frameshift_variant+splice_donor_variant+missense_variant+splice_region_variant+intron_variant	p.Ala372fs/c.1113_1118+2delAGCAAGGTinsTCCCA;p.Ala369fs/c.1104_1109+2delAGCAAGGTinsTCCCA;p.Ala372_Cys374delinsProSer/c.1113_1120delAGCAAGGTinsTCCCA;p.Ala369_Cys371delinsProSer/c.1104_1111delAGCAAGGTinsTCCCA;p.Ala337_Cys339delinsProSer/c.1008_1015delAGCAAGGTinsTCCCA;p.Ala56_Cys58delinsProSer/c.165_172delAGCAAGGTinsTCCCA;c.*182_*187+2delAGCAAGGTinsTCCCA
UPN145	9	139399386	C	G	1549	0	1551	0.00 %	2020	506	2532	19.98 %	NOTCH1	ENST00000277541	missense_variant	p.Arg1586Pro/c.4757G>C
UPN145	9	139399389	AGCTGCTCCGGCGGC	AGT	1528	6	1531	0.39 %	2049	348	2477	14.05 %	NOTCH1	ENST00000277541	inframe_deletion+synonymous_variant	p.Met1580_Gln1584delinsIle/c.4740_4752delGCCGCCGGAGCAGinsA
UPN145	9	139399396	C	CCGG	1576	1	1577	0.06 %	2099	128	2104	6.08 %	NOTCH1	ENST00000277541	inframe_insertion	p.Pro1582dup/c.4744_4746dupCCG
UPN146	9	139399344	A	G	1671	2	1675	0.12 %	748	386	1137	33.95 %	NOTCH1	ENST00000277541	missense_variant	p.Leu1600Pro/c.4799T>C

UPN146	4	153249384	C	T	1221	0	1221	0.00 %	343	140	483	28.99 %	FBXW7	ENST00000281708;ENST00000296555;ENST00000263981;ENST00000603548;ENST00000393956;	missense_variant	p.Arg465His/c.1394G>A;p.Arg347His/c.1040G>A;p.Arg385His/c.1154G>A;p.Arg289His/c.866G>A
UPN147	9	8341217	G	A	373	332	707	######	11	173	184	94.02 %	PTPRD	ENST00000356435;ENST00000381196;ENST00000355233;ENST00000358503;ENST00000360074;ENST00000397611;ENST00000397617;ENST00000537002;	missense_variant	p.Pro1667Ser/c.4999C>T;p.Pro1261Ser/c.3781C>T;p.Pro1645Ser/c.4933C>T;p.Pro1654Ser/c.4960C>T;p.Pro1257Ser/c.3769C>T;p.Pro1260Ser/c.3778C>T
UPN147	9	139399324	T	TGATCAGGTC	1241	2	1243	0.16 %	1212	804	1217	66.06 %	NOTCH1	ENST00000277541	inframe_insertion	p.Phe1606_Lys1607insAspLeuIle/c.4818_4819insGACCTGATC
UPN148	X	133527949	C	T	535	2	537	0.37 %	31	525	556	94.42 %	PHF6	ENST00000394292;ENST00000370803;ENST00000332070;ENST00000370799;ENST00000416404;	stop_gained	p.Arg129*/c.385C>T;p.Arg95*/c.283C>T
UPN148	3	178921548	G	A	1023	0	1030	0.00 %	106	543	653	83.15 %	PIK3CA	ENST00000263967	missense_variant	p.Val344Met/c.1030G>A
UPN148	11	118347544	A	G	1017	2	1021	0.20 %	660	680	1341	50.71 %	KMT2A	ENST00000534358;ENST00000531904;ENST00000389506;ENST00000354520;	missense_variant	p.Thr1061Ala/c.3181A>G;p.Thr1094Ala/c.3280A>G;p.Thr139Ala/c.415A>G
UPN148	7	148504761	C	T	1120	0	1121	0.00 %	777	714	1494	47.79 %	EZH2	ENST00000320356;ENST00000478654;ENST00000460911;ENST00000350995;ENST00000541220;	missense_variant	p.Glu745Lys/c.2233G>A;p.Glu689Lys/c.2065G>A;p.Glu740Lys/c.2218G>A;p.Glu701Lys/c.2101G>A;p.Glu731Lys/c.2191G>A
UPN148	12	122242753	A	G	1088	5	1111	0.45 %	969	851	1829	46.53 %	SETD1B	ENST00000604567;ENST00000542440;ENST00000267197	missense_variant	p.Asp37Gly/c.110A>G
UPN148	9	139397700	C	G	1212	1	1220	0.08 %	2229	148	2379	6.22 %	NOTCH1	ENST00000277541	missense_variant	p.Ala1701Pro/c.5101G>C
UPN148	9	139390984	G	GGCAGCAAAA	1435	0	1439	0.00 %	2412	57	2414	2.36 %	NOTCH1	ENST00000277541	inframe_insertion	p.Ile2402_Gln2403insPheCysCys/c.7206_7207insTTTTGCTGC
UPN149	17	40359659	T	A	1192	0	1195	0.00 %	382	210	592	35.47 %	STAT5B	ENST00000293328	missense_variant	p.Tyr665Phe/c.1994A>T
UPN149	17	40362212	G	C	996	1	998	0.10 %	313	107	421	25.42 %	STAT5B	ENST00000293328	missense_variant	p.Thr628Ser/c.1883C>G
UPN149	9	139390648	CAG	C	1316	0	1320	0.00 %	2252	282	2550	11.06 %	NOTCH1	ENST00000277541	frameshift_variant	p.Pro2514fs/c.7541_7542delCT
UPN149	9	139390734	G	T	1406	0	1406	0.00 %	2949	105	3058	3.43 %	NOTCH1	ENST00000277541	stop_gained	p.Ser2486*/c.7457C>A
UPN149	9	139399350	C	G	1596	2	1601	0.12 %	3313	84	3406	2.47 %	NOTCH1	ENST00000277541	missense_variant	p.Arg1598Pro/c.4793G>C

UPN150	9	139391008	G	A	2363	2	2370	0.08 %	1361	1200	2564	46.80 %	NOTCH1	ENST00000277541	stop_gained	p.Gln2395*/c.7183C>T
UPN150	9	139399389	A	G	2179	1	2180	0.05 %	1178	1026	2208	46.47 %	NOTCH1	ENST00000277541	missense_variant	p.Leu1585Pro/c.4754T>C
UPN150	14	99640612	T	C	1849	0	1851	0.00 %	1068	855	1928	44.35 %	BCL11B	ENST00000357195;ENST00000345514;ENST00000443726	missense_variant	p.Tyr854Cys/c.2561A>G;p.Tyr783Cys/c.2348A>G;p.Tyr660Cys/c.1979A>G
UPN150	19	10940881	G	GCC	1486	0	1488	0.00 %	1642	550	1653	33.27 %	DNM2	ENST00000314646;ENST00000585892;ENST00000359692;ENST00000389253;ENST00000355667;E	frameshift_variant	p.Leu793fs/c.2376_2377dupCC;p.Leu789fs/c.2364_2365dupCC;p.Leu81fs/c.240_241dupCC
UPN151	9	139390694	G	GCTCAC	1908	4	1914	0.21 %	2506	1021	2522	40.48 %	NOTCH1	ENST00000277541	frameshift_variant+s	p.Ser2499fs/c.7496_7497insGTGAG
UPN151	10	89717672	C	GA	1406	0	1413	0.00 %	526	307	840	36.55 %	PTEN	ENST00000371953	frameshift_variant+	p.Arg233fs/c.697delCinsGA
UPN151	10	89711972	AGAT	A	1298	4	1306	0.31 %	589	312	906	34.44 %	PTEN	ENST00000371953	inframe_deletion	p.Met199del/c.595_597delATG
UPN151	10	89717712	C	GGGGG	1549	0	1553	0.00 %	923	50	975	5.13 %	PTEN	ENST00000371953	frameshift_variant+	p.Pro246fs/c.737delCinsGGGGG
UPN152	10	89717695	CTT	C	1513	0	1517	0.00 %	87	61	148	41.22 %	PTEN	ENST00000371953	frameshift_variant	p.Phe241fs/c.722_723delTT
UPN152	3	178936082	G	A	1463	1	1464	0.07 %	472	129	602	21.43 %	PIK3CA	ENST00000263967	missense_variant	p.Glu542Lys/c.1624G>A
UPN152	5	67591139	GACCAAT	G	1225	0	1231	0.00 %	411	92	503	18.29 %	PIK3R1	ENST00000396611;ENST00000521381;ENST00000521657;ENST00000274335;ENST00000320694;E	inframe_deletion	p.Gln579_Tyr580del/c.1735_1740delCAATAC;p.Gln279_Tyr280del/c.835_840delCAATAC;p.Gln309_Tyr310del/c.925_930delCAATAC;p.Gln216_Tyr217del/c.646_651delCAATAC
UPN152	9	139390721	G	T	2976	1	2982	0.03 %	791	175	970	18.04 %	NOTCH1	ENST00000277541	stop_gained	p.Tyr2490*/c.7470C>A
UPN152	9	139399344	A	G	3120	0	3129	0.00 %	848	184	1032	17.83 %	NOTCH1	ENST00000277541	missense_variant	p.Leu1600Pro/c.4799T>C
UPN153	17	40359659	T	A	1366	1	1370	0.07 %	1303	252	1561	16.14 %	STAT5B	ENST00000293328	missense_variant	p.Tyr665Phe/c.1994A>T
UPN154	9	139399422	A	G	2028	0	2036	0.00 %	2323	643	2978	21.59 %	NOTCH1	ENST00000277541	missense_variant	p.Leu1574Pro/c.4721T>C
UPN154	9	139399389	A	G	2002	0	2004	0.00 %	2296	566	2867	19.74 %	NOTCH1	ENST00000277541	missense_variant	p.Leu1585Pro/c.4754T>C
UPN154	9	139390744	T	C	1729	1	1734	0.06 %	2601	41	2651	1.55 %	NOTCH1	ENST00000277541	missense_variant	p.Thr2483Ala/c.7447A>G
UPN155	4	153247366	C	T	1165	0	1166	0.00 %	847	539	1389	38.80 %	FBXW7	ENST00000281708;ENST00000296555;ENST00000263981;ENST00000603548;ENST00000393956;E	missense_variant	p.Arg479Gln/c.1436G>A;p.Arg361Gln/c.1082G>A;p.Arg399Gln/c.1196G>A;p.Arg303Gln/c.908G>A
UPN155	12	25398284	C	A	1304	0	1305	0.00 %	934	505	1439	35.09 %	KRAS	ENST00000256078;ENST00000311936;ENST00000557334;E	missense_variant	p.Gly12Val/c.35G>T
UPN155	9	139399389	A	G	1666	3	1675	0.18 %	1026	379	1409	26.90 %	NOTCH1	ENST00000277541	missense_variant	p.Leu1585Pro/c.4754T>C
UPN155	4	153245437	C	T	1256	0	1259	0.00 %	1233	381	1619	23.53 %	FBXW7	ENST00000281708;ENST00000296555;ENST00000263981;ENST00000603548;ENST00000393956;E	missense_variant	p.Ser585Asn/c.1754G>A;p.Ser467Asn/c.1400G>A;p.Ser505Asn/c.1514G>A;p.Ser409Asn/c.1226G>A

UPN155	16	9014211	CTTACGC	CTACCGGCCTTTACTTGTTCGGCTCTCAA	1125	6	1127	0.53 %	807	141	809	17.43 %	USP7	ENST00000344836;ENST00000381886;ENST00000535863;ENST00000563085;ENST00000542333;E	frameshift_variant+splice_donor_variant+missense_variant+splice_region_variant+intron_variant	p.Ala204fs/c.610_611+3delGCGTAinsTTGAGAGCCGAACAAGTAAAGGCCGGT;p.Ala188fs/c.562_563+3delGCGTAinsTTGAGAGCCGAACAAGTAAAGGCCGGT;p.Ala105fs/c.313_314+3delGCGTAinsTT
UPN155	4	153244138	C	T	1431	0	1437	0.00 %	1726	158	1891	8.36 %	FBXW7	ENST00000281708;ENST00000296555;ENST00000263981;ENST00000603548;ENST00000393956;E	stop_gained	p.Trp673*/c.2019G>A;p.Trp555*/c.1665G>A;p.Trp593*/c.1779G>A;p.Trp497*/c.1491G>A
UPN156	17	40461432	G	A	NA	NA	NA	NA	114	49	163	30.06 %	STAT5A	ENST00000345506;ENST00000590949;ENST00000546010;ENST00000452307;ENST00000588868;E	missense_variant	p.Ala718Thr/c.2152G>A;p.Ala688Thr/c.2062G>A;p.Ala715Thr/c.2143G>A;p.Ala687Thr/c.2059G>A;p.Ala206Thr/c.616G>A
UPN156	17	40461405	G	GC	NA	NA	NA	NA	118	21	120	17.50 %	STAT5A	ENST00000345506;ENST00000590949;ENST00000546010;ENST00000452307;ENST00000588868;E	frameshift_variant	p.Ser710fs/c.2126dupC;p.Ser680fs/c.2036dupC;p.Ser707fs/c.2117dupC;p.Ser679fs/c.2033dupC;p.Ser198fs/c.590dupC
UPN157	4	153247289	G	A	NA	NA	NA	NA	1144	373	1520	24.54 %	FBXW7	ENST00000281708;ENST00000296555;ENST00000263981;ENST00000603548;ENST00000393956;E	missense_variant	p.Arg505Cys/c.1513C>T;p.Arg387Cys/c.1159C>T;p.Arg425Cys/c.1273C>T;p.Arg329Cys/c.985C>T
UPN157	9	139397762	A	C	NA	NA	NA	NA	1599	496	2098	23.64 %	NOTCH1	ENST00000277541	missense_variant	p.Ile1680Ser/c.5039T>G
UPN157	9	139399344	A	G	NA	NA	NA	NA	1771	551	2350	23.45 %	NOTCH1	ENST00000277541	missense_variant	p.Leu1600Pro/c.4799T>C
UPN157	4	153247366	C	T	NA	NA	NA	NA	831	250	1082	23.11 %	FBXW7	ENST00000281708;ENST00000296555;ENST00000263981;ENST00000603548;ENST00000393956;E	missense_variant	p.Arg479Gln/c.1436G>A;p.Arg361Gln/c.1082G>A;p.Arg399Gln/c.1196G>A;p.Arg303Gln/c.908G>A
UPN157	9	139390636	G	A	NA	NA	NA	NA	1400	421	1825	23.07 %	NOTCH1	ENST00000277541	stop_gained	p.Gln2519*/c.7555C>T
UPN158	5	35874580	AGCATTTT	GAAAG	NA	NA	NA	NA	518	386	519	74.37 %	IL7R	ENST00000303115	missense_variant+disruptive_inframe_del	p.Ser246_Leu248delinsGluArg/c.736_743delAGCATTTTinsGAAAG
UPN158	19	54647469	G	A	NA	NA	NA	NA	550	441	994	44.37 %	CNOT3	ENST00000221232;ENST00000406403;ENST00000440571	missense_variant	p.Arg81His/c.242G>A;p.Arg1His/c.2G>A
UPN159	1	216371701	C	A	NA	NA	NA	NA	1015	243	1258	19.32 %	USH2A	ENST00000366943;ENST00000307340;ENST00000366942	missense_variant	p.Gly1346Val/c.4037G>T

UPN159	9	139440195	G	A	NA	NA	NA	NA	296	52	348	14.94 %	NOTCH1	ENST00000277541	missense_variant	p.Pro15Leu/c.44C>T
UPN159	9	139399400	C	CTGG	NA	NA	NA	NA	1713	155	1721	9.01 %	NOTCH1	ENST00000277541	disruptive_inframe_i	p.Pro1581_Pro1582insGln/c.4742_474
UPN159	5	35874568	TTA	AGAAGCCCCTGC	NA	NA	NA	NA	1411	138	1556	8.87 %	IL7R	ENST00000303115	missense_variant+inframe_insertion	p.Leu242delinsArgSerProCys/c.724_726delTTAinsAGAAGCCCCTGC
UPN159	X	44942738	CCTGCGGGGGTGCGTGTCGT	CTCTTGAGCCG	NA	NA	NA	NA	624	54	625	8.64 %	KDM6A	ENST00000382899;ENST00000377967;ENST00000536777;ENST00000543216;ENST00000414389;ENST00000433797	stop_gained+disruptive_inframe_deletion	p.Pro1114_Val1119delinsSerTerAla/c.3340_3358delCTGCGGGGGTGCGTGTCGTinsTCTTGAGCCG;p.Pro1107_Val1112delinsSerTerAla/c.3319_3337delCTGCGGGGGTGCGTGTCGTinsTCTTGAGCCG;p.Pro1062_Val1067delinsSerTerAla/c.3184_3202delCTGCGGGGGTGCGTGTCG
UPN159	9	139399350	C	G	NA	NA	NA	NA	1756	84	1842	4.56 %	NOTCH1	ENST00000277541	missense_variant	p.Arg1598Pro/c.4793G>C
UPN160	7	151860149	T	C	NA	NA	NA	NA	1066	480	1549	30.99 %	KMT2C	ENST00000355193;ENST00000360104;ENST00000262189;E	missense_variant	p.Asn3505Asp/c.10513A>G;p.Asn1010Asp/c.3028A>G;p.Asn91Asp/c.271A>G
UPN160	11	118344185	A	AC	NA	NA	NA	NA	958	294	977	30.09 %	KMT2A	ENST00000534358;ENST00000531904;ENST00000389506;E	frameshift_variant	p.Ser774fs/c.2318dupC;p.Ser807fs/c.2417dupC
UPN160	7	151970859	C	A	NA	NA	NA	NA	3699	393	4286	9.17 %	KMT2C	ENST00000355193;ENST00000262189;ENST00000558084	missense_variant	p.Gly315Cys/c.943G>T
UPN160	7	50450321	G	A	NA	NA	NA	NA	1221	113	1336	8.46 %	IKZF1	ENST00000331340;ENST00000359197;ENST00000440768;ENST00000343574;ENST00000357364;E	missense_variant	p.Gly169Arg/c.505G>A;p.Gly82Arg/c.244G>A
UPN160	7	50450331	C	T	NA	NA	NA	NA	1209	66	1276	5.17 %	IKZF1	ENST00000331340;ENST00000359197;ENST00000440768;ENST00000343574;ENST00000357364;E	missense_variant	p.Pro172Leu/c.515C>T;p.Pro85Leu/c.254C>T
UPN161	4	153244198	C	GG	NA	NA	NA	NA	140	810	962	84.20 %	FBXW7	ENST00000281708;ENST00000296555;ENST00000263981;ENST00000603548;ENST00000393956;E	frameshift_variant+synonymous_variant	p.Gly654fs/c.1959delGinsCC;p.Gly536fs/c.1605delGinsCC;p.Gly574fs/c.1719delGinsCC;p.Gly478fs/c.1431delGinsCC
UPN161	9	139397639	A	C	NA	NA	NA	NA	1166	1067	2235	47.74 %	NOTCH1	ENST00000277541	missense_variant	p.Val1721Gly/c.5162T>G
UPN161	14	99641837	G	A	NA	NA	NA	NA	801	720	1527	47.15 %	BCL11B	ENST00000357195;ENST00000345514;ENST00000443726	missense_variant	p.Arg446Trp/c.1336C>T;p.Arg375Trp/c.1123C>T;p.Arg252Trp/c.754C>T

UPN161	19	17943348	C	T	NA	NA	NA	NA	1013	900	1917	46.95 %	JAK3	ENST00000458235;ENST00000527670;ENST00000534444	missense_variant	p.Arg887His/c.2660G>A
UPN161	14	99641770	C	G	NA	NA	NA	NA	734	618	1354	45.64 %	BCL11B	ENST00000357195;ENST00000345514;ENST00000443726	missense_variant	p.Ser468Thr/c.1403G>C;p.Ser397Thr/c.1190G>C;p.Ser274Thr/c.821G>C
UPN162	12	122252357	C	G	NA	NA	NA	NA	216	227	450	50.44 %	SETD1B	ENST00000604567;ENST00000542440;ENST00000267197	missense_variant	p.Pro746Ala/c.2236C>G
UPN162	3	178916876	G	A	NA	NA	NA	NA	911	461	1373	33.58 %	PIK3CA	ENST00000263967;ENST00000468036	missense_variant	p.Arg88Gln/c.263G>A
UPN162	5	67591097	A	G	NA	NA	NA	NA	1103	132	1235	10.69 %	PIK3R1	ENST00000396611;ENST00000521381;ENST00000521657;ENST00000274335;ENST00000320694;E	missense_variant	p.Asn564Asp/c.1690A>G;p.Asn264Asp/c.790A>G;p.Asn294Asp/c.880A>G;p.Asn201Asp/c.601A>G
UPN163	X	133551305	T	C	NA	NA	NA	NA	486	562	1050	53.52 %	PHF6	ENST00000394292;ENST00000370803;ENST00000332070;ENST00000370799;E	missense_variant	p.Ile315Thr/c.944T>C;p.Ile314Thr/c.941T>C;p.Ile280Thr/c.839T>C
UPN163	10	89717672	C	T	NA	NA	NA	NA	718	306	1030	29.71 %	PTEN	ENST00000371953	stop_gained	p.Arg233*/c.697C>T
UPN163	10	89717674	A	ACCCTGTAATC	NA	NA	NA	NA	1013	201	1016	19.78 %	PTEN	ENST00000371953	frameshift_variant	p.Arg234fs/c.700_701insCCTGTAATCC
UPN163	9	139390734	G	A	NA	NA	NA	NA	2913	330	3246	10.17 %	NOTCH1	ENST00000277541	missense_variant	p.Ser2486Leu/c.7457C>T
UPN164	X	41204468	A	C	NA	NA	NA	NA	67	807	874	92.33 %	DDX3X	ENST00000399959;ENST00000457138	missense_variant	p.Asp354Ala/c.1061A>C;p.Asp338Ala/c.1013A>C
UPN164	X	133511767	A	AGCAGG	NA	NA	NA	NA	730	575	733	78.44 %	PHF6	ENST00000394292;ENST00000370803;ENST00000332070;ENST00000370799;ENST00000416404;E	frameshift_variant	p.His42fs/c.122_123insAGGGC
UPN164	10	89717661	CAGGACCCACACG	C	NA	NA	NA	NA	994	637	1635	38.96 %	PTEN	ENST00000371953	inframe_deletion	p.Gly230_Arg233del/c.688_699delGGACCCACACGA
UPN165	X	133527953	AAC	A	NA	NA	NA	NA	100	588	688	85.47 %	PHF6	ENST00000394292;ENST00000370803;ENST00000332070;ENST00000370799;ENST00000416404;E	frameshift_variant	p.His131fs/c.393_394delCA;p.His97fs/c.291_292delCA

UPN165	7	50459491	ATC	A	NA	NA	NA	NA	981	716	1727	41.46 %	IKZF1	ENST00000331340;ENST00000359197;ENST00000440768;ENST00000343574;ENST00000438033;E	frameshift_variant	p.Leu262fs/c.785_786delTC;p.Leu220fs/c.659_660delTC;p.Arg220fs/c.657_658delTC;p.Leu175fs/c.524_525delTC
UPN165	9	139399325	G	GCCC	NA	NA	NA	NA	1969	773	1980	39.04 %	NOTCH1	ENST00000277541	disruptive_inframe_i	p.Phe1606delinsLeuGly/c.4817_4818in
UPN166	9	139397768	A	T	NA	NA	NA	NA	443	1448	1906	75.97 %	NOTCH1	ENST00000277541	missense_variant	p.Leu1678Gln/c.5033T>A
UPN166	9	8404560	C	T	NA	NA	NA	NA	841	855	1702	50.24 %	PTPRD	ENST00000356435;ENST00000381196;ENST00000355233;ENST00000358503;ENST00000360074;ENST00000397611;ENST00000397617;ENST00000537002;E	missense_variant	p.Arg1396Gln/c.4187G>A;p.Arg990Gln/c.2969G>A;p.Arg1374Gln/c.4121G>A;p.Arg1383Gln/c.4148G>A;p.Arg986Gln/c.2957G>A;p.Arg989Gln/c.2966G>A
UPN166	1	216260093	G	A	NA	NA	NA	NA	740	664	1407	47.19 %	USH2A	ENST00000366943;ENST00000307340	missense_variant	p.Pro1652Leu/c.4955C>T
UPN166	9	139390631	C	CCACT	NA	NA	NA	NA	1887	97	1897	5.11 %	NOTCH1	ENST00000277541	frameshift_variant+s	p.Trp2520fs/c.7556_7559dupAGTG
UPN166	9	139399293	AAG	GGC	NA	NA	NA	NA	1896	43	1953	2.20 %	NOTCH1	ENST00000277541	missense_variant	p.IlePhe1616MetPro/c.4848_4850delC
UPN167	X	70362028	C	T	NA	NA	NA	NA	974	960	1936	49.59 %	MED12	ENST00000333646;ENST00000374102;ENST00000374080	missense_variant	p.Thr2168Ile/c.6503C>T;p.Thr2164Ile/c.6491C>T;p.Thr2165Ile/c.6494C>T
UPN167	4	153258983	G	A	NA	NA	NA	NA	900	733	1635	44.83 %	FBXW7	ENST00000281708;ENST00000296555;ENST00000263981;ENST00000603548;ENST00000393956;E	stop_gained	p.Arg278*/c.832C>T;p.Arg160*/c.478C>T;p.Arg198*/c.592C>T;p.Arg102*/c.304C>T
UPN167	3	178936082	G	A	NA	NA	NA	NA	875	657	1535	42.80 %	PIK3CA	ENST00000263967	missense_variant	p.Glu542Lys/c.1624G>A
UPN167	19	15285012	G	A	NA	NA	NA	NA	1367	1006	2377	42.32 %	NOTCH3	ENST00000263388	missense_variant	p.Leu1535Phe/c.4603C>T
UPN168	9	94519784	G	A	NA	NA	NA	NA	513	979	1494	65.53 %	ROR2	ENST00000375708	missense_variant	p.Thr78Met/c.233C>T
UPN168	3	178952085	A	G	NA	NA	NA	NA	631	141	775	18.19 %	PIK3CA	ENST00000263967	missense_variant	p.His1047Arg/c.3140A>G
UPN168	7	151860079	T	C	NA	NA	NA	NA	1184	257	1443	17.81 %	KMT2C	ENST00000355193;ENST00000360104;ENST00000262189;E	missense_variant	p.Asn3528Ser/c.10583A>G;p.Asn1033Ser/c.3098A>G;p.Asn114Ser/c.341A>G
UPN168	7	148525907	C	T	NA	NA	NA	NA	1000	70	1071	6.54 %	EZH2	ENST00000320356;ENST00000492143;ENST00000478654;ENST00000460911;ENST00000350995;ENST00000541220;ENST00000476773;E	missense_variant	p.Asp184Asn/c.550G>A;p.Asp175Asn/c.523G>A;p.Asp145Asn/c.433G>A;p.Asp75Asn/c.223G>A

UPN168	6	135518167	A	T	NA	NA	NA	NA	1621	45	1668	2.70 %	MYB	ENST00000341911;ENST00000526187;ENST00000528015;ENST00000528774;ENST00000534121;E	missense_variant	p.Gln424His/c.1272A>T;p.Asn393Ile/c.1178A>T;p.Asn396Ile/c.1187A>T;p.Gln421His/c.1263A>T;p.Gln408His/c.1224A>T
UPN169	19	17954247	C	T	NA	NA	NA	NA	745	1383	2132	64.87 %	JAK3	ENST00000458235;ENST00000527670;ENST00000534444	missense_variant	p.Arg121His/c.362G>A
UPN169	12	122248220	C	T	NA	NA	NA	NA	728	806	1543	52.24 %	SETD1B	ENST00000604567;ENST00000542440;ENST00000267197	missense_variant	p.Pro457Ser/c.1369C>T
UPN169	6	41903797	T	TCTCC	NA	NA	NA	NA	1661	645	1694	38.08 %	CCND3	ENST00000372991;ENST00000511642;ENST00000372987;ENST00000415497;ENST00000372988;E	frameshift_variant	p.Ser254fs/c.756_759dupGGAG;p.Ser173fs/c.513_516dupGGAG;p.Ser204fs/c.606_609dupGGAG;p.Ser58fs/c.168_171dupGGAG;p.Ser182fs/c.540_543dupGGAG;p.Glu127fs/c.376_379dupGGAG
UPN169	9	139400320	G	A	NA	NA	NA	NA	1217	683	1903	35.89 %	NOTCH1	ENST00000277541	missense_variant	p.Ala1343Val/c.4028C>T
UPN169	9	139390648	CAG	C	NA	NA	NA	NA	1325	626	1969	31.79 %	NOTCH1	ENST00000277541	frameshift_variant	p.Pro2514fs/c.7541_7542delCT
UPN169	9	139399408	GCAC	G	NA	NA	NA	NA	1700	633	2337	27.09 %	NOTCH1	ENST00000277541	inframe_deletion	p.Val1578del/c.4732_4734delGTG
UPN169	9	139397727	A	G	NA	NA	NA	NA	2102	169	2271	7.44 %	NOTCH1	ENST00000277541	missense_variant	p.Cys1692Arg/c.5074T>C
UPN169	7	151927070	C	T	NA	NA	NA	NA	5251	399	5666	7.04 %	KMT2C	ENST00000355193;ENST00000262189;ENST00000418673	missense_variant	p.Gly972Arg/c.2914G>A;p.Gly127Arg/c.379G>A
UPN169	7	151970811	T	C	NA	NA	NA	NA	6235	408	6654	6.13 %	KMT2C	ENST00000355193;ENST00000262189;ENST00000558084	missense_variant	p.Ile331Val/c.991A>G
UPN169	12	25398284	C	T	NA	NA	NA	NA	1196	73	1272	5.74 %	KRAS	ENST00000256078;ENST00000311936;ENST00000557334;E	missense_variant	p.Gly12Asp/c.35G>A
UPN169	5	67591097	A	G	NA	NA	NA	NA	1017	61	1079	5.65 %	PIK3R1	ENST00000396611;ENST00000521381;ENST00000521657;ENST00000274335;ENST00000320694;E	missense_variant	p.Asn564Asp/c.1690A>G;p.Asn264Asp/c.790A>G;p.Asn294Asp/c.880A>G;p.Asn201Asp/c.601A>G
UPN169	5	35874585	TTT	CCGCCC	NA	NA	NA	NA	1419	77	1498	5.14 %	IL7R	ENST00000303115	missense_variant+disruptive_inframe_inse	p.Leu248delinsArgPro/c.741_743delTTTinsCCGCCC
UPN170	6	41903812	C	T	NA	NA	NA	NA	1242	1091	2335	46.72 %	CCND3	ENST00000372991;ENST00000511642;ENST00000372987;ENST00000415497;ENST00000372988;E	missense_variant	p.Ala249Thr/c.745G>A;p.Ala168Thr/c.502G>A;p.Ala199Thr/c.595G>A;p.Ala53Thr/c.157G>A;p.Ala177Thr/c.529G>A;p.Ser122Asn/c.365G>A

UPN170	9	139390816	G	A	NA	NA	NA	NA	897	717	1617	44.34 %	NOTCH1	ENST00000277541	stop_gained	p.Gln2459*/c.7375C>T
UPN170	9	139399422	A	G	NA	NA	NA	NA	1110	709	1822	38.91 %	NOTCH1	ENST00000277541	missense_variant	p.Leu1574Pro/c.4721T>C
UPN170	14	99640811	TGGGCCGCCCGGGGCCC	T	NA	NA	NA	NA	760	272	1034	26.31 %	BCL11B	ENST00000357195;ENST00000345514;ENST00000443726	frameshift_variant	p.Gly783fs/c.2346_2361delGGGCCCCGGGCGGCCC;p.Gly712fs/c.2133_2148delGGGCCCCGGGCGGCCC;p.Gly589fs/c.1
UPN170	9	139399389	A	G	NA	NA	NA	NA	1582	132	1717	7.69 %	NOTCH1	ENST00000277541	missense_variant	p.Leu1585Pro/c.4754T>C
UPN170	7	148525907	C	T	NA	NA	NA	NA	1230	58	1291	4.49 %	EZH2	ENST00000320356;ENST00000492143;ENST00000478654;ENST00000460911;ENST00000350995;ENST00000541220;ENST00000476773;	missense_variant	p.Asp184Asn/c.550G>A;p.Asp175Asn/c.523G>A;p.Asp145Asn/c.433G>A;p.Asp75Asn/c.223G>A
UPN171	19	15302963	G	A	NA	NA	NA	NA	563	559	1126	49.64 %	NOTCH3	ENST00000263388;ENST00000601011	missense_variant	p.Arg163Trp/c.487C>T;p.Arg162Trp/c.484C>T
UPN171	19	17942515	G	T	NA	NA	NA	NA	602	538	1142	47.11 %	JAK3	ENST00000458235;ENST00000527670;ENST00000534444	missense_variant	p.Arg925Ser/c.2773C>A
UPN171	14	99641516	C	A	NA	NA	NA	NA	610	535	1150	46.52 %	BCL11B	ENST00000357195;ENST00000345514;ENST00000443726	stop_gained	p.Glu553*/c.1657G>T;p.Glu482*/c.1444G>T;p.Glu359*/c.1075G>T
UPN171	1	215824002	C	T	NA	NA	NA	NA	855	734	1590	46.16 %	USH2A	ENST00000366943;ENST00000307340	missense_variant	p.Gly4759Arg/c.14275G>A
UPN171	9	139399342	G	GTACTCGTAT	NA	NA	NA	NA	1161	236	1166	20.24 %	NOTCH1	ENST00000277541	inframe_insertion	p.Leu1600_His1601insIleArgVal/c.4800_4801insATACGAGTA
UPN171	9	139399344	A	G	NA	NA	NA	NA	1009	147	1159	12.68 %	NOTCH1	ENST00000277541	missense_variant	p.Leu1600Pro/c.4799T>C
UPN171	16	3828700	C	T	NA	NA	NA	NA	804	32	837	3.82 %	CREBBP	ENST00000262367;ENST00000382070;ENST00000572134	splice_donor_variant+intron_variant	c.1941+1G>A;c.1827+1G>A;c.252+1G>A
UPN172	X	133511706	GT	GGGAATTCAA	NA	NA	NA	NA	21	491	513	95.71 %	PHF6	ENST00000394292;ENST00000370803;ENST00000332070;ENST00000370799;ENST00000416404;E	frameshift_variant+missense_variant	p.Cys20fs/c.60delTinsGGAATTCAA
UPN172	19	10940819	C	T	NA	NA	NA	NA	793	680	1475	46.10 %	DNM2	ENST00000314646;ENST00000585892;ENST00000359692;ENST00000389253;ENST00000355667;E	stop_gained	p.Arg770*/c.2308C>T;p.Arg766*/c.2296C>T;p.Arg58*/c.172C>T
UPN172	9	139399422	A	G	NA	NA	NA	NA	1227	1047	2281	45.90 %	NOTCH1	ENST00000277541	missense_variant	p.Leu1574Pro/c.4721T>C

UPN172	17	7578503	C	T	NA	NA	NA	NA	1830	84	1917	4.38 %	TP53	ENST00000269305;ENST00000413465;ENST00000359597;ENST00000420246;ENST00000455263;ENST00000445888;ENST00000509690;E	missense_variant	p.Val143Met/c.427G>A;p.Val11Met/c.31G>A;p.Val50Met/c.148G>A;p.Val136Met/c.406G>A
UPN172	7	148525907	C	T	NA	NA	NA	NA	1381	45	1427	3.15 %	EZH2	ENST00000320356;ENST00000492143;ENST00000478654;ENST00000460911;ENST00000350995;ENST00000541220;ENST00000476773;E	missense_variant	p.Asp184Asn/c.550G>A;p.Asp175Asn/c.523G>A;p.Asp145Asn/c.433G>A;p.Asp75Asn/c.223G>A
UPN173	16	3831230	G	T	NA	NA	NA	NA	378	386	766	50.39 %	CREBBP	ENST00000262367;ENST00000382070	missense_variant	p.Leu551Ile/c.1651C>A;p.Leu513Ile/c.1537C>A
UPN173	9	139409753	G	A	NA	NA	NA	NA	503	491	995	49.35 %	NOTCH1	ENST00000277541	missense_variant	p.Pro668Leu/c.2003C>T
UPN173	9	139397774	A	T	NA	NA	NA	NA	558	309	868	35.60 %	NOTCH1	ENST00000277541	missense_variant	p.Val1676Asp/c.5027T>A
UPN174	14	99641813	G	T	NA	NA	NA	NA	1733	631	2367	26.66 %	BCL11B	ENST00000357195;ENST00000345514;ENST00000443726	missense_variant	p.Pro454Thr/c.1360C>A;p.Pro383Thr/c.1147C>A;p.Pro260Thr/c.778C>A
UPN174	9	139399389	A	G	NA	NA	NA	NA	2173	687	2863	24.00 %	NOTCH1	ENST00000277541	missense_variant	p.Leu1585Pro/c.4754T>C
UPN174	9	139399350	C	G	NA	NA	NA	NA	2484	530	3022	17.54 %	NOTCH1	ENST00000277541	missense_variant	p.Arg1598Pro/c.4793G>C
UPN174	9	139390930	C	GGA	NA	NA	NA	NA	2705	688	3420	20.12 %	NOTCH1	ENST00000277541	frameshift_variant+	p.Val2421fs/c.7261delGinsTCC
UPN175	X	133551319	C	T	NA	NA	NA	NA	56	454	511	88.85 %	PHF6	ENST00000394292;ENST00000370803;ENST00000332070;ENST00000370799;E	stop_gained	p.Arg320*/c.958C>T;p.Arg319*/c.955C>T;p.Arg285*/c.853C>T
UPN175	1	215808006	C	T	NA	NA	NA	NA	466	492	962	51.14 %	USH2A	ENST00000366943;ENST00000307340	missense_variant	p.Arg5031Gln/c.15092G>A
UPN175	9	139390684	G	A	NA	NA	NA	NA	558	501	1062	47.18 %	NOTCH1	ENST00000277541	stop_gained	p.Gln2503*/c.7507C>T
UPN175	3	178936082	G	A	NA	NA	NA	NA	1179	339	1519	22.32 %	PIK3CA	ENST00000263967	missense_variant	p.Glu542Lys/c.1624G>A
UPN175	10	89717672	C	T	NA	NA	NA	NA	1393	120	1516	7.92 %	PTEN	ENST00000371953	stop_gained	p.Arg233*/c.697C>T
UPN175	5	67591128	G	GTCC	NA	NA	NA	NA	1177	93	1177	7.90 %	PIK3R1	ENST00000396611;ENST00000521381;ENST00000521657;ENST00000274335;ENST00000320694;E	disruptive_inframe_insertion	p.Arg574delinsSerPro/c.1721_1722insTCC;p.Arg274delinsSerPro/c.821_822insTCC;p.Arg304delinsSerPro/c.911_912insTCC;p.Arg211delinsSerPro/c.632_633insTCC
UPN175	10	89717671	A	AG	NA	NA	NA	NA	1513	97	1516	6.40 %	PTEN	ENST00000371953	frameshift_variant	p.Arg233fs/c.696_697insG

UPN175	7	148525907	C	T	NA	NA	NA	NA	1349	51	1403	3.64 %	EZH2	ENST00000320356;ENST00000492143;ENST00000478654;ENST00000460911;ENST00000350995;ENST00000541220;ENST00000476773;E	missense_variant	p.Asp184Asn/c.550G>A;p.Asp175Asn/c.523G>A;p.Asp145Asn/c.433G>A;p.Asp75Asn/c.223G>A
UPN175	14	99641871	C	A	NA	NA	NA	NA	1258	40	1301	3.07 %	BCL11B	ENST00000357195;ENST00000345514;ENST00000443726	missense_variant	p.Lys434Asn/c.1302G>T;p.Lys363Asn/c.1089G>T;p.Lys240Asn/c.720G>T
UPN176	9	139399422	A	G	NA	NA	NA	NA	1592	582	2177	26.73 %	NOTCH1	ENST00000277541	missense_variant	p.Leu1574Pro/c.4721T>C
UPN176	16	3801796	C	A	NA	NA	NA	NA	1059	277	1339	20.69 %	CREBBP	ENST00000262367;ENST00000382070;ENST00000573517	missense_variant	p.Cys1237Phe/c.3710G>T;p.Cys1199Phe/c.3596G>T;p.Cys5Phe/c.14G>T
UPN176	9	139399389	A	G	NA	NA	NA	NA	1690	409	2099	19.49 %	NOTCH1	ENST00000277541	missense_variant	p.Leu1585Pro/c.4754T>C
UPN177	X	133527636	C	T	NA	NA	NA	NA	31	432	465	92.90 %	PHF6	ENST00000394292;ENST00000370803;ENST00000332070;ENST00000370799;ENST00000416404;E	stop_gained	p.Arg116*/c.346C>T;p.Arg82*/c.244C>T
UPN177	1	115252204	C	T	NA	NA	NA	NA	112	1012	1128	89.72 %	NRAS	ENST00000369535	missense_variant	p.Ala146Thr/c.436G>A
UPN177	7	148507485	C	A	NA	NA	NA	NA	888	681	1572	43.32 %	EZH2	ENST00000320356;ENST00000478654;ENST00000460911;ENST00000350995;ENST00000541220;E	missense_variant	p.Asp657Tyr/c.1969G>T;p.Asp601Tyr/c.1801G>T;p.Asp652Tyr/c.1954G>T;p.Asp613Tyr/c.1837G>T;p.Asp643Tyr/c.1927G>T
UPN177	19	15281484	C	T	NA	NA	NA	NA	360	215	590	36.44 %	NOTCH3	ENST00000263388	missense_variant+splice_region_variant	p.Arg1630Gln/c.4889G>A

UPN177	6	135507057	G	GTACGGTCTC	NA	NA	NA	NA	1041	351	1046	33.56 %	MYB	ENST00000341911;ENST00000339290;ENST00000367812;ENST00000463282;ENST00000525477;ENST00000533837;ENST00000316528;ENST00000442647;ENST00000367814;ENST00000527615;ENST00000420123;ENST00000525369;ENST00000524588;ENST00000525002;ENST00000525940;ENST00000526187;ENST00000526565;ENST00000528015;ENST00000528140;ENST00000528345;ENST00000528774;ENST00000529262;ENST00000531634;ENST00000531737;ENST00000533384;ENST00000533624;E	disruptive_inframe_insertion	p.Glu14delinsValArgSerGln/c.40_41insTACGGTCTC
UPN177	10	89717712	CG	TCCCCCA	NA	NA	NA	NA	841	211	1052	20.06 %	PTEN	ENST00000371953	frameshift_variant+	p.Pro246fs/c.737_738delCGinsTCCCCC
UPN177	10	89717678	G	GTAAGGCC	NA	NA	NA	NA	1066	201	1070	18.79 %	PTEN	ENST00000371953	frameshift_variant	p.Glu235fs/c.703_704insTAAGGCC
UPN177	4	153245521	C	T	NA	NA	NA	NA	894	105	999	10.51 %	FBXW7	ENST00000281708;ENST00000296555;ENST00000263981;ENST00000603548;ENST00000393956;E	missense_variant	p.Gly557Glu/c.1670G>A;p.Gly439Glu/c.1316G>A;p.Gly477Glu/c.1430G>A;p.Gly381Glu/c.1142G>A
UPN177	10	89717672	CGA	GAGAGGG	NA	NA	NA	NA	969	98	1021	9.60 %	PTEN	ENST00000371953	frameshift_variant+missense_variant	p.Arg233fs/c.697_699delCGAinsGAGAGGG
UPN177	10	89717674	AC	AGAGGTTAAGGCT	NA	NA	NA	NA	956	78	1021	7.64 %	PTEN	ENST00000371953	frameshift_variant+missense_variant	p.Arg234fs/c.700delCinsGAGGTTAAGGCT

UPN177	7	148525907	C	T	NA	NA	NA	NA	1283	47	1332	3.53 %	EZH2	ENST00000320356;ENST00000492143;ENST00000478654;ENST00000460911;ENST00000350995;ENST00000541220;ENST00000476773;E	missense_variant	p.Asp184Asn/c.550G>A;p.Asp175Asn/c.523G>A;p.Asp145Asn/c.433G>A;p.Asp75Asn/c.223G>A
UPN178	17	7752322	C	G	NA	NA	NA	NA	903	899	1804	49.83 %	KDM6B	ENST00000254846;ENST00000448097	missense_variant	p.Pro906Ala/c.2716C>G
UPN178	16	3794922	G	A	NA	NA	NA	NA	1327	46	1380	3.33 %	CREBBP	ENST00000262367;ENST00000382070;ENST00000573517;E	stop_gained	p.Arg1319*/c.3955C>T;p.Arg1281*/c.3841C>T;p.Arg87*/c.259C>T;p.Arg192*/c.574C>T
UPN179	9	94456669	T	C	NA	NA	NA	NA	626	817	1449	56.38 %	ROR2	ENST00000375715	missense_variant	p.His697Arg/c.2090A>G
UPN179	19	11123719	T	C	NA	NA	NA	NA	791	889	1684	52.79 %	SMARCA4	ENST00000358026;ENST00000344626;ENST00000429416;ENST00000541122;ENST00000589677;ENST00000444061;ENST00000590574;E	missense_variant	p.Ile790Thr/c.2369T>C
UPN179	4	55561678	G	A	NA	NA	NA	NA	764	531	1295	41.00 %	KIT	ENST00000288135;ENST00000412167	missense_variant+splice_region_variant	p.Gly23Asp/c.68G>A
UPN179	7	148526931	C	T	NA	NA	NA	NA	684	205	890	23.03 %	EZH2	ENST00000320356;ENST00000492143;ENST00000478654;ENST00000460911;ENST00000350995;ENST00000541220;ENST00000476773;E	missense_variant	p.Glu125Lys/c.373G>A;p.Glu116Lys/c.346G>A;p.Glu86Lys/c.256G>A;p.Glu16Lys/c.46G>A
UPN179	X	44942757	G	TCC	NA	NA	NA	NA	560	101	664	15.21 %	KDM6A	ENST00000382899;ENST00000377967;ENST00000536777;ENST00000543216;ENST00000414389;E	frameshift_variant+missense_variant	p.Val1120fs/c.3358delGinsTCC;p.Val1113fs/c.3337delGinsTCC;p.Val1068fs/c.3202delGinsTCC;p.Val1034fs/c.3100delGinsTCC;p.Val710fs/c.2128delGinsTCC;p.Val755fs/c.2263delGinsTCC
UPN179	9	139399323	TTGAAG	GTCTCC	NA	NA	NA	NA	1898	221	2122	10.41 %	NOTCH1	ENST00000277541	missense_variant	p.ValPheLys1605ValGluThr/c.4815_48
UPN180	5	67591097	A	G	NA	NA	NA	NA	625	517	1144	45.19 %	PIK3R1	ENST00000396611;ENST00000521381;ENST00000521657;ENST00000274335;ENST00000320694;E	missense_variant	p.Asn564Asp/c.1690A>G;p.Asn264Asp/c.790A>G;p.Asn294Asp/c.880A>G;p.Asn201Asp/c.601A>G

UPN180	5	67575543	A	G	NA	NA	NA	NA	760	598	1361	43.94 %	PIK3R1	ENST00000396611;ENST00000521381;ENST00000521657;ENST00000274335;E	missense_variant	p.Met206Val/c.616A>G;p.Met108Val/c.322A>G
UPN180	12	122265755	C	T	NA	NA	NA	NA	923	636	1561	40.74 %	SETD1B	ENST00000604567;ENST00000542440;ENST00000267197	missense_variant	p.Arg1862Cys/c.5584C>T;p.Arg1819Cys/c.5455C>T
UPN181	12	49441773	T	C	NA	NA	NA	NA	942	884	1831	48.28 %	KMT2D	ENST00000301067	missense_variant	p.Tyr1404Cys/c.4211A>G
UPN182	9	139399389	A	G	NA	NA	NA	NA	1055	1071	2129	50.31 %	NOTCH1	ENST00000277541	missense_variant	p.Leu1585Pro/c.4754T>C
UPN182	19	54652069	C	T	NA	NA	NA	NA	1063	935	2006	46.61 %	CNOT3	ENST00000221232;ENST00000358389;ENST00000406403;E	missense_variant	p.Pro361Ser/c.1081C>T;p.Pro180Ser/c.538C>T;p.Pro282Ser/c.844C>T
UPN183	4	153249384	C	T	NA	NA	NA	NA	1073	937	2014	46.52 %	FBXW7	ENST00000281708;ENST00000296555;ENST00000263981;ENST00000603548;ENST00000393956;E	missense_variant	p.Arg465His/c.1394G>A;p.Arg347His/c.1040G>A;p.Arg385His/c.1154G>A;p.Arg289His/c.866G>A
UPN183	14	99642113	T	TG	NA	NA	NA	NA	1763	814	1782	45.68 %	BCL11B	ENST00000357195;ENST00000345514;ENST00000443726	frameshift_variant	p.Met354fs/c.1059dupC;p.Met283fs/c.846dupC;p.Met160fs/c.477dupC
UPN183	9	139440184	C	G	NA	NA	NA	NA	229	145	374	38.77 %	NOTCH1	ENST00000277541	missense_variant	p.Ala19Pro/c.55G>C
UPN183	16	9017189	GGA	TACCCCTTGT	NA	NA	NA	NA	1008	571	1582	36.09 %	USP7	ENST00000344836;ENST00000381886;ENST00000542333;ENST00000566004;ENST00000569230;E	frameshift_variant+stop_gained	p.Pro89fs/c.264_266delTCCinsACAAGGGGTA;p.Pro73fs/c.216_218delTCCinsACAAGGGGTA;p.Pro31fs/c.90_92delTCCinsACAAGGGGTA;p.Pro75fs/c.222_224delTCCinsACAAGGGGTA
UPN183	4	153332917	T	TAGAAAAG	NA	NA	NA	NA	1772	561	1776	31.59 %	FBXW7	ENST00000281708;ENST00000603548;ENST00000603841;ENST00000604872;E	frameshift_variant	p.Arg14fs/c.38_39insCTTTTCT
UPN183	9	139399366	G	GGCC	NA	NA	NA	NA	2525	146	2530	5.77 %	NOTCH1	ENST00000277541	inframe_insertion	p.Phe1592_Leu1593insGly/c.4776_477
UPN184	9	139397768	A	G	NA	NA	NA	NA	1447	307	1758	17.46 %	NOTCH1	ENST00000277541	missense_variant	p.Leu1678Pro/c.5033T>C
UPN184	6	41903730	TGGCTGCTGGAGCCCCGGGGGGCTTTGGGCGCTG	T	NA	NA	NA	NA	1463	93	1559	5.97 %	CCND3	ENST00000372991;ENST00000511642;ENST00000372987;ENST00000415497;ENST00000372988;ENST00000414200;E	disruptive_inframe_deletion	p.Pro265_Ser275del/c.794_826delCAGCGCCCAAAGCCCCCCGGGGCTCCAGCAGCC;p.Pro184_Ser194del/c.551_583delCAGCGCCCAAAGCCCCCCGGGGCTCCAGCAGCC;p.Pro215_Ser225del/c.644_676delCAGCGCCCAAAGCCCCCCGGGGCT

ID	Chr	Pos	Ref	Alt	Germline #REF	Germline #ALT	Germline DP	Germline VAF	Tumor #REF	Tumor #ALT	Tumor DP	Tumor VAF	Gene	ENST	Type	Variant
UPN185_p	10	89717672	C	GG	1245	0	1246	0.00 %	161	122	416	29.33 %	PTEN	ENST00000371953	frameshift_variant+	p.Arg233fs/c.697delCinsGG
UPN185_p	10	89717672	C	AG	1245	0	1246	0.00 %	161	122	416	29.33 %	PTEN	ENST00000371953	frameshift_variant+s	p.Arg234fs/c.697delCinsAG
UPN185_p	3	178936082	G	A	1043	2	1048	0.19 %	359	23	382	6.02 %	PIK3CA	ENST00000263967	missense_variant	p.Glu542Lys/c.1624G>A
UPN186_p	5	67589609	GAAA	G	649	0	650	0.00 %	294	25	295	8.47 %	PIK3R1	ENST00000396611,ENST00000521381,ENST00000521657,ENST00000274335,ENST00000320694,ENST00000521409,ENST00000336483,E	disruptive_inframe_deletion	p.Lys459del/c.1376_1378delAAA;p.Lys159del/c.476_478delAAA;p.Lys96del/c.287_289delAAA;p.Lys189del/c.566_568delAAA;p.Lys132del/c.395_397delAAA
UPN187_p	12	49420940	G	GCCTT	1014	0	1030	0.00 %	194	44	196	22.45 %	KMT2D	ENST00000301067	frameshift_variant	p.Pro4937fs/c.14808_14809insAAGG
UPN187_p	5	67589589	AATAT	GTTCCCAA	748	4	749	0.53 %	289	41	329	12.46 %	PIK3R1	ENST00000396611;ENST00000521381;ENST00000521657;ENST00000274335;ENST00000320694;ENST00000521409;ENST00000336483;E	missense_variant+inframe_insertion	p.Glu451_Tyr452delinsGlySerGln/c.1352_1356delAATATinsGTTCCCAA;p.Glu151_Tyr152delinsGlySerGln/c.452_456delAATATinsGTTCCCAA;p.Glu88_Tyr89delinsGlySerGln/c.263_267delAATATinsGTTCCCAA;p.Glu181_Tyr182delinsGlySerGln/c.542_546delAATATinsGTTCCCAA;p.Glu124_Tyr125delinsG
UPN187_p	9	139396275	T	G	1443	11	1457	0.75 %	485	21	507	4.14 %	NOTCH1	ENST00000277541	missense_variant	p.Met1855Leu/c.5563A>C
UPN188_p	X	41206193	AGA	CGAGCCCACG	578	0	578	0.00 %	67	238	320	74.38 %	DDX3X	ENST00000399959;ENST00000457138	frameshift_variant+missense_variant	p.Glu566fs/c.1697_1699delAGAinsCGAGCCCACG;p.Glu550fs/c.1649_1651delAGAins
UPN188_p	4	153249456	C	T	1340	0	1341	0.00 %	687	577	1265	45.61 %	FBXW7	ENST00000281708;ENST00000296555;ENST00000263981;ENST00000603548;ENST00000393956;E	missense_variant	p.Arg441Gln/c.1322G>A;p.Arg323Gln/c.968G>A;p.Arg361Gln/c.1082G>A;p.Arg265Gln/c.794G>A
UPN188_p	19	54649666	T	TC	1734	13	1743	0.75 %	1187	361	1202	30.03 %	CNOT3	ENST00000221232;ENST00000358389;ENST00000406403;ENST00000440571	frameshift_variant	p.Pro244fs/c.728dupC;p.Pro63fs/c.185dupC;p.Pro165fs/c.491dupC
UPN188_p	9	139399389	A	G	1593	1	1596	0.06 %	1171	343	1518	22.60 %	NOTCH1	ENST00000277541	missense_variant	p.Leu1585Pro/c.4754T>C
UPN188_p	16	9014214	A	ACGGTCCAGTGGTC	936	0	938	0.00 %	721	158	731	21.61 %	USP7	ENST00000344836;ENST00000563961;ENST00000381886;ENST00000535863;ENST00000565455;ENST00000563085;E	splice_donor_variant+intron_variant	c.611+1_611+2insGACCACTGGACCG;c.*196+1_*196+2insGACCACTGGACCG;c.563+1_563+2insGACCACTGGACCG;c.314+1_314+2insGACCACTGGACCG;c.*594+1_*594+2insGACCACTGGACCG;c.437+1_437+2insGACCACTGGACCG

UPN188_p	4	153258983	G	A	907	0	907	0.00 %	631	148	781	18.95 %	FBXW7	ENST00000281708;ENST00000296555;ENST00000263981;ENST00000603548;ENST00000393956;E	stop_gained	p.Arg278*/c.832C>T;p.Arg160*/c.478C>T;p.Arg198*/c.592C>T;p.Arg102*/c.304C>T
UPN188_p	4	153249520	G	A	1182	0	1182	0.00 %	874	194	1069	18.15 %	FBXW7	ENST00000281708;ENST00000296555;ENST00000263981;ENST00000603548;ENST00000393956;E	missense_variant	p.His420Tyr/c.1258C>T;p.His302Tyr/c.904C>T;p.His340Tyr/c.1018C>T;p.His244Tyr/c.730C>T
UPN188_p	1	9779982	T	C	1305	0	1306	0.00 %	794	100	895	11.17 %	PIK3CD	ENST00000361110;ENST00000536656;ENST00000377346;ENST00000543390	missense_variant	p.Cys381Arg/c.1141T>C;p.Cys416Arg/c.1246T>C;p.Cys83Arg/c.247T>C
UPN188_p	1	9787030	G	A	1409	0	1412	0.00 %	873	28	904	3.10 %	PIK3CD	ENST00000361110;ENST00000536656;ENST00000377346	missense_variant	p.Glu1045Lys/c.3133G>A;p.Glu1021Lys/c.3061G>A
UPN189_p	16	9012969	G	TTA	1556	0	1581	0.00 %	1388	518	1914	27.06 %	USP7	ENST00000344836;ENST00000381886;ENST00000535863;ENST00000563085;E	frameshift_variant+stop_gained	p.Val214fs/c.639delCinsTAA;p.Val198fs/c.591delCinsTAA;p.Val115fs/c.342delCinsTAA;p.Val156fs/c.465delCinsTAA
UPN189_p	9	139399292	GAAGATCATCT	GATGTGAC	1737	15	1748	0.86 %	2253	138	2277	6.06 %	NOTCH1	ENST00000277541	missense_variant+disruptive_inframe_del	p.Gln1614_Phe1617delinsArgHisIle/c.4841_4849delAGATGATCTinsGTCACA
UPN189_p	5	35873684	AAA	GTGATGAACTTCACC	1154	7	1156	0.61 %	1533	88	1623	5.42 %	IL7R	ENST00000303115;ENST00000343305;ENST00000506850;ENST00000505093	missense_variant+inframe_insertion	p.Lys214delinsValMetAsnPheThr/c.640_642delAAAinsGTGATGAACTTCACC;p.Lys17delinsValMetAsnPheThr/c.49_51delAAAinsGTGATGAACTTCACC
UPN189_p	4	153247289	G	A	1523	0	1527	0.00 %	2293	26	2321	1.12 %	FBXW7	ENST00000281708;ENST00000296555;ENST00000263981;ENST00000603548;ENST00000393956;E	missense_variant	p.Arg505Cys/c.1513C>T;p.Arg387Cys/c.1159C>T;p.Arg425Cys/c.1273C>T;p.Arg329Cys/c.985C>T
UPN190_p	1	9779982	T	C	1613	0	1614	0.00 %	219	136	357	38.10 %	PIK3CD	ENST00000361110;ENST00000536656;ENST00000377346;ENST00000543390	missense_variant	p.Cys381Arg/c.1141T>C;p.Cys416Arg/c.1246T>C;p.Cys83Arg/c.247T>C
UPN190_p	9	139390684	G	A	2051	0	2056	0.00 %	502	217	720	30.14 %	NOTCH1	ENST00000277541	stop_gained	p.Gln2503*/c.7507C>T
UPN190_p	9	139399408	GCAC	G	2018	9	2029	0.44 %	341	119	461	25.81 %	NOTCH1	ENST00000277541	inframe_deletion	p.Val1578del/c.4732_4734delGTG

UPN190_p	7	148525907	C	T	1054	2	1057	0.19 %	714	40	756	5.29 %	EZH2	ENST00000320356;ENST00000492143;ENST00000478654;ENST00000460911;ENST00000350995;ENST00000541220;ENST00000476773;E	missense_variant	p.Asp184Asn/c.550G>A;p.Asp175Asn/c.523G>A;p.Asp145Asn/c.433G>A;p.Asp75Asn/c.223G>A
UPN190_p	7	151919130	T	C	990	0	992	0.00 %	1116	41	1159	3.54 %	KMT2C	ENST00000355193;ENST00000262189	missense_variant	p.Glu1152Gly/c.3455A>G
UPN190_p	6	135518167	A	T	1572	0	1575	0.00 %	2134	52	2188	2.38 %	MYB	ENST00000341911;ENST00000526187;ENST00000528015;ENST00000528774;ENST00000534121;E	missense_variant	p.Gln424His/c.1272A>T;p.Asn393Ile/c.1178A>T;p.Asn396Ile/c.1187A>T;p.Gln421His/c.1263A>T;p.Gln408His/c.1224A>T
UPN190_p	7	151945499	C	T	1277	0	1278	0.00 %	1674	40	1718	2.33 %	KMT2C	ENST00000355193;ENST00000262189;ENST00000558084	missense_variant	p.Glu674Lys/c.2020G>A
UPN190_p	1	215953220	G	T	1423	1	1426	0.07 %	1236	23	1260	1.83 %	USH2A	ENST00000366943;ENST00000307340	missense_variant	p.Thr3635Asn/c.10904C>A
UPN191_p	13	77720322	G	A	1118	2	1123	0.18 %	99	30	129	23.26 %	MYCBP2	ENST00000407578;ENST00000544440;ENST00000357337	missense_variant	p.Arg2388Cys/c.7162C>T;p.Arg2350Cys/c.7048C>T
UPN191_p	1	9775907	G	A	1361	0	1366	0.00 %	361	71	433	16.40 %	PIK3CD	ENST00000361110;ENST00000536656;ENST00000377346	missense_variant+splice_region_variant	p.Gly124Asp/c.371G>A
UPN191_p	10	89624227	A	C	1387	0	1391	0.00 %	326	50	376	13.30 %	PTEN	ENST00000371953	initiator_codon_vari	p.Met1?/c.1A>C
UPN191_p	4	55603417	G	T	1407	2	1410	0.14 %	202	20	222	9.01 %	KIT	ENST00000288135;ENST00000412167	stop_gained	p.Glu925*/c.2773G>T;p.Glu921*/c.2761G>T
UPN191_p	1	9780550	G	A	1388	0	1388	0.00 %	277	26	304	8.55 %	PIK3CD	ENST00000361110;ENST00000536656	missense_variant	p.Arg475His/c.1424G>A
UPN191_p	1	216061861	G	T	1537	0	1538	0.00 %	239	20	259	7.72 %	USH2A	ENST00000366943;ENST00000307340	missense_variant	p.Asn2710Lys/c.8130C>A
UPN192_p	17	40354811	C	A	2081	4	2091	0.19 %	341	597	942	63.38 %	STAT5B	ENST00000293328	missense_variant	p.Gly698Val/c.2093G>T
UPN192_p	17	40359729	T	G	1161	1	1162	0.09 %	196	287	483	59.42 %	STAT5B	ENST00000293328	missense_variant	p.Asn642His/c.1924A>C
UPN192_p	7	151945060	G	A	4639	26	4678	0.56 %	2326	42	2372	1.77 %	KMT2C	ENST00000355193;ENST00000262189;ENST00000558084;ENST00000418673	missense_variant	p.Thr820Ile/c.2459C>T;p.Thr15Ile/c.44C>T
UPN193_p	no Variants															
UPN194_p	10	89692905	G	A	1103	2	1108	0.18 %	758	381	1143	33.33 %	PTEN	ENST00000371953	missense_variant	p.Arg130Gln/c.389G>A
UPN194_p	10	89717670	C	CA	1168	0	1169	0.00 %	1099	348	1107	31.44 %	PTEN	ENST00000371953	frameshift_variant	p.Arg233fs/c.696dupA

UPN194_p	1	9787030	G	A	1518	0	1520	0.00 %	691	48	739	6.50 %	PIK3CD	ENST00000361110;ENST00000536656;ENST00000377346	missense_variant	p.Glu1045Lys/c.3133G>A;p.Glu1021Lys/c.3061G>A
UPN195_p	3	178936082	G	A	1057	0	1058	0.00 %	1481	227	1712	13.26 %	PIK3CA	ENST00000263967	missense_variant	p.Glu542Lys/c.1624G>A
UPN195_p	3	178927980	T	C	726	0	728	0.00 %	1049	132	1183	11.16 %	PIK3CA	ENST00000263967	missense_variant	p.Cys420Arg/c.1258T>C
UPN195_p	5	67591097	A	G	1117	0	1119	0.00 %	1435	168	1603	10.48 %	PIK3R1	ENST00000396611;ENST00000521381;ENST00000521657;ENST00000274335;ENST00000320694;ENST00000336483;E	missense_variant	p.Asn564Asp/c.1690A>G;p.Asn264Asp/c.790A>G;p.Asn294Asp/c.880A>G;p.Asn201Asp/c.601A>G
UPN195_p	3	178936091	G	A	1033	1	1036	0.10 %	1662	37	1699	2.18 %	PIK3CA	ENST00000263967	missense_variant	p.Glu545Lys/c.1633G>A
UPN196_p	4	153249384	C	T	1797	0	1801	0.00 %	700	417	1117	37.33 %	FBXW7	ENST00000281708;ENST00000296555;ENST00000263981;ENST00000603548;ENST00000393956;E	missense_variant	p.Arg465His/c.1394G>A;p.Arg347His/c.1040G>A;p.Arg385His/c.1154G>A;p.Arg289His/c.866G>A
UPN196_p	3	178936082	G	A	1457	0	1458	0.00 %	668	240	911	26.34 %	PIK3CA	ENST00000263967	missense_variant	p.Glu542Lys/c.1624G>A
UPN196_p	5	67591097	A	G	1431	1	1434	0.07 %	831	67	900	7.44 %	PIK3R1	ENST00000396611;ENST00000521381;ENST00000521657;ENST00000274335;ENST00000320694;ENST00000336483;E	missense_variant	p.Asn564Asp/c.1690A>G;p.Asn264Asp/c.790A>G;p.Asn294Asp/c.880A>G;p.Asn201Asp/c.601A>G
UPN197_p	17	40354460	A	T	1278	3	1283	0.23 %	23	977	1003	97.41 %	STAT5B	ENST00000293328	missense_variant	p.Val712Glu/c.2135T>A
UPN197_p	9	139399325	G	GCCT	2262	0	2267	0.00 %	1756	735	1761	41.74 %	NOTCH1	ENST00000277541	disruptive_inframe_i	p.Phe1606delinsLeuGly/c.4817_4818insAG
UPN197_p	9	139391183	G	GCTCA	2281	0	2284	0.00 %	1851	706	1854	38.08 %	NOTCH1	ENST00000277541	frameshift_variant	p.Thr2337fs/c.7004_7007dupTGAG
UPN197_p	5	35874575	C	CATGTCGGATGAGATGCCAGAT	813	0	1619	0.00 %	466	329	1268	25.95 %	IL7R	ENST00000303115	disruptive_inframe_insertion	p.Thr244_Ile245insCysArgMetArgCysGlnIle/c.731_732insATGTCGGATGAGATGCCAGAT
UPN198_p	no Variants															
UPN199_p	9	139390659	G	T	1302	1	1305	0.08 %	418	2404	2828	85.01 %	NOTCH1	ENST00000277541	missense_variant	p.Thr2511Asn/c.7532C>A
UPN199_p	1	115256528	T	G	922	1	925	0.11 %	1210	993	2209	44.95 %	NRAS	ENST00000369535	missense_variant	p.Gln61His/c.183A>C
UPN199_p	19	10940819	C	T	947	0	949	0.00 %	1473	888	2366	37.53 %	DNM2	ENST00000314646;ENST00000585892;ENST00000359692;ENST00000389253;ENST00000355667;ENST00000408974;E	stop_gained	p.Arg770*/c.2308C>T;p.Arg766*/c.2296C>T;p.Arg58*/c.172C>T
UPN199_p	10	89717727	GTGA	G	1073	0	1073	0.00 %	1298	589	1891	31.15 %	PTEN	ENST00000371953	inframe_deletion	p.Asp252del/c.754_756delGAT

UPN199_p	9	139440195	G	A	201	0	201	0.00 %	452	103	555	18.56 %	NOTCH1	ENST00000277541	missense_variant	p.Pro15Leu/c.44C>T
UPN199_p	9	139399344	A	G	1425	0	1431	0.00 %	3343	129	3479	3.71 %	NOTCH1	ENST00000277541	missense_variant	p.Leu1600Pro/c.4799T>C
UPN199_p	9	139397768	A	G	1233	1	1242	0.08 %	3020	103	3130	3.29 %	NOTCH1	ENST00000277541	missense_variant	p.Leu1678Pro/c.5033T>C
UPN200_p	12	49420055	T	C	NA	NA	NA	NA	288	236	524	45.04 %	KMT2D	ENST00000301067	missense_variant	p.Ile5232Val/c.15694A>G
UPN200_p	19	10908058	C	T	NA	NA	NA	NA	206	128	335	38.21 %	DNM2	ENST00000314646;ENST00000389253;ENST00000408974;ENST00000587830	missense_variant+splice_region_variant	p.Thr400Met/c.1199C>T;p.Thr152Met/c.455C>T
UPN200_p	19	17949108	C	T	NA	NA	NA	NA	207	127	335	37.91 %	JAK3	ENST00000458235;ENST00000527670;ENST00000534444	missense_variant	p.Met511Ile/c.1533G>A
UPN200_p	9	139391014	G	A	NA	NA	NA	NA	445	200	645	31.01 %	NOTCH1	ENST00000277541	stop_gained	p.Gln2393*/c.7177C>T
UPN200_p	19	10940842	C	CCCTGG	NA	NA	NA	NA	452	125	453	27.59 %	DNM2	ENST00000314646;ENST00000585892;ENST00000359692;ENST00000389253;ENST00000355667;ENST00000408974;E	frameshift_variant	p.Arg780fs/c.2334_2338dupTGGCC;p.Arg776fs/c.2322_2326dupTGGCC;p.Arg68fs/c.198_202dupTGGCC
UPN200_p	7	148515024	TC	T	NA	NA	NA	NA	224	58	282	20.57 %	EZH2	ENST00000320356;ENST00000478654;ENST00000460911;ENST00000350995;ENST00000541220;ENST00000476773;E	frameshift_variant	p.Gly395fs/c.1184delG;p.Gly381fs/c.1142delG;p.Gly390fs/c.1169delG;p.Gly351fs/c.1052delG
UPN201_p	1	9787030	G	A	673	0	675	0.00 %	1094	1168	2264	51.59 %	PIK3CD	ENST00000361110;ENST00000536656;ENST00000377346	missense_variant	p.Glu1045Lys/c.3133G>A;p.Glu1021Lys/c.3061G>A
UPN202_p	5	35874572	TA	GTGAGGAAGCCTCCGGGACTTGCCCT	479	6	485	1.24 %	223	43	266	16.17 %	IL7R	ENST00000303115	missense_variant+inframe_insertion	p.Leu243delinsArgGluGluAlaSerGlyThrCysPro/c.728_729delTAinsGTGAGGAAGCCTCCGGGACTTGCCCT
UPN202_p	9	139399389	A	G	735	10	754	1.33 %	1416	1063	2481	42.85 %	NOTCH1	ENST00000277541	missense_variant	p.Leu1585Pro/c.4754T>C
UPN202_p	19	10904475	G	A	501	0	502	0.00 %	211	160	372	43.01 %	DNM2	ENST00000314646;ENST00000585892;ENST00000359692;ENST00000389253;ENST00000355667;ENST00000408974;E	missense_variant	p.Gly358Arg/c.1072G>A;p.Gly110Arg/c.328G>A
UPN203_p	10	89717671	AC	CGG	804	6	808	0.74 %	110	627	737	85.07 %	PTEN	ENST00000371953	frameshift_variant+	p.Arg233fs/c.696_697delACinsCGG

UPN204_p	9	139390793	C	CAGGGGGTACGT	NA	NA	NA	NA	663	154	667	23.09 %	NOTCH1	ENST00000277541	frameshift_variant	p.Ser2467fs/c.7397_7398insACGTACCCCCT
UPN204_p	9	139397648	A	G	NA	NA	NA	NA	662	22	684	3.22 %	NOTCH1	ENST00000277541	missense_variant	p.Ile1718Thr/c.5153T>C
UPN205_p	3	178952007	A	G	773	0	774	0.00 %	1156	104	1260	8.25 %	PIK3CA	ENST00000263967	missense_variant	p.Tyr1021Cys/c.3062A>G
UPN205_p	5	67591097	A	G	783	0	784	0.00 %	829	216	1045	20.67 %	PIK3R1	ENST00000396611;ENST00000521381;ENST00000521657;ENST00000274335;ENST00000320694;ENST00000336483;E	missense_variant	p.Asn564Asp/c.1690A>G;p.Asn264Asp/c.790A>G;p.Asn294Asp/c.880A>G;p.Asn201Asp/c.601A>G
UPN205_p	16	3790511	C	G	713	86	805	######	889	760	1651	46.03 %	CREBBP	ENST00000262367,ENST00000382070,ENST00000573517,ENST00000570939	missense_variant	p.Arg1341Pro/c.4022G>C;p.Arg1303Pro/c.3908G>C;p.Arg109Pro/c.326G>C;p.Arg214Pro/c.641G>C
UPN205_p	3	178936094	C	A	651	17	668	2.54 %	1133	69	1204	5.73 %	PIK3CA	ENST00000263967	missense_variant	p.Gln546Lys/c.1636C>A
UPN206_p	3	178917478	G	A	411	0	413	0.00 %	714	37	754	4.91 %	PIK3CA	ENST00000263967	missense_variant+spl	p.Gly118Asp/c.353G>A
UPN206_p	6	41903779	G	A	638	0	642	0.00 %	802	87	890	9.78 %	CCND3	ENST00000372991;ENST00000511642;ENST00000372987;ENST00000415497;ENST00000372988;ENST00000414200;E	stop_gained	p.Gln260*/c.778C>T;p.Gln179*/c.535C>T;p.Gln210*/c.628C>T;p.Gln64*/c.190C>T;p.Gln188*/c.562C>T;p.Ser133Leu/c.398C>T
UPN207_p	1	115258748	C	A	563	2	567	0.35 %	219	188	409	45.97 %	NRAS	ENST00000369535	missense_variant	p.Gly12Cys/c.34G>T
UPN207_p	4	153249385	G	A	595	0	596	0.00 %	412	358	771	46.43 %	FBXW7	ENST00000281708;ENST00000296555;ENST00000263981;ENST00000603548;ENST00000393956;E	missense_variant	p.Arg465Cys/c.1393C>T;p.Arg347Cys/c.1039C>T;p.Arg385Cys/c.1153C>T;p.Arg289Cys/c.865C>T
UPN207_p	7	148504778	A	G	509	1	511	0.20 %	218	160	378	42.33 %	EZH2	ENST00000320356;ENST00000478654;ENST00000460911;ENST00000350995;ENST00000541220;ENST00000476773;E	missense_variant	p.Leu739Pro/c.2216T>C;p.Leu683Pro/c.2048T>C;p.Leu734Pro/c.2201T>C;p.Leu695Pro/c.2084T>C;p.Leu725Pro/c.2174T>C
UPN207_p	9	139399365	A	G	593	2	606	0.33 %	1227	305	1533	19.90 %	NOTCH1	ENST00000277541	missense_variant	p.Leu1593Pro/c.4778T>C
UPN207_p	9	139399389	A	G	554	5	564	0.89 %	1216	232	1448	16.02 %	NOTCH1	ENST00000277541	missense_variant	p.Leu1585Pro/c.4754T>C
UPN207_p	12	49436907	G	T	698	0	698	0.00 %	292	247	539	45.83 %	KMT2D	ENST00000301067	missense_variant	p.Pro1866Thr/c.5596C>A
UPN207_p	14	99641348	G	A	134	0	134	0.00 %	580	75	655	11.45 %	BCL11B	ENST00000357195;ENST00000345514;ENST00000443726	stop_gained	p.Gln609*/c.1825C>T;p.Gln538*/c.1612C>T;p.Gln415*/c.1243C>T

UPN207_p	17	40468806	C	T	458	0	459	0.00 %	338	217	555	39.10 %	STAT3	ENST00000264657;ENST00000585517;ENST00000389272;ENST00000588969;ENST00000491272;ENST00000404395;E	splice_donor_variant+intron_variant	c.2257+1G>Ac.*38+1G>Ac.1963+1G>Ac.2257+1G>An.435+1G>Ac.2254+1G>An.494+1G>A
UPN207_p	19	4048114	C	T	552	3	566	0.53 %	684	602	1287	46.78 %	ZBTB7A	ENST00000322357;ENST00000601588	missense_variant	p.Arg464His/c.1391G>A
UPN207_p	19	11096063	A	G	436	0	439	0.00 %	320	45	365	12.33 %	SMARCA4	ENST00000358026;ENST00000344626;ENST00000429416;ENST00000541122;ENST00000589677;ENST00000444061;ENST00000590574;E	missense_variant	p.Met113Val/c.337A>G
UPN207_p	19	11098557	CG	C	421	0	424	0.00 %	948	53	1003	5.28 %	SMARCA4	ENST00000358026;ENST00000344626;ENST00000429416;ENST00000541122;ENST00000589677;ENST00000444061;ENST00000590574;E	frameshift_variant	p.Gly360fs/c.1079delG
UPN208_p	1	215953220	G	T	664	1	666	0.15 %	316	20	336	5.95 %	USH2A	ENST00000366943;ENST00000307340	missense_variant	p.Thr3635Asn/c.10904C>A
UPN208_p	6	135518167	A	T	579	0	579	0.00 %	301	81	383	21.15 %	MYB	ENST00000341911;ENST00000526187;ENST00000528015;ENST00000528774;ENST00000534121;E	missense_variant	p.Gln424His/c.1272A>T;p.Asn393Ile/c.1178A>T;p.Asn396Ile/c.1187A>T;p.Gln421His/c.1263A>T;p.Gln408His/c.1224A>T
UPN208_p	7	148525907	C	T	546	1	549	0.18 %	463	29	492	5.89 %	EZH2	ENST00000320356;ENST00000492143;ENST00000478654;ENST00000460911;ENST00000350995;ENST00000541220;ENST00000476773;E	missense_variant	p.Asp184Asn/c.550G>A;p.Asp175Asn/c.523G>A;p.Asp145Asn/c.433G>A;p.Asp75Asn/c.223G>A
UPN208_p	10	89717675	CG	CAGCATTA	429	1	430	0.23 %	132	79	217	36.41 %	PTEN	ENST00000371953	stop_gained+missense_variant+inframe_in	p.Arg234delinsGlnHisTer
UPN208_p	12	122255456	C	G	541	0	541	0.00 %	131	33	164	20.12 %	SETD1B	ENST00000604567	missense_variant	p.Ser1053Trp/c.3158C>G

UPN208_p	21	36164460	A	G	127	1	128	0.78 %	59	22	81	27.16 %	RUNX1	ENST00000300305;ENST00000344691;ENST00000325074;ENST00000437180;ENST00000399240	missense_variant	p.Leu472Pro/c.1415T>C;p.Leu445Pro/c.1334T>C;p.Leu460Pro/c.1379T>C;p.Leu381Pro/c.1142T>C
UPN209_p	no Variants															
UPN210_p	10	89717672	C	CCCCTGT	749	0	751	0.00 %	835	47	837	5.62 %	PTEN	ENST00000371953	stop_gained+disruptive_inframe_insertion	p.Arg233delinsProLeuTer/c.697_698insCCCTGT
UPN211_p	no Variants															
UPN185_r	10	89717672	C	GA	1245	0	1246	0.00 %	153	1447	1629	88.83 %	PTEN	ENST00000371953	frameshift_variant+missense_variant	p.Arg233fs/c.697delCinsGA
UPN185_r	17	7577548	C	G	1525	0	1529	0.00 %	1778	54	1832	2.95 %	TP53	ENST00000269305;ENST00000413465;ENST00000359597;ENST00000420246;ENST00000455263;ENST00000445888;ENST00000509690;E	missense_variant	p.Gly245Arg/c.733G>C;p.Gly113Arg/c.337G>C;p.Gly152Arg/c.454G>C
UPN186_r	5	67589609	GAAA	G	649	0	650	0.00 %	47	6	53	11.32 %	PIK3R1	ENST00000396611,ENST00000521381,ENST00000521657,ENST00000274335,ENST00000320694,ENST00000521409,ENST00000336483,E	disruptive_inframe_deletion	p.Lys459del/c.1376_1378delAAA;p.Lys159del/c.476_478delAAA;p.Lys96del/c.287_289delAAA;p.Lys189del/c.566_568delAAA;p.Lys132del/c.395_397delAAA
UPN187_r	12	49420940	G	GCCTT	1014	0	1030	0.00 %	1031	392	1039	37.73 %	KMT2D	ENST00000301067	frameshift_variant	p.Pro4937fs/c.14808_14809insAAGG
UPN187_r	5	67589589	AATAT	GTTCCCCA	748	4	749	0.53 %	666	398	1071	37.16 %	PIK3R1	ENST00000396611;ENST00000521381;ENST00000521657;ENST00000274335;ENST00000320694;ENST00000521409;ENST00000336483;E	missense_variant+inframe_insertion	p.Glu451_Tyr452delinsGlySerGln/c.1352_1356delAATATinsGTTCCCAA;p.Glu151_Tyr152delinsGlySerGln/c.452_456delAATATinsGTTCCCAA;p.Glu88_Tyr89delinsGlySerGln/c.263_267delAATATinsGTTCCCAA;p.Glu181_Tyr182delinsGlySerGln/c.542_546delAATATinsGTTCCCAA;p.Glu124_Tyr125delinsG
UPN187_r	9	139396356	G	C	1210	0	1211	0.00 %	1086	96	1182	8.12 %	NOTCH1	ENST00000277541	missense_variant	p.Pro1828Ala/c.5482C>G
UPN188_r	X	41206193	AGA	CGAGCCCACG	578	0	578	0.00 %	50	259	321	80.69 %	DDX3X	ENST00000399959;ENST00000457138	frameshift_variant+missense_variant	p.Glu566fs/c.1697_1699delAGAinsCGAGCCCACG;p.Glu550fs/c.1649_1651delAGAins
UPN188_r	19	54649666	T	TC	1734	13	1743	0.75 %	754	223	759	29.38 %	CNOT3	ENST00000221232;ENST00000358389;ENST00000406403;ENST00000440571	frameshift_variant	p.Pro244fs/c.728dupC;p.Pro63fs/c.185dupC;p.Pro165fs/c.491dupC

UPN188_r	9	139399389	A	G	1593	1	1596	0.06 %	630	160	791	20.23 %	NOTCH1	ENST00000277541	missense_variant	p.Leu1585Pro/c.4754T>C
UPN188_r	16	9014214	A	ACGGTCCAGTGGTC	936	0	938	0.00 %	498	116	501	23.15 %	USP7	ENST00000344836;ENST00000563961;ENST00000381886;ENST00000535863;ENST00000565455;ENST00000563085;E	splice_donor_variant+intron_variant	c.611+1_611+2insGACCACTGGACCG;c.*196+1_*196+2insGACCACTGGACCG;c.563+1_563+2insGACCACTGGACCG;c.314+1_314+2insGACCACTGGACCG;c.*594+1_*594+2insGACCACTGGACCG;c.437+1_437+2insGACCACTGGACCG
UPN188_r	4	153258983	G	A	907	0	907	0.00 %	619	79	699	11.30 %	FBXW7	ENST00000281708;ENST00000296555;ENST00000263981;ENST00000603548;ENST00000393956;E	stop_gained	p.Arg278*/c.832C>T;p.Arg160*/c.478C>T;p.Arg198*/c.592C>T;p.Arg102*/c.304C>T
UPN188_r	4	153249520	G	A	1182	0	1182	0.00 %	482	228	710	32.11 %	FBXW7	ENST00000281708;ENST00000296555;ENST00000263981;ENST00000603548;ENST00000393956;E	missense_variant	p.His420Tyr/c.1258C>T;p.His302Tyr/c.904C>T;p.His340Tyr/c.1018C>T;p.His244Tyr/c.730C>T
UPN188_r	1	9779982	T	C	1305	0	1306	0.00 %	483	37	525	7.05 %	PIK3CD	ENST00000361110;ENST00000536656;ENST00000377346;ENST00000543390	missense_variant	p.Cys381Arg/c.1141T>C;p.Cys416Arg/c.1246T>C;p.Cys83Arg/c.247T>C
UPN188_r	1	9787030	G	A	1409	0	1412	0.00 %	490	35	526	6.65 %	PIK3CD	ENST00000361110;ENST00000536656;ENST00000377346	missense_variant	p.Glu1045Lys/c.3133G>A;p.Glu1021Lys/c.3061G>A
UPN188_r	6	135518167	A	T	1320	2	1323	0.15 %	999	24	1023	2.35 %	MYB	ENST00000341911;ENST00000526187;ENST00000528015;ENST00000528774;ENST00000534121;E	missense_variant	p.Gln424His/c.1272A>T;p.Asn393Ile/c.1178A>T;p.Asn396Ile/c.1187A>T;p.Gln421His/c.1263A>T;p.Gln408His/c.1224A>T
UPN189_r	16	9012969	G	TTA	1556	0	1581	0.00 %	679	372	1057	35.19 %	USP7	ENST00000344836;ENST00000381886;ENST00000535863;ENST00000563085;E	frameshift_variant+stop_gained	p.Val214fs/c.639delCinsTAA;p.Val198fs/c.591delCinsTAA;p.Val115fs/c.342delCinsTAA;p.Val156fs/c.465delCinsTAA
UPN189_r	10	104852955	C	T	1278	23	1302	1.77 %	1075	157	1234	12.72 %	NT5C2	ENST00000343289;ENST00000404739;ENST00000423468;ENST00000421281	missense_variant	p.Arg367Gln/c.1100G>A;p.Arg338Gln/c.1013G>A;p.Arg67Gln/c.200G>A
UPN189_r	1	115258747	C	T	1600	19	1621	1.17 %	716	616	1336	46.11 %	NRAS	ENST00000369535	missense_variant	p.Gly12Asp/c.35G>A

UPN189_r	19	11132432	G	A	1902	46	1952	2.36 %	774	775	1551	49.97 %	SMARCA4	ENST00000358026;ENST00000344626;ENST00000429416;ENST00000541122;ENST00000589677;ENST00000444061;ENST00000590574;E	missense_variant	p.Gly883Asp/c.2648G>A
UPN189_r	17	7577548	C	T	1746	5	1754	0.29 %	490	587	1084	54.15 %	TP53	ENST00000269305;ENST00000413465;ENST00000359597;ENST00000420246;ENST00000455263;ENST00000445888;ENST00000509690;E	missense_variant	p.Gly245Ser/c.733G>A;p.Gly113Ser/c.337G>A;p.Gly152Ser/c.454G>A
UPN190_r1	9	139390863	AC	AGCTTAAGGGGACTT	1932	0	1953	0.00 %	943	503	1579	31.86 %	NOTCH1	ENST00000277541	frameshift_variant+stop_gained+missense_variant	p.Val2443fs/c.7327delGinsAAGTCCCCTTAAGC
UPN190_r1	12	122248216	G	GC	1809	59	1831	3.22 %	1503	674	1528	44.11 %	SETD1B	ENST00000604567;ENST00000542440;ENST00000267197	frameshift_variant	p.Asp458fs/c.1371dupC
UPN191_r	1	9775907	G	A	1361	0	1366	0.00 %	892	679	1573	43.17 %	PIK3CD	ENST00000361110;ENST00000536656;ENST00000377346	missense_variant+splice_region_variant	p.Gly124Asp/c.371G>A
UPN191_r	1	65311261	C	T	1425	0	1428	0.00 %	315	370	686	53.94 %	JAK1	ENST00000342505	missense_variant	p.Asp684Asn/c.2050G>A
UPN191_r	7	152027764	A	C	1276	0	1277	0.00 %	552	38	591	6.43 %	KMT2C	ENST00000355193;ENST00000262189;ENST00000558084;ENST00000452749	missense_variant	p.Leu104Arg/c.311T>G;p.Leu105Arg/c.314T>G
UPN191_r	1	215953220	G	T	1517	1	1521	0.07 %	574	30	604	4.97 %	USH2A	ENST00000366943;ENST00000307340	missense_variant	p.Thr3635Asn/c.10904C>A
UPN191_r	7	148525907	C	T	1111	2	1115	0.18 %	826	30	861	3.48 %	EZH2	ENST00000320356;ENST00000492143;ENST00000478654;ENST00000460911;ENST00000350995;ENST00000541220;ENST00000476773;E	missense_variant	p.Asp184Asn/c.550G>A;p.Asp175Asn/c.523G>A;p.Asp145Asn/c.433G>A;p.Asp75Asn/c.223G>A
UPN192_r	17	40354811	C	A	2081	4	2091	0.19 %	199	1515	1716	88.29 %	STAT5B	ENST00000293328	missense_variant	p.Gly698Val/c.2093G>T
UPN192_r	17	40359729	T	G	1161	1	1162	0.09 %	112	860	973	88.39 %	STAT5B	ENST00000293328	missense_variant	p.Asn642His/c.1924A>C
UPN192_r	9	139407844	C	T	1660	0	1664	0.00 %	1305	37	1345	2.75 %	NOTCH1	ENST00000277541	missense_variant+spl	p.Gly785Ser/c.2353G>A

UPN192_r	10	104852955	C	T	1451	0	1454	0.00 %	1059	234	1293	18.10 %	NT5C2	ENST00000343289;ENST00000404739;ENST00000423468;ENST00000421281	missense_variant	p.Arg367Gln/c.1100G>A;p.Arg338Gln/c.1013G>A;p.Arg67Gln/c.200G>A
UPN192_r	9	139391008	G	A	2341	0	2348	0.00 %	1717	399	2118	18.84 %	NOTCH1	ENST00000277541	stop_gained	p.Gln2395*/c.7183C>T
UPN192_r	1	65344786	T	C	1441	0	1444	0.00 %	1196	270	1467	18.40 %	JAK1	ENST00000342505	missense_variant	p.Asn84Ser/c.251A>G
UPN192_r	9	139395276	C	T	1641	2	1646	0.12 %	1276	94	1375	6.84 %	NOTCH1	ENST00000277541	missense_variant	p.Ala1888Thr/c.5662G>A
UPN192_r	4	153247246	T	C	1639	0	1644	0.00 %	1587	56	1644	3.41 %	FBXW7	ENST00000281708;ENST00000296555;ENST00000263981;ENST00000603548;ENST00000393956;E	missense_variant	p.Tyr519Cys/c.1556A>G;p.Tyr401Cys/c.1202A>G;p.Tyr439Cys/c.1316A>G;p.Tyr343Cys/c.1028A>G
UPN192_r	19	4054080	C	T	1983	1	1990	0.05 %	1676	42	1722	2.44 %	ZBTB7A	ENST00000322357;ENST00000601588	missense_variant	p.Cys384Tyr/c.1151G>A
UPN193_r	9	8331661	C	A	1107	0	1108	0.00 %	1241	109	1353	8.06 %	PTPRD	ENST00000356435;ENST00000381196;ENST00000355233;ENST00000358503;ENST00000360074;ENST00000397611;ENST00000397617;ENST00000537002;ENST00000540109;E	stop_gained	p.Gly1819*/c.5455G>T;p.Gly1413*/c.4237G>T;p.Gly1797*/c.5389G>T;p.Gly1806*/c.5416G>T;p.Gly1409*/c.4225G>T;p.Gly1412*/c.4234G>T

UPN193_r	6	135514995	A	G	1155	0	1161	0.00 %	1282	44	1327	3.32 %	MYB	ENST00000341911;ENST00000339290;ENST00000367812;ENST00000463282;ENST00000525477;ENST00000533837;ENST00000316528;ENST00000442647;ENST00000367814;ENST00000527615;ENST00000420123;ENST00000525369;ENST00000525002;ENST00000525940;ENST00000526187;ENST00000526565;ENST00000528015;ENST00000528140;ENST00000528345;ENST00000528774;ENST00000529262;ENST00000531737;ENST00000533384;ENST00000533624;ENST00000534044;ENST00000534121;ENST00000528343	missense_variant	p.Tyr261Cys/c.782A>G;p.Tyr237Cys/c.710A>G;p.Tyr215Cys/c.644A>G
UPN193_r	12	49420493	G	A	1631	0	1631	0.00 %	1045	905	1955	46.29 %	KMT2D	ENST00000301067	stop_gained	p.Arg5086*/c.15256C>T
UPN193_r	6	41904985	G	T	1065	2	1067	0.19 %	1274	46	1322	3.48 %	CCND3	ENST00000372991;ENST00000511642;ENST00000372987;ENST00000372988;ENST00000414200;ENST00000510503;ENST00000512426;E	missense_variant	p.Leu188Ile/c.562C>A;p.Leu107Ile/c.319C>A;p.Leu138Ile/c.412C>A;p.Leu116Ile/c.346C>A;p.Leu122Ile/c.364C>A

UPN193_r	17	7578406	C	T	1360	0	1363	0.00 %	813	971	1787	54.34 %	TP53	ENST00000269305;ENST00000413465;ENST00000359597;ENST00000420246;ENST00000455263;ENST00000445888;ENST00000509690;E	missense_variant	p.Arg175His/c.524G>A;p.Arg43His/c.128G>A;p.Arg82His/c.245G>A
UPN193_r	X	70360623	ACAGCAG	A	580	0	585	0.00 %	301	485	788	61.55 %	MED12	ENST00000333646;ENST00000374102;ENST00000374080	disruptive_inframe_deletion	p.Gln2069_Gln2070del/c.6204_6209delGCAGCA;p.Gln2065_Gln2066del/c.6192_6197delGCAGCA;p.Gln2066_Gln2067del/c.619
UPN193_r	12	122260876	CCCCGGTGCTCCTGGAGC	TCCCGGTGCTCCTGGAGT	1	0	1122	0.00 %	750	817	1573	51.94 %	SETD1B	ENST00000604567;ENST00000542440;ENST00000267197	missense_variant	p.SerProValLeuLeuGluThr1464PheProValLeuLeuGluSer/c.4391_4408delCCCCGGTGCTCCTGGAGCinsTCCCGGTGCTCCTGGAGT;p.SerProValLeuLeuGluThr1421PheProValLeu
UPN193_r	16	3817889	C	A	1080	0	1081	0.00 %	647	592	1242	47.67 %	CREBBP	ENST00000262367;ENST00000382070	stop_gained	p.Gly1028*/c.3082G>T;p.Gly990*/c.2968G>T
UPN193_r	1	216262450	T	A	949	0	954	0.00 %	612	555	1170	47.44 %	USH2A	ENST00000366943;ENST00000307340	missense_variant	p.Asp1597Val/c.4790A>T
UPN193_r	3	178936091	G	A	1075	0	1080	0.00 %	635	576	1216	47.37 %	PIK3CA	ENST00000263967	missense_variant	p.Glu545Lys/c.1633G>A
UPN193_r	19	4055223	C	T	1293	0	1294	0.00 %	860	708	1572	45.04 %	ZBTB7A	ENST00000322357;ENST00000601588	missense_variant	p.Gly3Asp/c.8G>A
UPN193_r	6	41908112	A	T	1108	1	1112	0.09 %	685	533	1220	43.69 %	CCND3	ENST00000372991;ENST00000511642;ENST00000372987;ENST00000372988;ENST00000510503;ENST00000512426;E	stop_gained	p.Leu137*/c.410T>A;p.Leu56*/c.167T>A;p.Leu87*/c.260T>A;p.Leu71*/c.212T>A
UPN194_r	10	89692905	G	A	1103	2	1108	0.18 %	928	605	1534	39.44 %	PTEN	ENST00000371953	missense_variant	p.Arg130Gln/c.389G>A
UPN194_r	19	10904475	G	A	1179	0	1186	0.00 %	1220	89	1311	6.79 %	DNM2	ENST00000314646;ENST00000585892;ENST00000359692;ENST00000389253;ENST00000355667;ENST00000408974;E	missense_variant	p.Gly358Arg/c.1072G>A;p.Gly110Arg/c.328G>A
UPN194_r	19	4053987	C	T	1781	1	1784	0.06 %	1479	670	2157	31.06 %	ZBTB7A	ENST00000322357;ENST00000601588	missense_variant	p.Cys415Tyr/c.1244G>A

UPN194_r	9	8521333	C	G	1060	3	1066	0.28 %	1612	130	1746	7.45 %	PTPRD	ENST00000356435;ENST00000381196;ENST00000355233;ENST00000358503;ENST00000360074;ENST00000397611;ENST00000397617;ENST00000537002;ENST00000540109;E	missense_variant	p.Cys302Ser/c.905G>C;p.Cys289Ser/c.866G>C;p.Cys299Ser/c.896G>C;p.Cys292Ser/c.875G>C
UPN194_r	19	10934504	G	T	1367	2	1369	0.15 %	868	475	1345	35.32 %	DNM2	ENST00000314646;ENST00000585892;ENST00000359692;ENST00000389253;ENST00000355667;E	missense_variant	p.Asp608Tyr/c.1822G>T;p.Asp604Tyr/c.1810G>T
UPN194_r	1	215820981	G	C	1344	0	1346	0.00 %	1086	583	1672	34.87 %	USH2A	ENST00000366943;ENST00000307340	missense_variant	p.Gln4892Glu/c.14674C>G
UPN194_r	9	8524966	G	A	1155	0	1159	0.00 %	1682	86	1771	4.86 %	PTPRD	ENST00000356435;ENST00000381196;ENST00000355233;ENST00000358503;ENST00000360074;ENST00000397611;ENST00000397617;ENST00000537002;ENST00000540109;E	missense_variant	p.Ala213Val/c.638C>T;p.Ala204Val/c.611C>T;p.Ala210Val/c.629C>T;p.Ala207Val/c.620C>T
UPN194_r	17	7578406	C	T	1542	2	1547	0.13 %	1264	44	1310	3.36 %	TP53	ENST00000269305;ENST00000413465;ENST00000359597;ENST00000420246;ENST00000455263;ENST00000445888;ENST00000509690;E	missense_variant	p.Arg175His/c.524G>A;p.Arg43His/c.128G>A;p.Arg82His/c.245G>A
UPN195_r	3	178936082	G	A	1057	0	1058	0.00 %	1657	69	1729	3.99 %	PIK3CA	ENST00000263967	missense_variant	p.Glu542Lys/c.1624G>A
UPN195_r	5	67591097	A	G	1117	0	1119	0.00 %	1130	424	1556	27.25 %	PIK3R1	ENST00000396611;ENST00000521381;ENST00000521657;ENST00000274335;ENST00000320694;ENST00000336483;E	missense_variant	p.Asn564Asp/c.1690A>G;p.Asn264Asp/c.790A>G;p.Asn294Asp/c.880A>G;p.Asn201Asp/c.601A>G

UPN196_r	4	153249384	C	T	1797	0	1801	0.00 %	598	417	1017	41.00 %	FBXW7	ENST00000281708;ENST00000296555;ENST00000263981;ENST00000603548;ENST00000393956;E	missense_variant	p.Arg465His/c.1394G>A;p.Arg347His/c.1040G>A;p.Arg385His/c.1154G>A;p.Arg289His/c.866G>A
UPN197_r	17	40354460	A	T	1278	3	1283	0.23 %	0	70	70	100.00 %	STAT5B	ENST00000293328	missense_variant	p.Val712Glu/c.2135T>A
UPN198_r	no data															
UPN199_r	no data															
UPN200_r	no data															
UPN201_r1	1	9787030	G	A	673	0	675	0.00 %	448	418	868	48.16 %	PIK3CD	ENST00000361110;ENST00000536656;ENST00000377346	missense_variant	p.Glu1045Lys/c.3133G>A;p.Glu1021Lys/c.3061G>A
UPN201_r1	5	67591139	GACCAAT	G	610	0	614	0.00 %	373	251	628	39.97 %	PIK3R1	ENST00000396611;ENST00000521381;ENST00000521657;ENST00000274335;ENST00000320694;ENST00000336483;E	inframe_deletion	p.Gln579_Tyr580del/c.1735_1740delCAATAC;p.Gln279_Tyr280del/c.835_840delCAATAC;p.Gln309_Tyr310del/c.925_930delCAATAC;p.Gln216_Tyr217del/c.646_651delCAATAC
UPN201_r1	10	104850748	A	C	647	1	648	0.15 %	751	29	781	3.71 %	NT5C2	ENST00000343289;ENST00000404739;ENST00000423468;ENST00000421281	missense_variant	p.Leu406Arg/c.1217T>G;p.Leu377Arg/c.1130T>G;p.Leu106Arg/c.317T>G
UPN202_r1	no data															
UPN203_r	10	89717671	AC	CGG	804	6	808	0.74 %	35	163	204	79.90 %	PTEN	ENST00000371953	frameshift_variant+missense_variant	acacga/acCGGga|p.Arg233fs/c.696_697delACinsCGG
UPN204_r	17	7577581	A	G	451	0	458	0.00 %	170	293	463	63.28 %	TP53	ENST00000269305;ENST00000413465;ENST00000359597;ENST00000420246;ENST00000455263;ENST00000445888;ENST00000509690;E	missense_variant	p.Tyr234His/c.700T>C;p.Tyr102His/c.304T>C;p.Tyr141His/c.421T>C
UPN204_r	12	25398210	C	T	449	0	449	0.00 %	61	44	106	41.51 %	KRAS	ENST00000256078;ENST00000311936;ENST00000557334;ENST00000556131	missense_variant+splice_region_variant	p.Glu37Lys/c.109G>A
UPN205_r1	3	178927464	T	TAAACGG	667	55	667	8.25 %	259	81	261	31.03 %	PIK3CA	ENST00000263967	inframe_insertion	p.Lys410_Gly411insArgLys/c.1230_1231insCGGAAA

UPN205_r1	17	40500443	C	T	764	14	779	1.80 %	242	49	291	16.84 %	STAT3	ENST00000264657,ENST00000585517,ENST00000588969,ENST00000404395,E	missense_variant	p.Arg31Gln/c.92G>A;p.Arg33Gln/c.98G>A
UPN205_r1	3	178936096	G	C	593	78	674	######	187	103	291	35.40 %	PIK3CA	ENST00000263967	missense_variant	p.Gln546His/c.1638G>C
UPN205_r1	16	3790511	C	G	713	86	805	######	208	153	364	42.03 %	CREBBP	ENST00000262367,ENST00000382070,ENST00000573517,ENST00000570939	missense_variant	p.Arg1341Pro/c.4022G>C;p.Arg1303Pro/c.3908G>C;p.Arg109Pro/c.326G>C;p.Arg214Pro/c.641G>C
UPN206_r	no Variants															
UPN207_r	X	133527636	C	T	289	0	290	0.00 %	64	45	109	41.28 %	PHF6	ENST00000394292;ENST00000370803;ENST00000332070;ENST00000370799;ENST00000416404;ENST00000370800	stop_gained	p.Arg116*/c.346C>T;p.Arg82*/c.244C>T
UPN207_r	X	41201759	G	A	239	0	239	0.00 %	33	20	54	37.04 %	DDX3X	ENST00000399959,ENST00000457138,ENST00000441189,ENST00000542215	missense_variant	cGt/cAt|p.Arg99His/c.296G>A,cGt/cAt|p.Arg83His/c.248G>A,cGt/cAt|p.Arg99His/c.296G>A,cGt/cAt|p.Arg143His/c.428G>A
UPN207_r	14	99641212	C	T	9	0	9	0.00 %	125	70	195	35.90 %	BCL11B	ENST00000357195,ENST00000345514,ENST00000443726	missense_variant	cGc/cAc|p.Arg654His/c.1961G>A,cGc/cAc|p.Arg583His/c.1748G>A,cGc/cAc|p.Arg460His/c.1379G>A
UPN207_r	19	11094865	G	A	478	2	486	0.41 %	202	96	298	32.21 %	SMARCA4	ENST00000358026;ENST00000344626;ENST00000429416;ENST00000541122;ENST00000589677;ENST00000444061;ENST00000590574;E	missense_variant	p.Arg13Gln/c.38G>A
UPN207_r	16	3900713	G	T	590	1	592	0.17 %	342	145	487	29.77 %	CREBBP	ENST00000262367;ENST00000382070	missense_variant	p.Ser128Tyr/c.383C>A
UPN207_r	13	77671522	A	G	567	0	568	0.00 %	360	143	505	28.32 %	MYCBP2	ENST00000407578;ENST00000544440;ENST00000357337	missense_variant	p.Met3256Thr/c.9767T>C;p.Met3218Thr/c.9653T>C
UPN207_r	17	7753230	C	T	616	1	618	0.16 %	477	184	666	27.63 %	KDM6B	ENST00000254846;ENST00000448097	missense_variant	p.Arg1178Trp/c.3532C>T
UPN207_r	19	17953241	C	T	379	0	380	0.00 %	241	84	331	25.38 %	JAK3	ENST00000458235;ENST00000527670;ENST00000534444	missense_variant	p.Gly249Arg/c.745G>A

UPN207_r	19	54646728	G	A	590	1	594	0.17 %	218	72	292	24.66 %	CNOT3	ENST00000221232,ENST00000406403	missense_variant	cGc/cAc|p.Arg5His/c.14G>A,cGc/cAc|p.Arg5His/c.14G>A
UPN207_r	2	16086205	G	A	548	1	551	0.18 %	205	67	273	24.54 %	MYCN	ENST00000281043	missense_variant	p.Ala461Thr/c.1381G>A
UPN207_r	19	23542988	C	T	587	3	597	0.50 %	173	56	229	24.45 %	ZNF91	ENST00000300619;ENST00000397082	missense_variant	p.Met931Ile/c.2793G>A;p.Met899Ile/c.2697G>A
UPN207_r	4	153332919	G	A	658	3	663	0.45 %	476	152	628	24.20 %	FBXW7	ENST00000281708;ENST00000603548;ENST00000603841;ENST00000604872;E	stop_gained	p.Arg13*/c.37C>T
UPN207_r	4	55597511	A	G	626	0	627	0.00 %	292	91	384	23.70 %	KIT	ENST00000288135;ENST00000412167	missense_variant	p.Glu720Gly/c.2159A>G;p.Glu716Gly/c.2147A>G
UPN207_r	14	99641630	C	T	130	0	130	0.00 %	181	56	238	23.53 %	BCL11B	ENST00000357195;ENST00000345514;ENST00000443726	missense_variant	p.Gly515Ser/c.1543G>A;p.Gly444Ser/c.1330G>A;p.Gly321Ser/c.961G>A
UPN207_r	16	3777898	G	T	510	2	514	0.39 %	1084	322	1407	22.89 %	CREBBP	ENST00000262367;ENST00000382070	missense_variant	p.His2384Asn/c.7150C>A;p.His2346Asn/c.7036C>A
UPN207_r	9	139400029	A	G	589	6	603	1.00 %	667	197	868	22.70 %	NOTCH1	ENST00000277541	missense_variant	aTc/aCc|p.Ile1440Thr/c.4319T>C
UPN207_r	14	99641270	C	T	23	0	23	0.00 %	101	28	129	21.71 %	BCL11B	ENST00000357195,ENST00000345514,ENST00000443726	missense_variant	Gac/Aac|p.Asp635Asn/c.1903G>A,Gac/Aac|p.Asp564Asn/c.1690G>A,Gac/Aac|p.Asp441Asn/c.1321G>A
UPN207_r	19	15298797	C	T	451	0	455	0.00 %	144	38	182	20.88 %	NOTCH3	ENST00000263388,ENST00000601011	missense_variant	Ggc/Agc|p.Gly501Ser/c.1501G>A,Ggc/Agc|p.Gly500Ser/c.1498G>A
UPN207_r	12	49447842	C	T	674	2	680	0.29 %	342	85	427	19.91 %	KMT2D	ENST00000301067	missense_variant	p.Ala198Thr/c.592G>A
UPN207_r	7	148506443	C	T	569	1	572	0.17 %	224	55	280	19.64 %	EZH2	ENST00000320356;ENST00000478654;ENST00000460911;ENST00000350995;ENST00000541220;ENST00000476773;E	missense_variant	p.Arg690His/c.2069G>A;p.Arg634His/c.1901G>A;p.Arg685His/c.2054G>A;p.Arg646His/c.1937G>A;p.Arg676His/c.2027G>A
UPN207_r	7	148506443	C	T	569	1	572	0.17 %	224	55	280	19.64 %	EZH2	ENST00000320356,ENST00000478654,ENST00000460911,ENST00000350995,ENST00000541220,ENST00000476773,E	missense_variant	cGt/cAt|p.Arg690His/c.2069G>A,cGt/cAt|p.Arg634His/c.1901G>A,cGt/cAt|p.Arg685His/c.2054G>A,cGt/cAt|p.Arg646His/c.1937G>A,cGt/cAt|p.Arg634His/c.1901G>A,cGt/cAt|p.Arg634His/c.1901G>A,cGt/cAt|p.Arg676His/c.2027G>A
UPN207_r	9	139399389	A	G	554	5	564	0.89 %	476	116	594	19.53 %	NOTCH1	ENST00000277541	missense_variant	p.Leu1585Pro/c.4754T>C
UPN207_r	X	70339931	G	A	352	3	356	0.84 %	280	65	345	18.84 %	MED12	ENST00000333646,ENST00000374102,ENST00000374080	missense_variant	cGg/cAg|p.Arg155Gln/c.464G>A,cGg/cAg|p.Arg155Gln/c.464G>A,cGg/cAg|p.Arg155Gln/c.464G>A

UPN207_r	4	153249456	C	T	650	0	652	0.00 %	510	116	630	18.41 %	FBXW7	ENST00000281708,ENST00000296555,ENST00000263981,ENST00000603548,ENST00000393956,E	missense_variant	cGg/cAg|p.Arg441Gln/c.1322G>A,cGg/cAg|p.Arg323Gln/c.968G>A,cGg/cAg|p.Arg361Gln/c.1082G>A,cGg/cAg|p.Arg441Gln/c.1322G>A,cGg/cAg|p.Arg265Gln/c.794G>A,cGg/cAg|p.Arg441Gln/c.1322G>A
UPN207_r	19	4054173	A	G	445	1	453	0.22 %	1382	310	1699	18.25 %	ZBTB7A	ENST00000322357,ENST00000601588	missense_variant	cTg/cCg|p.Leu353Pro/c.1058T>C,cTg/cCg|p.Leu353Pro/c.1058T>C
UPN207_r	3	178947891	G	A	532	0	533	0.00 %	325	72	399	18.05 %	PIK3CA	ENST00000263967	missense_variant	atG/atA|p.Met922Ile/c.2766G>A
UPN207_r	16	3823913	G	A	656	0	659	0.00 %	613	136	754	18.04 %	CREBBP	ENST00000262367,ENST00000382070,ENST00000571826,ENST00000572134	stop_gained	Cga/Tga|p.Arg768*/c.2302C>T,Cga/Tga|p.Arg730*/c.2188C>T,Cga/Tga|p.Arg117*/c.349C>T,Cga/Tga|p.Arg190*/c.568C>T
UPN207_r	12	122247854	G	A	655	0	660	0.00 %	499	109	612	17.81 %	SETD1B	ENST00000604567,ENST00000542440,ENST00000267197	missense_variant	Gcg/Acg|p.Ala335Thr/c.1003G>A,Gcg/Acg|p.Ala335Thr/c.1003G>A,Gcg/Acg|p.Ala335Thr/c.1003G>A
UPN207_r	1	215844615	G	A	354	0	355	0.00 %	207	43	251	17.13 %	USH2A	ENST00000366943;ENST00000307340	missense_variant	p.Ala4611Val/c.13832C>T
UPN207_r	12	49425055	C	T	715	0	717	0.00 %	1100	225	1326	16.97 %	KMT2D	ENST00000301067	missense_variant	cGg/cAg|p.Arg4478Gln/c.13433G>A
UPN207_r	14	99641430	GC	G	187	0	187	0.00 %	282	58	344	16.86 %	BCL11B	ENST00000357195,ENST00000345514,ENST00000443726	frameshift_variant	ggc/|p.Gly581fs/c.1742delG,ggc/|p.Gly510fs/c.1529delG,ggc/|p.Gly387fs/c.1160delG
UPN207_r	X	41205629	G	A	306	0	308	0.00 %	248	50	298	16.78 %	DDX3X	ENST00000399959,ENST00000457138	missense_variant	cGc/cAc|p.Arg488His/c.1463G>A,cGc/cAc|p.Arg472His/c.1415G>A
UPN207_r	13	77657276	C	T	575	1	576	0.17 %	221	44	265	16.60 %	MYCBP2	ENST00000407578,ENST00000544440,ENST00000357337,ENST00000429715	missense_variant	Ggg/Agg|p.Gly3643Arg/c.10927G>A,Ggg/Agg|p.Gly3605Arg/c.10813G>A,Ggg/Agg|p.Gly3605Arg/c.10813G>A,Ggg/Agg|p.Gly28Arg/c.82G>A
UPN207_r	16	8995938	C	T	447	0	448	0.00 %	315	63	381	16.54 %	USP7	ENST00000344836,ENST00000563961,ENST00000381886,ENST00000535863,ENST00000565455,E	splice_donor_variant+intron_variant	|c.2047+1G>Ac.*1632+1G>Ac.1999+1G>Ac.1750+1G>Ac.*2030+1G>Ac.1750+1G>A
UPN207_r	19	4054707	G	A	369	0	371	0.00 %	490	97	587	16.52 %	ZBTB7A	ENST00000322357;ENST00000601588	missense_variant	p.Ala175Val/c.524C>T
UPN207_r	1	215987203	C	T	514	0	515	0.00 %	193	38	231	16.45 %	USH2A	ENST00000366943;ENST00000307340	missense_variant	p.Arg3205His/c.9614G>A
UPN207_r	14	99642434	G	A	306	1	310	0.32 %	409	80	489	16.36 %	BCL11B	ENST00000357195,ENST00000345514,ENST00000443726	missense_variant	Cgc/Tgc|p.Arg247Cys/c.739C>T,Cgc/Tgc|p.Arg176Cys/c.526C>T,Cgc/Tgc|p.Arg53Cys/c.157C>T

UPN207_r	4	106155467	G	A	616	2	618	0.32 %	180	34	214	15.89 %	TET2	ENST00000513237,ENST00000305737,ENST00000540549,ENST00000545826,ENST00000394764,ENST00000265149,ENST00000380013,E	missense_variant	cGt/cAt|p.Arg144His/c.431G>A,cGt/cAt|p.Arg123His/c.368G>A,cGt/cAt|p.Arg123His/c.368G>A,cGt/cAt|p.Arg123His/c.368G>A,cGt/cAt|p.Arg123His/c.368G>A,cGt/cAt|p.Arg123His/c.368G>A,cGt/cAt|p.Arg123His/c.368G>A
UPN207_r	7	150706020	C	T	486	1	491	0.20 %	413	77	491	15.68 %	NOS3	ENST00000297494,ENST00000461406	splice_region_variant+synonymous_varian	gcC/gcT|p.Ala705Ala/c.2115C>T,gcC/gcT|p.Ala499Ala/c.1497C>T
UPN207_r	16	3778767	G	A	581	0	585	0.00 %	1956	363	2322	15.63 %	CREBBP	ENST00000262367,ENST00000382070	missense_variant	cCg/cTg|p.Pro2094Leu/c.6281C>T,cCg/cTg|p.Pro2056Leu/c.6167C>T
UPN207_r	12	49432632	C	T	680	0	685	0.00 %	640	117	759	15.42 %	KMT2D	ENST00000301067	missense_variant	cGc/cAc|p.Arg2836His/c.8507G>A
UPN207_r	4	153247289	G	A	592	2	595	0.34 %	362	66	429	15.38 %	FBXW7	ENST00000281708;ENST00000296555;ENST00000263981;ENST00000603548;ENST00000393956;E	missense_variant	p.Arg505Cys/c.1513C>T;p.Arg387Cys/c.1159C>T;p.Arg425Cys/c.1273C>T;p.Arg329Cys/c.985C>T
UPN207_r	7	148506221	T	C	592	0	600	0.00 %	323	57	380	15.00 %	EZH2	ENST00000320356;ENST00000478654;ENST00000460911;ENST00000350995;ENST00000541220;ENST00000476773;E	missense_variant	p.Ile713Val/c.2137A>G;p.Ile657Val/c.1969A>G;p.Ile708Val/c.2122A>G;p.Ile669Val/c.2005A>G;p.Ile699Val/c.2095A>G
UPN207_r	12	49445178	G	A	452	0	453	0.00 %	722	127	850	14.94 %	KMT2D	ENST00000301067	missense_variant	p.Pro763Leu/c.2288C>T
UPN207_r	7	151846170	C	T	565	3	571	0.53 %	286	50	337	14.84 %	KMT2C	ENST00000355193,ENST00000360104,ENST00000262189,ENST00000424877	missense_variant	aGc/aAc|p.Ser4338Asn/c.13013G>A,aGc/aAc|p.Ser1841Asn/c.5522G>A,aGc/aAc|p.Ser4281Asn/c.12842G>A,aGc/aAc|p.Ser898Asn/c.2693G>A
UPN207_r	X	133559286	C	T	349	0	350	0.00 %	52	9	61	14.75 %	PHF6	ENST00000394292,ENST00000370803,ENST00000332070,ENST00000416404	stop_gained	Cga/Tga|p.Arg343*/c.1027C>T,Cga/Tga|p.Arg342*/c.1024C>T,Cga/Tga|p.Arg342*/c.1024C>T,Cga/Tga|p.Arg308*/c.922C>T
UPN207_r	19	4054015	C	T	758	2	763	0.26 %	790	132	924	14.29 %	ZBTB7A	ENST00000322357;ENST00000601588	missense_variant	p.Gly406Ser/c.1216G>A
UPN207_r	19	15271729	C	T	486	0	491	0.00 %	823	131	957	13.69 %	NOTCH3	ENST00000263388	missense_variant	p.Arg2237Gln/c.6710G>A
UPN207_r	14	99641468	C	T	226	0	227	0.00 %	234	37	273	13.55 %	BCL11B	ENST00000357195,ENST00000345514,ENST00000443726	missense_variant	Ggc/Agc|p.Gly569Ser/c.1705G>A,Ggc/Agc|p.Gly498Ser/c.1492G>A,Ggc/Agc|p.Gly375Ser/c.1123G>A
UPN207_r	2	16082313	AC	A	563	3	568	0.53 %	877	137	1014	13.51 %	MYCN	ENST00000281043	frameshift_variant	ccg/|p.Pro45fs/c.134delC

UPN207_r	16	8993546	C	T	579	0	583	0.00 %	442	65	508	12.80 %	USP7	ENST00000344836;ENST00000381886;ENST00000535863;ENST00000563085;E	missense_variant	p.Arg793His/c.2378G>A;p.Arg777His/c.2330G>A;p.Arg694His/c.2081G>A;p.Ala45Thr/c.133G>A
UPN207_r	19	10943735	A	G	704	1	712	0.14 %	719	105	832	12.62 %	TMED1	ENST00000214869,ENST00000591818,ENST00000591695,ENST00000588289,E	missense_variant	gTg/gCg|p.Val207Ala/c.620T>C,Acc/Gcc|p.Thr37Ala/c.109A>G,Tgg/Cgg|p.Trp146Arg/c.436T>C,gTg/gCg|p.Val62Ala/c.185T>C,gTg/gCg|p.Val189Ala/c.566T>C
UPN207_r	7	151835903	C	T	574	1	577	0.17 %	390	55	446	12.33 %	KMT2C	ENST00000355193,ENST00000360104,ENST00000262189,ENST00000485655,E	missense_variant	cGg/cAg|p.Arg4931Gln/c.14792G>A,cGg/cAg|p.Arg2430Gln/c.7289G>A,cGg/cAg|p.Arg4874Gln/c.14621G>A,cGg/cAg|p.Arg79Gln/c.236G>A,cGg/cAg|p.Arg1487Gln/c.446
UPN207_r	19	4047989	C	T	49	0	50	0.00 %	482	65	547	11.88 %	ZBTB7A	ENST00000322357,ENST00000601588	missense_variant	Ggg/Agg|p.Gly506Arg/c.1516G>A,Ggg/Agg|p.Gly506Arg/c.1516G>A
UPN207_r	19	15300132	C	T	688	1	692	0.14 %	818	109	929	11.73 %	NOTCH3	ENST00000263388,ENST00000601011	missense_variant	Ggc/Agc|p.Gly382Ser/c.1144G>A,Ggc/Agc|p.Gly381Ser/c.1141G>A
UPN207_r	9	8518261	C	T	696	4	703	0.57 %	459	61	522	11.69 %	PTPRD	ENST00000356435;ENST00000381196;ENST00000355233;ENST00000358503;ENST00000360074;ENST00000397611;ENST00000397617;ENST00000537002;ENST00000540109;E	missense_variant	p.Arg377His/c.1130G>A;p.Arg364His/c.1091G>A;p.Arg374His/c.1121G>A;p.Arg367His/c.1100G>A
UPN207_r	19	10904509	G	A	392	0	393	0.00 %	347	46	394	11.68 %	DNM2	ENST00000314646;ENST00000585892;ENST00000359692;ENST00000389253;ENST00000355667;ENST00000408974;E	missense_variant	p.Arg369Gln/c.1106G>A;p.Arg121Gln/c.362G>A
UPN207_r	10	104934630	C	T	509	0	510	0.00 %	492	64	557	11.49 %	NT5C2	ENST00000343289;ENST00000404739;ENST00000452156;ENST00000461461;E	missense_variant	p.Arg29Gln/c.86G>A
UPN207_r	19	4055175	T	C	551	2	561	0.36 %	563	70	633	11.06 %	ZBTB7A	ENST00000322357,ENST00000601588	missense_variant	gAc/gGc|p.Asp19Gly/c.56A>G,gAc/gGc|p.Asp19Gly/c.56A>G
UPN207_r	16	3779608	C	T	693	3	698	0.43 %	2286	278	2577	10.79 %	CREBBP	ENST00000262367;ENST00000382070	missense_variant	p.Gly1814Arg/c.5440G>A;p.Gly1776Arg/c.5326G>A

<table>
<tr><td>UPN207_r</td><td>9</td><td>8504320</td><td>C</td><td>T</td><td>544</td><td>0</td><td>547</td><td>0.00 %</td><td>258</td><td>31</td><td>291</td><td>10.65 %</td><td>PTPRD</td><td>ENST00000356435,ENST00000381196,ENST00000355233,ENST00000358503,ENST00000360074,ENST00000397611,ENST00000397617,ENST00000537002,ENST00000540109,E</td><td>missense_variant</td><td>cGc/cAc|p.Arg588His/c.1763G>A,cGc/cAc|p.Arg588His/c.1763G>A,cGc/cAc|p.Arg588His/c.1763G>A,cGc/cAc|p.Arg575His/c.1724G>A,cGc/cAc|p.Arg575His/c.1724G>A,cGc/cAc|p.Arg585His/c.1754G>A,cGc/cAc|p.Arg578His/c.1733G>A,cGc/cAc|p.Arg585His/c.1754G>A,cGc/cAc|p.Arg588His/c.1763G>A,cGc/cAc|p.Arg588His/c.1763G>A,cGc/cAc|p.Arg578His/c.1733G>A</td></tr>
<tr><td>UPN207_r</td><td>9</td><td>139399365</td><td>A</td><td>G</td><td>593</td><td>2</td><td>606</td><td>0.33 %</td><td>590</td><td>68</td><td>660</td><td>10.30 %</td><td>NOTCH1</td><td>ENST00000277541</td><td>missense_variant</td><td>p.Leu1593Pro/c.4778T>C</td></tr>
<tr><td>UPN207_r</td><td>9</td><td>8319965</td><td>C</td><td>T</td><td>469</td><td>0</td><td>470</td><td>0.00 %</td><td>141</td><td>16</td><td>157</td><td>10.19 %</td><td>PTPRD</td><td>ENST00000356435,ENST00000381196,ENST00000355233,ENST00000358503,ENST00000360074,ENST00000397611,ENST00000397617,ENST00000537002,ENST00000540109,E</td><td>missense_variant+splice_region_variant</td><td>Gcg/Acg|p.Ala1846Thr/c.5536G>A,Gcg/Acg|p.Ala1846Thr/c.5536G>A,Gcg/Acg|p.Ala1440Thr/c.4318G>A,Gcg/Acg|p.Ala1824Thr/c.5470G>A,Gcg/Acg|p.Ala1833Thr/c.5497G>A,Gcg/Acg|p.Ala1436Thr/c.4306G>A,Gcg/Acg|p.Ala1439Thr/c.4315G>A,Gcg/Acg|p.Ala1436Thr/c.4306G>A,Gcg/Acg|p.Ala1846Thr/c.5536G>A,Gcg/Acg|p.Ala1439Thr/c.4315G>A,Gcg/Acg|p.Ala1439Thr/c.4315</td></tr>
<tr><td>UPN207_r</td><td>19</td><td>17948006</td><td>G</td><td>A</td><td>540</td><td>2</td><td>547</td><td>0.37 %</td><td>664</td><td>75</td><td>740</td><td>10.14 %</td><td>JAK3</td><td>ENST00000458235,ENST00000527670,ENST00000534444</td><td>missense_variant</td><td>gCg/gTg|p.Ala573Val/c.1718C>T,gCg/gTg|p.Ala573Val/c.1718C>T,gCg/gTg|p.Ala573Val/c.1718C>T</td></tr>
<tr><td>UPN207_r</td><td>9</td><td>94495407</td><td>G</td><td>A</td><td>407</td><td>1</td><td>413</td><td>0.24 %</td><td>259</td><td>29</td><td>289</td><td>10.03 %</td><td>ROR2</td><td>ENST00000375708,ENST00000375715</td><td>missense_variant</td><td>Cgc/Tgc|p.Arg312Cys/c.934C>T,Cgc/Tgc|p.Arg172Cys/c.514C>T</td></tr>
<tr><td>UPN207_r</td><td>1</td><td>215987234</td><td>C</td><td>T</td><td>359</td><td>1</td><td>361</td><td>0.28 %</td><td>169</td><td>19</td><td>191</td><td>9.95 %</td><td>USH2A</td><td>ENST00000366943,ENST00000307340</td><td>missense_variant</td><td>Gga/Aga|p.Gly3195Arg/c.9583G>A,Gga/Aga|p.Gly3195Arg/c.9583G>A</td></tr>
<tr><td>UPN207_r</td><td>19</td><td>15281551</td><td>C</td><td>T</td><td>449</td><td>0</td><td>451</td><td>0.00 %</td><td>1427</td><td>156</td><td>1586</td><td>9.84 %</td><td>NOTCH3</td><td>ENST00000263388</td><td>missense_variant</td><td>Gct/Act|p.Ala1608Thr/c.4822G>A</td></tr>
<tr><td>UPN207_r</td><td>16</td><td>3778363</td><td>C</td><td>T</td><td>567</td><td>2</td><td>569</td><td>0.35 %</td><td>557</td><td>59</td><td>617</td><td>9.56 %</td><td>CREBBP</td><td>ENST00000262367,ENST00000382070</td><td>missense_variant</td><td>Ggc/Agc|p.Gly2229Ser/c.6685G>A,Ggc/Agc|p.Gly2191Ser/c.6571G>A</td></tr>
<tr><td>UPN207_r</td><td>13</td><td>77760071</td><td>C</td><td>T</td><td>558</td><td>0</td><td>558</td><td>0.00 %</td><td>222</td><td>23</td><td>245</td><td>9.39 %</td><td>MYCBP2</td><td>ENST00000407578,ENST00000544440,ENST00000357337</td><td>missense_variant</td><td>cGc/cAc|p.Arg1460His/c.4379G>A,cGc/cAc|p.Arg1422His/c.4265G>A,cGc/cAc|p.Arg1422His/c.4265G>A</td></tr>
<tr><td>UPN207_r</td><td>4</td><td>106196382</td><td>G</td><td>A</td><td>654</td><td>1</td><td>661</td><td>0.15 %</td><td>478</td><td>45</td><td>524</td><td>8.59 %</td><td>TET2</td><td>ENST00000513237,ENST00000540549,ENST00000380013</td><td>missense_variant</td><td>cGg/cAg|p.Arg1593Gln/c.4778G>A,cGg/cAg|p.Arg1572Gln/c.4715G>A,cGg/cAg|p.Arg1572Gln/c.4715G>A</td></tr>
<tr><td>UPN207_r</td><td>12</td><td>49420995</td><td>AG</td><td>A</td><td>511</td><td>0</td><td>513</td><td>0.00 %</td><td>444</td><td>42</td><td>496</td><td>8.47 %</td><td>KMT2D</td><td>ENST00000301067</td><td>frameshift_variant</td><td>cct/|p.Pro4918fs/c.14753delC</td></tr>
<tr><td>UPN207_r</td><td>3</td><td>178947821</td><td>G</td><td>A</td><td>547</td><td>0</td><td>550</td><td>0.00 %</td><td>530</td><td>48</td><td>581</td><td>8.26 %</td><td>PIK3CA</td><td>ENST00000263967</td><td>missense_variant</td><td>cGt/cAt|p.Arg899His/c.2696G>A</td></tr>
<tr><td>UPN207_r</td><td>19</td><td>17942592</td><td>C</td><td>T</td><td>560</td><td>0</td><td>567</td><td>0.00 %</td><td>535</td><td>44</td><td>581</td><td>7.57 %</td><td>JAK3</td><td>ENST00000458235,ENST00000527670,ENST00000534444</td><td>missense_variant</td><td>cGg/cAg|p.Arg899Gln/c.2696G>A,cGg/cAg|p.Arg899Gln/c.2696G>A,cGg/cAg|p.Arg899Gln/c.2696G>A</td></tr>
</table>

UPN207_r	19	4054491	C	T	84	0	84	0.00 %	292	22	314	7.01 %	ZBTB7A	ENST00000322357;ENST00000601588	missense_variant	p.Arg247Gln/c.740G>A
UPN207_r	16	9017112	C	T	488	1	492	0.20 %	266	18	287	6.27 %	USP7	ENST00000344836,ENST00000381886,ENST00000535863,ENST00000563085,ENST00000542333,ENST00000566004,ENST00000569230,E	missense_variant	Gta/Ata|p.Val115Ile/c.343G>A,Gta/Ata|p.Val99Ile/c.295G>A,Gta/Ata|p.Val16Ile/c.46G>A,Gta/Ata|p.Val16Ile/c.46G>A,Gta/Ata|p.Val57Ile/c.169G>A,Gta/Ata|p.Val57Ile/c.169G>A,Gta/Ata|p.Val57Ile/c.169G>A,Gta/Ata|p.Val101Ile/c.301G>A,Gta/Ata|p.Val57Ile/c.169G>A
UPN207_r	1	216595321	C	T	638	0	640	0.00 %	297	20	319	6.27 %	USH2A	ENST00000366943,ENST00000307340,ENST00000366942	missense_variant	Gcc/Acc|p.Ala120Thr/c.358G>A,Gcc/Acc|p.Ala120Thr/c.358G>A,Gcc/Acc|p.Ala120Thr/c.358G>A
UPN207_r	19	10943949	C	CA	615	0	619	0.00 %	635	38	638	5.96 %	DNM2	ENST00000591818	frameshift_variant	ccg/Accg|p.Pro111fs/c.330dupA
UPN207_r	7	152007083	C	T	561	0	561	0.00 %	495	29	524	5.53 %	KMT2C	ENST00000355193,ENST00000262189,ENST00000558084	missense_variant	Gtg/Atg|p.Val273Met/c.817G>A,Gtg/Atg|p.Val273Met/c.817G>A,Gtg/Atg|p.Val273Met/c.817G>A
UPN207_r	5	67588988	C	T	698	0	701	0.00 %	264	14	280	5.00 %	PIK3R1	ENST00000396611;ENST00000521381;ENST00000521657;ENST00000274335;ENST00000523807;ENST00000522084;ENST00000320694;E	missense_variant	p.Ala360Val/c.1079C>T;p.Ala90Val/c.269C>T;p.Ala60Val/c.179C>T;p.Ala33Val/c.98C>T
UPN207_r	7	151841899	C	T	592	1	594	0.17 %	539	27	568	4.75 %	KMT2C	ENST00000355193,ENST00000360104,ENST00000262189,ENST00000424877	missense_variant	Gca/Aca|p.Ala4805Thr/c.14413G>A,Gca/Aca|p.Ala2304Thr/c.6910G>A,Gca/Aca|p.Ala4748Thr/c.14242G>A,Gca/Aca|p.Ala1361Thr/c.4081G>A
UPN207_r	19	15281634	G	A	479	1	484	0.21 %	1077	28	1107	2.53 %	NOTCH3	ENST00000263388	missense_variant+splice_region_variant	tCg/tTg|p.Ser1580Leu/c.4739C>T
UPN208_r1	no data															
UPN209_r	no Variants															
UPN210_r1	no Variants															
UPN211_r1	3	178952085	A	G	735	0	736	0.00 %	113	84	197	42.64 %	PIK3CA	ENST00000263967	missense_variant	p.His1047Arg/c.3140A>G
UPN211_r1	10	104853038	C	A	639	5	645	0.78 %	121	57	178	32.02 %	NT5C2	ENST00000343289;ENST00000404739;ENST00000423468;ENST00000421281	missense_variant	p.Leu339Phe/c.1017G>T;p.Leu310Phe/c.930G>T;p.Leu39Phe/c.117G>T
UPN190_r2	1	215931942	G	T	2789	14	2805	0.50 %	1287	311	1598	19.46 %	USH2A	ENST00000366943,ENST00000307340	missense_variant	p.Pro3795Gln/c.11384C>A

UPN190_r2	9	139390863	AC	AGCTTAAGGGGACTT	2080	30	2089	1.44 %	913	266	913	29.13 %	NOTCH1	ENST00000277541	frameshift_variant+stop_gained+missense_variant	p.Val2443fs/c.7327delGinsAAGTCCCCTTAAGC
UPN190_r2	12	122247702	C	A	1387	1	1388	0.07 %	636	171	807	21.19 %	SETD1B	ENST00000604567, ENST00000542440, ENST00000267197	stop_gained	p.Ser284*/c.851C>A
UPN190_r2	12	122248216	G	GC	1077	8	1084	0.74 %	658	113	667	16.94 %	SETD1B	ENST00000604567,ENST00000542440,ENST00000267197	frameshift_variant	p.Asp458fs/c.1371dupC
UPN201_r2	1	9787030	G	A	673	0	675	0.00 %	303	318	625	50.88 %	PIK3CD	ENST00000361110;ENST00000536656;ENST00000377346	missense_variant	p.Glu1045Lys/c.3133G>A;p.Glu1021Lys/c.3061G>A
UPN201_r2	5	67591139	GACCAAT	G	610	0	614	0.00 %	227	237	466	50.86 %	PIK3R1	ENST00000396611;ENST00000521381;ENST00000521657;ENST00000274335;ENST00000320694;ENST00000336483;E	inframe_deletion	p.Gln579_Tyr580del/c.1735_1740delCAATAC;p.Gln279_Tyr280del/c.835_840delCAATAC;p.Gln309_Tyr310del/c.925_930delCAATAC;p.Gln216_Tyr217del/c.646_651delCAATAC
UPN202_r2	no dat															
UPN205_r2	9	139417356	C	T	223	0	225	0.00 %	261	15	278	5.40 %	NOTCH1	ENST00000277541	missense_variant	p.Gly230Arg/c.688G>A
UPN205_r2	16	3790511	C	G	713	86	805	######	405	330	738	44.72 %	CREBBP	ENST00000262367,ENST00000382070,ENST00000573517,ENST00000570939	missense_variant	p.Arg1341Pro/c.4022G>C;p.Arg1303Pro/c.3908G>C;p.Arg109Pro/c.326G>C;p.Arg214Pro/c.641G>C
UPN205_r2	3	178936094	C	A	651	17	668	2.54 %	278	217	495	43.84 %	PIK3CA	ENST00000263967	missense_variant	p.Gln546Lys/c.1636C>A
UPN208_r2	6	41903683	G	GGT	574	0	575	0.00 %	592	46	592	7.77 %	CCND3	ENST00000372991;ENST00000511642;ENST00000372987;ENST00000415497;ENST00000372988;ENST00000414200;E	frameshift_variant	p.Leu292fs/c.872_873dupAC;p.Leu211fs/c.629_630dupAC;p.Leu242fs/c.722_723dupAC;p.Leu96fs/c.284_285dupAC;p.Leu220fs/c.656_657dupAC;p.Pro165fs/c.492_493dupAC
UPN208_r2	6	41903745	C	CG	519	0	520	0.00 %	596	34	600	5.67 %	CCND3	ENST00000372991;ENST00000511642;ENST00000372987;ENST00000415497;ENST00000372988;ENST00000414200;E	frameshift_variant	p.Arg271fs/c.811dupC;p.Arg190fs/c.568dupC;p.Arg221fs/c.661dupC;p.Arg75fs/c.223dupC;p.Arg199fs/c.595dupC;p.Leu146fs/c.431dupC
UPN208_r2	10	89717675	CG	CAGCATTA	429	1	430	0.23 %	390	84	481	17.46 %	PTEN	ENST00000371953	stop_gained+missense_variant+inframe_insertion	p.Arg234delinsGlnHisTer
UPN210_r2	no Variants															

UPN211_r2	3	178952085	A	G	735	0	736	0.00 %	221	157	379	41.42 %	PIK3CA	ENST00000263967	missense_variant	p.His1047Arg/c.3140A>G
UPN211_r2	10	104853038	C	A	639	5	645	0.78 %	273	98	374	26.20 %	NT5C2	ENST00000343289;ENST00000404739;ENST00000423468;ENST00000421281	missense_variant	p.Leu339Phe/c.1017G>T;p.Leu310Phe/c.930G>T;p.Leu39Phe/c.117G>T
UPN211_r3	4	153249384	C	T	695	0	699	0.00 %	704	35	741	4.72 %	FBXW7	ENST00000281708;ENST00000296555;ENST00000263981;ENST00000603548;ENST00000393956;E	missense_variant	p.Arg465His/c.1394G>A;p.Arg347His/c.1040G>A;p.Arg385His/c.1154G>A;p.Arg289His/c.866G>A
UPN211_r3	5	67589571	TAGGGAAAAAATTACATGAATATAACAC	T	452	0	452	0.00 %	341	123	465	26.45 %	PIK3R1	ENST00000396611;ENST00000521381;ENST00000521657;ENST00000274335;ENST00000320694;ENST00000521409;ENST00000336483;E	disruptive_inframe_deletion	p.Gly446_Thr454del/c.1335_1361delAGGGAAAAAATTACATGAATATAACAC;p.Gly146_Thr154del/c.435_461delAGGGAAAAAATTACATGAATATAACAC;p.Gly83_Thr91del/c.246_272delAGGGAAAAAATTACATGAATATAACAC;p.Gly176_Thr184del/c.525_551delAGGGAAAAAATTACATGAATATAACAC;p.Gly
UPN211_r3	10	89717670	CAC	TACTCCGGAGGATCAT	643	7	650	1.08 %	414	58	467	12.42 %	PTEN	ENST00000371953	frameshift_variant+missense_variant	p.Thr232fs/c.695_697delCACinsTACTCCGGAGGATCAT
UPN211_r3	10	89717672	C	CGAGGGTCAGCA	653	0	655	0.00 %	414	46	472	9.75 %	PTEN	ENST00000371953	frameshift_variant	p.Arg234fs/c.699_700insGGGTCAGCAGA
UPN211_r3	17	7577538	C	T	701	0	701	0.00 %	592	269	867	31.03 %	TP53	ENST00000269305;ENST00000413465;ENST00000359597;ENST00000420246;ENST00000455263;ENST00000445888;ENST00000509690;E	missense_variant	p.Arg248Gln/c.743G>A;p.Arg116Gln/c.347G>A;p.Arg155Gln/c.464G>A
UPN211_r3	17	7577548	C	G	664	0	666	0.00 %	782	55	864	6.37 %	TP53	ENST00000269305;ENST00000413465;ENST00000359597;ENST00000420246;ENST00000455263;ENST00000445888;ENST00000509690;E	missense_variant	p.Gly245Arg/c.733G>C;p.Gly113Arg/c.337G>C;p.Gly152Arg/c.454G>C

UPN211_r3	17	7577548	C	T	664	1	666	0.15 %	782	23	864	2.66 %	TP53	ENST00000269305;ENST00000413465;ENST00000359597;ENST00000420246;ENST00000455263;ENST00000445888;ENST00000509690;E	missense_variant	p.Gly245Ser/c.733G>A;p.Gly113Ser/c.337G>A;p.Gly152Ser/c.454G>A
UPN211_r3	17	7579536	C	A	668	3	676	0.44 %	639	51	691	7.38 %	TP53	ENST00000269305;ENST00000413465;ENST00000359597;ENST00000420246;ENST00000455263;ENST00000445888;ENST00000508793;E	stop_gained	p.Glu51*/c.151G>T
