# Supplementary material for: Molecular variants, clonal evolution and clinical relevance in pediatric and adult T-cell lymphoblastic neoplasia
Source: Blood Cancer J. 2026 Apr 2;16(1):57. doi: 10.1038/s41408-026-01488-w (PMC13066408; doi:10.1038/s41408-026-01488-w)
Supplement: Supplementary file 3 — Supplemental Data 2 [file 41408_2026_1488_MOESM3_ESM.pdf]

Supplemental Data 2

					Germline				Tumor				
**ID**	**Chr**	**Start**	**End**	**Type**	**CCF**	**lower**	**upper**	**#SNPs**	**CCF**	**lower**	**upper**	**#SNPs**	**Variant**
UPN001	9	0	141213430	3	not detected				48.32 %	47.08 %	49.55 %	3782	dup9
UPN001	9	16054088	38782387	2	not detected				68.28 %	67.61 %	68.94 %	978	LOH in 9p
UPN001	12	10296130	26728960	1	not detected				50.21 %	49.18 %	51.25 %	802	del in 12p
UPN001	17	29007975	30415430	1	not detected				59.02 %	51.24 %	66.80 %	25	del in 17q
UPN001	19	13108717	14828116	3	not detected				47.47 %	42.67 %	52.27 %	75	dup in 19p
UPN001	19	37685602	59097933	2	not detected				66.97 %	66.09 %	67.85 %	1146	dup in 19q
UPN001	X	0	155270560	3	not detected				47.65 %	46.92 %	48.38 %	3229	dupX
UPN001	X	70445165	155270560	2	not detected				50.10 %	49.75 %	50.45 %	3643	LOH in Xq
UPN002	11	31647635	32493062	1	not detected				73.89 %	68.35 %	79.43 %	31	del in 11p
UPN002	12	0	31069369	1	not detected				69.24 %	68.54 %	69.94 %	1745	del in 12p
UPN002	17	28912879	30377486	1	not detected				69.27 %	64.31 %	74.24 %	46	del in 17q
UPN002	18	0	15386489	1	not detected				68.85 %	67.98 %	69.72 %	968	del in 18p
UPN003	7	3532126	4631793	3	not detected				86.01 %	79.26 %	92.76 %	65	dup in 7p
UPN004	9	70988363	106352698	1	not detected				22.99 %	22.06 %	23.93 %	1152	del in 9q
UPN005	1	23390522	24741042	1	not detected				95.14 %	94.10 %	96.19 %	48	del in 1p
UPN005	1	82726325	83604320	1	not detected				93.74 %	92.68 %	94.79 %	33	del in 1p
UPN005	8	130249199	130560035	1	not detected				88.70 %	80.06 %	97.35 %	25	del in 8p
UPN005	9	0	36997324	2	not detected				98.02 %	97.83 %	98.22 %	1698	LOH9p
UPN005	9	131462039	134032811	1	not detected				94.41 %	93.58 %	95.25 %	149	del in 9q
UPN005	12	12866352	12990148	1	not detected				89.22 %	82.97 %	95.47 %	12	del in 12p
UPN005	12	111107864	112037090	1	not detected				90.31 %	82.29 %	98.33 %	17	del in 12q
UPN005	17	20827600	22242355	1	not detected				58.48 %	35.60 %	81.36 %	15	del in 17p
UPN006	1	181718961	249110238	3	not detected				76.15 %	75.25 %	77.05 %	2700	dup in 1q
UPN006	7	14173817	54671049	1	not detected				55.40 %	54.86 %	55.93 %	1871	del in 7p
UPN006	11	0	39815247	1	not detected				87.94 %	87.65 %	88.23 %	1920	del in 11p
UPN006	13	27258429	30036364	1	not detected				87.63 %	86.85 %	88.41 %	144	del in 13q
UPN006	17	28380739	30415430	1	not detected				85.87 %	83.68 %	88.05 %	50	del in 17q
UPN007	6	0	171115067	3	not detected				59.43%	57.94%	60.92%	5210	dup6

T-ALL adult_Primary

UPN007	7	0	159138663	3	not detected	58.22%	56.32%	60.12%	4100	dup7
UPN007	8	0	146364022	3	not detected	59.99%	58.22%	61.76%	2295	dup8
UPN007	11	0	135006516	3	not detected	58.37%	57.48%	59.26%	4792	dup11
UPN007	13	0	115169878	3	not detected	59.24%	58.55%	59.92%	3995	dup13
UPN007	17	0	81195210	3	not detected	59.44%	57.51%	61.37%	1082	dup17
UPN007	18	0	78077248	3	not detected	59.25%	58.08%	60.43%	3270	dup18
UPN007	19	0	59128983	3	not detected	61.43 %	57.62 %	65.25 %	128	dup19
UPN007	20	0	63025520	3	not detected	58.49 %	56.88 %	60.11 %	668	dup20
UPN007	21	0	48129895	3	not detected	60.12 %	58.94 %	61.31 %	1309	dup21
UPN008	11	0	36366828	2	not detected	84.26 %	83.95 %	84.56 %	1868	LOH in 11p
UPN008	12	10370284	21390973	1	not detected	66.97 %	65.37 %	68.57 %	518	del in 12p
UPN009	no variants									
UPN010	1	5964799	7857492	1	not detected	91.90 %	91.27 %	92.53 %	76	del in 1p
UPN010	1	23005597	25045470	1	not detected	91.66 %	91.02 %	92.31 %	73	del in 1p
UPN010	2	60169426	60484834	1	not detected	92.25 %	77.13 %	107.37 %	2	del in 2p
UPN010	8	119883000	120425595	1	not detected	92.20 %	91.23 %	93.17 %	34	del in 8q
UPN010	11	0	44613265	2	not detected	95.36 %	95.24 %	95.48 %	2013	LOH in 11p
UPN010	12	7142875	18267486	1	not detected	83.21 %	82.72 %	83.70 %	457	del in 12p
UPN010	14	88502480	90131248	1	not detected	91.99 %	91.38 %	92.61 %	66	del in 14q
UPN010	14	98880365	99266175	1	not detected	91.38 %	89.78 %	92.98 %	23	del in 14q
UPN010	16	3531204	6196443	1	not detected	91.68 %	91.12 %	92.24 %	142	del in 16p
UPN010	18	2452614	2660866	1	not detected	90.63 %	87.86 %	93.40 %	9	del in 18p
UPN010	18	11708430	12995742	1	not detected	89.23 %	85.38 %	93.08 %	47	del in 18p
UPN011	9	19758771	22751855	1	not detected	35.60 %	31.96 %	39.24 %	156	del in 9p
UPN011	9	136037259	141068637	2	not detected	63.64 %	62.62 %	64.67 %	341	LOH in 9q
UPN011	15	41806373	43235557	1	not detected	44.62 %	41.45 %	47.78 %	38	del in 15q
UPN012	no data									
UPN013	9	0	32673400	2	not detected	92.38 %	92.06 %	92.71 %	1619	LOH in 9p
UPN013	10	88684336	94821204	3	not detected	67.56 %	64.38 %	70.74 %	273	dup in 10q
UPN014	1	4247126	7314630	1	not detected	30.66 %	28.57 %	32.76 %	170	del in 1p

UPN014	2	39513075	41219864	1	not detected	89.43 %	88.44 %	90.41 %	57	del in 2p
UPN014	6	99869216	100501128	1	not detected	89.94 %	88.61 %	91.28 %	25	del in 6q
UPN014	9	20617810	39162801	1	not detected	82.19 %	80.37 %	84.02 %	708	del in 9p
UPN014	9	108198444	110126658	1	not detected	89.32 %	87.97 %	90.68 %	75	del in 9q
UPN015	1	3249242	7314630	1	not detected	85.82 %	85.35 %	86.28 %	247	del in 1p
UPN015	3	71196518	71526270	1	not detected	94.76 %	93.94 %	95.59 %	28	del in 3p
UPN015	4	148092130	171776427	2	100.00 %	100.00 %				LOH in 4q
UPN015	5	133604789	133958309	1	not detected	94.42 %	92.88 %	95.95 %	13	del in 5q
UPN015	6	73751334	86282090	1	not detected	93.68 %	93.03 %	94.32 %	314	del in 6q
UPN015	8	18277912	21662646	1	not detected	93.48 %	92.37 %	94.59 %	231	del in 8p
UPN015	11	67101158	73686592	1	not detected	93.36 %	92.70 %	94.01 %	271	del in 11q
UPN015	11	83992418	116101801	1	not detected	92.16 %	91.45 %	92.86 %	1203	del in 11q
UPN015	12	7652308	29663330	1	not detected	93.88 %	93.60 %	94.16 %	1033	del in 12p
UPN015	13	33003696	63644720	1	not detected	93.81 %	93.51 %	94.11 %	1078	del in 13q
UPN015	14	34314291	50442382	2	100.00 %	100.00 %				LOH in 14q
UPN015	17	26145406	26938175	1	not detected	94.05 %	92.87 %	95.24 %	21	del in 17q
UPN016	9	133828	21780037	3	not detected	63.88 %	62.27 %	65.49 %	1194	dup in 9p
UPN016	9	28382826	32252919	3	not detected	65.91 %	61.46 %	70.35 %	147	dup in 9p
UPN017	8	0	146364022	3	not detected	51.14 %	48.43 %	53.85 %	4468	dup8
UPN017	9	20350021	22713304	1	not detected	72.91 %	67.94 %	77.88 %	133	del in 9p
UPN017	9	133370377	135424112	1	not detected	58.23 %	52.87 %	63.59 %	76	del in 9q
UPN017	11	117417973	118401125	1	not detected	87.66 %	86.26 %	89.07 %	55	del in 11p
UPN017	15	98982627	101551136	3	not detected	72.37 %	67.02 %	77.72 %	189	dup in 15q
UPN018	2	144191260	146494132	1	not detected	77.86 %	76.24 %	79.48 %	59	del in 2q
UPN018	2	223064508	224726901	1	not detected	76.96 %	75.85 %	78.08 %	75	del in 2q
UPN018	12	0	35800000	1	not detected	75.98 %	75.69 %	76.27 %	1807	del 12p
UPN019	9	20356117	22863791	1	not detected	52.40 %	45.96 %	58.84 %	123	del in 9p
UPN019	11	0	49700687	2	not detected	79.05 %	78.77 %	79.32 %	1302	LOH in 11p
UPN019	12	12703002	16900433	1	not detected	64.25 %	63.43 %	65.07 %	205	del in 12p
UPN019	19	903017	2365113	1	not detected	91.86 %	90.15 %	93.58 %	77	del in 19p

UPN020	4	147200455	191154276	2	not detected	96.44 %	96.33 %	96.56 %	1831	LOH in 4q
UPN020	9	0	38039604	2	not detected	96.75 %	96.55 %	96.96 %	1677	LOH9p
UPN021	12	94233225	133883364	1	not detected	28.13 %	27.59 %	28.07 %	1947	del in 12q
UPN022	1	33839493	41197538	3	not detected	68.71 %	64.52 %	72.90 %	282	dup in 1p
UPN022	2	113275957	114330596	3	not detected	67.62 %	56.53 %	78.71 %	54	dup in 2q
UPN022	2	119507392	191335186	3	not detected	63.29 %	61.97 %	64.62 %	2404	dup in 2q
UPN022	9	0	37574451	2	not detected	84.62 %	84.22 %	85.01 %	1927	LOH9p
UPN022	11	0	135006516	2	not detected	85.46 %	85.19 %	85.72 %	5637	LOH11
UPN022	12	45654233	94721983	3	not detected	70.52 %	68.73 %	72.31 %	1467	dup in 12p
UPN023	9	0	37180697	1	not detected	90.09 %	89.80 %	90.37 %	2066	del in 9p
UPN023	12	10841091	13547719	1	not detected	89.96 %	88.52 %	91.41 %	129	del in 12p
UPN024	7	0	159124173	3	not detected	81.99 %	80.77 %	83.22 %	5916	dup7
UPN024	17	28912879	30415430	1	not detected	88.19 %	83.71 %	92.66 %	29	del in 17q
UPN025	5	103919508	104286271	1	not detected	96.79 %	93.95 %	99.64 %	7	del in 5q
UPN025	9	131462039	134027165	1	not detected	91.95 %	91.13 %	92.76 %	126	del in 9q
UPN025	16	0	2249595	1	not detected	91.70 %	90.69 %	92.71 %	137	del in 16p
UPN025	19	0	14890476	3	not detected	72.57 %	70.20 %	74.94 %	804	dup in 19p
UPN026	1	199129328	199996598	1	not detected	76.36 %	67.80 %	84.92 %	28	del in 1q
UPN026	6	72835062	93551709	1	not detected	84.45 %	84.09 %	84.80 %	683	del in 6q
UPN026	9	21184923	22648917	1	not detected	74.79 %	69.13 %	80.45 %	69	del in 9p
UPN026	13	69164068	69460238	1	not detected	82.20 %	79.68 %	84.72 %	13	del in 13q
UPN027	6	75519783	109056448	1	not detected	84.81 %	84.06 %	85.56 %	1008	del in 6q
UPN027	8	108814632	146364022	2	not detected	93.05 %	92.55 %	93.55 %	1491	LOH in 8q
UPN027	19	8616347	18296566	3	not detected	79.88 %	76.95 %	82.81 %	491	dup in 19p
UPN027	21	0	48129895	3	not detected	83.03 %	81.48 %	84.57 %	1651	dup21
UPN028	1	0	109266813	2	not detected	95.54 %	95.44 %	95.64 %	3953	LOH in 1p
UPN028	6	73980933	171115067	2	not detected	95.66 %	95.51 %	95.82 %	3481	LOH in 6q
UPN028	15	40751801	102531392	2	not detected	95.31 %	95.10 %	95.51 %	2729	LOH in 15q
UPN028	17	28912879	30415430	1	not detected	90.76 %	87.93 %	93.58 %	27	del in 17q
UPN029	9	19499535	38825011	1	not detected	27.60 %	26.55 %	28.64 %	687	del in 9p

UPN030	1	125000000	249250621	3	not detected	65.18 %	63.66 %	66.70 %	4635	dup1q
UPN030	2	0	243199373	3	not detected	65.99 %	65.02 %	66.96 %	10330	dup2
UPN030	3	0	198022430	3	not detected	56.75 %	55.55 %	57.95 %	8221	dup3
UPN030	4	0	191154276	3	not detected	56.25 %	55.06 %	57.44 %	8066	dup4
UPN030	5	0	180915260	3	not detected	59.30 %	58.08 %	60.53 %	7386	dup5
UPN030	6	0	171115067	1	not detected	88.41 %	88.13 %	88.68 %	9315	del6
UPN030	7	0	159138663	3	not detected	65.44 %	64.21 %	66.66 %	7057	dup7
UPN030	8	0	146364022	3	not detected	52.90 %	51.83 %	53.98 %	6527	dup8
UPN030	9	0	141213431	3	not detected	51.32 %	50.17 %	52.48 %	5399	dup9
UPN030	11	0	135006516	3	not detected	64.84 %	63.55 %	66.13 %	6306	dup11
UPN030	13	19403621	88255179	1	not detected	79.51 %	78.73 %	80.29 %	3047	del in 13q
UPN030	13	104111901	112023123	1	not detected	86.55 %	85.40 %	87.69 %	566	del in 13q
UPN030	13	104428282	107032732	3	not detected	86.00 %				dup in 13q
UPN030	13	109130370	112023123	3	not detected	86.00 %				dup in 13q
UPN030	13	112023124	115169878	3	not detected	59.26 %	52.98 %	65.54 %	201	dup in 13q
UPN030	14	0	107349540	3	not detected	61.76 %	60.07 %	63.44 %	3975	dup14
UPN030	15	0	102531392	1	not detected	60.12 %	59.48 %	60.77 %	4039	del15
UPN030	16	0	90354753	3	not detected	64.33 %	62.73 %	65.93 %	4112	dup16
UPN030	17	0	16210580	1	not detected	91.73 %	91.07 %	92.39 %	1016	del in 17p
UPN030	18	0	78077248	3	not detected	67.46 %	65.86 %	69.06 %	3694	dup18
UPN030	19	0	59128983	3	not detected	55.41 %	53.54 %	57.29 %	2972	dup19
UPN030	X	0	155270560	3	not detected	48.98 %	47.99 %	49.96 %	6802	dupX
UPN031	1	104430395	119041203	1	not detected	62.67 %	61.74 %	63.61 %	604	del in 1p
UPN031	5	88450292	121857340	1	not detected	50.38 %	49.78 %	50.97 %	1045	del in 5q
UPN031	9	21661861	25308645	1	not detected	55.68 %	52.52 %	58.84 %	179	del in 9p
UPN031	12	0	16633880	1	not detected	50.67 %	50.00 %	51.34 %	822	del in 12p
UPN032	no data									
UPN033	4	109038217	109277343	1	NA	97.00 %				del in 4q
UPN033	6	77716141	121560345	1	NA	97.00 %				del in 6q
UPN033	9	0	34093703	2	NA	97.00 %				del in 9p

UPN034	9	5387145	22307655	1	not detected	89.38 %	89.00 %	89.76 %	949	del in 9p
UPN034	11	33976330	36616843	1	not detected	88.66 %	88.06 %	89.26 %	149	del in 11p
UPN034	17	37838775	81195210	3	not detected	40.66 %	39.44 %	41.89 %	1789	dup in 17q
UPN035	10	89690798	90153268	1	not detected	75.79 %	73.02 %	78.57 %	76.01 %	del in 10q
UPN036	6	0	32139689	2	not detected	89.78 %	89.62 %	89.95 %	2831	LOH in 6p
UPN036	9	19406107	39162801	1	not detected	82.05 %	81.52 %	82.59 %	752	del in 9p
UPN036	10	89510393	92967000	1	not detected	76.06 %	72.57 %	79.56 %	133	del in 10q
UPN037	9	0	35642313	2	not detected	88.23 %	87.63 %	88.82 %	1744	LOH in 9p
UPN037	10	0	36655857	3	not detected	74.16 %	72.69 %	75.64 %	1806	dup in 10p
UPN038_p	no data									
UPN039_p	7	43748	38291362	3	not detected	72.78 %	71.64 %	73.91 %	2118	dup in 7q
UPN039_p	7	156880398	159074587	1	not detected	78.69 %	77.30 %	80.07 %	115	del in 7p
UPN039_p	9	0	49000000	1	not detected	80.00 %	79.00 %	80.00 %	1730	del9p
UPN039_p	9	49000000	141213430	3	not detected	69.12 %	68.16 %	70.07 %	2917	dup9q
UPN040_p	6	0	32798629	2	not detected	83.16 %	82.95 %	83.37 %	2911	LOH6
UPN040_p	9	0	39162801	1	not detected	71.72 %	71.14 %	72.30 %	2028	del9p
UPN040_p	17	32662435	81195210	2	not detected	83.09 %	82.84 %	83.34 %	1982	LOH17
UPN041_p	2	0	81245586	2	not detected	95.24 %	95.13 %	95.35 %	3552	LOH2
UPN041_p	9	0	45755225	2	not detected	95.27 %	95.07 %	95.47 %	1927	LOH9
UPN041_p	13	19020095	115169878	3	not detected	81.16 %	80.16 %	82.15 %	4011	dup13
UPN041_p	20	0	63025520	3	not detected	79.13 %	77.40 %	80.87 %	2790	dup20

Supplemental Data 2

					Germline				Tumor				
**ID**	**Chr**	**Start**	**End**	**Type**	**CCF**	**lower**	**upper**	**#SNPs**	**CCF**	**lower**	**upper**	**#SNPs**	**Variant**
UPN038_r	no data												
UPN039_r	7	156880398	159074587	1	not detected				19.31 %	17.02 %	21.60 %	115	del in 7p
UPN039_r	7	43748	38291362	3	not detected				22.71 %	21.96 %	23.47 %	2118	dup in 7q
UPN040_r	6	0	32798629	2	not detected				44.35 %	43.91 %	44.78 %	2911	LOH6
UPN040_r	9	0	39162801	1	not detected				28.95 %	28.38 %	29.52 %	2028	del9p
UPN040_r	17	32662435	81195210	2	not detected				44.57 %	44.05 %	45.09 %	1982	LOH17
UPN041_r	2	0	81245586	2	not detected				92.49 %	92.37 %	92.60 %	3552	LOH2
UPN041_r	9	0	45755225	2	not detected				92.35 %	92.14 %	92.56 %	1927	LOH9
UPN041_r	13	19020095	115169878	3	not detected				16.69 %	16.25 %	17.14 %	4011	dup13
UPN041_r	20	0	63025520	3	not detected				16.41 %	15.69 %	17.14 %	2790	dup20

Supplemental Data 2

					Germline				Tumor				
ID	Chr	Start	End	Type	CCF	lower	upper	#SNPs	CCF	lower	upper	#SNPs	Variant
UPN042	1	78175328	80996752	1	not detected				66.67 %	64.03 %	69.31 %	65	del in 1p
UPN042	1	187070611	193289136	1	not detected				66.72 %	64.28 %	69.15 %	189	del in 1q
UPN042	1	193507297	228088833	3	not detected				66.38 %	64.94 %	67.82 %	1352	dup in 1q
UPN042	1	226870403	240942448	1	not detected				67.00 %	66.33 %	67.67 %	735	del in 1q
UPN042	9	0	40727909	1	not detected				73.41 %	72.93 %	73.89 %	1876	del9p
UPN043	9	19702541	24070963	1	not detected				46.76 %	43.45 %	50.06 %	217	del in 9p
UPN044	9	0	38899071	3	not detected				17.40 %	16.62 %	18.17 %	1838	dup in 9p
UPN045	9	180133	34298991	2	not detected				97.56 %	97.27 %	97.86 %	1647	LOH in 9p
UPN046	9	0	33577384	2	not detected				98.00 %	98.00 %	98.00 %	1580	LOH in 9p
UPN046	10	89624787	89705464	1	not detected				NA	NA	NA	2	del in 10q
UPN047	2	144620189	243199373	3	not detected				81.41 %	80.65 %	82.16 %	3620	dup in 2q
UPN047	6	64406323	171115067	1	not detected				91.90 %	91.75 %	92.05 %	3982	del6q
UPN047	9	0	39154913	1	not detected				83.57 %	82.46 %	84.68 %	1942	del in 9p
UPN047	14	22313479	22990890	1	not detected				88.84 %	83.20 %	94.47 %	56	del in 14q
UPN048	9	206143	44748466	2	not detected				95.06 %	94.62 %	95.49 %	1911	del9p
UPN049	9	0	13776568	1	not detected				95.87 %	95.55 %	96.19 %	848	del in 9p
UPN049	9	20818510	44734557	1	not detected				95.37 %	94.83 %	95.92 %	662	del in 9p
UPN049	12	0	20458666	1	not detected				94.60 %	94.30 %	94.89 %	1027	del in 12p
UPN050	4	161310770	162434592	3	not detected				90.79 %	82.31 %	99.27 %	62	deup in 4q
UPN050	5	35223427	35860332	1	not detected				84.28 %	67.87 %	100.69 %	15	del in 5p
UPN050	9	0	45755225	2	not detected				97.60 %	97.26 %	97.94 %	1974	LOH9p
UPN050	14	22052636	23006341	1	not detected				84.16 %	76.92 %	91.39 %	47	del in 14q
UPN051	9	0	36822378	2	not detected				97.49 %	97.12 %	97.87 %	1644	LOH in 9p
UPN052	9	0	44769133	2	not detected				95.24 %	94.89 %	95.60 %	1683	LOH9p
UPN053	9	0	44769133	2	not detected				97.00 %	96.00 %	97.00 %	1538	LOH9p
UPN054	9	0	19795098	1	not detected				61.70 %	60.99 %	62.40 %	1088	del in 9p
UPN054	9	27905132	40185526	1	not detected				61.00 %	59.89 %	62.11 %	382	del in 9p
UPN054	16	0	8994325	2	not detected				42.15 %	41.01 %	43.29 %	522	LOH in 16p

T-ALL pediatric_not relapsed

UPN055	9	0	35721467	2	not detected		73.13 %	71.69 %	74.57 %	1762	LOH in 9p
UPN056	4	108948898	109370588	1	not detected		96.44 %	95.12 %	97.76 %	8	del in 4q
UPN056	9	0	39147759	1	not detected		96.14 %	95.82 %	96.46 %	1784	del in 9p
UPN057	9	0	33294320	2	not detected		93.61 %	93.31 %	93.91 %	1709	LOH in 9p
UPN058	9	0	31550952	3	not detected		77.83 %	77.45 %	78.21 %	1775	dup in 9p
UPN058	10	89627829	89926115	1	not detected		60.62 %	52.70 %	68.55 %	58	del in 10q
UPN058	16	77759157	79010968	3	not detected		80.24 %	75.86 %	84.63 %	80	dup in 16q
UPN059	9	21475634	21910122	1	not detected		93.34 %	92.62 %	94.06 %	17	del in 9p
UPN059	9	22029144	23209563	3	not detected		91.02 %	84.12 %	97.92 %	81	dup in 9p
UPN059	11	81890697	126188806	1	not detected		48.44 %	48.00 %	48.89 %	1875	del in 11q
UPN059	17	2522660	5040264	1	not detected		45.09 %	42.99 %	47.20 %	134	del in 17p
UPN059	17	33318755	34441525	1	not detected		48.08 %	45.49 %	50.67 %	50	del in 17p
UPN059	17	41753427	42877663	1	not detected		43.95 %	40.04 %	47.86 %	38	del in 17p
UPN060	3	43661196	85064279	1	not detected		29.62 %	29.09 %	30.15 %	1455	del in 3p
UPN060	6	62442530	103219959	1	not detected		91.03 %	90.70 %	91.37 %	1167	del in 5q
UPN060	14	22392802	22962374	1	not detected		88.96 %	83.07 %	94.85 %	44	del in 14q
UPN061	7	91922155	159138663	3	not detected		79.78 %	78.81 %	80.75 %	2502	dup in 7q
UPN061	9	0	37748004	2	not detected		80.80 %	80.38 %	81.21 %	1831	LOH in 9p
UPN061	9	0	39189362	3	not detected		81.65 %	73.77 %	89.52 %	57	dup9p
UPN061	10	93083	36444351	3	not detected		92.16 %	90.88 %	93.44 %	1687	dup in 10p
UPN062	9	364604	36469174	2	not detected		81.92 %	81.47 %	82.37 %	1777	LOH in 9p
UPN063	6	65882750	170919470	2	not detected		52.35 %	52.02 %	52.68 %	3734	LOH in 6q
UPN063	9	0	39239013	1	not detected		94.20 %	93.54 %	94.86 %	1904	del9p
UPN063	9	70731742	141213431	3	not detected		76.25 %	75.35 %	77.14 %	3045	dup9q
UPN063	18	0	4916906	3	not detected		42.23 %	40.19 %	44.26 %	326	dup in 18p
UPN063	18	7170124	15047653	3	not detected		37.69 %	35.68 %	39.70 %	382	dup in 18p
UPN063	18	18846683	25347936	3	not detected		73.19 %	69.71 %	76.67 %	209	dup in 18q
UPN063	18	25347937	31402597	3	not detected		43.18 %	40.95 %	45.41 %	220	dup in 18q
UPN063	18	31402598	34203980	3	not detected		80.31 %	74.77 %	85.85 %	86	dup in 18q
UPN063	18	34203981	41388171	3	not detected		44.03 %	41.43 %	46.62 %	179	dup in 18q

UPN063	18	43692535	50244155	3	not detected		41.05 %	39.02 %	43.09 %	284	dup in 18q
UPN063	18	50244156	54084761	3	not detected		80.80 %	76.08 %	85.51 %	107	dup in 18q
UPN063	18	54084762	61314136	3	not detected		42.04 %	39.92 %	44.17 %	334	dup in 18q
UPN063	18	72971503	78077248	3	not detected		36.70 %	34.36 %	39.05 %	246	dup in 18q
UPN064	9	0	37230469	1	not detected		97.67 %	97.37 %	97.97 %	1752	del in 9p
UPN064	14	22527266	22921492	1	not detected		99.60 %	99.32 %	99.88 %	36	del in 14q
UPN065	9	19671057	27875005	1	not detected		82.93 %	80.02 %	85.85 %	367	del in 9p

Supplemental Data 2

ID	Chr	Start	End	Type	Germline CCF	Germline lower	Germline upper	Germline #SNPs	Tumor CCF	Tumor lower	Tumor upper	Tumor #SNPs	Variant
UPN066_p	8	0	146364022	3	not detected				62.96 %	62.35 %	63.57 %	5683	dup8
UPN066_p	9	0	36639060	2	not detected				78.94 %	78.54 %	79.34 %	1702	LOH in 9p
UPN067_p	6	61000000	171115067	1	not detected				21.83 %	21.46 %	22.20 %	3972	del6q
UPN067_p	9	0	32542278	3	not detected				44.43 %	43.67 %	45.19 %	1688	LOH in 9p
UPN067_p	14	48000000	107349540	1	not detected				20.76 %	20.30 %	21.22 %	2352	del in 14q
UPN068_p	8	0	146364022	3	not detected				81.00 %	79.00 %	82.00 %	5506	dup8
UPN069_p	9	20039346	35644453	1	not detected				57.97 %	56.29 %	59.66 %	610	del in 9p
UPN070_p	9	0	31181790	3	not detected				44.08 %	42.73 %	45.44 %	1540	dup in 9p
UPN070_p	16	14975292	16675394	3	not detected				84.88 %	78.66 %	91.10 %	58	dup in 16p
UPN071_p	no variants												
UPN072_p	9	11857975	40813425	1	not detected				92.59 %	91.51 %	93.66 %	1065	del in 9p
UPN073_p	9	0	36111474	2	not detected				98.44 %	98.24 %	98.65 %	1612	LOH in 9p
UPN073_p	14	23070121	107349540	2	not detected				97.37 %	97.18 %	97.57 %	3370	LOH in 14q
UPN074_p	2	1.85E+08	186629505	1	not detected				93.02 %	85.87 %	100.18 %	4	del in 2q
UPN074_p	7	1.48E+08	152259972	1	not detected				93.03 %	91.69 %	94.38 %	266	del in 7q
UPN075_p	9	0	44846312	2	not detected				94.09 %	93.74 %	94.44 %	1891	LOH9p
UPN076_p	9	180133	33953464	2	not detected				94.04 %	93.50 %	94.59 %	1639	LOH in 9p
UPN077_p	no variants												
UPN066_r	8	0	146364022	3	not detected				88.05 %	87.26 %	88.85 %	5683	dup8
UPN066_r	9	0	36639060	2	not detected				98.00 %	98.00 %	99.00 %	837	LOH in 9p
UPN067_r	2	43000000	48000000	1	not detected				31.29 %	28.84 %	33.73 %	269	del in 2p
UPN067_r	9	0	32542278	3	not detected				57.68 %	56.93 %	58.43 %	1688	LOH in 9p
UPN068_r	8	0	146364022	3	not detected				90.00 %				dup8
UPN069_r	1	#########	249250620	3	not detected				52.29 %	43.40 %	61.19 %	4151	dup in 1q
UPN069_r	2	#########	243199373	1	not detected				36.36 %	35.78 %	36.94 %	2023	del in 2q
UPN069_r	9	0	49000000	2	not detected				94.31 %	94.07 %	94.55 %	1957	LOH9p
UPN070_r	14	24731434	26078015	1	not detected				78.07 %	75.87 %	80.27 %	75	del in 14q
UPN070_r	14	99699113	100750635	1	not detected				73.22 %	66.10 %	80.33 %	34	del in 14q

T-ALL pediatric_relapsed

UPN070_r	17	0	19151115	1	not detected		83.24 %	82.92 %	83.57 %	1017	del in 17p
UPN070_r	17	29422358	30648308	1	not detected		82.08 %	80.24 %	83.92 %	36	del in 17q
UPN070_r	21	33118577	34436973	1	not detected		80.44 %	79.48 %	81.40 %	58	del in 21q
UPN071_r	7	0	59900000	1	not detected		71.45 %	71.12 %	71.78 %	2723	del 7p
UPN071_r	7	59900000	159138663	3	not detected		66.00 %	64.00 %	69.00 %	3471	dup 7q
UPN072_r	1	23031476	24244069	1	not detected		94.65 %	93.69 %	95.62 %	45	del in 1p
UPN072_r	9	11857975	40813425	1	not detected		86.17 %	84.83 %	87.51 %	1065	del in 9p
UPN072_r	17	40000000	75844453	2	not detected		96.85 %	96.66 %	97.04 %	1317	LOH in 17q
UPN073_r	no data										
UPN074_r	no data										
UPN075_r	no data										
UPN076_r	no data										
UPN077_r	no data										

Supplemental Data 2

					Germline				Tumor				
**ID**	**Chr**	**Start**	**End**	**Type**	**CCF**	**lower**	**upper**	**#SNPs**	**CCF**	**lower**	**upper**	**#SNPs**	**Variant**
UPN078	7	0	52122844	1	not detected				84.34 %	83.74 %	84.94 %	2569	del in 7q
UPN078	9	0	26484190	1	not detected				85.15 %	84.34 %	85.96 %	1378	del in 9q
UPN079	17	48714783	81195210	3	not detected				77.71 %	75.91 %	79.51 %	1452	dup in 17q
UPN080	9	0	44902403	3	not detected				17.45 %	16.65 %	18.26 %	1847	dup9p
UPN081	no data												
UPN082	2	2574901	28706639	2	100.00 %				100.00 %				LOH in 2p
UPN082	2	46256532	53507902	2	100.00 %				100.00 %				LOH in 2p
UPN082	2	164782436	171188134	2	100.00 %				100.00 %				LOH in 2q
UPN082	2	192134975	216823871	2	100.00 %				100.00 %				LOH in 2q
UPN082	3	185912262	188943804	2	100.00 %				100.00 %				LOH in 3q
UPN082	4	0	9926760	2	100.00 %				100.00 %				LOH in 4p
UPN082	5	5110842	24576253	2	100.00 %				100.00 %				LOH in 5p
UPN082	6	33077439	45356966	2	100.00 %				100.00 %				LOH in 6p
UPN082	6	52075963	61000000	2	100.00 %				100.00 %				LOH in 6p
UPN082	6	61000000	73463737	2	100.00 %				100.00 %				LOH in 6q
UPN082	9	29154819	37576500	2	100.00 %				100.00 %				LOH in 9p
UPN082	9	107920936	126819405	2	100.00 %				100.00 %				LOH in 9q
UPN082	10	25200701	28611874	2	100.00 %				100.00 %				LOH in 10p
UPN082	10	125510523	128460849	2	100.00 %				100.00 %				LOH in 10q
UPN082	12	22500913	35800000	2	100.00 %				100.00 %				LOH in 12p
UPN082	12	37891147	63295815	2	100.00 %				100.00 %				LOH in 12q
UPN082	12	108063049	116966247	2	100.00 %				100.00 %				LOH in 12q
UPN082	13	97517984	110819840	2	100.00 %				100.00 %				LOH in 13q
UPN082	15	68882819	92172224	2	100.00 %				100.00 %				LOH in 15q
UPN082	18	65237158	68039954	2	100.00 %				100.00 %				LOH in 18q
UPN082	20	4350387	11174695	2	100.00 %				100.00 %				LOH in 20p
UPN083	no variants												
UPN084	no data												

T-LBL adult_Primary

UPN085	4	71471569	191154276	2	not detected	96.38 %	96.24 %	96.53 %	4491	LOH in 4q
UPN086	9	20005523	21909086	1	not detected	91.23 %	88.89 %	93.58 %	81	del in 9p
UPN086	17	0	24000000	1	not detected	87.24 %	86.27 %	88.22 %	1204	del 17p
UPN086	21	14359894	44479208	3	not detected	71.76 %	69.49 %	74.02 %	1449	dup21q
UPN087	no data									
UPN088	1	0	29562685	2	not detected	92.32 %	92.02 %	92.61 %	1266	LOH in 1p
UPN088	2	0	74985347	2	not detected	90.77 %	90.27 %	91.27 %	3315	LOH in 2p
UPN088	4	55788718	191154276	2	not detected	93.36 %	93.25 %	93.47 %	4814	LOH4q
UPN088	9	0	47300000	3	not detected	16.81 %	16.06 %	17.56 %	1895	dup9p
UPN089	no variants									
UPN090	no data									
UPN091	9	0	35962463	2	not detected	95.09 %	94.74 %	95.45 %	1918	del in 9p
UPN092	1	0	35315737	1	not detected	85.99 %	85.42 %	86.57 %	1530	del in 1p
UPN092	1	35315504	73070449	3	not detected	76.07 %	73.70 %	78.45 %	1479	dup in 1p
UPN092	6	140169779	171115067	1	not detected	84.27 %	83.60 %	84.94 %	1435	del in 6q
UPN092	8	90136687	146364022	3	not detected	95.18 %	92.69 %	97.68 %	2251	dup in 8q
UPN092	19	0	3119534	2	not detected	87.19 %	84.71 %	89.67 %	191	LOH in 19p
UPN093	1	23007736	24847715	1	not detected	96.39 %	95.89 %	96.89 %	84	del in 1p
UPN093	4	30380358	32140465	1	not detected	96.12 %	94.93 %	97.30 %	60	del in 4p
UPN093	8	117202621	118408588	1	not detected	94.99 %	91.52 %	98.46 %	48	del in 8q
UPN093	9	0	47300000	3	not detected	19.01 %	18.11 %	19.92 %	2062	dup9p
UPN093	13	50079991	51382899	1	not detected	95.67 %	94.01 %	97.33 %	45	del in 13q
UPN094	no variants									
UPN095	no data									
UPN096	1	5925692	6895859	1	not detected	82.60 %	79.96 %	85.25 %	28	del in 1p
UPN096	8	100625908	146364022	3	not detected	87.48 %	86.27 %	88.70 %	1841	dup in 8q
UPN096	9	0	40786873	2	not detected	98.21 %	97.75 %	98.68 %	1818	LOH9p
UPN096	14	22087235	22989590	1	not detected	97.87 %	95.55 %	100.19 %	75	del in 14q
UPN097	1	35436232	76387705	3	not detected	74.73 %	73.42 %	76.04 %	1429	dup in 1p
UPN097	1	85905534	108762158	1	not detected	87.48 %	87.02 %	87.93 %	773	del in 1p

UPN097	1	108952455	110760120	3	not detected	74.83 %	68.99 %	80.66 %	82	dup in 1p
UPN097	1	164652404	215890201	2	100.00 %	100.00 %				LOH in 1q
UPN097	2	229503575	235638976	2	100.00 %	100.00 %				LOH in 2q
UPN097	3	22148081	38061659	2	100.00 %	100.00 %				LOH in 3p
UPN097	5	168835809	180915260	2	100.00 %	100.00 %				LOH in 5q
UPN097	8	96688963	106098919	2	100.00 %	100.00 %				LOH in 8q
UPN097	8	125954377	132620252	2	100.00 %	100.00 %				LOH in 8q
UPN097	9	0	39140379	1	not detected	89.43 %	89.04 %	89.83 %	1027	del in 9p
UPN097	9	17624108	49000000	2	100.00 %	100.00 %				LOH in 9p
UPN097	9	49000001	75666354	3	not detected	70.92 %	67.23 %	74.62 %	173	dup in 9q
UPN097	9	75666355	101681167	2	100.00 %	100.00 %				LOH in 9q
UPN097	9	101681168	136460764	3	not detected	75.45 %	74.19 %	76.70 %	1395	dup in 9q
UPN097	9	136460765	141213430	2	100.00 %	100.00 %				LOH in 9q
UPN097	10	112153834	114226900	2	100.00 %	100.00 %				LOH in 10q
UPN097	11	106531021	111038617	2	100.00 %	100.00 %				LOH in 11q
UPN097	15	53901900	54420787	3	not detected	93.44 %	86.80 %	100.09 %	57	dup in 15q
UPN097	15	98457055	101694349	2	100.00 %	100.00 %				LOH in 15q
UPN097	19	53390716	55687462	1	not detected	87.55 %	85.94 %	89.16 %	197	del in 19q
UPN097	20	0	19884606	2	100.00 %	100.00 %				LOH in 20p
UPN098	1	0	28643858	1	not detected	91.41 %	90.68 %	92.14 %	1373	del in 1p
UPN098	2	0	93300000	2	not detected	95.45 %	95.18 %	95.71 %	3809	LOH 2p
UPN098	5	131457395	180915260	1	not detected	91.38 %	90.83 %	91.92 %	2072	del in 5q
UPN098	9	0	34520001	1	not detected	86.44 %	85.53 %	87.36 %	1788	del in 9p
UPN098	17	42287433	81151539	3	not detected	74.42 %	72.02 %	76.82 %	1630	dup in 17q
UPN099	no data									
UPN100	1	81229731	95027807	2	100.00 %	100.00 %				LOH in 1p
UPN100	10	52094428	92911616	2	100.00 %	100.00 %				LOH in 10q
UPN100	12	109029564	119965332	2	100.00 %	100.00 %				LOH in 12q
UPN100	15	97265923	102531392	2	100.00 %	100.00 %				LOH in 15q
UPN100	17	38238839	81195210	2	not detected	94.64 %	94.04 %	95.24 %	1436	LOH in 17q

Supplemental Data 2

UPN101	no data										
UPN102	1	61553815	64144400	3	not detected		83.59 %	79.08 %	88.09 %	118	dup in 1p
UPN102	4	152985445	153262993	1	not detected		97.78 %	96.81 %	98.75 %	17	del in 4q
UPN102	9	20623104	39140379	1	not detected		94.36 %	93.19 %	95.54 %	702	del in 9p
UPN102	11	127411099	129408918	3	not detected		81.48 %	76.81 %	86.15 %	98	dup in 11q
UPN102	16	53862949	54090520	1	not detected		97.03 %	96.13 %	97.93 %	25	del in 16q
UPN102	20	29432371	31970929	3	not detected		115.57 %	101.98 %	129.15 %	69	dup in 20q
UPN103	1	9113226	10495556	1	not detected		94.07 %	91.06 %	97.07 %	57	del in 1p
UPN103	1	10495557	249250620	2	not detected		49.67 %	49.37 %	49.96 %	8500	LOH in 1p, LOH 1q
UPN103	2	0	243199373	2	not detected		49.80 %	49.53 %	50.06 %	9716	dup2
UPN103	3	0	198022430	2	not detected		49.77 %	49.48 %	50.06 %	7761	LOH3
UPN103	4	0	109047057	2	not detected		61.74 %	61.33 %	62.14 %	3800	LOH4p, LOH in 4q
UPN103	4	109047058	191154276	3	not detected		29.12 %	28.46 %	29.78 %	3126	dup in 4q
UPN103	5	0	180915260	2	not detected		49.66 %	49.33 %	49.99 %	6503	LOH5
UPN103	8	0	146364022	2	not detected		49.63 %	49.29 %	49.98 %	5813	LOH8
UPN103	9	0	38561623	3	not detected		18.53 %	17.66 %	19.40 %	1874	dup9p
UPN103	10	0	135534747	2	not detected		49.71 %	49.35 %	50.07 %	5621	LOH10
UPN103	12	0	133851895	2	not detected		49.77 %	49.41 %	50.12 %	5573	LOH12
UPN103	15	0	102531392	2	not detected		49.08 %	48.61 %	49.55 %	3431	LOH15
UPN103	17	38561623	81195210	2	not detected		98.43 %	98.21 %	98.65 %	1618	LOH in 17q
UPN103	X	0	155270560	2	not detected		48.61 %	48.37 %	48.85 %	6798	LOHX
UPN104	no data										
UPN105	no data										
UPN106	no data										
UPN107	1	5037962	8245849	1	not detected		73.81 %	71.05 %	76.58 %	144	del in 1p
UPN107	1	93009438	97544427	1	not detected		70.16 %	67.42 %	72.89 %	161	del in 1p
UPN107	4	0	191154276	3	not detected		61.00 %	59.00 %	64.00 %	4713	dup4
UPN107	7	32524272	51555695	1	not detected		69.52 %	68.12 %	70.91 %	678	del in 7p
UPN107	7	134580334	159138663	1	not detected		81.38 %	80.58 %	82.18 %	1185	del in 7q
UPN107	8	0	143486863	3	not detected		62.00 %	60.00 %	64.00 %	5199	dup8

UPN107	13	48248195	90539818	1	not detected		62.71 %	61.58 %	63.84 %	1354	del in 13q
UPN108	9	0	38919501	3	not detected		17.19 %	16.40 %	17.98 %	1840	dup9p
UPN109	9	0	36743439	2	not detected		99.19 %	98.88 %	99.50 %	1811	LOH in 9p
UPN110	9	0	27047050	2	not detected		88.23 %	87.48 %	88.99 %	1483	LOH in 9p
UPN110	9	131859245	141213430	2	not detected		29.80 %	28.34 %	31.27 %	578	LOH in 9q
UPN111	no data										
UPN112	no data										
UPN113	no data										
UPN114	14	20666516	42300823	3	not detected		67.68 %	64.82 %	70.54 %	955	dup in 14q
UPN114	14	55651083	106878749	1	not detected		96.91 %	96.68 %	97.13 %	2171	del in 14q
UPN115	4	174701814	190472164	1	not detected		29.00 %	26.00 %	31.00 %	908	del in 4q
UPN115	9	133828	21920346	3	not detected		90.82 %	88.29 %	93.34 %	1175	dup in 9p
UPN115	14	84860806	107349540	3	not detected		37.76 %	35.96 %	39.56 %	989	dup14q
UPN115	20	0	63025520	3	not detected		69.30 %	67.00 %	71.00 %	2610	dup20
UPN116	3	114054683	198022430	1	not detected		93.69 %	93.52 %	93.86 %	4080	del in 4q
UPN116	6	0	171115067	3	not detected		24.10 %	23.60 %	24.70 %	9598	dup6
UPN116	9	0	39680014	2	not detected		97.36 %	97.07 %	97.65 %	2219	LOH 9p
UPN116	11	50328682	51581487	1	not detected		95.19 %	90.14 %	100.24 %	9	del in 11p, del in 11q
UPN116	14	19047940	32333343	1	not detected		93.09 %	92.56 %	93.62 %	707	del in 14q
UPN116	14	36674265	37111071	1	not detected		86.39 %	73.55 %	99.24 %	21	del in 14q
UPN116	14	45128328	46256518	3	not detected		70.96 %	53.70 %	88.22 %	22	dup in 14q
UPN116	14	52838514	53744882	3	not detected		88.59 %	74.61 %	102.57 %	27	dup in 14q
UPN116	14	54337673	56549900	3	not detected		83.92 %	77.79 %	90.05 %	96	dup in 14q
UPN116	14	63540278	65680776	3	not detected		84.13 %	78.08 %	90.18 %	82	dup in 14q
UPN116	14	69314938	70220809	3	not detected		85.37 %	63.91 %	106.83 %	21	dup in 14q
UPN116	14	70988762	72023148	3	not detected		77.06 %	62.71 %	91.42 %	37	dup in 14q
UPN116	14	77217104	78693910	3	not detected		81.12 %	75.73 %	86.52 %	95	dup in 14q
UPN116	14	91577876	92630670	1	not detected		93.63 %	90.10 %	97.15 %	56	del in 14q
UPN116	14	95669965	96147568	3	not detected		80.65 %	70.77 %	90.53 %	44	dup in 14q
UPN116	14	99259751	99499206	1	not detected		91.17 %	84.57 %	97.77 %	28	del in 14q

UPN117	9	0	36711221	2	not detected		94.86 %	94.23 %	95.48 %	1863	LOH in 9p
UPN117	11	0	135006516	3	not detected		86.00 %	85.00 %	87.00 %	5390	dup11
UPN117	19	0	59128983	3	not detected		81.00 %	80.00 %	82.00 %	2475	dup19
UPN118	9	0	9953234	3	not detected		82.94 %	80.04 %	85.85 %	599	dup in 9p
UPN118	9	22890505	38705698	3	not detected		78.02 %	74.89 %	81.16 %	622	dup in 9p
UPN118	9	70731742	141068637	3	not detected		82.00 %	80.00 %	83.00 %	2784	dup9q
UPN118	12	11634179	13115894	1	not detected		88.89 %	85.40 %	92.38 %	104	del in 12p
UPN118	18	19519313	32848214	3	not detected		31.89 %	30.07 %	33.72 %	485	dup in 18q
UPN118	18	36147473	78077248	2	not detected		81.18 %	80.79 %	81.58 %	1730	LOH in 18q
UPN119	9	127255540	141213431	3	not detected		65.09 %	62.86 %	67.32 %	953	dup in 9q
UPN119	13	19020095	115169878	3	not detected		67.49 %	66.65 %	68.34 %	6408	dup13q
UPN119	19	16255842	24572955	3	not detected		67.82 %	63.37 %	72.28 %	346	dup in 19p
UPN119	19	27738372	59128983	3	not detected		66.38 %	64.79 %	67.96 %	2259	dup19q
UPN120	1	740281	26667650	1	not detected		81.84 %	81.15 %	82.53 %	1238	del in 1p
UPN120	2	0	167810087	3	not detected		68.76 %	68.16 %	69.36 %	5889	dup2p, dup in 2q
UPN120	2	167810088	185267449	1	not detected		83.40 %	82.88 %	83.93 %	695	del in 2q
UPN120	2	185267450	243199373	3	not detected		68.50 %	67.52 %	69.47 %	2216	dup in 2q
UPN120	3	0	19582271	1	not detected		83.97 %	83.63 %	84.31 %	1196	del in 3p
UPN120	4	0	50400000	3	not detected		40.39 %	39.51 %	41.28 %	1969	dup in 4p
UPN120	4	52683607	93205330	3	not detected		42.72 %	41.63 %	43.80 %	1389	dup in 4q
UPN120	4	92378037	191154276	2	not detected		89.05 %	88.87 %	89.23 %	3369	LOH in 4q
UPN120	5	6810714	14029187	1	not detected		40.68 %	39.36 %	42.01 %	310	del in 5p
UPN120	5	18102468	32539413	3	not detected		64.23 %	61.91 %	66.54 %	428	dup in 5p
UPN120	5	32539414	46303072	3	not detected		33.13 %	31.21 %	35.05 %	428	dup in 5p
UPN120	5	49760627	58938399	1	not detected		44.82 %	43.70 %	45.93 %	349	del in 5q
UPN120	5	116807579	180915260	1	not detected		83.98 %	83.71 %	84.25 %	2544	del in 5q
UPN120	6	0	171115067	2	not detected		40.40 %	40.17 %	40.64 %	8500	LOH6
UPN120	7	0	58050276	1	not detected		83.98 %	83.72 %	84.24 %	2707	del7p
UPN120	8	0	146292681	3	not detected		95.64 %	94.96 %	96.32 %	5848	dup8
UPN120	9	0	38783261	3	not detected		65.53 %	64.17 %	66.89 %	1908	dup9p

UPN120	9	70959750	95604240	3	not detected		57.30 %	55.88 %	58.71 %	933	dup in 9q
UPN120	9	95604241	133046525	1	not detected		83.39 %	83.05 %	83.72 %	1431	del in 9q
UPN120	9	133046526	141213430	3	not detected		59.47 %	57.15 %	61.79 %	476	dup in 9q
UPN120	10	2868377	20478672	1	not detected		83.38 %	82.98 %	83.77 %	1029	del in 10p
UPN120	11	0	135006516	2	not detected		37.09 %	36.77 %	37.41 %	5323	LOH11
UPN120	12	0	7788420	3	not detected		66.36 %	64.20 %	68.52 %	484	dup in 12p
UPN120	12	10917102	133851895	3	not detected		68.17 %	67.50 %	68.83 %	4793	dup in 12p, dup12q
UPN120	13	19020095	115169878	3	not detected		110.75 %	109.86 %	111.64 %	3847	dup13q
UPN120	15	22320346	43869365	1	not detected		83.36 %	82.90 %	83.81 %	775	del in 15q
UPN120	15	43869366	102014745	1	not detected		40.04 %	39.60 %	40.48 %	2568	del in 15q
UPN120	16	55484047	57395486	1	not detected		41.85 %	39.77 %	43.92 %	107	del in 16q
UPN120	17	0	18555537	1	not detected		82.79 %	82.32 %	83.27 %	985	del in 17p
UPN120	17	29128222	31206144	1	not detected		42.95 %	35.33 %	50.58 %	74	del in 17p+q
UPN120	18	0	77894226	3	not detected		31.02 %	30.39 %	31.65 %	3148	dup18
UPN120	20	0	63025520	3	not detected		28.58 %	27.95 %	29.21 %	2781	dup20
UPN120	21	19458097	48129895	1	not detected		83.90 %	83.54 %	84.26 %	1288	del in 21q
UPN121	3	59703278	61018717	3	not detected		91.03 %	87.00 %	95.06 %	133	dup in 3p
UPN121	9	0	30665434	3	not detected		16.77 %	15.94 %	17.60 %	1602	dup in 9p
UPN122	no data										
UPN123	no data										
UPN124_p	9	20115527	22047269	1	not detected		96.52 %	95.76 %	97.29 %	64	del9p

Supplemental Data 2

ID	Chr	Start	End	Type	Germline CCF	Germline lower	Germline upper	Germline #SNPs	Tumor CCF	Tumor lower	Tumor upper	Tumor #SNPs	Variant
UPN124_r	9	20115527	22047269	1	not detected				92.09 %	91.14 %	93.04 %	64	del9p

T-LBL adult_Relapse

Supplemental Data 2

					Germline				Tumor				
ID	Chr	Start	End	Type	CCF	lower	upper	#SNPs	CCF	lower	upper	#SNPs	Variant
UPN125	1	2152776	8150169	1	not detected				72.85 %	71.89 %	73.82 %	316	del in 1p
UPN125	1	9931222	16021144	1	not detected				72.29 %	71.27 %	73.30 %	274	del in 1p
UPN125	1	17221730	24901264	1	not detected				71.88 %	70.98 %	72.79 %	364	del in 1p
UPN125	5	137871981	170720430	2	not detected				85.27 %	84.89 %	85.65 %	1192	LOH in 5q
UPN125	5	170720430	180915260	1	not detected				74.21 %	73.55 %	74.87 %	606	del in 5q
UPN125	9	0	36639060	2	not detected				86.61 %	86.29 %	86.93 %	1750	LOH in 9p
UPN125	14	99355266	106053648	3	not detected				64.68 %	61.58 %	67.77 %	331	dup in 14q
UPN126	no data												
UPN127	21	14359894	48099610	3	not detected				52.11 %	49.47 %	54.75 %	1586	dup21q
UPN128	no data												
UPN129	6	75681568	96872082	1	not detected				33.03 %	31.88 %	34.17 %	677	del in 6q
UPN129	6	96872083	121487840	1	not detected				23.60 %	22.69 %	24.51 %	867	del in 6q
UPN129	9	0	40420994	2	not detected				96.17 %	95.85 %	96.50 %	1778	LOH 9p
UPN130	9	0	38899071	3	not detected				18.23 %	17.34 %	19.11 %	1922	dup in 9p
UPN130	12	12410513	13009119	1	not detected				71.56 %	68.22 %	74.90 %	54	del in 12p
UPN131	9	0	38919501	2	not detected				58.78 %	57.89 %	59.67 %	1773	LOH9p
UPN132	9	0	38899071	3	not detected				17.27 %	16.46 %	18.09 %	1864	dup in 9p
UPN132	13	67445133	67805530	1	not detected				98.62 %	97.68 %	99.56 %	15	del in 13q
UPN133	3	107818841	128247400	2	not detected				100.00 %				LOH in 3q
UPN133	4	14474484	38262811	2	not detected				100.00 %				LOH in 4p
UPN133	4	129569621	138596043	2	not detected				100.00 %				LOH in 4q
UPN133	7	39242146	55179265	2	not detected				100.00 %				LOH in 7p
UPN133	8	24866974	43791691	2	not detected				100.00 %				LOH in 8p
UPN133	8	46925940	77765631	2	not detected				100.00 %				LOH in 8q
UPN133	9	21296347	23296786	1	not detected				85.43 %	80.25 %	90.61 %	#####	del in 9p
UPN133	9	32407080	45755225	2	not detected				100.00 %				LOH in 9p
UPN133	9	70731742	80218340	2	not detected				100.00 %				LOH in 9q
UPN133	10	73857766	84399810	2	not detected				100.00 %				LOH in 10q

T-LBL pediatric_not relapsed

UPN133	10	114130142	125892909	2	not detected	100.00 %				LOH in 10q
UPN133	11	36283728	51581487	2	not detected	100.00 %				LOH in 11p
UPN133	11	54847856	87858441	2	not detected	100.00 %				LOH in 11q
UPN133	13	41126167	61854131	2	not detected	100.00 %				LOH in 13q
UPN133	13	105773160	115169878	2	not detected	100.00 %				LOH in 13q
UPN133	14	95481396	107349540	2	not detected	100.00 %				LOH in 14q
UPN133	15	79987432	93877687	2	not detected	100.00 %				LOH in 15q
UPN133	16	12785099	22962166	2	not detected	100.00 %				LOH in 16p
UPN133	17	0	8597112	2	not detected	100.00 %				LOH in 17p
UPN134	8	128754500	130204854	1	not detected	68.62 %	62.24 %	74.99 %	43	del in 8q
UPN134	9	0	38899071	3	not detected	17.89 %	17.03 %	18.75 %	1915	dup in 9p
UPN134	10	89627829	89855585	1	not detected	91.03 %	77.10 %	104.96 %	14	del in 10q
UPN134	11	33958586	36618126	1	not detected	98.28 %	98.01 %	98.54 %	181	del in 11p
UPN135	9	0	28189113	1	not detected	78.16 %	77.73 %	78.59 %	1582	del in 9p
UPN136	5	35405204	35855954	1	not detected	98.29 %	96.84 %	99.73 %	15	del in 5p
UPN136	5	66561416	70308242	1	not detected	96.80 %	94.05 %	99.56 %	69	del in 5q
UPN136	5	132265889	134674291	1	not detected	98.66 %	98.22 %	99.10 %	104	del in 5q
UPN136	5	169510228	172609525	1	not detected	99.08 %	98.88 %	99.28 %	172	del in 5q
UPN136	5	176717144	178495212	1	not detected	95.54 %	92.16 %	98.92 %	76	del in 5q
UPN136	9	0	38899071	3	not detected	18.10 %	17.24 %	18.96 %	1826	dup in 9p
UPN136	10	89624787	89943622	1	not detected	89.40 %	77.85 %	100.95 %	20	del in 10q
UPN136	14	64305463	69938694	1	not detected	98.43 %	98.13 %	98.73 %	206	del in 14q
UPN136	14	75146103	77570750	1	not detected	98.72 %	98.28 %	99.16 %	116	del in 14q
UPN136	14	99029731	99609320	1	not detected	99.14 %	98.67 %	99.60 %	42	del in 14q
UPN136	14	103002718	103421653	1	not detected	NA	NA	NA	3	del in 14q
UPN137	no variants									
UPN138	9	0	37428097	2	not detected	88.11 %	87.09 %	89.14 %	1800	LOH in 9p
UPN139	9	0	38899017	3	not detected	17.12 %	16.33 %	17.91 %	1819	dup in 9p
UPN139	18	0	78077248	3	not detected	84.00 %	83.00 %	85.00 %	3154	dup18
UPN140	no data									

Supplemental Data 2

UPN141	no data									
UPN142	6	0	78966583	3	not detected	76.83 %	74.60 %	79.05 %	7505	dup6
UPN142	6	79039661	116003773	1	not detected	83.70 %	82.94 %	84.46 %	1067	del in 6q
UPN142	20	0	62960292	3	not detected	73.40 %	71.18 %	75.62 %	2784	dup20
UPN143	4	151637268	190937862	3	not detected	62.14 %	61.69 %	62.59 %	1885	LOH in 4q
UPN143	9	0	38899017	3	not detected	16.75 %	15.99 %	17.51 %	1991	dup in 9p
UPN143	17	31148437	81195210	3	not detected	40.67 %	39.89 %	41.46 %	2070	dup in 17q
UPN144	9	2570295	30426501	1	not detected	82.09 %	80.74 %	83.45 %	1399	del in 9q
UPN145	1	151594736	240695998	3	not detected	32.85 %	32.28 %	33.42 %	3641	dup in 1q
UPN145	5	55086783	79297024	1	not detected	96.69 %	96.40 %	96.98 %	855	del in 5q
UPN145	9	0	38899071	3	not detected	17.63 %	16.82 %	18.45 %	1850	dup in 9p
UPN145	11	118358040	123875515	1	not detected	97.30 %	97.02 %	97.58 %	276	del in 11q
UPN145	13	50603622	51382899	1	not detected	97.30 %	96.53 %	98.06 %	54	del in 13q
UPN146	no data									
UPN147	9	0	141213430	1	NA	98.00 %				del9
UPN148	3	93513538	198022430	1	not detected	97.43 %	97.32 %	97.54 %	3685	del 3q
UPN148	4	185363859	185910783	1	not detected	97.88 %	97.41 %	98.35 %	44	del in 4q
UPN148	4	188817997	191154276	1	not detected	97.26 %	96.66 %	97.86 %	129	del in 4q
UPN148	9	130971290	141213431	2	not detected	85.55 %	84.28 %	86.83 %	591	LOH in 9q
UPN148	14	66528508	100573572	1	not detected	77.97 %	77.58 %	78.37 %	1500	del in 14q
UPN148	19	38666523	59128983	3	not detected	28.87 %	27.69 %	30.05 %	1103	dup in 19q
UPN149	9	0	28468067	1	not detected	81.84 %	81.04 %	82.65 %	1561	del in 9p
UPN149	12	118615643	130345983	1	not detected	42.86 %	41.75 %	43.97 %	603	del in 12q
UPN150	9	0	39239013	1	not detected	93.43 %	93.16 %	93.69 %	1964	del in 9p
UPN151	9	0	38899071	3	not detected	15.99 %	15.32 %	16.67 %	1989	del in 9p
UPN151	X	0	52385843	1	not detected	93.38 %	93.23 %	93.52 %	2662	del in Xp
UPN151	X	52385844	155270560	4	not detected	100.00 %				amp in Xp, ampXq
UPN152	9	0	35862688	2	not detected	93.75 %	93.39 %	94.11 %	1777	LOH in 9p
UPN152	9	35867528	39111928	3	not detected	105.29 %	98.76 %	111.83 %	86	dup in 9p
UPN152	10	89311267	90567151	1	not detected	51.31 %	45.44 %	57.18 %	50	del in 10q

T-LBL pediatric_not relapsed

UPN153	8	0	146364022	3	not detected	33.05 %	32.62 %	33.48 %	5619	dup8
UPN153	9	90212822	141213430	3	not detected	39.96 %	39.14 %	40.79 %	2105	dup in 9q
UPN153	11	0	47892937	3	not detected	30.43 %	29.71 %	31.15 %	2124	dup in 11p
UPN154	6	29372139	35418228	2	not detected	75.72 %	67.09 %	84.34 %	48	LOH in 6p
UPN154	9	0	43414113	1	not detected	98.28 %	97.93 %	98.63 %	2006	del9p
UPN154	9	70731742	141213431	3	not detected	62.88 %	61.94 %	63.82 %	2643	dup9q
UPN154	14	21580430	21737296	1	not detected	98.06 %	90.76 %	105.36 %	3	del in 14q
UPN154	17	37865993	81195210	3	not detected	86.25 %	85.03 %	87.46 %	1756	dup in 17q
UPN154	20	0	8981898	3	not detected	36.98 %	35.50 %	38.47 %	523	dup in 20p
UPN155	9	0	45755225	2	not detected	72.20 %	71.71 %	72.69 %	1853	LOH9p
UPN155	12	11657322	13573768	1	not detected	70.21 %	68.60 %	71.81 %	133	del in 12p
UPN155	14	22097755	22975384	1	not detected	68.63 %	65.87 %	71.38 %	66	del in 14q
UPN155	14	87631498	107349540	1	not detected	50.17 %	49.48 %	50.87 %	887	del in 14q
UPN155	20	0	25651492	3	not detected	49.51 %	48.39 %	50.63 %	1345	dup20p
UPN156	no data									
UPN157	6	78838295	86455108	1	not detected	80.00 %				del in 6q
UPN157	8	0	146364022	3	not detected	50.00 %				dup8
UPN157	9	0	35181291	2	not detected	65.00 %				LOH in 9p
UPN157	20	0	25957169	1	not detected	50.00 %				del20p
UPN157	20	29432371	62818622	3	not detected	50.00 %				dup20q
UPN158	9	0	40185526	1	not detected	100.00 %				del9p
UPN158	9	70731742	141213431	3	not detected	100.00 %				dup9q
UPN159	no data									
UPN160	no data									
UPN161	no data									
UPN162	no data									
UPN163	no data									
UPN164	no data									
UPN165	no data									
UPN166	no data									

UPN167	no data			
UPN168	no data			
UPN169	no data			
UPN170	no data			
UPN171	no data			
UPN172	no data			
UPN173	no data			
UPN174	no data			
UPN175	no data			
UPN176	no data			
UPN177	no data			
UPN178	no data			
UPN179	no data			
UPN180	no data			
UPN181	no data			
UPN182	no data			
UPN183	no data			
UPN184	no data			

Supplemental Data 2

					Germline				Tumor				
**ID**	**Chr**	**Start**	**End**	**Type**	**CCF**	**lower**	**upper**	**#SNPs**	**CCF**	**lower**	**upper**	**#SNPs**	**Variant**
UPN185_p	5	0	136827498	3	not detected				72.00 %	71.10 %	72.90 %	5424	dup5
UPN185_p	5	98916337	124952368	1	not detected				83.34 %	82.87 %	83.81 %	912	del in 5q
UPN185_p	6	70957822	89983131	1	not detected				84.35 %	83.79 %	84.91 %	553	del in 6q
UPN185_p	7	0	58050276	1	not detected				73.46 %	73.12 %	73.79 %	2689	del7p
UPN185_p	7	61070337	159138663	3	not detected				60.08 %	59.29 %	60.87 %	3430	dup7q
UPN185_p	9	0	35897175	2	not detected				92.10 %	91.44 %	92.76 %	1511	LOH9p
UPN185_p	9	21118459	25914625	0	not detected				92.00 %				del in 9p
UPN185_p	9	70907741	141017240	3	not detected				62.06 %	61.14 %	62.99 %	2237	dup9q
UPN185_p	9	131846125	141017240	4	not detected				59.50 %	57.87 %	61.12 %	510	dup in 9q
UPN186_p	1	171510590	213685379	3	not detected				51.86 %	49.26 %	54.45 %	1436	dup in 1q
UPN186_p	1	215911808	234840680	1	not detected				67.67 %	66.26 %	69.08 %	802	del in 1q
UPN186_p	10	86480408	91443450	1	not detected				70.54 %	67.00 %	74.08 %	219	del in 10q
UPN186_p	13	78818381	115091079	3	not detected				52.39 %	49.77 %	55.01 %	1674	dup in 13q
UPN187_p	no data												
UPN188_p	9	0	38919501	2	not detected				86.96 %	86.02 %	87.91 %	1752	LOH in 9p
UPN189_p	9	0	36073497	2	not detected				86.00 %	85.00 %	86.00 %	1677	LOH in 9p
UPN190_p	4	88742318	101108191	2	100.00 %				100.00 %				LOH in 4q
UPN190_p	6	134484015	139645139	2	100.00 %				100.00 %				LOH in 6q
UPN190_p	8	130734461	144807849	2	100.00 %				100.00 %				LOH in 8q
UPN190_p	9	216128	24488294	2	20.00 %				96.83 %	96.71 %	96.96 %	5854	LOH in 9p
UPN190_p	X	3354951	22800911	2	100.00 %				100.00 %				LOH in Xp
UPN190_p	X	67952300	109686425	2	100.00 %				100.00 %				LOH in Xq
UPN191_p	no data												
UPN192_p	1	47698310	47881595	1	not detected				71.12 %	61.12 %	81.11 %	11	del in 1q
UPN192_p	11	33934855	36528431	1	not detected				55.09 %	51.97 %	58.20 %	153	del in 11p
UPN192_p	17	28044101	36049552	2	not detected				51.70 %	49.00 %	54.40 %	309	LOH in 17q
UPN192_p	17	37277386	81195210	2	not detected				61.52 %	60.63 %	62.42 %	1904	LOH in 17q
UPN193_p	4	62715443	63773346	1	not detected				86.01 %	77.34 %	94.68 %	42	del4

T-LBL pediatric_relapsed

UPN193_p	7	141768685	159138663	3	not detected	47.38 %	45.80 %	48.97 %	911	dup in 7q
UPN193_p	9	0	141213431	2	not detected	98.37 %	98.24 %	98.51 %	4725	LOH9
UPN193_p	16	0	3779195	3	not detected	90.76 %	84.79 %	96.73 %	171	dup in 16p
UPN193_p	16	18895142	25050906	3	not detected	86.02 %	82.19 %	89.84 %	198	dup in 16p
UPN193_p	16	26297708	35257261	3	not detected	88.95 %	84.04 %	93.87 %	228	dup in 16p
UPN193_p	16	54498748	67510779	3	not detected	92.35 %	89.46 %	95.24 %	488	dup in 16q
UPN193_p	20	0	63025520	3	not detected	86.94 %	85.92 %	87.95 %	2725	dup20
UPN194_p	no data									
UPN195_p	7	142013744	142447199	2	not detected	88.79 %	79.83 %	97.75 %	21	LOH in 7q
UPN195_p	9	0	33168809	1	not detected	97.33 %	96.98 %	97.69 %	1697	del in 9p
UPN195_p	14	54238121	54420853	1	not detected	63.35 %	57.74 %	68.96 %	7	del in 14q
UPN195_p	20	0	63025520	3	not detected	80.03 %	78.86 %	81.19 %	2556	dup20
UPN196_p	6	70136477	112545288	1	not detected	34.68 %	33.63 %	35.73 %	1320	del in 6q
UPN196_p	9	0	34244358	2	not detected	86.22 %	85.51 %	86.94 %	1702	LOH in 9p
UPN196_p	11	127156972	132946652	1	not detected	71.95 %	69.63 %	74.28 %	388	del in 11q
UPN197_p	7	38277971	50414177	1	not detected	96.23 %	95.47 %	96.99 %	415	del in 7p
UPN197_p	17	35439904	81195210	2	not detected	98.55 %	98.41 %	98.69 %	1775	LOH in 17q
UPN198_p	5	83120842	91804825	1	not detected	85.20 %	82.99 %	87.41 %	195	del in 5q
UPN198_p	5	117224712	149797863	1	not detected	92.20 %	91.51 %	92.89 %	1120	del in 5q
UPN198_p	6	144417730	152707063	1	not detected	92.73 %	91.48 %	93.98 %	341	del in 6q
UPN198_p	7	0	159138663	3	not detected	74.99 %	73.93 %	76.05 %	6058	dup7
UPN198_p	8	0	146364022	3	not detected	46.41 %	45.55 %	47.28 %	5635	dup8
UPN198_p	9	0	38919047	1	not detected	93.20 %	92.47 %	93.94 %	1842	del9p
UPN198_p	9	70731742	141068637	3	not detected	81.10 %	79.54 %	82.67 %	3014	dup9q
UPN198_p	12	7624073	30105836	1	not detected	92.13 %	91.45 %	92.80 %	1057	del in 12p
UPN198_p	17	25268142	81151539	3	not detected	76.81 %	74.97 %	78.65 %	2236	dup17q
UPN198_p	19	247265	18995201	3	not detected	68.65 %	65.56 %	71.74 %	879	del in 19p
UPN199_p	1	5101221	7563670	1	not detected	85.14 %	84.07 %	86.22 %	130	del in 1p
UPN199_p	5	179045300	180915260	1	not detected	84.10 %	82.16 %	86.05 %	89	del in 5q
UPN199_p	9	0	21122352	2	not detected	93.63 %	93.25 %	94.02 %	1181	LOH in 9p

UPN199_p	9	21122352	25926300	1	not detected		64.33 %	59.49 %	69.16 %	193	del in 9p
UPN199_p	9	25926300	38919047	3	not detected		77.60 %	74.97 %	80.22 %	503	dup in 9p
UPN199_p	9	136000445	141213431	2	not detected		91.72 %	90.71 %	92.73 %	284	LOH in 9q
UPN199_p	10	17483123	39150257	1	not detected		85.31 %	84.84 %	85.78 %	757	del in 10p
UPN199_p	11	33901602	36595166	1	not detected		85.54 %	84.80 %	86.27 %	174	del in 11p
UPN199_p	14	53065714	86108154	1	not detected		17.59 %	16.96 %	18.22 %	1312	del in 14q
UPN200_p	no data										
UPN201_p	6	71365227	107475978	1	not detected		20.69 %	19.97 %	21.42 %	1163	del in 6q
UPN201_p	8	99132583	146279735	3	not detected		24.05 %	23.24 %	24.85 %	1797	dup in 8q
UPN201_p	11	88893201	134945120	1	not detected		19.74 %	19.17 %	20.31 %	2113	del in 11q
UPN201_p	20	29432371	62960292	3	not detected		73.99 %	72.49 %	75.50 %	1381	dup20q
UPN202_p	5	52403003	121409850	3	not detected		82.64 %	81.40 %	83.88 %	2182	dup in 5q
UPN202_p	5	131828979	134951695	1	not detected		91.15 %	89.01 %	93.30 %	121	del in 5q
UPN202_p	5	140142174	146696255	1	not detected		92.73 %	91.46 %	93.99 %	206	del in 5q
UPN202_p	5	156531694	159744304	3	not detected		82.30 %	76.89 %	87.70 %	120	dup in 5q
UPN202_p	5	168816501	170108410	3	not detected		92.20 %	85.86 %	98.54 %	96	dup in 5q
UPN202_p	5	173120513	180696860	1	not detected		92.72 %	92.11 %	93.33 %	418	del in 5q
UPN202_p	11	0	135006516	3	not detected		88.73 %	85.79 %	91.66 %	5028	dup11
UPN202_p	14	22909257	30780042	1	not detected		90.79 %	89.52 %	92.06 %	294	del in 14q
UPN202_p	14	33832140	37095489	3	not detected		87.57 %	82.81 %	92.33 %	149	dup in 14q
UPN202_p	14	39901339	49729161	1	not detected		92.92 %	92.03 %	93.82 %	224	del in 14q
UPN202_p	14	57433402	72288249	1	not detected		93.14 %	92.65 %	93.63 %	467	del in 14q
UPN202_p	14	78477684	80606203	1	not detected		92.03 %	88.98 %	95.08 %	60	del in 14q
UPN202_p	14	90499510	98124407	1	not detected		85.90 %	83.77 %	88.02 %	397	del in 14q
UPN202_p	14	99521433	103536749	3	not detected		73.30 %	68.89 %	77.71 %	178	dup in 14q
UPN203_p	3	137373114	149384373	1	not detected		93.27 %	92.48 %	94.05 %	447	del in 3q
UPN203_p	3	173379185	176906607	1	not detected		82.53 %	77.97 %	87.09 %	147	del in 3q
UPN203_p	6	63474976	94766857	1	not detected		92.91 %	92.48 %	93.33 %	1033	del in 6q
UPN203_p	10	89588888	91109343	1	not detected		91.57 %	89.03 %	94.11 %	64	del in 10q
UPN203_p	20	0	60223822	3	not detected		78.50 %	77.20 %	80.00 %	2437	dup20

UPN204_p	8	0	36437573	1	not detected	98.14 %	98.08 %	98.21 %	11387	del in 8p
UPN204_p	8	49000000	51920563	1	not detected	47.85 %	43.38 %	52.32 %	53	del in 8q
UPN204_p	8	59938578	64656906	1	not detected	45.86 %	43.48 %	48.24 %	134	del in 8q
UPN204_p	8	128757780	130055525	1	not detected	74.29 %	71.65 %	76.93 %	59	del in 8q
UPN204_p	9	0	27755942	1	not detected	97.95 %	97.85 %	98.05 %	8073	del in 9p
UPN204_p	12	121297815	123002143	1	not detected	98.27 %	97.99 %	98.55 %	471	del in 12q
UPN205_p	1	19126626	27353142	1	30.00 %	93.09 %	92.02 %	94.16 %	327	del in 1p
UPN205_p	9	133828	39297857	3	not detected	55.83 %	55.26 %	56.40 %	1915	LOH in 9p
UPN205_p	12	52869402	133226035	2	30.00 %	96.84 %	96.70 %	96.98 %	3049	LOH in 12q
UPN205_p	15	85913790	92088994	2	100.00 %	100.00 %				LOH in 15q
UPN206_p	19	0	59132134	3	not detected	66.00 %	64.00 %	67.00 %	2339	dup19
UPN206_p	20	0	63061994	3	not detected	62.00 %	61.00 %	64.00 %	2586	dup20
UPN207_p	no variants									
UPN208_p	9	0	32907608	2	not detected	68.84 %	68.32 %	69.36 %	1673	LOH in 9p
UPN209_p	11	30847247	32874844	1	not detected	52.43 %	48.72 %	56.15 %	53	del in 11p
UPN209_p	12	110696466	111887659	1	not detected	53.69 %	43.96 %	63.42 %	20	del in 12q
UPN210_p	data									
UPN211_p	9	19458272	27303051	1	not detected	86.86 %	84.72 %	88.99 %	393	del in 9p
UPN211_p	13	50204777	51773007	1	not detected	95.11 %	94.51 %	95.71 %	89	del in 13q
UPN211_p	16	3895914	10413681	1	not detected	98.72 %	98.37 %	99.07 %	576	del in 16p
UPN211_p	20	0	62960292	3	not detected	75.50 %	74.00 %	77.00 %	2882	dup 20
UPN185_r	5	0	136827498	3	not detected	76.72 %	75.02 %	78.42 %	1728	dup5
UPN185_r	5	98916337	124952368	1	not detected	87.58 %	87.15 %	88.02 %	912	del in 5q
UPN185_r	5	136827498	180915260	2	not detected	93.52 %	93.31 %	93.74 %	1952	LOH in 5q
UPN185_r	6	70957822	89983131	1	not detected	88.35 %	87.87 %	88.82 %	553	del in 6q
UPN185_r	7	0	58050276	1	not detected	87.18 %	86.90 %	87.47 %	2689	del7p
UPN185_r	7	61070337	142496294	3	not detected	76.48 %	75.42 %	77.55 %	2513	dup7q
UPN185_r	9	0	35897175	2	not detected	94.71 %	94.09 %	95.34 %	1511	LOH9p
UPN185_r	9	21118459	25914625	0	not detected	92.00 %				del in 9p
UPN185_r	9	139406104	141213431	3	not detected	73.95 %	66.84 %	81.06 %	56	dup in 9q

UPN185_r	10	0	39150257	1	not detected	87.76 %	87.48 %	88.05 %	1823	del10p
UPN185_r	10	42389768	135534747	3	not detected	77.62 %	76.38 %	78.87 %	1553	dup10q
UPN185_r	10	84057054	135534747	2	not detected	95.49 %	95.39 %	95.59 %	2257	LOH in 10q
UPN185_r	17	46004828	81195210	3	not detected	75.74 %	74.50 %	76.99 %	1515	dup in 17q
UPN186_r	1	171510590	213685379	3	not detected	95.91 %	87.63 %	104.18 %	44	dup in 1q
UPN186_r	1	215911808	234840680	1	not detected	72.48 %	66.20 %	78.76 %	36	del in 1q
UPN186_r	10	86480408	91443450	1	not detected	80.00 %				del in 10q
UPN186_r	13	78818381	115091079	3	not detected	92.63 %	71.64 %	113.61 %	13	dup in 13q
UPN187_r	5	14782	45860933	3	not detected	75.66 %	74.39 %	76.93 %	1841	dup5p
UPN187_r	5	49491374	67067011	3	not detected	78.92 %	76.41 %	81.43 %	523	dup in 5q
UPN187_r	5	67067011	180917280	2	not detected	96.00 %	95.70 %	96.30 %	3330	LOH in 5q
UPN187_r	7	0	59900000	1	not detected	58.89 %	58.44 %	59.34 %	2694	del7p
UPN187_r	7	59900000	159124173	3	not detected	58.03 %	57.15 %	58.91 %	3443	dup7q
UPN187_r	18	0	78122432	3	not detected	47.52 %	46.74 %	48.30 %	3333	dup18
UPN188_r	data									
UPN189_r	4	0	191154276	1	not detected	55.00 %	55.00 %	55.00 %	6733	del4
UPN189_r	9	0	36073497	2	not detected	99.60 %	99.40 %	99.80 %	1677	LOH in 9p
UPN189_r	17	0	19069543	1	not detected	66.47 %	65.84 %	67.10 %	1058	del in 17p
UPN189_r	17	19069543	21416601	3	not detected	59.74 %	53.36 %	66.12 %	52	dup in 17p
UPN190_r1	1	82154	8835010	1	20.00 %	98.86 %	98.69 %	99.02 %	1641	del in 1p
UPN190_r1	4	88742318	101108191	2	100.00 %	100.00 %				LOH in 4q
UPN190_r1	6	134484015	139645139	2	100.00 %	100.00 %				LOH in 6q
UPN190_r1	8	130734461	144807849	2	100.00 %	100.00 %				LOH in 8q
UPN190_r1	9	216128	24488294	2	20.00 %	99.59 %	99.55 %	99.63 %	5861	LOH in 9p
UPN190_r1	17	0	81060040	3	not detected	92.23 %	89.18 %	95.29 %	12996	dup17
UPN190_r1	X	3354951	22800911	2	100.00 %	100.00 %				LOH in Xp
UPN190_r1	X	67952300	109686425	2	100.00 %	100.00 %				LOH in Xq
UPN191_r	1	114973429	213068595	3	not detected	77.68 %	69.66 %	85.71 %	116	dup in 1q
UPN191_r	7	0	59900000	1	not detected	89.48 %	87.77 %	91.18 %	65	del 7p
UPN191_r	7	59900000	159124173	3	not detected	78.41 %	70.56 %	86.27 %	108	dup 7q

Supplemental Data 2

UPN192_r	1	47698310	47881595	1	not detected		89.43 %	87.19 %	91.67 %	11	del in 1q
UPN192_r	11	33934855	36528431	1	not detected		88.63 %	88.16 %	89.10 %	153	del in 11p
UPN192_r	17	28044101	36049552	2	not detected		70.04 %	69.27 %	70.81 %	309	LOH in 17q
UPN192_r	17	37277386	81195210	2	not detected		93.51 %	93.33 %	93.68 %	1904	LOH in 17q
UPN193_r	4	62715443	63773346	1	not detected		85.59 %	75.44 %	95.74 %	42	del4
UPN193_r	7	141768685	159138663	3	not detected		79.44 %	77.52 %	81.36 %	911	dup in 7q
UPN193_r	9	0	141213431	2	not detected		99.28 %	99.15 %	99.41 %	4725	LOH9
UPN193_r	16	0	3779195	3	not detected		90.76 %	85.00 %	96.52 %	171	dup in 16p
UPN193_r	16	18895142	25050906	3	not detected		83.34 %	79.98 %	86.70 %	198	dup in 16p
UPN193_r	16	26297708	35257261	3	not detected		88.89 %	84.18 %	93.59 %	228	dup in 16p
UPN193_r	16	54498748	67510779	3	not detected		90.83 %	88.11 %	93.55 %	488	dup in 16q
UPN193_r	20	0	63025520	3	not detected		83.35 %	82.46 %	84.23 %	2725	dup20
UPN194_r	no data										
UPN195_r	7	142013744	142447199	2	not detected		83.87 %	74.06 %	93.68 %	21	LOH in 7q
UPN195_r	9	0	33168809	1	not detected		95.08 %	94.81 %	95.35 %	1697	del in 9p
UPN195_r	14	54238121	54420853	1	not detected		87.43 %	83.44 %	91.43 %	7	del in 14q
UPN195_r	20	0	63025520	3	not detected		75.28 %	74.36 %	76.20 %	2556	dup20
UPN196_r	6	70136477	112545288	1	not detected		64.23 %	63.24 %	65.23 %	1320	del in 6q
UPN196_r	9	0	34244358	2	not detected		90.29 %	89.72 %	90.87 %	1702	LOH in 9p
UPN196_r	11	127156972	132946652	1	not detected		79.47 %	77.29 %	81.65 %	388	del in 11q
UPN197_r	7	38277971	50414177	1	not detected		98.85 %	97.79 %	99.92 %	7	del in 7p
UPN197_r	17	35439904	81195210	2	not detected		99.59 %	99.50 %	99.68 %	96	LOH in 17q
UPN198_r	no data										
UPN199_r	no data										
UPN200_r	no data										
UPN201_r1	1	149815536	209067712	3	not detected		81.12 %	80.02 %	82.22 %	2156	dup in 1q
UPN201_r1	5	140877905	148474643	1	not detected		98.68 %	98.50 %	98.85 %	275	del in 5q
UPN201_r1	6	71365227	107475978	1	not detected		98.91 %	98.78 %	99.04 %	1163	del in 6q
UPN201_r1	8	99132583	146279735	3	not detected		79.54 %	78.40 %	80.69 %	1798	dup in 8q
UPN201_r1	11	88893201	134945120	1	not detected		98.64 %	98.40 %	98.87 %	2113	del in 11q

Supplemental Data 2

UPN201_r1	20	29432371	62960292	3	not detected	98.00 %	96.36 %	99.65 %	1382	dup20q
UPN202_r	no data									
UPN203_r	3	137373114	149384373	1	not detected	94.53 %	93.82 %	95.23 %	447	del in 3q
UPN203_r	3	173379185	176906607	1	not detected	83.78 %	79.38 %	88.18 %	147	del in 3q
UPN203_r	6	63474976	94766857	1	not detected	93.72 %	93.17 %	94.27 %	1032	del in 6q
UPN203_r	10	89588888	91109343	1	not detected	94.54 %	92.70 %	96.39 %	64	del in 10q
UPN203_r	20	0	60223822	3	not detected	78.50 %	77.20 %	80.00 %	2437	dup20
UPN204_r	8	0	36437573	1	not detected	74.52 %	72.17 %	76.87 %	42	del in 8p
UPN204_r	8	69020496	125061895	3	not detected	76.06 %	62.79 %	89.33 %	34	dup in 8q
UPN204_r	9	0	27755942	1	not detected	68.93 %	64.28 %	73.57 %	26	del in 9p
UPN204_r	12	121297815	123002143	1	not detected	70.00 %				del in 12q
UPN204_r	17	0	18051447	1	not detected	83.24 %	80.57 %	85.90 %	51	del in 17p
UPN205_r1	1	19126626	27353142	1	30.00 %	96.96 %	95.43 %	98.48 %	24	del in 1p
UPN205_r1	12	52869402	133226035	2	30.00 %	97.61 %	96.13 %	99.10 %	90	LOH in 12q
UPN205_r1	15	85913790	92088994	2	100.00 %	100.00 %				LOH in 15q
UPN206_r	7	87213901	141898023	3	not detected	58.67 %	57.09 %	60.25 %	1781	dup in 7q
UPN206_r	17	0	81195210	3	not detected	60.94 %	59.74 %	62.14 %	3284	dup17
UPN206_r	18	0	78077248	3	not detected	64.76 %	63.62 %	65.91 %	3480	dup18
UPN206_r	19	0	59132134	3	not detected	46.43 %	45.02 %	47.85 %	2549	dup19
UPN206_r	20	0	63061994	3	not detected	64.12 %	62.39 %	65.85 %	1633	dup20
UPN206_r	21	0	48129895	3	not detected	64.12 %	62.39 %	65.85 %	1633	dup21
UPN207_r	no variants									
UPN208_r1	no data									
UPN209_r	11	30847247	32874844	1	not detected	92.11 %	90.92 %	93.31 %	53	del in 11p
UPN209_r	12	110696466	111887659	1	not detected	91.74 %	89.18 %	94.30 %	20	del in 12q
UPN210_r1	5	55422681	57966893	1	30.00 %	25.03 %	22.04 %	28.02 %	83	del in 5q
UPN210_r1	10	27795153	30663275	1	30.00 %	29.73 %	27.67 %	31.79 %	169	del in 10p
UPN210_r1	11	125773768	128807694	1	30.00 %	27.37 %	25.85 %	28.88 %	227	del in 11q
UPN211_r1	9	19458272	27303051	1	not detected	91.88 %	89.78 %	93.98 %	397	del in 9p
UPN211_r1	13	50204777	51773007	1	not detected	98.96 %	98.19 %	99.72 %	87	del in 13q

T-LBL pediatric_relapsed

Supplemental Data 2

UPN211_r1	16	0	90161959	3	not detected	73.00 %	69.00 %	77.00 %	3463	dup16
UPN211_r1	16	3895914	10413681	1	not detected	98.06 %	97.63 %	98.50 %	576	del in 16p
UPN211_r1	20	0	62960292	3	not detected	73.00 %	71.00 %	75.00 %	2882	dup 20
UPN190_r2	1	82154	8835010	1	20.00 %	98.58 %	98.44 %	98.73 %	1641	del in 1p
UPN190_r2	4	88742318	101108191	2	100.00 %	100.00 %				LOH in 4q
UPN190_r2	6	134484015	139645139	2	100.00 %	100.00 %				LOH in 6q
UPN190_r2	8	130734461	144807849	2	100.00 %	100.00 %				LOH in 8q
UPN190_r2	9	216128	24488294	2	20.00 %	99.43 %	99.37 %	99.48 %	5856	LOH in 9p
UPN190_r2	10	89630424	89931019	1	not detected	97.52 %	96.80 %	98.25 %	40	del in 10q
UPN190_r2	17	0	17398278	3	not detected	80.56 %	77.13 %	83.99 %	1109	dup in 17q
UPN190_r2	X	2767637	3227120	3	not detected	89.32 %	78.56 %	100.07 %	116	dup in Xp
UPN190_r2	X	3354951	22800911	2	100.00 %	100.00 %				LOH in Xp
UPN190_r2	X	23673122	54092870	3	not detected	82.82 %	81.32 %	84.32 %	2621	dup in Xp
UPN190_r2	X	67952300	109686425	2	100.00 %	100.00 %				LOH in Xq
UPN201_r2	1	149815536	209067712	3	not detected	85.79 %	84.62 %	86.96 %	2157	dup in 1q
UPN201_r2	5	140877905	148474643	1	not detected	98.71 %	98.48 %	98.94 %	276	del in 5q
UPN201_r2	6	71365227	107475978	1	not detected	98.72 %	98.56 %	98.88 %	1162	del in 6q
UPN201_r2	8	99132583	146279735	3	not detected	83.53 %	82.28 %	84.78 %	1798	dup in 8q
UPN201_r2	11	88893201	134945120	1	not detected	98.50 %	98.26 %	98.75 %	2112	del in 11q
UPN201_r2	20	29432371	62960292	3	not detected	109.88 %	106.56 %	113.20 %	1378	dup20q
UPN205_r2	1	19126626	27353142	1	30.00 %	93.00 %	92.11 %	93.89 %	######	del in 1p
UPN205_r2	12	52869402	133226035	2	30.00 %	96.82 %	96.68 %	96.95 %	2895	LOH in 12q
UPN205_r2	15	85913790	92088994	2	100.00 %	100.00 %				LOH in 15q
UPN208_r2	9	0	32907608	2	not detected	45.60 %	44.81 %	46.39 %	1673	LOH in 9p
UPN210_r2	5	55422681	57966893	1	30.00 %	86.38 %	80.41 %	92.35 %	49	del in 5q
UPN210_r2	10	27795153	30663275	1	30.00 %	103.97 %	88.04 %	119.91 %	75	del in 10p
UPN210_r2	11	125773768	128807694	1	30.00 %	112.56 %	96.91 %	128.21 %	79	del in 11q
UPN211_r2	9	19458272	27303051	1	not detected	93.85 %	92.18 %	95.53 %	396	del in 9p
UPN211_r2	13	50204777	51773007	1	not detected	98.99 %	98.40 %	99.59 %	87	del in 13q
UPN211_r2	16	0	90161959	3	not detected	74.00 %	69.00 %	77.00 %	3463	dup16

UPN211_r2	16	3895914	10413681	1	not detected	98.45 %	97.86 %	99.05 %	576	del in 16p
UPN211_r2	20	0	62960292	3	not detected	74.00 %	72.00 %	75.00 %	2882	dup 20
UPN211_r3	9	19458272	27303051	1	not detected	93.11 %	91.45 %	94.77 %	397	del in 9p
UPN211_r3	13	50204777	51773007	1	not detected	96.22 %	95.62 %	96.82 %	87	del in 13q
UPN211_r3	16	0	90161959	3	not detected	55.00 %	53.00 %	59.00 %	3463	dup16
UPN211_r3	16	3895914	10413681	1	not detected	95.21 %	94.81 %	95.61 %	576	del in 16p
UPN211_r3	20	0	62960292	3	not detected	75.00 %	73.00 %	76.00 %	2882	dup 20
