# Supplementary material for: Molecular variants, clonal evolution and clinical relevance in pediatric and adult T-cell lymphoblastic neoplasia
Source: Blood Cancer J. 2026 Apr 2;16(1):57. doi: 10.1038/s41408-026-01488-w (PMC13066408; doi:10.1038/s41408-026-01488-w)
Supplement: Supplementary file 4 — Supplemental Data 3 [file 41408_2026_1488_MOESM4_ESM.pdf]

Supplemental Data 3

Disease	Category	ID	Variant	Clone	CCF G	CCF P	CCF R1	CCF R2	CCF R3	Evolution P	Evolution R1	Evolution R2	Evolution R3	Nested	Siblings	Parent	Alleles available	Alleles not mutated
T-LBL	pediatric	UPN211	del in 9p	1	0	96	98	98	95	linear	linear	linear	branching (6)	1	0	0	1	1
T-LBL	pediatric	UPN211	del in 13q	1	0	96	98	98	95	linear	linear	linear	branching (6)	1	0	0	1	1
T-LBL	pediatric	UPN211	del in 16p	1	0	96	98	98	95	linear	linear	linear	branching (6)	1	0	0	1	1
T-LBL	pediatric	UPN211	dup20	2	0	75	75	76	75	linear	linear	linear	branching (6)	2	0	1	3	3
T-LBL	pediatric	UPN211	dup16	3	0	0	73	74	62	linear	linear	linear	branching (6)	3	0	2	3	3
T-LBL	pediatric	UPN211	PIK3CA (p.His1047Arg)	4	0	0	71	71	0	linear	linear	linear	branching (6)	4	1	3	2	1
T-LBL	pediatric	UPN211	NT5C2 (p.Leu339Phe)	5	0	0	62	52	0	linear	linear	linear	branching (6)	5	0	4	2	1
T-LBL	pediatric	UPN211	PIK3R1 (p.Gly446_Thr454del)	6	0	0	0	0	62	linear	linear	linear	branching (6)	4	1	3	2	1
T-LBL	pediatric	UPN211	TP53 (p.Arg248Gln)	6	0	0	0	0	62	linear	linear	linear	branching (6)	4	1	3	2	1
T-LBL	pediatric	UPN211	FBXW7 (p.Arg465His)	7	0	0	0	0	5	linear	linear	linear	branching (6)	5	5	6	2	1
T-LBL	pediatric	UPN211	PTEN (p.Thr232fs)	8	0	0	0	0	20	linear	linear	linear	branching (6)	5	5	6	2	1
T-LBL	pediatric	UPN211	PTEN (p.Arg234fs)	9	0	0	0	0	15	linear	linear	linear	branching (6)	5	5	6	2	1
T-LBL	pediatric	UPN211	TP53 (p.Gly245Arg)	10	0	0	0	0	9	linear	linear	linear	branching (6)	5	5	6	2	0
T-LBL	pediatric	UPN211	TP53 (p.Gly245Ser)	11	0	0	0	0	2	linear	linear	linear	branching (6)	5	5	6	2	0
T-LBL	pediatric	UPN211	TP53 (p.Glu51*)	12	0	0	0	0	11	linear	linear	linear	branching (6)	5	5	6	2	0.5

4 time points

Supplemental Data 3

SNVs + Indels

Chr	Pos	Ref	Alt	Germline #REF	Germline #ALT	Germline DP	Germline VAF	Primary #REF	Primary #ALT	Primary DP	Primary VAF	Relapse 1 #REF	Relapse 1 #ALT	Relapse 1 DP	Relapse 1 VAF	Relapse 2 #REF	Relapse 2 #ALT	Relapse 2 DP	Relapse 2 VAF	Relapse 3 #REF	Relapse 3 #ALT	Relapse 3 DP	Relapse 3 VAF
3	178952085	A	G	735	0	736	0.00 %	660	1	665	0.15 %	113	84	197	42.64 %	221	157	379	41.42 %	821	10	833	1.20 %
10	104853038	C	A	639	5	645	0.78 %	624	1	625	0.16 %	121	57	178	32.02 %	273	98	374	26.20 %	678	2	680	0.29 %
5	67589571	TAGGGAAAAAATTACAT GAATATAACAC	T	452	0	452	0.00 %	475	0	476	0.00 %	183	2	185	1.08 %	327	4	333	1.20 %	341	123	465	26.45 %
17	7577538	C	T	701	0	701	0.00 %	860	0	864	0.00 %	212	0	212	0.00 %	424	1	425	0.24 %	592	269	867	31.03 %
4	153249384	C	T	695	0	699	0.00 %	782	0	786	0.00 %	289	0	289	0.00 %	424	0	427	0.00 %	704	35	741	4.72 %
10	89717670	CAC	TACTCCGGAGGATCAT	643	7	650	1.08 %	648	5	653	0.77 %	174	4	176	2.27 %	405	2	407	0.49 %	414	58	467	12.42 %
10	89717672	C	CGAGGGTCAGCA	653	0	655	0.00 %	648	0	653	0.00 %	174	0	176	0.00 %	408	0	409	0.00 %	414	46	472	9.75 %
17	7577548	C	G	664	0	666	0.00 %	840	0	845	0.00 %	204	1	205	0.49 %	416	2	421	0.48 %	782	55	864	6.37 %
17	7577548	C	T	664	1	666	0.15 %	840	2	845	0.24 %	204	0	205	0.00 %	416	0	421	0.00 %	782	23	864	2.66 %
17	7579536	C	A	668	3	676	0.44 %	744	3	749	0.40 %	193	1	195	0.51 %	446	1	449	0.22 %	639	51	691	7.38 %

4 time points

Supplemental Data 3

CNVs

				Germline				Primary				Relapse 1				Relapse 2				Relapse 3				
**Chr**	**Start**	**End**	**Type**	**CCF**	**lower**	**upper**	**#SNPs**	**CCF**	**lower**	**upper**	**#SNPs**	**CCF**	**lower**	**upper**	**#SNPs**	**CCF**	**lower**	**upper**	**#SNPs**	**CCF**	**lower**	**upper**	**#SNPs**	**Varianttype**
9	19458272	27303051	1	not detected				86.86 %	84.72 %	88.99 %	393	91.88 %	89.78 %	93.98 %	397	93.85 %	92.18 %	95.53 %	396	93.11 %	91.45 %	94.77 %	397	CNV
13	50204777	51773007	1	not detected				95.11 %	94.51 %	95.71 %	89	98.96 %	98.19 %	99.72 %	87	98.99 %	98.40 %	99.59 %	87	96.22 %	95.62 %	96.82 %	87	CNV
16	3895914	10413681	1	not detected				98.72 %	98.37 %	99.07 %	576	98.06 %	97.63 %	98.50 %	576	98.45 %	97.86 %	99.05 %	576	95.21 %	94.81 %	95.61 %	576	CNV
20	0	62960292	3	not detected				75.50 %	74.00 %	77.00 %	2882	73.00 %	71.00 %	75.00 %	2882	74.00 %	72.00 %	75.00 %	2882	75.00 %	73.00 %	76.00 %	2882	CNV
16	0	90161959	3	not detected				not detected				73.00 %	69.00 %	77.00 %	3463	74.00 %	69.00 %	77.00 %	3463	55.00 %	53.00 %	59.00 %	3463	CNV
																								SNV/Indel
																								SNV/Indel
																								SNV/Indel
																								SNV/Indel
																								SNV/Indel
																								SNV/Indel
																								SNV/Indel
																								SNV/Indel
																								SNV/Indel
																								SNV/Indel

4 time points

Supplemental Data 3

Disease	Category	ID	Variant	Clone	CCF G	CCF P	CCF R1	CCF R2	Evolution P	Evolution R1	Evolution R2	Nested	Siblings	Parent	Alleles available	Alleles not mutated
T-LBL	pediatric	UPN190	LOH in 4q	1	100	100	100	100	branching (2)	linear	branching (2)	1	0	0	2	2
T-LBL	pediatric	UPN190	LOH in 6q	1	100	100	100	100	branching (2)	linear	branching (2)	1	0	0	2	2
T-LBL	pediatric	UPN190	LOH in 8q	1	100	100	100	100	branching (2)	linear	branching (2)	1	0	0	2	2
T-LBL	pediatric	UPN190	LOH in Xp	1	100	100	100	100	branching (2)	linear	branching (2)	1	0	0	2	2
T-LBL	pediatric	UPN190	LOH in Xq	1	100	100	100	100	branching (2)	linear	branching (2)	1	0	0	2	2
T-LBL	pediatric	UPN190	LOH in 9p	2	20	96	100	100	branching (2)	linear	branching (2)	2	0	1	2	2
T-LBL	pediatric	UPN190	del in 1p	2	20	96	100	100	branching (2)	linear	branching (2)	2	0	1	1	1
T-LBL	pediatric	UPN190	dup17 → only dup in 17q at relapse 2	3	0	81	92	100	branching (2)	linear	branching (2)	3	0	2	3	3
T-LBL	pediatric	UPN190	NOTCH1 (p.Gln2503*)	4	0	60	0	0	branching (2)	linear	branching (2)	4	2	3	2	0.5
T-LBL	pediatric	UPN190	NOTCH1 (p.Val1578del)	4	0	60	0	0	branching (2)	linear	branching (2)	4	2	3	2	0.5
T-LBL	pediatric	UPN190	PIK3CD (p.Cys381Arg)	5	0	30	0	0	branching (2)	linear	branching (2)	4	2	4	2	0
T-LBL	pediatric	UPN190	EZH2 (p.Asp184Asn)	6	0	5	0	0	branching (2)	linear	branching (2)	5	0	5	2	1
T-LBL	pediatric	UPN190	MYB (p.Gln424His)	6	0	5	0	0	branching (2)	linear	branching (2)	6	0	5	2	1
T-LBL	pediatric	UPN190	KMT2C (p.Glu674Lys)	6	0	5	0	0	branching (2)	linear	branching (2)	6	0	5	2	1
T-LBL	pediatric	UPN190	USH2A (p.Thr3635Asn)	6	0	5	0	0	branching (2)	linear	branching (2)	6	0	5	2	1
T-LBL	pediatric	UPN190	PHF6 (p.Phe147Leu)	6	0	5	0	0	branching (2)	linear	branching (2)	6	0	5	2	1
T-LBL	pediatric	UPN190	NOTCH1 (p.Val2443fs)	7	0	20	62	32	branching (2)	linear	branching (2)	4	2	3	2	1
T-LBL	pediatric	UPN190	SETD1B (p.Asp458fs)	7	0	20	62	32	branching (2)	linear	branching (2)	4	2	3	2	1
T-LBL	pediatric	UPN190	dup in Xp	8	0	0	0	68	branching (2)	linear	branching (2)	5	0	3	3	3
T-LBL	pediatric	UPN190	dup in Xp	8	0	0	0	68	branching (2)	linear	branching (2)	5	0	3	3	3
T-LBL	pediatric	UPN190	del in 10q	8	0	0	0	68	branching (2)	linear	branching (2)	5	0	3	1	1
T-LBL	pediatric	UPN190	normal 17p, normal in 17q	9	0	0	0	40	branching (2)	linear	branching (2)	5	0	8	2	2
T-LBL	pediatric	UPN190	USH2A (p.Pro3795Gln)	9	0	0	0	40	branching (2)	linear	branching (2)	5	0	8	2	1
T-LBL	pediatric	UPN190	SETD1B (p.Ser284*)	9	0	0	0	40	branching (2)	linear	branching (2)	5	0	8	2	1
T-LBL	pediatric	UPN201	PIK3CD (p.Glu1045Lys)	1	0	100	100	100	linear	linear	linear	1	0	0	2	1
T-LBL	pediatric	UPN201	dup20q	2	0	74	98	100	linear	linear	linear	2	0	1	3	3
T-LBL	pediatric	UPN201	del in 5q	3	0	21	98	100	linear	linear	linear	3	0	2	1	1
T-LBL	pediatric	UPN201	del in 6q	3	0	21	98	100	linear	linear	linear	3	0	2	1	1
T-LBL	pediatric	UPN201	del in 11q	3	0	21	98	100	linear	linear	linear	3	0	2	1	1
T-LBL	pediatric	UPN201	dup in 8q	4	0	19	80	86	linear	linear	linear	4	0	3	3	3
T-LBL	pediatric	UPN201	dup in 1q	5	0	2	80	86	linear	linear	linear	5	0	4	3	3
T-LBL	pediatric	UPN201	PIK3R1 (p.Gln579_Tyr580del)	5	0	2	80	86	linear	linear	linear	5	0	4	2	1
T-LBL	pediatric	UPN201	NT5C2 (p.Leu406Arg)	6	0	0	7	0	linear	linear	linear	6	0	5	2	1
T-LBL	pediatric	UPN205	LOH in 15q	1	100	100	100	100	branching (3)	branching (2)	linear	1	0	0	2	2
T-LBL	pediatric	UPN205	LOH in 12q	2	30	97	97	97	branching (3)	branching (2)	linear	2	0	1	2	2
T-LBL	pediatric	UPN205	del in 1p	3	27	93	93	93	branching (3)	branching (2)	linear	3	0	2	1	1
T-LBL	pediatric	UPN205	CREBBP (p.Arg1341Pro)	4	25	90	84	89	branching (3)	branching (2)	linear	4	0	3	2	1

3 time points

Supplemental Data 3

			SNVs + Indels																			
							Germline				Primary				Relapse 1				Relapse 2			
**Disease**	**Category**	**ID**	**Chr**	**Pos**	**Ref**	**Alt**	**#REF**	**#ALT**	**DP**	**VAF**	**#REF**	**#ALT**	**DP**	**VAF**	**#REF**	**#ALT**	**DP**	**VAF**	**#REF**	**#ALT**	**DP**	**VAF**
T-LBL	pediatric	UPN190																				
T-LBL	pediatric	UPN190																				
T-LBL	pediatric	UPN190																				
T-LBL	pediatric	UPN190																				
T-LBL	pediatric	UPN190																				
T-LBL	pediatric	UPN190																				
T-LBL	pediatric	UPN190																				
T-LBL	pediatric	UPN190																				
T-LBL	pediatric	UPN190	9	139390684	G	A	2051	0	2056	0.00 %	502	217	720	30.14 %	1753	2	1756	0.11 %	1710	0	1711	0.00 %
T-LBL	pediatric	UPN190	9	139399408	GCAC	G	2018	9	2029	0.44 %	341	119	461	25.81 %	1827	5	1838	0.27 %	2097	2	2100	0.10 %
T-LBL	pediatric	UPN190	1	9779982	T	C	1613	0	1614	0.00 %	219	136	357	38.10 %	1412	1	1417	0.07 %	739	2	742	0.27 %
T-LBL	pediatric	UPN190	7	148525907	C	T	1054	2	1057	0.19 %	714	40	756	5.29 %	1101	0	1103	0.00 %	636	0	638	0.00 %
T-LBL	pediatric	UPN190	6	135518167	A	T	1572	0	1575	0.00 %	2134	52	2188	2.38 %	1507	1	1509	0.07 %	971	0	971	0.00 %
T-LBL	pediatric	UPN190	7	151945499	C	T	1277	0	1278	0.00 %	1674	40	1718	2.33 %	1258	0	1261	0.00 %	824	2	827	0.24 %
T-LBL	pediatric	UPN190	1	215953220	G	T	1423	1	1426	0.07 %	1236	23	1260	1.83 %	1372	0	1375	0.00 %	616	2	618	0.32 %
T-LBL	pediatric	UPN190	X	133547540	T	A	2252	1	2255	0.04 %	2279	55	2334	2.36 %	525	0	525	0.00 %	1246	2	1248	0.16 %
T-LBL	pediatric	UPN190	9	139390863	AC	AGCTTAAGGGGACTT	1932	0	1953	0.00 %	495	55	540	10.19 %	943	503	1579	31.86 %	913	266	913	29.13 %
T-LBL	pediatric	UPN190	12	122248216	G	GC	1809	59	1831	3.22 %	687	0	692	0.00 %	1503	674	1528	44.11 %	658	113	667	16.94 %
T-LBL	pediatric	UPN190																				
T-LBL	pediatric	UPN190																				
T-LBL	pediatric	UPN190																				
T-LBL	pediatric	UPN190																				
T-LBL	pediatric	UPN190	1	215,931,942	G	T	2789	14	2805	0.50 %	1278	13	1293	1.01 %	586	7	594	1.18 %	1287	311	1598	19.46 %
T-LBL	pediatric	UPN190	12	122,247,702	C	A	1387	1	1388	0.07 %	442	2	445	0.45 %	459	2	461	0.43 %	636	171	807	21.19 %
T-LBL	pediatric	UPN201	1	9787030	G	A	673	0	675	0.00 %	1094	1168	2264	51.59 %	448	418	868	48.16 %	303	318	625	50.88 %
T-LBL	pediatric	UPN201																				
T-LBL	pediatric	UPN201																				
T-LBL	pediatric	UPN201																				
T-LBL	pediatric	UPN201																				
T-LBL	pediatric	UPN201																				
T-LBL	pediatric	UPN201																				
T-LBL	pediatric	UPN201	5	67591139	GACCAAT	G	610	0	614	0.00 %	1241	8	1242	0.64 %	373	251	628	39.97 %	227	237	466	50.86 %
T-LBL	pediatric	UPN201	10	104850748	A	C	647	1	648	0.15 %	1846	0	1846	0.00 %	751	29	781	3.71 %	615	0	615	0.00 %
T-LBL	pediatric	UPN205																				
T-LBL	pediatric	UPN205																				
T-LBL	pediatric	UPN205																				
T-LBL	pediatric	UPN205	16	3790511	C	G	713	86	805	10.68 %	889	760	1651	46.03 %	208	153	364	42.03 %	405	330	738	44.72 %

3 time points

Supplemental Data 3

							CNVs																
							Germline				Primary				Relapse 1				Relapse 2				
**Disease**	**Category**	**ID**	**Chr**	**Start**	**End**	**Type**	**CCF**	**lower**	**upper**	**#SNPs**	**CCF**	**lower**	**upper**	**#SNPs**	**CCF**	**lower**	**upper**	**#SNPs**	**CCF**	**lower**	**upper**	**#SNPs**	**Varianttype**
T-LBL	pediatric	UPN190	4	88742318	101108191	2	100.00 %				100.00 %				100.00 %				100.00 %				CNV
T-LBL	pediatric	UPN190	6	134484015	139645139	2	100.00 %				100.00 %				100.00 %				100.00 %				CNV
T-LBL	pediatric	UPN190	8	130734461	144807849	2	100.00 %				100.00 %				100.00 %				100.00 %				CNV
T-LBL	pediatric	UPN190	X	3354951	22800911	2	100.00 %				100.00 %				100.00 %				100.00 %				CNV
T-LBL	pediatric	UPN190	X	67952300	109686425	2	100.00 %				100.00 %				100.00 %				100.00 %				CNV
T-LBL	pediatric	UPN190	9	216128	24488294	2	20.00 %				96.83 %	96.71 %	96.96 %	5854	99.59 %	99.55 %	99.63 %	5861	99.43 %	99.37 %	99.48 %	5856	CNV
T-LBL	pediatric	UPN190	1	82154	8835010	1	20.00 %				NA	NA	NA	NA	98.86 %	98.69 %	99.02 %	1641	98.58 %	98.44 %	98.73 %	1641	CNV
T-LBL	pediatric	UPN190	17	0	81060040	3	not detected				NA	NA	NA	NA	92.23 %	89.18 %	95.29 %	12996	80.56 %	77.13 %	83.99 %	1109	CNV
T-LBL	pediatric	UPN190																					SNV/Indel
T-LBL	pediatric	UPN190																					SNV/Indel
T-LBL	pediatric	UPN190																					SNV/Indel
T-LBL	pediatric	UPN190																					SNV/Indel
T-LBL	pediatric	UPN190																					SNV/Indel
T-LBL	pediatric	UPN190																					SNV/Indel
T-LBL	pediatric	UPN190																					SNV/Indel
T-LBL	pediatric	UPN190																					SNV/Indel
T-LBL	pediatric	UPN190																					SNV/Indel
T-LBL	pediatric	UPN190																					SNV/Indel
T-LBL	pediatric	UPN190	X	23673122	54092870	3	not detected				not detected				not detected				82.82 %	81.32 %	84.32 %	2621	CNV
T-LBL	pediatric	UPN190	X	2767637	3227120	3	not detected				not detected				not detected				89.32 %	78.56 %	100.07 %	116	CNV
T-LBL	pediatric	UPN190	10	89630424	89931019	1	not detected				not detected				not detected				97.52 %	96.80 %	98.25 %	40	CNV
T-LBL	pediatric	UPN190	17	17398278	81060040	2	not detected				former dup17				former dup17				42.01 %	39.57 %	44.45 %	9208	CNV
T-LBL	pediatric	UPN190																					SNV/Indel
T-LBL	pediatric	UPN190																					SNV/Indel
T-LBL	pediatric	UPN201																					SNV/Indel
T-LBL	pediatric	UPN201	20	29432371	62960292	3	not detected				73.99 %	72.49 %	75.50 %	1381	98.00 %	96.36 %	99.65 %	1382	109.88 %	106.56 %	113.20 %	1378	CNV
T-LBL	pediatric	UPN201	5	140877905	148474643	1	not detected				not detected				98.68 %	98.50 %	98.85 %	275	98.71 %	98.48 %	98.94 %	276	CNV
T-LBL	pediatric	UPN201	6	71365227	107475978	1	not detected				20.69 %	19.97 %	21.42 %	1163	98.91 %	98.78 %	99.04 %	1163	98.72 %	98.56 %	98.88 %	1162	CNV
T-LBL	pediatric	UPN201	11	88893201	134945120	1	not detected				19.74 %	19.17 %	20.31 %	2113	98.64 %	98.40 %	98.87 %	2113	98.50 %	98.26 %	98.75 %	2112	CNV
T-LBL	pediatric	UPN201	8	99132583	146279735	3	not detected				24.05 %	23.24 %	24.85 %	1797	79.54 %	78.40 %	80.69 %	1798	83.53 %	82.28 %	84.78 %	1798	CNV
T-LBL	pediatric	UPN201	1	149815536	209067712	3	not detected				not detected				81.12 %	80.02 %	82.22 %	2156	85.79 %	84.62 %	86.96 %	2157	CNV
T-LBL	pediatric	UPN201																					SNV/Indel
T-LBL	pediatric	UPN201																					SNV/Indel
T-LBL	pediatric	UPN205	15	85913790	92088994	2	100.00 %				100.00 %				100.00 %				100.00 %				CNV
T-LBL	pediatric	UPN205	12	52869402	133226035	2	30.00 %				96.84 %	96.70 %	96.98 %	3049	97.61 %	96.13 %	99.10 %	90	96.82 %	96.68 %	96.95 %	2895	CNV
T-LBL	pediatric	UPN205	1	19126626	27353142	1	30.00 %				93.09 %	92.02 %	94.16 %	327	96.96 %	95.43 %	98.48 %	24	93.00 %	92.11 %	93.89 %	94	CNV
T-LBL	pediatric	UPN205																					SNV/Indel

3 time points

T-LBL	pediatric	UPN205	PIK3CA (p.Lys410_Gly411insArgLys)	5	18	2	70	0	branching (3)	branching (2)	linear	5	2	4	2	0.5
T-LBL	pediatric	UPN205	PIK3CA (p.Gln546Lys)	5	18	2	70	0	branching (3)	branching (2)	linear	5	2	4	2	0.5
T-LBL	pediatric	UPN205	STAT3 (p.Arg31Gln)	6	0	0	34	0	branching (3)	branching (2)	linear	6	0	5	2	1
T-LBL	pediatric	UPN205	PIK3CA (p.Gln546His)	7	3	11	2	88	branching (3)	branching (2)	linear	5	2	4	2	1
T-LBL	pediatric	UPN205	NOTCH1 (p.Gly230Arg)	8	0	0	0	11	branching (3)	branching (2)	linear	6	0	7	2	1
T-LBL	pediatric	UPN205	LOH in 9p	9	0	56	0	0	branching (3)	branching (2)	linear	5	2	4	2	2
T-LBL	pediatric	UPN205	PIK3R1 (p.Asn564Asp)	10	0	41	0	0	branching (3)	branching (2)	linear	6	0	9	2	1
T-LBL	pediatric	UPN205	PIK3CA (p.Tyr1021Cys)	11	0	16	0	0	branching (3)	branching (2)	linear	7	0	10	2	1
T-LBL	pediatric	UPN208	LOH in 9p	1	0	71	NA	46	branching (2)	branching (2)	branching (2)	1	0	0	2	2
T-LBL	pediatric	UPN208	PTEN (p.Arg234delinsGlnHisTer)	2	0	69	NA	35	branching (2)	branching (2)	branching (2)	2	0	1	2	1
T-LBL	pediatric	UPN208	RUNX1 (p.Leu472Pro)	3	0	54	NA	4	branching (2)	branching (2)	branching (2)	3	1	2	2	1
T-LBL	pediatric	UPN208	MYB (p.Gln424His)	4	0	41	NA	4	branching (2)	branching (2)	branching (2)	4	0	3	2	1
T-LBL	pediatric	UPN208	SETD1B (p.Ser1053Trp)	4	0	41	NA	4	branching (2)	branching (2)	branching (2)	4	0	3	2	1
T-LBL	pediatric	UPN208	USH2A (p.Thr3635Asn)	5	0	12	NA	4	branching (2)	branching (2)	branching (2)	5	0	4	2	1
T-LBL	pediatric	UPN208	EZH2 (p.Asp184Asn)	5	0	12	NA	4	branching (2)	branching (2)	branching (2)	5	0	4	2	1
T-LBL	pediatric	UPN208	CCND3 (p.Leu292fs)	6	0	13	NA	13	branching (2)	branching (2)	branching (2)	3	1	2	2	1
T-LBL	pediatric	UPN208	CCND3 (p.Arg271fs)	6	0	13	NA	13	branching (2)	branching (2)	branching (2)	3	1	2	2	1
T-LBL	pediatric	UPN210	del in 5q	1	27	NA	57	100	linear	linear	linear	1	0	0	1	1
T-LBL	pediatric	UPN210	del in 10p	1	27	NA	57	100	linear	linear	linear	1	0	0	1	1
T-LBL	pediatric	UPN210	del in 11q	1	27	NA	57	100	linear	linear	linear	1	0	0	1	1
T-LBL	pediatric	UPN210	PTEN (p.Arg233delinsProLeuTer)	2	0	11	0	0	linear	linear	linear	2	0	1	2	1

3 time points

T-LBL	pediatric	UPN205	3	178927464	T	TAAACGG	667	55	667	8.25 %	986	20	986	2.03 %	259	81	261	31.03 %	551	2	555	0.36 %
T-LBL	pediatric	UPN205	3	178936094	C	A	651	17	668	2.54 %	1133	69	1204	5.73 %	300	1	301	0.33 %	278	217	495	43.84 %
T-LBL	pediatric	UPN205	17	40500443	C	T	764	14	779	1.80 %	1477	1	1485	0.07 %	242	49	291	16.84 %	670	10	685	1.46 %
T-LBL	pediatric	UPN205	3	178936096	G	C	593	78	674	11.57 %	1173	7	1182	0.59 %	187	103	291	35.40 %	493	10	505	1.98 %
T-LBL	pediatric	UPN205	9	139417356	C	T	223	0	225	0.00 %	916	0	918	0.00 %	160	0	160	0.00 %	261	15	278	5.40 %
T-LBL	pediatric	UPN205																				
T-LBL	pediatric	UPN205	5	67591097	A	G	783	0	784	0.00 %	829	216	1045	20.67 %	322	0	322	0.00 %	654	0	655	0.00 %
T-LBL	pediatric	UPN205	3	178952007	A	G	773	0	774	0.00 %	1156	104	1260	8.25 %	375	0	375	0.00 %	682	0	683	0.00 %
T-LBL	pediatric	UPN208																				
T-LBL	pediatric	UPN208	10	89717675	CG	CAGCATTA	429	1	430	0.23 %	132	79	217	36.41 %	NA	NA	NA	NA	390	84	481	17.46 %
T-LBL	pediatric	UPN208	21	36164460	A	G	127	1	128	0.78 %	59	22	81	27.16 %	NA	NA	NA	NA	196	3	200	1.50 %
T-LBL	pediatric	UPN208	6	135518167	A	T	579	0	579	0.00 %	301	81	383	21.15 %	NA	NA	NA	NA	926	18	947	1.90 %
T-LBL	pediatric	UPN208	12	122255456	C	G	541	0	541	0.00 %	131	33	164	20.12 %	NA	NA	NA	NA	276	8	284	2.82 %
T-LBL	pediatric	UPN208	1	215953220	G	T	664	1	666	0.15 %	316	20	336	5.95 %	NA	NA	NA	NA	783	10	794	1.26 %
T-LBL	pediatric	UPN208	7	148525907	C	T	546	1	549	0.18 %	463	29	492	5.89 %	NA	NA	NA	NA	621	15	639	2.35 %
T-LBL	pediatric	UPN208	6	41903683	G	GGT	574	0	575	0.00 %	95	6	95	6.32 %	NA	NA	NA	NA	592	46	592	7.77 %
T-LBL	pediatric	UPN208	6	41903745	C	CG	519	0	520	0.00 %	93	6	93	6.45 %	NA	NA	NA	NA	596	34	600	5.67 %
T-LBL	pediatric	UPN210																				
T-LBL	pediatric	UPN210																				
T-LBL	pediatric	UPN210																				
T-LBL	pediatric	UPN210	10	89717672	C	CCCCTGT	749	0	751	0.00 %	835	47	837	5.62 %	592	0	593	0.00 %	508	0	508	0.00 %

3 time points

T-LBL	pediatric	UPN205																	SNV/Indel
T-LBL	pediatric	UPN205																	SNV/Indel
T-LBL	pediatric	UPN205																	SNV/Indel
T-LBL	pediatric	UPN205																	SNV/Indel
T-LBL	pediatric	UPN205																	SNV/Indel
T-LBL	pediatric	UPN205	9	133828	39297857	3	not detected	55.83 %	55.26 %	56.40 %	1915	not detected				not detected			CNV
T-LBL	pediatric	UPN205																	SNV/Indel
T-LBL	pediatric	UPN205																	SNV/Indel
T-LBL	pediatric	UPN208	9	0	32907608	2	not detected	68.84 %	68.32 %	69.36 %	1673	NA	NA	NA	NA	45.60 %	44.81 %	46.39 %	1673	CNV
T-LBL	pediatric	UPN208																	SNV/Indel
T-LBL	pediatric	UPN208																	SNV/Indel
T-LBL	pediatric	UPN208																	SNV/Indel
T-LBL	pediatric	UPN208																	SNV/Indel
T-LBL	pediatric	UPN208																	SNV/Indel
T-LBL	pediatric	UPN208																	SNV/Indel
T-LBL	pediatric	UPN208																	SNV/Indel
T-LBL	pediatric	UPN208																	SNV/Indel
T-LBL	pediatric	UPN210	5	55422681	57966893	1	30.00 %	NA	NA	NA	NA	25.03 %	22.04 %	28.02 %	83	86.38 %	80.41 %	92.35 %	49	CNV
T-LBL	pediatric	UPN210	10	27795153	30663275	1	30.00 %	NA	NA	NA	NA	29.73 %	27.67 %	31.79 %	169	103.97 %	88.04 %	119.91 %	75	CNV
T-LBL	pediatric	UPN210	11	125773768	128807694	1	30.00 %	NA	NA	NA	NA	27.37 %	25.85 %	28.88 %	227	112.56 %	96.91 %	128.21 %	79	CNV
T-LBL	pediatric	UPN210																	SNV/Indel

3 time points

Supplemental Data 3

Disease	Category	ID	Variant	Clone	CCF G	CCF P	CCF R1	Evolution P	Evolution R1	Nested	Siblings	Parent	Alleles available	Alleles not mutated
T-ALL	adult	UPN039	del in 7p	1	0	79	20	linear	linear	1	0	0	1	1
T-ALL	adult	UPN039	del9p	1	0	79	20	linear	linear	1	0	0	1	1
T-ALL	adult	UPN039	dup in 7q	2	0	70	20	linear	linear	2	0	1	3	3
T-ALL	adult	UPN039	dup9q	2	0	70	20	linear	linear	2	0	1	3	3
T-ALL	adult	UPN039	PTEN (p.Glu235fs)	3	0	63	13	linear	linear	3	0	2	2	1
T-ALL	adult	UPN039	MED12 (p.Arg1138Pro)	4	0	0	2	linear	linear	4	0	3	1	0
T-ALL	adult	UPN040	STAT5B (p.Asn642His)	1	0	79	45	linear	linear	1	0	0	2	0
T-ALL	adult	UPN040	LOH6	2	0	79	45	linear	linear	2	0	1	2	2
T-ALL	adult	UPN040	LOH17	2	0	79	45	linear	linear	2	0	1	2	2
T-ALL	adult	UPN040	del9p	3	0	71.5	29	linear	linear	3	0	2	1	1
T-ALL	adult	UPN040	JAK3 (p.Arg657Gln)	4	0	66	28	linear	linear	4	0	3	2	1
T-ALL	adult	UPN040	CNOT3 (p.Arg188His)	4	0	66	28	linear	linear	4	0	3	2	1
T-ALL	adult	UPN040	JAK1 (p.Arg724Cys)	4	0	66	28	linear	linear	4	0	3	2	1
T-ALL	adult	UPN040	JAK1 (p.Cys787Phe)	5	0	3.5	0.5	linear	linear	5	1	4	2	0.5
T-ALL	adult	UPN041	LOH2	1	0	97	92	branching (2)	branching (2)	1	0	0	2	2
T-ALL	adult	UPN041	LOH9	1	0	97	92	branching (2)	branching (2)	1	0	0	2	2
T-ALL	adult	UPN041	NOTCH1 (p.Glu2515*)	1	0	97	92	branching (2)	branching (2)	1	0	0	2	1
T-ALL	adult	UPN041	DNM2 (p.Arg364Cys)	2	0	90	10	branching (2)	branching (2)	2	1	1	2	1
T-ALL	adult	UPN041	SMARCA4 (p.Cys998*)	2	0	90	10	branching (2)	branching (2)	2	1	1	2	1
T-ALL	adult	UPN041	NOTCH1 (p.Ala1700Asp)	2	0	90	10	branching (2)	branching (2)	2	1	1	2	0.5
T-ALL	adult	UPN041	dup13	3	0	80	10	branching (2)	branching (2)	3	0	2	3	3
T-ALL	adult	UPN041	dup20	3	0	80	10	branching (2)	branching (2)	3	0	2	3	3
T-ALL	adult	UPN041	PIK3CA (p.Tyr644His)	4	0	8	10	branching (2)	branching (2)	4	0	3	2	1
T-ALL	adult	UPN041	NOTCH1 (p.Leu1593Pro)	5	0	2	80	branching (2)	branching (2)	2	1	1	2	0.5
T-ALL	adult	UPN041	NT5C2 (p.Arg367Gln)	6	0	0	40	branching (2)	branching (2)	3	0	5	2	1
T-ALL	adult	UPN041	TP53 (p.Arg248Gln)	7	0	0	3	branching (2)	branching (2)	4	0	6	2	1
T-ALL	adult	UPN041	COG1 (p.Asn604Ile)	7	0	0	3	branching (2)	branching (2)	4	0	6	2	1
T-ALL	pediatric	UPN066	LOH in 9p	1	0	79	100	branching (2)	branching (2)	1	0	0	2	2
T-ALL	pediatric	UPN066	dup8	2	0	63	100	branching (2)	branching (2)	2	0	1	3	3
T-ALL	pediatric	UPN066	NOTCH1 (p.Gln2393*)	3	0	51	100	branching (2)	branching (2)	3	0	2	2	1

2 time points

Supplemental Data 3

			SNVs + Indels				Germline				Primary				Relapse 1			
**Disease**	**Category**	**ID**	**Chr**	**Pos**	**Ref**	**Alt**	**#REF**	**#ALT**	**DP**	**VAF**	**#REF**	**#ALT**	**DP**	**VAF**	**#REF**	**#ALT**	**DP**	**VAF**
T-ALL	adult	UPN039																
T-ALL	adult	UPN039																
T-ALL	adult	UPN039																
T-ALL	adult	UPN039																
T-ALL	adult	UPN039	10	89717672	C	CGGCGG	1154	0	1156	0.00 %	1280	408	1281	31.85 %	1291	83	1293	6.42 %
T-ALL	adult	UPN039	X	70348506	G	C	815	1	819	0.12 %	1303	0	1304	0.00 %	695	17	715	2.38 %
T-ALL	adult	UPN040	17	40359729	T	G	934	1	939	0.11 %	248	636	886	71.78 %	549	248	799	31.04 %
T-ALL	adult	UPN040																
T-ALL	adult	UPN040																
T-ALL	adult	UPN040																
T-ALL	adult	UPN040	19	17945969	C	T	1214	1	1217	0.08 %	776	422	1201	35.14 %	1014	132	1148	11.50 %
T-ALL	adult	UPN040	19	54649413	G	A	1357	6	1367	0.44 %	838	415	1254	33.09 %	1131	206	1343	15.34 %
T-ALL	adult	UPN040	1	65310518	G	A	738	0	740	0.00 %	560	243	806	30.15 %	848	144	994	14.49 %
T-ALL	adult	UPN040	1	65309790	C	A	777	0	777	0.00 %	958	12	970	1.24 %	1059	1	1060	0.09 %
T-ALL	adult	UPN041																
T-ALL	adult	UPN041																
T-ALL	adult	UPN041	9	139390648	C	A	825	6	837	0.72 %	504	480	989	48.53 %	485	412	905	45.52 %
T-ALL	adult	UPN041	19	10904493	C	T	622	2	625	0.32 %	412	390	806	48.39 %	632	22	657	3.35 %
T-ALL	adult	UPN041	19	11135027	C	A	823	3	827	0.36 %	540	446	989	45.10 %	886	20	909	2.20 %
T-ALL	adult	UPN041	9	139397702	G	T	873	0	876	0.00 %	658	527	1189	44.32 %	904	28	933	3.00 %
T-ALL	adult	UPN041																
T-ALL	adult	UPN041																
T-ALL	adult	UPN041	3	178937755	T	C	595	32	629	5.09 %	741	34	778	4.37 %	746	48	796	6.03 %
T-ALL	adult	UPN041	9	139399365	A	G	826	3	840	0.36 %	1178	11	1195	0.92 %	594	400	1002	39.92 %
T-ALL	adult	UPN041	10	104852955	C	T	714	0	715	0.00 %	975	1	978	0.10 %	621	158	782	20.20 %
T-ALL	adult	UPN041	17	7577538	C	T	843	0	843	0.00 %	964	1	966	0.10 %	790	15	807	1.86 %
T-ALL	adult	UPN041	17	71197777	A	T	828	0	829	0.00 %	1100	0	1102	0.00 %	964	18	983	1.83 %
T-ALL	pediatric	UPN066																
T-ALL	pediatric	UPN066																
T-ALL	pediatric	UPN066	9	139391014	G	A	2182	0	2188	0.00 %	1679	576	2265	25.43 %	1145	1155	2302	50.17 %

2 time points

Supplemental Data 3

			CNVs				Germline				Primary				Relapse 1				
**Disease**	**Category**	**ID**	**Chr**	**Start**	**End**	**Type**	**CCF**	**lower**	**upper**	**#SNPs**	**CCF**	**lower**	**upper**	**#SNPs**	**CCF**	**lower**	**upper**	**#SNPs**	**Varianttype**
T-ALL	adult	UPN039	7	156880398	159074587	1	not detected				78.69 %	77.30 %	80.07 %	115	19.31 %	17.02 %	21.60 %	115	CNV
T-ALL	adult	UPN039	9	0	49000000	1	not detected				80.00 %	79.00 %	80.00 %	1730	not detected				CNV
T-ALL	adult	UPN039	7	43748	38291362	3	not detected				72.78 %	71.64 %	73.91 %	2118	22.71 %	21.96 %	23.47 %	2118	CNV
T-ALL	adult	UPN039	9	49000000	141213430	3	not detected				69.12 %	68.16 %	70.07 %	2917	not detected				CNV
T-ALL	adult	UPN039																	SNV/Indel
T-ALL	adult	UPN039																	SNV/Indel
T-ALL	adult	UPN040																	SNV/Indel
T-ALL	adult	UPN040	6	0	32798629	2	not detected				83.16 %	82.95 %	83.37 %	2911	44.35 %	43.91 %	44.78 %	2911	CNV
T-ALL	adult	UPN040	17	32662435	81195210	2	not detected				83.09 %	82.84 %	83.34 %	1982	44.57 %	44.05 %	45.09 %	1982	CNV
T-ALL	adult	UPN040	9	0	39162801	1	not detected				71.72 %	71.14 %	72.30 %	2028	28.95 %	28.38 %	29.52 %	2028	CNV
T-ALL	adult	UPN040																	SNV/Indel
T-ALL	adult	UPN040																	SNV/Indel
T-ALL	adult	UPN040																	SNV/Indel
T-ALL	adult	UPN040																	SNV/Indel
T-ALL	adult	UPN041	2	0	81245586	2	not detected				95.24 %	95.13 %	95.35 %	3552	92.49 %	92.37 %	92.60 %	3552	CNV
T-ALL	adult	UPN041	9	0	45755225	2	not detected				95.27 %	95.07 %	95.47 %	1927	92.35 %	92.14 %	92.56 %	1927	CNV
T-ALL	adult	UPN041																	SNV/Indel
T-ALL	adult	UPN041																	SNV/Indel
T-ALL	adult	UPN041																	SNV/Indel
T-ALL	adult	UPN041																	SNV/Indel
T-ALL	adult	UPN041	13	19020095	115169878	3	not detected				81.16 %	80.16 %	82.15 %	4011	16.69 %	16.25 %	17.14 %	4011	CNV
T-ALL	adult	UPN041	20	0	63025520	3	not detected				79.13 %	77.40 %	80.87 %	2790	16.41 %	15.69 %	17.14 %	2790	CNV
T-ALL	adult	UPN041																	SNV/Indel
T-ALL	adult	UPN041																	SNV/Indel
T-ALL	adult	UPN041																	SNV/Indel
T-ALL	adult	UPN041																	SNV/Indel
T-ALL	adult	UPN041																	SNV/Indel
T-ALL	pediatric	UPN066	9	0	36639060	2	not detected				78.94 %	78.54 %	79.34 %	1702	98.00 %	98.00 %	99.00 %	837	CNV
T-ALL	pediatric	UPN066	8	0	146364022	3	not detected				62.96 %	62.35 %	63.57 %	5683	88.05 %	87.26 %	88.85 %	5683	CNV
T-ALL	pediatric	UPN066																	SNV/Indel

2 time points

T-ALL	pediatric	UPN066	NOTCH1 (p.His1611_Tyr1619del)	4	0	29	66	branching (2)	branching (2)	4	1	3	2	0.5
T-ALL	pediatric	UPN066	PIK3R1 (p.Tyr580delinsProThrAspValSer)	4	0	29	66	branching (2)	branching (2)	4	1	3	2	1
T-ALL	pediatric	UPN066	PIK3R1 (p.Asp337_Arg340delinsLys)	5	0	0	66	branching (2)	branching (2)	5	0	4	2	0.5
T-ALL	pediatric	UPN066	NOTCH1 (p.His1611Tyr)	6	0	15	34	branching (2)	branching (2)	4	1	3	2	0.5
T-ALL	pediatric	UPN067	LOH in 9p	1	0	45	58	linear	linear	1	0	0	2	2
T-ALL	pediatric	UPN067	BCL11B (p.Ser765*)	2	0	29	48	linear	linear	2	0	1	2	1
T-ALL	pediatric	UPN067	ZBTB7A (p.Arg28Gln)	2	0	29	48	linear	linear	2	0	1	2	1
T-ALL	pediatric	UPN067	NOTCH1 (p.Ser2513fs)	2	0	29	48	linear	linear	2	0	1	2	1
T-ALL	pediatric	UPN067	del6q	3	0	21	0	linear	linear	3	1	2	1	1
T-ALL	pediatric	UPN067	del in 14q	3	0	21	0	linear	linear	3	1	2	1	1
T-ALL	pediatric	UPN067	NOTCH1 (p.Leu2468fs)	3	0	21	0	linear	linear	3	1	2	2	0
T-ALL	pediatric	UPN067	USP7 (p.Arg340_Ser341insAlaArg)	4	0	10	0	linear	linear	4	0	3	2	1
T-ALL	pediatric	UPN067	NOTCH1 (p.Val1676Phe)	4	0	10	0	linear	linear	4	0	3	2	0
T-ALL	pediatric	UPN067	STAT5B (p.Asn642His)	4	0	10	0	linear	linear	4	0	3	2	1
T-ALL	pediatric	UPN067	PIK3CA (p.Glu542Lys)	4	0	10	0	linear	linear	4	0	3	2	1
T-ALL	pediatric	UPN067	del in 2p	5	0	0	35	linear	linear	3	1	4	1	1
T-ALL	pediatric	UPN067	MSH2 (p.Tyr757*)	5	0	0	35	linear	linear	3	1	4	2	1
T-ALL	pediatric	UPN067	NT5C2 (p.Arg39Gln)	5	0	0	35	linear	linear	3	1	4	2	1
T-ALL	pediatric	UPN067	NOTCH1 (p.Leu1678Pro)	5	0	0	35	linear	linear	3	1	4	2	0.5
T-ALL	pediatric	UPN067	KMT2A (p.Ile3105fs)	6	0	0	26	linear	linear	4	0	5	2	1
T-ALL	pediatric	UPN067	USH2A (p.Gly368Arg)	6	0	0	26	linear	linear	4	0	5	2	1
T-ALL	pediatric	UPN067	KMT2D (p.Gly1235fs)	6	0	0	26	linear	linear	4	0	5	2	1
T-ALL	pediatric	UPN067	EZH2 (p.Asp184Asn)	7	0	0	11	linear	linear	5	0	6	2	1
T-ALL	pediatric	UPN067	NOTCH1 (p.Leu1585Pro)	7	0	0	11	linear	linear	5	0	6	2	0.125
T-ALL	pediatric	UPN067	JAK1 (p.Ser1043Ile)	7	0	0	11	linear	linear	5	0	6	2	1

T-ALL	pediatric	UPN066	9	139399285	AGTAGG GGAAGA TCATCTG CTGGCCG TG	A	1871	0	1872	0.00 %	1389	271	1664	16.29 %	958	532	1494	35.61 %
T-ALL	pediatric	UPN066	5	67591144	ATA	ACCTACG GACGTGT C	1063	6	1069	0.56 %	961	149	1122	13.28 %	497	246	747	32.93 %
T-ALL	pediatric	UPN066	5	67588178	AGATATC TCGAG	GAA	1345	9	1349	0.67 %	1469	0	1473	0.00 %	590	296	911	32.49 %
T-ALL	pediatric	UPN066	9	139399312	G	A	1912	0	1914	0.00 %	1430	121	1553	7.79 %	955	197	1156	17.04 %
T-ALL	pediatric	UPN067																
T-ALL	pediatric	UPN067	14	99640879	G	T	689	0	690	0.00 %	431	80	514	15.56 %	465	170	637	26.69 %
T-ALL	pediatric	UPN067	19	4055148	C	T	2223	0	2228	0.00 %	1425	236	1663	14.19 %	1976	592	2573	23.01 %
T-ALL	pediatric	UPN067	9	139390655	C	CG	2154	0	2164	0.00 %	1555	211	1558	13.54 %	2419	510	2426	21.02 %
T-ALL	pediatric	UPN067																
T-ALL	pediatric	UPN067																
T-ALL	pediatric	UPN067	9	139390789	G	GCGGCC	2315	0	2320	0.00 %	1609	174	1614	10.78 %	2439	0	2444	0.00 %
T-ALL	pediatric	UPN067	16	9009168	A	ACCGGGC	1505	0	1506	0.00 %	1127	84	1131	7.43 %	1370	0	1372	0.00 %
T-ALL	pediatric	UPN067	9	139397775	C	A	2149	1	2154	0.05 %	1593	74	1671	4.43 %	2726	2	2731	0.07 %
T-ALL	pediatric	UPN067	17	40359729	T	G	1348	3	1353	0.22 %	1017	42	1061	3.96 %	1299	2	1307	0.15 %
T-ALL	pediatric	UPN067	3	178936082	G	A	1647	0	1649	0.00 %	1251	48	1304	3.68 %	489	1	491	0.20 %
T-ALL	pediatric	UPN067																
T-ALL	pediatric	UPN067	2	47705471	C	G	1637	2	1642	0.12 %	1327	2	1333	0.15 %	714	168	884	19.00 %
T-ALL	pediatric	UPN067	10	104899222	C	T	1104	2	1106	0.18 %	989	0	994	0.00 %	577	121	698	17.34 %
T-ALL	pediatric	UPN067	9	139397768	A	G	2213	0	2223	0.00 %	1731	0	1736	0.00 %	2351	463	2822	16.41 %
T-ALL	pediatric	UPN067	11	118375914	CA	C	2017	4	2028	0.20 %	1505	4	1514	0.26 %	1301	219	1523	14.38 %
T-ALL	pediatric	UPN067	1	216498688	C	G	1304	2	1309	0.15 %	1113	1	1116	0.09 %	288	40	329	12.16 %
T-ALL	pediatric	UPN067	12	49443666	AC	A	1752	7	1762	0.40 %	1305	1	1307	0.08 %	1801	292	2096	13.93 %
T-ALL	pediatric	UPN067	7	148525907	C	T	1532	1	1539	0.06 %	1110	6	1117	0.54 %	953	43	999	4.30 %
T-ALL	pediatric	UPN067	9	139399389	A	G	2455	2	2463	0.08 %	1764	4	1771	0.23 %	2566	152	2720	5.59 %
T-ALL	pediatric	UPN067	1	65303627	C	A	1337	1	1342	0.07 %	962	2	965	0.21 %	837	65	903	7.20 %

2 time points

T-ALL	pediatric	UPN066														SNV/Indel
T-ALL	pediatric	UPN066														SNV/Indel
T-ALL	pediatric	UPN066														SNV/Indel
T-ALL	pediatric	UPN066														SNV/Indel
T-ALL	pediatric	UPN067	9	0	32542278	2	not detected	44.43 %	43.67 %	45.19 %	1688	57.68 %	56.93 %	58.43 %	1688	CNV
T-ALL	pediatric	UPN067														SNV/Indel
T-ALL	pediatric	UPN067														SNV/Indel
T-ALL	pediatric	UPN067														SNV/Indel
T-ALL	pediatric	UPN067	6	61000000	171115067	1	not detected	21.83 %	21.46 %	22.20 %	3972	not detected				CNV
T-ALL	pediatric	UPN067	14	48000000	107349540	1	not detected	20.76 %	20.30 %	21.22 %	2352	not detected				CNV
T-ALL	pediatric	UPN067														SNV/Indel
T-ALL	pediatric	UPN067														SNV/Indel
T-ALL	pediatric	UPN067														SNV/Indel
T-ALL	pediatric	UPN067														SNV/Indel
T-ALL	pediatric	UPN067														SNV/Indel
T-ALL	pediatric	UPN067	2	43000000	48000000	1	not detected	not detected				31.29 %	28.84 %	33.73 %	269	CNV
T-ALL	pediatric	UPN067														SNV/Indel
T-ALL	pediatric	UPN067														SNV/Indel
T-ALL	pediatric	UPN067														SNV/Indel
T-ALL	pediatric	UPN067														SNV/Indel
T-ALL	pediatric	UPN067														SNV/Indel
T-ALL	pediatric	UPN067														SNV/Indel
T-ALL	pediatric	UPN067														SNV/Indel
T-ALL	pediatric	UPN067														SNV/Indel
T-ALL	pediatric	UPN067														SNV/Indel

Supplemental Data 3

T-ALL	pediatric	UPN067	KMT2D (p.Arg5303His)	7	0	0	11	linear	linear	5	0	6	2	0.5
T-ALL	pediatric	UPN067	NOS3 (p.Ala720Thr)	7	0	0	11	linear	linear	5	0	6	2	1
T-ALL	pediatric	UPN067	PHF6 (p.Arg129*)	7	0	0	11	linear	linear	5	0	6	1	0
T-ALL	pediatric	UPN067	NOTCH1 (p.Glu1724Lys)	7	0	0	11	linear	linear	5	0	6	2	0.125
T-ALL	pediatric	UPN067	STAT3 (p.Ala682Val)	7	0	0	11	linear	linear	5	0	6	2	1
T-ALL	pediatric	UPN067	SETD1B (p.Arg511His)	7	0	0	11	linear	linear	5	0	6	2	0.5
T-ALL	pediatric	UPN067	SETD1B (p.Gln53*)	7	0	0	11	linear	linear	5	0	6	2	0.5
T-ALL	pediatric	UPN067	MYB (p.Gln424His)	8	0	0	5	linear	linear	6	0	7	2	1
T-ALL	pediatric	UPN067	CREBBP (p.Ala2392Thr)	8	0	0	5	linear	linear	6	0	7	2	1
T-ALL	pediatric	UPN067	NOTCH1 (p.Arg1598Pro)	8	0	0	5	linear	linear	6	0	7	2	0
T-ALL	pediatric	UPN068	dup8	1	0	81	90	linear	linear	1	1	0	3	3
T-ALL	pediatric	UPN068	FBXW7 (p.Arg465His)	1	0	81	90	linear	linear	1	0	0	2	1
T-ALL	pediatric	UPN068	NOTCH1 (p.Leu1678Pro)	1	0	81	90	linear	linear	1	0	0	2	1
T-ALL	pediatric	UPN068	KDM6A (p.Val1119_Val1120insGly TyrGlnProPheVal)	2	0	54	61	linear	linear	2	0	1	2	0
T-ALL	pediatric	UPN068	NOTCH3 (p.Asp887His)	3	0	27	0	linear	linear	3	0	2	2	1
T-ALL	pediatric	UPN069	del in 9p	1	0	59	0	branching (6)	linear	1	1	0	1	1
T-ALL	pediatric	UPN069	PHF6 (p.Arg275*)	2	0	56	0	branching (6)	linear	2	0	1	1	0
T-ALL	pediatric	UPN069	FBXW7 (p.Arg465His)	3	0	39	0	branching (6)	linear	3	1	2	2	1
T-ALL	pediatric	UPN069	NOTCH1 (p.Phe1606delinsLeuGlyPro)	4	0	12	0	branching (6)	linear	4	5	3	2	1
T-ALL	pediatric	UPN069	NOTCH1 (p.Phe1606delinsLeuGlyPheSer)	5	0	10	0	branching (6)	linear	4	5	3	2	1
T-ALL	pediatric	UPN069	NOTCH1 (p.Leu1678Pro)	6	0	7	0	branching (6)	linear	4	5	3	2	1
T-ALL	pediatric	UPN069	NOTCH1 (p.Arg1598Pro)	7	0	4	0	branching (6)	linear	4	5	3	2	1
T-ALL	pediatric	UPN069	NOTCH1 (p.Leu1600Gln)	8	0	3	0	branching (6)	linear	4	5	3	2	1
T-ALL	pediatric	UPN069	NOTCH1 (p.Leu1600Pro)	9	0	3	0	branching (6)	linear	4	5	3	2	1
T-ALL	pediatric	UPN069	FBXW7 (p.Gly423Val)	10	0	10	0	branching (6)	linear	3	1	2	2	1
T-ALL	pediatric	UPN069	LOH9p	11	0	0	94	branching (6)	linear	1	1	0	2	1
T-ALL	pediatric	UPN069	FBXW7 (p.Arg465Cys)	12	0	0	87	branching (6)	linear	2	0	11	2	1
T-ALL	pediatric	UPN069	dup in 1q	13	0	0	35	branching (6)	linear	4	0	12	3	3
T-ALL	pediatric	UPN069	del in 2q	13	0	0	35	branching (6)	linear	4	0	12	1	1

2 time points

T-ALL	pediatric	UPN067	12	49418606	C	T	2011	4	2019	0.20 %	1459	3	1466	0.20 %	1984	114	2101	5.43 %
T-ALL	pediatric	UPN067	7	150706063	G	A	2156	0	2159	0.00 %	1648	1	1650	0.06 %	2438	169	2611	6.47 %
T-ALL	pediatric	UPN067	X	133527949	C	T	711	2	715	0.28 %	574	0	575	0.00 %	422	63	485	12.99 %
T-ALL	pediatric	UPN067	9	139396938	C	T	829	0	831	0.00 %	577	0	578	0.00 %	607	52	661	7.87 %
T-ALL	pediatric	UPN067	17	40474356	G	A	1778	0	1782	0.00 %	1339	0	1339	0.00 %	1192	91	1286	7.08 %
T-ALL	pediatric	UPN067	12	122248383	G	A	2401	3	2407	0.12 %	1638	0	1641	0.00 %	2497	170	2674	6.36 %
T-ALL	pediatric	UPN067	12	122242800	C	T	1675	0	1680	0.00 %	1304	0	1310	0.00 %	1495	93	1590	5.85 %
T-ALL	pediatric	UPN067	6	135518167	A	T	2044	1	2052	0.05 %	1423	0	1430	0.00 %	1119	36	1156	3.11 %
T-ALL	pediatric	UPN067	16	3777874	C	T	1951	0	1954	0.00 %	1484	0	1485	0.00 %	2211	43	2264	1.90 %
T-ALL	pediatric	UPN067	9	139399350	C	G	2532	0	2535	0.00 %	1814	0	1815	0.00 %	2893	35	2935	1.19 %
T-ALL	pediatric	UPN068																
T-ALL	pediatric	UPN068	4	153249384	C	T	1053	0	1055	0.00 %	745	514	1264	40.66 %	743	637	1381	46.13 %
T-ALL	pediatric	UPN068	9	139397768	A	G	1399	1	1402	0.07 %	1103	695	1803	38.55 %	1006	789	1804	43.74 %
T-ALL	pediatric	UPN068	X	44942752	G	GTGTCGGATACCAGCCATT	461	0	461	0.00 %	500	274	503	54.47 %	408	253	414	61.11 %
T-ALL	pediatric	UPN068	19	15292520	C	G	1209	0	1214	0.00 %	1425	225	1651	13.63 %	1620	0	1626	0.00 %
T-ALL	pediatric	UPN069																
T-ALL	pediatric	UPN069	X	133549136	C	T	514	0	516	0.00 %	180	228	408	55.88 %	334	0	334	0.00 %
T-ALL	pediatric	UPN069	4	153249384	C	T	1342	1	1346	0.07 %	857	204	1064	19.17 %	725	0	727	0.00 %
T-ALL	pediatric	UPN069	9	139399325	G	GGGCCCC	1847	0	1849	0.00 %	1520	91	1523	5.98 %	1395	0	1399	0.00 %
T-ALL	pediatric	UPN069	9	139399325	G	GGAGAATCCT	1847	0	1849	0.00 %	1520	77	1523	5.06 %	1395	0	1399	0.00 %
T-ALL	pediatric	UPN069	9	139397768	A	G	1618	0	1622	0.00 %	1354	52	1419	3.66 %	1489	0	1495	0.00 %
T-ALL	pediatric	UPN069	9	139399350	C	G	1798	1	1800	0.06 %	1505	32	1538	2.08 %	1472	1	1474	0.07 %
T-ALL	pediatric	UPN069	9	139399344	A	T	1879	0	1888	0.00 %	1507	29	1562	1.86 %	1485	1	1490	0.07 %
T-ALL	pediatric	UPN069	9	139399344	A	G	1879	0	1888	0.00 %	1507	24	1562	1.54 %	1485	1	1490	0.07 %
T-ALL	pediatric	UPN069	4	153249510	C	A	1308	2	1313	0.15 %	1071	56	1127	4.97 %	748	0	749	0.00 %
T-ALL	pediatric	UPN069																
T-ALL	pediatric	UPN069	4	153249385	G	A	1330	0	1336	0.00 %	1043	18	1064	1.69 %	401	313	715	43.78 %
T-ALL	pediatric	UPN069																
T-ALL	pediatric	UPN069																

T-ALL	pediatric	UPN067														SNV/Indel
T-ALL	pediatric	UPN067														SNV/Indel
T-ALL	pediatric	UPN067														SNV/Indel
T-ALL	pediatric	UPN067														SNV/Indel
T-ALL	pediatric	UPN067														SNV/Indel
T-ALL	pediatric	UPN067														SNV/Indel
T-ALL	pediatric	UPN067														SNV/Indel
T-ALL	pediatric	UPN067														SNV/Indel
T-ALL	pediatric	UPN067														SNV/Indel
T-ALL	pediatric	UPN067														SNV/Indel
T-ALL	pediatric	UPN068	8	0	146364022	3	not detected	81.00 %	79.00 %	82.00 %	5506	90.00 %				CNV
T-ALL	pediatric	UPN068														SNV/Indel
T-ALL	pediatric	UPN068														SNV/Indel
T-ALL	pediatric	UPN068														SNV/Indel
T-ALL	pediatric	UPN068														SNV/Indel
T-ALL	pediatric	UPN069	9	20039346	35644453	1	not detected	57.97 %	56.29 %	59.66 %	610	not detected				CNV
T-ALL	pediatric	UPN069														SNV/Indel
T-ALL	pediatric	UPN069														SNV/Indel
T-ALL	pediatric	UPN069														SNV/Indel
T-ALL	pediatric	UPN069														SNV/Indel
T-ALL	pediatric	UPN069														SNV/Indel
T-ALL	pediatric	UPN069														SNV/Indel
T-ALL	pediatric	UPN069														SNV/Indel
T-ALL	pediatric	UPN069														SNV/Indel
T-ALL	pediatric	UPN069														SNV/Indel
T-ALL	pediatric	UPN069	9	0	49000000	2	not detected	not detected				94.31 %	94.07 %	94.55 %	1957	CNV
T-ALL	pediatric	UPN069														SNV/Indel
T-ALL	pediatric	UPN069	1	149732007	249250620	3	not detected	not detected				52.29 %	43.40 %	61.19 %	4151	CNV
T-ALL	pediatric	UPN069	2	200540672	243199373	1	not detected	not detected				36.36 %	35.78 %	36.94 %	2023	CNV

2 time points

Supplemental Data 3

T-ALL	pediatric	UPN069	FBXW7 (p.Glu316*)	13	0	0	80	branching (6)	linear	3	0	12	2	0.5
T-ALL	pediatric	UPN069	NOTCH1 (p.Val1605del)	13	0	0	80	branching (6)	linear	3	0	12	2	1
T-ALL	pediatric	UPN069	USP7 (p.Thr276fs)	14	0	0	35	branching (6)	linear	4	0	13	2	1
T-ALL	pediatric	UPN069	NOTCH1 (p.Leu1574Pro)	15	0	0	3	branching (6)	linear	5	0	14	2	0
T-ALL	pediatric	UPN070	dup in 16p	1	0	85	85	branching (2)	linear	1	0	0	3	3
T-ALL	pediatric	UPN070	PHF6 (p.Arg116*)	2	0	28	83	branching (2)	linear	2	1	1	1	0
T-ALL	pediatric	UPN070	TP53 (p.Gly245Cys)	2	0	28	83	branching (2)	linear	2	1	1	**2**	**1**
T-ALL	pediatric	UPN070	JAK3 (p.Arg657Gln)	2	0	28	83	branching (2)	linear	2	1	1	2	1
T-ALL	pediatric	UPN070	STAT5B (p.Val712Glu)	2	0	28	83	branching (2)	linear	2	1	1	2	1
T-ALL	pediatric	UPN070	JAK1 (p.Arg724His)	2	0	28	83	branching (2)	linear	2	1	1	2	1
T-ALL	pediatric	UPN070	del in 17q	3	0	0	83	branching (2)	linear	3	0	2	1	1
T-ALL	pediatric	UPN070	del in 21q	3	0	0	83	branching (2)	linear	3	0	2	1	1
T-ALL	pediatric	UPN070	del in 17p	3	0	0	83	branching (2)	linear	3	0	2	1	**0**
T-ALL	pediatric	UPN070	del in 14q	4	0	0	78	branching (2)	linear	4	0	3	1	1
T-ALL	pediatric	UPN070	del in 14q	4	0	0	78	branching (2)	linear	4	0	3	1	1
T-ALL	pediatric	UPN070	EZH2 (p.Asp184Asn)	5	0	0	4	branching (2)	linear	5	0	4	2	1
T-ALL	pediatric	UPN070	dup in 9p	6	0	44	0	branching (2)	linear	2	1	1	3	3
T-ALL	pediatric	UPN071	NOTCH1 (p.Arg1586Pro)	1	0	88	0	linear	linear	1	1	0	2	1
T-ALL	pediatric	UPN071	MED12 (p.His11_Arg12del)	2	0	35	0	linear	linear	2	0	1	2	1
T-ALL	pediatric	UPN071	TP53 (p.Arg248Gln)	3	0	0	76	linear	linear	1	1	2	2	1
T-ALL	pediatric	UPN071	del 7p	4	0	0	71	linear	linear	2	0	3	1	1
T-ALL	pediatric	UPN071	dup 7q	5	0	0	66	linear	linear	3	0	4	3	3
T-ALL	pediatric	UPN071	NOTCH1 (p.Leu1574Pro)	6	0	0	41	linear	linear	4	0	5	2	1
T-ALL	pediatric	UPN071	NOS3 (p.Pro592Ala)	7	0	0	4	linear	linear	5	0	6	2	1
T-ALL	pediatric	UPN071	EZH2 (p.Asp184Asn)	7	0	0	4	linear	linear	5	0	6	2	1
T-ALL	pediatric	UPN071	TET2 (p.Gln1055Lys)	7	0	0	4	linear	linear	5	0	6	2	1
T-ALL	pediatric	UPN072	del in 9p	1	0	98	95	branching (12)	linear	1	0	0	1	1
T-ALL	pediatric	UPN072	IL7R (p.Leu243delinsGlyLeuLysCysGly)	2	0	22	0	branching (12)	linear	2	11	1	2	1
T-ALL	pediatric	UPN072	NOTCH1 (p.Pro2512Leu)	3	0	20	0	branching (12)	linear	2	11	1	2	1
T-ALL	pediatric	UPN072	NOTCH1 (p.Val1721Met)	4	0	15	0	branching (12)	linear	2	11	1	2	1

2 time points

T-ALL	pediatric	UPN069	4	153253787	C	A	1145	0	1145	0.00 %	899	1	901	0.11 %	304	206	511	40.31 %
T-ALL	pediatric	UPN069	9	139399328	GACC	G	1827	0	1831	0.00 %	1501	0	1505	0.00 %	802	559	1363	41.01 %
T-ALL	pediatric	UPN069	16	9010890	ACTTTGT<br>TAACTTT<br>TTTGTT	GCA	1133	98	1223	8.01 %	818	0	886	0.00 %	468	112	593	18.89 %
T-ALL	pediatric	UPN069	9	139399422	A	G	1774	2	1778	0.11 %	1583	1	1586	0.06 %	1482	26	1510	1.72 %
T-ALL	pediatric	UPN070																
T-ALL	pediatric	UPN070	X	133527636	C	T	332	0	332	0.00 %	304	94	398	23.62 %	144	406	551	73.68 %
T-ALL	pediatric	UPN070	17	7577548	C	A	1204	1	1205	0.08 %	1313	248	1564	15.86 %	560	1335	1898	70.34 %
T-ALL	pediatric	UPN070	19	17945969	C	T	1253	3	1258	0.24 %	1575	270	1848	14.61 %	2074	1382	3464	39.90 %
T-ALL	pediatric	UPN070	17	40354460	A	T	645	0	648	0.00 %	719	115	834	13.79 %	821	672	1496	44.92 %
T-ALL	pediatric	UPN070	1	65310517	C	T	1080	0	1084	0.00 %	1102	173	1279	13.53 %	1564	925	2498	37.03 %
T-ALL	pediatric	UPN070																
T-ALL	pediatric	UPN070																
T-ALL	pediatric	UPN070																
T-ALL	pediatric	UPN070																
T-ALL	pediatric	UPN070																
T-ALL	pediatric	UPN070	7	148525907	C	T	916	0	917	0.00 %	1116	0	1118	0.00 %	1558	31	1594	1.94 %
T-ALL	pediatric	UPN070																
T-ALL	pediatric	UPN071	9	139399386	C	G	2004	2	2008	0.10 %	860	678	1542	43.97 %	2533	4	2541	0.16 %
T-ALL	pediatric	UPN071	X	70338634	ACACCGG	A	1542	0	1543	0.00 %	677	373	1052	35.46 %	1779	0	1780	0.00 %
T-ALL	pediatric	UPN071	17	7577538	C	T	1869	0	1869	0.00 %	1351	0	1353	0.00 %	1284	792	2079	38.10 %
T-ALL	pediatric	UPN071																
T-ALL	pediatric	UPN071																
T-ALL	pediatric	UPN071	9	139399422	A	G	2008	0	2011	0.00 %	1509	0	1513	0.00 %	2182	569	2756	20.65 %
T-ALL	pediatric	UPN071	7	150700420	C	G	1219	1	1223	0.08 %	992	1	994	0.10 %	2083	44	2128	2.07 %
T-ALL	pediatric	UPN071	7	148525907	C	T	1274	0	1279	0.00 %	888	0	888	0.00 %	1375	30	1407	2.13 %
T-ALL	pediatric	UPN071	4	106158199	C	A	1467	0	1471	0.00 %	1177	0	1180	0.00 %	1256	23	1282	1.79 %
T-ALL	pediatric	UPN072																
T-ALL	pediatric	UPN072	5	35874570	ACTA	GGGGTTA<br>AAGTGTG<br>GT	1387	6	1393	0.43 %	1086	150	1240	12.10 %	1525	0	1528	0.00 %
T-ALL	pediatric	UPN072	9	139390656	G	A	1873	2	1879	0.11 %	1682	214	1903	11.25 %	2153	3	2163	0.14 %
T-ALL	pediatric	UPN072	9	139397640	C	T	1979	0	1982	0.00 %	1776	172	1951	8.82 %	2290	0	2298	0.00 %

T-ALL	pediatric	UPN069														SNV/Indel
T-ALL	pediatric	UPN069														SNV/Indel
T-ALL	pediatric	UPN069														SNV/Indel
T-ALL	pediatric	UPN069														SNV/Indel
T-ALL	pediatric	UPN070	16	14975292	16675394	3	not detected	84.88 %	78.66 %	91.10 %	58	not detected				CNV
T-ALL	pediatric	UPN070														SNV/Indel
T-ALL	pediatric	UPN070														SNV/Indel
T-ALL	pediatric	UPN070														SNV/Indel
T-ALL	pediatric	UPN070														SNV/Indel
T-ALL	pediatric	UPN070														SNV/Indel
T-ALL	pediatric	UPN070	17	29422358	30648308	1	not detected	not detected				82.08 %	80.24 %	83.92 %	75	CNV
T-ALL	pediatric	UPN070	21	33118577	34436973	1	not detected	not detected				80.44 %	79.48 %	81.40 %	34	CNV
T-ALL	pediatric	UPN070	17	0	19151115	1	not detected	not detected				83.24 %	82.92 %	83.57 %	36	CNV
T-ALL	pediatric	UPN070	14	24731434	26078015	1	not detected	not detected				78.07 %	75.87 %	80.27 %	58	CNV
T-ALL	pediatric	UPN070	14	99699113	100750635	1	not detected	not detected				73.22 %	66.10 %	80.33 %	1017	CNV
T-ALL	pediatric	UPN070														SNV/Indel
T-ALL	pediatric	UPN070	9	0	31181790	3	not detected	44.08 %	42.73 %	45.44 %	1540	not detected				CNV
T-ALL	pediatric	UPN071														SNV/Indel
T-ALL	pediatric	UPN071														SNV/Indel
T-ALL	pediatric	UPN071														SNV/Indel
T-ALL	pediatric	UPN071	7	0	59900000	1	not detected	not detected				71.45 %	71.12 %	71.78 %	2723	CNV
T-ALL	pediatric	UPN071	7	59900000	159138663	3	not detected	not detected				66.00 %	64.00 %	69.00 %	3471	CNV
T-ALL	pediatric	UPN071														SNV/Indel
T-ALL	pediatric	UPN071														SNV/Indel
T-ALL	pediatric	UPN071														SNV/Indel
T-ALL	pediatric	UPN071														SNV/Indel
T-ALL	pediatric	UPN072	9	11857975	40813425	1	not detected	92.59 %	91.51 %	93.66 %	1065	86.17 %	84.83 %	87.51 %	1065	CNV
T-ALL	pediatric	UPN072														SNV/Indel
T-ALL	pediatric	UPN072														SNV/Indel
T-ALL	pediatric	UPN072														SNV/Indel

T-ALL	pediatric	UPN072	KRAS (p.Ala146Thr)	5	0	12	0	branching (12)	linear	2	11	1	2	1
T-ALL	pediatric	UPN072	LOH in 17q	6	0	7	95	branching (12)	linear	2	11	1	2	2
T-ALL	pediatric	UPN072	del in 1p	6	0	7	95	branching (12)	linear	2	11	1	1	1
T-ALL	pediatric	UPN072	STAT5B (p.Asn642His)	6	0	7	95	branching (12)	linear	2	11	1	2	0
T-ALL	pediatric	UPN072	NOTCH1 (p.His1591_Phe1592insTyrPheAsnAsnSerSerPheHis)	7	0	6	0	branching (12)	linear	2	11	1	2	1
T-ALL	pediatric	UPN072	NOTCH1 (p.Ala1741_Ala1742insAspSerPheArgLeuTrpTyrGlyAlaProPro)	8	0	3	0	branching (12)	linear	2	11	1	2	1
T-ALL	pediatric	UPN072	NOTCH1 (p.Ala1742_Ala1743insSerSerThrSerAsnTyrValAlaAlaAla)	9	0	3	0	branching (12)	linear	2	11	1	2	1
T-ALL	pediatric	UPN072	TP53 (p.Cys238Arg)	10	0	3	0	branching (12)	linear	2	11	1	2	1
T-ALL	pediatric	UPN072	NOTCH1 (p.Ala1701Pro)	11	0	3	0	branching (12)	linear	2	11	1	2	1
T-ALL	pediatric	UPN072	NOTCH1 (p.Leu1600Pro)	12	0	2	0	branching (12)	linear	2	11	1	2	1
T-ALL	pediatric	UPN072	NOTCH1 (p.Leu1678Pro)	13	0	2	0	branching (12)	linear	2	11	1	2	1
T-ALL	pediatric	UPN072	NOTCH1 (p.Val1578del)	14	0	6	80	branching (12)	linear	3	0	6	2	1
T-ALL	pediatric	UPN072	FBXW7 (p.Arg689Gln)	15	0	0	34	branching (12)	linear	4	0	14	2	1
T-LBL	adult	UPN124	del9p	1	0	96	96	branching (2)	branching (2)	1	0	0	1	1
T-LBL	adult	UPN124	NOTCH1 (p.Leu1596His)	2	0	43	96	branching (2)	branching (2)	2	0	1	2	1
T-LBL	adult	UPN124	NOTCH1 (p.Leu1678Pro)	3	0	38	0	branching (2)	branching (2)	3	2	2	2	1
T-LBL	adult	UPN124	NOTCH1 (p.Arg1598Pro)	4	0	6	0	branching (2)	branching (2)	4	0	3	2	0
T-LBL	adult	UPN124	STAT5B (p.Asn642His)	4	0	6	0	branching (2)	branching (2)	4	0	3	2	1
T-LBL	adult	UPN124	JAK3 (p.Val674Ala)	4	0	6	0	branching (2)	branching (2)	4	0	3	2	1
T-LBL	adult	UPN124	NOTCH1 (p.His1611_Tyr1619delinsProIleGlnArgGly)	5	0	3	4	branching (2)	branching (2)	3	2	2	2	0

2 time points

Supplemental Data 3

T-ALL	pediatric	UPN072	12	25378562	C	T	1302	3	1311	0.23 %	1225	88	1316	6.69 %	1182	0	1184	0.00 %
T-ALL	pediatric	UPN072																
T-ALL	pediatric	UPN072																
T-ALL	pediatric	UPN072	17	40359729	T	G	1314	2	1318	0.15 %	1162	56	1218	4.60 %	65	1183	1251	94.56 %
T-ALL	pediatric	UPN072	9	139399368	A	AAGTGGAAGGAGCTGTTGTTAAAGT	2264	0	2275	0.00 %	2175	79	2190	3.61 %	2558	0	2564	0.00 %
T-ALL	pediatric	UPN072	9	139396883	G	GCCGGAGGAGCCCCATACCAAAGCCGAAAAGAAT	1156	0	1159	0.00 %	1209	28	1211	2.31 %	1213	0	1217	0.00 %
T-ALL	pediatric	UPN072	9	139396881	C	CGGCCGCCGCCACGTAATTAGAAGTAGAGGA	1171	0	1176	0.00 %	1201	26	1203	2.16 %	1236	0	1239	0.00 %
T-ALL	pediatric	UPN072	17	7577569	A	G	1961	0	1964	0.00 %	1685	37	1726	2.14 %	2369	0	2378	0.00 %
T-ALL	pediatric	UPN072	9	139397700	C	G	2321	3	2333	0.13 %	2102	43	2149	2.00 %	2648	0	2654	0.00 %
T-ALL	pediatric	UPN072	9	139399344	A	G	2375	1	2381	0.04 %	2223	38	2265	1.68 %	2588	0	2593	0.00 %
T-ALL	pediatric	UPN072	9	139397768	A	G	2229	1	2234	0.04 %	2001	22	2029	1.08 %	2702	0	2718	0.00 %
T-ALL	pediatric	UPN072	9	139399408	GCAC	G	2277	13	2293	0.57 %	2220	80	2307	3.47 %	1562	1022	2584	39.55 %
T-ALL	pediatric	UPN072	4	153244091	C	T	1574	0	1578	0.00 %	1481	0	1483	0.00 %	1532	321	1860	17.26 %
T-LBL	adult	UPN124																
T-LBL	adult	UPN124	9	139399356	A	T	1773	0	1776	0.00 %	1472	404	1879	21.50 %	461	467	942	49.58 %
T-LBL	adult	UPN124	9	139397768	A	G	1751	3	1758	0.17 %	1496	354	1854	19.09 %	840	2	843	0.24 %
T-LBL	adult	UPN124	9	139399350	C	G	1782	0	1783	0.00 %	1831	69	1907	3.62 %	956	3	964	0.31 %
T-LBL	adult	UPN124	17	40359729	T	G	1127	1	1130	0.09 %	1018	32	1052	3.04 %	694	3	699	0.43 %
T-LBL	adult	UPN124	19	17945918	A	G	1782	0	1783	0.00 %	1841	38	1884	2.02 %	853	0	858	0.00 %
T-LBL	adult	UPN124	9	139399287	TAGGGGAAGATCATCTGCTGGCCGT	CCCCTCTGGATAG	1555	16	1567	1.02 %	1736	22	1747	1.26 %	937	18	951	1.89 %

2 time points

T-ALL	pediatric	UPN072														SNV/Indel
T-ALL	pediatric	UPN072	17	40000000	75844453	2	not detected	not detected				96.85 %	96.66 %	97.04 %	1317	CNV
T-ALL	pediatric	UPN072	1	23031476	24244069	1	not detected	not detected				94.65 %	93.69 %	95.62 %	45	CNV
T-ALL	pediatric	UPN072														SNV/Indel
T-ALL	pediatric	UPN072														SNV/Indel
T-ALL	pediatric	UPN072														SNV/Indel
T-ALL	pediatric	UPN072														SNV/Indel
T-ALL	pediatric	UPN072														SNV/Indel
T-ALL	pediatric	UPN072														SNV/Indel
T-ALL	pediatric	UPN072														SNV/Indel
T-ALL	pediatric	UPN072														SNV/Indel
T-ALL	pediatric	UPN072														SNV/Indel
T-ALL	pediatric	UPN072														SNV/Indel
T-LBL	adult	UPN124	9	20115527	22047269	1	not detected	96.52 %	95.76 %	97.29 %	64	92.09 %	91.14 %	93.04 %	64	CNV
T-LBL	adult	UPN124														SNV/Indel
T-LBL	adult	UPN124														SNV/Indel
T-LBL	adult	UPN124														SNV/Indel
T-LBL	adult	UPN124														SNV/Indel
T-LBL	adult	UPN124														SNV/Indel
T-LBL	adult	UPN124														SNV/Indel

Supplemental Data 3

T-LBL	adult	UPN124	CREBBP (p.Arg1664His)	6	0	0	7	branching (2)	branching (2)	3	2	2	2	1
T-LBL	pediatric	UPN185	LOH9p	1	0	91	94	linear	linear	1	0	0	2	2
T-LBL	pediatric	UPN185	del in 9p	1	0	91	94	linear	linear	1	0	0	0	0
T-LBL	pediatric	UPN185	del in 6q	2	0	84	88	linear	linear	2	0	1	1	1
T-LBL	pediatric	UPN185	del in 5q	2	0	84	88	linear	linear	2	0	1	1	1
T-LBL	pediatric	UPN185	del7p	3	0	73	87	linear	linear	3	0	2	1	1
T-LBL	pediatric	UPN185	dup5	4	0	72	79	linear	linear	4	0	3	3	3
T-LBL	pediatric	UPN185	dup7q	5	0	61	77	linear	linear	5	0	4	3	3
T-LBL	pediatric	UPN185	dup9q	6	0	60	0	linear	linear	6	1	5	3	3
T-LBL	pediatric	UPN185	dup in 9q	6	0	60	0	linear	linear	6	1	5	3	3
T-LBL	pediatric	UPN185	PTEN (p.Arg233fs_a)	7	0	60	0	linear	linear	7	1	6	2	0
T-LBL	pediatric	UPN185	PTEN (p.Arg234fs)	7	0	60	0	linear	linear	7	1	6	2	0
T-LBL	pediatric	UPN185	PIK3CA (p.Glu542Lys)	8	0	12	0	linear	linear	8	0	7	2	1
T-LBL	pediatric	UPN185	dup in 17q	9	0	0	77	linear	linear	6	1	5	3	3
T-LBL	pediatric	UPN185	LOH in 10q	9	0	0	77	linear	linear	6	1	5	2	2
T-LBL	pediatric	UPN185	del10p	9	0	0	77	linear	linear	6	1	5	1	1
T-LBL	pediatric	UPN185	dup10q	9	0	0	77	linear	linear	6	1	5	3	3
T-LBL	pediatric	UPN185	del in 5q → leading to LOH	9	0	0	77	linear	linear	6	1	5	2	2
T-LBL	pediatric	UPN185	dup in 9q	9	0	0	77	linear	linear	7	0	6	3	3
T-LBL	pediatric	UPN185	PTEN (p.Arg233fs_b)	9	0	0	77	linear	linear	7	1	6	2	1
T-LBL	pediatric	UPN185	normal in 7q	10	0	0	66	linear	linear	7	0	6	2	2
T-LBL	pediatric	UPN185	TP53 (p.Gly245Arg)	11	0	0	6	linear	linear	9	0	8	2	1
T-LBL	pediatric	UPN186	del in 1q	1	0	70	80	linear	linear	1	0	0	1	1
T-LBL	pediatric	UPN186	del in 10q	1	0	70	80	linear	linear	1	0	0	1	1
T-LBL	pediatric	UPN186	dup in 1q	2	0	52	80	linear	linear	2	0	1	3	3
T-LBL	pediatric	UPN186	dup in 13q	2	0	52	80	linear	linear	2	0	1	3	3
T-LBL	pediatric	UPN186	PIK3R1 (p.Lys459del)	3	0	17	22	linear	linear	3	0	2	2	1
T-LBL	pediatric	UPN187	LOH in 5q	1	0	61	96	linear	linear	1	0	0	2	2
T-LBL	pediatric	UPN187	dup7q	2	0	60	75	linear	linear	2	0	1	3	3
T-LBL	pediatric	UPN187	dup5p	2	0	60	75	linear	linear	2	0	1	3	3
T-LBL	pediatric	UPN187	dup in 5q	2	0	60	75	linear	linear	2	0	1	3	3
T-LBL	pediatric	UPN187	KMT2D (p.Pro4937fs)	3	0	45	75	linear	linear	3	0	2	2	1

2 time points

T-LBL	adult	UPN124	16	3781374	C	T	1724	3	1729	0.17 %	1786	0	1791	0.00 %	757	30	800	3.75 %
T-LBL	pediatric	UPN185																
T-LBL	pediatric	UPN185																
T-LBL	pediatric	UPN185																
T-LBL	pediatric	UPN185																
T-LBL	pediatric	UPN185																
T-LBL	pediatric	UPN185																
T-LBL	pediatric	UPN185																
T-LBL	pediatric	UPN185																
T-LBL	pediatric	UPN185																
T-LBL	pediatric	UPN185	10	89717672	C	GG	1245	0	1246	0.00 %	161	122	416	29.33 %	153	0	1629	0.00 %
T-LBL	pediatric	UPN185	10	89717672	C	AG	1245	0	1246	0.00 %	161	122	416	29.33 %	153	0	1629	0.00 %
T-LBL	pediatric	UPN185	3	178936082	G	A	1043	2	1048	0.19 %	359	23	382	6.02 %	1398	0	1400	0.00 %
T-LBL	pediatric	UPN185																
T-LBL	pediatric	UPN185																
T-LBL	pediatric	UPN185																
T-LBL	pediatric	UPN185																
T-LBL	pediatric	UPN185																
T-LBL	pediatric	UPN185																
T-LBL	pediatric	UPN185	10	89717672	C	GA	1245	0	1246	0.00 %	161	0	416	0.00 %	153	1447	1629	88.83 %
T-LBL	pediatric	UPN185																
T-LBL	pediatric	UPN185	17	7577548	C	G	1525	0	1529	0.00 %	558	0	560	0.00 %	1778	54	1832	2.95 %
T-LBL	pediatric	UPN186																
T-LBL	pediatric	UPN186																
T-LBL	pediatric	UPN186																
T-LBL	pediatric	UPN186																
T-LBL	pediatric	UPN186	5	67589609	GAAA	G	649	0	650	0.00 %	294	25	295	8.47 %	47	6	53	11.32 %
T-LBL	pediatric	UPN187																
T-LBL	pediatric	UPN187																
T-LBL	pediatric	UPN187																
T-LBL	pediatric	UPN187																
T-LBL	pediatric	UPN187	12	49420940	G	GCCTT	1014	0	1030	0.00 %	194	44	196	22.45 %	1031	392	1039	37.73 %

2 time points

Supplemental Data 3

T-LBL	adult	UPN124														SNV/Indel
T-LBL	pediatric	UPN185	9	0	35897175	2	not detected	92.10 %	91.44 %	92.76 %	1511	94.71 %	94.09 %	95.34 %	1511	CNV
T-LBL	pediatric	UPN185	9	21118459	25914625	0	not detected	92.00 %				92.00 %				CNV
T-LBL	pediatric	UPN185	6	70957822	89983131	1	not detected	84.35 %	83.79 %	84.91 %	553	88.35 %	87.87 %	88.82 %	553	CNV
T-LBL	pediatric	UPN185	5	98916337	124952368	1	not detected	83.34 %	82.87 %	83.81 %	912	87.58 %	87.15 %	88.02 %	912	CNV
T-LBL	pediatric	UPN185	7	0	58050276	1	not detected	73.46 %	73.12 %	73.79 %	2689	87.18 %	86.90 %	87.47 %	2689	CNV
T-LBL	pediatric	UPN185	5	0	136827498	3	not detected	72.00 %	71.10 %	72.90 %	5424	76.72 %	75.02 %	78.42 %	1728	CNV
T-LBL	pediatric	UPN185	7	61070337	159138663	3	not detected	60.08 %	59.29 %	60.87 %	3430	76.48 %	75.42 %	77.55 %	2513	CNV
T-LBL	pediatric	UPN185	9	70907741	141017240	3	not detected	62.06 %	61.14 %	62.99 %	2237	not detected				CNV
T-LBL	pediatric	UPN185	9	131846125	141017240	4	not detected	59.50 %	57.87 %	61.12 %	510	not detected				CNV
T-LBL	pediatric	UPN185														SNV/Indel
T-LBL	pediatric	UPN185														SNV/Indel
T-LBL	pediatric	UPN185														SNV/Indel
T-LBL	pediatric	UPN185	17	46004828	81195210	3	not detected	not detected				75.74 %	74.50 %	76.99 %	1515	CNV
T-LBL	pediatric	UPN185	10	84057054	135534747	2	not detected	not detected				95.49 %	95.39 %	95.59 %	2257	CNV
T-LBL	pediatric	UPN185	10	0	39150257	1	not detected	not detected				87.76 %	87.48 %	88.05 %	1823	CNV
T-LBL	pediatric	UPN185	10	42389768	135534747	3	not detected	not detected				77.62 %	76.38 %	78.87 %	1553	CNV
T-LBL	pediatric	UPN185	5	136827498	180915260	2	not detected	not detected				93.52 %	93.31 %	93.74 %	1952	CNV
T-LBL	pediatric	UPN185	9	139406104	141213431	3	not detected	not detected				73.95 %	66.84 %	81.06 %	56	CNV
T-LBL	pediatric	UPN185														SNV/Indel
T-LBL	pediatric	UPN185	7	142496294	159138663	2	not detected	not detected				66.00 %				CNV
T-LBL	pediatric	UPN185														SNV/Indel
T-LBL	pediatric	UPN186	1	215911808	234840680	1	not detected	67.67 %	66.26 %	69.08 %	802	72.48 %	66.20 %	78.76 %	36	CNV
T-LBL	pediatric	UPN186	10	86480408	91443450	1	not detected	70.54 %	67.00 %	74.08 %	219	80.00 %				CNV
T-LBL	pediatric	UPN186	1	171510590	213685379	3	not detected	51.86 %	49.26 %	54.45 %	1436	95.91 %	87.63 %	104.18 %	44	CNV
T-LBL	pediatric	UPN186	13	78818381	115091079	3	not detected	52.39 %	49.77 %	55.01 %	1674	92.63 %	71.64 %	113.61 %	13	CNV
T-LBL	pediatric	UPN186														SNV/Indel
T-LBL	pediatric	UPN187	5	67067011	180917280	2	not detected	NA				96.00 %	95.70 %	96.30 %	3330	CNV
T-LBL	pediatric	UPN187	7	59900000	159124173	3	not detected	NA				58.03 %	57.15 %	58.91 %	3443	CNV
T-LBL	pediatric	UPN187	5	14782	45860933	3	not detected	NA				75.66 %	74.39 %	76.93 %	1841	CNV
T-LBL	pediatric	UPN187	5	49491374	67067011	3	not detected	NA				78.92 %	76.41 %	81.43 %	523	CNV
T-LBL	pediatric	UPN187														SNV/Indel

2 time points

Supplemental Data 3

T-LBL	pediatric	UPN187	PIK3R1 (p.Glu451_Tyr452delinsGly SerGln)	4	0	25	75	linear	linear	4	0	3	2	1
T-LBL	pediatric	UPN187	del7p	5	0	24	58	linear	linear	5	0	4	1	1
T-LBL	pediatric	UPN187	NOTCH1 (p.Met1855Leu)	6	0	8	0	linear	linear	5	1	4	2	1
T-LBL	pediatric	UPN187	dup18	7	0	0	47	linear	linear	6	1	5	3	3
T-LBL	pediatric	UPN187	NOTCH1 (p.Pro1828Ala)	8	0	0	16	linear	linear	7	0	6	2	1
T-LBL	pediatric	UPN188	LOH in 9p	1	0	93	95	linear	linear	1	0	0	2	2
T-LBL	pediatric	UPN188	FBXW7 (p.Arg441Gln)	2	0	90	81	linear	linear	2	0	1	2	1
T-LBL	pediatric	UPN188	DDX3X (p.Glu566fs)	3	0	75	81	linear	linear	3	0	2	1	0
T-LBL	pediatric	UPN188	CNOT3 (p.Pro244fs)	4	0	60	62	linear	linear	4	0	3	2	1
T-LBL	pediatric	UPN188	FBXW7 (p.His420Tyr)	5	0	42	62	linear	linear	5	0	4	2	0
T-LBL	pediatric	UPN188	NOTCH1 (p.Leu1585Pro)	6	0	40	44	linear	linear	6	0	5	2	1
T-LBL	pediatric	UPN188	USP7 (c.611+1_611+2insGACCAC TGGACCG)	6	0	40	44	linear	linear	6	0	5	2	1
T-LBL	pediatric	UPN188	FBXW7 (p.Arg278*)	7	0	38	23	linear	linear	7	0	6	2	1
T-LBL	pediatric	UPN188	PIK3CD (p.Cys381Arg)	8	0	22	14	linear	linear	8	0	7	2	0.5
T-LBL	pediatric	UPN188	PIK3CD (p.Glu1045Lys)	9	0	6	13	linear	linear	9	0	8	2	0.5
T-LBL	pediatric	UPN188	MYB (p.Gln424His)	10	0	0	5	linear	linear	10	0	9	2	1
T-LBL	pediatric	UPN189	LOH in 9p	1	0	86	100	linear	linear	1	0	0	2	2
T-LBL	pediatric	UPN189	USP7 (p.Val214fs)	2	0	54	92	linear	linear	2	0	1	2	1
T-LBL	pediatric	UPN189	NOTCH1 (p.Gln1614_Phe1617delins ArgHisIle)	3	0	11	0	linear	linear	3	1	2	2	1
T-LBL	pediatric	UPN189	IL7R (p.Lys214delinsValMetAsn PheThr)	3	0	11	0	linear	linear	3	1	2	2	1
T-LBL	pediatric	UPN189	FBXW7 (p.Arg505Cys)	4	0	2	0	linear	linear	4	0	3	2	1
T-LBL	pediatric	UPN189	NRAS (p.Gly12Asp)	5	0	0	92	linear	linear	3	1	2	2	1
T-LBL	pediatric	UPN189	SMARCA4 (p.Gly883Asp)	5	0	0	92	linear	linear	3	1	2	2	1
T-LBL	pediatric	UPN189	del in 17p	6	0	0	70	linear	linear	4	1	3	1	1
T-LBL	pediatric	UPN189	TP53 (p.Gly245Ser)	6	0	0	70	linear	linear	4	0	3	2	0
T-LBL	pediatric	UPN189	dup in 17p	7	0	0	55	linear	linear	5	0	4	3	3

T-LBL	pediatric	UPN187	5	67589589	AATAT	GTTCCCAA	748	4	749	0.53 %	289	41	329	12.46 %	666	398	1071	37.16 %
T-LBL	pediatric	UPN187																
T-LBL	pediatric	UPN187	9	139396275	T	G	1443	11	1457	0.75 %	485	21	507	4.14 %	1356	11	1374	0.80 %
T-LBL	pediatric	UPN187																
T-LBL	pediatric	UPN187	9	139396356	G	C	1210	0	1211	0.00 %	371	0	373	0.00 %	1086	96	1182	8.12 %
T-LBL	pediatric	UPN188																
T-LBL	pediatric	UPN188	4	153249456	C	T	1340	0	1341	0.00 %	687	577	1265	45.61 %	490	320	811	39.46 %
T-LBL	pediatric	UPN188	X	41206193	AGA	CGAGCCC ACG	578	0	578	0.00 %	67	238	320	74.38 %	50	259	321	80.69 %
T-LBL	pediatric	UPN188	19	54649666	T	TC	1734	13	1743	0.75 %	1187	361	1202	30.03 %	754	223	759	29.38 %
T-LBL	pediatric	UPN188	4	153249520	G	A	1182	0	1182	0.00 %	874	194	1069	18.15 %	482	228	710	32.11 %
T-LBL	pediatric	UPN188	9	139399389	A	G	1593	1	1596	0.06 %	1171	343	1518	22.60 %	630	160	791	20.23 %
T-LBL	pediatric	UPN188	16	9014214	A	ACGGTCC AGTGGTC	936	0	938	0.00 %	721	158	731	21.61 %	498	116	501	23.15 %
T-LBL	pediatric	UPN188	4	153258983	G	A	907	0	907	0.00 %	631	148	781	18.95 %	619	79	699	11.30 %
T-LBL	pediatric	UPN188	1	9779982	T	C	1305	0	1306	0.00 %	794	100	895	11.17 %	483	37	525	7.05 %
T-LBL	pediatric	UPN188	1	9787030	G	A	1409	0	1412	0.00 %	873	28	904	3.10 %	490	35	526	6.65 %
T-LBL	pediatric	UPN188	6	135518167	A	T	1320	2	1323	0.15 %	1223	5	1228	0.41 %	999	24	1023	2.35 %
T-LBL	pediatric	UPN189																
T-LBL	pediatric	UPN189	16	9012969	G	TTA	1556	0	1581	0.00 %	1388	518	1914	27.06 %	679	372	1057	35.19 %
T-LBL	pediatric	UPN189	9	139399292	GAAGATC ATCT	GATGTGA C	1737	15	1748	0.86 %	2253	138	2277	6.06 %	1588	0	1594	0.00 %
T-LBL	pediatric	UPN189	5	35873684	AAA	GTGATGA ACTTCACC	1154	7	1156	0.61 %	1533	88	1623	5.42 %	992	0	998	0.00 %
T-LBL	pediatric	UPN189	4	153247289	G	A	1523	0	1527	0.00 %	2293	26	2321	1.12 %	1017	0	1019	0.00 %
T-LBL	pediatric	UPN189	1	115258747	C	T	1600	19	1621	1.17 %	2480	1	2486	0.04 %	716	616	1336	46.11 %
T-LBL	pediatric	UPN189	19	11132432	G	A	1902	46	1952	2.36 %	2529	1	2537	0.04 %	774	775	1551	49.97 %
T-LBL	pediatric	UPN189																
T-LBL	pediatric	UPN189	17	7577548	C	T	1746	5	1754	0.29 %	2314	0	2317	0.00 %	490	587	1084	54.15 %
T-LBL	pediatric	UPN189																

2 time points

T-LBL	pediatric	UPN187														SNV/Indel
T-LBL	pediatric	UPN187	7	0	59900000	1	not detected	NA				58.89 %	58.44 %	59.34 %	2694	CNV
T-LBL	pediatric	UPN187														SNV/Indel
T-LBL	pediatric	UPN187	18	0	78122432	3	not detected	NA				47.52 %	46.74 %	48.30 %	3333	CNV
T-LBL	pediatric	UPN187														SNV/Indel
T-LBL	pediatric	UPN188	9	0	38919501	2	not detected	86.96 %	86.02 %	87.91 %	1752	present				CNV
T-LBL	pediatric	UPN188														SNV/Indel
T-LBL	pediatric	UPN188														SNV/Indel
T-LBL	pediatric	UPN188														SNV/Indel
T-LBL	pediatric	UPN188														SNV/Indel
T-LBL	pediatric	UPN188														SNV/Indel
T-LBL	pediatric	UPN188														SNV/Indel
T-LBL	pediatric	UPN188														SNV/Indel
T-LBL	pediatric	UPN188														SNV/Indel
T-LBL	pediatric	UPN188														SNV/Indel
T-LBL	pediatric	UPN188														SNV/Indel
T-LBL	pediatric	UPN189	9	0	36073497	2	not detected	86.00 %	85.00 %	86.00 %	1677	99.60 %	99.40 %	99.80 %	1677	CNV
T-LBL	pediatric	UPN189														SNV/Indel
T-LBL	pediatric	UPN189														SNV/Indel
T-LBL	pediatric	UPN189														SNV/Indel
T-LBL	pediatric	UPN189														SNV/Indel
T-LBL	pediatric	UPN189														SNV/Indel
T-LBL	pediatric	UPN189														SNV/Indel
T-LBL	pediatric	UPN189	17	0	19069543	1	not detected	not detected				66.47 %	65.84 %	67.10 %	1058	CNV
T-LBL	pediatric	UPN189														SNV/Indel
T-LBL	pediatric	UPN189	17	19069543	21416601	3	not detected	not detected				59.74 %	53.36 %	66.12 %	52	CNV

T-LBL	pediatric	UPN189	del4	7	0	0	55	linear	linear	5	0	4	1	1
T-LBL	pediatric	UPN189	NT5C2 (p.Arg367Gln)	8	0	0	24	linear	linear	6	0	5	2	1
T-LBL	pediatric	UPN191	dup in 1q	1	0	90	90	branching (2)	linear	1	0	0	3	3
T-LBL	pediatric	UPN191	del7p	1	0	90	90	branching (2)	linear	1	0	0	1	1
T-LBL	pediatric	UPN191	dup7q	1	0	90	90	branching (2)	linear	1	0	0	3	3
T-LBL	pediatric	UPN191	MYCBP2 (p.Arg2388Cys)	2	0	47	0	branching (2)	linear	2	1	1	2	1
T-LBL	pediatric	UPN191	PTEN (p.Met1?)	3	0	26	0	branching (2)	linear	3	0	2	2	1
T-LBL	pediatric	UPN191	KIT (p.Glu925*)	4	0	17	0	branching (2)	linear	4	0	3	2	1
T-LBL	pediatric	UPN191	PIK3CD (p.Arg475His)	4	0	17	0	branching (2)	linear	4	0	3	2	1
T-LBL	pediatric	UPN191	USH2A (p.Asn2710Lys)	4	0	17	0	branching (2)	linear	4	0	3	2	1
T-LBL	pediatric	UPN191	PIK3CD (p.Gly124Asp)	5	0	33	90	branching (2)	linear	2	1	1	2	1
T-LBL	pediatric	UPN191	JAK1 (p.Asp684Asn)	6	0	0	90	branching (2)	linear	3	0	5	2	1
T-LBL	pediatric	UPN191	KMT2C (p.Leu104Arg)	7	0	0	19	branching (2)	linear	4	0	6	2	0
T-LBL	pediatric	UPN191	USH2A (p.Thr3635Asn)	8	0	0	10	branching (2)	linear	5	0	7	2	1
T-LBL	pediatric	UPN191	EZH2 (p.Asp184Asn)	8	0	0	10	branching (2)	linear	5	0	7	2	0
T-LBL	pediatric	UPN192	del in 1q	1	0	70	90	linear	branching (2)	1	0	0	1	1
T-LBL	pediatric	UPN192	del in 11p	2	0	60	90	linear	branching (2)	2	0	1	1	1
T-LBL	pediatric	UPN192	LOH in 17q	2	0	60	90	linear	branching (2)	2	0	1	2	2
T-LBL	pediatric	UPN192	STAT5B (p.Gly698Val)	2	0	60	90	linear	branching (2)	2	0	1	2	0
T-LBL	pediatric	UPN192	STAT5B (p.Asn642His)	2	0	60	90	linear	branching (2)	2	0	1	2	0
T-LBL	pediatric	UPN192	LOH in 17q	3	0	54	70	linear	branching (2)	3	0	2	2	2
T-LBL	pediatric	UPN192	NT5C2 (p.Arg367Gln)	4	0	0	37	linear	branching (2)	4	1	3	2	1
T-LBL	pediatric	UPN192	NOTCH1 (p.Gln2395*)	4	0	0	37	linear	branching (2)	4	1	3	2	1
T-LBL	pediatric	UPN192	JAK1 (p.Asn84Ser)	4	0	0	37	linear	branching (2)	4	1	3	2	1
T-LBL	pediatric	UPN192	NOTCH1 (p.Ala1888Thr)	5	0	0	13	linear	branching (2)	5	0	4	2	1
T-LBL	pediatric	UPN192	NOTCH1 (p.Gly785Ser)	6	0	0	6	linear	branching (2)	6	0	5	2	1
T-LBL	pediatric	UPN192	FBXW7 (p.Tyr519Cys)	6	0	0	6	linear	branching (2)	6	0	5	2	1
T-LBL	pediatric	UPN192	ZBTB7A (p.Cys384Tyr)	6	0	0	6	linear	branching (2)	6	0	5	2	1
T-LBL	pediatric	UPN192	KMT2C (p.Thr820Ile)	7	0	3	1	linear	branching (2)	4	1	3	2	1
T-LBL	pediatric	UPN193	del4	1	0	98	99	linear	linear	1	0	0	1	1
T-LBL	pediatric	UPN193	LOH9	1	0	98	99	linear	linear	1	0	0	2	2
T-LBL	pediatric	UPN193	dup in 16p	2	0	90	95	linear	linear	2	0	1	3	3
T-LBL	pediatric	UPN193	dup in 16p	2	0	90	95	linear	linear	2	0	1	3	3
T-LBL	pediatric	UPN193	dup in 16q	2	0	90	95	linear	linear	2	0	1	3	3
T-LBL	pediatric	UPN193	dup in 16p	3	0	86	95	linear	linear	3	0	2	3	3

T-LBL	pediatric	UPN189																
T-LBL	pediatric	UPN189	10	104852955	C	T	1278	23	1302	1.77 %	1871	1	1879	0.05 %	1075	157	1234	12.72 %
T-LBL	pediatric	UPN191																
T-LBL	pediatric	UPN191																
T-LBL	pediatric	UPN191																
T-LBL	pediatric	UPN191	13	77720322	G	A	1118	2	1123	0.18 %	99	30	129	23.26 %	496	0	498	0.00 %
T-LBL	pediatric	UPN191	10	89624227	A	C	1387	0	1391	0.00 %	326	50	376	13.30 %	87	0	87	0.00 %
T-LBL	pediatric	UPN191	4	55603417	G	T	1407	2	1410	0.14 %	202	20	222	9.01 %	594	1	597	0.17 %
T-LBL	pediatric	UPN191	1	9780550	G	A	1388	0	1388	0.00 %	277	26	304	8.55 %	1588	1	1590	0.06 %
T-LBL	pediatric	UPN191	1	216061861	G	T	1537	0	1538	0.00 %	239	20	259	7.72 %	554	0	554	0.00 %
T-LBL	pediatric	UPN191	1	9775907	G	A	1361	0	1366	0.00 %	361	71	433	16.40 %	892	679	1573	43.17 %
T-LBL	pediatric	UPN191	1	65311261	C	T	1425	0	1428	0.00 %	337	0	338	0.00 %	315	370	686	53.94 %
T-LBL	pediatric	UPN191	7	152027764	A	C	1276	0	1277	0.00 %	159	0	160	0.00 %	552	38	591	6.43 %
T-LBL	pediatric	UPN191	1	215953220	G	T	1517	1	1521	0.07 %	262	0	262	0.00 %	574	30	604	4.97 %
T-LBL	pediatric	UPN191	7	148525907	C	T	1111	2	1115	0.18 %	117	0	117	0.00 %	826	30	861	3.48 %
T-LBL	pediatric	UPN192																
T-LBL	pediatric	UPN192																
T-LBL	pediatric	UPN192																
T-LBL	pediatric	UPN192	17	40354811	C	A	2081	4	2091	0.19 %	341	597	942	63.38 %	199	1515	1716	88.29 %
T-LBL	pediatric	UPN192	17	40359729	T	G	1161	1	1162	0.09 %	196	287	483	59.42 %	112	860	973	88.39 %
T-LBL	pediatric	UPN192																
T-LBL	pediatric	UPN192	10	104852955	C	T	1451	0	1454	0.00 %	747	1	749	0.13 %	1059	234	1293	18.10 %
T-LBL	pediatric	UPN192	9	139391008	G	A	2341	0	2348	0.00 %	1279	0	1288	0.00 %	1717	399	2118	18.84 %
T-LBL	pediatric	UPN192	1	65344786	T	C	1441	0	1444	0.00 %	906	0	910	0.00 %	1196	270	1467	18.40 %
T-LBL	pediatric	UPN192	9	139395276	C	T	1641	2	1646	0.12 %	785	0	785	0.00 %	1276	94	1375	6.84 %
T-LBL	pediatric	UPN192	9	139407844	C	T	1660	0	1664	0.00 %	728	2	731	0.27 %	1305	37	1345	2.75 %
T-LBL	pediatric	UPN192	4	153247246	T	C	1639	0	1644	0.00 %	1245	0	1251	0.00 %	1587	56	1644	3.41 %
T-LBL	pediatric	UPN192	19	4054080	C	T	1983	1	1990	0.05 %	1213	0	1215	0.00 %	1676	42	1722	2.44 %
T-LBL	pediatric	UPN192	7	151945060	G	A	4639	26	4678	0.56 %	2326	42	2372	1.77 %	4265	26	4295	0.61 %
T-LBL	pediatric	UPN193																
T-LBL	pediatric	UPN193																
T-LBL	pediatric	UPN193																
T-LBL	pediatric	UPN193																
T-LBL	pediatric	UPN193																
T-LBL	pediatric	UPN193																

T-LBL	pediatric	UPN189	4	0	191154276	1	not detected	not detected				55.00 %	55.00 %	55.00 %	6733	CNV
T-LBL	pediatric	UPN189														SNV/Indel
T-LBL	pediatric	UPN191	1	114973429	213068595	3	not detected	NA				77.68 %	69.66 %	85.71 %	116	CNV
T-LBL	pediatric	UPN191	7	0	59900000	1	not detected	NA				89.48 %	87.77 %	91.18 %	65	CNV
T-LBL	pediatric	UPN191	7	59900000	159124173	3	not detected	NA				78.41 %	70.56 %	86.27 %	108	CNV
T-LBL	pediatric	UPN191														SNV/Indel
T-LBL	pediatric	UPN191														SNV/Indel
T-LBL	pediatric	UPN191														SNV/Indel
T-LBL	pediatric	UPN191														SNV/Indel
T-LBL	pediatric	UPN191														SNV/Indel
T-LBL	pediatric	UPN191														SNV/Indel
T-LBL	pediatric	UPN191														SNV/Indel
T-LBL	pediatric	UPN191														SNV/Indel
T-LBL	pediatric	UPN191														SNV/Indel
T-LBL	pediatric	UPN191														SNV/Indel
T-LBL	pediatric	UPN192	1	47698310	47881595	1	not detected	71.12 %	61.12 %	81.11 %	11	89.43 %	87.19 %	91.67 %	11	CNV
T-LBL	pediatric	UPN192	11	33934855	36528431	1	not detected	55.09 %	51.97 %	58.20 %	153	88.63 %	88.16 %	89.10 %	153	CNV
T-LBL	pediatric	UPN192	17	37277386	81195210	2	not detected	61.52 %	60.63 %	62.42 %	1904	93.51 %	93.33 %	93.68 %	1904	CNV
T-LBL	pediatric	UPN192														SNV/Indel
T-LBL	pediatric	UPN192														SNV/Indel
T-LBL	pediatric	UPN192	17	28044101	36049552	2	not detected	51.70 %	49.00 %	54.40 %	309	70.04 %	69.27 %	70.81 %	309	CNV
T-LBL	pediatric	UPN192														SNV/Indel
T-LBL	pediatric	UPN192														SNV/Indel
T-LBL	pediatric	UPN192														SNV/Indel
T-LBL	pediatric	UPN192														SNV/Indel
T-LBL	pediatric	UPN192														SNV/Indel
T-LBL	pediatric	UPN192														SNV/Indel
T-LBL	pediatric	UPN192														SNV/Indel
T-LBL	pediatric	UPN192														SNV/Indel
T-LBL	pediatric	UPN193	4	62715443	63773346	1	not detected	86.01 %	77.34 %	94.68 %	42	85.59 %	75.44 %	95.74 %	42	CNV
T-LBL	pediatric	UPN193	9	0	141213431	2	not detected	98.37 %	98.24 %	98.51 %	4725	99.28 %	99.15 %	99.41 %	4725	CNV
T-LBL	pediatric	UPN193	16	0	3779195	3	not detected	90.76 %	84.79 %	96.73 %	171	90.76 %	85.00 %	96.52 %	171	CNV
T-LBL	pediatric	UPN193	16	26297708	35257261	3	not detected	88.95 %	84.04 %	93.87 %	228	88.89 %	84.18 %	93.59 %	228	CNV
T-LBL	pediatric	UPN193	16	54498748	67510779	3	not detected	92.35 %	89.46 %	95.24 %	488	90.83 %	88.11 %	93.55 %	488	CNV
T-LBL	pediatric	UPN193	16	18895142	25050906	3	not detected	86.02 %	82.19 %	89.84 %	198	83.34 %	79.98 %	86.70 %	198	CNV

T-LBL	pediatric	UPN193	dup20	3	0	86	95	linear	linear	3	0	2	3	3
T-LBL	pediatric	UPN193	dup in 7q	4	0	47	95	linear	linear	4	0	3	3	3
T-LBL	pediatric	UPN193	TP53 (p.Arg175His)	5	0	0	95	linear	linear	5	4	4	2	1
T-LBL	pediatric	UPN193	SETD1B (p.SerProValLeuLeuGluThr1464PheProValLeuLeuGluSer)	5	0	0	95	linear	linear	5	6	4	2	1
T-LBL	pediatric	UPN193	CREBBP (p.Gly1028*)	5	0	0	95	linear	linear	5	7	4	2	1
T-LBL	pediatric	UPN193	USH2A (p.Asp1597Val)	5	0	0	95	linear	linear	5	8	4	2	1
T-LBL	pediatric	UPN193	PIK3CA (p.Glu545Lys)	5	0	0	95	linear	linear	5	9	4	2	1
T-LBL	pediatric	UPN193	KMT2D (p.Arg5086*)	6	0	0	89	linear	linear	6	2	5	2	1
T-LBL	pediatric	UPN193	ZBTB7A (p.Gly3Asp)	6	0	0	89	linear	linear	6	10	5	2	1
T-LBL	pediatric	UPN193	CCND3 (p.Leu137*)	6	0	0	89	linear	linear	6	11	5	2	1
T-LBL	pediatric	UPN193	MED12 (p.Gln2069_Gln2070del)	7	0	0	62	linear	linear	7	5	6	2	1
T-LBL	pediatric	UPN193	PTPRD (p.Gly1819*)	8	0	0	16	linear	linear	8	0	7	2	1
T-LBL	pediatric	UPN193	MYB (p.Tyr261Cys)	9	0	0	7	linear	linear	9	1	8	2	1
T-LBL	pediatric	UPN193	CCND3 (p.Leu188Ile)	9	0	0	7	linear	linear	9	3	8	2	0.5
T-LBL	pediatric	UPN195	del in 9p	1	0	99	95	branching (2)	branching (2)	1	0	0	1	1
T-LBL	pediatric	UPN195	LOH in 7q	2	0	97	92	branching (2)	branching (2)	2	0	1	2	2
T-LBL	pediatric	UPN195	dup20	3	0	80	75	branching (2)	branching (2)	3	0	2	3	3
T-LBL	pediatric	UPN195	del in 14q	4	0	63	75	branching (2)	branching (2)	4	0	3	1	1
T-LBL	pediatric	UPN195	PIK3CA (p.Glu542Lys)	5	0	20	8	branching (2)	branching (2)	5	1	4	2	1
T-LBL	pediatric	UPN195	PIK3CA (p.Cys420Arg)	6	0	22	1	branching (2)	branching (2)	6	0	5	2	0.5
T-LBL	pediatric	UPN195	PIK3CA (p.Glu545Lys)	7	0	4	1	branching (2)	branching (2)	7	0	6	2	0
T-LBL	pediatric	UPN195	PIK3R1 (p.Asn564Asp)	8	0	21	54	branching (2)	branching (2)	5	1	4	2	1
T-LBL	pediatric	UPN196	LOH in 9p	1	0	92	95	branching (2)	linear	1	0	0	2	2
T-LBL	pediatric	UPN196	del in 11q	2	0	85	92	branching (2)	linear	2	0	1	1	1
T-LBL	pediatric	UPN196	FBXW7 (p.Arg465His)	3	0	75	82	branching (2)	linear	3	0	2	2	1
T-LBL	pediatric	UPN196	PIK3CA (p.Glu542Lys)	4	0	50	0	branching (2)	linear	4	1	3	2	1
T-LBL	pediatric	UPN196	del in 6q	5	0	25	72	branching (2)	linear	4	1	3	1	1
T-LBL	pediatric	UPN196	PIK3R1 (p.Asn564Asp)	6	0	15	5	branching (2)	linear	5	0	5	2	1
T-LBL	pediatric	UPN197	del in 7p	1	0	97	100	linear	linear	1	0	0	1	1
T-LBL	pediatric	UPN197	LOH in 17q	1	0	97	100	linear	linear	1	0	0	2	2
T-LBL	pediatric	UPN197	STAT5B (p.Val712Glu)	1	0	97	100	linear	linear	1	0	0	2	1

2 time points

T-LBL	pediatric	UPN193																
T-LBL	pediatric	UPN193																
T-LBL	pediatric	UPN193	17	7578406	C	T	1360	0	1363	0.00 %	2540	1	2546	0.04 %	813	971	1787	54.34 %
T-LBL	pediatric	UPN193	12	122260876	CCCCGGTGCTCCTGGAGC	TCCCGGTGCTCCTGGAGT	1	0	1122	0.00 %	1973	0	1977	0.00 %	750	817	1573	51.94 %
T-LBL	pediatric	UPN193	16	3817889	C	A	1080	0	1081	0.00 %	1995	0	1995	0.00 %	647	592	1242	47.67 %
T-LBL	pediatric	UPN193	1	216262450	T	A	949	0	954	0.00 %	1726	0	1733	0.00 %	612	555	1170	47.44 %
T-LBL	pediatric	UPN193	3	178936091	G	A	1075	0	1080	0.00 %	1954	0	1956	0.00 %	635	576	1216	47.37 %
T-LBL	pediatric	UPN193	12	49420493	G	A	1631	0	1631	0.00 %	2992	2	3003	0.07 %	1045	905	1955	46.29 %
T-LBL	pediatric	UPN193	19	4055223	C	T	1293	0	1294	0.00 %	2345	0	2351	0.00 %	860	708	1572	45.04 %
T-LBL	pediatric	UPN193	6	41908112	A	T	1108	1	1112	0.09 %	1848	0	1850	0.00 %	685	533	1220	43.69 %
T-LBL	pediatric	UPN193	X	70360623	ACAGCAG	A	580	0	585	0.00 %	1094	0	1103	0.00 %	301	485	788	61.55 %
T-LBL	pediatric	UPN193	9	8331661	C	A	1107	0	1108	0.00 %	1642	2	1646	0.12 %	1241	109	1353	8.06 %
T-LBL	pediatric	UPN193	6	135514995	A	G	1155	0	1161	0.00 %	2218	2	2225	0.09 %	1282	44	1327	3.32 %
T-LBL	pediatric	UPN193	6	41904985	G	T	1065	2	1067	0.19 %	2033	1	2035	0.05 %	1274	46	1322	3.48 %
T-LBL	pediatric	UPN195																
T-LBL	pediatric	UPN195																
T-LBL	pediatric	UPN195																
T-LBL	pediatric	UPN195																
T-LBL	pediatric	UPN195	3	178936082	G	A	1057	0	1058	0.00 %	1481	227	1712	13.26 %	1657	69	1729	3.99 %
T-LBL	pediatric	UPN195	3	178927980	T	C	726	0	728	0.00 %	1049	132	1183	11.16 %	1077	4	1083	0.37 %
T-LBL	pediatric	UPN195	3	178936091	G	A	1033	1	1036	0.10 %	1662	37	1699	2.18 %	1687	8	1697	0.47 %
T-LBL	pediatric	UPN195	5	67591097	A	G	1117	0	1119	0.00 %	1435	168	1603	10.48 %	1130	424	1556	27.25 %
T-LBL	pediatric	UPN196																
T-LBL	pediatric	UPN196																
T-LBL	pediatric	UPN196	4	153249384	C	T	1797	0	1801	0.00 %	700	417	1117	37.33 %	598	417	1017	41.00 %
T-LBL	pediatric	UPN196	3	178936082	G	A	1457	0	1458	0.00 %	668	240	911	26.34 %	657	0	658	0.00 %
T-LBL	pediatric	UPN196																
T-LBL	pediatric	UPN196	5	67591097	A	G	1431	1	1434	0.07 %	831	67	900	7.44 %	715	19	735	2.59 %
T-LBL	pediatric	UPN197																
T-LBL	pediatric	UPN197																
T-LBL	pediatric	UPN197	17	40354460	A	T	1278	3	1283	0.23 %	23	977	1003	97.41 %	0	70	70	100.00 %

2 time points

T-LBL	pediatric	UPN193	20	0	63025520	3	not detected	86.94 %	85.92 %	87.95 %	2725	83.35 %	82.46 %	84.23 %	2725	CNV
T-LBL	pediatric	UPN193	7	141768685	159138663	3	not detected	47.38 %	45.80 %	48.97 %	911	79.44 %	77.52 %	81.36 %	911	CNV
T-LBL	pediatric	UPN193														SNV/Indel
T-LBL	pediatric	UPN193														SNV/Indel
T-LBL	pediatric	UPN193														SNV/Indel
T-LBL	pediatric	UPN193														SNV/Indel
T-LBL	pediatric	UPN193														SNV/Indel
T-LBL	pediatric	UPN193														SNV/Indel
T-LBL	pediatric	UPN193														SNV/Indel
T-LBL	pediatric	UPN193														SNV/Indel
T-LBL	pediatric	UPN193														SNV/Indel
T-LBL	pediatric	UPN193														SNV/Indel
T-LBL	pediatric	UPN193														SNV/Indel
T-LBL	pediatric	UPN193														SNV/Indel
T-LBL	pediatric	UPN195	9	0	33168809	1	not detected	97.33 %	96.98 %	97.69 %	1697	95.08 %	94.81 %	95.35 %	1697	CNV
T-LBL	pediatric	UPN195	7	142013744	142447199	2	not detected	88.79 %	79.83 %	97.75 %	21	83.87 %	74.06 %	93.68 %	21	CNV
T-LBL	pediatric	UPN195	20	0	63025520	3	not detected	80.03 %	78.86 %	81.19 %	2556	75.28 %	74.36 %	76.20 %	2556	CNV
T-LBL	pediatric	UPN195	14	54238121	54420853	1	not detected	63.35 %	57.74 %	68.96 %	7	87.43 %	83.44 %	91.43 %	7	CNV
T-LBL	pediatric	UPN195														SNV/Indel
T-LBL	pediatric	UPN195														SNV/Indel
T-LBL	pediatric	UPN195														SNV/Indel
T-LBL	pediatric	UPN195														SNV/Indel
T-LBL	pediatric	UPN196	9	0	34244358	2	not detected	86.22 %	85.51 %	86.94 %	1702	90.29 %	89.72 %	90.87 %	1702	CNV
T-LBL	pediatric	UPN196	11	127156972	132946652	1	not detected	71.95 %	69.63 %	74.28 %	388	79.47 %	77.29 %	81.65 %	388	CNV
T-LBL	pediatric	UPN196														SNV/Indel
T-LBL	pediatric	UPN196														SNV/Indel
T-LBL	pediatric	UPN196	6	70136477	112545288	1	not detected	34.68 %	33.63 %	35.73 %	1320	64.23 %	63.24 %	65.23 %	1320	CNV
T-LBL	pediatric	UPN196														SNV/Indel
T-LBL	pediatric	UPN197	7	38277971	50414177	1	not detected	96.23 %	95.47 %	96.99 %	415	98.85 %	97.79 %	99.92 %	7	CNV
T-LBL	pediatric	UPN197	17	35439904	81195210	2	not detected	98.55 %	98.41 %	98.69 %	1775	99.59 %	99.50 %	99.68 %	96	CNV
T-LBL	pediatric	UPN197														SNV/Indel

T-LBL	pediatric	UPN197	NOTCH1 (p.Phe1606delinsLeuGly)	2	0	80	0	linear	linear	2	0	1	2	0.5
T-LBL	pediatric	UPN197	NOTCH1 (p.Thr2337fs)	2	0	80	0	linear	linear	2	0	1	2	0.5
T-LBL	pediatric	UPN197	IL7R (p.Thr244_Ile245insCysArgMetArgCysGlnIle)	3	0	52	0	linear	linear	3	0	2	2	1
T-LBL	pediatric	UPN203	del in 3q	1	0	93	95	linear	linear	1	0	0	1	1
T-LBL	pediatric	UPN203	del in 3q	1	0	93	95	linear	linear	1	0	0	1	1
T-LBL	pediatric	UPN203	del in 6q	1	0	93	95	linear	linear	1	0	0	1	1
T-LBL	pediatric	UPN203	del in 10q	1	0	93	95	linear	linear	1	0	0	1	1
T-LBL	pediatric	UPN203	PTEN (p.Arg233fs)	1	0	93	95	linear	linear	1	0	0	1	0
T-LBL	pediatric	UPN203	dup20	2	0	79	79	linear	linear	2	0	1	3	3
T-LBL	pediatric	UPN204	del in 8p	1	0	95	70	linear	linear	1	0	0	1	1
T-LBL	pediatric	UPN204	del in 9p	1	0	95	70	linear	linear	1	0	0	1	1
T-LBL	pediatric	UPN204	del in 12q	1	0	95	70	linear	linear	1	0	0	1	1
T-LBL	pediatric	UPN204	NOTCH1 (p.Ser2467fs)	2	0	60	70	linear	linear	2	0	1	2	1
T-LBL	pediatric	UPN204	del in 8q	3	0	55	0	linear	linear	3	1	2	1	1
T-LBL	pediatric	UPN204	del in 8q	4	0	40	0	linear	linear	4	0	3	1	1
T-LBL	pediatric	UPN204	del in 8q	4	0	40	0	linear	linear	4	0	3	1	1
T-LBL	pediatric	UPN204	NOTCH1 (p.Ile1718Thr)	5	0	6	0	linear	linear	5	0	4	2	0.5
T-LBL	pediatric	UPN204	del in 17p	6	0	0	70	linear	linear	3	1	2	1	1
T-LBL	pediatric	UPN204	dup in 8q	6	0	0	70	linear	linear	3	1	2	3	3
T-LBL	pediatric	UPN204	TP53 (p.Tyr234His)	6	0	0	70	linear	linear	3	1	2	1	0
T-LBL	pediatric	UPN204	KRAS (p.Glu37Lys)	6	0	0	70	linear	linear	3	1	2	2	1
T-LBL	pediatric	UPN206	dup19	1	0	63	64	linear	linear	1	0	0	3	3
T-LBL	pediatric	UPN206	dup20	1	0	63	64	linear	linear	1	0	0	3	3
T-LBL	pediatric	UPN206	PIK3CA (p.Gly118Asp)	2	0	19	0	linear	linear	2	1	1	2	1
T-LBL	pediatric	UPN206	CCND3 (p.Gln260*)	2	0	19	0	linear	linear	2	1	1	2	1
T-LBL	pediatric	UPN206	dup17	3	0	0	64	linear	linear	2	1	1	3	3
T-LBL	pediatric	UPN206	dup18	3	0	0	64	linear	linear	2	1	1	3	3
T-LBL	pediatric	UPN206	dup21	3	0	0	64	linear	linear	2	1	1	3	3
T-LBL	pediatric	UPN206	dup in 7q	4	0	0	58	linear	linear	3	0	2	3	3
T-LBL	pediatric	UPN207	ZBTB7A (p.Arg464His)	1	0	78	0	branching (2)	branching (2)	1	2	0	2	1

2 time points

T-LBL	pediatric	UPN197	9	139399325	G	GCCT	2262	0	2267	0.00 %	1756	735	1761	41.74 %	358	0	358	0.00 %
T-LBL	pediatric	UPN197	9	139391183	G	GCTCA	2281	0	2284	0.00 %	1851	706	1854	38.08 %	198	0	198	0.00 %
T-LBL	pediatric	UPN197	5	35874575	C	CATGTCGGATGAGATGCCAGAT	813	0	1619	0.00 %	466	329	1268	25.95 %	348	0	348	0.00 %
T-LBL	pediatric	UPN203																
T-LBL	pediatric	UPN203																
T-LBL	pediatric	UPN203																
T-LBL	pediatric	UPN203																
T-LBL	pediatric	UPN203	10	89717671	AC	CGG	804	6	808	0.74 %	110	627	737	85.07 %	35	163	204	79.90 %
T-LBL	pediatric	UPN203																
T-LBL	pediatric	UPN204																
T-LBL	pediatric	UPN204																
T-LBL	pediatric	UPN204																
T-LBL	pediatric	UPN204	9	139390793	C	CAGGGGGTACGT	136	0	137	0.00 %	663	154	667	23.09 %	191	48	191	25.13 %
T-LBL	pediatric	UPN204																
T-LBL	pediatric	UPN204																
T-LBL	pediatric	UPN204																
T-LBL	pediatric	UPN204	9	139397648	A	G	175	0	176	0.00 %	662	22	684	3.22 %	242	0	242	0.00 %
T-LBL	pediatric	UPN204																
T-LBL	pediatric	UPN204																
T-LBL	pediatric	UPN204	17	7577581	A	G	451	0	458	0.00 %	383	2	385	0.52 %	170	293	463	63.28 %
T-LBL	pediatric	UPN204	12	25398210	C	T	449	0	449	0.00 %	117	0	117	0.00 %	61	44	106	41.51 %
T-LBL	pediatric	UPN206																
T-LBL	pediatric	UPN206																
T-LBL	pediatric	UPN206	3	178917478	G	A	411	0	413	0.00 %	714	37	754	4.91 %	218	0	220	0.00 %
T-LBL	pediatric	UPN206	6	41903779	G	A	638	0	642	0.00 %	802	87	890	9.78 %	1094	0	1097	0.00 %
T-LBL	pediatric	UPN206																
T-LBL	pediatric	UPN206																
T-LBL	pediatric	UPN206																
T-LBL	pediatric	UPN206																
T-LBL	pediatric	UPN207	19	4048114	C	T	552	3	566	0.53 %	684	602	1287	46.78 %	1877	0	1882	0.00 %

T-LBL	pediatric	UPN197														SNV/Indel
T-LBL	pediatric	UPN197														SNV/Indel
T-LBL	pediatric	UPN197														SNV/Indel
T-LBL	pediatric	UPN203	3	137373114	149384373	1	not detected	93.27 %	92.48 %	94.05 %	447	94.53 %	93.82 %	95.23 %	447	CNV
T-LBL	pediatric	UPN203	3	173379185	176906607	1	not detected	82.53 %	77.97 %	87.09 %	147	83.78 %	79.38 %	88.18 %	147	CNV
T-LBL	pediatric	UPN203	6	63474976	94766857	1	not detected	92.91 %	92.48 %	93.33 %	1033	93.72 %	93.17 %	94.27 %	1032	CNV
T-LBL	pediatric	UPN203	10	89588888	91109343	1	not detected	91.57 %	89.03 %	94.11 %	64	94.54 %	92.70 %	96.39 %	64	CNV
T-LBL	pediatric	UPN203														SNV/Indel
T-LBL	pediatric	UPN203	20	0	60223822	3	not detected	78.50 %	77.20 %	80.00 %	2437	78.50 %	77.20 %	80.00 %	2437	CNV
T-LBL	pediatric	UPN204	8	0	36437573	1	not detected	98.14 %	98.08 %	98.21 %	11387	74.52 %	72.17 %	76.87 %	42	CNV
T-LBL	pediatric	UPN204	9	0	27755942	1	not detected	97.95 %	97.85 %	98.05 %	8073	68.93 %	64.28 %	73.57 %	26	CNV
T-LBL	pediatric	UPN204	12	121297815	123002143	1	not detected	98.27 %	97.99 %	98.55 %	471	70.00 %				CNV
T-LBL	pediatric	UPN204														SNV/Indel
T-LBL	pediatric	UPN204	8	128757780	130055525	1	not detected	74.29 %	71.65 %	76.93 %	59	not detected				CNV
T-LBL	pediatric	UPN204	8	49000000	51920563	1	not detected	47.85 %	43.38 %	52.32 %	53	not detected				CNV
T-LBL	pediatric	UPN204	8	59938578	64656906	1	not detected	45.86 %	43.48 %	48.24 %	134	not detected				CNV
T-LBL	pediatric	UPN204														SNV/Indel
T-LBL	pediatric	UPN204	17	0	18051447	1	not detected	not detected				83.24 %	80.57 %	85.90 %	51	CNV
T-LBL	pediatric	UPN204	8	69020496	125061895	3	not detected	not detected				76.06 %	62.79 %	89.33 %	34	CNV
T-LBL	pediatric	UPN204														SNV/Indel
T-LBL	pediatric	UPN204														SNV/Indel
T-LBL	pediatric	UPN206	19	0	59132134	3	not detected	66.00 %	64.00 %	67.00 %	2339	46.43 %	45.02 %	47.85 %	2549	CNV
T-LBL	pediatric	UPN206	20	0	63061994	3	not detected	62.00 %	61.00 %	64.00 %	2586	64.12 %	62.39 %	65.85 %	1633	CNV
T-LBL	pediatric	UPN206														SNV/Indel
T-LBL	pediatric	UPN206														SNV/Indel
T-LBL	pediatric	UPN206	17	0	81195210	3	not detected	not detected				60.94 %	59.74 %	62.14 %	3284	CNV
T-LBL	pediatric	UPN206	18	0	78077248	3	not detected	not detected				64.76 %	63.62 %	65.91 %	3480	CNV
T-LBL	pediatric	UPN206	21	0	48129895	3	not detected	not detected				64.12 %	62.39 %	65.85 %	1633	CNV
T-LBL	pediatric	UPN206	7	87213901	141898023	3	not detected	not detected				58.67 %	57.09 %	60.25 %	1781	CNV
T-LBL	pediatric	UPN207														SNV/Indel

T-LBL	pediatric	UPN207	FBXW7 (p.Arg465Cys)	1	0	78	0	branching (2)	branching (2)	1	2	0	2	1
T-LBL	pediatric	UPN207	NRAS (p.Gly12Cys)	1	0	78	0	branching (2)	branching (2)	1	2	0	2	1
T-LBL	pediatric	UPN207	KMT2D (p.Pro1866Thr)	1	0	78	0	branching (2)	branching (2)	1	2	0	2	1
T-LBL	pediatric	UPN207	EZH2 (p.Leu739Pro)	1	0	78	0	branching (2)	branching (2)	1	2	0	2	1
T-LBL	pediatric	UPN207	STAT3 (c.2257+1G>A)	1	0	78	0	branching (2)	branching (2)	1	2	0	2	1
T-LBL	pediatric	UPN207	SMARCA4 (p.Met113Val)	2	0	22	0	branching (2)	branching (2)	2	0	1	2	1
T-LBL	pediatric	UPN207	BCL11B (p.Gln609*)	2	0	22	0	branching (2)	branching (2)	2	0	1	2	1
T-LBL	pediatric	UPN207	SMARCA4 (p.Gly360fs)	3	0	10	0	branching (2)	branching (2)	3	0	2	2	0.5
T-LBL	pediatric	UPN207	NOTCH1 (p.Leu1593Pro)	4	0	22	30	branching (2)	branching (2)	1	2	0	2	0
T-LBL	pediatric	UPN207	NOTCH1 (p.Leu1585Pro)	4	0	22	30	branching (2)	branching (2)	1	2	0	2	0
T-LBL	pediatric	UPN207	BCL11B (p.Arg654His)	5	0	0	68	branching (2)	branching (2)	1	2	0	2	1
T-LBL	pediatric	UPN207	SMARCA4 (p.Arg13Gln)	5	0	0	68	branching (2)	branching (2)	1	2	0	2	1
T-LBL	pediatric	UPN207	MYCBP2 (p.Met3256Thr)	6	0	0	56	branching (2)	branching (2)	2	0	5	2	1
T-LBL	pediatric	UPN207	CREBBP (p.Ser128Tyr)	6	0	0	56	branching (2)	branching (2)	2	0	5	2	1
T-LBL	pediatric	UPN207	KDM6B (p.Arg1178Trp)	6	0	0	56	branching (2)	branching (2)	2	0	5	2	1
T-LBL	pediatric	UPN207	ZNF91 (p.Met931Ile)	7	0	0	46	branching (2)	branching (2)	3	0	6	2	1
T-LBL	pediatric	UPN207	PHF6 (p.Arg116*)	7	0	0	46	branching (2)	branching (2)	3	0	6	1	0
T-LBL	pediatric	UPN207	BCL11B (p.Asp635Asn)	7	0	0	46	branching (2)	branching (2)	3	0	6	2	0.25
T-LBL	pediatric	UPN207	BCL11B (p.Gly515Ser)	7	0	0	46	branching (2)	branching (2)	3	0	6	2	0.25
T-LBL	pediatric	UPN207	CNOT3 (p.Arg5His)	7	0	0	46	branching (2)	branching (2)	3	0	6	2	1
T-LBL	pediatric	UPN207	CREBBP (p.His2384Asn)	7	0	0	46	branching (2)	branching (2)	3	0	6	2	0.5
T-LBL	pediatric	UPN207	FBXW7 (p.Arg13*)	7	0	0	46	branching (2)	branching (2)	3	0	6	2	1
T-LBL	pediatric	UPN207	JAK3 (p.Gly249Arg)	7	0	0	46	branching (2)	branching (2)	3	0	6	2	1
T-LBL	pediatric	UPN207	KIT (p.Glu720Gly)	7	0	0	46	branching (2)	branching (2)	3	0	6	2	1
T-LBL	pediatric	UPN207	MYCN (p.Ala461Thr)	7	0	0	46	branching (2)	branching (2)	3	0	6	2	1
T-LBL	pediatric	UPN207	NOTCH1 (p.Ile1440Thr)	7	0	0	46	branching (2)	branching (2)	3	0	6	2	1
T-LBL	pediatric	UPN207	NOTCH3 (p.Gly501Ser)	7	0	0	46	branching (2)	branching (2)	3	0	6	2	1
T-LBL	pediatric	UPN207	BCL11B (p.Gly581fs)	8	0	0	35	branching (2)	branching (2)	4	0	7	2	0.0625
T-LBL	pediatric	UPN207	MYCBP2 (p.Gly3643Arg)	8	0	0	35	branching (2)	branching (2)	4	0	7	2	0.5
T-LBL	pediatric	UPN207	KMT2D (p.Arg4478Gln)	8	0	0	35	branching (2)	branching (2)	4	0	7	2	0.5
T-LBL	pediatric	UPN207	DDX3X (p.Arg99His)	8	0	0	35	branching (2)	branching (2)	4	0	7	1	0
T-LBL	pediatric	UPN207	BCL11B (p.Arg247Cys)	8	0	0	35	branching (2)	branching (2)	4	0	7	2	0.0625
T-LBL	pediatric	UPN207	CREBBP (p.Arg768*)	8	0	0	35	branching (2)	branching (2)	4	0	7	2	0.25
T-LBL	pediatric	UPN207	EZH2 (p.Arg690His)	8	0	0	35	branching (2)	branching (2)	4	0	7	2	1
T-LBL	pediatric	UPN207	FBXW7 (p.Arg441Gln)	8	0	0	35	branching (2)	branching (2)	4	0	7	2	0.5

Supplemental Data 3

T-LBL	pediatric	UPN207	4	153249385	G	A	595	0	596	0.00 %	412	358	771	46.43 %	466	0	468	0.00 %
T-LBL	pediatric	UPN207	1	115258748	C	A	563	2	567	0.35 %	219	188	409	45.97 %	413	0	413	0.00 %
T-LBL	pediatric	UPN207	12	49436907	G	T	698	0	698	0.00 %	292	247	539	45.83 %	344	2	346	0.58 %
T-LBL	pediatric	UPN207	7	148504778	A	G	509	1	511	0.20 %	218	160	378	42.33 %	295	0	295	0.00 %
T-LBL	pediatric	UPN207	17	40468806	C	T	458	0	459	0.00 %	338	217	555	39.10 %	323	0	323	0.00 %
T-LBL	pediatric	UPN207	19	11096063	A	G	436	0	439	0.00 %	320	45	365	12.33 %	1478	1	1482	0.07 %
T-LBL	pediatric	UPN207	14	99641348	G	A	134	0	134	0.00 %	580	75	655	11.45 %	438	0	439	0.00 %
T-LBL	pediatric	UPN207	19	11098557	CG	C	421	0	424	0.00 %	948	53	1003	5.28 %	316	0	316	0.00 %
T-LBL	pediatric	UPN207	9	139399365	A	G	593	2	606	0.33 %	1227	305	1533	19.90 %	590	68	660	10.30 %
T-LBL	pediatric	UPN207	9	139399389	A	G	554	5	564	0.89 %	1216	232	1448	16.02 %	476	116	594	19.53 %
T-LBL	pediatric	UPN207	14	99641212	C	T	9	0	9	0.00 %	59	0	60	0.00 %	125	70	195	35.90 %
T-LBL	pediatric	UPN207	19	11094865	G	A	478	2	486	0.41 %	251	0	251	0.00 %	202	96	298	32.21 %
T-LBL	pediatric	UPN207	13	77671522	A	G	567	0	568	0.00 %	619	2	621	0.32 %	360	143	505	28.32 %
T-LBL	pediatric	UPN207	16	3900713	G	T	590	1	592	0.17 %	514	1	515	0.19 %	342	145	487	29.77 %
T-LBL	pediatric	UPN207	17	7753230	C	T	616	1	618	0.16 %	421	0	421	0.00 %	477	184	666	27.63 %
T-LBL	pediatric	UPN207	19	23542988	C	T	587	3	597	0.50 %	401	2	423	0.47 %	173	56	229	24.45 %
T-LBL	pediatric	UPN207	X	133527636	C	T	289	0	290	0.00 %	727	2	730	0.27 %	64	45	109	41.28 %
T-LBL	pediatric	UPN207	14	99641270	C	T	23	0	23	0.00 %	174	0	175	0.00 %	101	28	129	21.71 %
T-LBL	pediatric	UPN207	14	99641630	C	T	130	0	130	0.00 %	559	0	562	0.00 %	181	56	238	23.53 %
T-LBL	pediatric	UPN207	19	54646728	G	A	590	1	594	0.17 %	594	0	594	0.00 %	218	72	292	24.66 %
T-LBL	pediatric	UPN207	16	3777898	G	T	510	2	514	0.39 %	247	0	247	0.00 %	1084	322	1407	22.89 %
T-LBL	pediatric	UPN207	4	153332919	G	A	658	3	663	0.45 %	467	0	469	0.00 %	476	152	628	24.20 %
T-LBL	pediatric	UPN207	19	17953241	C	T	379	0	380	0.00 %	644	0	648	0.00 %	241	84	331	25.38 %
T-LBL	pediatric	UPN207	4	55597511	A	G	626	0	627	0.00 %	594	0	594	0.00 %	292	91	384	23.70 %
T-LBL	pediatric	UPN207	2	16086205	G	A	548	1	551	0.18 %	324	0	324	0.00 %	205	67	273	24.54 %
T-LBL	pediatric	UPN207	9	139400029	A	G	589	6	603	1.00 %	1323	0	1327	0.00 %	667	197	868	22.70 %
T-LBL	pediatric	UPN207	19	15298797	C	T	451	0	455	0.00 %	257	0	259	0.00 %	144	38	182	20.88 %
T-LBL	pediatric	UPN207	14	99641430	GC	G	187	0	187	0.00 %	713	3	716	0.42 %	282	58	344	16.86 %
T-LBL	pediatric	UPN207	13	77657276	C	T	575	1	576	0.17 %	526	2	529	0.38 %	221	44	265	16.60 %
T-LBL	pediatric	UPN207	12	49425055	C	T	715	0	717	0.00 %	594	2	598	0.33 %	1100	225	1326	16.97 %
T-LBL	pediatric	UPN207	X	41201759	G	A	239	0	239	0.00 %	320	1	321	0.31 %	33	20	54	37.04 %
T-LBL	pediatric	UPN207	14	99642434	G	A	306	1	310	0.32 %	1029	1	1030	0.10 %	409	80	489	16.36 %
T-LBL	pediatric	UPN207	16	3823913	G	A	656	0	659	0.00 %	437	0	438	0.00 %	613	136	754	18.04 %
T-LBL	pediatric	UPN207	7	148506443	C	T	569	1	572	0.17 %	596	0	596	0.00 %	224	55	280	19.64 %
T-LBL	pediatric	UPN207	4	153249456	C	T	650	0	652	0.00 %	899	0	901	0.00 %	510	116	630	18.41 %

2 time points

T-LBL	pediatric	UPN207					SNV/Indel
T-LBL	pediatric	UPN207					SNV/Indel
T-LBL	pediatric	UPN207					SNV/Indel
T-LBL	pediatric	UPN207					SNV/Indel
T-LBL	pediatric	UPN207					SNV/Indel
T-LBL	pediatric	UPN207					SNV/Indel
T-LBL	pediatric	UPN207					SNV/Indel
T-LBL	pediatric	UPN207					SNV/Indel
T-LBL	pediatric	UPN207					SNV/Indel
T-LBL	pediatric	UPN207					SNV/Indel
T-LBL	pediatric	UPN207					SNV/Indel
T-LBL	pediatric	UPN207					SNV/Indel
T-LBL	pediatric	UPN207					SNV/Indel
T-LBL	pediatric	UPN207					SNV/Indel
T-LBL	pediatric	UPN207					SNV/Indel
T-LBL	pediatric	UPN207					SNV/Indel
T-LBL	pediatric	UPN207					SNV/Indel
T-LBL	pediatric	UPN207					SNV/Indel
T-LBL	pediatric	UPN207					SNV/Indel
T-LBL	pediatric	UPN207					SNV/Indel
T-LBL	pediatric	UPN207					SNV/Indel
T-LBL	pediatric	UPN207					SNV/Indel
T-LBL	pediatric	UPN207					SNV/Indel
T-LBL	pediatric	UPN207					SNV/Indel
T-LBL	pediatric	UPN207					SNV/Indel
T-LBL	pediatric	UPN207					SNV/Indel
T-LBL	pediatric	UPN207					SNV/Indel
T-LBL	pediatric	UPN207					SNV/Indel
T-LBL	pediatric	UPN207					SNV/Indel
T-LBL	pediatric	UPN207					SNV/Indel
T-LBL	pediatric	UPN207					SNV/Indel
T-LBL	pediatric	UPN207					SNV/Indel
T-LBL	pediatric	UPN207					SNV/Indel
T-LBL	pediatric	UPN207					SNV/Indel
T-LBL	pediatric	UPN207					SNV/Indel

T-LBL	pediatric	UPN207	KMT2D (p.Ala198Thr)	8	0	0	35	branching (2)	branching (2)	4	0	7	2	0.5
T-LBL	pediatric	UPN207	PIK3CA (p.Met922Ile)	8	0	0	35	branching (2)	branching (2)	4	0	7	2	1
T-LBL	pediatric	UPN207	SETD1B (p.Ala335Thr)	8	0	0	35	branching (2)	branching (2)	4	0	7	2	1
T-LBL	pediatric	UPN207	USH2A (p.Ala4611Val)	8	0	0	35	branching (2)	branching (2)	4	0	7	2	0.5
T-LBL	pediatric	UPN207	USH2A (p.Arg3205His)	8	0	0	35	branching (2)	branching (2)	4	0	7	2	0.5
T-LBL	pediatric	UPN207	USP7 (c.2047+1G>A)	8	0	0	35	branching (2)	branching (2)	4	0	7	2	1
T-LBL	pediatric	UPN207	ZBTB7A (p.Ala175Val)	8	0	0	35	branching (2)	branching (2)	4	0	7	2	0.5
T-LBL	pediatric	UPN207	ZBTB7A (p.Leu353Pro)	8	0	0	35	branching (2)	branching (2)	4	0	7	2	0.5
T-LBL	pediatric	UPN207	MYCN (p.Pro45fs)	9	0	0	26	branching (2)	branching (2)	5	0	8	2	0.5
T-LBL	pediatric	UPN207	ZBTB7A (p.Gly506Arg)	9	0	0	26	branching (2)	branching (2)	5	0	8	2	0.0625
T-LBL	pediatric	UPN207	DNM2 (p.Arg369Gln)	9	0	0	26	branching (2)	branching (2)	5	0	8	2	1
T-LBL	pediatric	UPN207	TET2 (p.Arg144His)	9	0	0	26	branching (2)	branching (2)	5	0	8	2	1
T-LBL	pediatric	UPN207	PTPRD (p.Arg377His)	9	0	0	26	branching (2)	branching (2)	5	0	8	2	1
T-LBL	pediatric	UPN207	NT5C2 (p.Arg29Gln)	9	0	0	26	branching (2)	branching (2)	5	0	8	2	1
T-LBL	pediatric	UPN207	BCL11B (p.Gly569Ser)	9	0	0	26	branching (2)	branching (2)	5	0	8	2	0
T-LBL	pediatric	UPN207	CREBBP (p.Pro2094Leu)	9	0	0	26	branching (2)	branching (2)	5	0	8	2	0.125
T-LBL	pediatric	UPN207	EZH2 (p.Ile713Val)	9	0	0	26	branching (2)	branching (2)	5	0	8	2	0.5
T-LBL	pediatric	UPN207	FBXW7 (p.Arg505Cys)	9	0	0	26	branching (2)	branching (2)	5	0	8	2	0.25
T-LBL	pediatric	UPN207	KMT2C (p.Arg4931Gln)	9	0	0	26	branching (2)	branching (2)	5	0	8	2	0.5
T-LBL	pediatric	UPN207	KMT2C (p.Ser4338Asn)	9	0	0	26	branching (2)	branching (2)	5	0	8	2	0.5
T-LBL	pediatric	UPN207	KMT2D (p.Arg2836His)	9	0	0	26	branching (2)	branching (2)	5	0	8	2	0.125
T-LBL	pediatric	UPN207	KMT2D (p.Pro763Leu)	9	0	0	26	branching (2)	branching (2)	5	0	8	2	0.125
T-LBL	pediatric	UPN207	NOS3 (p.Ala705Ala)	9	0	0	26	branching (2)	branching (2)	5	0	8	2	1
T-LBL	pediatric	UPN207	NOTCH3 (p.Arg2237Gln)	9	0	0	26	branching (2)	branching (2)	5	0	8	2	0.25
T-LBL	pediatric	UPN207	NOTCH3 (p.Gly382Ser)	9	0	0	26	branching (2)	branching (2)	5	0	8	2	0.25
T-LBL	pediatric	UPN207	TMED1 (p.Val207Ala)	9	0	0	26	branching (2)	branching (2)	5	0	8	2	1
T-LBL	pediatric	UPN207	USP7 (p.Arg793His)	9	0	0	26	branching (2)	branching (2)	5	0	8	2	0.5
T-LBL	pediatric	UPN207	ZBTB7A (p.Asp19Gly)	9	0	0	26	branching (2)	branching (2)	5	0	8	2	0.0625
T-LBL	pediatric	UPN207	ZBTB7A (p.Gly406Ser)	9	0	0	26	branching (2)	branching (2)	5	0	8	2	0.0625
T-LBL	pediatric	UPN207	USH2A (p.Ala120Thr)	10	0	0	16	branching (2)	branching (2)	6	0	9	2	0
T-LBL	pediatric	UPN207	TET2 (p.Arg1593Gln)	10	0	0	16	branching (2)	branching (2)	6	0	9	2	0.5
T-LBL	pediatric	UPN207	ROR2 (p.Arg312Cys)	10	0	0	16	branching (2)	branching (2)	6	0	9	2	1
T-LBL	pediatric	UPN207	DDX3X (p.Arg488His)	10	0	0	16	branching (2)	branching (2)	6	0	9	1	0
T-LBL	pediatric	UPN207	CREBBP (p.Gly1814Arg)	10	0	0	16	branching (2)	branching (2)	6	0	9	2	0.0322
T-LBL	pediatric	UPN207	CREBBP (p.Gly2229Ser)	10	0	0	16	branching (2)	branching (2)	6	0	9	2	0.0322

T-LBL	pediatric	UPN207	12	49447842	C	T	674	2	680	0.29 %	546	0	546	0.00 %	342	85	427	19.91 %
T-LBL	pediatric	UPN207	3	178947891	G	A	532	0	533	0.00 %	958	0	963	0.00 %	325	72	399	18.05 %
T-LBL	pediatric	UPN207	12	122247854	G	A	655	0	660	0.00 %	580	0	581	0.00 %	499	109	612	17.81 %
T-LBL	pediatric	UPN207	1	215844615	G	A	354	0	355	0.00 %	250	0	253	0.00 %	207	43	251	17.13 %
T-LBL	pediatric	UPN207	1	215987203	C	T	514	0	515	0.00 %	668	0	670	0.00 %	193	38	231	16.45 %
T-LBL	pediatric	UPN207	16	8995938	C	T	447	0	448	0.00 %	350	0	351	0.00 %	315	63	381	16.54 %
T-LBL	pediatric	UPN207	19	4054707	G	A	369	0	371	0.00 %	777	0	777	0.00 %	490	97	587	16.52 %
T-LBL	pediatric	UPN207	19	4054173	A	G	445	1	453	0.22 %	1162	0	1163	0.00 %	1382	310	1699	18.25 %
T-LBL	pediatric	UPN207	2	16082313	AC	A	563	3	568	0.53 %	459	10	472	2.12 %	877	137	1014	13.51 %
T-LBL	pediatric	UPN207	19	4047989	C	T	49	0	50	0.00 %	113	2	116	1.72 %	482	65	547	11.88 %
T-LBL	pediatric	UPN207	19	10904509	G	A	392	0	393	0.00 %	225	1	226	0.44 %	347	46	394	11.68 %
T-LBL	pediatric	UPN207	4	106155467	G	A	616	2	618	0.32 %	570	2	574	0.35 %	180	34	214	15.89 %
T-LBL	pediatric	UPN207	9	8518261	C	T	696	4	703	0.57 %	735	2	738	0.27 %	459	61	522	11.69 %
T-LBL	pediatric	UPN207	10	104934630	C	T	509	0	510	0.00 %	518	1	519	0.19 %	492	64	557	11.49 %
T-LBL	pediatric	UPN207	14	99641468	C	T	226	0	227	0.00 %	770	0	770	0.00 %	234	37	273	13.55 %
T-LBL	pediatric	UPN207	16	3778767	G	A	581	0	585	0.00 %	526	0	526	0.00 %	1956	363	2322	15.63 %
T-LBL	pediatric	UPN207	7	148506221	T	C	592	0	600	0.00 %	631	0	631	0.00 %	323	57	380	15.00 %
T-LBL	pediatric	UPN207	4	153247289	G	A	592	2	595	0.34 %	752	0	752	0.00 %	362	66	429	15.38 %
T-LBL	pediatric	UPN207	7	151835903	C	T	574	1	577	0.17 %	486	0	487	0.00 %	390	55	446	12.33 %
T-LBL	pediatric	UPN207	7	151846170	C	T	565	3	571	0.53 %	321	0	321	0.00 %	286	50	337	14.84 %
T-LBL	pediatric	UPN207	12	49432632	C	T	680	0	685	0.00 %	597	0	598	0.00 %	640	117	759	15.42 %
T-LBL	pediatric	UPN207	12	49445178	G	A	452	0	453	0.00 %	572	0	572	0.00 %	722	127	850	14.94 %
T-LBL	pediatric	UPN207	7	150706020	C	T	486	1	491	0.20 %	346	0	346	0.00 %	413	77	491	15.68 %
T-LBL	pediatric	UPN207	19	15271729	C	T	486	0	491	0.00 %	344	0	345	0.00 %	823	131	957	13.69 %
T-LBL	pediatric	UPN207	19	15300132	C	T	688	1	692	0.14 %	781	0	781	0.00 %	818	109	929	11.73 %
T-LBL	pediatric	UPN207	19	10943735	A	G	704	1	712	0.14 %	523	0	524	0.00 %	719	105	832	12.62 %
T-LBL	pediatric	UPN207	16	8993546	C	T	579	0	583	0.00 %	520	0	521	0.00 %	442	65	508	12.80 %
T-LBL	pediatric	UPN207	19	4055175	T	C	551	2	561	0.36 %	534	0	536	0.00 %	563	70	633	11.06 %
T-LBL	pediatric	UPN207	19	4054015	C	T	758	2	763	0.26 %	724	0	725	0.00 %	790	132	924	14.29 %
T-LBL	pediatric	UPN207	1	216595321	C	T	638	0	640	0.00 %	632	4	637	0.63 %	297	20	319	6.27 %
T-LBL	pediatric	UPN207	4	106196382	G	A	654	1	661	0.15 %	503	3	507	0.59 %	478	45	524	8.59 %
T-LBL	pediatric	UPN207	9	94495407	G	A	407	1	413	0.24 %	342	2	344	0.58 %	259	29	289	10.03 %
T-LBL	pediatric	UPN207	X	41205629	G	A	306	0	308	0.00 %	640	1	643	0.16 %	248	50	298	16.78 %
T-LBL	pediatric	UPN207	16	3779608	C	T	693	3	698	0.43 %	498	0	498	0.00 %	2286	278	2577	10.79 %
T-LBL	pediatric	UPN207	16	3778363	C	T	567	2	569	0.35 %	391	0	391	0.00 %	557	59	617	9.56 %

2 time points

T-LBL	pediatric	UPN207					SNV/Indel
T-LBL	pediatric	UPN207					SNV/Indel
T-LBL	pediatric	UPN207					SNV/Indel
T-LBL	pediatric	UPN207					SNV/Indel
T-LBL	pediatric	UPN207					SNV/Indel
T-LBL	pediatric	UPN207					SNV/Indel
T-LBL	pediatric	UPN207					SNV/Indel
T-LBL	pediatric	UPN207					SNV/Indel
T-LBL	pediatric	UPN207					SNV/Indel
T-LBL	pediatric	UPN207					SNV/Indel
T-LBL	pediatric	UPN207					SNV/Indel
T-LBL	pediatric	UPN207					SNV/Indel
T-LBL	pediatric	UPN207					SNV/Indel
T-LBL	pediatric	UPN207					SNV/Indel
T-LBL	pediatric	UPN207					SNV/Indel
T-LBL	pediatric	UPN207					SNV/Indel
T-LBL	pediatric	UPN207					SNV/Indel
T-LBL	pediatric	UPN207					SNV/Indel
T-LBL	pediatric	UPN207					SNV/Indel
T-LBL	pediatric	UPN207					SNV/Indel
T-LBL	pediatric	UPN207					SNV/Indel
T-LBL	pediatric	UPN207					SNV/Indel
T-LBL	pediatric	UPN207					SNV/Indel
T-LBL	pediatric	UPN207					SNV/Indel
T-LBL	pediatric	UPN207					SNV/Indel
T-LBL	pediatric	UPN207					SNV/Indel
T-LBL	pediatric	UPN207					SNV/Indel
T-LBL	pediatric	UPN207					SNV/Indel
T-LBL	pediatric	UPN207					SNV/Indel
T-LBL	pediatric	UPN207					SNV/Indel
T-LBL	pediatric	UPN207					SNV/Indel
T-LBL	pediatric	UPN207					SNV/Indel
T-LBL	pediatric	UPN207					SNV/Indel
T-LBL	pediatric	UPN207					SNV/Indel
T-LBL	pediatric	UPN207					SNV/Indel

T-LBL	pediatric	UPN207	JAK3 (p.Ala573Val)	10	0	0	16	branching (2)	branching (2)	6	0	9	2	0.25
T-LBL	pediatric	UPN207	JAK3 (p.Arg899Gln)	10	0	0	16	branching (2)	branching (2)	6	0	9	2	0.25
T-LBL	pediatric	UPN207	KMT2D (p.Pro4918fs)	10	0	0	16	branching (2)	branching (2)	6	0	9	2	0.0625
T-LBL	pediatric	UPN207	MED12 (p.Arg155Gln)	10	0	0	16	branching (2)	branching (2)	6	0	9	1	0
T-LBL	pediatric	UPN207	MYCBP2 (p.Arg1460His)	10	0	0	16	branching (2)	branching (2)	6	0	9	2	0.25
T-LBL	pediatric	UPN207	NOTCH3 (p.Ala1608Thr)	10	0	0	16	branching (2)	branching (2)	6	0	9	2	0.125
T-LBL	pediatric	UPN207	PHF6 (p.Arg343*)	10	0	0	16	branching (2)	branching (2)	6	0	9	1	0
T-LBL	pediatric	UPN207	PIK3CA (p.Arg899His)	10	0	0	16	branching (2)	branching (2)	6	0	9	2	0
T-LBL	pediatric	UPN207	PTPRD (p.Ala1846Thr)	10	0	0	16	branching (2)	branching (2)	6	0	9	2	0.25
T-LBL	pediatric	UPN207	PTPRD (p.Arg588His)	10	0	0	16	branching (2)	branching (2)	6	0	9	2	0.25
T-LBL	pediatric	UPN207	USH2A (p.Gly3195Arg)	10	0	0	16	branching (2)	branching (2)	6	0	9	2	0
T-LBL	pediatric	UPN207	USP7 (p.Val115Ile)	10	0	0	16	branching (2)	branching (2)	6	0	9	2	0.25
T-LBL	pediatric	UPN207	ZBTB7A (p.Arg247Gln)	10	0	0	16	branching (2)	branching (2)	6	0	9	2	0.0322
T-LBL	pediatric	UPN207	DNM2 (p.Pro111fs)	11	0	0	9	branching (2)	branching (2)	7	0	10	2	0.5
T-LBL	pediatric	UPN207	KMT2C (p.Ala4805Thr)	11	0	0	9	branching (2)	branching (2)	7	0	10	2	0.125
T-LBL	pediatric	UPN207	KMT2C (p.Val273Met)	11	0	0	9	branching (2)	branching (2)	7	0	10	2	0.125
T-LBL	pediatric	UPN207	NOTCH3 (p.Ser1580Leu)	11	0	0	9	branching (2)	branching (2)	7	0	10	2	0.1425
T-LBL	pediatric	UPN207	PIK3R1 (p.Ala360Val)	11	0	0	9	branching (2)	branching (2)	7	0	10	2	1
T-LBL	pediatric	UPN209	del in 11p	1	0	53	92	linear	linear	1	0	1	1	1
T-LBL	pediatric	UPN209	del in 12q	1	0	53	92	linear	linear	1	0	1	1	1

2 time points

Supplemental Data 3

T-LBL	pediatric	UPN207	19	17948006	G	A	540	2	547	0.37 %	493	0	496	0.00 %	664	75	740	10.14 %
T-LBL	pediatric	UPN207	19	17942592	C	T	560	0	567	0.00 %	526	0	526	0.00 %	535	44	581	7.57 %
T-LBL	pediatric	UPN207	12	49420995	AG	A	511	0	513	0.00 %	452	0	452	0.00 %	444	42	496	8.47 %
T-LBL	pediatric	UPN207	X	70339931	G	A	352	3	356	0.84 %	353	0	354	0.00 %	280	65	345	18.84 %
T-LBL	pediatric	UPN207	13	77760071	C	T	558	0	558	0.00 %	589	0	589	0.00 %	222	23	245	9.39 %
T-LBL	pediatric	UPN207	19	15281551	C	T	449	0	451	0.00 %	503	0	503	0.00 %	1427	156	1586	9.84 %
T-LBL	pediatric	UPN207	X	133559286	C	T	349	0	350	0.00 %	960	0	960	0.00 %	52	9	61	14.75 %
T-LBL	pediatric	UPN207	3	178947821	G	A	547	0	550	0.00 %	772	0	772	0.00 %	530	48	581	8.26 %
T-LBL	pediatric	UPN207	9	8319965	C	T	469	0	470	0.00 %	527	0	527	0.00 %	141	16	157	10.19 %
T-LBL	pediatric	UPN207	9	8504320	C	T	544	0	547	0.00 %	515	0	516	0.00 %	258	31	291	10.65 %
T-LBL	pediatric	UPN207	1	215987234	C	T	359	1	361	0.28 %	363	0	365	0.00 %	169	19	191	9.95 %
T-LBL	pediatric	UPN207	16	9017112	C	T	488	1	492	0.20 %	421	0	421	0.00 %	266	18	287	6.27 %
T-LBL	pediatric	UPN207	19	4054491	C	T	84	0	84	0.00 %	613	0	614	0.00 %	292	22	314	7.01 %
T-LBL	pediatric	UPN207	19	10943949	C	CA	615	0	619	0.00 %	610	4	611	0.65 %	635	38	638	5.96 %
T-LBL	pediatric	UPN207	7	151841899	C	T	592	1	594	0.17 %	486	0	487	0.00 %	539	27	568	4.75 %
T-LBL	pediatric	UPN207	7	152007083	C	T	561	0	561	0.00 %	532	0	532	0.00 %	495	29	524	5.53 %
T-LBL	pediatric	UPN207	19	15281634	G	A	479	1	484	0.21 %	344	0	344	0.00 %	1077	28	1107	2.53 %
T-LBL	pediatric	UPN207	5	67588988	C	T	698	0	701	0.00 %	576	0	576	0.00 %	264	14	280	5.00 %
T-LBL	pediatric	UPN209																
T-LBL	pediatric	UPN209																

2 time points

T-LBL	pediatric	UPN207														SNV/Indel
T-LBL	pediatric	UPN207														SNV/Indel
T-LBL	pediatric	UPN207														SNV/Indel
T-LBL	pediatric	UPN207														SNV/Indel
T-LBL	pediatric	UPN207														SNV/Indel
T-LBL	pediatric	UPN207														SNV/Indel
T-LBL	pediatric	UPN207														SNV/Indel
T-LBL	pediatric	UPN207														SNV/Indel
T-LBL	pediatric	UPN207														SNV/Indel
T-LBL	pediatric	UPN207														SNV/Indel
T-LBL	pediatric	UPN207														SNV/Indel
T-LBL	pediatric	UPN207														SNV/Indel
T-LBL	pediatric	UPN207														SNV/Indel
T-LBL	pediatric	UPN207														SNV/Indel
T-LBL	pediatric	UPN207														SNV/Indel
T-LBL	pediatric	UPN207														SNV/Indel
T-LBL	pediatric	UPN207														SNV/Indel
T-LBL	pediatric	UPN207														SNV/Indel
T-LBL	pediatric	UPN209	11	30847247	32874844	1	not detected	52.43 %	48.72 %	56.15 %	53	92.11 %	90.92 %	93.31 %	53	CNV
T-LBL	pediatric	UPN209	12	110696466	111887659	1	not detected	53.69 %	43.96 %	63.42 %	20	91.74 %	89.18 %	94.30 %	20	CNV

Supplemental Data 3

Disease	Category	ID	Variant	Clone	CCF G	CCF P	Evolution P	Nested	Siblings	Parent	Alleles available	Alleles not mutated
T-ALL	adult	UPN001	dup in 19q	1	0	68	linear	1	0	0	3	3
T-ALL	adult	UPN001	del in 9p → becoming LOH by subsequent dup9	1	0	68	linear	1	0	0	1	1
T-ALL	adult	UPN001	del in 12p	2	0	50	linear	2	0	1	1	1
T-ALL	adult	UPN001	del in 17q	2	0	50	linear	2	0	1	1	1
T-ALL	adult	UPN001	LOH in Xq	2	0	50	linear	2	0	1	2	2
T-ALL	adult	UPN001	dupX	2	0	50	linear	2	0	1	3	3
T-ALL	adult	UPN001	dup9	2	0	50	linear	2	0	1	3	3
T-ALL	adult	UPN001	dup in 19p	2	0	50	linear	2	0	1	3	3
T-ALL	adult	UPN001	NOTCH1 (p.Phe1592_Leu1593insAsn)	2	0	50	linear	2	0	1	3	1
T-ALL	adult	UPN001	PHF6 (p.Ser138fs)	2	0	50	linear	2	0	1	2	1
T-ALL	adult	UPN001	FBXW7 (p.Arg479Gln)	2	0	50	linear	2	0	1	2	1
T-ALL	adult	UPN001	CREBBP (p.Asp1253_Thr1260del)	3	0	13	linear	3	0	2	2	1
T-ALL	adult	UPN002	del in 11p	1	0	65	linear	1	0	0	1	1
T-ALL	adult	UPN002	del in 12p	1	0	65	linear	1	0	0	1	1
T-ALL	adult	UPN002	del in 17q	1	0	65	linear	1	0	0	1	1
T-ALL	adult	UPN002	del in 18p	1	0	65	linear	1	0	0	1	1
T-ALL	adult	UPN002	PHF6 (p.Val299_Lys300delinsArg)	1	0	65	linear	1	0	0	2	0
T-ALL	adult	UPN002	NRAS (p.Gly12Asp)	1	0	65	linear	1	0	0	2	1
T-ALL	adult	UPN003	dup in 7p	1	0	86	branching (3)	1	0	0	3	3
T-ALL	adult	UPN003	PTEN (p.Arg233fs)	2	0	13	branching (3)	2	2	1	2	1
T-ALL	adult	UPN003	PTEN (p.Arg234_Glu235delinsProLeuProPheTerAlaGlyGluAsp)	3	0	7	branching (3)	2	2	1	2	1

1 time point

Supplemental Data 3

			SNVs + Indels				Germline				Primary			
Disease	Category	ID	Chr	Pos	Ref	Alt	#REF	#ALT	DP	VAF	#REF	#ALT	DP	VAF
T-ALL	adult	UPN001												
T-ALL	adult	UPN001												
T-ALL	adult	UPN001												
T-ALL	adult	UPN001												
T-ALL	adult	UPN001												
T-ALL	adult	UPN001												
T-ALL	adult	UPN001												
T-ALL	adult	UPN001												
T-ALL	adult	UPN001	9	139399367	G	GTTA	1498	0	1501	0.00 %	1966	781	1974	39.56 %
T-ALL	adult	UPN001	X	133527976	T	TTGGGGGC	941	0	946	0.00 %	732	177	738	23.98 %
T-ALL	adult	UPN001	4	153247366	C	T	988	0	991	0.00 %	719	226	946	23.89 %
T-ALL	adult	UPN001	16	3801697	CCCCAGAGAAAATGACAGGACGGTACTTACGTCTGGGGCTGTGAAGGGTCGTCA	C	674	0	675	0.00 %	569	41	611	6.71 %
T-ALL	adult	UPN002												
T-ALL	adult	UPN002												
T-ALL	adult	UPN002												
T-ALL	adult	UPN002												
T-ALL	adult	UPN002	X	133551254	GTGTTAA	GTAG	619	3	620	0.48 %	190	312	502	62.15 %
T-ALL	adult	UPN002	1	115258747	C	T	2009	0	2011	0.00 %	937	445	1385	32.13 %
T-ALL	adult	UPN003												
T-ALL	adult	UPN003	10	89717671	AC	AGGGATCAACAGGTACGCA	965	0	970	0.00 %	814	60	910	6.59 %
T-ALL	adult	UPN003	10	89717676	GGGAA	CACTCCCCTTTTAAGCCGGAGAGGAT	920	4	922	0.43 %	847	32	879	3.64 %

Supplemental Data 3

			CNVs													
							Germline				Primary				
Disease	Category	ID	Chr	Start	End	Type	CCF	lower	upper	#SNPs	CCF	lower	upper	#SNPs	Varianttype
T-ALL	adult	UPN001	19	37685602	59097933	2	not detected				66.97 %	66.09 %	67.85 %	1146	CNV
T-ALL	adult	UPN001	9	16054088	38782387	2	not detected				68.28 %	67.61 %	68.94 %	978	CNV
T-ALL	adult	UPN001	12	10296130	26728960	1	not detected				50.21 %	49.18 %	51.25 %	802	CNV
T-ALL	adult	UPN001	17	29007975	30415430	1	not detected				59.02 %	51.24 %	66.80 %	25	CNV
T-ALL	adult	UPN001	X	70445165	155270560	2	not detected				50.10 %	49.75 %	50.45 %	3643	CNV
T-ALL	adult	UPN001	X	0	155270560	3	not detected				47.65 %	46.92 %	48.38 %	3229	CNV
T-ALL	adult	UPN001	9	0	141213430	3	not detected				48.32 %	47.08 %	49.55 %	3782	CNV
T-ALL	adult	UPN001	19	13108717	14828116	3	not detected				47.47 %	42.67 %	52.27 %	75	CNV
T-ALL	adult	UPN001													SNV/Indel
T-ALL	adult	UPN001													SNV/Indel
T-ALL	adult	UPN001													SNV/Indel
T-ALL	adult	UPN001													SNV/Indel
T-ALL	adult	UPN002	11	31647635	32493062	1	not detected				73.89 %	68.35 %	79.43 %	31	CNV
T-ALL	adult	UPN002	12	0	31069369	1	not detected				69.24 %	68.54 %	69.94 %	1745	CNV
T-ALL	adult	UPN002	17	28912879	30377486	1	not detected				69.27 %	64.31 %	74.24 %	46	CNV
T-ALL	adult	UPN002	18	0	15386489	1	not detected				68.85 %	67.98 %	69.72 %	968	CNV
T-ALL	adult	UPN002													SNV/Indel
T-ALL	adult	UPN002													SNV/Indel
T-ALL	adult	UPN003	7	3532126	4631793	3	not detected				86.01 %	79.26 %	92.76 %	65	CNV
T-ALL	adult	UPN003													SNV/Indel
T-ALL	adult	UPN003													SNV/Indel

1 time point

Supplemental Data 3

T-ALL	adult	UPN003	PTEN (p.Arg233delinsPheArgTer)	4	0	7	branching (3)	2	2	1	2	1
T-ALL	adult	UPN004	MED12 (p.Ile320fs)	1	0	71	linear	1	0	0	2	1
T-ALL	adult	UPN004	PIK3R1 (p.Thr454_Gln455insProLeu)	2	0	60	linear	2	0	1	2	1
T-ALL	adult	UPN004	PTEN (p.Glu235fs)	2	0	60	linear	2	0	1	2	0
T-ALL	adult	UPN004	PTEN (p.Leu247fs)	2	0	60	linear	2	0	1	2	0
T-ALL	adult	UPN004	SUZ12 (p.Val186fs)	3	0	50	linear	3	0	2	2	1
T-ALL	adult	UPN004	del in 9q	4	0	23	linear	4	0	3	1	1
T-ALL	adult	UPN005	LOH9p	1	0	98	linear	1	0	0	2	2
T-ALL	adult	UPN005	del in 1p	2	0	90	linear	2	0	1	1	1
T-ALL	adult	UPN005	del in 1p	2	0	90	linear	2	0	1	1	1
T-ALL	adult	UPN005	del in 8p	2	0	90	linear	2	0	1	1	1
T-ALL	adult	UPN005	del in 9q	2	0	90	linear	2	0	1	1	1
T-ALL	adult	UPN005	del in 12p	2	0	90	linear	2	0	1	1	1
T-ALL	adult	UPN005	del in 12q	2	0	90	linear	2	0	1	1	1
T-ALL	adult	UPN005	del in 17p	2	0	90	linear	2	0	1	1	1
T-ALL	adult	UPN005	RUNX1 (p.Lys171*)	2	0	90	linear	2	0	1	2	1
T-ALL	adult	UPN005	NOTCH1 (p.Val1721Met)	2	0	90	linear	2	0	1	2	1
T-ALL	adult	UPN005	DNM2 (p.Gln768*)	2	0	90	linear	2	0	1	2	1
T-ALL	adult	UPN005	USP7 (p.Glu68fs)	3	0	79	linear	3	0	2	2	1
T-ALL	adult	UPN005	IL7R (p.Leu243_Thr244insGlyArgGlyCysLeu)	4	0	26	linear	4	0	3	2	1
T-ALL	adult	UPN006	del in 11p	1	0	87	linear	1	0	0	1	1
T-ALL	adult	UPN006	del in 13q	1	0	87	linear	1	0	0	1	1
T-ALL	adult	UPN006	del in 17q	1	0	87	linear	1	0	0	1	1
T-ALL	adult	UPN006	dup in 1q	2	0	75	linear	2	0	1	3	3
T-ALL	adult	UPN006	del in 7p	3	0	55	linear	3	0	2	1	1
T-ALL	adult	UPN007	dup6	1	0	60	linear	1	0	0	3	3
T-ALL	adult	UPN007	dup7	1	0	60	linear	1	0	0	3	3
T-ALL	adult	UPN007	dup8	1	0	60	linear	1	0	0	3	3
T-ALL	adult	UPN007	dup11	1	0	60	linear	1	0	0	3	3
T-ALL	adult	UPN007	dup13	1	0	60	linear	1	0	0	3	3
T-ALL	adult	UPN007	dup17	1	0	60	linear	1	0	0	3	3

1 time point

T-ALL	adult	UPN003	10	89717671	AC	ATTCCGCT	965	2	965	0.21 %	814	30	910	3.30 %
T-ALL	adult	UPN004	X	70341524	T	TGTTCG	900	0	905	0.00 %	786	577	814	70.88 %
T-ALL	adult	UPN004	5	67589599	T	TCCCCTC	592	0	592	0.00 %	490	160	491	32.59 %
T-ALL	adult	UPN004	10	89717674	A	ACGGG	827	0	830	0.00 %	823	263	827	31.80 %
T-ALL	adult	UPN004	10	89717712	CGTT	CTTCCCTTTGTG	841	0	844	0.00 %	825	235	828	28.38 %
T-ALL	adult	UPN004	17	30300212	GA	G	699	0	700	0.00 %	739	241	983	24.52 %
T-ALL	adult	UPN004												
T-ALL	adult	UPN005												
T-ALL	adult	UPN005												
T-ALL	adult	UPN005												
T-ALL	adult	UPN005												
T-ALL	adult	UPN005												
T-ALL	adult	UPN005												
T-ALL	adult	UPN005												
T-ALL	adult	UPN005												
T-ALL	adult	UPN005	21	36231873	T	A	1048	1	1051	0.10 %	660	573	1234	46.43 %
T-ALL	adult	UPN005	9	139397640	C	T	1204	2	1208	0.17 %	1004	792	1799	44.02 %
T-ALL	adult	UPN005	19	10940813	C	T	1109	0	1112	0.00 %	743	571	1319	43.29 %
T-ALL	adult	UPN005	16	9017254	G	GGACCCCA	1085	0	1090	0.00 %	1208	479	1212	39.52 %
T-ALL	adult	UPN005	5	35874570	A	ACTAGGGCGCGGGTGC	1102	0	1106	0.00 %	1265	165	1271	12.98 %
T-ALL	adult	UPN006												
T-ALL	adult	UPN006												
T-ALL	adult	UPN006												
T-ALL	adult	UPN006												
T-ALL	adult	UPN006												
T-ALL	adult	UPN007												
T-ALL	adult	UPN007												
T-ALL	adult	UPN007												
T-ALL	adult	UPN007												
T-ALL	adult	UPN007												
T-ALL	adult	UPN007												

T-ALL	adult	UPN003										SNV/Indel
T-ALL	adult	UPN004										SNV/Indel
T-ALL	adult	UPN004										SNV/Indel
T-ALL	adult	UPN004										SNV/Indel
T-ALL	adult	UPN004										SNV/Indel
T-ALL	adult	UPN004										SNV/Indel
T-ALL	adult	UPN004	9	70988363	106352698	1	not detected	22.99 %	22.06 %	23.93 %	1152	CNV
T-ALL	adult	UPN005	9	0	36997324	2	not detected	98.02 %	97.83 %	98.22 %	1698	CNV
T-ALL	adult	UPN005	1	23390522	24741042	1	not detected	95.14 %	94.10 %	96.19 %	48	CNV
T-ALL	adult	UPN005	1	82726325	83604320	1	not detected	93.74 %	92.68 %	94.79 %	33	CNV
T-ALL	adult	UPN005	8	130249199	130560035	1	not detected	88.70 %	80.06 %	97.35 %	25	CNV
T-ALL	adult	UPN005	9	131462039	134032811	1	not detected	94.41 %	93.58 %	95.25 %	149	CNV
T-ALL	adult	UPN005	12	12866352	12990148	1	not detected	89.22 %	82.97 %	95.47 %	12	CNV
T-ALL	adult	UPN005	12	111107864	112037090	1	not detected	90.31 %	82.29 %	98.33 %	17	CNV
T-ALL	adult	UPN005	17	20827600	22242355	1	not detected	58.48 %	35.60 %	81.36 %	15	CNV
T-ALL	adult	UPN005										SNV/Indel
T-ALL	adult	UPN005										SNV/Indel
T-ALL	adult	UPN005										SNV/Indel
T-ALL	adult	UPN005										SNV/Indel
T-ALL	adult	UPN005										SNV/Indel
T-ALL	adult	UPN006	11	0	39815247	1	not detected	87.94 %	87.65 %	88.23 %	1920	CNV
T-ALL	adult	UPN006	13	27258429	30036364	1	not detected	87.63 %	86.85 %	88.41 %	144	CNV
T-ALL	adult	UPN006	17	28380739	30415430	1	not detected	85.87 %	83.68 %	88.05 %	50	CNV
T-ALL	adult	UPN006	1	181718961	249110238	3	not detected	76.15 %	75.25 %	77.05 %	2700	CNV
T-ALL	adult	UPN006	7	14173817	54671049	1	not detected	55.40 %	54.86 %	55.93 %	1871	CNV
T-ALL	adult	UPN007	6	0	171115067	3	not detected	59.43%	57.94%	60.92%	5210	CNV
T-ALL	adult	UPN007	7	0	159138663	3	not detected	58.22%	56.32%	60.12%	4100	CNV
T-ALL	adult	UPN007	8	0	146364022	3	not detected	59.99%	58.22%	61.76%	2295	CNV
T-ALL	adult	UPN007	11	0	135006516	3	not detected	58.37%	57.48%	59.26%	4792	CNV
T-ALL	adult	UPN007	13	0	115169878	3	not detected	59.24%	58.55%	59.92%	3995	CNV
T-ALL	adult	UPN007	17	0	81195210	3	not detected	59.44%	57.51%	61.37%	1082	CNV

T-ALL	adult	UPN007	dup18	1	0	60	linear	1	0	0	3	3
T-ALL	adult	UPN007	dup19	1	0	60	linear	1	0	0	3	3
T-ALL	adult	UPN007	dup20	1	0	60	linear	1	0	0	3	3
T-ALL	adult	UPN007	dup21	1	0	60	linear	1	0	0	3	3
T-ALL	adult	UPN007	PHF6 (p.Arg275*)	1	0	60	linear	1	0	0	2	1
T-ALL	adult	UPN007	BCL11B (p.Val109fs)	1	0	60	linear	1	0	0	2	1
T-ALL	adult	UPN007	KDM6A (p.Arg1118fs)	1	0	60	linear	1	0	0	2	1
T-ALL	adult	UPN008	LOH in 11p	1	0	84	linear	1	0	0	2	2
T-ALL	adult	UPN008	del in 12p	2	0	67	linear	2	0	1	1	1
T-ALL	adult	UPN008	SUZ12 (p.His620Arg)	3	0	60	linear	3	0	2	2	0.5
T-ALL	adult	UPN008	JAK3 (p.Arg657Gln)	3	0	60	linear	3	0	2	2	1
T-ALL	adult	UPN008	SUZ12 (p.Ile428Lys)	3	0	60	linear	3	0	2	2	0.5
T-ALL	adult	UPN008	PHF6 (p.Leu783Phe)	4	0	36	linear	4	0	3	2	1
T-ALL	adult	UPN008	JAK1 (p.Leu783Phe)	5	0	24	linear	5	0	4	2	1
T-ALL	adult	UPN008	JAK1 (p.Thr901Lys)	6	0	10	linear	6	0	5	2	0.5
T-ALL	adult	UPN009	TET2 (p.Arg1917Lys)	1	0	44	linear	1	0	0	2	1
T-ALL	adult	UPN009	RUNX1 (p.Ala142fs)	2	0	31	linear	2	0	1	2	1
T-ALL	adult	UPN010	del in 1p	1	0	90	branching (5)	1	0	0	1	1
T-ALL	adult	UPN010	del in 1p	1	0	90	branching (5)	1	0	0	1	1
T-ALL	adult	UPN010	del in 2p	1	0	90	branching (5)	1	0	0	1	1
T-ALL	adult	UPN010	del in 8q	1	0	90	branching (5)	1	0	0	1	1
T-ALL	adult	UPN010	LOH in 11p	1	0	90	branching (5)	1	0	0	2	2
T-ALL	adult	UPN010	del in 14q	1	0	90	branching (5)	1	0	0	1	1
T-ALL	adult	UPN010	del in 14q	1	0	90	branching (5)	1	0	0	1	1
T-ALL	adult	UPN010	del in 16p	1	0	90	branching (5)	1	0	0	1	1
T-ALL	adult	UPN010	del in 18p	1	0	90	branching (5)	1	0	0	1	1
T-ALL	adult	UPN010	del in 18p	1	0	90	branching (5)	1	0	0	1	1
T-ALL	adult	UPN010	PHF6 (p.Arg320*)	1	0	90	branching (5)	1	0	0	2	0
T-ALL	adult	UPN010	STAT5B (p.Ile704Leu)	1	0	90	branching (5)	1	0	0	2	1
T-ALL	adult	UPN010	del in 12p	2	0	83	branching (5)	2	0	1	1	1
T-ALL	adult	UPN010	NOTCH1 (p.Leu1593Pro)	3	0	70	branching (5)	3	0	2	2	1
T-ALL	adult	UPN010	KRAS (p.Gly12Asp)	4	0	25	branching (5)	4	4	3	2	1
T-ALL	adult	UPN010	NOTCH1 (p.Arg1598dup)	5	0	11	branching (5)	4	4	3	2	0
T-ALL	adult	UPN010	NRAS (p.Gly13Val)	6	0	7	branching (5)	4	4	3	2	1

1 time point

Supplemental Data 3

T-ALL	adult	UPN007												
T-ALL	adult	UPN007												
T-ALL	adult	UPN007												
T-ALL	adult	UPN007												
T-ALL	adult	UPN007	X	133549136	C	T	695	0	695	0.00 %	625	262	889	29.47 %
T-ALL	adult	UPN007	14	99723911	T	TTTCC	1306	0	1309	0.00 %	1183	338	1187	28.48 %
T-ALL	adult	UPN007	X	44942749	TGC	TGGG	608	2	609	0.33 %	653	245	898	27.28 %
T-ALL	adult	UPN008												
T-ALL	adult	UPN008												
T-ALL	adult	UPN008	17	30323881	A	G	655	0	655	0.00 %	472	218	692	31.50 %
T-ALL	adult	UPN008	19	17945969	C	T	1491	0	1493	0.00 %	1141	527	1673	31.50 %
T-ALL	adult	UPN008	17	30320342	T	A	679	0	680	0.00 %	457	195	653	29.86 %
T-ALL	adult	UPN008	X	133511772	ACCA	ACCGATCGCTTGCCC	900	0	901	0.00 %	876	136	877	15.51 %
T-ALL	adult	UPN008	1	65309803	G	A	1209	0	1209	0.00 %	1164	153	1320	11.59 %
T-ALL	adult	UPN008	1	65305426	G	T	1514	0	1518	0.00 %	1514	85	1603	5.30 %
T-ALL	adult	UPN009	4	106197354	G	A	1227	57	1285	4.44 %	1026	291	1325	21.96 %
T-ALL	adult	UPN009	21	36252938	C	CGGGA	1406	0	1408	0.00 %	1287	200	1292	15.48 %
T-ALL	adult	UPN010												
T-ALL	adult	UPN010												
T-ALL	adult	UPN010												
T-ALL	adult	UPN010												
T-ALL	adult	UPN010												
T-ALL	adult	UPN010												
T-ALL	adult	UPN010												
T-ALL	adult	UPN010												
T-ALL	adult	UPN010												
T-ALL	adult	UPN010												
T-ALL	adult	UPN010	X	133551319	C	T	377	0	379	0.00 %	51	409	460	88.91 %
T-ALL	adult	UPN010	17	40354794	T	G	1157	1	1161	0.09 %	1032	803	1837	43.71 %
T-ALL	adult	UPN010												
T-ALL	adult	UPN010	9	139399365	A	G	1230	1	1235	0.08 %	1209	640	1852	34.56 %
T-ALL	adult	UPN010	12	25398284	C	T	890	0	891	0.00 %	1268	185	1460	12.67 %
T-ALL	adult	UPN010	9	139399348	C	CCCT	1232	0	1238	0.00 %	1889	105	1892	5.55 %
T-ALL	adult	UPN010	1	115258744	C	A	947	1	948	0.11 %	1552	55	1611	3.41 %

1 time point

T-ALL	adult	UPN007	18	0	78077248	3	not detected	59.25%	58.08%	60.43%	3270	CNV
T-ALL	adult	UPN007	19	0	59128983	3	not detected	61.43 %	57.62 %	65.25 %	128	CNV
T-ALL	adult	UPN007	20	0	63025520	3	not detected	58.49 %	56.88 %	60.11 %	668	CNV
T-ALL	adult	UPN007	21	0	48129895	3	not detected	60.12 %	58.94 %	61.31 %	1309	CNV
T-ALL	adult	UPN007										SNV/Indel
T-ALL	adult	UPN007										SNV/Indel
T-ALL	adult	UPN007										SNV/Indel
T-ALL	adult	UPN008	11	0	36366828	2	not detected	84.26 %	83.95 %	84.56 %	1868	CNV
T-ALL	adult	UPN008	12	10370284	21390973	1	not detected	66.97 %	65.37 %	68.57 %	518	CNV
T-ALL	adult	UPN008										SNV/Indel
T-ALL	adult	UPN008										SNV/Indel
T-ALL	adult	UPN008										SNV/Indel
T-ALL	adult	UPN008										SNV/Indel
T-ALL	adult	UPN008										SNV/Indel
T-ALL	adult	UPN008										SNV/Indel
T-ALL	adult	UPN009										SNV/Indel
T-ALL	adult	UPN009										SNV/Indel
T-ALL	adult	UPN010	1	5964799	7857492	1	not detected	91.90 %	91.27 %	92.53 %	76	CNV
T-ALL	adult	UPN010	1	23005597	25045470	1	not detected	91.66 %	91.02 %	92.31 %	73	CNV
T-ALL	adult	UPN010	2	60169426	60484834	1	not detected	92.25 %	77.13 %	107.37 %	2	CNV
T-ALL	adult	UPN010	8	119883000	120425595	1	not detected	92.20 %	91.23 %	93.17 %	34	CNV
T-ALL	adult	UPN010	11	0	44613265	2	not detected	95.36 %	95.24 %	95.48 %	2013	CNV
T-ALL	adult	UPN010	14	88502480	90131248	1	not detected	91.99 %	91.38 %	92.61 %	66	CNV
T-ALL	adult	UPN010	14	98880365	99266175	1	not detected	91.38 %	89.78 %	92.98 %	23	CNV
T-ALL	adult	UPN010	16	3531204	6196443	1	not detected	91.68 %	91.12 %	92.24 %	142	CNV
T-ALL	adult	UPN010	18	2452614	2660866	1	not detected	90.63 %	87.86 %	93.40 %	9	CNV
T-ALL	adult	UPN010	18	11708430	12995742	1	not detected	89.23 %	85.38 %	93.08 %	47	CNV
T-ALL	adult	UPN010										SNV/Indel
T-ALL	adult	UPN010										SNV/Indel
T-ALL	adult	UPN010	12	7142875	18267486	1	not detected	83.21 %	82.72 %	83.70 %	457	CNV
T-ALL	adult	UPN010										SNV/Indel
T-ALL	adult	UPN010										SNV/Indel
T-ALL	adult	UPN010										SNV/Indel
T-ALL	adult	UPN010										SNV/Indel

1 time point

T-ALL	adult	UPN010	NOTCH1 (p.Leu1678Pro)	7	0	6	branching (5)	4	4	3	2	0.5
T-ALL	adult	UPN010	NRAS (p.Gly12Ala)	8	0	5	branching (5)	4	4	3	2	1
T-ALL	adult	UPN011	NOTCH1 (p.Ser1708Pro)	1	0	100	linear	1	0	0	2	1
T-ALL	adult	UPN011	LOH in 9q	2	0	60	linear	2	0	1	2	2
T-ALL	adult	UPN011	FBXW7 (p.Arg465His)	2	0	60	linear	2	0	1	2	1
T-ALL	adult	UPN011	NRAS (p.Gly60Glu)	2	0	60	linear	2	0	1	2	1
T-ALL	adult	UPN011	del in 9p	3	0	45	linear	3	0	2	1	1
T-ALL	adult	UPN011	del in 15q	3	0	45	linear	3	0	2	1	1
T-ALL	adult	UPN013	LOH in 9p	1	0	92	linear	1	0	0	2	2
T-ALL	adult	UPN013	dup in 10q	2	0	70	linear	2	0	1	3	3
T-ALL	adult	UPN013	USP7 (p.Val256fs)	2	0	70	linear	2	0	1	2	1
T-ALL	adult	UPN013	NOTCH1 (p.Gln2459*)	3	0	50	linear	3	0	2	2	1
T-ALL	adult	UPN013	NOTCH1 (p.Ser2423*)	4	0	40	linear	4	0	3	2	0
T-ALL	adult	UPN013	NOTCH1 (p.Leu1579_Glu1583delinsGlnLys)	4	0	40	linear	4	0	3	2	1
T-ALL	adult	UPN013	PTEN (p.Asp368_Val369delinsMetLeuPro)	4	0	40	linear	4	0	3	2	1
T-ALL	adult	UPN013	PIK3R1 (p.Leu449delinsArgThr)	5	0	10	linear	5	0	4	2	0.5
T-ALL	adult	UPN013	PIK3R1 (p.Thr576del)	5	0	10	linear	5	0	4	2	0.5
T-ALL	adult	UPN014	del in 2p	1	0	86	linear	1	0	0	1	1
T-ALL	adult	UPN014	del in 6q	1	0	86	linear	1	0	0	1	1
T-ALL	adult	UPN014	del in 9p	1	0	86	linear	1	0	0	1	1
T-ALL	adult	UPN014	del in 9q	1	0	86	linear	1	0	0	1	1
T-ALL	adult	UPN014	PHF6 (p.Lys133fs)	1	0	86	linear	1	0	0	2	0
T-ALL	adult	UPN014	NOTCH1 (p.Pro2334fs)	1	0	86	linear	1	0	0	2	1
T-ALL	adult	UPN014	DNM2 (p.Asn582fs)	2	0	62	linear	2	0	1	2	1
T-ALL	adult	UPN014	SUZ12 (p.Gly43fs)	2	0	62	linear	2	0	1	2	1
T-ALL	adult	UPN014	del in 1p	3	0	30	linear	3	0	2	1	1
T-ALL	adult	UPN015	LOH in 4q	1	100	100	linear	1	0	0	2	2
T-ALL	adult	UPN015	LOH in 14q	1	100	100	linear	1	0	0	2	2
T-ALL	adult	UPN015	del in 3p	2	0	94	linear	2	0	1	1	1
T-ALL	adult	UPN015	del in 5q	2	0	94	linear	2	0	1	1	1

Supplemental Data 3

T-ALL	adult	UPN010	9	139397768	A	G	1036	0	1040	0.00 %	1636	51	1692	3.01 %
T-ALL	adult	UPN010	1	115258747	C	G	919	2	924	0.22 %	1518	39	1563	2.50 %
T-ALL	adult	UPN011	9	139397679	A	G	1424	0	1425	0.00 %	991	938	1931	48.58 %
T-ALL	adult	UPN011												
T-ALL	adult	UPN011	4	153249384	C	T	1099	0	1101	0.00 %	1045	446	1499	29.75 %
T-ALL	adult	UPN011	1	115256532	C	T	900	0	901	0.00 %	900	319	1225	26.04 %
T-ALL	adult	UPN011												
T-ALL	adult	UPN011												
T-ALL	adult	UPN013												
T-ALL	adult	UPN013												
T-ALL	adult	UPN013	16	9010967	ACG	ACCCA	777	3	780	0.38 %	622	374	1001	37.36 %
T-ALL	adult	UPN013	9	139390816	G	A	1237	1	1244	0.08 %	1093	370	1465	25.26 %
T-ALL	adult	UPN013	9	139390923	G	C	1526	1	1532	0.07 %	1392	290	1685	17.21 %
T-ALL	adult	UPN013	9	139399396	CCGGCGGCATCA	TCT	1441	5	1445	0.35 %	1362	251	1620	15.49 %
T-ALL	adult	UPN013	10	89725118	AGATGTT	AATGTTACCC	887	2	887	0.23 %	839	125	964	12.97 %
T-ALL	adult	UPN013	5	67589582	TT	AGGAC	579	4	583	0.69 %	679	46	726	6.34 %
T-ALL	adult	UPN013	5	67591131	AGAC	A	722	0	724	0.00 %	792	34	828	4.11 %
T-ALL	adult	UPN014												
T-ALL	adult	UPN014												
T-ALL	adult	UPN014												
T-ALL	adult	UPN014												
T-ALL	adult	UPN014	X	133527961	AA	ATTGAAGGCGCC	505	0	507	0	34	244	287	85.02%
T-ALL	adult	UPN014	9	139391191	G	GT	1785	0	1786	0	1489	668	1527	43.75%
T-ALL	adult	UPN014	19	10930723	T	TGTCCAAGGCG	1547	0	1551	0	1174	373	1176	31.72%
T-ALL	adult	UPN014	17	30264386	T	TCCGGC	228	0	229	0	325	101	327	30.89%
T-ALL	adult	UPN014												
T-ALL	adult	UPN015												
T-ALL	adult	UPN015												
T-ALL	adult	UPN015												
T-ALL	adult	UPN015												

1 time point

T-ALL	adult	UPN010										SNV/Indel
T-ALL	adult	UPN010										SNV/Indel
T-ALL	adult	UPN011										SNV/Indel
T-ALL	adult	UPN011	9	136037259	141068637	2	not detected	63.64 %	62.62 %	64.67 %	341	CNV
T-ALL	adult	UPN011										SNV/Indel
T-ALL	adult	UPN011										SNV/Indel
T-ALL	adult	UPN011	9	19758771	22751855	1	not detected	35.60 %	31.96 %	39.24 %	156	CNV
T-ALL	adult	UPN011	15	41806373	43235557	1	not detected	44.62 %	41.45 %	47.78 %	38	CNV
T-ALL	adult	UPN013	9	0	32673400	2	not detected	92.38 %	92.06 %	92.71 %	1619	CNV
T-ALL	adult	UPN013	10	88684336	94821204	3	not detected	67.56 %	64.38 %	70.74 %	273	CNV
T-ALL	adult	UPN013										SNV/Indel
T-ALL	adult	UPN013										SNV/Indel
T-ALL	adult	UPN013										SNV/Indel
T-ALL	adult	UPN013										SNV/Indel
T-ALL	adult	UPN013										SNV/Indel
T-ALL	adult	UPN013										SNV/Indel
T-ALL	adult	UPN013										SNV/Indel
T-ALL	adult	UPN014	2	39513075	41219864	1	not detected	89.43 %	88.44 %	90.41 %	57	CNV
T-ALL	adult	UPN014	6	99869216	100501128	1	not detected	89.94 %	88.61 %	91.28 %	25	CNV
T-ALL	adult	UPN014	9	20617810	39162801	1	not detected	82.19 %	80.37 %	84.02 %	708	CNV
T-ALL	adult	UPN014	9	108198444	110126658	1	not detected	89.32 %	87.97 %	90.68 %	75	CNV
T-ALL	adult	UPN014										SNV/Indel
T-ALL	adult	UPN014										SNV/Indel
T-ALL	adult	UPN014										SNV/Indel
T-ALL	adult	UPN014										SNV/Indel
T-ALL	adult	UPN014	1	4247126	7314630	1	not detected	30.66 %	28.57 %	32.76 %	170	CNV
T-ALL	adult	UPN015	4	148092130	171776427	2	100.00 %	100.00 %				CNV
T-ALL	adult	UPN015	14	34314291	50442382	2	100.00 %	100.00 %				CNV
T-ALL	adult	UPN015	3	71196518	71526270	1	not detected	94.76 %	93.94 %	95.59 %	28	CNV
T-ALL	adult	UPN015	5	133604789	133958309	1	not detected	94.42 %	92.88 %	95.95 %	13	CNV

1 time point

T-ALL	adult	UPN015	del in 6q	2	0	94	linear	2	0	1	1	1
T-ALL	adult	UPN015	del in 8p	2	0	94	linear	2	0	1	1	1
T-ALL	adult	UPN015	del in 11q	2	0	94	linear	2	0	1	1	1
T-ALL	adult	UPN015	del in 11q	2	0	94	linear	2	0	1	1	1
T-ALL	adult	UPN015	del in 12p	2	0	94	linear	2	0	1	1	1
T-ALL	adult	UPN015	del in 13q	2	0	94	linear	2	0	1	1	1
T-ALL	adult	UPN015	del in 17q	2	0	94	linear	2	0	1	1	1
T-ALL	adult	UPN015	del in 1p	3	0	85	linear	3	0	2	1	1
T-ALL	adult	UPN015	JAK1 (p.Tyr652His)	4	0	11	linear	4	0	3	2	1
T-ALL	adult	UPN015	JAK1 (p.Tyr652Asp)	5	0	6	linear	5	0	4	2	0
T-ALL	adult	UPN015	JAK3 (p.Met511Ile)	5	0	6	linear	5	0	4	2	1
T-ALL	adult	UPN016	dup in 9p	1	0	63	linear	1	0	0	3	3
T-ALL	adult	UPN016	dup in 9p	1	0	63	linear	1	0	0	3	3
T-ALL	adult	UPN016	PHF6 (p.His330Arg)	2	0	26	linear	2	0	1	2	0
T-ALL	adult	UPN016	NRAS (p.Gly12Ser)	2	0	26	linear	2	0	1	2	1
T-ALL	adult	UPN016	NOTCH1 (p.Pro2514fs)	3	0	18	linear	3	0	2	2	0.5
T-ALL	adult	UPN016	FBXW7 (p.Arg479Gln)	3	0	18	linear	3	0	2	2	1
T-ALL	adult	UPN016	NOTCH1 (p.Leu1678Pro)	3	0	18	linear	3	0	2	2	0.5
T-ALL	adult	UPN016	SUZ12 (p.Arg129Trp)	3	0	18	linear	3	0	2	2	1
T-ALL	adult	UPN017	del in 9p	1	0	87	branching (2)	1	0	0	1	1
T-ALL	adult	UPN017	del in 11p	1	0	87	branching (2)	1	0	0	1	1
T-ALL	adult	UPN017	dup in 15q	2	0	75	branching (2)	2	0	1	3	3
T-ALL	adult	UPN017	PHF6 (p.His240_Tyr241delinsGluGluGlu)	2	0	75	branching (2)	2	0	1	2	1
T-ALL	adult	UPN017	del in 9q	3	0	54	branching (2)	3	0	2	1	1
T-ALL	adult	UPN017	dup8	3	0	54	branching (2)	3	0	2	3	3
T-ALL	adult	UPN017	DNM2 (p.Arg385*)	4	0	30	branching (2)	4	1	3	2	1
T-ALL	adult	UPN017	NOTCH1 (p.Val2443fs)	4	0	30	branching (2)	4	1	3	2	0
T-ALL	adult	UPN017	NOTCH1 (p.Leu1593Pro)	4	0	30	branching (2)	4	1	3	2	0
T-ALL	adult	UPN017	NOTCH1 (p.Phe2433fs)	4	0	30	branching (2)	4	1	3	2	0
T-ALL	adult	UPN017	NOTCH1 (p.Phe1606_Lys1607insPheSer)	4	0	30	branching (2)	4	1	3	2	0

1 time point

T-ALL	adult	UPN015												
T-ALL	adult	UPN015												
T-ALL	adult	UPN015												
T-ALL	adult	UPN015												
T-ALL	adult	UPN015												
T-ALL	adult	UPN015												
T-ALL	adult	UPN015												
T-ALL	adult	UPN015												
T-ALL	adult	UPN015	1	65312365	A	G	1280	1	1281	0.08 %	1359	84	1499	5.60 %
T-ALL	adult	UPN015	1	65312365	A	C	1280	0	1281	0.00 %	1359	55	1499	3.67 %
T-ALL	adult	UPN015	19	17949108	C	T	1215	0	1218	0.00 %	1384	41	1429	2.87 %
T-ALL	adult	UPN016												
T-ALL	adult	UPN016												
T-ALL	adult	UPN016	X	133559248	A	G	455	0	457	0.00 %	355	122	478	25.52 %
T-ALL	adult	UPN016	1	115258748	C	T	1377	0	1378	0.00 %	1273	200	1477	13.54 %
T-ALL	adult	UPN016	9	139390648	CAG	C	1492	0	1500	0.00 %	1193	142	1343	10.57 %
T-ALL	adult	UPN016	4	153247366	C	T	995	0	997	0.00 %	1033	113	1147	9.85 %
T-ALL	adult	UPN016	9	139397768	A	G	1569	0	1571	0.00 %	1500	159	1669	9.53 %
T-ALL	adult	UPN016	17	30267504	A	T	664	0	666	0.00 %	816	65	884	7.35 %
T-ALL	adult	UPN017												
T-ALL	adult	UPN017												
T-ALL	adult	UPN017												
T-ALL	adult	UPN017	X	133547981	CCATTAT	CGAGGAAGAA	341	2	342	0.58 %	71	231	301	76.74 %
T-ALL	adult	UPN017												
T-ALL	adult	UPN017												
T-ALL	adult	UPN017	19	10906072	C	T	1569	0	1571	0.00 %	1477	298	1778	16.76 %
T-ALL	adult	UPN017	9	139390863	AC	AGGCCAGATTTTA	1576	4	1578	0.25 %	1390	215	1613	13.33 %
T-ALL	adult	UPN017	9	139399365	A	G	1766	0	1770	0.00 %	1647	239	1889	12.65 %
T-ALL	adult	UPN017	9	139390887	CTCAGGAAGCT	CGGTGCTAAACCTGATAGA	1590	8	1593	0.50 %	1322	194	1556	12.47 %
T-ALL	adult	UPN017	9	139399325	G	GCTAAAA	1824	0	1825	0.00 %	1925	221	1925	11.48 %

1 time point

T-ALL	adult	UPN015	6	73751334	86282090	1	not detected	93.68 %	93.03 %	94.32 %	314	CNV
T-ALL	adult	UPN015	8	18277912	21662646	1	not detected	93.48 %	92.37 %	94.59 %	231	CNV
T-ALL	adult	UPN015	11	67101158	73686592	1	not detected	93.36 %	92.70 %	94.01 %	271	CNV
T-ALL	adult	UPN015	11	83992418	116101801	1	not detected	92.16 %	91.45 %	92.86 %	1203	CNV
T-ALL	adult	UPN015	12	7652308	29663330	1	not detected	93.88 %	93.60 %	94.16 %	1033	CNV
T-ALL	adult	UPN015	13	33003696	63644720	1	not detected	93.81 %	93.51 %	94.11 %	1078	CNV
T-ALL	adult	UPN015	17	26145406	26938175	1	not detected	94.05 %	92.87 %	95.24 %	21	CNV
T-ALL	adult	UPN015	1	3249242	7314630	1	not detected	85.82 %	85.35 %	86.28 %	247	CNV
T-ALL	adult	UPN015										SNV/Indel
T-ALL	adult	UPN015										SNV/Indel
T-ALL	adult	UPN015										SNV/Indel
T-ALL	adult	UPN016	9	133828	21780037	3	not detected	63.88 %	62.27 %	65.49 %	1194	CNV
T-ALL	adult	UPN016	9	28382826	32252919	3	not detected	65.91 %	61.46 %	70.35 %	147	CNV
T-ALL	adult	UPN016										SNV/Indel
T-ALL	adult	UPN016										SNV/Indel
T-ALL	adult	UPN016										SNV/Indel
T-ALL	adult	UPN016										SNV/Indel
T-ALL	adult	UPN016										SNV/Indel
T-ALL	adult	UPN016										SNV/Indel
T-ALL	adult	UPN017	9	20350021	22713304	1	not detected	72.91 %	67.94 %	77.88 %	133	CNV
T-ALL	adult	UPN017	11	117417973	118401125	1	not detected	87.66 %	86.26 %	89.07 %	55	CNV
T-ALL	adult	UPN017	15	98982627	101551136	3	not detected	72.37 %	67.02 %	77.72 %	189	CNV
T-ALL	adult	UPN017										SNV/Indel
T-ALL	adult	UPN017	9	133370377	135424112	1	not detected	58.23 %	52.87 %	63.59 %	76	CNV
T-ALL	adult	UPN017	8	0	146364022	3	not detected	51.14 %	48.43 %	53.85 %	4468	CNV
T-ALL	adult	UPN017										SNV/Indel
T-ALL	adult	UPN017										SNV/Indel
T-ALL	adult	UPN017										SNV/Indel
T-ALL	adult	UPN017										SNV/Indel
T-ALL	adult	UPN017										SNV/Indel

T-ALL	adult	UPN017	NOTCH1 (p.Arg1598Pro)	5	0	10	branching (2)	4	1	3	2	1
T-ALL	adult	UPN018	del in 2q	1	0	75	linear	1	0	0	1	1
T-ALL	adult	UPN018	del in 2q	1	0	75	linear	1	0	0	1	1
T-ALL	adult	UPN018	del12p	1	0	75	linear	1	0	0	1	1
T-ALL	adult	UPN018	NOTCH1 (p.Leu1593Pro)	1	0	75	linear	1	0	0	2	1
T-ALL	adult	UPN019	del in 19p	1	0	90	linear	1	0	0	1	1
T-ALL	adult	UPN019	PHF6 (p.Ile315Thr)	1	0	90	linear	1	0	0	2	0
T-ALL	adult	UPN019	LOH in 11p	2	0	79	linear	2	0	1	2	2
T-ALL	adult	UPN019	del in 12p	3	0	65	linear	3	0	2	1	1
T-ALL	adult	UPN019	NOTCH1 (p.Leu1574Pro)	3	0	65	linear	3	0	2	2	1
T-ALL	adult	UPN019	DNM2 (p.Arg770*)	3	0	65	linear	3	0	2	2	1
T-ALL	adult	UPN019	FBXW7 (p.Arg479Gln)	3	0	65	linear	3	0	2	2	1
T-ALL	adult	UPN019	BCL11B (p.Arg472His)	3	0	65	linear	3	0	2	2	1
T-ALL	adult	UPN019	del in 9p	4	0	52	linear	4	0	3	1	1
T-ALL	adult	UPN020	LOH in 4q	1	0	94	linear	1	0	0	2	2
T-ALL	adult	UPN020	LOH9p	1	0	94	linear	1	0	0	2	2
T-ALL	adult	UPN020	FBXW7 (p.Arg689Trp)	1	0	94	linear	1	0	0	2	0
T-ALL	adult	UPN020	BCL11B (p.Gln848*)	1	0	94	linear	1	0	0	2	1
T-ALL	adult	UPN020	NOTCH1 (p.Leu1678Pro)	2	0	83	linear	2	0	1	2	1
T-ALL	adult	UPN020	PHF6 (c.138_138+1insA))	2	0	83	linear	2	0	1	2	1
T-ALL	adult	UPN020	CCND3 (p.Leu255fs)	2	0	83	linear	2	0	1	2	1
T-ALL	adult	UPN020	USP7 (p.Asp483fs)	3	0	60	linear	3	0	2	2	1
T-ALL	adult	UPN021	MYB (p.Asn68Ile)	1	0	41	linear	1	0	0	2	1
T-ALL	adult	UPN021	del in 12q	2	0	28	linear	2	0	1	1	1
T-ALL	adult	UPN021	SUZ12 (p.Met137Leu)	3	0	7	linear	3	0	2	2	1
T-ALL	adult	UPN021	IL7R (p.SerValAlaLeu252SerLeuGlyMet))	3	0	7	linear	3	0	2	2	1
T-ALL	adult	UPN021	IL7R (p.SerValAlaLeu252SerArgGlyVal))	4	0	4	linear	4	0	3	2	1
T-ALL	adult	UPN022	LOH9p	1	0	84	linear	1	0	0	2	2
T-ALL	adult	UPN022	LOH11	1	0	84	linear	1	0	0	2	2
T-ALL	adult	UPN022	dup in 1p	2	0	68	linear	2	0	1	3	3

T-ALL	adult	UPN017	9	139399350	C	G	1817	1	1819	0.05 %	1788	99	1892	5.23 %
T-ALL	adult	UPN018												
T-ALL	adult	UPN018												
T-ALL	adult	UPN018												
T-ALL	adult	UPN018	9	139399365	A	G	2019	1	2023	0.05 %	925	515	1444	35.66 %
T-ALL	adult	UPN019												
T-ALL	adult	UPN019	X	133551305	T	C	792	1	794	0.13 %	43	399	442	90.27 %
T-ALL	adult	UPN019												
T-ALL	adult	UPN019												
T-ALL	adult	UPN019	9	139399422	A	G	2489	1	2491	0.04 %	988	540	1529	35.32 %
T-ALL	adult	UPN019	19	10940819	C	T	1475	0	1480	0.00 %	521	258	781	33.03 %
T-ALL	adult	UPN019	4	153247366	C	T	1513	0	1515	0.00 %	665	302	969	31.17 %
T-ALL	adult	UPN019	14	99641758	C	T	1259	0	1262	0.00 %	694	313	1008	31.05 %
T-ALL	adult	UPN019												
T-ALL	adult	UPN020												
T-ALL	adult	UPN020												
T-ALL	adult	UPN020	4	153244092	G	A	1226	0	1231	0.00 %	121	1201	1327	90.50 %
T-ALL	adult	UPN020	14	99640631	G	A	1397	0	1399	0.00 %	752	704	1458	48.29 %
T-ALL	adult	UPN020	9	139397768	A	G	1438	1	1444	0.07 %	897	704	1603	43.92 %
T-ALL	adult	UPN020	X	133511785	G	GA	754	0	755	0.00 %	1005	428	1014	42.21 %
T-ALL	adult	UPN020	6	41903793	A	AGGCT	1618	0	1623	0.00 %	1591	637	1594	39.96 %
T-ALL	adult	UPN020	16	8999170	CG	CAGGTAGGCCCGTATA	1105	0	1101	0.00 %	655	271	934	29.01 %
T-ALL	adult	UPN021	6	135509033	A	T	1106	2	1110	0.18 %	733	190	923	20.59 %
T-ALL	adult	UPN021												
T-ALL	adult	UPN021	17	30274658	A	T	910	1	913	0.11 %	656	32	689	4.64 %
T-ALL	adult	UPN021	5	35874599	TTCTCTGTC	CCCTGGGAA	1501	0	1501	0.00 %	1170	31	1232	2.52 %
T-ALL	adult	UPN021	5	35874599	TTCTCTGTC	CCCGGGGAG	1501	0	1501	0.00 %	1170	26	1232	2.11 %
T-ALL	adult	UPN022												
T-ALL	adult	UPN022												
T-ALL	adult	UPN022												

T-ALL	adult	UPN017											SNV/Indel
T-ALL	adult	UPN018	2	144191260	146494132	1	not detected		77.86 %	76.24 %	79.48 %	59	CNV
T-ALL	adult	UPN018	2	223064508	224726901	1	not detected		76.96 %	75.85 %	78.08 %	75	CNV
T-ALL	adult	UPN018	12	0	35800000	1	not detected		75.98 %	75.69 %	76.27 %	1807	CNV
T-ALL	adult	UPN018											SNV/Indel
T-ALL	adult	UPN019	19	903017	2365113	1	not detected		91.86 %	90.15 %	93.58 %	77	CNV
T-ALL	adult	UPN019											SNV/Indel
T-ALL	adult	UPN019	11	0	49700687	2	not detected		79.05 %	78.77 %	79.32 %	1302	CNV
T-ALL	adult	UPN019	12	12703002	16900433	1	not detected		64.25 %	63.43 %	65.07 %	205	CNV
T-ALL	adult	UPN019											SNV/Indel
T-ALL	adult	UPN019											SNV/Indel
T-ALL	adult	UPN019											SNV/Indel
T-ALL	adult	UPN019											SNV/Indel
T-ALL	adult	UPN019	9	20356117	22863791	1	not detected		52.40 %	45.96 %	58.84 %	123	CNV
T-ALL	adult	UPN020	4	147200455	191154276	2	not detected		96.44 %	96.33 %	96.56 %	1831	CNV
T-ALL	adult	UPN020	9	0	38039604	2	not detected		96.75 %	96.55 %	96.96 %	1677	CNV
T-ALL	adult	UPN020											SNV/Indel
T-ALL	adult	UPN020											SNV/Indel
T-ALL	adult	UPN020											SNV/Indel
T-ALL	adult	UPN020											SNV/Indel
T-ALL	adult	UPN020											SNV/Indel
T-ALL	adult	UPN020											SNV/Indel
T-ALL	adult	UPN021											SNV/Indel
T-ALL	adult	UPN021	12	94233225	133883364	1	not detected		28.13 %	27.59 %	28.07 %	1947	CNV
T-ALL	adult	UPN021											SNV/Indel
T-ALL	adult	UPN021											SNV/Indel
T-ALL	adult	UPN021											SNV/Indel
T-ALL	adult	UPN022	9	0	37574451	2	not detected		84.62 %	84.22 %	85.01 %	1927	CNV
T-ALL	adult	UPN022	11	0	135006516	2	not detected		85.46 %	85.19 %	85.72 %	5637	CNV
T-ALL	adult	UPN022	1	33839493	41197538	3	not detected		68.71 %	64.52 %	72.90 %	282	CNV

T-ALL	adult	UPN022	dup in 2q	2	0	68	linear	2	0	1	3	3
T-ALL	adult	UPN022	dup in 2q	2	0	68	linear	2	0	1	3	3
T-ALL	adult	UPN022	dup in 12p	2	0	68	linear	2	0	1	3	3
T-ALL	adult	UPN022	PHF6 (p.Cys213Tyr)	2	0	68	linear	2	0	1	2	0
T-ALL	adult	UPN022	SETD1B (p.Tyr1873*)	2	0	68	linear	2	0	1	2	1
T-ALL	adult	UPN022	NOTCH1 (p.Leu1678Pro)	2	0	68	linear	2	0	1	2	1
T-ALL	adult	UPN022	SUZ12 (p.Arg196fs)	3	0	48	linear	3	0	2	2	1
T-ALL	adult	UPN023	del in 9p	1	0	88	linear	1	0	0	1	1
T-ALL	adult	UPN023	del in 12p	1	0	88	linear	1	0	0	1	1
T-ALL	adult	UPN023	CNOT3 (p.Glu730Lys)	1	0	88	linear	1	0	0	2	0.5
T-ALL	adult	UPN023	CNOT3 (p.Ile84Phe)	1	0	88	linear	1	0	0	2	0.5
T-ALL	adult	UPN023	NOTCH1 (p.Ser2449fs)	2	0	80	linear	2	0	1	2	0.5
T-ALL	adult	UPN023	FBXW7 (p.Gln631*)	2	0	80	linear	2	0	1	2	1
T-ALL	adult	UPN023	IKZF1 (p.Arg213*)	2	0	80	linear	2	0	1	2	1
T-ALL	adult	UPN023	NOTCH1 (p.Phe1592delinsLeuHis)	2	0	80	linear	2	0	1	2	0.5
T-ALL	adult	UPN024	del in 17q	1	0	91	linear	1	0	0	1	1
T-ALL	adult	UPN024	STAT5B (p.Ala766Val)	1	0	91	linear	1	0	0	2	1
T-ALL	adult	UPN024	dup7	2	0	82	linear	2	0	1	3	3
T-ALL	adult	UPN024	DDX3X (p.Pro167Ser)	3	0	60	linear	3	0	2	2	1
T-ALL	adult	UPN024	CCND3 (p.Pro265_Ser275del)	4	0	43	linear	4	0	3	2	1
T-ALL	adult	UPN025	del in 5q	1	0	92	linear	1	0	0	1	1
T-ALL	adult	UPN025	del in 9q	1	0	92	linear	1	0	0	1	1
T-ALL	adult	UPN025	del in 16p	1	0	92	linear	1	0	0	1	1
T-ALL	adult	UPN025	DNM2 (p.Lys562del)	2	0	85	linear	2	0	1	3	1
T-ALL	adult	UPN025	BCL11B (p.Thr450Met)	2	0	85	linear	2	0	1	2	1
T-ALL	adult	UPN025	NOTCH1 (p.Pro2448fs)	2	0	85	linear	2	0	1	2	0.5
T-ALL	adult	UPN025	EZH2 (p.Pro531Ser)	2	0	85	linear	2	0	1	2	1
T-ALL	adult	UPN025	NOTCH1 (p.Glu1719_Ala1720del)	2	0	85	linear	2	0	1	2	0.5
T-ALL	adult	UPN025	dup in 19p	3	0	72	linear	3	0	2	3	3
T-ALL	adult	UPN025	JAK1 (p.Tyr652Asn)	4	0	8	linear	4	0	3	2	1
T-ALL	adult	UPN026	del in 1q	1	0	84	linear	1	0	0	1	1

1 time point

Supplemental Data 3

T-ALL	adult	UPN022												
T-ALL	adult	UPN022												
T-ALL	adult	UPN022												
T-ALL	adult	UPN022	X	133547902	G	A	456	0	456	0.00 %	91	210	303	69.31 %
T-ALL	adult	UPN022	12	122265868	T	G	1970	3	1975	0.15 %	783	394	1180	33.39 %
T-ALL	adult	UPN022	9	139397768	A	G	1825	1	1828	0.05 %	649	313	966	32.40 %
T-ALL	adult	UPN022	17	30300238	C	CA	678	0	682	0.00 %	444	108	445	24.27 %
T-ALL	adult	UPN023												
T-ALL	adult	UPN023												
T-ALL	adult	UPN023	19	54659071	G	A	3110	0	3120	0.00 %	875	686	1568	43.75 %
T-ALL	adult	UPN023	19	54647477	A	T	2372	4	2381	0.17 %	625	475	1102	43.10 %
T-ALL	adult	UPN023	9	139390846	TG	T	3219	2	3232	0.06 %	823	622	1449	42.93 %
T-ALL	adult	UPN023	4	153244266	G	A	1945	0	1947	0.00 %	742	539	1285	41.95 %
T-ALL	adult	UPN023	7	50455090	C	T	2207	0	2212	0.00 %	706	493	1203	40.98 %
T-ALL	adult	UPN023	9	139399367	G	GTGT	3708	0	3718	0.00 %	1471	571	1477	38.66 %
T-ALL	adult	UPN024												
T-ALL	adult	UPN024	17	40353823	G	A	1091	0	1092	0.00 %	603	506	1110	45.59 %
T-ALL	adult	UPN024												
T-ALL	adult	UPN024	X	41202045	C	T	1020	0	1023	0.00 %	955	412	1369	30.09 %
T-ALL	adult	UPN024	6	41903730	TGGCTGCTGGAGCCCCGGGGGGCTTTGGGCGCTG	T	1405	0	1407	0.00 %	1114	311	1426	21.81 %
T-ALL	adult	UPN025												
T-ALL	adult	UPN025												
T-ALL	adult	UPN025												
T-ALL	adult	UPN025	19	10930662	GAGA	G	1076	0	1076	0.00 %	880	1237	2119	58.38 %
T-ALL	adult	UPN025	14	99641824	G	A	861	0	862	0.00 %	827	670	1499	44.70 %
T-ALL	adult	UPN025	9	139390849	GG	TGAT	1110	11	1117	0.98 %	1102	880	1990	44.22 %
T-ALL	adult	UPN025	7	148512087	G	A	916	0	917	0.00 %	914	718	1637	43.86 %
T-ALL	adult	UPN025	9	139397639	ACGGCCT	A	1004	0	1004	0.00 %	971	675	1649	40.93 %
T-ALL	adult	UPN025												
T-ALL	adult	UPN025	1	65312365	A	T	1049	1	1052	0.10 %	1794	78	1874	4.16 %
T-ALL	adult	UPN026												

1 time point

Supplemental Data 3

T-ALL	adult	UPN022	2	113275957	114330596	3	not detected	67.62 %	56.53 %	78.71 %	54	CNV
T-ALL	adult	UPN022	2	119507392	191335186	3	not detected	63.29 %	61.97 %	64.62 %	2404	CNV
T-ALL	adult	UPN022	12	45654233	94721983	3	not detected	70.52 %	68.73 %	72.31 %	1467	CNV
T-ALL	adult	UPN022										SNV/Indel
T-ALL	adult	UPN022										SNV/Indel
T-ALL	adult	UPN022										SNV/Indel
T-ALL	adult	UPN022										SNV/Indel
T-ALL	adult	UPN023	9	0	37180697	1	not detected	90.09 %	89.80 %	90.37 %	2066	CNV
T-ALL	adult	UPN023	12	10841091	13547719	1	not detected	89.96 %	88.52 %	91.41 %	129	CNV
T-ALL	adult	UPN023										SNV/Indel
T-ALL	adult	UPN023										SNV/Indel
T-ALL	adult	UPN023										SNV/Indel
T-ALL	adult	UPN023										SNV/Indel
T-ALL	adult	UPN023										SNV/Indel
T-ALL	adult	UPN023										SNV/Indel
T-ALL	adult	UPN024	17	28912879	30415430	1	not detected	88.19 %	83.71 %	92.66 %	29	CNV
T-ALL	adult	UPN024										SNV/Indel
T-ALL	adult	UPN024	7	0	159124173	3	not detected	81.99 %	80.77 %	83.22 %	5916	CNV
T-ALL	adult	UPN024										SNV/Indel
T-ALL	adult	UPN024										SNV/Indel
T-ALL	adult	UPN025	5	103919508	104286271	1	not detected	96.79 %	93.95 %	99.64 %	7	CNV
T-ALL	adult	UPN025	9	131462039	134027165	1	not detected	91.95 %	91.13 %	92.76 %	126	CNV
T-ALL	adult	UPN025	16	0	2249595	1	not detected	91.70 %	90.69 %	92.71 %	137	CNV
T-ALL	adult	UPN025										SNV/Indel
T-ALL	adult	UPN025										SNV/Indel
T-ALL	adult	UPN025										SNV/Indel
T-ALL	adult	UPN025										SNV/Indel
T-ALL	adult	UPN025										SNV/Indel
T-ALL	adult	UPN025	19	0	14890476	3	not detected	72.57 %	70.20 %	74.94 %	804	CNV
T-ALL	adult	UPN025										SNV/Indel
T-ALL	adult	UPN026	1	199129328	199996598	1	not detected	76.36 %	67.80 %	84.92 %	28	CNV

1 time point

T-ALL	adult	UPN026	del in 6q	1	0	84	linear	1	0	0	1	1
T-ALL	adult	UPN026	del in 9p	1	0	84	linear	1	0	0	1	1
T-ALL	adult	UPN026	del in 13q	1	0	84	linear	1	0	0	1	1
T-ALL	adult	UPN026	NOTCH1 (p.Ile1616Asn)	1	0	84	linear	1	0	0	2	1
T-ALL	adult	UPN026	PHF6 (p.Ser257*)	1	0	84	linear	1	0	0	2	1
T-ALL	adult	UPN026	MYB (p.His266Tyr)	1	0	84	linear	1	0	0	2	1
T-ALL	adult	UPN026	FBXW7 (p.Arg465Pro)	1	0	84	linear	1	0	0	2	1
T-ALL	adult	UPN026	DNM2 (p.Pro405His)	1	0	84	linear	1	0	0	2	1
T-ALL	adult	UPN026	NOTCH1 (p.Ser2471fs)	2	0	63	linear	2	0	1	2	0.5
T-ALL	adult	UPN026	RUNX1 (p.Val164_Gly165insValPro)	2	0	63	linear	2	0	1	2	1
T-ALL	adult	UPN027	LOH in 8q	1	0	93	linear	1	0	0	2	2
T-ALL	adult	UPN027	del in 6q	2	0	84	linear	2	0	1	1	1
T-ALL	adult	UPN027	dup21	2	0	84	linear	2	0	1	3	3
T-ALL	adult	UPN027	FBXW7 (p.Arg441Gln)	2	0	84	linear	2	0	1	2	0.5
T-ALL	adult	UPN027	NRAS (p.Ala146Val)	2	0	84	linear	2	0	1	2	1
T-ALL	adult	UPN027	FBXW7 (p.Arg479Gln)	2	0	84	linear	2	0	1	2	0.5
T-ALL	adult	UPN027	dup in 19p	3	0	76	linear	3	0	2	3	3
T-ALL	adult	UPN027	NOTCH1 (p.Pro1582_Glu1583insValCys)	3	0	76	linear	3	0	2	2	1
T-ALL	adult	UPN027	PHF6 (p.His131fs)	4	0	49	linear	4	0	3	2	1
T-ALL	adult	UPN028	LOH in 1p	1	0	92	linear	1	0	0	2	2
T-ALL	adult	UPN028	LOH in 6q	1	0	92	linear	1	0	0	2	2
T-ALL	adult	UPN028	LOH in 15q	1	0	92	linear	1	0	0	2	2
T-ALL	adult	UPN028	del in 17q	1	0	92	linear	1	0	0	1	1
T-ALL	adult	UPN028	JAK1 (p.Ser703Ile)	1	0	92	linear	1	0	0	2	0
T-ALL	adult	UPN028	RUNX1 (p.Tyr385*)	1	0	92	linear	1	0	0	2	1
T-ALL	adult	UPN028	USP7 (p.Leu95fs)	2	0	69	linear	2	0	1	2	1
T-ALL	adult	UPN028	NOTCH1 (p.Ile1616_Phe1617insGlnAsnProGln)	2	0	69	linear	2	0	1	2	1
T-ALL	adult	UPN029	NOTCH1 (p.Leu1678Pro)	1	0	38	linear	1	0	0	2	1
T-ALL	adult	UPN029	FBXW7 (p.Arg689Trp)	1	0	38	linear	1	0	0	2	1
T-ALL	adult	UPN029	SMARCA4 (p.Val1228Leu)	1	0	38	linear	1	0	0	2	1
T-ALL	adult	UPN029	del in 9p	2	0	28	linear	2	0	1	1	1

1 time point

Supplemental Data 3

T-ALL	adult	UPN026												
T-ALL	adult	UPN026												
T-ALL	adult	UPN026												
T-ALL	adult	UPN026	9	139399296	A	T	1448	0	1450	0.00 %	970	758	1732	43.76 %
T-ALL	adult	UPN026	X	133549083	C	G	898	0	901	0.00 %	656	474	1133	41.84 %
T-ALL	adult	UPN026	6	135515009	C	T	1069	0	1071	0.00 %	849	605	1462	41.38 %
T-ALL	adult	UPN026	4	153249384	C	G	1272	3	1276	0.24 %	942	665	1613	41.23 %
T-ALL	adult	UPN026	19	10908073	C	A	1272	0	1273	0.00 %	948	644	1597	40.33 %
T-ALL	adult	UPN026	9	139390778	CGAGGAT	CGGAGAGGCGAC	1355	10	1359	0.74 %	883	431	1309	32.93 %
T-ALL	adult	UPN026	21	36252868	C	CCAGGGA	1017	0	1020	0.00 %	1237	386	1273	30.32 %
T-ALL	adult	UPN027												
T-ALL	adult	UPN027												
T-ALL	adult	UPN027												
T-ALL	adult	UPN027	4	153249456	C	T	1047	1	1049	0.10 %	716	558	1279	43.63 %
T-ALL	adult	UPN027	1	115252203	G	A	1007	0	1007	0.00 %	661	496	1158	42.83 %
T-ALL	adult	UPN027	4	153247366	C	T	1007	1	1010	0.10 %	595	443	1039	42.64 %
T-ALL	adult	UPN027												
T-ALL	adult	UPN027	9	139399395	T	TCACACA	1495	0	1500	0.00 %	1681	654	1688	38.74 %
T-ALL	adult	UPN027	X	133527956	ACAAGAAAAC	GGAAAGGGGAGG	815	6	821	0.73 %	588	201	814	24.69 %
T-ALL	adult	UPN028												
T-ALL	adult	UPN028												
T-ALL	adult	UPN028												
T-ALL	adult	UPN028												
T-ALL	adult	UPN028	1	65311203	C	A	1398	1	1403	0.07 %	111	1070	1185	90.30 %
T-ALL	adult	UPN028	21	36164720	G	T	1691	0	1693	0.00 %	992	808	1803	44.81 %
T-ALL	adult	UPN028	16	9017172	G	GATCCT	1563	0	1565	0.00 %	1344	487	1344	36.24 %
T-ALL	adult	UPN028	9	139399294	A	ATTGGGGGTTCTG	1559	0	1574	0.00 %	1477	499	1503	33.20 %
T-ALL	adult	UPN029	9	139397768	A	G	1214	1	1216	0.08 %	1232	320	1559	20.53 %
T-ALL	adult	UPN029	4	153244092	G	A	1074	0	1076	0.00 %	1139	248	1390	17.84 %
T-ALL	adult	UPN029	19	11144101	G	C	1239	0	1242	0.00 %	1340	278	1621	17.15 %
T-ALL	adult	UPN029												

1 time point

T-ALL	adult	UPN026	6	72835062	93551709	1	not detected	84.45 %	84.09 %	84.80 %	683	CNV
T-ALL	adult	UPN026	9	21184923	22648917	1	not detected	74.79 %	69.13 %	80.45 %	69	CNV
T-ALL	adult	UPN026	13	69164068	69460238	1	not detected	82.20 %	79.68 %	84.72 %	13	CNV
T-ALL	adult	UPN026										SNV/Indel
T-ALL	adult	UPN026										SNV/Indel
T-ALL	adult	UPN026										SNV/Indel
T-ALL	adult	UPN026										SNV/Indel
T-ALL	adult	UPN026										SNV/Indel
T-ALL	adult	UPN026										SNV/Indel
T-ALL	adult	UPN026										SNV/Indel
T-ALL	adult	UPN027	8	108814632	146364022	2	not detected	93.05 %	92.55 %	93.55 %	1491	CNV
T-ALL	adult	UPN027	6	75519783	109056448	1	not detected	84.81 %	84.06 %	85.56 %	1008	CNV
T-ALL	adult	UPN027	21	0	48129895	3	not detected	83.03 %	81.48 %	84.57 %	1651	CNV
T-ALL	adult	UPN027										SNV/Indel
T-ALL	adult	UPN027										SNV/Indel
T-ALL	adult	UPN027										SNV/Indel
T-ALL	adult	UPN027	19	8616347	18296566	3	not detected	79.88 %	76.95 %	82.81 %	491	CNV
T-ALL	adult	UPN027										SNV/Indel
T-ALL	adult	UPN027										SNV/Indel
T-ALL	adult	UPN028	1	0	109266813	2	not detected	95.54 %	95.44 %	95.64 %	3953	CNV
T-ALL	adult	UPN028	6	73980933	171115067	2	not detected	95.66 %	95.51 %	95.82 %	3481	CNV
T-ALL	adult	UPN028	15	40751801	102531392	2	not detected	95.31 %	95.10 %	95.51 %	2729	CNV
T-ALL	adult	UPN028	17	28912879	30415430	1	not detected	90.76 %	87.93 %	93.58 %	27	CNV
T-ALL	adult	UPN028										SNV/Indel
T-ALL	adult	UPN028										SNV/Indel
T-ALL	adult	UPN028										SNV/Indel
T-ALL	adult	UPN028										SNV/Indel
T-ALL	adult	UPN029										SNV/Indel
T-ALL	adult	UPN029										SNV/Indel
T-ALL	adult	UPN029										SNV/Indel
T-ALL	adult	UPN029	9	19499535	38825011	1	not detected	27.60 %	26.55 %	28.64 %	687	CNV

T-ALL	adult	UPN030	del6	1	0	86	linear	1	0	0	1	1
T-ALL	adult	UPN030	del in 17p	1	0	86	linear	1	0	0	1	1
T-ALL	adult	UPN030	del in 13q	1	0	86	linear	1	0	0	1	1
T-ALL	adult	UPN030	del in 13q	1	0	86	linear	1	0	0	1	1
T-ALL	adult	UPN030	dup in 13q coming from del	1	0	86	linear	1	0	0	3	3
T-ALL	adult	UPN030	dup in 13q coming from del	1	0	86	linear	1	0	0	3	3
T-ALL	adult	UPN030	TP53 (p.His193Leu)	1	0	86	linear	1	0	0	1	0
T-ALL	adult	UPN030	dup1q	2	0	65	linear	2	0	1	3	3
T-ALL	adult	UPN030	dup2	2	0	65	linear	2	0	1	3	3
T-ALL	adult	UPN030	dup3	2	0	65	linear	2	0	1	3	3
T-ALL	adult	UPN030	dup4	2	0	65	linear	2	0	1	3	3
T-ALL	adult	UPN030	dup5	2	0	65	linear	2	0	1	3	3
T-ALL	adult	UPN030	dup7	2	0	65	linear	2	0	1	3	3
T-ALL	adult	UPN030	dup11	2	0	65	linear	2	0	1	3	3
T-ALL	adult	UPN030	dup14	2	0	65	linear	2	0	1	3	3
T-ALL	adult	UPN030	del15	2	0	65	linear	2	0	1	1	1
T-ALL	adult	UPN030	dup16	2	0	65	linear	2	0	1	3	3
T-ALL	adult	UPN030	dup18	2	0	65	linear	2	0	1	3	3
T-ALL	adult	UPN030	dup in 13q	2	0	65	linear	2	0	1	3	3
T-ALL	adult	UPN030	EZH2 (p.Gly660Val)	2	0	62	linear	2	0	1	3	1
T-ALL	adult	UPN030	dup8	3	0	50	linear	3	0	2	3	3
T-ALL	adult	UPN030	dup9	3	0	50	linear	3	0	2	3	3
T-ALL	adult	UPN030	dup19	3	0	50	linear	3	0	2	3	3
T-ALL	adult	UPN030	dupX	3	0	50	linear	3	0	2	3	3
T-ALL	adult	UPN030	PHF6 (p.Arg116*)	3	0	50	linear	3	0	2	3	1
T-ALL	adult	UPN030	NOTCH1 (p.Ala1700Asp)	3	0	50	linear	3	0	2	3	1
T-ALL	adult	UPN030	KDM6A (p.Val1120fs)	3	0	50	linear	3	0	2	3	1
T-ALL	adult	UPN031	del in 1p	1	0	60	linear	1	0	0	1	1
T-ALL	adult	UPN031	del in 9p	1	0	60	linear	1	0	0	1	1
T-ALL	adult	UPN031	PHF6 (p.Gly187*)	1	0	60	linear	1	0	0	2	1
T-ALL	adult	UPN031	del in 5q	2	0	50	linear	2	0	1	1	1
T-ALL	adult	UPN031	del in 12p	2	0	50	linear	2	0	1	1	1
T-ALL	adult	UPN031	NOTCH1 (p.Phe1606_Lys1607insAspPro)	2	0	50	linear	2	0	1	2	1

1 time point

Supplemental Data 3

T-ALL	adult	UPN030												
T-ALL	adult	UPN030												
T-ALL	adult	UPN030												
T-ALL	adult	UPN030												
T-ALL	adult	UPN030												
T-ALL	adult	UPN030												
T-ALL	adult	UPN030	17	7578271	T	A	1296	0	1298	0.00 %	316	1957	2280	85.83 %
T-ALL	adult	UPN030												
T-ALL	adult	UPN030												
T-ALL	adult	UPN030												
T-ALL	adult	UPN030												
T-ALL	adult	UPN030												
T-ALL	adult	UPN030												
T-ALL	adult	UPN030												
T-ALL	adult	UPN030												
T-ALL	adult	UPN030												
T-ALL	adult	UPN030												
T-ALL	adult	UPN030												
T-ALL	adult	UPN030												
T-ALL	adult	UPN030	7	148507475	C	A	913	0	915	0.00 %	477	475	952	49.89 %
T-ALL	adult	UPN030												
T-ALL	adult	UPN030												
T-ALL	adult	UPN030												
T-ALL	adult	UPN030												
T-ALL	adult	UPN030	X	133527636	C	T	551	1	552	0.18 %	285	242	530	45.66 %
T-ALL	adult	UPN030	9	139397702	G	T	1406	2	1409	0.14 %	2157	1328	3492	38.03 %
T-ALL	adult	UPN030	X	44942755	TCG	TCCC	604	5	605	0.83 %	583	259	845	30.65 %
T-ALL	adult	UPN031												
T-ALL	adult	UPN031												
T-ALL	adult	UPN031	X	133547658	G	T	656	0	657	0.00 %	727	306	1036	29.54 %
T-ALL	adult	UPN031												
T-ALL	adult	UPN031												
T-ALL	adult	UPN031	9	139399324	T	TGGGATC	1407	0	1411	0.00 %	1789	418	1799	23.24 %

1 time point

Supplemental Data 3

T-ALL	adult	UPN030	6	0	171115067	1	not detected	88.41 %	88.13 %	88.68 %	9315	CNV
T-ALL	adult	UPN030	17	0	16210580	1	not detected	91.73 %	91.07 %	92.39 %	1016	CNV
T-ALL	adult	UPN030	13	19403621	88255179	1	not detected	79.51 %	78.73 %	80.29 %	3047	CNV
T-ALL	adult	UPN030	13	104111901	112023123	1	not detected	86.55 %	85.40 %	87.69 %	566	CNV
T-ALL	adult	UPN030	13	104428282	107032732	3	not detected	86.00 %				CNV
T-ALL	adult	UPN030	13	109130370	112023123	3	not detected	86.00 %				CNV
T-ALL	adult	UPN030										SNV/Indel
T-ALL	adult	UPN030	1	125000000	249250621	3	not detected	65.18 %	63.66 %	66.70 %	4635	CNV
T-ALL	adult	UPN030	2	0	243199373	3	not detected	65.99 %	65.02 %	66.96 %	10330	CNV
T-ALL	adult	UPN030	3	0	198022430	3	not detected	56.75 %	55.55 %	57.95 %	8221	CNV
T-ALL	adult	UPN030	4	0	191154276	3	not detected	56.25 %	55.06 %	57.44 %	8066	CNV
T-ALL	adult	UPN030	5	0	180915260	3	not detected	59.30 %	58.08 %	60.53 %	7386	CNV
T-ALL	adult	UPN030	7	0	159138663	3	not detected	65.44 %	64.21 %	66.66 %	7057	CNV
T-ALL	adult	UPN030	11	0	135006516	3	not detected	64.84 %	63.55 %	66.13 %	6306	CNV
T-ALL	adult	UPN030	14	0	107349540	3	not detected	61.76 %	60.07 %	63.44 %	3975	CNV
T-ALL	adult	UPN030	15	0	102531392	1	not detected	60.12 %	59.48 %	60.77 %	4039	CNV
T-ALL	adult	UPN030	16	0	90354753	3	not detected	64.33 %	62.73 %	65.93 %	4112	CNV
T-ALL	adult	UPN030	18	0	78077248	3	not detected	67.46 %	65.86 %	69.06 %	3694	CNV
T-ALL	adult	UPN030	13	112023124	115169878	3	not detected	59.26 %	52.98 %	65.54 %	201	CNV
T-ALL	adult	UPN030										SNV/Indel
T-ALL	adult	UPN030	8	0	146364022	3	not detected	52.90 %	51.83 %	53.98 %	6527	CNV
T-ALL	adult	UPN030	9	0	141213431	3	not detected	51.32 %	50.17 %	52.48 %	5399	CNV
T-ALL	adult	UPN030	19	0	59128983	3	not detected	55.41 %	53.54 %	57.29 %	2972	CNV
T-ALL	adult	UPN030	X	0	155270560	3	not detected	48.98 %	47.99 %	49.96 %	6802	CNV
T-ALL	adult	UPN030										SNV/Indel
T-ALL	adult	UPN030										SNV/Indel
T-ALL	adult	UPN030										SNV/Indel
T-ALL	adult	UPN031	1	104430395	119041203	1	not detected	62.67 %	61.74 %	63.61 %	604	CNV
T-ALL	adult	UPN031	9	21661861	25308645	1	not detected	55.68 %	52.52 %	58.84 %	179	CNV
T-ALL	adult	UPN031										SNV/Indel
T-ALL	adult	UPN031	5	88450292	121857340	1	not detected	50.38 %	49.78 %	50.97 %	1045	CNV
T-ALL	adult	UPN031	12	0	16633880	1	not detected	50.67 %	50.00 %	51.34 %	822	CNV
T-ALL	adult	UPN031										SNV/Indel

1 time point

Supplemental Data 3

T-ALL	adult	UPN031	IL7R (p.Leu243_Thr244insArgProArg GluValLysCysLeu)	3	0	27	linear	3	0	2	2	1
T-ALL	adult	UPN031	TP53 (p.Arg248Trp)	4	0	17	linear	4	0	3	2	1
T-ALL	adult	UPN033	del in 4q	1	0	97	linear	1	0	0	1	1
T-ALL	adult	UPN033	del in 6q	1	0	97	linear	1	0	0	1	1
T-ALL	adult	UPN033	del in 9p	1	0	97	linear	1	0	0	1	1
T-ALL	adult	UPN033	NOTCH1 (p.Glu2515fs)	2	0	88	linear	2	0	1	2	1
T-ALL	adult	UPN033	NOTCH1 (p.Val1578Gly)	3	0	59	linear	3	0	2	2	0.5
T-ALL	adult	UPN033	NOTCH1 (p.Leu1593Pro)	4	0	31	linear	4	0	3	2	0
T-ALL	adult	UPN034	del in 9p	1	0	90	linear	1	0	0	1	1
T-ALL	adult	UPN034	del in 11p	1	0	90	linear	1	0	0	1	1
T-ALL	adult	UPN034	BCL11B (p.Arg472His)	1	0	90	linear	1	0	0	2	1
T-ALL	adult	UP0N34	NOTCH1 (p.Gln2405*)	1	0	90	linear	1	0	0	2	1
T-ALL	adult	UPN034	STAT5B (p.Asn642His)	2	0	72	linear	2	0	1	3	1
T-ALL	adult	UPN034	USP7 (p.Phe264fs)	2	0	72	linear	2	0	1	2	1
T-ALL	adult	UPN034	dup in 17q	3	0	40	linear	3	0	2	3	3
T-ALL	adult	UPN034	NOTCH1 (p.Ser1597Ile)	4	0	20	linear	4	0	3	2	0.25
T-ALL	adult	UPN034	NOTCH1 (p.Cys1692Arg)	4	0	20	linear	4	0	3	2	0.25
T-ALL	adult	UPN035	del in 10q	1	0	75	linear	1	0	0	1	1
T-ALL	adult	UPN035	PTEN (p.Asn228fs)	2	0	50	linear	2	0	1	1	0
T-ALL	adult	UPN036	LOH in 6p	1	0	89	linear	1	0	0	2	2
T-ALL	adult	UPN036	del in 9p	2	0	81	linear	2	0	1	1	1
T-ALL	adult	UPN036	del in 10q	2	0	81	linear	2	0	1	1	1
T-ALL	adult	UPN036	MYCN (p.Pro44Leu)	3	0	53	linear	3	0	2	2	1
T-ALL	adult	UPN037	LOH in 9p	1	0	88	linear	1	0	0	2	2
T-ALL	adult	UPN037	USP7 (p.Ser114fs)	2	0	81	linear	2	0	1	2	1
T-ALL	adult	UPN037	FBXW7 (p.Arg465Cys)	2	0	81	linear	2	0	1	2	1
T-ALL	adult	UPN037	dup in 10p	3	0	74	linear	3	0	2	3	3
T-ALL	pediatric	UPN042	del9p	1	0	74	linear	1	0	0	1	1
T-ALL	pediatric	UPN042	del in 1p	2	0	65	linear	2	0	1	1	1
T-ALL	pediatric	UPN042	del in 1q	2	0	65	linear	2	0	1	1	1
T-ALL	pediatric	UPN042	del in 1q	2	0	65	linear	2	0	1	1	1
T-ALL	pediatric	UPN042	dup in 1q	2	0	65	linear	2	0	1	3	3

1 time point

T-ALL	adult	UPN031	5	35874570	A	ACTAAGGCCCCGTGA GGTTAAGTGC	979	0	982	0.00 %	1254	167	1256	13.30 %
T-ALL	adult	UPN031	17	7577539	G	A	1241	0	1243	0.00 %	1568	151	1725	8.75 %
T-ALL	adult	UPN033												
T-ALL	adult	UPN033												
T-ALL	adult	UPN033												
T-ALL	adult	UPN033	9	139390649	A	AT	1127	0	1137	0.00 %	1231	546	1241	44.00 %
T-ALL	adult	UPN033	9	139399410	A	C	1220	0	1221	0.00 %	1039	434	1476	29.40 %
T-ALL	adult	UPN033	9	139399365	A	G	1256	1	1257	0.08 %	1236	229	1472	15.56 %
T-ALL	adult	UPN034												
T-ALL	adult	UPN034												
T-ALL	adult	UPN034	14	99641758	C	T	680	0	684	0.00 %	521	468	992	47.18 %
T-ALL	adult	UP0N34	9	139390978	G	A	1425	0	1428	0.00 %	1009	756	1768	42.76 %
T-ALL	adult	UPN034	17	40359729	T	G	746	0	747	0.00 %	442	404	850	47.53 %
T-ALL	adult	UPN034	16	9010942	G	GGACCT	753	0	766	0.00 %	1058	371	1071	34.64 %
T-ALL	adult	UPN034												
T-ALL	adult	UPN034	9	139399353	C	A	1357	1	1360	0.07 %	1616	178	1794	9.92 %
T-ALL	adult	UPN034	9	139397727	A	G	1295	0	1298	0.00 %	1447	134	1584	8.46 %
T-ALL	adult	UPN035												
T-ALL	adult	UPN035	10	89717659	TTCAGGACCCACA	GGGATCCCTAATTCT	959	7	963	0.73 %	263	174	443	39.28 %
T-ALL	adult	UPN036												
T-ALL	adult	UPN036												
T-ALL	adult	UPN036												
T-ALL	adult	UPN036	2	16082317	C	T	1773	0	1775	0.00 %	1096	402	1500	26.80 %
T-ALL	adult	UPN037												
T-ALL	adult	UPN037	16	9017114	C	CT	1177	0	1179	0.00 %	1372	584	1373	42.53 %
T-ALL	adult	UPN037	4	153249385	G	A	1593	1	1598	0.06 %	983	672	1660	40.48 %
T-ALL	adult	UPN037												
T-ALL	pediatric	UPN042												
T-ALL	pediatric	UPN042												
T-ALL	pediatric	UPN042												
T-ALL	pediatric	UPN042												
T-ALL	pediatric	UPN042												

T-ALL	adult	UPN031											SNV/Indel
T-ALL	adult	UPN031											SNV/Indel
T-ALL	adult	UPN033	4	109038217	109277343	1	NA		97.00 %				CNV
T-ALL	adult	UPN033	6	77716141	121560345	1	NA		97.00 %				CNV
T-ALL	adult	UPN033	9	0	34093703	2	NA		97.00 %				CNV
T-ALL	adult	UPN033											SNV/Indel
T-ALL	adult	UPN033											SNV/Indel
T-ALL	adult	UPN033											SNV/Indel
T-ALL	adult	UPN034	9	5387145	22307655	1	not detected		89.38 %	89.00 %	89.76 %	949	CNV
T-ALL	adult	UPN034	11	33976330	36616843	1	not detected		88.66 %	88.06 %	89.26 %	149	CNV
T-ALL	adult	UPN034											SNV/Indel
T-ALL	adult	UP0N34											SNV/Indel
T-ALL	adult	UPN034											SNV/Indel
T-ALL	adult	UPN034											SNV/Indel
T-ALL	adult	UPN034	17	37838775	81195210	3	not detected		40.66 %	39.44 %	41.89 %	1789	CNV
T-ALL	adult	UPN034											SNV/Indel
T-ALL	adult	UPN034											SNV/Indel
T-ALL	adult	UPN035	10	89690798	90153268	1	not detected		75.79 %	73.02 %	78.57 %	76.01 %	CNV
T-ALL	adult	UPN035											SNV/Indel
T-ALL	adult	UPN036	6	0	32139689	2	not detected		89.78 %	89.62 %	89.95 %	2831	CNV
T-ALL	adult	UPN036	9	19406107	39162801	1	not detected		82.05 %	81.52 %	82.59 %	752	CNV
T-ALL	adult	UPN036	10	89510393	92967000	1	not detected		76.06 %	72.57 %	79.56 %	133	CNV
T-ALL	adult	UPN036											SNV/Indel
T-ALL	adult	UPN037	9	0	35642313	2	not detected		88.23 %	87.63 %	88.82 %	1744	CNV
T-ALL	adult	UPN037											SNV/Indel
T-ALL	adult	UPN037											SNV/Indel
T-ALL	adult	UPN037	10	0	36655857	3	not detected		74.16 %	72.69 %	75.64 %	1806	CNV
T-ALL	pediatric	UPN042	9	0	40727909	1	not detected		73.41 %	72.93 %	73.89 %	1876	CNV
T-ALL	pediatric	UPN042	1	78175328	80996752	1	not detected		66.67 %	64.03 %	69.31 %	65	CNV
T-ALL	pediatric	UPN042	1	187070611	193289136	1	not detected		66.72 %	64.28 %	69.15 %	189	CNV
T-ALL	pediatric	UPN042	1	226870403	240942448	1	not detected		67.00 %	66.33 %	67.67 %	735	CNV
T-ALL	pediatric	UPN042	1	193507297	228088833	3	not detected		66.38 %	64.94 %	67.82 %	1352	CNV

T-ALL	pediatric	UPN042	DDX3X (p.Ser131*)	2	0	65	linear	2	0	1	2	1
T-ALL	pediatric	UPN042	FBXW7 (p.Arg465Cys)	2	0	65	linear	2	0	1	2	1
T-ALL	pediatric	UPN043	del in 9p	1	0	47	linear	1	0	0	1	1
T-ALL	pediatric	UPN043	BCL11B (p.Phe73fs)	2	0	39	linear	2	0	1	2	1
T-ALL	pediatric	UPN043	NOTCH1 (p.Val1605_Phe1606insLeuGlnGly)	2	0	39	linear	2	0	1	2	1
T-ALL	pediatric	UPN043	NOTCH1 (p.Leu1574Pro)	3	0	16	linear	3	0	2	2	0
T-ALL	pediatric	UPN043	NOTCH1 (p.Leu1678Pro)	4	0	11	linear	4	0	3	2	1
T-ALL	pediatric	UPN044	NOTCH1 (p.Leu1596His)	1	0	90	linear	1	0	0	2	1
T-ALL	pediatric	UPN044	dup in 9p	2	0	17	linear	2	0	1	3	3
T-ALL	pediatric	UPN045	LOH in 9p	1	0	98	linear	1	0	0	2	2
T-ALL	pediatric	UPN045	BCL11B (p.His473Arg)	1	0	98	linear	1	0	0	2	1
T-ALL	pediatric	UPN045	NOTCH1 (p.Glu2460*)	1	0	98	linear	1	0	0	2	1
T-ALL	pediatric	UPN045	NRAS (p.Gly12Ser)	2	0	80	linear	2	0	1	2	1
T-ALL	pediatric	UPN045	PTEN (p.Phe241delinsLeuGluLysAla)	3	0	71	linear	3	0	2	2	0
T-ALL	pediatric	UPN045	PTEN (p.Leu247fs)	3	0	71	linear	3	0	2	2	0
T-ALL	pediatric	UPN046	LOH in 9p	1	0	99	linear	1	0	0	2	2
T-ALL	pediatric	UPN046	del in 10q	1	0	99	linear	1	0	0	1	1
T-ALL	pediatric	UPN046	PTEN (p.Val54fs)	2	0	73	linear	2	0	1	1	0
T-ALL	pediatric	UPN047	del6q	1	0	92	linear	1	0	0	1	1
T-ALL	pediatric	UPN047	del in 9p	1	0	92	linear	1	0	0	1	1
T-ALL	pediatric	UPN047	del in 14q	1	0	92	linear	1	0	0	1	1
T-ALL	pediatric	UPN047	dup in 2q	2	0	81	linear	2	0	1	3	3
T-ALL	pediatric	UPN047	NOTCH1 (p.Phe2509fs)	3	0	64	linear	3	0	2	2	1
T-ALL	pediatric	UPN048	del9p	1	0	95	linear	1	0	0	1	1
T-ALL	pediatric	UPN048	NOTCH1 (p.Leu1600Pro)	2	0	90	linear	2	0	1	2	1
T-ALL	pediatric	UPN048	FBXW7 (p.Arg479Leu)	2	0	76	linear	2	0	1	2	1
T-ALL	pediatric	UPN048	MYCBP2 (p.Leu55fs)	3	0	76	linear	3	0	2	2	1
T-ALL	pediatric	UPN049	del in 9p	1	0	95	linear	1	0	0	1	1
T-ALL	pediatric	UPN049	del in 9p	1	0	95	linear	1	0	0	1	1
T-ALL	pediatric	UPN049	del in 12p	1	0	95	linear	1	0	0	1	1
T-ALL	pediatric	UPN049	PTEN (p.Glu91_Asp92insGly)	2	0	65	linear	2	0	1	2	1

T-ALL	pediatric	UPN042	X	41201855	C	G	804	0	806	0.00 %	288	506	795	63.65 %
T-ALL	pediatric	UPN042	4	153249385	G	A	1545	0	1548	0.00 %	1107	581	1692	34.34 %
T-ALL	pediatric	UPN043												
T-ALL	pediatric	UPN043	14	99724015	TA	T	1825	0	1831	0.00 %	1233	311	1548	20.09 %
T-ALL	pediatric	UPN043	9	139399325	G	GAACCCCTGT	2023	0	2029	0.00 %	1693	324	1706	18.99 %
T-ALL	pediatric	UPN043	9	139399422	A	G	1954	2	1961	0.10 %	1672	150	1827	8.21 %
T-ALL	pediatric	UPN043	9	139397768	A	G	1873	0	1879	0.00 %	1654	95	1756	5.41 %
T-ALL	pediatric	UPN044	9	139399356	A	T	2377	3	2382	0.13 %	1148	947	2097	45.16 %
T-ALL	pediatric	UPN044												
T-ALL	pediatric	UPN045												
T-ALL	pediatric	UPN045	14	99641755	T	C	1159	0	1164	0.00 %	480	471	954	49.37 %
T-ALL	pediatric	UPN045	9	139390813	C	A	2154	1	2160	0.05 %	941	913	1857	49.17 %
T-ALL	pediatric	UPN045	1	115258748	C	T	1855	0	1859	0.00 %	793	533	1329	40.11 %
T-ALL	pediatric	UPN045	10	89717697	T	TGGAGAAGGC	1555	0	1558	0.00 %	1092	399	1096	36.41 %
T-ALL	pediatric	UPN045	10	89717712	C	CAGGAAAGT	1698	0	1699	0.00 %	1096	386	1099	35.12 %
T-ALL	pediatric	UPN046												
T-ALL	pediatric	UPN046												
T-ALL	pediatric	UPN046	10	89653861	A	ACC	1012	0	1014	0.00 %	400	293	401	73.07 %
T-ALL	pediatric	UPN047												
T-ALL	pediatric	UPN047												
T-ALL	pediatric	UPN047												
T-ALL	pediatric	UPN047												
T-ALL	pediatric	UPN047	9	139390666	A	AG	1646	0	1665	0.00 %	1812	598	1860	32.15 %
T-ALL	pediatric	UPN048												
T-ALL	pediatric	UPN048	9	139399344	A	G	1717	0	1721	0.00 %	1176	965	2146	44.97 %
T-ALL	pediatric	UPN048	4	153247366	C	A	1077	0	1079	0.00 %	639	516	1156	44.64 %
T-ALL	pediatric	UPN048	13	77900748	G	GC	1178	0	1180	0.00 %	1324	506	1332	37.99 %
T-ALL	pediatric	UPN049												
T-ALL	pediatric	UPN049												
T-ALL	pediatric	UPN049												
T-ALL	pediatric	UPN049	10	89692788	A	AAGG	989	0	993	0.00 %	870	286	877	32.61 %

T-ALL	pediatric	UPN042										SNV/Indel
T-ALL	pediatric	UPN042										SNV/Indel
T-ALL	pediatric	UPN043	9	19702541	24070963	1	not detected	46.76 %	43.45 %	50.06 %	217	CNV
T-ALL	pediatric	UPN043										SNV/Indel
T-ALL	pediatric	UPN043										SNV/Indel
T-ALL	pediatric	UPN043										SNV/Indel
T-ALL	pediatric	UPN043										SNV/Indel
T-ALL	pediatric	UPN044										SNV/Indel
T-ALL	pediatric	UPN044	9	0	38899071	3	not detected	17.40 %	16.62 %	18.17 %	1838	CNV
T-ALL	pediatric	UPN045	9	180133	34298991	2	not detected	97.56 %	97.27 %	97.86 %	1647	CNV
T-ALL	pediatric	UPN045										SNV/Indel
T-ALL	pediatric	UPN045										SNV/Indel
T-ALL	pediatric	UPN045										SNV/Indel
T-ALL	pediatric	UPN045										SNV/Indel
T-ALL	pediatric	UPN045										SNV/Indel
T-ALL	pediatric	UPN046	9	0	33577384	2	not detected	98.00 %	98.00 %	98.00 %	1580	CNV
T-ALL	pediatric	UPN046	10	89624787	89705464	1	not detected	NA	NA	NA	2	CNV
T-ALL	pediatric	UPN046										SNV/Indel
T-ALL	pediatric	UPN047	6	64406323	171115067	1	not detected	91.90 %	91.75 %	92.05 %	3982	CNV
T-ALL	pediatric	UPN047	9	0	39154913	1	not detected	83.57 %	82.46 %	84.68 %	1942	CNV
T-ALL	pediatric	UPN047	14	22313479	22990890	1	not detected	88.84 %	83.20 %	94.47 %	56	CNV
T-ALL	pediatric	UPN047	2	144620189	243199373	3	not detected	81.41 %	80.65 %	82.16 %	3620	CNV
T-ALL	pediatric	UPN047										SNV/Indel
T-ALL	pediatric	UPN048	9	206143	44748466	2	not detected	95.06 %	94.62 %	95.49 %	1911	CNV
T-ALL	pediatric	UPN048										SNV/Indel
T-ALL	pediatric	UPN048										SNV/Indel
T-ALL	pediatric	UPN048										SNV/Indel
T-ALL	pediatric	UPN049	9	0	13776568	1	not detected	95.87 %	95.55 %	96.19 %	848	CNV
T-ALL	pediatric	UPN049	9	20818510	44734557	1	not detected	95.37 %	94.83 %	95.92 %	662	CNV
T-ALL	pediatric	UPN049	12	0	20458666	1	not detected	94.60 %	94.30 %	94.89 %	1027	CNV
T-ALL	pediatric	UPN049										SNV/Indel

1 time point

T-ALL	pediatric	UPN049	NOTCH1 (p.Ala1741_Ala1742insIleProPheGlnLeuHisPheMetTyrValAlaAla)	3	0	5	linear	3	0	2	2	1
T-ALL	pediatric	UPN050	LOH9p	1	0	98	branching (3)	1	0	0	2	2
T-ALL	pediatric	UPN050	FBXW7 (p.Arg505Cys)	1	0	98	branching (3)	1	0	0	2	1
T-ALL	pediatric	UPN050	deup in 4q	2	0	94	branching (3)	2	0	1	3	3
T-ALL	pediatric	UPN050	del in 5p	2	0	94	branching (3)	2	0	1	1	1
T-ALL	pediatric	UPN050	del in 14q	2	0	94	branching (3)	2	0	1	1	1
T-ALL	pediatric	UPN050	NOTCH1 (p.Leu1600Pro)	3	0	62	branching (3)	3	0	2	2	1
T-ALL	pediatric	UPN050	STAT5B (p.Tyr665Phe)	3	0	62	branching (3)	3	0	2	2	1
T-ALL	pediatric	UPN050	NOTCH1 (p.Ile1680Asn)	4	0	18	branching (3)	4	2	3	2	0.5
T-ALL	pediatric	UPN050	NOTCH1 (p.Leu1574Pro)	5	0	16	branching (3)	4	2	3	2	0
T-ALL	pediatric	UPN050	FBXW7 (p.Met240fs)	6	0	11	branching (3)	4	2	3	2	0.5
T-ALL	pediatric	UPN051	LOH in 9p	1	0	96	linear	1	0	0	2	2
T-ALL	pediatric	UPN051	PHF6 (p.Glu224*)	1	0	96	linear	1	0	0	2	0
T-ALL	pediatric	UPN052	LOH9p	1	0	95	linear	1	0	0	2	2
T-ALL	pediatric	UPN052	DDX3X (p.Ser74fs)	2	0	81	linear	2	0	1	1	0
T-ALL	pediatric	UPN052	NOTCH1 (p.Phe1592delinsLeuGly)	2	0	81	linear	2	0	1	2	1
T-ALL	pediatric	UPN052	PIK3CD (p.Cys381Arg)	2	0	81	linear	2	0	1	2	1
T-ALL	pediatric	UPN052	NOTCH1 (p.Gln2391delinsProTer)	3	0	72	linear	3	0	2	2	0.5
T-ALL	pediatric	UPN052	PHF6 (p.Arg24fs)	4	0	64	linear	4	0	3	1	0
T-ALL	pediatric	UPN053	LOH9p	1	0	97	linear	1	0	0	2	2
T-ALL	pediatric	UPN053	CREBBP (p.Gly1815fs)	2	0	41	linear	2	0	1	2	1
T-ALL	pediatric	UPN053	ZBTB7A (p.Lys424Asn)	2	0	41	linear	2	0	1	2	1
T-ALL	pediatric	UPN053	NOTCH1 (p.Val1578Gly)	2	0	41	linear	2	0	1	2	1
T-ALL	pediatric	UPN053	PIK3CD (p.Cys381Arg)	3	0	15	linear	3	0	2	2	1
T-ALL	pediatric	UPN054	del in 9p	1	0	61	linear	1	0	0	1	1
T-ALL	pediatric	UPN054	del in 9p	1	0	61	linear	1	0	0	1	1
T-ALL	pediatric	UPN054	LOH in 16p	2	0	42	linear	2	0	1	2	2
T-ALL	pediatric	UPN054	CREBBP (p.Asn1272fs)	2	0	42	linear	2	0	1	2	0
T-ALL	pediatric	UPN054	NOTCH1 (p.Val1578del)	2	0	42	linear	2	0	1	2	1

1 time point

T-ALL	pediatric	UPN049	9	139396885	C	CGCCGCCACGTACATGAAGTGCAGCTGGAATGGGATG	1060	0	1062	0.00 %	928	26	928	2.80 %
T-ALL	pediatric	UPN050												
T-ALL	pediatric	UPN050	4	153247289	G	A	1075	0	1077	0.00 %	664	644	1309	49.20 %
T-ALL	pediatric	UPN050												
T-ALL	pediatric	UPN050												
T-ALL	pediatric	UPN050												
T-ALL	pediatric	UPN050	9	139399344	A	G	1516	2	1522	0.13 %	1167	573	1740	32.93 %
T-ALL	pediatric	UPN050	17	40359659	T	A	1109	1	1114	0.09 %	865	379	1244	30.47 %
T-ALL	pediatric	UPN050	9	139397762	A	T	1445	0	1451	0.00 %	1531	149	1684	8.85 %
T-ALL	pediatric	UPN050	9	139399422	A	G	1422	1	1426	0.07 %	1584	135	1723	7.84 %
T-ALL	pediatric	UPN050	4	153268089	A	AT	904	0	904	0.00 %	993	57	994	5.73 %
T-ALL	pediatric	UPN051												
T-ALL	pediatric	UPN051	X	133547934	G	T	487	0	488	0.00 %	30	449	479	93.74 %
T-ALL	pediatric	UPN052												
T-ALL	pediatric	UPN052	X	41200805	TCTC	CCTCTCGG	503	0	504	0.00 %	84	365	452	80.75 %
T-ALL	pediatric	UPN052	9	139399367	G	GCCC	1553	0	1554	0.00 %	1389	571	1392	41.02 %
T-ALL	pediatric	UPN052	1	9779982	T	C	1185	0	1186	0.00 %	714	469	1188	39.48 %
T-ALL	pediatric	UPN052	9	139391019	T	TAAG	1535	0	1544	0.00 %	1516	554	1536	36.07 %
T-ALL	pediatric	UPN052	X	133511716	T	TTGGGTTAAAATAGG	520	0	520	0.00 %	461	297	464	64.01 %
T-ALL	pediatric	UPN053												
T-ALL	pediatric	UPN053	16	3779603	GC	G	1089	0	1090	0.00 %	1119	318	1446	21.99 %
T-ALL	pediatric	UPN053	19	4048233	C	A	1284	2	1289	0.16 %	1164	329	1497	21.98 %
T-ALL	pediatric	UPN053	9	139399410	A	C	1270	0	1271	0.00 %	1116	264	1388	19.02 %
T-ALL	pediatric	UPN053	1	9779982	T	C	1049	1	1052	0.10 %	1260	105	1368	7.68 %
T-ALL	pediatric	UPN054												
T-ALL	pediatric	UPN054												
T-ALL	pediatric	UPN054												
T-ALL	pediatric	UPN054	16	3799648	A	AT	777	0	778	0.00 %	591	242	596	40.60 %
T-ALL	pediatric	UPN054	9	139399408	GCAC	G	1823	10	1837	0.54 %	1169	343	1513	22.67 %

T-ALL	pediatric	UPN049										SNV/Indel
T-ALL	pediatric	UPN050	9	0	45755225	2	not detected	97.60 %	97.26 %	97.94 %	1974	CNV
T-ALL	pediatric	UPN050										SNV/Indel
T-ALL	pediatric	UPN050	4	161310770	162434592	3	not detected	90.79 %	82.31 %	99.27 %	62	CNV
T-ALL	pediatric	UPN050	5	35223427	35860332	1	not detected	84.28 %	67.87 %	100.69 %	15	CNV
T-ALL	pediatric	UPN050	14	22052636	23006341	1	not detected	84.16 %	76.92 %	91.39 %	47	CNV
T-ALL	pediatric	UPN050										SNV/Indel
T-ALL	pediatric	UPN050										SNV/Indel
T-ALL	pediatric	UPN050										SNV/Indel
T-ALL	pediatric	UPN050										SNV/Indel
T-ALL	pediatric	UPN050										SNV/Indel
T-ALL	pediatric	UPN051	9	0	36822378	2	not detected	97.49 %	97.12 %	97.87 %	1644	CNV
T-ALL	pediatric	UPN051										SNV/Indel
T-ALL	pediatric	UPN052	9	0	44769133	2	not detected	95.24 %	94.89 %	95.60 %	1683	CNV
T-ALL	pediatric	UPN052										SNV/Indel
T-ALL	pediatric	UPN052										SNV/Indel
T-ALL	pediatric	UPN052										SNV/Indel
T-ALL	pediatric	UPN052										SNV/Indel
T-ALL	pediatric	UPN052										SNV/Indel
T-ALL	pediatric	UPN053	9	0	44769133	2	not detected	97.00 %	96.00 %	97.00 %	1538	CNV
T-ALL	pediatric	UPN053										SNV/Indel
T-ALL	pediatric	UPN053										SNV/Indel
T-ALL	pediatric	UPN053										SNV/Indel
T-ALL	pediatric	UPN053										SNV/Indel
T-ALL	pediatric	UPN054	9	27905132	40185526	1	not detected	61.00 %	59.89 %	62.11 %	382	CNV
T-ALL	pediatric	UPN054	9	0	19795098	1	not detected	61.70 %	60.99 %	62.40 %	1088	CNV
T-ALL	pediatric	UPN054	16	0	8994325	2	not detected	42.15 %	41.01 %	43.29 %	522	CNV
T-ALL	pediatric	UPN054										SNV/Indel
T-ALL	pediatric	UPN054										SNV/Indel

1 time point

Supplemental Data 3

T-ALL	pediatric	UPN054	NRAS (p.Gly12Asp)	3	0	31	linear	3	0	2	2	1
T-ALL	pediatric	UPN054	CREBBP (c.3982+1G>A))	4	0	16	linear	4	0	3	2	1
T-ALL	pediatric	UPN054	SETD1B (p.Pro733fs)	4	0	16	linear	4	0	3	2	1
T-ALL	pediatric	UPN055	LOH in 9p	1	0	89	branching (3)	1	0	0	2	2
T-ALL	pediatric	UPN055	PHF6 (p.Lys75fs)	2	0	63	branching (3)	2	0	1	1	0
T-ALL	pediatric	UPN055	NOTCH1 (p.Leu1600Pro)	2	0	63	branching (3)	2	0	1	2	1
T-ALL	pediatric	UPN055	FBXW7 (p.Arg658*)	3	0	58	branching (3)	3	0	2	2	0.5
T-ALL	pediatric	UPN055	FBXW7 (p.Trp311fs)	3	0	58	branching (3)	3	0	2	2	0.5
T-ALL	pediatric	UPN055	BCL11B (p.Cys856_Asp857insIleArg)	4	0	48	branching (3)	4	0	3	2	1
T-ALL	pediatric	UPN055	NOTCH1 (p.Ile1616Asn)	5	0	12	branching (3)	5	2	4	2	0
T-ALL	pediatric	UPN055	FBXW7 (p.Arg465Cys)	6	0	3	branching (3)	5	2	4	2	0.25
T-ALL	pediatric	UPN055	NOTCH1 (p.ProTyr1618LeuGly)	7	0	3	branching (3)	5	2	4	2	0
T-ALL	pediatric	UPN056	del in 4q	1	0	94	branching (5)	1	0	0	1	1
T-ALL	pediatric	UPN056	del in 9p	1	0	94	branching (5)	1	0	0	1	1
T-ALL	pediatric	UPN056	NOTCH1 (p.Ile1680Asn)	1	0	94	branching (5)	1	0	0	2	1
T-ALL	pediatric	UPN056	PIK3CA (p.Cys420Arg)	2	0	39	branching (5)	2	4	1	2	1
T-ALL	pediatric	UPN056	PIK3CA (p.Asn107_Arg108delinsLys)	3	0	12	branching (5)	2	4	1	2	1
T-ALL	pediatric	UPN056	PIK3R1 (p.Asn453_Thr454insGly)	4	0	8	branching (5)	2	4	1	2	1
T-ALL	pediatric	UPN056	PIK3CA (p.Glu545Lys)	5	0	8	branching (5)	2	4	1	2	1
T-ALL	pediatric	UPN056	PIK3CA (p.Asn1068fs)	6	0	4	branching (5)	2	4	1	2	1
T-ALL	pediatric	UPN057	LOH in 9p	1	0	93	linear	1	0	0	2	2
T-ALL	pediatric	UPN057	BCL11B (p.Phe431Cys)	2	0	84	linear	2	0	1	2	1
T-ALL	pediatric	UPN057	FBXW7 (p.Arg505Cys)	2	0	84	linear	2	0	1	2	1
T-ALL	pediatric	UPN057	CREBBP (p.Gln1796fs)	2	0	84	linear	2	0	1	2	1
T-ALL	pediatric	UPN057	PHF6 (p.Cys298Tyr)	3	0	77	linear	3	0	2	1	0
T-ALL	pediatric	UPN057	NOTCH1 (p.GluIle1679AspSer)	3	0	77	linear	3	0	2	2	1
T-ALL	pediatric	UPN057	USP7 (p.Ser252delinsTerValGluGlyPro)	4	0	55	linear	4	0	3	2	1
T-ALL	pediatric	UPN057	PIK3R1 (p.Asn564_Pro568del)	5	0	16	linear	5	0	4	2	1

1 time point

Supplemental Data 3

T-ALL	pediatric	UPN054	1	115258747	C	T	1260	0	1261	0.00 %	974	179	1156	15.48 %
T-ALL	pediatric	UPN054	16	3794894	C	T	1189	0	1193	0.00 %	912	82	996	8.23 %
T-ALL	pediatric	UPN054	12	122252315	G	GCGAGCCCGGAGCC GGC	615	0	624	0.00 %	458	35	463	7.56 %
T-ALL	pediatric	UPN055												
T-ALL	pediatric	UPN055	X	133512118	T	TTTTGGGG	606	0	607	0.00 %	362	233	364	64.01 %
T-ALL	pediatric	UPN055	9	139399344	A	G	2389	2	2396	0.08 %	1306	607	1919	31.63 %
T-ALL	pediatric	UPN055	4	153244185	G	A	1644	0	1649	0.00 %	881	365	1249	29.22 %
T-ALL	pediatric	UPN055	4	153253801	C	CA	1335	0	1338	0.00 %	1087	298	1091	27.31 %
T-ALL	pediatric	UPN055	14	99640604	C	CGCGAAT	1715	0	1719	0.00 %	1604	391	1607	24.33 %
T-ALL	pediatric	UPN055	9	139399296	A	T	2048	0	2050	0.00 %	1532	100	1637	6.11 %
T-ALL	pediatric	UPN055	4	153249385	G	A	1691	1	1696	0.06 %	1343	24	1370	1.75 %
T-ALL	pediatric	UPN055	9	139399286	GTAGG	CCCCA	2072	0	2082	0.00 %	1631	21	1658	1.27 %
T-ALL	pediatric	UPN056												
T-ALL	pediatric	UPN056												
T-ALL	pediatric	UPN056	9	139397762	A	T	1454	1	1459	0.07 %	896	779	1682	46.31 %
T-ALL	pediatric	UPN056	3	178927980	T	C	633	1	635	0.16 %	589	144	735	19.59 %
T-ALL	pediatric	UPN056	3	178916931	CAACCGT	AAAA	663	4	665	0.60 %	700	45	745	6.04 %
T-ALL	pediatric	UPN056	5	67589596	C	CGGG	769	0	769	0.00 %	751	30	753	3.98 %
T-ALL	pediatric	UPN056	3	178936091	G	A	917	0	920	0.00 %	966	38	1006	3.78 %
T-ALL	pediatric	UPN056	3	178952146	G	GA	825	0	825	0.00 %	921	20	924	2.16 %
T-ALL	pediatric	UPN057												
T-ALL	pediatric	UPN057	14	99641881	A	C	1303	1	1309	0.08 %	679	512	1192	42.95 %
T-ALL	pediatric	UPN057	4	153247289	G	A	1389	2	1394	0.14 %	646	478	1124	42.53 %
T-ALL	pediatric	UPN057	16	3779661	T	TA	1375	0	1378	0.00 %	1384	583	1392	41.88 %
T-ALL	pediatric	UPN057	X	133551254	G	A	601	0	602	0.00 %	101	353	455	77.58 %
T-ALL	pediatric	UPN057	9	139397761	AATC	GCTA	1638	1	1643	0.06 %	832	515	1352	38.09 %
T-ALL	pediatric	UPN057	16	9010979	G	GGTCCCTCCACTC	926	0	930	0.00 %	792	222	803	27.65 %
T-ALL	pediatric	UPN057	5	67591096	GAACAGCATTAAACCA	G	1099	0	1103	0.00 %	802	73	891	8.19 %

1 time point

T-ALL	pediatric	UPN054									SNV/Indel
T-ALL	pediatric	UPN054									SNV/Indel
T-ALL	pediatric	UPN054									SNV/Indel
T-ALL	pediatric	UPN055	9	0	35721467	2	not detected	73.13 %	71.69 %	74.57 %	1762	CNV
T-ALL	pediatric	UPN055										SNV/Indel
T-ALL	pediatric	UPN055										SNV/Indel
T-ALL	pediatric	UPN055										SNV/Indel
T-ALL	pediatric	UPN055										SNV/Indel
T-ALL	pediatric	UPN055										SNV/Indel
T-ALL	pediatric	UPN055										SNV/Indel
T-ALL	pediatric	UPN055										SNV/Indel
T-ALL	pediatric	UPN055										SNV/Indel
T-ALL	pediatric	UPN056	4	108948898	109370588	1	not detected	96.44 %	95.12 %	97.76 %	8	CNV
T-ALL	pediatric	UPN056	9	0	39147759	1	not detected	96.14 %	95.82 %	96.46 %	1784	CNV
T-ALL	pediatric	UPN056										SNV/Indel
T-ALL	pediatric	UPN056										SNV/Indel
T-ALL	pediatric	UPN056										SNV/Indel
T-ALL	pediatric	UPN056										SNV/Indel
T-ALL	pediatric	UPN056										SNV/Indel
T-ALL	pediatric	UPN056										SNV/Indel
T-ALL	pediatric	UPN057	9	0	33294320	2	not detected	93.61 %	93.31 %	93.91 %	1709	CNV
T-ALL	pediatric	UPN057										SNV/Indel
T-ALL	pediatric	UPN057										SNV/Indel
T-ALL	pediatric	UPN057										SNV/Indel
T-ALL	pediatric	UPN057										SNV/Indel
T-ALL	pediatric	UPN057										SNV/Indel
T-ALL	pediatric	UPN057										SNV/Indel
T-ALL	pediatric	UPN057										SNV/Indel

1 time point

T-ALL	pediatric	UPN058	dup in 16q	1	0	78	linear	1	0	0	3	3
T-ALL	pediatric	UPN058	dup in 9p coming from del	1	0	78	linear	1	0	0	3	3
T-ALL	pediatric	UPN058	del in 10q	2	0	58	linear	2	0	1	1	1
T-ALL	pediatric	UPN058	NOTCH1 (p.Gln2487*)	2	0	58	linear	2	0	1	2	1
T-ALL	pediatric	UPN058	NOTCH1 (p.Ser385del)	3	0	42	linear	3	0	2	2	0.5
T-ALL	pediatric	UPN058	NOTCH1 (p.Gln2395*)	4	0	2	linear	4	0	3	2	0.25
T-ALL	pediatric	UPN059	del in 9p	1	0	92	branching (4)	1	0	0	1	1
T-ALL	pediatric	UPN059	dup in 9p	1	0	92	branching (4)	1	0	0	3	3
T-ALL	pediatric	UPN059	del in 11q	2	0	46	branching (4)	2	0	1	1	1
T-ALL	pediatric	UPN059	del in 17p	2	0	46	branching (4)	2	0	1	1	1
T-ALL	pediatric	UPN059	del in 17p	2	0	46	branching (4)	2	0	1	1	1
T-ALL	pediatric	UPN059	del in 17p	2	0	46	branching (4)	2	0	1	1	1
T-ALL	pediatric	UPN059	NOTCH1 (p.Phe1592Ser)	2	0	46	branching (4)	2	0	1	2	1
T-ALL	pediatric	UPN059	NOTCH1 (p.Ser2492*)	2	0	46	branching (4)	2	0	1	2	0
T-ALL	pediatric	UPN059	NOTCH1 (p.Ser2471*)	2	0	46	branching (4)	2	0	1	2	0
T-ALL	pediatric	UPN059	JAK3 (p.Gln507Pro)	3	0	30	branching (4)	3	0	2	2	1
T-ALL	pediatric	UPN059	JAK3 (p.Ala573Val)	4	0	22	branching (4)	4	0	3	2	0.5
T-ALL	pediatric	UPN059	STAT5B (p.Asn642His)	5	0	11	branching (4)	5	3	4	2	1
T-ALL	pediatric	UPN059	NOTCH1 (p.Cys1692Arg)	6	0	6	branching (4)	5	3	4	2	1
T-ALL	pediatric	UPN059	NOTCH1 (p.Ile1616Thr)	7	0	3	branching (4)	5	3	4	2	1
T-ALL	pediatric	UPN059	NOTCH1 (p.Leu1585Gln)	8	0	2	branching (4)	5	3	4	2	1
T-ALL	pediatric	UPN060	del in 5q	1	0	90	linear	1	0	0	1	1
T-ALL	pediatric	UPN060	del in 14q	1	0	90	linear	1	0	0	1	1
T-ALL	pediatric	UPN060	del in 3p	2	0	29	linear	2	0	1	1	1
T-ALL	pediatric	UPN061	dup in 10p	1	0	92	linear	1	0	0	3	3
T-ALL	pediatric	UPN061	dup in 7q	2	0	80	linear	2	0	1	3	3
T-ALL	pediatric	UPN061	LOH in 9p	2	0	80	linear	2	0	1	2	2
T-ALL	pediatric	UPN061	dup9p	2	0	80	linear	2	0	1	3	3
T-ALL	pediatric	UPN061	PHF6 (p.Phe215Ser)	2	0	80	linear	2	0	1	2	1
T-ALL	pediatric	UPN061	CCND3 (p.Arg271fs)	3	0	27	linear	3	0	2	2	0
T-ALL	pediatric	UPN061	NOTCH1 (p.Leu1600Pro)	3	0	27	linear	3	0	2	2	1
T-ALL	pediatric	UPN061	BCL11B (p.Asn352fs)	3	0	27	linear	3	0	2	2	0.5
T-ALL	pediatric	UPN061	CCND3 (p.Arg252fs)	3	0	27	linear	3	0	2	2	0
T-ALL	pediatric	UPN061	BCL11B (p.Gln466fs)	3	0	27	linear	3	0	2	2	0.5
T-ALL	pediatric	UPN061	NOTCH1 (p.Pro2514fs)	4	0	14	linear	4	0	3	2	0.5

Supplemental Data 3

T-ALL	pediatric	UPN058												
T-ALL	pediatric	UPN058												
T-ALL	pediatric	UPN058												
T-ALL	pediatric	UPN058	9	139390732	G	A	1447	1	1452	0.07 %	1316	534	1851	28.85 %
T-ALL	pediatric	UPN058	9	139412688	TGGA	T	1268	0	1273	0.00 %	1417	380	1801	21.10 %
T-ALL	pediatric	UPN058	9	139391008	G	A	1678	1	1685	0.06 %	2034	22	2058	1.07 %
T-ALL	pediatric	UPN059												
T-ALL	pediatric	UPN059												
T-ALL	pediatric	UPN059												
T-ALL	pediatric	UPN059												
T-ALL	pediatric	UPN059												
T-ALL	pediatric	UPN059												
T-ALL	pediatric	UPN059	9	139399368	A	G	1663	0	1665	0.00 %	1291	403	1696	23.76 %
T-ALL	pediatric	UPN059	9	139390716	G	T	1607	0	1613	0.00 %	1238	363	1605	22.62 %
T-ALL	pediatric	UPN059	9	139390779	G	T	1595	1	1599	0.06 %	1204	338	1544	21.89 %
T-ALL	pediatric	UPN059	19	17949121	T	G	1491	4	1497	0.27 %	1142	200	1346	14.86 %
T-ALL	pediatric	UPN059	19	17948006	G	A	1329	1	1333	0.08 %	1209	141	1354	10.41 %
T-ALL	pediatric	UPN059	17	40359729	T	G	903	0	905	0.00 %	840	62	904	6.86 %
T-ALL	pediatric	UPN059	9	139397727	A	G	1700	1	1704	0.06 %	1523	53	1587	3.34 %
T-ALL	pediatric	UPN059	9	139399296	A	G	1567	2	1570	0.13 %	1516	31	1551	2.00 %
T-ALL	pediatric	UPN059	9	139399389	A	T	1602	0	1612	0.00 %	1574	23	1603	1.43 %
T-ALL	pediatric	UPN060												
T-ALL	pediatric	UPN060												
T-ALL	pediatric	UPN060												
T-ALL	pediatric	UPN061												
T-ALL	pediatric	UPN061												
T-ALL	pediatric	UPN061												
T-ALL	pediatric	UPN061												
T-ALL	pediatric	UPN061	X	133547908	T	C	1061	0	1064	0.00 %	451	326	777	41.96 %
T-ALL	pediatric	UPN061	6	41903745	C	CG	1739	0	1742	0.00 %	1883	304	1896	16.03 %
T-ALL	pediatric	UPN061	9	139399344	A	G	2176	0	2183	0.00 %	1630	254	1892	13.42 %
T-ALL	pediatric	UPN061	14	99642119	T	TCA	1585	0	1588	0.00 %	1218	160	1227	13.04 %
T-ALL	pediatric	UPN061	6	41903803	T	TCC	1777	0	1783	0.00 %	1969	245	1977	12.39 %
T-ALL	pediatric	UPN061	14	99641777	G	GGTAAC	1021	0	1022	0.00 %	1018	126	1018	12.38 %
T-ALL	pediatric	UPN061	9	139390648	CAG	C	1722	0	1726	0.00 %	1430	139	1581	8.79 %

1 time point

T-ALL	pediatric	UPN058	16	77759157	79010968	3	not detected	80.24 %	75.86 %	84.63 %	79.88 %	CNV
T-ALL	pediatric	UPN058	9	0	31550952	3	not detected	77.83 %	77.45 %	78.21 %	77.59 %	CNV
T-ALL	pediatric	UPN058	10	89627829	89926115	1	not detected	60.62 %	52.70 %	68.55 %	57.88 %	CNV
T-ALL	pediatric	UPN058										SNV/Indel
T-ALL	pediatric	UPN058										SNV/Indel
T-ALL	pediatric	UPN058										SNV/Indel
T-ALL	pediatric	UPN059	9	21475634	21910122	1	not detected	93.34 %	92.62 %	94.06 %	17	CNV
T-ALL	pediatric	UPN059	9	22029144	23209563	3	not detected	91.02 %	84.12 %	97.92 %	81	CNV
T-ALL	pediatric	UPN059	11	81890697	126188806	1	not detected	48.44 %	48.00 %	48.89 %	1875	CNV
T-ALL	pediatric	UPN059	17	2522660	5040264	1	not detected	45.09 %	42.99 %	47.20 %	134	CNV
T-ALL	pediatric	UPN059	17	33318755	34441525	1	not detected	48.08 %	45.49 %	50.67 %	50	CNV
T-ALL	pediatric	UPN059	17	41753427	42877663	1	not detected	43.95 %	40.04 %	47.86 %	38	CNV
T-ALL	pediatric	UPN059										SNV/Indel
T-ALL	pediatric	UPN059										SNV/Indel
T-ALL	pediatric	UPN059										SNV/Indel
T-ALL	pediatric	UPN059										SNV/Indel
T-ALL	pediatric	UPN059										SNV/Indel
T-ALL	pediatric	UPN059										SNV/Indel
T-ALL	pediatric	UPN059										SNV/Indel
T-ALL	pediatric	UPN059										SNV/Indel
T-ALL	pediatric	UPN059										SNV/Indel
T-ALL	pediatric	UPN060	6	62442530	103219959	1	not detected	91.03 %	90.70 %	91.37 %	1167	CNV
T-ALL	pediatric	UPN060	14	22392802	22962374	1	not detected	88.96 %	83.07 %	94.85 %	44	CNV
T-ALL	pediatric	UPN060	3	43661196	85064279	1	not detected	29.62 %	29.09 %	30.15 %	1455	CNV
T-ALL	pediatric	UPN061	10	93083	36444351	3	not detected	92.16 %	90.88 %	93.44 %	1687	CNV
T-ALL	pediatric	UPN061	7	91922155	159138663	3	not detected	79.78 %	78.81 %	80.75 %	2502	CNV
T-ALL	pediatric	UPN061	9	0	37748004	2	not detected	80.80 %	80.38 %	81.21 %	1831	CNV
T-ALL	pediatric	UPN061	9	0	39189362	3	not detected	81.65 %	73.77 %	89.52 %	57	CNV
T-ALL	pediatric	UPN061										SNV/Indel
T-ALL	pediatric	UPN061										SNV/Indel
T-ALL	pediatric	UPN061										SNV/Indel
T-ALL	pediatric	UPN061										SNV/Indel
T-ALL	pediatric	UPN061										SNV/Indel
T-ALL	pediatric	UPN061										SNV/Indel
T-ALL	pediatric	UPN061										SNV/Indel

T-ALL	pediatric	UPN061	PTEN (p.Asp92Gly)	4	0	14	linear	4	0	3	2	0
T-ALL	pediatric	UPN061	PTEN (p.Arg130Gln)	4	0	14	linear	4	0	3	2	0
T-ALL	pediatric	UPN062	LOH in 9p	1	0	82	linear	1	0	0	2	2
T-ALL	pediatric	UPN062	USP7 (p.Asp482Glu)	2	0	52	linear	2	0	1	2	1
T-ALL	pediatric	UPN062	PTEN (p.Phe241fs)	3	0	41	linear	3	0	2	2	1
T-ALL	pediatric	UPN062	NOTCH1 (p.Leu2457fs)	4	0	12	linear	4	0	3	2	1
T-ALL	pediatric	UPN063	del9p	1	0	97	linear	1	0	0	1	1
T-ALL	pediatric	UPN063	RUNX1 (p.Arg320*)	2	0	89	linear	2	0	1	2	1
T-ALL	pediatric	UPN063	JAK3 (p.Gln507Pro)	2	0	89	linear	2	0	1	2	0.5
T-ALL	pediatric	UPN063	JAK3 (p.Ala573Val)	2	0	89	linear	2	0	1	2	0.5
T-ALL	pediatric	UPN063	dup in 18q	3	0	80	linear	3	0	2	3	3
T-ALL	pediatric	UPN063	dup in 18q	3	0	80	linear	3	0	2	3	3
T-ALL	pediatric	UPN063	dup9q	4	0	75	linear	4	0	3	3	3
T-ALL	pediatric	UPN063	dup in 18q	4	0	75	linear	4	0	3	3	3
T-ALL	pediatric	UPN063	LOH in 6q	5	0	52	linear	5	0	4	2	2
T-ALL	pediatric	UPN063	dup in 18p	6	0	40	linear	6	0	5	3	3
T-ALL	pediatric	UPN063	dup in 18p	6	0	40	linear	6	0	5	3	3
T-ALL	pediatric	UPN063	dup in 18q	6	0	40	linear	6	0	5	3	3
T-ALL	pediatric	UPN063	dup in 18q	6	0	40	linear	6	0	5	3	3
T-ALL	pediatric	UPN063	dup in 18q	6	0	40	linear	6	0	5	3	3
T-ALL	pediatric	UPN063	dup in 18q	6	0	40	linear	6	0	5	3	3
T-ALL	pediatric	UPN063	dup in 18q	6	0	40	linear	6	0	5	3	3
T-ALL	pediatric	UPN063	NOTCH1 (p.Leu1600Gln)	7	0	20	linear	7	0	6	2	1
T-ALL	pediatric	UPN064	del in 9p	1	0	98	linear	1	0	0	1	1
T-ALL	pediatric	UPN064	del in 14q	1	0	98	linear	1	0	0	1	1
T-ALL	pediatric	UPN064	NOTCH1 (p.Arg1598Pro)	1	0	98	linear	1	0	0	2	1
T-ALL	pediatric	UPN064	FBXW7 (p.Arg479Gln)	2	0	88	linear	2	0	1	2	1
T-ALL	pediatric	UPN064	NRAS (p.Ala59Thr)	3	0	12	linear	3	0	2	2	1
T-ALL	pediatric	UPN064	SETD1B (p.Asp1566fs)	3	0	12	linear	3	0	2	2	1
T-ALL	pediatric	UPN065	del in 9p	1	0	98	linear	1	0	0	1	1
T-ALL	pediatric	UPN065	NOTCH1 (p.Leu1678Pro)	1	0	98	linear	1	0	0	2	1
T-ALL	pediatric	UPN065	DNM2 (p.Pro791fs)	1	0	98	linear	1	0	0	2	1
T-ALL	pediatric	UPN065	KMT2A (p.Arg3789fs)	2	0	67	linear	2	0	1	2	1
T-ALL	pediatric	UPN065	CCND3 (p.Leu251fs)	3	0	48	linear	3	0	2	2	1

Supplemental Data 3

T-ALL	pediatric	UPN061	10	89692791	A	G	969	1	971	0.10 %	709	67	778	8.61 %
T-ALL	pediatric	UPN061	10	89692905	G	A	1241	0	1243	0.00 %	954	60	1014	5.92 %
T-ALL	pediatric	UPN062												
T-ALL	pediatric	UPN062	16	8999171	G	C	1055	0	1058	0.00 %	829	291	1122	25.94 %
T-ALL	pediatric	UPN062	10	89717697	T	TGGGTAGTA	878	0	880	0.00 %	1047	219	1053	20.80 %
T-ALL	pediatric	UPN062	9	139390821	A	AGG	1324	0	1325	0.00 %	1846	108	1855	5.82 %
T-ALL	pediatric	UPN063												
T-ALL	pediatric	UPN063	21	36171607	G	A	1347	0	1349	0.00 %	675	564	1239	45.52 %
T-ALL	pediatric	UPN063	19	17949121	T	G	1435	3	1446	0.21 %	693	567	1264	44.86 %
T-ALL	pediatric	UPN063	19	17948006	G	A	1375	0	1376	0.00 %	765	619	1391	44.50 %
T-ALL	pediatric	UPN063												
T-ALL	pediatric	UPN063												
T-ALL	pediatric	UPN063												
T-ALL	pediatric	UPN063												
T-ALL	pediatric	UPN063												
T-ALL	pediatric	UPN063												
T-ALL	pediatric	UPN063												
T-ALL	pediatric	UPN063												
T-ALL	pediatric	UPN063												
T-ALL	pediatric	UPN063												
T-ALL	pediatric	UPN063												
T-ALL	pediatric	UPN063												
T-ALL	pediatric	UPN063	9	139399344	A	T	1923	0	1930	0.00 %	2159	242	2403	10.07 %
T-ALL	pediatric	UPN064												
T-ALL	pediatric	UPN064												
T-ALL	pediatric	UPN064	9	139399350	C	G	2672	3	2679	0.11 %	1390	1332	2727	48.84 %
T-ALL	pediatric	UPN064	4	153247366	C	T	1674	0	1680	0.00 %	777	618	1399	44.17 %
T-ALL	pediatric	UPN064	1	115256536	C	T	1645	0	1649	0.00 %	1413	97	1517	6.39 %
T-ALL	pediatric	UPN064	12	122261179	A	AT	2242	0	2294	0.00 %	2292	141	2358	5.98 %
T-ALL	pediatric	UPN065												
T-ALL	pediatric	UPN065	9	139397768	A	G	1990	1	1996	0.05 %	1072	1114	2216	50.27 %
T-ALL	pediatric	UPN065	19	10940881	GCC	GA	1453	2	1454	0.14 %	810	752	1566	48.02 %
T-ALL	pediatric	UPN065	11	118390713	ATC	ATGGTCCTTT	1526	6	1532	0.39 %	911	481	1431	33.61 %
T-ALL	pediatric	UPN065	6	41903807	T	TGCAGGTTACGGGG	2167	0	2175	0.00 %	2121	523	2152	24.30 %

1 time point

T-ALL	pediatric	UPN061										SNV/Indel
T-ALL	pediatric	UPN061										SNV/Indel
T-ALL	pediatric	UPN062	9	364604	36469174	2	not detected	81.92 %	81.47 %	82.37 %	1777	CNV
T-ALL	pediatric	UPN062										SNV/Indel
T-ALL	pediatric	UPN062										SNV/Indel
T-ALL	pediatric	UPN062										SNV/Indel
T-ALL	pediatric	UPN063	9	0	39239013	1	not detected	94.20 %	93.54 %	94.86 %	1904	CNV
T-ALL	pediatric	UPN063										SNV/Indel
T-ALL	pediatric	UPN063										SNV/Indel
T-ALL	pediatric	UPN063										SNV/Indel
T-ALL	pediatric	UPN063	18	31402598	34203980	3	not detected	80.31 %	74.77 %	85.85 %	86	CNV
T-ALL	pediatric	UPN063	18	50244156	54084761	3	not detected	80.80 %	76.08 %	85.51 %	107	CNV
T-ALL	pediatric	UPN063	9	70731742	141213431	3	not detected	76.25 %	75.35 %	77.14 %	3045	CNV
T-ALL	pediatric	UPN063	18	18846683	25347936	3	not detected	73.19 %	69.71 %	76.67 %	209	CNV
T-ALL	pediatric	UPN063	6	65882750	170919470	2	not detected	52.35 %	52.02 %	52.68 %	3734	CNV
T-ALL	pediatric	UPN063	18	0	4916906	3	not detected	42.23 %	40.19 %	44.26 %	326	CNV
T-ALL	pediatric	UPN063	18	7170124	15047653	3	not detected	37.69 %	35.68 %	39.70 %	382	CNV
T-ALL	pediatric	UPN063	18	25347937	31402597	3	not detected	43.18 %	40.95 %	45.41 %	220	CNV
T-ALL	pediatric	UPN063	18	34203981	41388171	3	not detected	44.03 %	41.43 %	46.62 %	179	CNV
T-ALL	pediatric	UPN063	18	43692535	50244155	3	not detected	41.05 %	39.02 %	43.09 %	284	CNV
T-ALL	pediatric	UPN063	18	54084762	61314136	3	not detected	42.04 %	39.92 %	44.17 %	334	CNV
T-ALL	pediatric	UPN063	18	72971503	78077248	3	not detected	36.70 %	34.36 %	39.05 %	246	CNV
T-ALL	pediatric	UPN063										SNV/Indel
T-ALL	pediatric	UPN064	9	0	37230469	1	not detected	97.67 %	97.37 %	97.97 %	1752	CNV
T-ALL	pediatric	UPN064	14	22527266	22921492	1	not detected	99.60 %	99.32 %	99.88 %	36	CNV
T-ALL	pediatric	UPN064										SNV/Indel
T-ALL	pediatric	UPN064										SNV/Indel
T-ALL	pediatric	UPN064										SNV/Indel
T-ALL	pediatric	UPN064										SNV/Indel
T-ALL	pediatric	UPN065	9	19671057	27875005	1	not detected	82.93 %	80.02 %	85.85 %	367	CNV
T-ALL	pediatric	UPN065										SNV/Indel
T-ALL	pediatric	UPN065										SNV/Indel
T-ALL	pediatric	UPN065										SNV/Indel
T-ALL	pediatric	UPN065										SNV/Indel

1 time point

T-LBL	adult	UPN078	FBXW7 (p.Trp649*)	1	0	96	linear	1	0	0	2	1
T-LBL	adult	UPN078	NOTCH1 (p.Arg1598Pro)	1	0	96	linear	1	0	0	2	1
T-LBL	adult	UPN078	del in 7q	2	0	86	linear	2	0	1	1	1
T-LBL	adult	UPN078	del in 9q	2	0	86	linear	2	0	1	1	1
T-LBL	adult	UPN078	FBXW7 (p.Arg465Cys)	2	0	86	linear	2	0	1	2	0.5
T-LBL	adult	UPN078	NRAS (p.Gly12Val)	3	0	54	linear	3	0	2	2	1
T-LBL	adult	UPN078	PIK3CA (p.Glu542Lys)	4	0	33	linear	4	0	3	2	1
T-LBL	adult	UPN079	FBXW7 (p.Arg505Leu)	1	0	91	linear	1	0	0	2	1
T-LBL	adult	UPN079	dup in 17q	2	0	79	linear	2	0	1	3	3
T-LBL	adult	UPN079	NOTCH1 (p.Glu1583delinsProGly)	2	0	79	linear	2	0	1	2	1
T-LBL	adult	UPN080	dup9p	1	0	17	linear	1	0	0	3	3
T-LBL	adult	UPN080	NOTCH1 (p.Leu1574Pro)	2	0	2	linear	2	0	1	2	1
T-LBL	adult	UPN082	LOH in 2p	1	100	100	linear	1	0	0	2	2
T-LBL	adult	UPN082	LOH in 2p	1	100	100	linear	1	0	0	2	2
T-LBL	adult	UPN082	LOH in 2q	1	100	100	linear	1	0	0	2	2
T-LBL	adult	UPN082	LOH in 2q	1	100	100	linear	1	0	0	2	2
T-LBL	adult	UPN082	LOH in 3q	1	100	100	linear	1	0	0	2	2
T-LBL	adult	UPN082	LOH in 4p	1	100	100	linear	1	0	0	2	2
T-LBL	adult	UPN082	LOH in 5p	1	100	100	linear	1	0	0	2	2
T-LBL	adult	UPN082	LOH in 6p	1	100	100	linear	1	0	0	2	2
T-LBL	adult	UPN082	LOH in 6p	1	100	100	linear	1	0	0	2	2
T-LBL	adult	UPN082	LOH in 6q	1	100	100	linear	1	0	0	2	2
T-LBL	adult	UPN082	LOH in 9p	1	100	100	linear	1	0	0	2	2
T-LBL	adult	UPN082	LOH in 9q	1	100	100	linear	1	0	0	2	2
T-LBL	adult	UPN082	LOH in 10p	1	100	100	linear	1	0	0	2	2
T-LBL	adult	UPN082	LOH in 10q	1	100	100	linear	1	0	0	2	2
T-LBL	adult	UPN082	LOH in 12p	1	100	100	linear	1	0	0	2	2
T-LBL	adult	UPN082	LOH in 12q	1	100	100	linear	1	0	0	2	2
T-LBL	adult	UPN082	LOH in 12q	1	100	100	linear	1	0	0	2	2
T-LBL	adult	UPN082	LOH in 13q	1	100	100	linear	1	0	0	2	2
T-LBL	adult	UPN082	LOH in 15q	1	100	100	linear	1	0	0	2	2
T-LBL	adult	UPN082	LOH in 18q	1	100	100	linear	1	0	0	2	2
T-LBL	adult	UPN082	LOH in 20p	1	100	100	linear	1	0	0	2	2
T-LBL	adult	UPN082	STAT5B (p.Gln368fs)	2	0	29	linear	2	0	1	2	1

1 time point

T-LBL	adult	UPN078	4	153244210	C	T	1671	2	1675	0.12 %	496	491	988	49.70 %
T-LBL	adult	UPN078	9	139399350	C	G	1822	0	1824	0.00 %	617	564	1182	47.72 %
T-LBL	adult	UPN078												
T-LBL	adult	UPN078												
T-LBL	adult	UPN078	4	153249385	G	A	1795	1	1801	0.06 %	604	476	1085	43.87 %
T-LBL	adult	UPN078	1	115258747	C	A	1862	1	1864	0.05 %	834	307	1141	26.91 %
T-LBL	adult	UPN078	3	178936082	G	A	1496	0	1500	0.00 %	636	126	763	16.51 %
T-LBL	adult	UPN079	4	153247288	C	A	1749	2	1754	0.11 %	764	642	1410	45.53 %
T-LBL	adult	UPN079												
T-LBL	adult	UPN079	9	139399394	CTCC	TCCCGGG	1864	6	1865	0.32 %	1141	748	1895	39.47 %
T-LBL	adult	UPN080												
T-LBL	adult	UPN080	9	139399422	A	G	2082	4	2090	0.19 %	2204	25	2236	1.12 %
T-LBL	adult	UPN082												
T-LBL	adult	UPN082												
T-LBL	adult	UPN082												
T-LBL	adult	UPN082												
T-LBL	adult	UPN082												
T-LBL	adult	UPN082												
T-LBL	adult	UPN082												
T-LBL	adult	UPN082												
T-LBL	adult	UPN082												
T-LBL	adult	UPN082												
T-LBL	adult	UPN082												
T-LBL	adult	UPN082												
T-LBL	adult	UPN082												
T-LBL	adult	UPN082												
T-LBL	adult	UPN082												
T-LBL	adult	UPN082												
T-LBL	adult	UPN082												
T-LBL	adult	UPN082												
T-LBL	adult	UPN082												
T-LBL	adult	UPN082												
T-LBL	adult	UPN082												
T-LBL	adult	UPN082	17	40370235	T	TG	1855	16	1865	0.86 %	859	140	868	16.13 %

T-LBL	adult	UPN078										SNV/Indel
T-LBL	adult	UPN078										SNV/Indel
T-LBL	adult	UPN078	7	0	52122844	1	not detected	84.34 %	83.74 %	84.94 %	2569	CNV
T-LBL	adult	UPN078	9	0	26484190	1	not detected	85.15 %	84.34 %	85.96 %	1378	CNV
T-LBL	adult	UPN078										SNV/Indel
T-LBL	adult	UPN078										SNV/Indel
T-LBL	adult	UPN078										SNV/Indel
T-LBL	adult	UPN079										SNV/Indel
T-LBL	adult	UPN079	17	48714783	81195210	3	not detected	77.71 %	75.91 %	79.51 %	1452	CNV
T-LBL	adult	UPN079										SNV/Indel
T-LBL	adult	UPN080	9	0	44902403	3	not detected	17.45 %	16.65 %	18.26 %	1847	CNV
T-LBL	adult	UPN080										SNV/Indel
T-LBL	adult	UPN082	2	2574901	28706639	2	100.00 %	100.00 %				CNV
T-LBL	adult	UPN082	2	46256532	53507902	2	100.00 %	100.00 %				CNV
T-LBL	adult	UPN082	2	164782436	171188134	2	100.00 %	100.00 %				CNV
T-LBL	adult	UPN082	2	192134975	216823871	2	100.00 %	100.00 %				CNV
T-LBL	adult	UPN082	3	185912262	188943804	2	100.00 %	100.00 %				CNV
T-LBL	adult	UPN082	4	0	9926760	2	100.00 %	100.00 %				CNV
T-LBL	adult	UPN082	5	5110842	24576253	2	100.00 %	100.00 %				CNV
T-LBL	adult	UPN082	6	33077439	45356966	2	100.00 %	100.00 %				CNV
T-LBL	adult	UPN082	6	52075963	61000000	2	100.00 %	100.00 %				CNV
T-LBL	adult	UPN082	6	61000000	73463737	2	100.00 %	100.00 %				CNV
T-LBL	adult	UPN082	9	29154819	37576500	2	100.00 %	100.00 %				CNV
T-LBL	adult	UPN082	9	107920936	126819405	2	100.00 %	100.00 %				CNV
T-LBL	adult	UPN082	10	25200701	28611874	2	100.00 %	100.00 %				CNV
T-LBL	adult	UPN082	10	125510523	128460849	2	100.00 %	100.00 %				CNV
T-LBL	adult	UPN082	12	22500913	35800000	2	100.00 %	100.00 %				CNV
T-LBL	adult	UPN082	12	37891147	63295815	2	100.00 %	100.00 %				CNV
T-LBL	adult	UPN082	12	108063049	116966247	2	100.00 %	100.00 %				CNV
T-LBL	adult	UPN082	13	97517984	110819840	2	100.00 %	100.00 %				CNV
T-LBL	adult	UPN082	15	68882819	92172224	2	100.00 %	100.00 %				CNV
T-LBL	adult	UPN082	18	65237158	68039954	2	100.00 %	100.00 %				CNV
T-LBL	adult	UPN082	20	4350387	11174695	2	100.00 %	100.00 %				CNV
T-LBL	adult	UPN082										SNV/Indel

1 time point

T-LBL	adult	UPN082	NOTCH3 (p.Gly2035fs)	2	0	29	linear	2	0	1	2	1
T-LBL	adult	UPN082	NOTCH3 (p.Ala1802fs)	3	0	19	linear	3	0	2	2	0.5
T-LBL	adult	UPN082	KMT2D (p.Leu434fs)	4	0	10	linear	4	0	3	2	0.25
T-LBL	adult	UPN082	MED12 (p.Lys1768fs)	4	0	10	linear	4	0	3	2	0.5
T-LBL	adult	UPN082	TET2 (p.Asp1335Gly)	4	0	10	linear	4	0	3	2	1
T-LBL	adult	UPN082	NOS3 (p.Arg1108Trp)	4	0	10	linear	4	0	3	2	1
T-LBL	adult	UPN082	SETD1B (p.Ala1776fs)	4	0	10	linear	4	0	3	2	0.5
T-LBL	adult	UPN082	KMT2D (p.Ala1390fs)	4	0	10	linear	4	0	3	2	0.25
T-LBL	adult	UPN082	SETD1B (p.His1362fs)	4	0	10	linear	4	0	3	2	0.5
T-LBL	adult	UPN082	KMT2D (p.Asn2517fs)	4	0	10	linear	4	0	3	2	0.25
T-LBL	adult	UPN082	MYB (p.Thr526Asn)	4	0	10	linear	4	0	3	2	1
T-LBL	adult	UPN082	ROR2 (p.His362fs)	4	0	10	linear	4	0	3	2	1
T-LBL	adult	UPN082	KMT2A (p.Ser774fs)	4	0	10	linear	4	0	3	2	1
T-LBL	adult	UPN082	SMARCA4 (p.Gly271fs)	4	0	10	linear	4	0	3	2	1
T-LBL	adult	UPN082	CNOT3 (p.Ser392fs)	4	0	10	linear	4	0	3	2	1
T-LBL	adult	UPN082	MED12 (p.Ser1970fs)	4	0	10	linear	4	0	3	2	0.5
T-LBL	adult	UPN082	USH2A (p.Arg2662Ile)	4	0	10	linear	4	0	3	2	1
T-LBL	adult	UPN082	NOTCH3 (p.Ala2233fs)	4	0	10	linear	4	0	3	2	1
T-LBL	adult	UPN082	TP53 (p.Val73fs)	5	0	3	linear	5	0	4	2	1
T-LBL	adult	UPN082	SMARCA4 (p.Asp558Val)	5	0	3	linear	5	0	4	2	0.5
T-LBL	adult	UPN083	NOTCH1 (p.Leu1600Pro)	1	0	87	linear	1	0	0	2	1
T-LBL	adult	UPN083	CRLF2 (p.Val125Met)	1	0	87	linear	1	0	0	2	1
T-LBL	adult	UPN083	IL7R (p.Thr244_Ile245insPheGluAlaGluCysGlyGluProThr)	2	0	31	linear	2	0	1	2	1
T-LBL	adult	UPN085	LOH in 4q	1	0	97	linear	1	0	0	2	2
T-LBL	adult	UPN085	FBXW7 (p.Ser640Asn)	1	0	97	linear	1	0	0	2	0
T-LBL	adult	UPN085	PHF6 (c.375-1G>A))	1	0	97	linear	1	0	0	2	1
T-LBL	adult	UPN085	NRAS (p.Gly12Val)	2	0	90	linear	2	0	1	2	1
T-LBL	adult	UPN085	NOTCH1 (p.Phe1592_Leu1593insPro)	3	0	80	linear	3	0	2	2	1
T-LBL	adult	UPN085	NOTCH3 (p.Val1629Met)	4	0	9	linear	4	0	3	2	1
T-LBL	adult	UPN085	NOTCH1 (p.Ala1700Pro)	5	0	4	linear	5	0	4	2	0.5
T-LBL	adult	UPN086	del17p	1	0	96	linear	1	0	0	1	1
T-LBL	adult	UPN086	del in 9p	1	0	96	linear	1	0	0	1	1

Supplemental Data 3

T-LBL	adult	UPN082	19	15272336	C	CG	1848	5	1850	0.27 %	1292	169	1297	13.03 %
T-LBL	adult	UPN082	19	15276860	G	GC	2144	4	2150	0.19 %	1432	136	1437	9.46 %
T-LBL	adult	UPN082	12	49446165	A	AG	1665	2	1671	0.12 %	1779	138	1788	7.72 %
T-LBL	adult	UPN082	X	70356400	G	GC	874	0	874	0.00 %	842	60	843	7.12 %
T-LBL	adult	UPN082	4	106180913	A	G	1471	2	1477	0.14 %	631	48	679	7.07 %
T-LBL	adult	UPN082	7	150710878	C	T	1735	0	1740	0.00 %	441	30	472	6.36 %
T-LBL	adult	UPN082	12	122263254	G	GC	1698	1	1707	0.06 %	723	46	725	6.34 %
T-LBL	adult	UPN082	12	49441815	G	GC	2198	1	2200	0.05 %	897	57	900	6.33 %
T-LBL	adult	UPN082	12	122260561	A	AC	2134	3	2143	0.14 %	1904	111	1939	5.72 %
T-LBL	adult	UPN082	12	49434004	T	TG	2058	0	2069	0.00 %	674	38	679	5.60 %
T-LBL	adult	UPN082	6	135520056	C	A	1216	2	1222	0.16 %	540	32	574	5.57 %
T-LBL	adult	UPN082	9	94493291	G	GC	2246	2	2254	0.09 %	1056	57	1056	5.40 %
T-LBL	adult	UPN082	11	118344185	A	AC	1902	6	1908	0.31 %	894	48	909	5.28 %
T-LBL	adult	UPN082	19	11097624	G	GC	2022	3	2023	0.15 %	1145	58	1148	5.05 %
T-LBL	adult	UPN082	19	54652155	G	GC	1650	0	1652	0.00 %	1693	81	1696	4.78 %
T-LBL	adult	UPN082	X	70357641	G	GC	856	2	858	0.23 %	884	39	888	4.39 %
T-LBL	adult	UPN082	1	216062006	C	A	1906	0	1908	0.00 %	1021	43	1066	4.03 %
T-LBL	adult	UPN082	19	15271746	T	TG	2016	2	2024	0.10 %	1325	48	1328	3.61 %
T-LBL	adult	UPN082	17	7579470	C	CG	1746	0	1750	0.00 %	3459	73	3478	2.10 %
T-LBL	adult	UPN082	19	11106968	A	T	2031	0	2035	0.00 %	2767	37	2820	1.31 %
T-LBL	adult	UPN083	9	139399344	A	G	2105	1	2108	0.05 %	1702	1367	3075	44.46 %
T-LBL	adult	UPN083	X	1321382	C	T	1740	2	1745	0.11 %	764	568	1333	42.61 %
T-LBL	adult	UPN083	5	35874572	T	TAACCTTCGAGGCAG AGTGTGGAGAACC	1446	0	1447	0.00 %	1860	293	1875	15.63 %
T-LBL	adult	UPN085												
T-LBL	adult	UPN085	4	153244238	C	T	1360	0	1361	0.00 %	16	1300	1322	98.34 %
T-LBL	adult	UPN085	X	133527938	G	A	1095	0	1100	0.00 %	516	487	1006	48.41 %
T-LBL	adult	UPN085	1	115258747	C	A	1434	1	1437	0.07 %	866	723	1595	45.33 %
T-LBL	adult	UPN085	9	139399367	G	GGGA	1924	0	1927	0.00 %	2424	966	2429	39.77 %
T-LBL	adult	UPN085	19	15281488	C	T	1152	0	1157	0.00 %	976	44	1025	4.29 %
T-LBL	adult	UPN085	9	139397703	C	G	1866	0	1870	0.00 %	2121	43	2167	1.98 %
T-LBL	adult	UPN086												
T-LBL	adult	UPN086												

T-LBL	adult	UPN082										SNV/Indel
T-LBL	adult	UPN082										SNV/Indel
T-LBL	adult	UPN082										SNV/Indel
T-LBL	adult	UPN082										SNV/Indel
T-LBL	adult	UPN082										SNV/Indel
T-LBL	adult	UPN082										SNV/Indel
T-LBL	adult	UPN082										SNV/Indel
T-LBL	adult	UPN082										SNV/Indel
T-LBL	adult	UPN082										SNV/Indel
T-LBL	adult	UPN082										SNV/Indel
T-LBL	adult	UPN082										SNV/Indel
T-LBL	adult	UPN082										SNV/Indel
T-LBL	adult	UPN082										SNV/Indel
T-LBL	adult	UPN082										SNV/Indel
T-LBL	adult	UPN082										SNV/Indel
T-LBL	adult	UPN082										SNV/Indel
T-LBL	adult	UPN082										SNV/Indel
T-LBL	adult	UPN082										SNV/Indel
T-LBL	adult	UPN082										SNV/Indel
T-LBL	adult	UPN082										SNV/Indel
T-LBL	adult	UPN083										SNV/Indel
T-LBL	adult	UPN083										SNV/Indel
T-LBL	adult	UPN083										SNV/Indel
T-LBL	adult	UPN085	4	71471569	191154276	2	not detected	96.38 %	96.24 %	96.53 %	4491	CNV
T-LBL	adult	UPN085										SNV/Indel
T-LBL	adult	UPN085										SNV/Indel
T-LBL	adult	UPN085										SNV/Indel
T-LBL	adult	UPN085										SNV/Indel
T-LBL	adult	UPN085										SNV/Indel
T-LBL	adult	UPN085										SNV/Indel
T-LBL	adult	UPN086	17	0	24000000	1	not detected	87.24 %	86.27 %	88.22 %	1204	CNV
T-LBL	adult	UPN086	9	20005523	21909086	1	not detected	91.23 %	88.89 %	93.58 %	81	CNV

1 time point

Supplemental Data 3

T-LBL	adult	UPN086	PHF6 (p.His303Arg)	1	0	96	linear	1	0	0	1	0
T-LBL	adult	UPN086	TP53 (p.Trp91*)	1	0	96	linear	1	0	0	1	0
T-LBL	adult	UPN086	dup21q	2	0	76	linear	2	0	1	3	3
T-LBL	adult	UPN086	BCL11B (p.Glu31fs)	2	0	76	linear	2	0	1	2	1
T-LBL	adult	UPN086	NOTCH1 (p.Val1578del)	2	0	76	linear	2	0	1	2	1
T-LBL	adult	UPN086	FBXW7 (p.Trp673*)	3	0	5	linear	3	0	2	2	1
T-LBL	adult	UPN088	LOH in 1p	1	45	93	linear	1	0	0	2	2
T-LBL	adult	UPN088	LOH in 2p	1	45	93	linear	1	0	0	2	2
T-LBL	adult	UPN088	LOH4q	1	45	93	linear	1	0	0	2	2
T-LBL	adult	UPN088	KRAS (p.Gly12Arg)	2	33	86	linear	2	0	1	2	1
T-LBL	adult	UPN088	dup9p	3	0	17	linear	3	0	2	3	3
T-LBL	adult	UPN089	PHF6 (p.Tyr302fs)	1	0	20	linear	1	0	0	1	0
T-LBL	adult	UPN089	NOTCH1 (p.Leu1600Pro)	1	0	20	linear	1	0	0	2	1
T-LBL	adult	UPN089	CCND3 (p.Ala250fs)	2	0	11	linear	2	0	1	2	1
T-LBL	adult	UPN089	BCL11B (p.Lys838Arg)	2	0	11	linear	2	0	1	2	1
T-LBL	adult	UPN091	del in 9p	1	0	94	linear	1	0	0	1	1
T-LBL	adult	UPN091	PHF6 (p.Cys284Arg)	1	0	94	linear	1	0	0	1	0
T-LBL	adult	UPN091	FBXW7 (p.Arg505Cys)	1	0	94	linear	1	0	0	2	1
T-LBL	adult	UPN091	USP7 (p.Asp198fs)	1	0	94	linear	1	0	0	2	1
T-LBL	adult	UPN091	FBXW7 (p.Ser476Gly)	2	0	86	linear	2	0	1	2	0
T-LBL	adult	UPN091	NOTCH1 (p.Ser1690_Cys1692del)	3	0	17	linear	3	0	2	2	1
T-LBL	adult	UPN091	NOTCH1 (p.Gln1733_Leu1734insGlnLeuGluProProProProAlaGln)	4	0	7	linear	4	0	3	2	0.5
T-LBL	adult	UPN092	dup in 8q	1	0	94	linear	1	0	0	3	3
T-LBL	adult	UPN092	BCL11B (p.Arg4Pro)	1	0	94	linear	1	0	0	2	1
T-LBL	adult	UPN092	del in 1p	2	0	86	linear	2	0	1	1	1
T-LBL	adult	UPN092	del in 6q	2	0	86	linear	2	0	1	1	1
T-LBL	adult	UPN092	LOH in 19p	2	0	86	linear	2	0	1	2	2
T-LBL	adult	UPN092	JAK3 (p.Met511Ile)	2	0	86	linear	2	0	1	2	0
T-LBL	adult	UPN092	JAK3 (p.Ala573Val)	2	0	86	linear	2	0	1	2	0
T-LBL	adult	UPN092	MED12 (p.Val760fs)	2	0	86	linear	2	0	1	2	1
T-LBL	adult	UPN092	dup in 1p	3	0	76	linear	3	0	2	3	3

1 time point

T-LBL	adult	UPN086	X	133551269	A	G	651	1	655	0.15 %	42	771	814	94.72 %
T-LBL	adult	UPN086	17	7579415	C	T	1229	0	1231	0.00 %	24	384	408	94.12 %
T-LBL	adult	UPN086												
T-LBL	adult	UPN086	14	99724135	CGTCTTCT	GGGATG	1460	6	1465	0.41 %	729	461	1210	38.10 %
T-LBL	adult	UPN086	9	139399408	GCAC	G	1603	5	1612	0.31 %	809	518	1331	38.92 %
T-LBL	adult	UPN086	4	153244138	C	T	1568	0	1570	0.00 %	2173	52	2227	2.33 %
T-LBL	adult	UPN088												
T-LBL	adult	UPN088												
T-LBL	adult	UPN088												
T-LBL	adult	UPN088	12	25398285	C	G	1040	209	1249	16.73 %	797	605	1404	43.09 %
T-LBL	adult	UPN088												
T-LBL	adult	UPN089	X	133551265	T	TA	660	0	662	0.00 %	500	91	501	18.16 %
T-LBL	adult	UPN089	9	139399344	A	G	3187	2	3193	0.06 %	1732	201	1940	10.36 %
T-LBL	adult	UPN089	6	41903805	AGTG	ATTTTCTAATTGGGCGGC	3041	13	3051	0.43 %	1562	105	1671	6.28 %
T-LBL	adult	UPN089	14	99640660	T	C	2586	0	2599	0.00 %	1515	78	1597	4.88 %
T-LBL	adult	UPN091												
T-LBL	adult	UPN091	X	133551211	T	C	441	0	441	0.00 %	18	308	327	94.19 %
T-LBL	adult	UPN091	4	153247289	G	A	1342	0	1344	0.00 %	717	632	1352	46.75 %
T-LBL	adult	UPN091	16	9014234	TC	T	1250	0	1252	0.00 %	431	375	807	46.47 %
T-LBL	adult	UPN091	4	153247376	T	C	1014	2	1016	0.20 %	509	391	902	43.35 %
T-LBL	adult	UPN091	9	139397723	AAGCACTGCG	A	1962	0	1963	0.00 %	1024	96	1121	8.56 %
T-LBL	adult	UPN091	9	139396907	A	AGCTGCGCCGGCGGGGGCGGCTCCAATT	1121	0	1122	0.00 %	589	21	593	3.54 %
T-LBL	adult	UPN092												
T-LBL	adult	UPN092	14	99737545	C	G	786	2	792	0.25 %	662	562	1229	45.73 %
T-LBL	adult	UPN092												
T-LBL	adult	UPN092												
T-LBL	adult	UPN092												
T-LBL	adult	UPN092	19	17949108	C	A	1214	13	1228	1.06 %	109	815	925	88.11 %
T-LBL	adult	UPN092	19	17948006	G	A	1165	23	1190	1.93 %	134	865	1000	86.50 %
T-LBL	adult	UPN092	X	70345251	GGT	G	1192	9	1205	0.75 %	859	626	1487	42.10 %
T-LBL	adult	UPN092												

T-LBL	adult	UPN086										SNV/Indel
T-LBL	adult	UPN086										SNV/Indel
T-LBL	adult	UPN086	21	14359894	44479208	3	not detected	71.76 %	69.49 %	74.02 %	1449	CNV
T-LBL	adult	UPN086										SNV/Indel
T-LBL	adult	UPN086										SNV/Indel
T-LBL	adult	UPN086										SNV/Indel
T-LBL	adult	UPN088	1	0	29562685	2	not detected	92.32 %	92.02 %	92.61 %	1266	CNV
T-LBL	adult	UPN088	2	0	74985347	2	not detected	90.77 %	90.27 %	91.27 %	3315	CNV
T-LBL	adult	UPN088	4	55788718	191154276	2	not detected	93.36 %	93.25 %	93.47 %	4814	CNV
T-LBL	adult	UPN088										SNV/Indel
T-LBL	adult	UPN088	9	0	47300000	3	not detected	16.81 %	16.06 %	17.56 %	1895	CNV
T-LBL	adult	UPN089										SNV/Indel
T-LBL	adult	UPN089										SNV/Indel
T-LBL	adult	UPN089										SNV/Indel
T-LBL	adult	UPN089										SNV/Indel
T-LBL	adult	UPN091	9	0	35962463	2	not detected	95.09 %	94.74 %	95.45 %	1918	CNV
T-LBL	adult	UPN091										SNV/Indel
T-LBL	adult	UPN091										SNV/Indel
T-LBL	adult	UPN091										SNV/Indel
T-LBL	adult	UPN091										SNV/Indel
T-LBL	adult	UPN091										SNV/Indel
T-LBL	adult	UPN091										SNV/Indel
T-LBL	adult	UPN092	8	90136687	146364022	3	not detected	95.18 %	92.69 %	97.68 %	2251	CNV
T-LBL	adult	UPN092										SNV/Indel
T-LBL	adult	UPN092	1	0	35315737	1	not detected	85.99 %	85.42 %	86.57 %	1530	CNV
T-LBL	adult	UPN092	6	140169779	171115067	1	not detected	84.27 %	83.60 %	84.94 %	1435	CNV
T-LBL	adult	UPN092	19	0	3119534	2	not detected	87.19 %	84.71 %	89.67 %	191	CNV
T-LBL	adult	UPN092										SNV/Indel
T-LBL	adult	UPN092										SNV/Indel
T-LBL	adult	UPN092										SNV/Indel
T-LBL	adult	UPN092	1	35315504	73070449	3	not detected	76.07 %	73.70 %	78.45 %	1479	CNV

Supplemental Data 3

T-LBL	adult	UPN093	del in 1p	1	0	97	linear	1	0	0	1	1
T-LBL	adult	UPN093	del in 4p	1	0	97	linear	1	0	0	1	1
T-LBL	adult	UPN093	del in 8q	1	0	97	linear	1	0	0	1	1
T-LBL	adult	UPN093	del in 13q	1	0	97	linear	1	0	0	1	1
T-LBL	adult	UPN093	PHF6 (p.Arg226*)	1	0	97	linear	1	0	0	1	0
T-LBL	adult	UPN093	NOTCH1 (p.Leu1593Pro)	2	0	90	linear	2	0	1	2	1
T-LBL	adult	UPN093	USH2A (p.Arg2323*)	2	0	90	linear	2	0	1	2	1
T-LBL	adult	UPN093	NOTCH1 (p.Leu2472fs)	3	0	66	linear	3	0	2	2	0.5
T-LBL	adult	UPN093	dup9p	4	0	19	linear	4	0	3	3	3
T-LBL	adult	UPN093	NOTCH1 (p.Leu1600Pro)	5	0	4	linear	5	0	4	2	0
T-LBL	adult	UPN094	FBXW7 (p.Gly423Val)	1	0	9	linear	1	0	0	2	1
T-LBL	adult	UPN094	JAK3 (p.Leu857Pro)	1	0	9	linear	1	0	0	2	1
T-LBL	adult	UPN094	JAK1 (p.Pro960His)	2	0	4	linear	2	0	1	2	1
T-LBL	adult	UPN094	NOTCH1 (p.Ile1680Asn)	2	0	4	linear	2	0	1	2	1
T-LBL	adult	UPN096	LOH9p	1	0	100	linear	1	0	0	2	2
T-LBL	adult	UPN096	del in 14q	1	0	100	linear	1	0	0	1	1
T-LBL	adult	UPN096	NOTCH1 (p.Gln2394*)	1	0	100	linear	1	0	0	2	0.5
T-LBL	adult	UPN096	NOTCH1 (p.Leu1593Pro)	1	0	100	linear	1	0	0	2	0.5
T-LBL	adult	UPN096	KDM6A (p.Phe1116fs)	2	0	94	linear	2	0	1	1	0
T-LBL	adult	UPN096	DNM2 (p.Gly358Arg)	2	0	94	linear	2	0	1	2	1
T-LBL	adult	UPN096	del in 1p	3	0	85	linear	3	0	2	1	1
T-LBL	adult	UPN096	dup in 8q	3	0	85	linear	3	0	2	3	3
T-LBL	adult	UPN096	DDX3X (p.Val496Glu)	4	0	74	linear	4	0	3	1	0
T-LBL	adult	UPN096	DDX3X (p.Pro568Leu)	5	0	20	linear	5	0	4	1	0
T-LBL	adult	UPN097	LOH in 1q	1	100	100	linear	1	0	0	2	2
T-LBL	adult	UPN097	LOH in 3p	1	100	100	linear	1	0	0	2	2
T-LBL	adult	UPN097	LOH in 5q	1	100	100	linear	1	0	0	2	2
T-LBL	adult	UPN097	LOH in 8q	1	100	100	linear	1	0	0	2	2
T-LBL	adult	UPN097	LOH in 8q	1	100	100	linear	1	0	0	2	2
T-LBL	adult	UPN097	LOH in 9p	1	100	100	linear	1	0	0	2	2
T-LBL	adult	UPN097	LOH in 9q	1	100	100	linear	1	0	0	2	2
T-LBL	adult	UPN097	LOH in 9q	1	100	100	linear	1	0	0	2	2
T-LBL	adult	UPN097	LOH in 10q	1	100	100	linear	1	0	0	2	2
T-LBL	adult	UPN097	LOH in 11q	1	100	100	linear	1	0	0	2	2
T-LBL	adult	UPN097	LOH in 15q	1	100	100	linear	1	0	0	2	2

1 time point

T-LBL	adult	UPN093												
T-LBL	adult	UPN093												
T-LBL	adult	UPN093												
T-LBL	adult	UPN093												
T-LBL	adult	UPN093	X	133547940	C	T	615	2	617	0.32 %	5	523	528	99.05 %
T-LBL	adult	UPN093	9	139399365	A	G	1889	1	1894	0.05 %	828	709	1539	46.07 %
T-LBL	adult	UPN093	1	216138812	G	A	1043	0	1044	0.00 %	527	408	938	43.50 %
T-LBL	adult	UPN093	9	139390777	G	GAGGTTCCC	1715	0	1720	0.00 %	1240	412	1240	33.23 %
T-LBL	adult	UPN093												
T-LBL	adult	UPN093	9	139399344	A	G	1940	0	1943	0.00 %	1563	34	1600	2.13 %
T-LBL	adult	UPN094	4	153249510	C	A	1720	0	1724	0.00 %	1801	98	1903	5.15 %
T-LBL	adult	UPN094	19	17943438	A	G	2115	1	2119	0.05 %	1923	84	2010	4.18 %
T-LBL	adult	UPN094	1	65304236	G	T	1675	3	1680	0.18 %	1687	47	1736	2.71 %
T-LBL	adult	UPN094	9	139397762	A	T	2077	2	2083	0.10 %	2149	33	2186	1.51 %
T-LBL	adult	UPN096												
T-LBL	adult	UPN096												
T-LBL	adult	UPN096	9	139391011	G	A	2005	0	2008	0.00 %	1058	1067	2128	50.14 %
T-LBL	adult	UPN096	9	139399365	A	G	1927	1	1939	0.05 %	1046	980	2030	48.28 %
T-LBL	adult	UPN096	X	44942747	TGTGCGTGTCGTATC	GGGGGGCAAACGG	548	4	548	0.73 %	9	253	269	94.05 %
T-LBL	adult	UPN096	19	10904475	G	A	1421	2	1427	0.14 %	715	643	1365	47.11 %
T-LBL	adult	UPN096												
T-LBL	adult	UPN096												
T-LBL	adult	UPN096	X	41205653	T	A	585	0	586	0.00 %	160	454	614	73.94 %
T-LBL	adult	UPN096	X	41206199	C	T	628	0	630	0.00 %	655	72	727	9.90 %
T-LBL	adult	UPN097												
T-LBL	adult	UPN097												
T-LBL	adult	UPN097												
T-LBL	adult	UPN097												
T-LBL	adult	UPN097												
T-LBL	adult	UPN097												
T-LBL	adult	UPN097												
T-LBL	adult	UPN097												
T-LBL	adult	UPN097												
T-LBL	adult	UPN097												
T-LBL	adult	UPN097												

Supplemental Data 3

T-LBL	adult	UPN093	1	23007736	24847715	1	not detected	96.39 %	95.89 %	96.89 %	84	CNV
T-LBL	adult	UPN093	4	30380358	32140465	1	not detected	96.12 %	94.93 %	97.30 %	60	CNV
T-LBL	adult	UPN093	8	117202621	118408588	1	not detected	94.99 %	91.52 %	98.46 %	48	CNV
T-LBL	adult	UPN093	13	50079991	51382899	1	not detected	95.67 %	94.01 %	97.33 %	45	CNV
T-LBL	adult	UPN093										SNV/Indel
T-LBL	adult	UPN093										SNV/Indel
T-LBL	adult	UPN093										SNV/Indel
T-LBL	adult	UPN093										SNV/Indel
T-LBL	adult	UPN093	9	0	47300000	3	not detected	19.01 %	18.11 %	19.92 %	2062	CNV
T-LBL	adult	UPN093										SNV/Indel
T-LBL	adult	UPN094										SNV/Indel
T-LBL	adult	UPN094										SNV/Indel
T-LBL	adult	UPN094										SNV/Indel
T-LBL	adult	UPN094										SNV/Indel
T-LBL	adult	UPN096	9	0	40786873	2	not detected	98.21 %	97.75 %	98.68 %	1818	CNV
T-LBL	adult	UPN096	14	22087235	22989590	1	not detected	97.87 %	95.55 %	100.19 %	75	CNV
T-LBL	adult	UPN096										SNV/Indel
T-LBL	adult	UPN096										SNV/Indel
T-LBL	adult	UPN096										SNV/Indel
T-LBL	adult	UPN096										SNV/Indel
T-LBL	adult	UPN096	1	5925692	6895859	1	not detected	82.60 %	79.96 %	85.25 %	28	CNV
T-LBL	adult	UPN096	8	100625908	146364022	3	not detected	87.48 %	86.27 %	88.70 %	1841	CNV
T-LBL	adult	UPN096										SNV/Indel
T-LBL	adult	UPN096										SNV/Indel
T-LBL	adult	UPN097	1	164652404	215890201	2	100.00 %	100.00 %				CNV
T-LBL	adult	UPN097	3	22148081	38061659	2	100.00 %	100.00 %				CNV
T-LBL	adult	UPN097	5	168835809	180915260	2	100.00 %	100.00 %				CNV
T-LBL	adult	UPN097	8	96688963	106098919	2	100.00 %	100.00 %				CNV
T-LBL	adult	UPN097	8	125954377	132620252	2	100.00 %	100.00 %				CNV
T-LBL	adult	UPN097	9	17624108	49000000	2	100.00 %	100.00 %				CNV
T-LBL	adult	UPN097	9	75666355	101681167	2	100.00 %	100.00 %				CNV
T-LBL	adult	UPN097	9	136460765	141213430	2	100.00 %	100.00 %				CNV
T-LBL	adult	UPN097	10	112153834	114226900	2	100.00 %	100.00 %				CNV
T-LBL	adult	UPN097	11	106531021	111038617	2	100.00 %	100.00 %				CNV
T-LBL	adult	UPN097	15	98457055	101694349	2	100.00 %	100.00 %				CNV

1 time point

T-LBL	adult	UPN097	LOH in 20p	1	100	100	linear	1	0	0	2	2
T-LBL	adult	UPN097	LOH in 2q	1	100	100	linear	1	0	0	2	2
T-LBL	adult	UPN097	del in 1p	2	0	87	linear	2	0	1	1	1
T-LBL	adult	UPN097	del in 9p	2	0	87	linear	2	0	1	1	1
T-LBL	adult	UPN097	dup in 15q	2	0	87	linear	2	0	1	3	3
T-LBL	adult	UPN097	del in 19q	2	0	87	linear	2	0	1	1	1
T-LBL	adult	UPN097	JAK3 (p.Met511Ile)	2	0	87	linear	2	0	1	2	1
T-LBL	adult	UPN097	dup in 1p	3	0	74	linear	3	0	2	3	3
T-LBL	adult	UPN097	dup in 1p	3	0	74	linear	3	0	2	3	3
T-LBL	adult	UPN097	dup in 9q	3	0	74	linear	3	0	2	3	3
T-LBL	adult	UPN097	dup in 9q	3	0	74	linear	3	0	2	3	3
T-LBL	adult	UPN097	NOTCH1 (p.Gln1614_Tyr1620delinsProLeu)	4	0	56	linear	4	0	3	2	1
T-LBL	adult	UPN097	JAK1 (p.Ser703Ile)	4	0	56	linear	4	0	3	2	1
T-LBL	adult	UPN097	RUNX1 (p.Asp126fs)	5	0	42	linear	5	0	4	2	1
T-LBL	adult	UPN098	del in 5q	1	0	94	linear	1	0	0	1	1
T-LBL	adult	UPN098	del in 1p	1	0	94	linear	1	0	0	1	1
T-LBL	adult	UPN098	LOH2p	1	0	94	linear	1	0	0	2	2
T-LBL	adult	UPN098	ROR2 (p.Arg242Cys)	1	0	94	linear	1	0	0	2	1
T-LBL	adult	UPN098	del in 9p	2	0	84	linear	2	0	1	1	1
T-LBL	adult	UPN098	EZH2 (p.Gly159Trp)	2	0	84	linear	2	0	1	2	1
T-LBL	adult	UPN098	dup in 17q	3	0	74	linear	3	0	2	3	3
T-LBL	adult	UPN098	BCL11B (p.Gly631Arg)	4	0	40	linear	4	0	3	2	1
T-LBL	adult	UPN098	EZH2 (p.Arg347Gln)	4	0	40	linear	4	0	3	2	0.5
T-LBL	adult	UPN098	IKZF1 (p.Arg69His)	4	0	40	linear	4	0	3	2	1
T-LBL	adult	UPN098	FBXW7 (p.Arg505Cys)	4	0	40	linear	4	0	3	2	1
T-LBL	adult	UPN098	FBXW7 (p.Arg689Gln)	5	0	29	linear	5	0	4	2	0.125
T-LBL	adult	UPN098	USH2A (p.Ala1267Val)	5	0	29	linear	5	0	4	2	1
T-LBL	adult	UPN098	ZBTB7A (p.Gly273Ser)	5	0	29	linear	5	0	4	2	1
T-LBL	adult	UPN098	FBXW7 (p.Arg278Gln)	5	0	29	linear	5	0	4	2	0.125
T-LBL	adult	UPN098	FBXW7 (p.Arg465His)	5	0	29	linear	5	0	4	2	0.125
T-LBL	adult	UPN098	KIT (p.Arg49His)	5	0	29	linear	5	0	4	2	1
T-LBL	adult	UPN098	EZH2 (p.Arg418Gln)	5	0	29	linear	5	0	4	2	0.25
T-LBL	adult	UPN098	NOTCH1 (p.Ile1616Asn)	6	0	18	linear	6	0	5	2	0.5

1 time point

T-LBL	adult	UPN097												
T-LBL	adult	UPN097												
T-LBL	adult	UPN097												
T-LBL	adult	UPN097												
T-LBL	adult	UPN097												
T-LBL	adult	UPN097												
T-LBL	adult	UPN097	19	17949108	C	T	1489	0	1491	0.00 %	919	673	1599	42.09 %
T-LBL	adult	UPN097												
T-LBL	adult	UPN097												
T-LBL	adult	UPN097												
T-LBL	adult	UPN097												
T-LBL	adult	UPN097	9	139399283	GTAGTAGGGGAAGATCATCT	AAGAG	1541	16	1557	1.03 %	1655	676	2347	28.80 %
T-LBL	adult	UPN097	1	65311203	C	A	1292	1	1294	0.08 %	1433	543	1979	27.44 %
T-LBL	adult	UPN097	21	36252987	T	TGGAACAGGGCC	1269	0	1271	0.00 %	1515	326	1521	21.43 %
T-LBL	adult	UPN098												
T-LBL	adult	UPN098												
T-LBL	adult	UPN098												
T-LBL	adult	UPN098	9	94495617	G	A	1776	2	1781	0.11 %	223	199	422	47.16 %
T-LBL	adult	UPN098												
T-LBL	adult	UPN098	7	148526829	C	A	750	3	756	0.40 %	115	81	197	41.12 %
T-LBL	adult	UPN098												
T-LBL	adult	UPN098	14	99641282	C	T	294	0	295	0.00 %	114	32	147	21.77 %
T-LBL	adult	UPN098	7	148515169	C	T	1018	1	1021	0.10 %	274	75	351	21.37 %
T-LBL	adult	UPN098	7	50444276	G	A	1078	0	1085	0.00 %	359	88	449	19.60 %
T-LBL	adult	UPN098	4	153247289	G	A	1230	0	1231	0.00 %	357	86	447	19.24 %
T-LBL	adult	UPN098	4	153244091	C	T	1031	0	1034	0.00 %	261	53	314	16.88 %
T-LBL	adult	UPN098	1	216372980	G	A	806	1	808	0.12 %	161	31	192	16.15 %
T-LBL	adult	UPN098	19	4054414	C	T	367	1	370	0.27 %	115	22	137	16.06 %
T-LBL	adult	UPN098	4	153258982	C	T	858	0	858	0.00 %	252	46	298	15.44 %
T-LBL	adult	UPN098	4	153249384	C	T	1014	0	1017	0.00 %	337	61	398	15.33 %
T-LBL	adult	UPN098	4	55561756	G	A	1238	0	1240	0.00 %	364	64	428	14.95 %
T-LBL	adult	UPN098	7	148514471	C	T	673	0	679	0.00 %	246	36	284	12.68 %
T-LBL	adult	UPN098	9	139399296	A	T	1428	0	1436	0.00 %	594	69	664	10.39 %

T-LBL	adult	UPN097	20	0	19884606	2	100.00 %	100.00 %				CNV
T-LBL	adult	UPN097	2	229503575	235638976	2	100.00 %	100.00 %				CNV
T-LBL	adult	UPN097	1	85905534	108762158	1	not detected	87.48 %	87.02 %	87.93 %	773	CNV
T-LBL	adult	UPN097	9	0	39140379	1	not detected	89.43 %	89.04 %	89.83 %	1027	CNV
T-LBL	adult	UPN097	15	53901900	54420787	3	not detected	93.44 %	86.80 %	100.09 %	57	CNV
T-LBL	adult	UPN097	19	53390716	55687462	1	not detected	87.55 %	85.94 %	89.16 %	197	CNV
T-LBL	adult	UPN097										SNV/Indel
T-LBL	adult	UPN097	1	35436232	76387705	3	not detected	74.73 %	73.42 %	76.04 %	1429	CNV
T-LBL	adult	UPN097	1	108952455	110760120	3	not detected	74.83 %	68.99 %	80.66 %	82	CNV
T-LBL	adult	UPN097	9	49000001	75666354	3	not detected	70.92 %	67.23 %	74.62 %	173	CNV
T-LBL	adult	UPN097	9	101681168	136460764	3	not detected	75.45 %	74.19 %	76.70 %	1395	CNV
T-LBL	adult	UPN097										SNV/Indel
T-LBL	adult	UPN097										SNV/Indel
T-LBL	adult	UPN097										SNV/Indel
T-LBL	adult	UPN098	5	131457395	180915260	1	not detected	91.38 %	90.83 %	91.92 %	2072	CNV
T-LBL	adult	UPN098	1	0	28643858	1	not detected	91.41 %	90.68 %	92.14 %	1373	CNV
T-LBL	adult	UPN098	2	0	93300000	2	not detected	95.45 %	95.18 %	95.71 %	3809	CNV
T-LBL	adult	UPN098										SNV/Indel
T-LBL	adult	UPN098	9	0	34520001	1	not detected	86.44 %	85.53 %	87.36 %	1788	CNV
T-LBL	adult	UPN098										SNV/Indel
T-LBL	adult	UPN098	17	42287433	81151539	3	not detected	74.42 %	72.02 %	76.82 %	1630	CNV
T-LBL	adult	UPN098										SNV/Indel
T-LBL	adult	UPN098										SNV/Indel
T-LBL	adult	UPN098										SNV/Indel
T-LBL	adult	UPN098										SNV/Indel
T-LBL	adult	UPN098										SNV/Indel
T-LBL	adult	UPN098										SNV/Indel
T-LBL	adult	UPN098										SNV/Indel
T-LBL	adult	UPN098										SNV/Indel
T-LBL	adult	UPN098										SNV/Indel
T-LBL	adult	UPN098										SNV/Indel
T-LBL	adult	UPN098										SNV/Indel
T-LBL	adult	UPN098										SNV/Indel

Supplemental Data 3

T-LBL	adult	UPN098	NOTCH1 (p.Gln2519*)	6	0	18	linear	6	0	5	2	0.5
T-LBL	adult	UPN098	NOS3 (p.Leu289Val)	6	0	18	linear	6	0	5	2	1
T-LBL	adult	UPN098	NOS3 (p.Pro592Ala)	7	0	10	linear	7	0	6	2	0
T-LBL	adult	UPN098	ZBTB7A (p.Val119Met)	7	0	10	linear	7	0	6	2	0.5
T-LBL	adult	UPN098	MYB (p.Gln424His)	7	0	10	linear	7	0	6	2	1
T-LBL	adult	UPN098	NOS3 (p.Val620Ala)	7	0	10	linear	7	0	6	2	0
T-LBL	adult	UPN100	LOH in 1p	1	100	100	linear	1	0	0	2	2
T-LBL	adult	UPN100	LOH in 10q	1	100	100	linear	1	0	0	2	2
T-LBL	adult	UPN100	LOH in 12q	1	100	100	linear	1	0	0	2	2
T-LBL	adult	UPN100	LOH in 15q	1	100	100	linear	1	0	0	2	2
T-LBL	adult	UPN100	LOH in 17q	2	0	94	linear	2	0	1	2	2
T-LBL	adult	UPN100	STAT5B (p.Asn642His)	2	0	94	linear	2	0	1	2	0
T-LBL	adult	UPN100	NOTCH1 (p.Leu2482fs)	3	0	89	linear	3	0	2	2	1
T-LBL	adult	UPN100	JAK3 (p.Val674Ala)	4	0	77	linear	4	0	3	2	1
T-LBL	adult	UPN100	JAK1 (p.Asp899Glu)	5	0	28	linear	5	0	4	2	1
T-LBL	adult	UPN100	PIK3CD (p.Glu1045Lys)	5	0	28	linear	5	0	4	2	1
T-LBL	adult	UPN100	STAT5B (p.Gly698Val)	6	0	17	linear	6	0	5	2	1
T-LBL	adult	UPN102	del in 4q	1	0	97	linear	1	0	0	1	1
T-LBL	adult	UPN102	del in 9p	1	0	97	linear	1	0	0	1	1
T-LBL	adult	UPN102	del in 16q	1	0	97	linear	1	0	0	1	1
T-LBL	adult	UPN102	dup in 20q	1	0	97	linear	1	0	0	3	3
T-LBL	adult	UPN102	FBXW7 (p.His460Asn)	1	0	97	linear	1	0	0	1	0
T-LBL	adult	UPN102	USP7 (p.Gln1031fs)	2	0	88	linear	2	0	1	2	1
T-LBL	adult	UPN102	dup in 1p	3	0	82	linear	3	0	2	3	3
T-LBL	adult	UPN102	dup in 11q	3	0	82	linear	3	0	2	3	3
T-LBL	adult	UPN102	NOTCH1 (p.Val1578Glu)	4	0	63	linear	4	0	3	2	1
T-LBL	adult	UPN103	del in 1p	1	0	98	linear	1	0	0	1	1
T-LBL	adult	UPN103	LOH in 17q	1	0	98	linear	1	0	0	2	2
T-LBL	adult	UPN103	STAT5B (p.Asn642His)	1	0	98	linear	1	0	0	2	0
T-LBL	adult	UPN103	NOTCH1 (p.Val1576Glu)	1	0	98	linear	1	0	0	2	1
T-LBL	adult	UPN103	USP7 (p.Val952fs)	2	0	91	linear	2	0	1	2	1
T-LBL	adult	UPN103	LOH4p, LOH in 4q	3	0	62	linear	3	0	2	2	2
T-LBL	adult	UPN103	LOH in 1p, LOH 1q	4	0	49	linear	4	0	3	2	2
T-LBL	adult	UPN103	dup2	4	0	49	linear	4	0	3	3	3
T-LBL	adult	UPN103	LOH3	4	0	49	linear	4	0	3	2	2

1 time point

T-LBL	adult	UPN098	9	139390636	G	A	1371	0	1374	0.00 %	465	49	514	9.53 %
T-LBL	adult	UPN098	7	150708877	C	G	816	2	820	0.24 %	466	44	513	8.58 %
T-LBL	adult	UPN098	7	150700420	C	G	841	0	842	0.00 %	716	53	777	6.82 %
T-LBL	adult	UPN098	19	4054876	C	T	1462	1	1465	0.07 %	373	25	400	6.25 %
T-LBL	adult	UPN098	6	135518167	A	T	1237	0	1243	0.00 %	460	26	487	5.34 %
T-LBL	adult	UPN098	7	150700345	T	C	780	0	786	0.00 %	893	32	925	3.46 %
T-LBL	adult	UPN100												
T-LBL	adult	UPN100												
T-LBL	adult	UPN100												
T-LBL	adult	UPN100												
T-LBL	adult	UPN100												
T-LBL	adult	UPN100	17	40359729	T	G	1188	0	1191	0.00 %	33	552	585	94.36 %
T-LBL	adult	UPN100	9	139390746	AG	A	2203	0	2205	0.00 %	567	460	1029	44.70 %
T-LBL	adult	UPN100	19	17945918	A	G	2432	1	2436	0.04 %	531	338	872	38.76 %
T-LBL	adult	UPN100	1	65305431	G	T	1956	1	1963	0.05 %	814	132	948	13.92 %
T-LBL	adult	UPN100	1	9787030	G	A	1805	2	1809	0.11 %	775	124	901	13.76 %
T-LBL	adult	UPN100	17	40354811	C	A	2142	1	2143	0.05 %	888	86	975	8.82 %
T-LBL	adult	UPN102												
T-LBL	adult	UPN102												
T-LBL	adult	UPN102												
T-LBL	adult	UPN102												
T-LBL	adult	UPN102	4	153249400	G	T	1209	1	1210	0.08 %	74	981	1059	92.63 %
T-LBL	adult	UPN102	16	8988661	G	GGCTC	1417	0	1419	0.00 %	1950	869	1966	44.20 %
T-LBL	adult	UPN102												
T-LBL	adult	UPN102												
T-LBL	adult	UPN102	9	139399410	A	T	1606	0	1612	0.00 %	2894	1348	4252	31.70 %
T-LBL	adult	UPN103												
T-LBL	adult	UPN103												
T-LBL	adult	UPN103	17	40359729	T	G	1226	2	1231	0.16 %	19	1287	1307	98.47 %
T-LBL	adult	UPN103	9	139399416	A	T	2622	0	2627	0.00 %	1175	1359	2538	53.55 %
T-LBL	adult	UPN103	16	8989563	A	AC	1415	0	1422	0.00 %	1383	638	1393	45.80 %
T-LBL	adult	UPN103												
T-LBL	adult	UPN103												
T-LBL	adult	UPN103												
T-LBL	adult	UPN103												

Supplemental Data 3

T-LBL	adult	UPN098										SNV/Indel
T-LBL	adult	UPN098										SNV/Indel
T-LBL	adult	UPN098										SNV/Indel
T-LBL	adult	UPN098										SNV/Indel
T-LBL	adult	UPN098										SNV/Indel
T-LBL	adult	UPN098										SNV/Indel
T-LBL	adult	UPN100	1	81229731	95027807	2	100.00 %	100.00 %				CNV
T-LBL	adult	UPN100	10	52094428	92911616	2	100.00 %	100.00 %				CNV
T-LBL	adult	UPN100	12	109029564	119965332	2	100.00 %	100.00 %				CNV
T-LBL	adult	UPN100	15	97265923	102531392	2	100.00 %	100.00 %				CNV
T-LBL	adult	UPN100	17	38238839	81195210	2	not detected	94.64 %	94.04 %	95.24 %	1436	CNV
T-LBL	adult	UPN100										SNV/Indel
T-LBL	adult	UPN100										SNV/Indel
T-LBL	adult	UPN100										SNV/Indel
T-LBL	adult	UPN100										SNV/Indel
T-LBL	adult	UPN100										SNV/Indel
T-LBL	adult	UPN100										SNV/Indel
T-LBL	adult	UPN102	4	152985445	153262993	1	not detected	97.78 %	96.81 %	98.75 %	17	CNV
T-LBL	adult	UPN102	9	20623104	39140379	1	not detected	94.36 %	93.19 %	95.54 %	702	CNV
T-LBL	adult	UPN102	16	53862949	54090520	1	not detected	97.03 %	96.13 %	97.93 %	25	CNV
T-LBL	adult	UPN102	20	29432371	31970929	3	not detected	115.57 %	101.98 %	129.15 %	69	CNV
T-LBL	adult	UPN102										SNV/Indel
T-LBL	adult	UPN102										SNV/Indel
T-LBL	adult	UPN102	1	61553815	64144400	3	not detected	83.59 %	79.08 %	88.09 %	118	CNV
T-LBL	adult	UPN102	11	127411099	129408918	3	not detected	81.48 %	76.81 %	86.15 %	98	CNV
T-LBL	adult	UPN102										SNV/Indel
T-LBL	adult	UPN103	1	9113226	10495556	1	not detected	94.07 %	91.06 %	97.07 %	57	CNV
T-LBL	adult	UPN103	17	38561623	81195210	2	not detected	98.43 %	98.21 %	98.65 %	1618	CNV
T-LBL	adult	UPN103										SNV/Indel
T-LBL	adult	UPN103										SNV/Indel
T-LBL	adult	UPN103										SNV/Indel
T-LBL	adult	UPN103	4	0	109047057	2	not detected	61.74 %	61.33 %	62.14 %	3800	CNV
T-LBL	adult	UPN103	1	10495557	249250620	2	not detected	49.67 %	49.37 %	49.96 %	8500	CNV
T-LBL	adult	UPN103	2	0	243199373	2	not detected	49.80 %	49.53 %	50.06 %	9716	CNV
T-LBL	adult	UPN103	3	0	198022430	2	not detected	49.77 %	49.48 %	50.06 %	7761	CNV

1 time point

Supplemental Data 3

T-LBL	adult	UPN103	LOH5	4	0	49	linear	4	0	3	2	2
T-LBL	adult	UPN103	LOH8	4	0	49	linear	4	0	3	2	2
T-LBL	adult	UPN103	LOH10	4	0	49	linear	4	0	3	2	2
T-LBL	adult	UPN103	LOH12	4	0	49	linear	4	0	3	2	2
T-LBL	adult	UPN103	LOH15	4	0	49	linear	4	0	3	2	2
T-LBL	adult	UPN103	LOHX	4	0	49	linear	4	0	3	2	2
T-LBL	adult	UPN103	dup in 4q	5	0	29	linear	5	0	4	3	3
T-LBL	adult	UPN103	dup9p	6	0	19	linear	6	0	5	3	3
T-LBL	adult	UPN107	FBXW7 (p.Arg689Trp)	1	0	93	linear	1	0	0	3	1
T-LBL	adult	UPN107	del in 7q	2	0	86	linear	2	0	1	1	1
T-LBL	adult	UPN107	PHF6 (p.Arg129*)	2	0	86	linear	2	0	1	2	1
T-LBL	adult	UPN107	del in 1p	3	0	76	linear	3	0	2	1	1
T-LBL	adult	UPN107	SUZ12 (p.Ser249Pro)	3	0	76	linear	3	0	2	2	1
T-LBL	adult	UPN107	del in 1p	4	0	70	linear	4	0	3	1	1
T-LBL	adult	UPN107	del in 7p	4	0	70	linear	4	0	3	1	1
T-LBL	adult	UPN107	del in 13q	5	0	60	linear	5	0	4	1	1
T-LBL	adult	UPN107	dup8	5	0	60	linear	5	0	4	3	3
T-LBL	adult	UPN107	dup4	5	0	60	linear	5	0	4	3	3
T-LBL	adult	UPN107	DNM2 (p.Thr175fs)	5	0	60	linear	5	0	4	2	1
T-LBL	adult	UPN107	PTEN (p.Asp107Tyr)	5	0	60	linear	5	0	4	2	1
T-LBL	adult	UPN107	BCL11B (p.His743_Glu746del)	5	0	60	linear	5	0	4	2	1
T-LBL	adult	UPN107	SUZ12 (p.Arg129Gly)	6	0	44	linear	6	0	5	2	1
T-LBL	adult	UPN107	PTEN (p.Pro246Leu)	6	0	44	linear	6	0	5	2	0.5
T-LBL	adult	UPN107	PTEN (p.Arg233delinsGlyGlyPro)	7	0	12	linear	7	0	6	2	0
T-LBL	adult	UPN108	dup9p	1	0	17	linear	1	0	0	3	3
T-LBL	adult	UPN108	JAK3 (p.Gln507Pro)	2	0	6	linear	2	0	1	2	1
T-LBL	adult	UPN109	LOH in 9p	1	0	99	linear	1	0	0	2	2
T-LBL	adult	UPN109	NRAS (p.Gln61Lys)	1	0	99	linear	1	0	0	2	1
T-LBL	adult	UPN109	FBXW7 (p.Arg465Cys)	1	0	99	linear	1	0	0	2	1
T-LBL	adult	UPN109	NOTCH3 (p.Ala1607Thr)	2	0	34	linear	2	0	1	2	1
T-LBL	adult	UPN109	NOTCH3 (p.Ser1536Ile)	3	0	21	linear	3	0	2	2	0.5
T-LBL	adult	UPN109	IKZF1 (p.Asn317Lys)	4	0	5	linear	4	0	3	2	1
T-LBL	adult	UPN110	LOH in 9p	1	0	88	linear	1	0	0	2	2
T-LBL	adult	UPN110	NOTCH1 (p.Ile1364Leu)	2	0	53	linear	2	0	1	2	0

1 time point

T-LBL	adult	UPN103												
T-LBL	adult	UPN103												
T-LBL	adult	UPN103												
T-LBL	adult	UPN103												
T-LBL	adult	UPN103												
T-LBL	adult	UPN103												
T-LBL	adult	UPN103												
T-LBL	adult	UPN103												
T-LBL	adult	UPN107	4	153244092	G	A	1287	2	1292	0.15 %	765	1136	1902	59.73 %
T-LBL	adult	UPN107												
T-LBL	adult	UPN107	X	133527949	C	T	1023	1	1029	0.10 %	185	146	332	43.98 %
T-LBL	adult	UPN107												
T-LBL	adult	UPN107	17	30302654	T	C	801	2	807	0.25 %	621	389	1011	38.48 %
T-LBL	adult	UPN107												
T-LBL	adult	UPN107												
T-LBL	adult	UPN107												
T-LBL	adult	UPN107												
T-LBL	adult	UPN107												
T-LBL	adult	UPN107	19	10886515	C	CAGTGAAAA	1395	0	1396	0.00 %	890	262	891	29.41 %
T-LBL	adult	UPN107	10	89692835	G	T	1044	1	1046	0.10 %	624	241	866	27.83 %
T-LBL	adult	UPN107	14	99640934	TCTCGGACGAGTG	T	988	0	990	0.00 %	320	120	441	27.21 %
T-LBL	adult	UPN107	17	30267504	A	G	710	0	713	0.00 %	202	64	266	24.06 %
T-LBL	adult	UPN107	10	89717712	C	T	1103	0	1106	0.00 %	468	118	586	20.14 %
T-LBL	adult	UPN107	10	89717671	ACGA	AGGGGGCCCC	1009	4	1011	0.40 %	534	35	569	6.15 %
T-LBL	adult	UPN108												
T-LBL	adult	UPN108	19	17949121	T	G	1474	2	1481	0.14 %	1186	35	1222	2.86 %
T-LBL	adult	UPN109												
T-LBL	adult	UPN109	1	115256530	G	T	1361	2	1364	0.15 %	568	574	1150	49.91 %
T-LBL	adult	UPN109	4	153249385	G	A	1553	2	1560	0.13 %	675	652	1333	48.91 %
T-LBL	adult	UPN109	19	15281554	C	T	1528	0	1529	0.00 %	1042	219	1264	17.33 %
T-LBL	adult	UPN109	19	15285008	C	A	2036	4	2043	0.20 %	1605	173	1783	9.70 %
T-LBL	adult	UPN109	7	50467716	C	A	1625	2	1630	0.12 %	1672	42	1718	2.44 %
T-LBL	adult	UPN110												
T-LBL	adult	UPN110	9	139400258	T	G	1608	3	1615	0.19 %	1294	467	1765	26.46 %

T-LBL	adult	UPN103	5	0	180915260	2	not detected	49.66 %	49.33 %	49.99 %	6503	CNV
T-LBL	adult	UPN103	8	0	146364022	2	not detected	49.63 %	49.29 %	49.98 %	5813	CNV
T-LBL	adult	UPN103	10	0	135534747	2	not detected	49.71 %	49.35 %	50.07 %	5621	CNV
T-LBL	adult	UPN103	12	0	133851895	2	not detected	49.77 %	49.41 %	50.12 %	5573	CNV
T-LBL	adult	UPN103	15	0	102531392	2	not detected	49.08 %	48.61 %	49.55 %	3431	CNV
T-LBL	adult	UPN103	X	0	155270560	2	not detected	48.61 %	48.37 %	48.85 %	6798	CNV
T-LBL	adult	UPN103	4	109047058	191154276	3	not detected	29.12 %	28.46 %	29.78 %	3126	CNV
T-LBL	adult	UPN103	9	0	38561623	3	not detected	18.53 %	17.66 %	19.40 %	1874	CNV
T-LBL	adult	UPN107										SNV/Indel
T-LBL	adult	UPN107	7	134580334	159138663	1	not detected	81.38 %	80.58 %	82.18 %	1185	CNV
T-LBL	adult	UPN107										SNV/Indel
T-LBL	adult	UPN107	1	5037962	8245849	1	not detected	73.81 %	71.05 %	76.58 %	144	CNV
T-LBL	adult	UPN107										SNV/Indel
T-LBL	adult	UPN107	1	93009438	97544427	1	not detected	70.16 %	67.42 %	72.89 %	161	CNV
T-LBL	adult	UPN107	7	32524272	51555695	1	not detected	69.52 %	68.12 %	70.91 %	678	CNV
T-LBL	adult	UPN107	13	48248195	90539818	1	not detected	62.71 %	61.58 %	63.84 %	1354	CNV
T-LBL	adult	UPN107	8	0	143486863	3	not detected	62.00 %	60.00 %	64.00 %	5199	CNV
T-LBL	adult	UPN107	4	0	191154276	3	not detected	61.00 %	59.00 %	64.00 %	4713	CNV
T-LBL	adult	UPN107										SNV/Indel
T-LBL	adult	UPN107										SNV/Indel
T-LBL	adult	UPN107										SNV/Indel
T-LBL	adult	UPN107										SNV/Indel
T-LBL	adult	UPN107										SNV/Indel
T-LBL	adult	UPN107										SNV/Indel
T-LBL	adult	UPN108	9	0	38919501	3	not detected	17.19 %	16.40 %	17.98 %	1840	CNV
T-LBL	adult	UPN108										SNV/Indel
T-LBL	adult	UPN109	9	0	36743439	2	not detected	99.19 %	98.88 %	99.50 %	1811	CNV
T-LBL	adult	UPN109										SNV/Indel
T-LBL	adult	UPN109										SNV/Indel
T-LBL	adult	UPN109										SNV/Indel
T-LBL	adult	UPN109										SNV/Indel
T-LBL	adult	UPN109										SNV/Indel
T-LBL	adult	UPN110	9	0	27047050	2	not detected	88.23 %	87.48 %	88.99 %	1483	CNV
T-LBL	adult	UPN110										SNV/Indel

1 time point

T-LBL	adult	UPN110	LOH in 9q	3	0	28	linear	3	0	2	2	2
T-LBL	adult	UPN110	PIK3R1 (p.Lys567Glu)	3	0	28	linear	3	0	2	2	1
T-LBL	adult	UPN110	NOTCH1 (p.Leu2350fs)	4	0	16	linear	4	0	3	2	1
T-LBL	adult	UPN110	FBXW7 (p.Arg465Cys)	4	0	16	linear	4	0	3	2	1
T-LBL	adult	UPN110	NOTCH1 (p.Ala2265_Glu2267delinsGluThrGlyGln)	5	0	10	linear	5	0	4	2	1
T-LBL	adult	UPN114	del in 14q	1	0	97	linear	1	0	0	1	1
T-LBL	adult	UPN114	NOTCH1 (p.PheLysArg1606CysTrpPro)	2	0	75	linear	2	0	1	2	1
T-LBL	adult	UPN114	MYB (p.Ser8Cys)	2	0	75	linear	2	0	1	2	1
T-LBL	adult	UPN114	PTEN (p.Ser229fs)	2	0	75	linear	2	0	1	2	1
T-LBL	adult	UPN114	dup in 14q	3	0	68	linear	3	0	2	3	3
T-LBL	adult	UPN114	PTEN (p.Phe154Leu)	4	0	34	linear	4	0	3	2	0
T-LBL	adult	UPN115	dup in 9p	1	0	91	linear	1	0	0	3	3
T-LBL	adult	UPN115	dup20	2	0	69	linear	2	0	1	3	3
T-LBL	adult	UPN115	dup14q	3	0	38	linear	3	0	2	3	3
T-LBL	adult	UPN115	del in 4q	4	0	29	linear	4	0	3	1	1
T-LBL	adult	UPN115	PIK3CA (p.Val344Gly)	5	0	17	linear	5	0	4	2	1
T-LBL	adult	UPN116	LOH9p	1	0	99	linear	1	0	0	2	2
T-LBL	adult	UPN116	TP53 (p.Glu286Gly)	1	0	99	linear	1	0	0	2	1
T-LBL	adult	UPN116	del in 4q	2	0	93	linear	2	0	1	1	1
T-LBL	adult	UPN116	del in 11p, del in 11q	2	0	93	linear	2	0	1	1	1
T-LBL	adult	UPN116	del in 14q	2	0	93	linear	2	0	1	1	1
T-LBL	adult	UPN116	PHF6 (p.Ala51fs)	2	0	93	linear	2	0	1	1	0
T-LBL	adult	UPN116	del in 14q	3	0	88	linear	3	0	2	1	1
T-LBL	adult	UPN116	dup in 14q	3	0	88	linear	3	0	2	3	3
T-LBL	adult	UPN116	dup in 14q	3	0	88	linear	3	0	2	3	3
T-LBL	adult	UPN116	dup in 14q	3	0	88	linear	3	0	2	3	3
T-LBL	adult	UPN116	dup in 14q	3	0	88	linear	3	0	2	3	3
T-LBL	adult	UPN116	dup in 14q	3	0	88	linear	3	0	2	3	3
T-LBL	adult	UPN116	dup in 14q	3	0	88	linear	3	0	2	3	3
T-LBL	adult	UPN116	dup in 14q	3	0	88	linear	3	0	2	3	3
T-LBL	adult	UPN116	del in 14q	3	0	88	linear	3	0	2	1	1

1 time point

Supplemental Data 3

T-LBL	adult	UPN110												
T-LBL	adult	UPN110	5	67591106	A	G	1075	3	1079	0.28 %	816	135	953	14.17 %
T-LBL	adult	UPN110	9	139391137	TGTGCAGC	T	1677	0	1679	0.00 %	1621	159	1783	8.92 %
T-LBL	adult	UPN110	4	153249385	G	A	1249	1	1256	0.08 %	1258	104	1365	7.62 %
T-LBL	adult	UPN110	9	139391392	CAAAGG	GGCCCGTCT	1584	10	1592	0.63 %	1656	85	1737	4.89 %
T-LBL	adult	UPN114												
T-LBL	adult	UPN114	9	139399320	CGCTTCA	GGCCAAC	0	0	1520	0.00 %	994	605	1551	39.01 %
T-LBL	adult	UPN114	6	135502673	A	T	108	0	108	0.00 %	105	64	170	37.65 %
T-LBL	adult	UPN114	10	89717660	TCAGGACCCACACGAC	GCT	1141	4	1144	0.35 %	430	246	679	36.23 %
T-LBL	adult	UPN114												
T-LBL	adult	UPN114	10	89692978	C	A	912	2	915	0.22 %	416	87	503	17.30 %
T-LBL	adult	UPN115												
T-LBL	adult	UPN115												
T-LBL	adult	UPN115												
T-LBL	adult	UPN115												
T-LBL	adult	UPN115	3	178921549	T	G	1191	0	1193	0.00 %	984	92	1080	8.52 %
T-LBL	adult	UPN116												
T-LBL	adult	UPN116	17	7577081	T	C	1785	1	1794	0.06 %	697	789	1489	52.99 %
T-LBL	adult	UPN116												
T-LBL	adult	UPN116												
T-LBL	adult	UPN116												
T-LBL	adult	UPN116	X	133512045	CT	C	268	0	269	0.00 %	18	238	259	91.89 %
T-LBL	adult	UPN116												
T-LBL	adult	UPN116												
T-LBL	adult	UPN116												
T-LBL	adult	UPN116												
T-LBL	adult	UPN116												
T-LBL	adult	UPN116												
T-LBL	adult	UPN116												
T-LBL	adult	UPN116												
T-LBL	adult	UPN116												

1 time point

T-LBL	adult	UPN110	9	131859245	141213430	2	not detected	29.80 %	28.34 %	31.27 %	578	CNV
T-LBL	adult	UPN110										SNV/Indel
T-LBL	adult	UPN110										SNV/Indel
T-LBL	adult	UPN110										SNV/Indel
T-LBL	adult	UPN110										SNV/Indel
T-LBL	adult	UPN114	14	55651083	106878749	1	not detected	96.91 %	96.68 %	97.13 %	2171	CNV
T-LBL	adult	UPN114										SNV/Indel
T-LBL	adult	UPN114										SNV/Indel
T-LBL	adult	UPN114										SNV/Indel
T-LBL	adult	UPN114	14	20666516	42300823	3	not detected	67.68 %	64.82 %	70.54 %	955	CNV
T-LBL	adult	UPN114										SNV/Indel
T-LBL	adult	UPN115	9	133828	21920346	3	not detected	90.82 %	88.29 %	93.34 %	1175	CNV
T-LBL	adult	UPN115	20	0	63025520	3	not detected	69.30 %	67.00 %	71.00 %	2610	CNV
T-LBL	adult	UPN115	14	84860806	107349540	3	not detected	37.76 %	35.96 %	39.56 %	989	CNV
T-LBL	adult	UPN115	4	174701814	190472164	1	not detected	29.00 %	26.00 %	31.00 %	908	CNV
T-LBL	adult	UPN115										SNV/Indel
T-LBL	adult	UPN116	9	0	39680014	2	not detected	97.36 %	97.07 %	97.65 %	2219	CNV
T-LBL	adult	UPN116										SNV/Indel
T-LBL	adult	UPN116	3	114054683	198022430	1	not detected	93.69 %	93.52 %	93.86 %	4080	CNV
T-LBL	adult	UPN116	11	50328682	51581487	1	not detected	95.19 %	90.14 %	100.24 %	9	CNV
T-LBL	adult	UPN116	14	19047940	32333343	1	not detected	93.09 %	92.56 %	93.62 %	707	CNV
T-LBL	adult	UPN116										SNV/Indel
T-LBL	adult	UPN116	14	36674265	37111071	1	not detected	86.39 %	73.55 %	99.24 %	21	CNV
T-LBL	adult	UPN116	14	45128328	46256518	3	not detected	70.96 %	53.70 %	88.22 %	22	CNV
T-LBL	adult	UPN116	14	52838514	53744882	3	not detected	88.59 %	74.61 %	102.57 %	27	CNV
T-LBL	adult	UPN116	14	54337673	56549900	3	not detected	83.92 %	77.79 %	90.05 %	96	CNV
T-LBL	adult	UPN116	14	63540278	65680776	3	not detected	84.13 %	78.08 %	90.18 %	82	CNV
T-LBL	adult	UPN116	14	69314938	70220809	3	not detected	85.37 %	63.91 %	106.83 %	21	CNV
T-LBL	adult	UPN116	14	70988762	72023148	3	not detected	77.06 %	62.71 %	91.42 %	37	CNV
T-LBL	adult	UPN116	14	77217104	78693910	3	not detected	81.12 %	75.73 %	86.52 %	95	CNV
T-LBL	adult	UPN116	14	91577876	92630670	1	not detected	93.63 %	90.10 %	97.15 %	56	CNV

T-LBL	adult	UPN116	dup in 14q	3	0	88	linear	3	0	2	3	3
T-LBL	adult	UPN116	del in 14q	3	0	88	linear	3	0	2	1	1
T-LBL	adult	UPN116	NOTCH1 (p.Ile1718Thr)	4	0	61	linear	4	0	3	2	1
T-LBL	adult	UPN116	NOTCH1 (p.Val2443fs)	5	0	52	linear	5	0	4	2	0.5
T-LBL	adult	UPN116	dup6	6	0	26	linear	6	0	5	3	3
T-LBL	adult	UPN116	NOTCH1 (p.Thr2497fs)	6	0	26	linear	6	0	5	2	0.125
T-LBL	adult	UPN116	NOTCH1 (p.Ala1610_His1611insGlnValLeu)	6	0	26	linear	6	0	5	2	0.125
T-LBL	adult	UPN117	LOH in 9p	1	0	93	linear	1	0	0	2	2
T-LBL	adult	UPN117	PHF6 (p.Ser156*)	1	0	93	linear	1	0	0	2	1
T-LBL	adult	UPN117	NOTCH1 (p.Leu1585Pro)	1	0	93	linear	1	0	0	2	1
T-LBL	adult	UPN117	dup11	2	0	88	linear	2	0	1	3	3
T-LBL	adult	UPN117	STAT5B (p.Asn642His)	2	0	88	linear	2	0	1	2	1
T-LBL	adult	UPN117	dup19	3	0	80	linear	3	0	2	3	3
T-LBL	adult	UPN117	JAK1 (p.Val1045_Phe1046insProLeu)	4	0	73	linear	4	0	3	2	1
T-LBL	adult	UPN118	del in 12p	1	0	93	linear	1	0	0	1	1
T-LBL	adult	UPN118	PHF6 (p.Arg116*)	1	0	93	linear	1	0	0	1	0
T-LBL	adult	UPN118	dup in 9p	2	0	83	linear	2	0	1	3	3
T-LBL	adult	UPN118	dup in 9p	2	0	83	linear	2	0	1	3	3
T-LBL	adult	UPN118	LOH in 18q	2	0	83	linear	2	0	1	2	2
T-LBL	adult	UPN118	dup9q	2	0	83	linear	2	0	1	3	3
T-LBL	adult	UPN118	BCL11B (p.Asp122fs)	2	0	83	linear	2	0	1	2	0.5
T-LBL	adult	UPN118	BCL11B (p.Thr450Met)	2	0	83	linear	2	0	1	2	0.5
T-LBL	adult	UPN118	NOTCH1 (p.Leu1593Pro)	2	0	83	linear	2	0	1	2	1
T-LBL	adult	UPN118	dup in 18q	3	0	32	linear	3	0	2	3	3
T-LBL	adult	UPN118	NOTCH1 (p.Glu2515fs)	3	0	32	linear	3	0	2	2	0.5
T-LBL	adult	UPN118	EZH2 (p.Asp184Asn)	4	0	20	linear	4	0	3	2	1
T-LBL	adult	UPN118	RUNX1 (p.Leu472Pro)	5	0	14	linear	5	0	4	2	1
T-LBL	adult	UPN118	MYB (p.Gln424His)	5	0	14	linear	5	0	4	2	1
T-LBL	adult	UPN118	NOS3 (p.Pro592Ala)	5	0	14	linear	5	0	4	2	1

T-LBL	adult	UPN116												
T-LBL	adult	UPN116												
T-LBL	adult	UPN116	9	139397648	A	G	1592	2	1598	0.13 %	1126	498	1626	30.63 %
T-LBL	adult	UPN116	9	139390864	C	CGG	1826	0	1828	0.00 %	1540	406	1566	25.93 %
T-LBL	adult	UPN116												
T-LBL	adult	UPN116	9	139390703	G	GTTACAAA	1897	0	1903	0.00 %	1758	250	1762	14.19 %
T-LBL	adult	UPN116	9	139399313	T	TAGGACCTGC	2060	0	2067	0.00 %	1848	235	1877	12.52 %
T-LBL	adult	UPN117												
T-LBL	adult	UPN117	X	133547566	C	A	1334	0	1337	0.00 %	679	585	1265	46.25 %
T-LBL	adult	UPN117	9	139399389	A	G	1947	0	1952	0.00 %	870	744	1618	45.98 %
T-LBL	adult	UPN117												
T-LBL	adult	UPN117	17	40359729	T	G	1163	0	1166	0.00 %	560	454	1014	44.77 %
T-LBL	adult	UPN117												
T-LBL	adult	UPN117	1	65303620	C	CAAAGGG	1047	0	1049	0.00 %	895	330	896	36.83 %
T-LBL	adult	UPN118												
T-LBL	adult	UPN118	X	133527636	C	T	426	0	428	0.00 %	8	108	116	93.10 %
T-LBL	adult	UPN118												
T-LBL	adult	UPN118												
T-LBL	adult	UPN118												
T-LBL	adult	UPN118												
T-LBL	adult	UPN118	14	99723871	C	CG	1179	0	1182	0.00 %	1084	449	1088	41.27 %
T-LBL	adult	UPN118	14	99641824	G	A	1057	0	1057	0.00 %	940	661	1607	41.13 %
T-LBL	adult	UPN118	9	139399365	A	G	1624	0	1629	0.00 %	2422	1153	3587	32.14 %
T-LBL	adult	UPN118												
T-LBL	adult	UPN118	9	139390642	GGGACT	GGACCCGGACTCGAGAGGATGAGATCTAAAACC	1369	7	1371	0.51 %	1744	218	2008	10.86 %
T-LBL	adult	UPN118	7	148525907	C	T	992	2	998	0.20 %	528	59	588	10.03 %
T-LBL	adult	UPN118	21	36164460	A	G	783	0	785	0.00 %	878	81	962	8.42 %
T-LBL	adult	UPN118	6	135518167	A	T	1584	0	1585	0.00 %	846	73	920	7.93 %
T-LBL	adult	UPN118	7	150700420	C	G	1177	3	1181	0.25 %	1855	145	2005	7.23 %

T-LBL	adult	UPN116	14	95669965	96147568	3	not detected	80.65 %	70.77 %	90.53 %	44	CNV
T-LBL	adult	UPN116	14	99259751	99499206	1	not detected	91.17 %	84.57 %	97.77 %	28	CNV
T-LBL	adult	UPN116										SNV/Indel
T-LBL	adult	UPN116										SNV/Indel
T-LBL	adult	UPN116	6	0	171115067	3	not detected	24.10 %	23.60 %	24.70 %	9598	CNV
T-LBL	adult	UPN116										SNV/Indel
T-LBL	adult	UPN116										SNV/Indel
T-LBL	adult	UPN117	9	0	36711221	2	not detected	94.86 %	94.23 %	95.48 %	1863	CNV
T-LBL	adult	UPN117										SNV/Indel
T-LBL	adult	UPN117										SNV/Indel
T-LBL	adult	UPN117	11	0	135006516	3	not detected	86.00 %	85.00 %	87.00 %	5390	CNV
T-LBL	adult	UPN117										SNV/Indel
T-LBL	adult	UPN117	19	0	59128983	3	not detected	81.00 %	80.00 %	82.00 %	2475	CNV
T-LBL	adult	UPN117										SNV/Indel
T-LBL	adult	UPN118	12	11634179	13115894	1	not detected	88.89 %	85.40 %	92.38 %	104	CNV
T-LBL	adult	UPN118										SNV/Indel
T-LBL	adult	UPN118	9	0	9953234	3	not detected	82.94 %	80.04 %	85.85 %	599	CNV
T-LBL	adult	UPN118	9	22890505	38705698	3	not detected	78.02 %	74.89 %	81.16 %	622	CNV
T-LBL	adult	UPN118	18	36147473	78077248	2	not detected	81.18 %	80.79 %	81.58 %	1730	CNV
T-LBL	adult	UPN118	9	70731742	141068637	3	not detected	82.00 %	80.00 %	83.00 %	2784	CNV
T-LBL	adult	UPN118										SNV/Indel
T-LBL	adult	UPN118										SNV/Indel
T-LBL	adult	UPN118										SNV/Indel
T-LBL	adult	UPN118	18	19519313	32848214	3	not detected	31.89 %	30.07 %	33.72 %	485	CNV
T-LBL	adult	UPN118										SNV/Indel
T-LBL	adult	UPN118										SNV/Indel
T-LBL	adult	UPN118										SNV/Indel
T-LBL	adult	UPN118										SNV/Indel
T-LBL	adult	UPN118										SNV/Indel

T-LBL	adult	UPN118	KMT2C (p.Met1?)	5	0	14	linear	5	0	4	2	1
T-LBL	adult	UPN118	MED12 (p.Cys1203Ser)	6	0	6	linear	6	0	5	1	0
T-LBL	adult	UPN118	SETD1B (p.Ser1053Trp)	6	0	6	linear	6	0	5	2	1
T-LBL	adult	UPN118	SMARCA4 (p.Ser660Asn)	6	0	6	linear	6	0	5	2	1
T-LBL	adult	UPN118	USH2A (p.Thr3635Asn)	6	0	6	linear	6	0	5	2	1
T-LBL	adult	UPN118	NRAS (p.LeuAsn171IleThr)	6	0	6	linear	6	0	5	2	1
T-LBL	adult	UPN118	NOS3 (p.Asn605His)	6	0	6	linear	6	0	5	2	0.25
T-LBL	adult	UPN118	MYCBP2 (p.Lys3200Thr)	6	0	6	linear	6	0	5	2	1
T-LBL	adult	UPN118	KMT2C (p.Glu674Lys)	6	0	6	linear	6	0	5	2	0.5
T-LBL	adult	UPN118	USH2A (p.Arg2662Ile)	6	0	6	linear	6	0	5	2	0.5
T-LBL	adult	UPN118	BCL11B (p.Ser795Cys)	6	0	6	linear	6	0	5	2	0.25
T-LBL	adult	UPN118	CREBBP (p.Gly876Ala)	6	0	6	linear	6	0	5	2	1
T-LBL	adult	UPN118	KMT2A (p.Pro519Ser)	6	0	6	linear	6	0	5	2	1
T-LBL	adult	UPN118	NOS3 (p.Ser603Asn)	6	0	6	linear	6	0	5	2	0.25
T-LBL	adult	UPN118	ROR2 (p.Leu746Phe)	6	0	6	linear	6	0	5	2	1
T-LBL	adult	UPN118	USH2A (p.Tyr1941Phe)	7	0	3	linear	7	0	6	2	0.25
T-LBL	adult	UPN118	NOS3 (p.Leu289Val)	7	0	3	linear	7	0	6	2	1
T-LBL	adult	UPN118	KMT2D (p.Val5082Gly)	7	0	3	linear	7	0	6	2	1
T-LBL	adult	UPN118	NOS3 (p.Ter630Glnext*?)	7	0	3	linear	7	0	6	2	0
T-LBL	adult	UPN118	SETD1B (p.Ser238Pro)	7	0	3	linear	7	0	6	2	0.5
T-LBL	adult	UPN118	NOS3 (p.Gly327Asp)	7	0	3	linear	7	0	6	2	1
T-LBL	adult	UPN118	NOTCH1 (p.Ala2293Gly)	7	0	3	linear	7	0	6	2	0.25
T-LBL	adult	UPN119	dup in 9q	1	0	66	linear	1	0	0	3	3
T-LBL	adult	UPN119	dup13q	1	0	66	linear	1	0	0	3	3
T-LBL	adult	UPN119	dup in 19p	1	0	66	linear	1	0	0	3	3
T-LBL	adult	UPN119	dup19q	1	0	66	linear	1	0	0	3	3
T-LBL	adult	UPN119	SETD1B (p.Pro733fs)	2	0	50	linear	2	0	1	2	1
T-LBL	adult	UPN120	dup13q	1	0	100	linear	1	0	0	3	3
T-LBL	adult	UPN120	dup8	2	0	95	linear	2	0	1	3	3
T-LBL	adult	UPN120	LOH in 4q	3	0	89	linear	3	0	2	2	2
T-LBL	adult	UPN120	del in 1p	4	0	83	linear	4	0	3	1	1
T-LBL	adult	UPN120	del in 2q	4	0	83	linear	4	0	3	1	1
T-LBL	adult	UPN120	del in 3p	4	0	83	linear	4	0	3	1	1
T-LBL	adult	UPN120	del in 5q	4	0	83	linear	4	0	3	1	1
T-LBL	adult	UPN120	del7p	4	0	83	linear	4	0	3	1	1

T-LBL	adult	UPN118	7	151836833	A	G	1185	0	1188	0.00 %	648	42	691	6.08 %
T-LBL	adult	UPN118	X	70349196	G	C	615	1	617	0.16 %	527	35	562	6.23 %
T-LBL	adult	UPN118	12	122255456	C	G	1534	0	1536	0.00 %	1433	63	1500	4.20 %
T-LBL	adult	UPN118	19	11114051	G	A	1209	5	1214	0.41 %	479	21	503	4.17 %
T-LBL	adult	UPN118	1	215953220	G	T	1329	1	1336	0.07 %	737	31	769	4.03 %
T-LBL	adult	UPN118	1	115251211	TTGAG	GTGAT	1198	3	1201	0.25 %	530	22	554	3.97 %
T-LBL	adult	UPN118	7	150700299	A	C	998	0	1001	0.00 %	1407	56	1471	3.81 %
T-LBL	adult	UPN118	13	77671690	T	G	1379	2	1385	0.14 %	857	30	887	3.38 %
T-LBL	adult	UPN118	7	151945499	C	T	1239	0	1241	0.00 %	722	25	749	3.34 %
T-LBL	adult	UPN118	1	216062006	C	A	1322	1	1325	0.08 %	800	26	828	3.14 %
T-LBL	adult	UPN118	14	99640790	T	A	898	0	898	0.00 %	1182	37	1222	3.03 %
T-LBL	adult	UPN118	16	3820824	C	G	1752	0	1755	0.00 %	707	22	731	3.01 %
T-LBL	adult	UPN118	11	118343429	C	T	1305	2	1308	0.15 %	696	20	717	2.79 %
T-LBL	adult	UPN118	7	150700294	G	A	999	0	1000	0.00 %	1406	40	1447	2.76 %
T-LBL	adult	UPN118	9	94486540	G	A	1656	0	1662	0.00 %	1784	42	1827	2.30 %
T-LBL	adult	UPN118	1	216246266	T	A	1251	2	1255	0.16 %	887	20	908	2.20 %
T-LBL	adult	UPN118	7	150708877	C	G	837	0	837	0.00 %	1213	27	1243	2.17 %
T-LBL	adult	UPN118	12	49420504	A	C	1479	1	1480	0.07 %	1352	26	1380	1.88 %
T-LBL	adult	UPN118	7	150700374	T	C	1302	0	1303	0.00 %	2304	44	2353	1.87 %
T-LBL	adult	UPN118	12	122247563	T	C	1457	2	1462	0.14 %	1880	21	1902	1.10 %
T-LBL	adult	UPN118	7	150696301	G	A	1393	0	1397	0.00 %	1912	21	1938	1.08 %
T-LBL	adult	UPN118	9	139391313	G	C	1598	0	1599	0.00 %	3053	33	3092	1.07 %
T-LBL	adult	UPN119												
T-LBL	adult	UPN119												
T-LBL	adult	UPN119												
T-LBL	adult	UPN119												
T-LBL	adult	UPN119	12	122252318	C	CTA	945	0	947	0.00 %	648	164	651	25.19 %
T-LBL	adult	UPN120												
T-LBL	adult	UPN120												
T-LBL	adult	UPN120												
T-LBL	adult	UPN120												
T-LBL	adult	UPN120												
T-LBL	adult	UPN120												
T-LBL	adult	UPN120												
T-LBL	adult	UPN120												

Supplemental Data 3

T-LBL	adult	UPN118										SNV/Indel
T-LBL	adult	UPN118										SNV/Indel
T-LBL	adult	UPN118										SNV/Indel
T-LBL	adult	UPN118										SNV/Indel
T-LBL	adult	UPN118										SNV/Indel
T-LBL	adult	UPN118										SNV/Indel
T-LBL	adult	UPN118										SNV/Indel
T-LBL	adult	UPN118										SNV/Indel
T-LBL	adult	UPN118										SNV/Indel
T-LBL	adult	UPN118										SNV/Indel
T-LBL	adult	UPN118										SNV/Indel
T-LBL	adult	UPN118										SNV/Indel
T-LBL	adult	UPN118										SNV/Indel
T-LBL	adult	UPN118										SNV/Indel
T-LBL	adult	UPN118										SNV/Indel
T-LBL	adult	UPN118										SNV/Indel
T-LBL	adult	UPN118										SNV/Indel
T-LBL	adult	UPN118										SNV/Indel
T-LBL	adult	UPN118										SNV/Indel
T-LBL	adult	UPN118										SNV/Indel
T-LBL	adult	UPN118										SNV/Indel
T-LBL	adult	UPN118										SNV/Indel
T-LBL	adult	UPN119	9	127255540	141213431	3	not detected	65.09 %	62.86 %	67.32 %	953	CNV
T-LBL	adult	UPN119	13	19020095	115169878	3	not detected	67.49 %	66.65 %	68.34 %	6408	CNV
T-LBL	adult	UPN119	19	16255842	24572955	3	not detected	67.82 %	63.37 %	72.28 %	346	CNV
T-LBL	adult	UPN119	19	27738372	59128983	3	not detected	66.38 %	64.79 %	67.96 %	2259	CNV
T-LBL	adult	UPN119										SNV/Indel
T-LBL	adult	UPN120	13	19020095	115169878	3	not detected	110.75 %	109.86 %	111.64 %	3847	CNV
T-LBL	adult	UPN120	8	0	146292681	3	not detected	95.64 %	94.96 %	96.32 %	5848	CNV
T-LBL	adult	UPN120	4	92378037	191154276	2	not detected	89.05 %	88.87 %	89.23 %	3369	CNV
T-LBL	adult	UPN120	1	740281	26667650	1	not detected	81.84 %	81.15 %	82.53 %	1238	CNV
T-LBL	adult	UPN120	2	167810088	185267449	1	not detected	83.40 %	82.88 %	83.93 %	695	CNV
T-LBL	adult	UPN120	3	0	19582271	1	not detected	83.97 %	83.63 %	84.31 %	1196	CNV
T-LBL	adult	UPN120	5	116807579	180915260	1	not detected	83.98 %	83.71 %	84.25 %	2544	CNV
T-LBL	adult	UPN120	7	0	58050276	1	not detected	83.98 %	83.72 %	84.24 %	2707	CNV

1 time point

T-LBL	adult	UPN120	del in 9q	4	0	83	linear	4	0	3	1	1
T-LBL	adult	UPN120	del in 10p	4	0	83	linear	4	0	3	1	1
T-LBL	adult	UPN120	del in 15q	4	0	83	linear	4	0	3	1	1
T-LBL	adult	UPN120	del in 17p	4	0	83	linear	4	0	3	1	1
T-LBL	adult	UPN120	del in 21q	4	0	83	linear	4	0	3	1	1
T-LBL	adult	UPN120	dup2p, dup in 2q	5	0	66	linear	5	0	4	3	3
T-LBL	adult	UPN120	dup in 2q	5	0	66	linear	5	0	4	3	3
T-LBL	adult	UPN120	dup in 5p	5	0	66	linear	5	0	4	3	3
T-LBL	adult	UPN120	dup9p	5	0	66	linear	5	0	4	3	3
T-LBL	adult	UPN120	dup in 12p	5	0	66	linear	5	0	4	3	3
T-LBL	adult	UPN120	dup in 12p, dup12q	5	0	66	linear	5	0	4	3	3
T-LBL	adult	UPN120	dup in 9q	6	0	58	linear	6	0	5	3	3
T-LBL	adult	UPN120	dup in 9q	6	0	58	linear	6	0	5	3	3
T-LBL	adult	UPN120	dup in 4p	7	0	41	linear	7	0	6	3	3
T-LBL	adult	UPN120	dup in 4q	7	0	41	linear	7	0	6	3	3
T-LBL	adult	UPN120	del in 5p	7	0	41	linear	7	0	6	1	1
T-LBL	adult	UPN120	del in 5q	7	0	41	linear	7	0	6	1	1
T-LBL	adult	UPN120	LOH6	7	0	41	linear	7	0	6	2	2
T-LBL	adult	UPN120	del in 15q	7	0	41	linear	7	0	6	1	1
T-LBL	adult	UPN120	del in 16q	7	0	41	linear	7	0	6	1	1
T-LBL	adult	UPN120	del in 17p+q	7	0	41	linear	7	0	6	1	1
T-LBL	adult	UPN120	LOH11	8	0	37	linear	8	0	7	2	2
T-LBL	adult	UPN120	dup in 5p	9	0	31	linear	9	0	8	3	3
T-LBL	adult	UPN120	dup18	9	0	31	linear	9	0	8	3	3
T-LBL	adult	UPN120	dup20	9	0	31	linear	9	0	8	3	3
T-LBL	adult	UPN121	dup in 3p	1	0	91	linear	1	0	0	3	3
T-LBL	adult	UPN121	dup in 9p	2	0	16	linear	2	0	1	3	3
T-LBL	pediatric	UPN125	LOH in 5q	1	0	85	linear	1	0	0	2	2
T-LBL	pediatric	UPN125	LOH in 9p	1	0	85	linear	1	0	0	2	2
T-LBL	pediatric	UPN125	NOTCH1 (p.Leu1574Pro)	1	0	85	linear	1	0	0	2	1
T-LBL	pediatric	UPN125	NOTCH1 (p.Gln2501*)	2	0	79	linear	2	0	1	2	0.5
T-LBL	pediatric	UPN125	del in 1p	3	0	73	linear	3	0	2	1	1
T-LBL	pediatric	UPN125	del in 1p	3	0	73	linear	3	0	2	1	1
T-LBL	pediatric	UPN125	del in 1p	3	0	73	linear	3	0	2	1	1
T-LBL	pediatric	UPN125	del in 5q	3	0	73	linear	3	0	2	1	1

1 time point

T-LBL	adult	UPN120												
T-LBL	adult	UPN120												
T-LBL	adult	UPN120												
T-LBL	adult	UPN120												
T-LBL	adult	UPN120												
T-LBL	adult	UPN120												
T-LBL	adult	UPN120												
T-LBL	adult	UPN120												
T-LBL	adult	UPN120												
T-LBL	adult	UPN120												
T-LBL	adult	UPN120												
T-LBL	adult	UPN120												
T-LBL	adult	UPN120												
T-LBL	adult	UPN120												
T-LBL	adult	UPN120												
T-LBL	adult	UPN120												
T-LBL	adult	UPN120												
T-LBL	adult	UPN120												
T-LBL	adult	UPN120												
T-LBL	adult	UPN120												
T-LBL	adult	UPN120												
T-LBL	adult	UPN120												
T-LBL	adult	UPN120												
T-LBL	adult	UPN120												
T-LBL	adult	UPN120												
T-LBL	adult	UPN121												
T-LBL	adult	UPN121												
T-LBL	pediatric	UPN125												
T-LBL	pediatric	UPN125												
T-LBL	pediatric	UPN125	9	139399422	A	G	1974	1	1985	0.05 %	1520	1126	2650	42.49 %
T-LBL	pediatric	UPN125	9	139390690	G	A	1871	1	1876	0.05 %	1398	912	2315	39.40 %
T-LBL	pediatric	UPN125												
T-LBL	pediatric	UPN125												
T-LBL	pediatric	UPN125												
T-LBL	pediatric	UPN125												

T-LBL	adult	UPN120	9	95604241	133046525	1	not detected	83.39 %	83.05 %	83.72 %	1431	CNV
T-LBL	adult	UPN120	10	2868377	20478672	1	not detected	83.38 %	82.98 %	83.77 %	1029	CNV
T-LBL	adult	UPN120	15	22320346	43869365	1	not detected	83.36 %	82.90 %	83.81 %	775	CNV
T-LBL	adult	UPN120	17	0	18555537	1	not detected	82.79 %	82.32 %	83.27 %	985	CNV
T-LBL	adult	UPN120	21	19458097	48129895	1	not detected	83.90 %	83.54 %	84.26 %	1288	CNV
T-LBL	adult	UPN120	2	0	167810087	3	not detected	68.76 %	68.16 %	69.36 %	5889	CNV
T-LBL	adult	UPN120	2	185267450	243199373	3	not detected	68.50 %	67.52 %	69.47 %	2216	CNV
T-LBL	adult	UPN120	5	18102468	32539413	3	not detected	64.23 %	61.91 %	66.54 %	428	CNV
T-LBL	adult	UPN120	9	0	38783261	3	not detected	65.53 %	64.17 %	66.89 %	1908	CNV
T-LBL	adult	UPN120	12	0	7788420	3	not detected	66.36 %	64.20 %	68.52 %	484	CNV
T-LBL	adult	UPN120	12	10917102	133851895	3	not detected	68.17 %	67.50 %	68.83 %	4793	CNV
T-LBL	adult	UPN120	9	70959750	95604240	3	not detected	57.30 %	55.88 %	58.71 %	933	CNV
T-LBL	adult	UPN120	9	133046526	141213430	3	not detected	59.47 %	57.15 %	61.79 %	476	CNV
T-LBL	adult	UPN120	4	0	50400000	3	not detected	40.39 %	39.51 %	41.28 %	1969	CNV
T-LBL	adult	UPN120	4	52683607	93205330	3	not detected	42.72 %	41.63 %	43.80 %	1389	CNV
T-LBL	adult	UPN120	5	6810714	14029187	1	not detected	40.68 %	39.36 %	42.01 %	310	CNV
T-LBL	adult	UPN120	5	49760627	58938399	1	not detected	44.82 %	43.70 %	45.93 %	349	CNV
T-LBL	adult	UPN120	6	0	171115067	2	not detected	40.40 %	40.17 %	40.64 %	8500	CNV
T-LBL	adult	UPN120	15	43869366	102014745	1	not detected	40.04 %	39.60 %	40.48 %	2568	CNV
T-LBL	adult	UPN120	16	55484047	57395486	1	not detected	41.85 %	39.77 %	43.92 %	107	CNV
T-LBL	adult	UPN120	17	29128222	31206144	1	not detected	42.95 %	35.33 %	50.58 %	74	CNV
T-LBL	adult	UPN120	11	0	135006516	2	not detected	37.09 %	36.77 %	37.41 %	5323	CNV
T-LBL	adult	UPN120	5	32539414	46303072	3	not detected	33.13 %	31.21 %	35.05 %	428	CNV
T-LBL	adult	UPN120	18	0	77894226	3	not detected	31.02 %	30.39 %	31.65 %	3148	CNV
T-LBL	adult	UPN120	20	0	63025520	3	not detected	28.58 %	27.95 %	29.21 %	2781	CNV
T-LBL	adult	UPN121	3	59703278	61018717	3	not detected	91.03 %	87.00 %	95.06 %	133	CNV
T-LBL	adult	UPN121	9	0	30665434	3	not detected	16.77 %	15.94 %	17.60 %	1602	CNV
T-LBL	pediatric	UPN125	5	137871981	170720430	2	not detected	85.27 %	84.89 %	85.65 %	1192	CNV
T-LBL	pediatric	UPN125	9	0	36639060	2	not detected	86.61 %	86.29 %	86.93 %	1750	CNV
T-LBL	pediatric	UPN125										SNV/Indel
T-LBL	pediatric	UPN125										SNV/Indel
T-LBL	pediatric	UPN125	1	2152776	8150169	1	not detected	72.85 %	71.89 %	73.82 %	316	CNV
T-LBL	pediatric	UPN125	1	9931222	16021144	1	not detected	72.29 %	71.27 %	73.30 %	274	CNV
T-LBL	pediatric	UPN125	1	17221730	24901264	1	not detected	71.88 %	70.98 %	72.79 %	364	CNV
T-LBL	pediatric	UPN125	5	170720430	180915260	1	not detected	74.21 %	73.55 %	74.87 %	606	CNV

T-LBL	pediatric	UPN125	DNM2 (p.Lys562del)	3	0	73	linear	3	0	2	2	1
T-LBL	pediatric	UPN125	dup in 14q	4	0	62	linear	4	0	3	3	3
T-LBL	pediatric	UPN125	CREBBP (p.Ala1398Asp)	4	0	62	linear	4	0	3	2	1
T-LBL	pediatric	UPN125	CCND3 (p.Ser254fs)	5	0	54	linear	5	0	4	2	1
T-LBL	pediatric	UPN125	CREBBP (p.Gln816*)	6	0	5	linear	6	0	5	2	1
T-LBL	pediatric	UPN127	NOTCH1 (p.Leu1678Pro)	1	0	100	linear	1	0	0	2	1
T-LBL	pediatric	UPN127	PHF6 (p.Cys327fs)	2	0	92	linear	2	0	1	1	0
T-LBL	pediatric	UPN127	BCL11B (p.Ser358*)	2	0	92	linear	2	0	1	2	1
T-LBL	pediatric	UPN127	FBXW7 (p.Arg465Cys)	3	0	78	linear	3	0	2	2	1
T-LBL	pediatric	UPN127	dup21q	4	0	52	linear	4	0	3	3	3
T-LBL	pediatric	UPN127	NOTCH3 (p.Ile161Leu)	5	0	37	linear	5	0	4	2	1
T-LBL	pediatric	UPN127	KMT2C (p.Trp1056*)	6	0	22	linear	6	0	5	2	1
T-LBL	pediatric	UPN129	LOH9p	1	0	96	branching (3)	1	0	0	2	2
T-LBL	pediatric	UPN129	USP7 (p.Glu973Gly)	2	0	84	branching (3)	2	0	1	2	1
T-LBL	pediatric	UPN129	del in 6q	3	0	33	branching (3)	3	0	2	1	1
T-LBL	pediatric	UPN129	NOTCH1 (p.Leu2457fs)	3	0	33	branching (3)	3	0	2	2	1
T-LBL	pediatric	UPN129	del in 6q	4	0	23	branching (3)	4	0	3	1	1
T-LBL	pediatric	UPN129	NOTCH1 (p.Arg1598Pro)	5	0	6	branching (3)	5	2	4	2	0.5
T-LBL	pediatric	UPN129	NOTCH1 (p.Leu1585Pro)	6	0	6	branching (3)	5	2	4	2	0.5
T-LBL	pediatric	UPN129	FBXW7 (p.Arg505Cys)	7	0	5	branching (3)	5	2	4	2	1
T-LBL	pediatric	UPN130	del in 12p	1	0	69	linear	1	0	0	1	1
T-LBL	pediatric	UPN130	USP7 (p.Cys300_Arg301insCysSer)	1	0	69	linear	1	0	0	2	1
T-LBL	pediatric	UPN130	NOTCH1 (p.Leu1574Pro)	1	0	69	linear	1	0	0	2	1
T-LBL	pediatric	UPN130	KMT2D (p.Gln1522*)	1	0	69	linear	1	0	0	2	1
T-LBL	pediatric	UPN130	FBXW7 (p.Arg465His)	2	0	32	linear	2	0	1	2	0
T-LBL	pediatric	UPN130	FBXW7 (p.Arg465Cys)	2	0	32	linear	2	0	1	2	0
T-LBL	pediatric	UPN130	CCND3 (p.Met202Val)	3	0	25	linear	3	0	2	2	0
T-LBL	pediatric	UPN130	NOTCH1 (p.Phe1592delinsLeuGly)	3	0	25	linear	3	0	2	2	0
T-LBL	pediatric	UPN130	CCND3 (p.Met1?)	3	0	25	linear	3	0	2	2	0
T-LBL	pediatric	UPN130	dup in 9p	4	0	16	linear	4	0	3	3	3
T-LBL	pediatric	UPN131	LOH9p	1	0	59	linear	1	0	0	2	2

1 time point

T-LBL	pediatric	UPN125	19	10930662	GAGA	G	1422	0	1425	0.00 %	1108	604	1715	35.22 %
T-LBL	pediatric	UPN125												
T-LBL	pediatric	UPN125	16	3789666	G	T	1443	0	1450	0.00 %	1212	526	1741	30.21 %
T-LBL	pediatric	UPN125	6	41903798	C	CTTTAAGGGGGT	1766	0	1769	0.00 %	2180	588	2184	26.92 %
T-LBL	pediatric	UPN125	16	3823769	G	A	1164	0	1169	0.00 %	1293	35	1333	2.63 %
T-LBL	pediatric	UPN127	9	139397768	A	G	2506	4	2519	0.16 %	611	699	1312	53.28 %
T-LBL	pediatric	UPN127	X	133559238	TG	T	598	0	600	0.00 %	4	48	52	92.31 %
T-LBL	pediatric	UPN127	14	99642100	G	T	1702	2	1707	0.12 %	1521	1310	2834	46.22 %
T-LBL	pediatric	UPN127	4	153249385	G	A	1755	0	1760	0.00 %	196	127	325	39.08 %
T-LBL	pediatric	UPN127												
T-LBL	pediatric	UPN127	19	15273221	T	G	0	0	1602	0.00 %	0	68	366	18.58 %
T-LBL	pediatric	UPN127	7	151921255	C	T	1397	78	1478	5.28 %	375	46	421	10.93 %
T-LBL	pediatric	UPN129												
T-LBL	pediatric	UPN129	16	8989500	T	C	1003	1	1005	0.10 %	794	583	1380	42.25 %
T-LBL	pediatric	UPN129												
T-LBL	pediatric	UPN129	9	139390823	A	AATTTGTGAGGT	1582	0	1583	0.00 %	2126	383	2154	17.78 %
T-LBL	pediatric	UPN129												
T-LBL	pediatric	UPN129	9	139399350	C	G	1674	0	1682	0.00 %	2168	71	2241	3.17 %
T-LBL	pediatric	UPN129	9	139399389	A	G	1555	0	1555	0.00 %	2255	70	2329	3.01 %
T-LBL	pediatric	UPN129	4	153247289	G	A	1404	0	1408	0.00 %	1998	48	2048	2.34 %
T-LBL	pediatric	UPN130												
T-LBL	pediatric	UPN130	16	9010364	G	GGCTACA	1002	8	1015	0.79 %	896	327	905	36.13 %
T-LBL	pediatric	UPN130	9	139399422	A	G	1958	21	1983	1.06 %	1491	811	2309	35.12 %
T-LBL	pediatric	UPN130	12	49440062	G	A	1817	29	1848	1.57 %	1234	601	1840	32.66 %
T-LBL	pediatric	UPN130	4	153249384	C	T	1518	4	1526	0.26 %	1281	265	1548	17.12 %
T-LBL	pediatric	UPN130	4	153249385	G	A	1498	4	1512	0.26 %	1289	243	1534	15.84 %
T-LBL	pediatric	UPN130	6	41904404	T	C	1476	1	1482	0.07 %	1454	234	1691	13.84 %
T-LBL	pediatric	UPN130	9	139399367	G	GCCC	1851	0	1851	0.00 %	2274	284	2279	12.46 %
T-LBL	pediatric	UPN130	6	41904418	A	T	1389	4	1395	0.29 %	1413	192	1610	11.93 %
T-LBL	pediatric	UPN130												
T-LBL	pediatric	UPN131												

T-LBL	pediatric	UPN125										SNV/Indel
T-LBL	pediatric	UPN125	14	99355266	106053648	3	not detected	64.68 %	61.58 %	67.77 %	331	CNV
T-LBL	pediatric	UPN125										SNV/Indel
T-LBL	pediatric	UPN125										SNV/Indel
T-LBL	pediatric	UPN125										SNV/Indel
T-LBL	pediatric	UPN127										SNV/Indel
T-LBL	pediatric	UPN127										SNV/Indel
T-LBL	pediatric	UPN127										SNV/Indel
T-LBL	pediatric	UPN127										SNV/Indel
T-LBL	pediatric	UPN127	21	14359894	48099610	3	not detected	52.11 %	49.47 %	54.75 %	1586	CNV
T-LBL	pediatric	UPN127										SNV/Indel
T-LBL	pediatric	UPN127										SNV/Indel
T-LBL	pediatric	UPN129	9	0	40420994	2	not detected	96.17 %	95.85 %	96.50 %	1778	CNV
T-LBL	pediatric	UPN129										SNV/Indel
T-LBL	pediatric	UPN129	6	75681568	96872082	1	not detected	33.03 %	31.88 %	34.17 %	677	CNV
T-LBL	pediatric	UPN129										SNV/Indel
T-LBL	pediatric	UPN129	6	96872083	121487840	1	not detected	23.60 %	22.69 %	24.51 %	867	CNV
T-LBL	pediatric	UPN129										SNV/Indel
T-LBL	pediatric	UPN129										SNV/Indel
T-LBL	pediatric	UPN129										SNV/Indel
T-LBL	pediatric	UPN130	12	12410513	13009119	1	not detected	71.56 %	68.22 %	74.90 %	54	CNV
T-LBL	pediatric	UPN130										SNV/Indel
T-LBL	pediatric	UPN130										SNV/Indel
T-LBL	pediatric	UPN130										SNV/Indel
T-LBL	pediatric	UPN130										SNV/Indel
T-LBL	pediatric	UPN130										SNV/Indel
T-LBL	pediatric	UPN130										SNV/Indel
T-LBL	pediatric	UPN130										SNV/Indel
T-LBL	pediatric	UPN130										SNV/Indel
T-LBL	pediatric	UPN130	9	0	38899071	3	not detected	18.23 %	17.34 %	19.11 %	1922	CNV
T-LBL	pediatric	UPN131	9	0	38919501	2	10.00 %	58.78 %	57.89 %	59.67 %	1773	CNV

T-LBL	pediatric	UPN131	NOTCH1 (p.Phe1606_Lys1607insGlySerLeu)	2	0	41	linear	2	0	1	2	1
T-LBL	pediatric	UPN132	del in 13q	1	0	98	linear	1	0	0	1	1
T-LBL	pediatric	UPN132	NOTCH1 (p.Ser2513fs)	2	0	88	linear	2	0	1	2	1
T-LBL	pediatric	UPN132	NOTCH1 (p.Phe1592delinsLeuGluArgProLysLeu)	3	0	67	linear	3	0	2	2	0
T-LBL	pediatric	UPN132	dup in 9p	4	0	16	linear	4	0	3	3	3
T-LBL	pediatric	UPN133	LOH in 3q	1	0	100	linear	1	0	0	2	2
T-LBL	pediatric	UPN133	LOH in 4p	1	0	100	linear	1	0	0	2	2
T-LBL	pediatric	UPN133	LOH in 7p	1	0	100	linear	1	0	0	2	2
T-LBL	pediatric	UPN133	LOH in 8p	1	0	100	linear	1	0	0	2	2
T-LBL	pediatric	UPN133	LOH in 8q	1	0	100	linear	1	0	0	2	2
T-LBL	pediatric	UPN133	LOH in 9p	1	0	100	linear	1	0	0	2	2
T-LBL	pediatric	UPN133	LOH in 9q	1	0	100	linear	1	0	0	2	2
T-LBL	pediatric	UPN133	LOH in 10q	1	0	100	linear	1	0	0	2	2
T-LBL	pediatric	UPN133	LOH in 10q	1	0	100	linear	1	0	0	2	2
T-LBL	pediatric	UPN133	LOH in 11p	1	0	100	linear	1	0	0	2	2
T-LBL	pediatric	UPN133	LOH in 11q	1	0	100	linear	1	0	0	2	2
T-LBL	pediatric	UPN133	LOH in 13q	1	0	100	linear	1	0	0	2	2
T-LBL	pediatric	UPN133	LOH in 13q	1	0	100	linear	1	0	0	2	2
T-LBL	pediatric	UPN133	LOH in 14q	1	0	100	linear	1	0	0	2	2
T-LBL	pediatric	UPN133	LOH in 15q	1	0	100	linear	1	0	0	2	2
T-LBL	pediatric	UPN133	LOH in 16p	1	0	100	linear	1	0	0	2	2
T-LBL	pediatric	UPN133	LOH in 4q	1	0	100	linear	1	0	0	2	2
T-LBL	pediatric	UPN133	LOH in 17p	1	0	100	linear	1	0	0	2	2
T-LBL	pediatric	UPN133	del in 9p	2	0	94	linear	2	0	1	1	1
T-LBL	pediatric	UPN133	PIK3CA (p.Met1005Lys)	2	0	94	linear	2	0	1	2	1
T-LBL	pediatric	UPN133	BCL11B (p.Glu430*)	2	0	94	linear	2	0	1	2	1
T-LBL	pediatric	UPN133	NOTCH1 (p.Phe1592Ser)	3	0	88	linear	3	0	2	2	1
T-LBL	pediatric	UPN133	MED12 (p.Ala38_Leu39insArgValAla)	4	0	18	linear	4	0	3	1	0
T-LBL	pediatric	UPN133	PIK3CD (p.Asn299Lys)	4	0	18	linear	5	0	4	2	1
T-LBL	pediatric	UPN133	CNOT3 (p.Gly493fs)	4	0	18	linear	5	0	4	2	1

T-LBL	pediatric	UPN131	9	139399324	T	TGAGGGATCC	1812	0	1819	0.00 %	1565	326	1567	20.80 %
T-LBL	pediatric	UPN132												
T-LBL	pediatric	UPN132	9	139390655	C	CG	1331	0	1335	0.00 %	2029	899	2038	44.11 %
T-LBL	pediatric	UPN132	9	139399367	G	GAGTTTCGGGCGCTCC	1621	3	1622	0.18 %	2185	733	2189	33.49 %
T-LBL	pediatric	UPN132												
T-LBL	pediatric	UPN133												
T-LBL	pediatric	UPN133												
T-LBL	pediatric	UPN133												
T-LBL	pediatric	UPN133												
T-LBL	pediatric	UPN133												
T-LBL	pediatric	UPN133												
T-LBL	pediatric	UPN133												
T-LBL	pediatric	UPN133												
T-LBL	pediatric	UPN133												
T-LBL	pediatric	UPN133												
T-LBL	pediatric	UPN133												
T-LBL	pediatric	UPN133												
T-LBL	pediatric	UPN133												
T-LBL	pediatric	UPN133												
T-LBL	pediatric	UPN133												
T-LBL	pediatric	UPN133												
T-LBL	pediatric	UPN133												
T-LBL	pediatric	UPN133												
T-LBL	pediatric	UPN133												
T-LBL	pediatric	UPN133	3	178951959	T	A	1351	13	1367	0.95 %	673	599	1273	47.05 %
T-LBL	pediatric	UPN133	14	99641885	C	A	1593	11	1609	0.68 %	695	618	1314	47.03 %
T-LBL	pediatric	UPN133	9	139399368	A	G	2139	21	2163	0.97 %	933	743	1679	44.25 %
T-LBL	pediatric	UPN133	X	70339233	C	CGGCCAGGGT	794	0	798	0.00 %	548	90	550	16.36 %
T-LBL	pediatric	UPN133	1	9777666	C	A	1705	3	1715	0.17 %	1252	152	1407	10.80 %
T-LBL	pediatric	UPN133	19	54653360	CAG	C	2058	0	2063	0.00 %	1365	119	1488	8.00 %

Supplemental Data 3

T-LBL	pediatric	UPN131											SNV/Indel
T-LBL	pediatric	UPN132	13	67445133	67805530	1	not detected		98.62 %	97.68 %	99.56 %	15	CNV
T-LBL	pediatric	UPN132											SNV/Indel
T-LBL	pediatric	UPN132											SNV/Indel
T-LBL	pediatric	UPN132	9	0	38899071	3	not detected		17.27 %	16.46 %	18.09 %	1864	CNV
T-LBL	pediatric	UPN133	3	107818841	128247400	2	not detected		97.34 %	96.24 %	98.44 %	596	CNV
T-LBL	pediatric	UPN133	4	14474484	38262811	2	not detected		99.33 %	99.21 %	99.46 %	795	CNV
T-LBL	pediatric	UPN133	7	39242146	55179265	2	not detected		99.41 %	99.34 %	99.49 %	661	CNV
T-LBL	pediatric	UPN133	8	24866974	43791691	2	not detected		99.34 %	99.26 %	99.42 %	493	CNV
T-LBL	pediatric	UPN133	8	46925940	77765631	2	not detected		99.39 %	99.32 %	99.45 %	901	CNV
T-LBL	pediatric	UPN133	9	32407080	45755225	2	not detected		95.14 %	92.78 %	97.49 %	259	CNV
T-LBL	pediatric	UPN133	9	70731742	80218340	2	not detected		98.96 %	98.38 %	99.54 %	370	CNV
T-LBL	pediatric	UPN133	10	73857766	84399810	2	not detected		98.90 %	98.16 %	99.65 %	273	CNV
T-LBL	pediatric	UPN133	10	114130142	125892909	2	not detected		99.44 %	99.31 %	99.56 %	503	CNV
T-LBL	pediatric	UPN133	11	36283728	51581487	2	not detected		99.21 %	98.89 %	99.54 %	443	CNV
T-LBL	pediatric	UPN133	11	54847856	87858441	2	not detected		99.26 %	99.07 %	99.45 %	1242	CNV
T-LBL	pediatric	UPN133	13	41126167	61854131	2	not detected		99.27 %	98.93 %	99.61 %	732	CNV
T-LBL	pediatric	UPN133	13	105773160	115169878	2	not detected		99.58 %	99.51 %	99.65 %	563	CNV
T-LBL	pediatric	UPN133	14	95481396	107349540	2	not detected		99.54 %	99.47 %	99.61 %	480	CNV
T-LBL	pediatric	UPN133	15	79987432	93877687	2	not detected		99.21 %	98.86 %	99.56 %	714	CNV
T-LBL	pediatric	UPN133	16	12785099	22962166	2	not detected		99.47 %	99.38 %	99.56 %	377	CNV
T-LBL	pediatric	UPN133	4	129569621	138596043	2	not detected		99.42 %	99.30 %	99.54 %	237	CNV
T-LBL	pediatric	UPN133	17	0	8597112	2	not detected		98.89 %	98.22 %	99.56 %	450	CNV
T-LBL	pediatric	UPN133	9	21296347	23296786	1	not detected		85.43 %	80.25 %	90.61 %	94.53 %	CNV
T-LBL	pediatric	UPN133											SNV/Indel
T-LBL	pediatric	UPN133											SNV/Indel
T-LBL	pediatric	UPN133											SNV/Indel
T-LBL	pediatric	UPN133											SNV/Indel
T-LBL	pediatric	UPN133											SNV/Indel
T-LBL	pediatric	UPN133											SNV/Indel

1 time point

T-LBL	pediatric	UPN134	del in 11p	1	0	98	linear	1	0	0	1	1
T-LBL	pediatric	UPN134	del in 10q	2	0	93	linear	2	0	1	1	1
T-LBL	pediatric	UPN134	PTEN (p.Arg233fs)	2	0	93	linear	2	0	1	1	0
T-LBL	pediatric	UPN134	del in 8q	3	0	65	linear	3	0	2	1	1
T-LBL	pediatric	UPN134	CCND3 (p.Glu253fs)	3	0	65	linear	3	0	2	2	1
T-LBL	pediatric	UPN134	KMT2C (p.Arg4268fs)	4	0	33	linear	4	0	3	2	1
T-LBL	pediatric	UPN134	dup in 9p	5	0	16	linear	5	0	4	3	3
T-LBL	pediatric	UPN134	SMARCA4 (p.Arg526His)	6	0	9	linear	6	0	5	2	1
T-LBL	pediatric	UPN135	del in 9p	1	0	78	linear	1	0	0	1	1
T-LBL	pediatric	UPN135	BCL11B (p.Ser465Leu)	1	0	78	linear	1	0	0	2	1
T-LBL	pediatric	UPN135	PHF6 (p.Glu151fs)	2	0	49	linear	2	0	1	1	0
T-LBL	pediatric	UPN135	USP7 (p.Tyr265_Glu266insAlaLeuAlaLeuGlnArgValPheTyr)	3	0	22	linear	3	0	2	2	1
T-LBL	pediatric	UPN136	del in 5p	1	0	97	linear	1	0	0	1	1
T-LBL	pediatric	UPN136	del in 5q	1	0	97	linear	1	0	0	1	1
T-LBL	pediatric	UPN136	del in 5q	1	0	97	linear	1	0	0	1	1
T-LBL	pediatric	UPN136	del in 5q	1	0	97	linear	1	0	0	1	1
T-LBL	pediatric	UPN136	del in 5q	1	0	97	linear	1	0	0	1	1
T-LBL	pediatric	UPN136	del in 14q	1	0	97	linear	1	0	0	1	1
T-LBL	pediatric	UPN136	del in 14q	1	0	97	linear	1	0	0	1	1
T-LBL	pediatric	UPN136	del in 14q	1	0	97	linear	1	0	0	1	1
T-LBL	pediatric	UPN136	del in 14q	1	0	97	linear	1	0	0	1	1
T-LBL	pediatric	UPN136	del in 10q	1	0	97	linear	1	0	0	1	1
T-LBL	pediatric	UPN136	NOTCH1 (p.Ile1680Asn)	1	0	97	linear	1	0	0	2	1
T-LBL	pediatric	UPN136	KDM6A (p.Arg1118dup)	2	0	89	linear	2	0	1	1	0
T-LBL	pediatric	UPN136	dup in 9p	3	0	17	linear	3	0	2	3	3
T-LBL	pediatric	UPN137	IL7R (p.Leu248_Ser249insGlyTrp)	1	0	80	linear	1	0	0	2	1
T-LBL	pediatric	UPN137	NOTCH1 (p.Lys1607delinsProGln)	1	0	80	linear	1	0	0	2	1
T-LBL	pediatric	UPN138	LOH in 9p	1	0	95	linear	1	0	0	2	2

1 time point

T-LBL	pediatric	UPN134												
T-LBL	pediatric	UPN134												
T-LBL	pediatric	UPN134	10	89717668	C	CACCG	941	68	942	7.22 %	568	491	569	86.29 %
T-LBL	pediatric	UPN134												
T-LBL	pediatric	UPN134	6	41903800	C	CCCTACTTTTT	1552	127	1554	8.17 %	1537	497	1541	32.25 %
T-LBL	pediatric	UPN134	7	151848562	G	GGGACCCAGTTCACTTTCCCTCTGAAGTATTCA	969	29	972	2.98 %	982	164	986	16.63 %
T-LBL	pediatric	UPN134												
T-LBL	pediatric	UPN134	19	11105661	G	A	1178	2	1182	0.17 %	1534	75	1609	4.66 %
T-LBL	pediatric	UPN135												
T-LBL	pediatric	UPN135	14	99641779	G	A	753	12	769	1.56 %	896	563	1462	38.51 %
T-LBL	pediatric	UPN135	X	133547550	G	GGAACTCCTGGGGGATGA	471	1	472	0.21 %	269	136	277	49.10 %
T-LBL	pediatric	UPN135	16	9010937	T	TCATAGAACACTCTTTGTAATGCTAAAG	929	3	933	0.32 %	1075	122	1096	11.13 %
T-LBL	pediatric	UPN136												
T-LBL	pediatric	UPN136												
T-LBL	pediatric	UPN136												
T-LBL	pediatric	UPN136												
T-LBL	pediatric	UPN136												
T-LBL	pediatric	UPN136												
T-LBL	pediatric	UPN136												
T-LBL	pediatric	UPN136												
T-LBL	pediatric	UPN136												
T-LBL	pediatric	UPN136												
T-LBL	pediatric	UPN136	9	139397762	A	T	1602	65	1669	3.89 %	1073	1010	2090	48.33 %
T-LBL	pediatric	UPN136	X	44942749	T	TGAG	607	63	608	10.36 %	526	477	532	89.66 %
T-LBL	pediatric	UPN136												
T-LBL	pediatric	UPN137	5	35874587	T	TGGGGTG	1360	0	1363	0.00 %	723	287	730	39.32 %
T-LBL	pediatric	UPN137	9	139399324	T	GGGG	1726	0	1729	0.00 %	1461	1008	2500	40.32 %
T-LBL	pediatric	UPN138												

Supplemental Data 3

T-LBL	pediatric	UPN134	11	33958586	36618126	1	not detected	98.28 %	98.01 %	98.54 %	181	CNV
T-LBL	pediatric	UPN134	10	89627829	89855585	1	not detected	91.03 %	77.10 %	104.96 %	14	CNV
T-LBL	pediatric	UPN134										SNV/Indel
T-LBL	pediatric	UPN134	8	128754500	130204854	1	not detected	68.62 %	62.24 %	74.99 %	43	CNV
T-LBL	pediatric	UPN134										SNV/Indel
T-LBL	pediatric	UPN134										SNV/Indel
T-LBL	pediatric	UPN134	9	0	38899071	3	not detected	17.89 %	17.03 %	18.75 %	1915	CNV
T-LBL	pediatric	UPN134										SNV/Indel
T-LBL	pediatric	UPN135	9	0	28189113	1	not detected	78.16 %	77.73 %	78.59 %	1582	CNV
T-LBL	pediatric	UPN135										SNV/Indel
T-LBL	pediatric	UPN135										SNV/Indel
T-LBL	pediatric	UPN135										SNV/Indel
T-LBL	pediatric	UPN136	5	35405204	35855954	1	not detected	98.29 %	96.84 %	99.73 %	15	CNV
T-LBL	pediatric	UPN136	5	66561416	70308242	1	not detected	96.80 %	94.05 %	99.56 %	69	CNV
T-LBL	pediatric	UPN136	5	132265889	134674291	1	not detected	98.66 %	98.22 %	99.10 %	104	CNV
T-LBL	pediatric	UPN136	5	169510228	172609525	1	not detected	99.08 %	98.88 %	99.28 %	172	CNV
T-LBL	pediatric	UPN136	5	176717144	178495212	1	not detected	95.54 %	92.16 %	98.92 %	76	CNV
T-LBL	pediatric	UPN136	14	64305463	69938694	1	not detected	98.43 %	98.13 %	98.73 %	206	CNV
T-LBL	pediatric	UPN136	14	75146103	77570750	1	not detected	98.72 %	98.28 %	99.16 %	116	CNV
T-LBL	pediatric	UPN136	14	99029731	99609320	1	not detected	99.14 %	98.67 %	99.60 %	42	CNV
T-LBL	pediatric	UPN136	14	103002718	103421653	1	not detected	NA	NA	NA	3	CNV
T-LBL	pediatric	UPN136	10	89624787	89943622	1	not detected	89.40 %	77.85 %	100.95 %	20	CNV
T-LBL	pediatric	UPN136										SNV/Indel
T-LBL	pediatric	UPN136										SNV/Indel
T-LBL	pediatric	UPN136	9	0	38899071	3	not detected	18.10 %	17.24 %	18.96 %	1826	CNV
T-LBL	pediatric	UPN137										SNV/Indel
T-LBL	pediatric	UPN137										SNV/Indel
T-LBL	pediatric	UPN138	9	0	37428097	2	not detected	88.11 %	87.09 %	89.14 %	1800	CNV

1 time point

T-LBL	pediatric	UPN138	PHF6 (p.Ile315Thr)	1	0	95	linear	1	0	0	1	0
T-LBL	pediatric	UPN138	BCL11B (p.Lys425fs)	2	0	71	linear	2	0	1	2	1
T-LBL	pediatric	UPN138	NOTCH1 (p.Ser2449fs)	2	0	71	linear	2	0	1	2	1
T-LBL	pediatric	UPN139	FBXW7 (p.Arg465His)	1	0	100	linear	1	0	0	2	1
T-LBL	pediatric	UPN139	PHF6 (p.His303fs)	2	0	92	linear	2	0	1	1	0
T-LBL	pediatric	UPN139	NOTCH1 (p.Leu1600Pro)	2	0	92	linear	2	0	1	2	1
T-LBL	pediatric	UPN139	dup18	3	0	86	linear	3	0	2	3	3
T-LBL	pediatric	UPN139	FBXW7 (p.Lys189fs)	3	0	86	linear	3	0	2	2	0.5
T-LBL	pediatric	UPN139	KMT2D (p.Asp5040Asn)	4	0	41	linear	4	0	3	2	1
T-LBL	pediatric	UPN139	KMT2D (p.Arg1757*)	5	0	32	linear	5	0	4	2	0.5
T-LBL	pediatric	UPN139	dup in 9p	6	0	16	linear	6	0	5	3	3
T-LBL	pediatric	UPN142	del in 6q	1	0	80	branching (2)	1	0	0	1	1
T-LBL	pediatric	UPN142	dup6	1	0	80	branching (2)	1	0	0	3	3
T-LBL	pediatric	UPN142	NOTCH1 (p.Val2453fs)	1	0	80	branching (2)	1	0	0	2	1
T-LBL	pediatric	UPN142	dup20	2	0	72	branching (2)	2	0	1	3	3
T-LBL	pediatric	UPN142	BCL11B (p.Glu531fs)	2	0	72	branching (2)	2	0	1	2	1
T-LBL	pediatric	UPN142	DDX3X (p.Ser28fs)	3	0	56	branching (2)	3	0	2	1	0
T-LBL	pediatric	UPN142	PTEN (p.Glu235fs)	4	0	38	branching (2)	4	1	3	2	1
T-LBL	pediatric	UPN142	PTEN (p.Arg233fs)	5	0	30	branching (2)	5	0	4	2	0
T-LBL	pediatric	UPN142	FBXW7 (p.Arg465His)	6	0	11	branching (2)	6	0	5	2	1
T-LBL	pediatric	UPN142	SMARCA4 (p.Lys835Asn)	7	0	11	branching (2)	7	0	6	2	1
T-LBL	pediatric	UPN142	DDX3X (p.Ser654fs)	8	0	17	branching (2)	4	1	3	1	0
T-LBL	pediatric	UPN142	PTEN (p.Asn228fs)	8	0	17	branching (2)	4	1	3	2	1
T-LBL	pediatric	UPN143	FBXW7 (p.Arg465Cys)	1	0	100	linear	1	0	0	2	1
T-LBL	pediatric	UPN143	LOH in 4q	2	0	57	linear	2	0	1	2	2
T-LBL	pediatric	UPN143	NOTCH1 (p.Gln2444*)	3	0	48	linear	3	0	2	2	1
T-LBL	pediatric	UPN143	dup in 17q	4	0	40	linear	4	0	3	3	3
T-LBL	pediatric	UPN143	NOTCH1 (p.Arg1598Pro)	5	0	34	linear	5	0	4	2	0.5
T-LBL	pediatric	UPN143	dup in 9p	6	0	17	linear	6	0	5	3	3
T-LBL	pediatric	UPN143	NOTCH1 (p.Leu1678Gln)	7	0	6	linear	7	0	6	2	0.25
T-LBL	pediatric	UPN144	del in 9q	1	0	82	linear	1	0	0	1	1
T-LBL	pediatric	UPN144	NOTCH1 (p.Asp2442fs)	2	0	71	linear	2	0	1	2	0.5

1 time point

T-LBL	pediatric	UPN138	X	133551305	T	C	527	0	527	0.00 %	3	130	134	97.01 %
T-LBL	pediatric	UPN138	14	99641900	T	TG	1315	0	1319	0.00 %	1605	585	1614	36.25 %
T-LBL	pediatric	UPN138	9	139390809	CTCTCCTGGGGCAGAATAGTGTGCACCGCCAGGCTGC	CCCGGA	1485	7	1487	0.47 %	734	262	735	35.65 %
T-LBL	pediatric	UPN139	4	153249384	C	T	1560	1	1567	0.06 %	737	818	1559	52.47 %
T-LBL	pediatric	UPN139	X	133551266	AC	A	661	0	662	0.00 %	38	576	629	91.57 %
T-LBL	pediatric	UPN139	9	139399344	A	G	1921	0	1925	0.00 %	1027	914	1947	46.94 %
T-LBL	pediatric	UPN139												
T-LBL	pediatric	UPN139	4	153271213	T	TG	1033	0	1033	0.00 %	952	422	961	43.91 %
T-LBL	pediatric	UPN139	12	49420631	C	T	1762	0	1766	0.00 %	1482	390	1873	20.82 %
T-LBL	pediatric	UPN139	12	49437701	G	A	1701	0	1705	0.00 %	1539	293	1836	15.96 %
T-LBL	pediatric	UPN139												
T-LBL	pediatric	UPN142												
T-LBL	pediatric	UPN142												
T-LBL	pediatric	UPN142	9	139390834	C	CCG	1634	0	1635	0.00 %	1094	442	1103	40.07 %
T-LBL	pediatric	UPN142												
T-LBL	pediatric	UPN142	14	99641583	C	CGGGGGTT	1040	0	1041	0.00 %	1017	349	1018	34.28 %
T-LBL	pediatric	UPN142	X	41196694	CAG	C	523	0	526	0.00 %	123	154	277	55.60 %
T-LBL	pediatric	UPN142	10	89717678	GAAGAC	CAGTAGG	1105	7	1106	0.63 %	352	85	440	19.32 %
T-LBL	pediatric	UPN142	10	89717672	C	GA	1131	0	1133	0.00 %	370	73	483	15.11 %
T-LBL	pediatric	UPN142	4	153249384	C	T	1229	4	1239	0.32 %	647	40	689	5.81 %
T-LBL	pediatric	UPN142	19	11129699	G	C	1458	0	1461	0.00 %	684	37	723	5.12 %
T-LBL	pediatric	UPN142	X	41206941	A	AC	572	0	573	0.00 %	297	51	299	17.06 %
T-LBL	pediatric	UPN142	10	89717658	ATTCAGGACCCACACGA	TTTTTTTTGAGGG	1112	4	1114	0.36 %	370	37	452	8.19 %
T-LBL	pediatric	UPN143	4	153249385	G	A	1028	2	1032	0.19 %	722	647	1372	47.16 %
T-LBL	pediatric	UPN143												
T-LBL	pediatric	UPN143	9	139390861	G	A	1574	3	1578	0.19 %	1524	491	2021	24.29 %
T-LBL	pediatric	UPN143												
T-LBL	pediatric	UPN143	9	139399350	C	G	1619	0	1622	0.00 %	1829	378	2210	17.10 %
T-LBL	pediatric	UPN143												
T-LBL	pediatric	UPN143	9	139397768	A	T	1581	1	1590	0.06 %	1905	64	1979	3.23 %
T-LBL	pediatric	UPN144												
T-LBL	pediatric	UPN144	9	139390863	ACG	AATTCGAGGAC	1254	9	1255	0.72 %	1779	671	1784	37.61 %

T-LBL	pediatric	UPN138										SNV/Indel
T-LBL	pediatric	UPN138										SNV/Indel
T-LBL	pediatric	UPN138										SNV/Indel
T-LBL	pediatric	UPN139										SNV/Indel
T-LBL	pediatric	UPN139										SNV/Indel
T-LBL	pediatric	UPN139										SNV/Indel
T-LBL	pediatric	UPN139	18	0	78077248	3	not detected	84.00 %	83.00 %	85.00 %	3154	CNV
T-LBL	pediatric	UPN139										SNV/Indel
T-LBL	pediatric	UPN139										SNV/Indel
T-LBL	pediatric	UPN139										SNV/Indel
T-LBL	pediatric	UPN139	9	0	38899017	3	not detected	17.12 %	16.33 %	17.91 %	1819	CNV
T-LBL	pediatric	UPN142	6	79039661	116003773	1	not detected	83.70 %	82.94 %	84.46 %	1067	CNV
T-LBL	pediatric	UPN142	6	0	78966583	3	not detected	76.83 %	74.60 %	79.05 %	7505	CNV
T-LBL	pediatric	UPN142										SNV/Indel
T-LBL	pediatric	UPN142	20	0	62960292	3	not detected	73.40 %	71.18 %	75.62 %	2784	CNV
T-LBL	pediatric	UPN142										SNV/Indel
T-LBL	pediatric	UPN142										SNV/Indel
T-LBL	pediatric	UPN142										SNV/Indel
T-LBL	pediatric	UPN142										SNV/Indel
T-LBL	pediatric	UPN142										SNV/Indel
T-LBL	pediatric	UPN142										SNV/Indel
T-LBL	pediatric	UPN142										SNV/Indel
T-LBL	pediatric	UPN142										SNV/Indel
T-LBL	pediatric	UPN143										SNV/Indel
T-LBL	pediatric	UPN143	4	151637268	190937862	3	not detected	62.14 %	61.69 %	62.59 %	1885	CNV
T-LBL	pediatric	UPN143										SNV/Indel
T-LBL	pediatric	UPN143	17	31148437	81195210	3	not detected	40.67 %	39.89 %	41.46 %	2070	CNV
T-LBL	pediatric	UPN143										SNV/Indel
T-LBL	pediatric	UPN143	9	0	38899017	3	not detected	16.75 %	15.99 %	17.51 %	1991	CNV
T-LBL	pediatric	UPN143										SNV/Indel
T-LBL	pediatric	UPN144	9	2570295	30426501	1	not detected	82.09 %	80.74 %	83.45 %	1399	CNV
T-LBL	pediatric	UPN144										SNV/Indel

T-LBL	pediatric	UPN144	NOTCH1 (p.Phe1606delinsLeuGlyHis)	2	0	71	linear	2	0	1	2	0.5
T-LBL	pediatric	UPN144	SMARCA4 (p.Arg885His)	3	0	52	linear	3	0	2	2	1
T-LBL	pediatric	UPN144	BCL11B (p.Glu553*)	4	0	7	linear	4	0	3	2	1
T-LBL	pediatric	UPN144	MYB (p.Ala372fs)	4	0	7	linear	4	0	3	2	1
T-LBL	pediatric	UPN145	del in 5q	1	0	97	branching (3)	1	0	0	1	1
T-LBL	pediatric	UPN145	del in 11q	1	0	97	branching (3)	1	0	0	1	1
T-LBL	pediatric	UPN145	del in 13q	1	0	97	branching (3)	1	0	0	1	1
T-LBL	pediatric	UPN145	NOTCH1 (p.Arg1586Pro)	2	0	40	branching (3)	2	2	1	2	1
T-LBL	pediatric	UPN145	dup in 1q	3	0	30	branching (3)	2	2	1	3	3
T-LBL	pediatric	UPN145	NOTCH1 (p.Met1580_Gln1584delinsIle)	3	0	30	branching (3)	2	2	1	2	1
T-LBL	pediatric	UPN145	dup in 9p	4	0	15	branching (3)	2	2	1	3	3
T-LBL	pediatric	UPN145	NOTCH1 (p.Pro1582dup)	4	0	15	branching (3)	2	2	1	2	1
T-LBL	pediatric	UPN147	del9	1	0	97	linear	1	0	0	1	1
T-LBL	pediatric	UPN147	PTPRD (p.Pro1667Ser)	1	0	97	linear	1	0	0	1	0
T-LBL	pediatric	UPN147	NOTCH1 (p.Phe1606_Lys1607insAspLeuIle)	2	0	68	linear	2	0	1	1	0
T-LBL	pediatric	UPN148	del3q	1	0	96	linear	1	0	0	1	1
T-LBL	pediatric	UPN148	del in 4q	1	0	96	linear	1	0	0	1	1
T-LBL	pediatric	UPN148	del in 4q	1	0	96	linear	1	0	0	1	1
T-LBL	pediatric	UPN148	PHF6 (p.Arg129*)	1	0	96	linear	1	0	0	1	0
T-LBL	pediatric	UPN148	KMT2A (p.Thr1061Ala)	1	0	96	linear	1	0	0	2	1
T-LBL	pediatric	UPN148	EZH2 (p.Glu745Lys)	1	0	96	linear	1	0	0	2	1
T-LBL	pediatric	UPN148	SETD1B (p.Asp37Gly)	1	0	96	linear	1	0	0	2	1
T-LBL	pediatric	UPN148	LOH in 9q	2	0	86	linear	2	0	1	2	2
T-LBL	pediatric	UPN148	PIK3CA (p.Val344Met)	2	0	86	linear	2	0	1	1	0
T-LBL	pediatric	UPN148	del in 14q	3	0	78	linear	3	0	2	1	1
T-LBL	pediatric	UPN148	dup in 19q	4	0	28	linear	4	0	3	2	2
T-LBL	pediatric	UPN148	NOTCH1 (p.Ala1701Pro)	5	0	12	linear	5	0	4	2	1
T-LBL	pediatric	UPN148	NOTCH1 (p.Ile2402_Gln2403insPheCysCys)	6	0	5	linear	6	0	5	2	0.5
T-LBL	pediatric	UPN149	del in 9p	1	0	82	branching (3)	1	0	0	1	1

T-LBL	pediatric	UPN144	9	139399325	G	GTGGCCT	1213	1	1213	0.08 %	2373	817	2382	34.30 %
T-LBL	pediatric	UPN144	19	11132438	G	A	1227	0	1229	0.00 %	1573	553	2131	25.95 %
T-LBL	pediatric	UPN144	14	99641516	C	A	988	7	996	0.70 %	1729	85	1816	4.68 %
T-LBL	pediatric	UPN144	6	135517050	AGCAAGGT	TCCCA	1252	7	1258	0.56 %	1773	49	1828	2.68 %
T-LBL	pediatric	UPN145												
T-LBL	pediatric	UPN145												
T-LBL	pediatric	UPN145												
T-LBL	pediatric	UPN145	9	139399386	C	G	1549	0	1551	0.00 %	2020	506	2532	19.98 %
T-LBL	pediatric	UPN145												
T-LBL	pediatric	UPN145	9	139399389	AGCTGCTCCGGCGGC	AGT	1528	6	1531	0.39 %	2049	348	2477	14.05 %
T-LBL	pediatric	UPN145												
T-LBL	pediatric	UPN145	9	139399396	C	CCGG	1576	1	1577	0.06 %	2099	128	2104	6.08 %
T-LBL	pediatric	UPN147												
T-LBL	pediatric	UPN147	9	8341217	G	A	373	332	707	46.96 %	11	173	184	94.02 %
T-LBL	pediatric	UPN147	9	139399324	T	TGATCAGGTC	1241	2	1243	0.16 %	1212	804	1217	66.06 %
T-LBL	pediatric	UPN148												
T-LBL	pediatric	UPN148												
T-LBL	pediatric	UPN148												
T-LBL	pediatric	UPN148	X	133527949	C	T	535	2	537	0.37 %	31	525	556	94.42 %
T-LBL	pediatric	UPN148	11	118347544	A	G	1017	2	1021	0.20 %	660	680	1341	50.71 %
T-LBL	pediatric	UPN148	7	148504761	C	T	1120	0	1121	0.00 %	777	714	1494	47.79 %
T-LBL	pediatric	UPN148	12	122242753	A	G	1088	5	1111	0.45 %	969	851	1829	46.53 %
T-LBL	pediatric	UPN148												
T-LBL	pediatric	UPN148	3	178921548	G	A	1023	0	1030	0.00 %	106	543	653	83.15 %
T-LBL	pediatric	UPN148												
T-LBL	pediatric	UPN148												
T-LBL	pediatric	UPN148	9	139397700	C	G	1212	1	1220	0.08 %	2229	148	2379	6.22 %
T-LBL	pediatric	UPN148	9	139390984	G	GGCAGCAAAA	1435	0	1439	0.00 %	2412	57	2414	2.36 %
T-LBL	pediatric	UPN149												

1 time point

T-LBL	pediatric	UPN144										SNV/Indel
T-LBL	pediatric	UPN144										SNV/Indel
T-LBL	pediatric	UPN144										SNV/Indel
T-LBL	pediatric	UPN144										SNV/Indel
T-LBL	pediatric	UPN145	5	55086783	79297024	1	not detected	96.69 %	96.40 %	96.98 %	855	CNV
T-LBL	pediatric	UPN145	11	118358040	123875515	1	not detected	97.30 %	97.02 %	97.58 %	276	CNV
T-LBL	pediatric	UPN145	13	50603622	51382899	1	not detected	97.30 %	96.53 %	98.06 %	54	CNV
T-LBL	pediatric	UPN145										SNV/Indel
T-LBL	pediatric	UPN145	1	151594736	240695998	3	not detected	32.85 %	32.28 %	33.42 %	3641	CNV
T-LBL	pediatric	UPN145										SNV/Indel
T-LBL	pediatric	UPN145	9	0	38899071	3	not detected	17.63 %	16.82 %	18.45 %	1850	CNV
T-LBL	pediatric	UPN145										SNV/Indel
T-LBL	pediatric	UPN147	9	0	141213430	1	NA	98.00 %				CNV
T-LBL	pediatric	UPN147										SNV/Indel
T-LBL	pediatric	UPN147										SNV/Indel
T-LBL	pediatric	UPN148	3	93513538	198022430	1	not detected	97.43 %	97.32 %	97.54 %	3685	CNV
T-LBL	pediatric	UPN148	4	185363859	185910783	1	not detected	97.88 %	97.41 %	98.35 %	44	CNV
T-LBL	pediatric	UPN148	4	188817997	191154276	1	not detected	97.26 %	96.66 %	97.86 %	129	CNV
T-LBL	pediatric	UPN148										SNV/Indel
T-LBL	pediatric	UPN148										SNV/Indel
T-LBL	pediatric	UPN148										SNV/Indel
T-LBL	pediatric	UPN148										SNV/Indel
T-LBL	pediatric	UPN148	9	130971290	141213431	2	not detected	85.55 %	84.28 %	86.83 %	591	CNV
T-LBL	pediatric	UPN148										SNV/Indel
T-LBL	pediatric	UPN148	14	66528508	100573572	1	not detected	77.97 %	77.58 %	78.37 %	1500	CNV
T-LBL	pediatric	UPN148	19	38666523	59128983	3	not detected	28.87 %	27.69 %	30.05 %	1103	CNV
T-LBL	pediatric	UPN148										SNV/Indel
T-LBL	pediatric	UPN148										SNV/Indel
T-LBL	pediatric	UPN149	9	0	28468067	1	not detected	81.84 %	81.04 %	82.65 %	1561	CNV

Supplemental Data 3

T-LBL	pediatric	UPN149	STAT5B (p.Tyr665Phe)	2	0	71	branching (3)	2	0	1	2	1
T-LBL	pediatric	UPN149	STAT5B (p.Thr628Ser)	3	0	51	branching (3)	3	0	2	2	0.5
T-LBL	pediatric	UPN149	del in 12q	4	0	43	branching (3)	4	0	3	1	1
T-LBL	pediatric	UPN149	NOTCH1 (p.Pro2514fs)	5	0	22	branching (3)	5	2	4	2	1
T-LBL	pediatric	UPN149	NOTCH1 (p.Ser2486*)	6	0	7	branching (3)	5	2	4	2	1
T-LBL	pediatric	UPN149	NOTCH1 (p.Arg1598Pro)	7	0	5	branching (3)	5	2	4	2	1
T-LBL	pediatric	UPN150	del in 9p	1	0	91	linear	1	0	0	1	1
T-LBL	pediatric	UPN150	NOTCH1 (p.Gln2395*)	1	0	91	linear	1	0	0	2	0.5
T-LBL	pediatric	UPN150	NOTCH1 (p.Leu1585Pro)	1	0	91	linear	1	0	0	2	0.5
T-LBL	pediatric	UPN150	BCL11B (p.Tyr854Cys)	1	0	91	linear	1	0	0	2	1
T-LBL	pediatric	UPN150	DNM2 (p.Leu793fs)	2	0	66	linear	2	0	1	2	1
T-LBL	pediatric	UPN151	amp in Xp, ampXq	1	0	100	linear	1	0	0	4	4
T-LBL	pediatric	UPN151	del in Xp	2	0	93	linear	2	0	1	1	1
T-LBL	pediatric	UPN151	NOTCH1 (p.Ser2499fs)	3	0	81	linear	3	0	2	2	1
T-LBL	pediatric	UPN151	PTEN (p.Arg233fs)	4	0	71	linear	4	0	3	2	0.5
T-LBL	pediatric	UPN151	PTEN (p.Met199del)	4	0	71	linear	4	0	3	2	0.5
T-LBL	pediatric	UPN151	del in 9p	5	0	15	linear	5	0	4	1	1
T-LBL	pediatric	UPN151	PTEN (p.Pro246fs)	6	0	10	linear	6	0	5	2	0
T-LBL	pediatric	UPN152	LOH in 9p	1	0	95	linear	1	0	0	2	2
T-LBL	pediatric	UPN152	dup in 9p	1	0	95	linear	1	0	0	3	3
T-LBL	pediatric	UPN152	del in 10q	2	0	58	linear	2	0	1	1	1
T-LBL	pediatric	UPN152	PTEN (p.Phe241fs)	2	0	58	linear	2	0	1	1	0
T-LBL	pediatric	UPN152	PIK3CA (p.Glu542Lys)	3	0	43	linear	3	0	2	2	1
T-LBL	pediatric	UPN152	PIK3R1 (p.Gln579_Tyr580del)	4	0	36	linear	4	0	3	2	1
T-LBL	pediatric	UPN152	NOTCH1 (p.Tyr2490*)	4	0	36	linear	4	0	3	2	0.5
T-LBL	pediatric	UPN152	NOTCH1 (p.Leu1600Pro)	4	0	36	linear	4	0	3	2	0.5
T-LBL	pediatric	UPN153	dup in 9q	1	0	40	linear	1	0	0	3	3
T-LBL	pediatric	UPN153	dup8	2	0	31	linear	2	0	1	3	3
T-LBL	pediatric	UPN153	dup in 11p	2	0	31	linear	2	0	1	3	3
T-LBL	pediatric	UPN153	STAT5B (p.Tyr665Phe)	2	0	31	linear	2	0	1	2	1
T-LBL	pediatric	UPN154	del in 14q	1	0	98	linear	1	0	0	1	1
T-LBL	pediatric	UPN154	del9p	1	0	98	linear	1	0	0	1	1
T-LBL	pediatric	UPN154	dup in 17q	2	0	86	linear	2	0	1	3	3
T-LBL	pediatric	UPN154	LOH in 6p	2	0	86	linear	2	0	1	2	2
T-LBL	pediatric	UPN154	dup9q	3	0	62	linear	3	0	2	3	3

1 time point

T-LBL	pediatric	UPN149	17	40359659	T	A	1192	0	1195	0.00 %	382	210	592	35.47 %
T-LBL	pediatric	UPN149	17	40362212	G	C	996	1	998	0.10 %	313	107	421	25.42 %
T-LBL	pediatric	UPN149												
T-LBL	pediatric	UPN149	9	139390648	CAG	C	1316	0	1320	0.00 %	2252	282	2550	11.06 %
T-LBL	pediatric	UPN149	9	139390734	G	T	1406	0	1406	0.00 %	2949	105	3058	3.43 %
T-LBL	pediatric	UPN149	9	139399350	C	G	1596	2	1601	0.12 %	3313	84	3406	2.47 %
T-LBL	pediatric	UPN150												
T-LBL	pediatric	UPN150	9	139391008	G	A	2363	2	2370	0.08 %	1361	1200	2564	46.80 %
T-LBL	pediatric	UPN150	9	139399389	A	G	2179	1	2180	0.05 %	1178	1026	2208	46.47 %
T-LBL	pediatric	UPN150	14	99640612	T	C	1849	0	1851	0.00 %	1068	855	1928	44.35 %
T-LBL	pediatric	UPN150	19	10940881	G	GCC	1486	0	1488	0.00 %	1642	550	1653	33.27 %
T-LBL	pediatric	UPN151												
T-LBL	pediatric	UPN151												
T-LBL	pediatric	UPN151	9	139390694	G	GCTCAC	1908	4	1914	0.21 %	2506	1021	2522	40.48 %
T-LBL	pediatric	UPN151	10	89717672	C	GA	1406	0	1413	0.00 %	526	307	840	36.55 %
T-LBL	pediatric	UPN151	10	89711972	AGAT	A	1298	4	1306	0.31 %	589	312	906	34.44 %
T-LBL	pediatric	UPN151												
T-LBL	pediatric	UPN151	10	89717712	C	GGGGG	1549	0	1553	0.00 %	923	50	975	5.13 %
T-LBL	pediatric	UPN152												
T-LBL	pediatric	UPN152												
T-LBL	pediatric	UPN152												
T-LBL	pediatric	UPN152	10	89717695	CTT	C	1513	0	1517	0.00 %	87	61	148	41.22 %
T-LBL	pediatric	UPN152	3	178936082	G	A	1463	1	1464	0.07 %	472	129	602	21.43 %
T-LBL	pediatric	UPN152	5	67591139	GACCAAT	G	1225	0	1231	0.00 %	411	92	503	18.29 %
T-LBL	pediatric	UPN152	9	139390721	G	T	2976	1	2982	0.03 %	791	175	970	18.04 %
T-LBL	pediatric	UPN152	9	139399344	A	G	3120	0	3129	0.00 %	848	184	1032	17.83 %
T-LBL	pediatric	UPN153												
T-LBL	pediatric	UPN153												
T-LBL	pediatric	UPN153												
T-LBL	pediatric	UPN153	17	40359659	T	A	1366	1	1370	0.07 %	1303	252	1561	16.14 %
T-LBL	pediatric	UPN154												
T-LBL	pediatric	UPN154												
T-LBL	pediatric	UPN154												
T-LBL	pediatric	UPN154												
T-LBL	pediatric	UPN154												

T-LBL	pediatric	UPN149										SNV/Indel
T-LBL	pediatric	UPN149										SNV/Indel
T-LBL	pediatric	UPN149	12	118615643	130345983	1	not detected	42.86 %	41.75 %	43.97 %	603	CNV
T-LBL	pediatric	UPN149										SNV/Indel
T-LBL	pediatric	UPN149										SNV/Indel
T-LBL	pediatric	UPN149										SNV/Indel
T-LBL	pediatric	UPN150	9	0	39239013	1	not detected	93.43 %	93.16 %	93.69 %	1964	CNV
T-LBL	pediatric	UPN150										SNV/Indel
T-LBL	pediatric	UPN150										SNV/Indel
T-LBL	pediatric	UPN150										SNV/Indel
T-LBL	pediatric	UPN150										SNV/Indel
T-LBL	pediatric	UPN151	X	52385844	155270560	4	not detected	100.00 %				CNV
T-LBL	pediatric	UPN151	X	0	52385843	1	not detected	93.38 %	93.23 %	93.52 %	2662	CNV
T-LBL	pediatric	UPN151										SNV/Indel
T-LBL	pediatric	UPN151										SNV/Indel
T-LBL	pediatric	UPN151										SNV/Indel
T-LBL	pediatric	UPN151	9	0	38899071	3	not detected	15.99 %	15.32 %	16.67 %	1989	CNV
T-LBL	pediatric	UPN151										SNV/Indel
T-LBL	pediatric	UPN152	9	0	35862688	2	not detected	93.75 %	93.39 %	94.11 %	1777	CNV
T-LBL	pediatric	UPN152	9	35867528	39111928	3	not detected	105.29 %	98.76 %	111.83 %	86	CNV
T-LBL	pediatric	UPN152	10	89311267	90567151	1	not detected	51.31 %	45.44 %	57.18 %	50	CNV
T-LBL	pediatric	UPN152										SNV/Indel
T-LBL	pediatric	UPN152										SNV/Indel
T-LBL	pediatric	UPN152										SNV/Indel
T-LBL	pediatric	UPN152										SNV/Indel
T-LBL	pediatric	UPN152										SNV/Indel
T-LBL	pediatric	UPN153	9	90212822	141213430	3	not detected	39.96 %	39.14 %	40.79 %	2105	CNV
T-LBL	pediatric	UPN153	8	0	146364022	3	not detected	33.05 %	32.62 %	33.48 %	5619	CNV
T-LBL	pediatric	UPN153	11	0	47892937	3	not detected	30.43 %	29.71 %	31.15 %	2124	CNV
T-LBL	pediatric	UPN153										SNV/Indel
T-LBL	pediatric	UPN154	14	21580430	21737296	1	not detected	98.06 %	90.76 %	105.36 %	3	CNV
T-LBL	pediatric	UPN154	9	0	43414113	1	not detected	98.28 %	97.93 %	98.63 %	2006	CNV
T-LBL	pediatric	UPN154	17	37865993	81195210	3	not detected	86.25 %	85.03 %	87.46 %	1756	CNV
T-LBL	pediatric	UPN154	6	29372139	35418228	2	not detected	75.72 %	67.09 %	84.34 %	48	CNV
T-LBL	pediatric	UPN154	9	70731742	141213431	3	not detected	62.88 %	61.94 %	63.82 %	2643	CNV

T-LBL	pediatric	UPN154	NOTCH1 (p.Leu1574Pro)	4	0	52	linear	4	0	3	2	0
T-LBL	pediatric	UPN154	NOTCH1 (p.Leu1585Pro)	4	0	52	linear	4	0	3	2	0
T-LBL	pediatric	UPN154	dup in 20p	5	0	37	linear	5	0	4	3	3
T-LBL	pediatric	UPN154	NOTCH1 (p.Thr2483Ala)	6	0	4	linear	6	0	5	3	2
T-LBL	pediatric	UPN155	FBXW7 (p.Arg479Gln)	1	0	77	linear	1	0	0	2	1
T-LBL	pediatric	UPN155	del in 12p	2	0	70	linear	2	0	1	1	1
T-LBL	pediatric	UPN155	LOH9p	2	0	70	linear	2	0	1	2	2
T-LBL	pediatric	UPN155	del in 14q	2	0	70	linear	2	0	1	1	1
T-LBL	pediatric	UPN155	KRAS (p.Gly12Val)	2	0	70	linear	2	0	1	2	1
T-LBL	pediatric	UPN155	dup20p	3	0	50	linear	3	0	2	3	3
T-LBL	pediatric	UPN155	del in 14q	3	0	50	linear	3	0	2	1	1
T-LBL	pediatric	UPN155	NOTCH1 (p.Leu1585Pro)	3	0	50	linear	3	0	2	2	1
T-LBL	pediatric	UPN155	FBXW7 (p.Ser585Asn)	3	0	50	linear	3	0	2	2	0.5
T-LBL	pediatric	UPN155	USP7 (p.Ala204fs)	4	0	35	linear	4	0	3	2	1
T-LBL	pediatric	UPN155	FBXW7 (p.Trp673*)	5	0	17	linear	5	0	4	2	0.25

T-LBL	pediatric	UPN154	9	139399422	A	G	2028	0	2036	0.00 %	2323	643	2978	21.59 %
T-LBL	pediatric	UPN154	9	139399389	A	G	2002	0	2004	0.00 %	2296	566	2867	19.74 %
T-LBL	pediatric	UPN154												
T-LBL	pediatric	UPN154	9	139390744	T	C	1729	1	1734	0.06 %	2601	41	2651	1.55 %
T-LBL	pediatric	UPN155	4	153247366	C	T	1165	0	1166	0.00 %	847	539	1389	38.80 %
T-LBL	pediatric	UPN155												
T-LBL	pediatric	UPN155												
T-LBL	pediatric	UPN155												
T-LBL	pediatric	UPN155	12	25398284	C	A	1304	0	1305	0.00 %	934	505	1439	35.09 %
T-LBL	pediatric	UPN155												
T-LBL	pediatric	UPN155												
T-LBL	pediatric	UPN155	9	139399389	A	G	1666	3	1675	0.18 %	1026	379	1409	26.90 %
T-LBL	pediatric	UPN155	4	153245437	C	T	1256	0	1259	0.00 %	1233	381	1619	23.53 %
T-LBL	pediatric	UPN155	16	9014211	CTTACGC	CTACCGGCCTTTACTTGTTCGGCTCTCAA	1125	6	1127	0.53 %	807	141	809	17.43 %
T-LBL	pediatric	UPN155	4	153244138	C	T	1431	0	1437	0.00 %	1726	158	1891	8.36 %

T-LBL	pediatric	UPN154										SNV/Indel
T-LBL	pediatric	UPN154										SNV/Indel
T-LBL	pediatric	UPN154	20	0	8981898	3	not detected	36.98 %	35.50 %	38.47 %	523	CNV
T-LBL	pediatric	UPN154										SNV/Indel
T-LBL	pediatric	UPN155										SNV/Indel
T-LBL	pediatric	UPN155	12	11657322	13573768	1	not detected	70.21 %	68.60 %	71.81 %	133	CNV
T-LBL	pediatric	UPN155	9	0	45755225	2	not detected	72.20 %	71.71 %	72.69 %	1853	CNV
T-LBL	pediatric	UPN155	14	22097755	22975384	1	not detected	68.63 %	65.87 %	71.38 %	66	CNV
T-LBL	pediatric	UPN155										SNV/Indel
T-LBL	pediatric	UPN155	20	0	25651492	3	not detected	49.51 %	48.39 %	50.63 %	1345	CNV
T-LBL	pediatric	UPN155	14	87631498	107349540	1	not detected	50.17 %	49.48 %	50.87 %	887	CNV
T-LBL	pediatric	UPN155										SNV/Indel
T-LBL	pediatric	UPN155										SNV/Indel
T-LBL	pediatric	UPN155										SNV/Indel
T-LBL	pediatric	UPN155										SNV/Indel
